# Boron-to-gold transmetalation: a useful platform for gold(iii) cyclometalation

**DOI:** 10.1039/d5sc10046a

**Published:** 2026-08-13

**Authors:** Doriane Chauvin, Jaime Martín, Beatriz Pires Alves, Pau Font, Xavi Ribas, Cristina Nevado

**Affiliations:** a Department of Chemistry, University of Zurich Winterthurerstrasse 190 Zurich CH-8057 Switzerland cristina.nevado@chem.uzh.ch; b Institut de Química Computacional i Catàlisi (IQCC) and Departament de Química, Universitat de Girona, Campus de Montilivi Girona E-17003 Catalonia Spain

## Abstract

Boron-to-gold transmetalation represents a fundamental step in the generation of organogold species, yet its potential for constructing complex architectures remains underexplored. Here, we harness this elementary step to devise a general platform for the synthesis of mono- and bis-cyclometalated gold(iii) complexes. The approach enables access to structurally diverse (N^C)- and (N^C^C)-frameworks, as well as a single example of a (C^N^C)-framework, under remarkably mild, neutral conditions devoid of toxic reagents. Mechanistically, an intriguing B–N dative interaction confirmed by X-ray diffraction analysis underpins the transmetalation process, establishing a new paradigm for the modular construction of cyclometalated gold(iii) species.

## Abstract 2

Boron-to-gold transmetalation represents a fundamental step in the generation of organogold species, yet its potential for constructing complex architectures remains underexplored.

## Graphical abstract

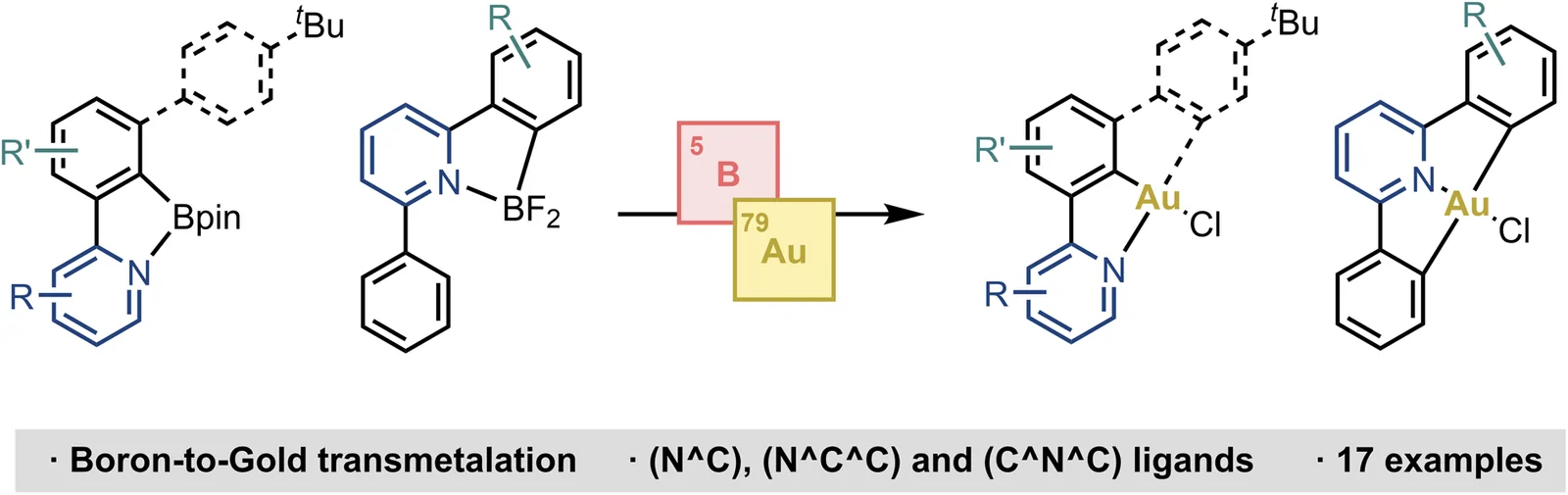

## Introduction

Cyclometalated gold(iii) complexes have found widespread application in biomedical research,^1–5^ materials science,^6–10^ and catalysis,^11–15^ owing to their versatile properties, which include potent cytotoxicity against specific cancer cell lines, tunable photoluminescence, and Lewis acidity. Bi- and tridentate ligands, especially those featuring C and N donor groups, play a pivotal role in tuning the redox properties of this precious metal in order to both stabilize and facilitate detailed investigations of the corresponding complexes.^16–18^ The incorporation of gold onto such templates presents, still today, significant challenges. Classic syntheses of mono- and bis-cyclometalated (C^N) and (C^N^C)gold(iii) complexes typically proceed either *via* transmetalation from organomercury reagents,^19–23^ halide abstraction using stoichiometric amounts of silver salts^24,25^ or direct cycloauration under microwave irradiation or harsh conditions (Fig. 1a, top).^26^ Alternative routes have been devised involving prefunctionalization of ligands with diazonium salts^27,28^ or C(sp^2^)–I bonds,^29^ that undergo oxidative addition onto gold(i) precursors (Fig. 1a, bottom right). Recently, a catalytic approach to directly incorporate gold onto (C^N) ligands leveraging an unprecedent Rh-to-Au transmetalation was also reported by our group (Fig. 1a, bottom left).^30^ Along the same lines, the synthesis of (N^C^C)Au–X complexes^31–36^ relies on microwave conditions to facilitate two consecutive C–H bond functionalizations within a diphenyl-substituted pyridine ligand (Fig. 1b, left).^31^ While this method proved valuable to obtain a diversity of (N^C^C)Au–X species,^37–39^ several factors limited its broad applicability: first and foremost, only symmetrically substituted scaffolds bearing two identical aromatic groups in 1,3-relative position of the central phenyl ring could be attained. Second, high temperatures and small reaction scales with moderate yields were also typical for this protocol. Alternative strategies have also been sought and thus, in 2020, Breher and coworkers showed that transmetalation of a pyridino dibenzostannole with KAuCl_4_ afforded the corresponding (N^C^C)Au–Cl complex in 81% yield (Fig. 1b, right). Besides the production of tin waste in this last step, the method led to a single example.^40^ Our group also reported that oxidative addition of AuI onto the C–C bond of a 1-pyridino-biphenylene system could deliver the corresponding (N^C^C)Au–I complex in 60% yield but the cumbersome preparation of such sophisticated precursor did not allow to significantly expand the scope for further applications.^41^

**Fig. 1:**
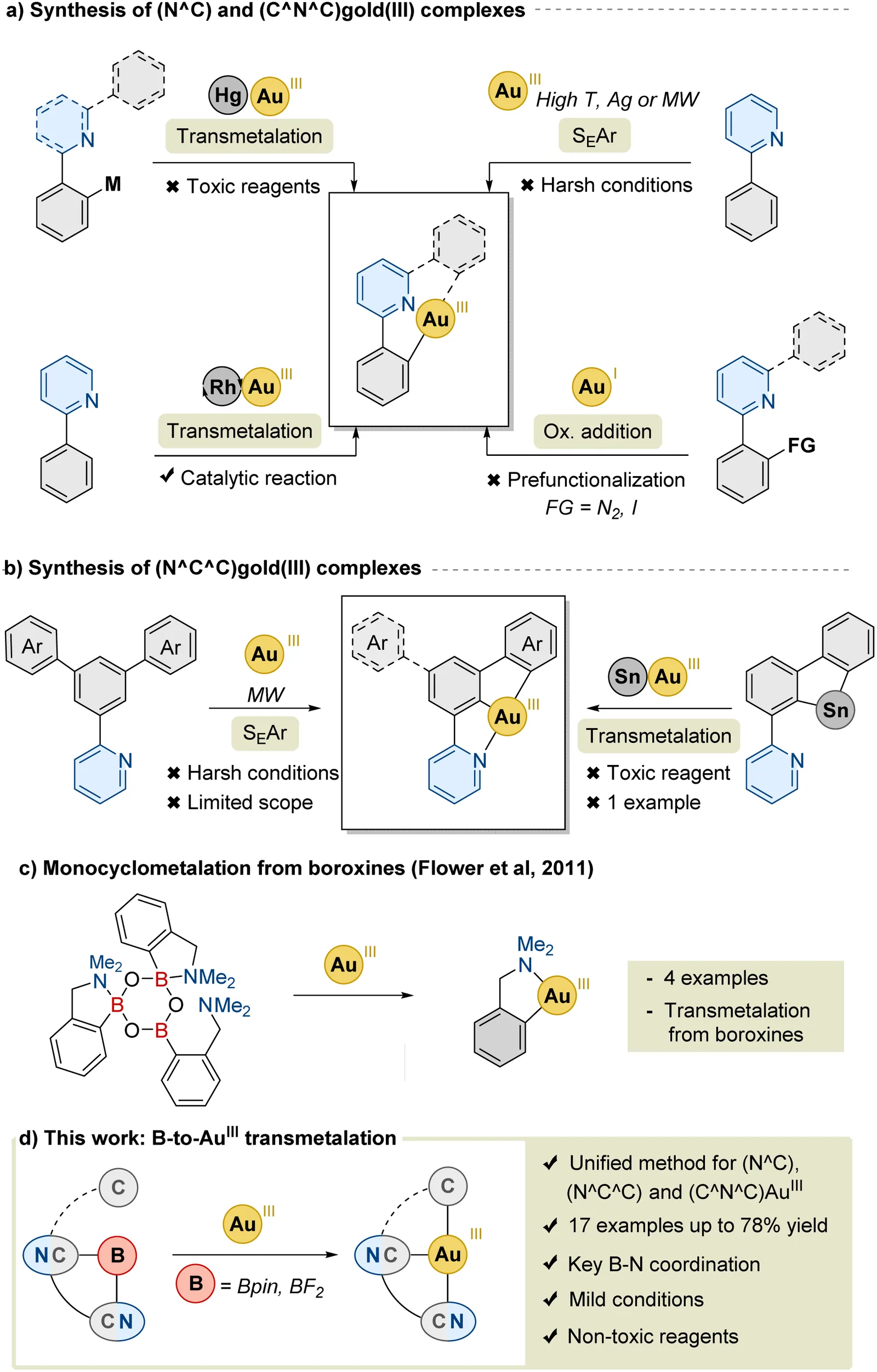
(a and b) Previous strategies to access mono- and bis-cyclometalated (N^C), (C^N^C) and (N^C^C)gold(iii) complexes. (c) Monocyclometalation using boroxine precursor (Flower *et al.*, ref. 67). (d) This work: B-to-Au^III^ transmetalation, a unifying method.

Given the challenges associated with the cycloauration step, a promising solution, lies in the chemoselective functionalization^42,43^ of both existing and newly developed unfunctionalized ligands using a non-toxic, stable, and readily accessible functional group.^44–46^ Boronic acids and esters represent an attractive class of synthetic handles for gold-catalyzed cross-coupling reactions^47–58^ and have demonstrated their ability to efficiently transmetalate with gold(i)^59–79^ and gold(iii)^80–88^ species under mild reaction conditions. To the best of our knowledge though, this reactivity remains underexplored in the cyclometalation realm except for a report of Flower and co-workers featuring the auration of phenylmethanamine derivatives using boroxine precursors (Fig. 1c).^67^ Herein, we present a unified strategy to access mono- and bis-cyclometalated gold(iii) complexes through B-to-Au transmetalation (Fig. 1d). This approach enables the efficient cyclometalation of both established and newly developed (N^C)- and (N^C^C)-scaffolds as well as a single example of a (C^N^C)-framework, under mild, neutral conditions devoid of toxic reagents. X-ray diffraction analysis reveals a key B–N dative interaction in the substrates underpinning the transmetalation step and establishes a new design element towards the efficient construction of complex gold architectures.

## Results and discussion

Our investigation commenced with the preparation of boron-functionalized precursors. Boron pinacolate (Bpin) was chosen as transfer reagent given its straightforward synthesis and ease of purification.^89^ Regioselective bromination of the (N^C^C) template followed by Br–Li exchange and quenching with 2-isopropoxy-4,4,5,5-tetramethyl-1,3,2-dioxaborolane delivered B-containing ligands 3 (Fig. 2a). For ligand 1a the bromination of the central ring with NBS and CuBr^90^ selectively delivered 2a in 75% yield. For the non-symmetrically substituted ligand 1b, bromination with TBABr_3_,^91^ proceeded exclusively at the desired position, furnishing 2b in 82% yield. Lithium–halogen exchange, followed by the addition of ^i^PrOBpin, provided B-containing precursors 3a and 3b in 62 and 61% yield, respectively. Suitable crystals for X-ray diffraction analysis were obtained for these compounds, revealing the coordination of the N-atom to the boronic ester group, with B–N bond distances of 1.728(3) Å for 3a and 1.689(2) Å for 3b, which renders complex 3b yellow-emissive under UV-light (Fig. 2b).^93^ Interestingly, the coordination geometry around the B atom in these ligands adopts a distorted tetrahedral geometry, with the angles involving the B atom ranging from 93.38(17)° (C8–B1–N1) and 119.9(2)° (O2–B1–C8) for 3a, and 94.34(12)° (C21–B1–N10) and 118.42(14)° (O2–B1–C21) for 3b. Further, the second aryl ring in these κ^2^-(N^C)-Bpin precursors is twisted due to the steric hindrance imposed by the Bpin moiety, as reflected by the torsion angles (C8–C9–C23–C24 67.7(3)° for 3a, and C21–C20–C22–C23 −55.6(2)° for 3b).

**Fig. 2:**
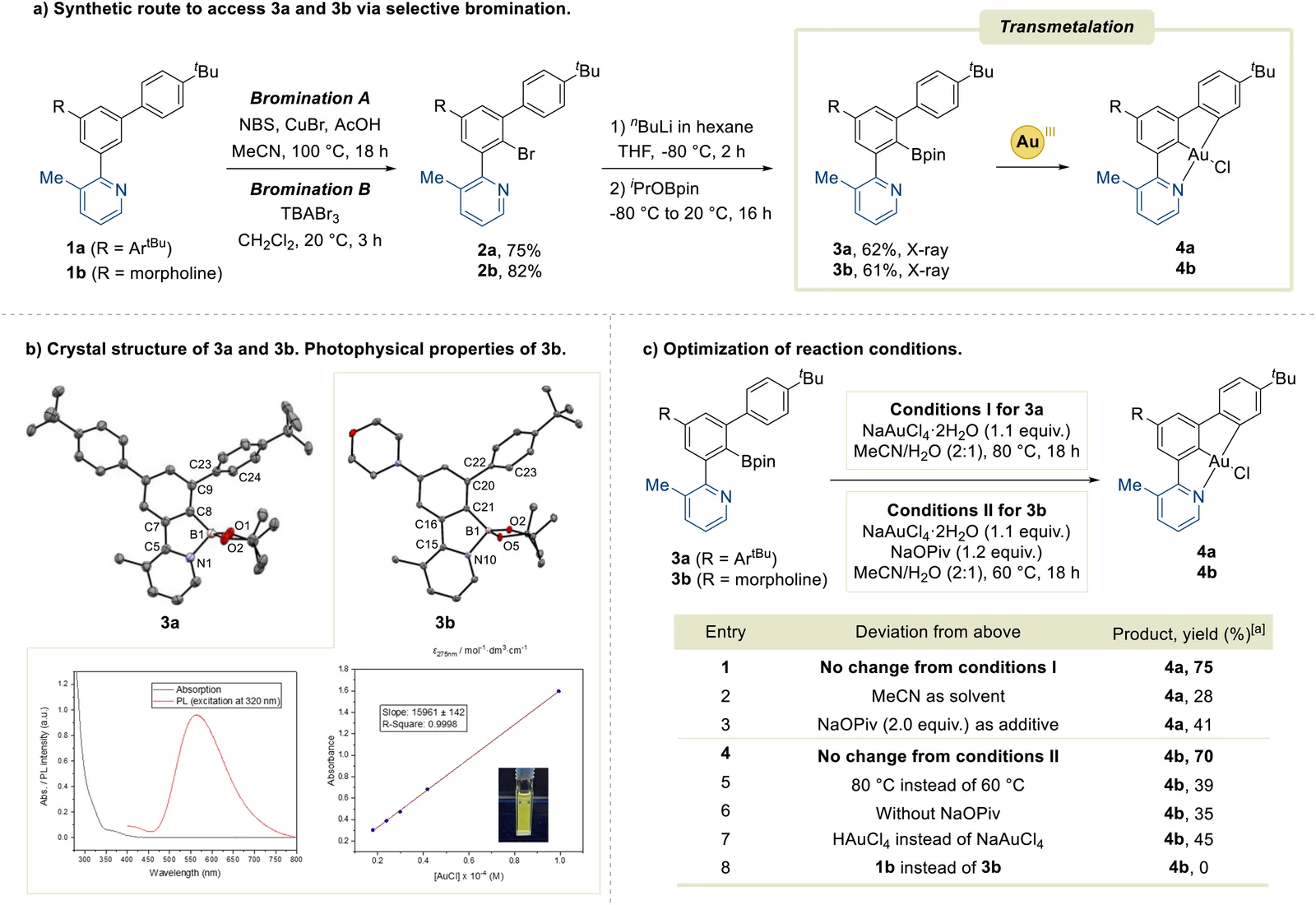
(a) Synthesis of 3a and 3b. (b) ORTEP representation of 3a and 3b with 50% probability ellipsoids. Hydrogen atoms are omitted for clarity. Selected distances (Å) in 3a: B1–O1 1.411(3), B1–O2 1.434(3), B1–N1 1.728(3), B1–C8 1.625(3); and 3b: B1–O2 1.441(2), B1–O5 1.451(2), B1–N10 1.689(2), B1–C21 1.642(2). Selected angles (deg.) in 3a: O2–B1–C8 119.9(2), C8–B1–N1 93.38(17); and 3b: O2–B1–C21 118.42(14), C21–B1–N10 94.34(12). The smaller and the larger angles of the distorted tetrahedral geometry around the B center are given. Selected torsion angles (deg.) in 3a: N1–C5–C7–C8 4.6(3), C8–C9–C23–C24 67.7(3); and 3b: N10–C15–C16–C21 0.58(19), C21–C20–C22–C23 −55.6(2). Highest emission peak and molar absorption coefficient *ε*_275nm_ of compound 3b measured in degassed CH_2_Cl_2_ at 298 K (inset: picture taken in the dark under 365 nm lamp). (c) Optimization of reaction conditions to obtain 4a/4b from 3a/3b. ^[a]^The yield was determined by ^1^H NMR with mesitylene (for 4a) and (1,3,5)-trimethoxybenzene (for 4b) as internal standards, respectively.

With compounds 3a and 3b in hand, we turned our attention to the B-to-Au transmetalation step. After an extensive evaluation of different parameters (gold source, solvent, temperature, concentration, additives)^94^ the bis-cyclometalated complex 4a could be successfully obtained in 75% yield by reaction of 3a with NaAuCl_4_·2H_2_O (1.1 equiv.) in a 2 : 1 mixture of acetonitrile and water at 80 °C after 18 h (Fig. 2c, entry 1). The binary solvent system was essential for the success of this transformation, as a substantial decrease in yield was observed with pure acetonitrile (Fig. 2c, entry 2). Interestingly, the use of an external base (NaOPiv)^94^ had a negative impact on the reaction efficiency delivering 4a in 41% yield (Fig. 2c, entry 3). In parallel, the optimal conditions were re-evaluated in the synthesis of the non-symmetrically substituted complex 4b. The reaction of 3b with NaOPiv (1.2 equiv.) and NaAuCl_4_·2H_2_O (1.1 equiv.) furnished the desired gold(iii) complex 4b in a comparable 70% yield (Fig. 2c, entry 4). Both, lowering the temperature from 80 to 60 °C and the use of an external base were crucial to prevent decomposition of the ligand and maintain reaction efficiency (Fig. 2c, entries 5 and 6). A decrease in yield (45%) was observed when HAuCl_4_·3H_2_O was employed (Fig. 2c, entry 7), further confirming the negative impact of acid in the reaction outcome. Control experiments confirmed the pivotal role of boron, as no traces of 4b were detected when Bpin-3b was replaced with H-1b (Fig. 2c, entry 8).

With the optimized conditions in hand, we sought to explore the generality of this transformation (Fig. 3). Following a similar synthetic route as for 3a and 3b, a diverse array of non-symmetrically substituted Bpin-functionalized (N^C^C) and (N^C) precursors 3c–p were prepared in good to excellent yields.^94^ Suitable crystals for X-ray diffraction were successfully obtained for 3e, 3i, and 3m, all of which exhibited the same B–N coordination observed in 3a and 3b.^92^ Piperazine, piperidine dioxolane and 4,4-difluropiperidine groups were well tolerated, providing the corresponding bis-cyclometalated gold(iii) complexes 4c–e in up to 76% isolated yield. For compound 4d a modest yield of 32% was obtained due to partial decomposition of the acetal moiety. To our delight, linear aliphatic and aromatic amines could also be successfully incorporated on the ligand. Complexes 4f–i were obtained in moderate to high yields, and exhibited an increased solubility compared to cyclic amines. Variations on the pyridine ring were also explored: the incorporation of an unprotected alcohol was tolerated providing complex 4j in 67% yield. The bis-cyclometalated structure of complex 4j was unequivocally confirmed by X-ray diffraction analysis.^92^ The structural parameters around the Au atom in 4j (Au1–Cl1 2.3801(9) Å, Au1–N1 2.135(3) Å, Au1–C7 1.969(3) Å, Au1–C13 2.035(4) Å) closely resemble those of previously reported symmetrically substituted (N^C^C)Au–Cl complexes,^37–41^ showcasing that the morpholine substituent has a minimal influence on the coordination sphere of the Au center. Interestingly, along complex 4j, we identified and crystallographically characterized mono-cyclometalated dimeric complex (N^C^C)_2_Au(iii)Cl 4j′, formed as minor side product during the reaction. This compound is proposed to stem from the double transmetalation of a single gold atom with two Bpin-precursors. Consequently, the metal center adopts a κ^2^-(N^C) coordination mode with one ligand and a κ^1^-C mode with the second ligand. The Au–C bond, which lies in the same axis as the morpholine group in the (N^C) ligand, is significantly longer in 4j′ than in 4j (2.056(3) *vs.* 1.969(3) Å) and the second aryl fragment is twisted, preventing the formation of a Au1–C39 bond in 4j′, as evidenced by the torsion angles (C7–C8–C12–C13 0.1(5)° for 4j, C33–C34–C38–C39 −57.7(7)° for 4j′). The second (N^C^C) ligand in 4j′ is coordinated to the Au center exclusively by its central aryl ring (Au1–C7 2.009(5) Å), with its second aryl ring twisted (torsion angle C7–C8–C12–C13 −56.8(7)°). The pyridine ring of this second (N^C^C) ligand lies perpendicular to the (N^C) metallacycle plane (torsion angle C33–Au1–C7–C6 112.9(4)°) but exhibits a long Au1–N1 distance of 2.765(4) Å, consistent with gold's reluctance to adopt a five-coordinate geometry.^11,17,87^ Alternative N-donor rings were also tested. Both isoquinoline and benzothiazole groups were compatible with the reaction conditions, furnishing 4k and 4l in 51 and 28% yield, respectively. The compatibility of this protocol with bidentate (N^C) ligands was investigated next. A series of 2-phenylpyridine Bpin-precursors 3m–p, were successfully transformed into the corresponding cyclometalated gold(iii) 4m–p with high efficiency (Fig. 3). Substitution at the 3- and 4-positions of the phenyl ring (*e.g.* morpholino, ^*t*^Bu, Ph), as well as on the pyridine moiety (OMe), proved compatible with the standard reaction conditions (Fig. 3). The structure of these complexes was further confirmed by X-ray diffraction analysis of complex 4n.^92^ Encouraged by the successful results obtained in the synthesis of (N^C^C) and (N^C)gold(iii) complexes, we set out to apply the B-to-Au transmetalation strategy to the synthesis of (C^N^C)Au complexes. To this end, (C^N^C) ligand 3q was prepared using the previously described bromination/borylation protocol. When subjected to the previously established transmetalation conditions, complete protodeborylation was observed, yielding only the protonated ligand 1q (Fig. 4c, entries 1–3). X-ray diffraction analysis of 3q^92^ reveals that the Bpin moiety is oriented opposite to the pyridine nitrogen (torsion angle N2–C11–C9–C8 138.43(13)°) in contrast to ligand 3b (torsion angle N10–C15–C16–C21 0.58(19)°), thus preventing the formation of a dative B–N bond (Fig. 4b). We hypothesized that the steric hindrance imposed by the Bpin group in the (C^N^C) system hampers the interaction between the B and the N atoms, complicating the transmetalation from a non-activated sp^2^-configured boron to the gold(iii) center. To confirm this hypothesis, we sought to reduce the size of the boron substituents. Among others, BF_*x*_ represents a promising candidate due to the small size of fluorine (64 pm for single bond radii)^95^ and its well-established applicability in cross-coupling reactions.^96^ Ligand 3r was prepared by treating the *in situ* prepared boronic ester with KHF_2_. The X-ray structure of 3r^92^ reveals a B–N bond distance of 1.660(2) Å, comparable to that found in a 2-(2-(BF_2_)phenyl)pyridine (1.594(14) and 1.597(15) Å),^97^ and a tetrahedral geometry around the B atom (F1–B1–C7 117.22(17)°, C7–B1–N1 97.20(13)°) (Fig. 4b). In addition, 3r displayed yellow emission under UV-light, consistent with the luminescent properties of four-coordinate organoboron compounds based on (N^C) ligands,^93^ whereas 3q was non-emissive.

**Fig. 3:**
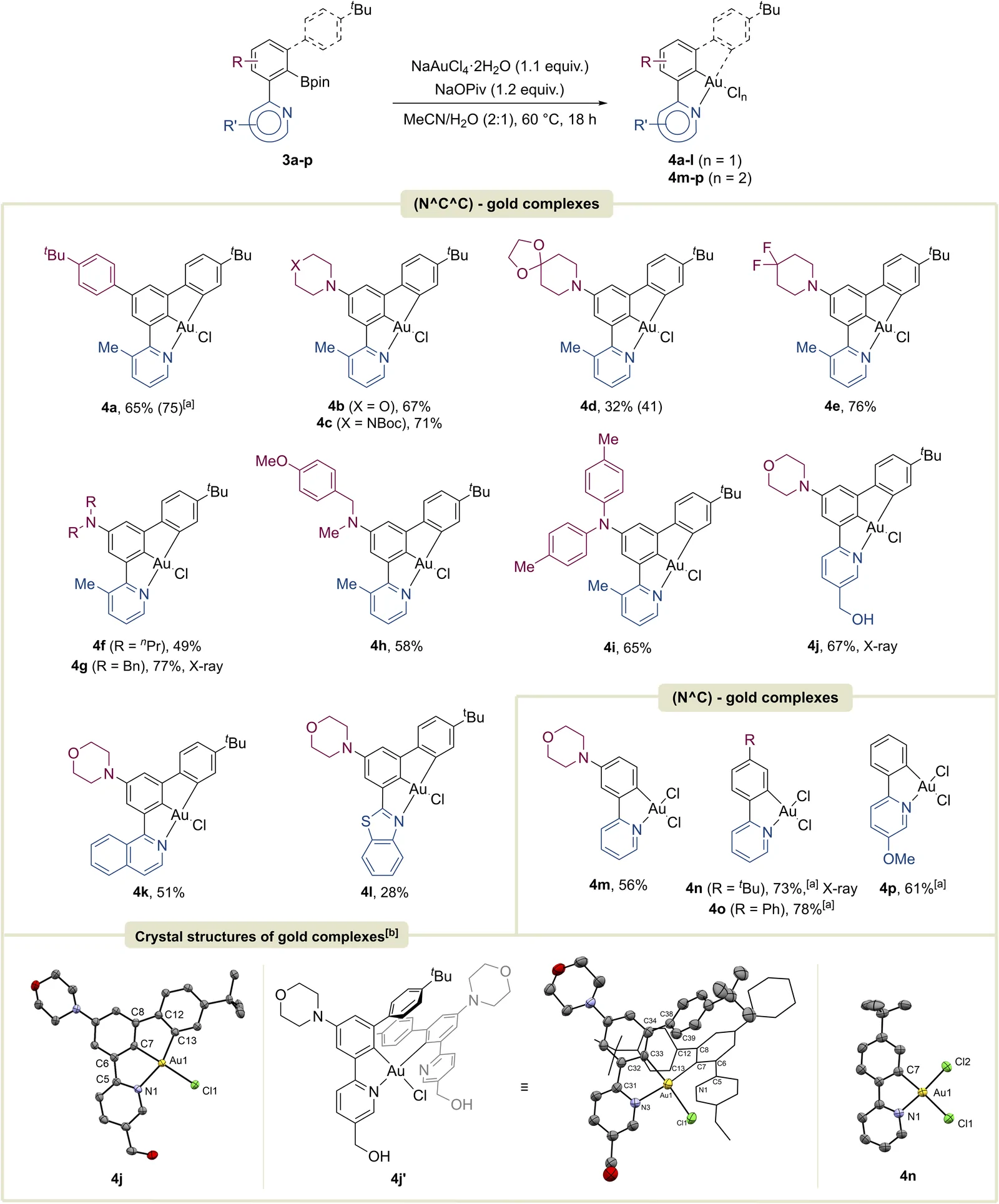
Substrate scope for (N^C^C)AuCl 4a–l and (N^C)AuCl_2_4m–p. Isolated yields are given. ^1^H NMR yield is given in brackets and was determined with 1,3,5-trimethoxybenzene as internal standard. ^[a]^Without NaOPiv, 80 °C, 18 h. ^[b]^ORTEP representation of 4j (one of the two molecules in the asymmetric unit), 4j′ and 4n with 50% probability ellipsoids. Solvent molecules and hydrogen atoms are omitted for clarity. Selected distances (Å) in 4j: Au1–Cl1 2.3801(9), Au1–N1 2.135(3), Au1–C7 1.969(3), Au1–C13 2.035(4); 4j′: Au1–C7 2.009(5), Au1–C33 2.056(4), Au1–Cl1 2.3839(12), Au1–N3 2.105(4); and 4n: Au1–Cl1 2.3776(11), Au1–Cl2 2.2700(11), Au1–N1 2042(4), Au1–C7 2.030(4). Selected angles (deg.) in 4j: N1–Au1–Cl1 99.70(9), C7–Au1–Cl1 177.78(11), C13–Au1–N1 159.96(14); 4j′: C7–Au1–N3 166.53(16), C33–Au1–Cl1 171.40(12), C33–Au1–N3 80.86(11); and 4n: C7–Au1–Cl1 175.99(13), N1–Au1–Cl2 174.94(10), C7–Au1–N1 81.24(15). Selected torsion angles (deg.) in 4j: N1–C5–C6–C7 1.8(4), C7–C8–C12–C13 0.1(5); and 4j′: N3–C31–C32–C33 4.5(7), C33–C34–C38–C39 −57.7(7), N1–C5–C6–C7 −17.0(6), C7–C8–C12–C13 −56.8(7), C33–Au1–C7–C6 112.9(4).

**Fig. 4:**
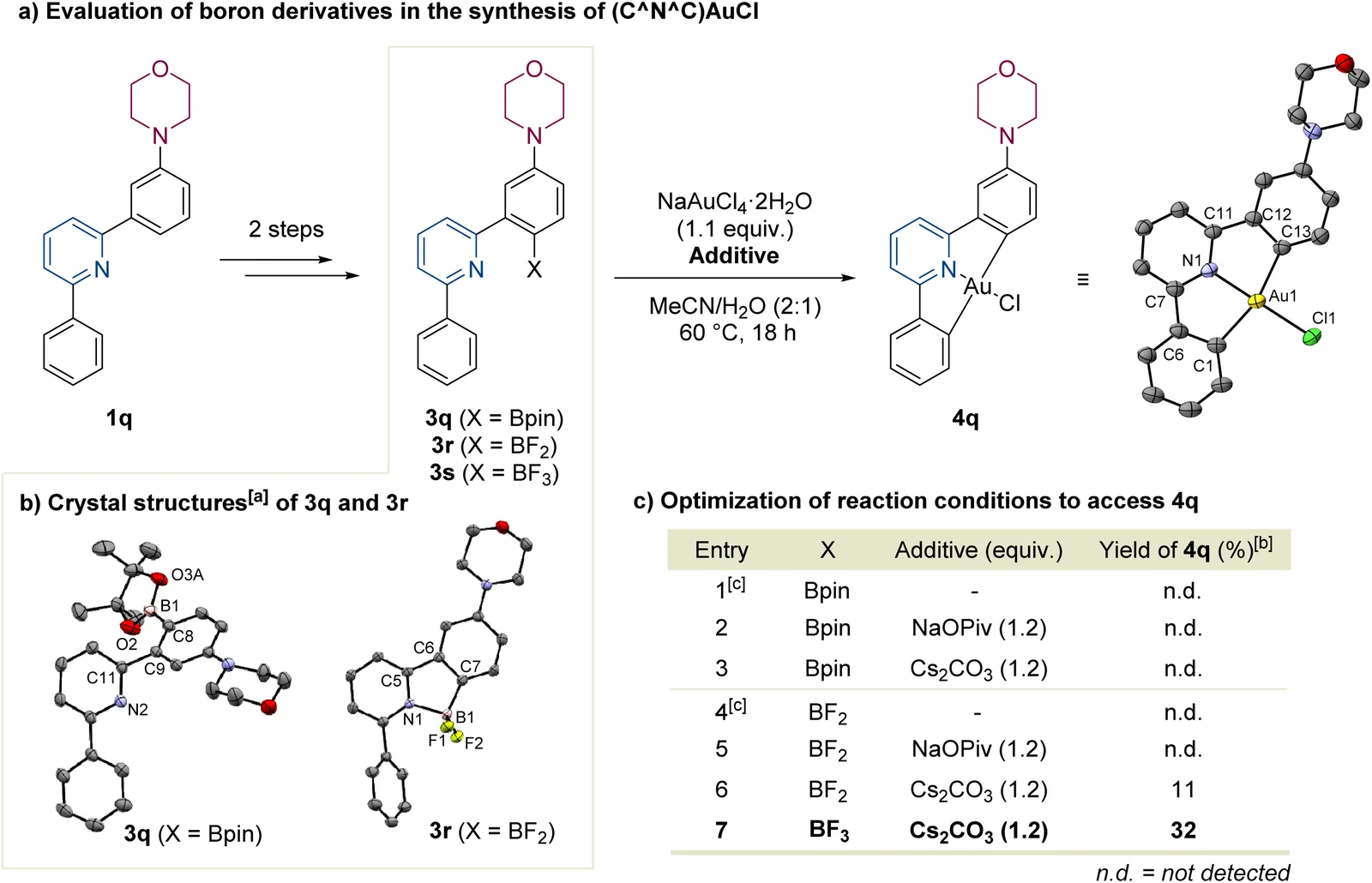
(a–c) Optimization and synthesis of (C^N^C)AuCl 4q. ^[a]^ORTEP representation of 3q and 3r with 50% probability ellipsoids. Solvent molecules and hydrogen atoms are omitted for clarity. Selected distances (Å) in 3q: B1–O2 1.3315(19), B1–O3A 1.381(3); 3r: B1–F1 1.393(2), B1–F2 1.3823(2), B1–C7 1.598(3), B1–N1 1.660(2); and 4q: Au1–Cl1 2.2809(10), Au–C1 2.076(4), Au1–N1 1.981(3), Au–C13 2.067(4). Selected angles (deg.) in 3q: O2–B1–C8 125.55(13), O3A–B1–C8 121.45(15), O2–B1–O3A 11.60(15); 3r: F1–B1–C7 117.22(17), C7–B1–N1 97.20(13); and 4q: C1–Au1–Cl1 98.96(12), C1–Au1–C13 162.73(17), N1–Au1–Cl1 179.27(9). Selected torsion angles (deg.) in 3q: C8–C9–C11–N2 138.43(13); 3r: N1–C5–C6–C7 −3.8(2); and 4q C1–C6–C7–N1 0.2(5), N1–C11–C12–C13 2.5(5). ^[b]^Isolated yield. ^[c]^80 °C instead of 60 °C.

Unfortunately, 3r did not undergo transmetalation under the standard conditions (Fig. 4c, entries 4 and 5). However, when Cs_2_CO_3_ was used as base instead of NaOPiv, small amounts of the desired complex 4q could be detected. Finally, 4q could be isolated in 32% yield using the corresponding trifluoroborate salt 3s (18% overall yield from ligand 1q)^87^ (Fig. 4c, entries 6 and 7). Likely, in this case 3s behaves as a reservoir, being transformed into more labile 3r thus enabling B–N coordination (not possible in the case of the boronate salt directly) and subsequent transmetalation. Our strategy favorably compares with the two-step yields reported for the mercury route for the same ligand.^20,98,99^ The structure of 4q was confirmed by X-ray diffraction analysis, which reveals a short Au–Cl bond distance of 2.2809(10) Å, comparable to other (C^N^C)gold(iii) complexes,^21–23,28,100–102^ in line with the weak trans influence of the pyridine. To the best of our knowledge, transmetalation between fluoroboronic compounds and gold(iii) has not been previously reported. These findings highlight the critical role of the B–N dative bond for transmetalation.

## Conclusions

In conclusion, we report a novel and environmentally friendly strategy for the synthesis of mono- and bis-cyclometalated gold(iii) complexes, enabled by a boron-mediated transmetalation applicable to (N^C), (N^C^C), and (C^N^C) ligands. Crystallographic data revealed the pivotal role of an internal B–N dative bond in stabilizing these precursors and promoting smooth transmetalation under mild conditions. By harnessing the unique synergy between boron and Au(iii), this methodology provides a sustainable alternative to mercury and unlocks a modular, broadly applicable route to cyclometalated gold(iii) architectures. Beyond avoiding harsh reagents, this paradigm expands the synthetic toolbox for gold chemistry, offering direct opportunities for tailored designs with implications in catalysis, optoelectronic materials, and medicinal applications.

## Author contributions

Investigation and methodology: D. C., B. P. A., and P. F. Supervision: J. M., X. R., and C. N. Conceptualisation: J. M. and C. N. Writing – original draft: D. C., J. M. and C. N., with contributions from all the authors.

## Conflicts of interest

There are no conflicts to declare.

## Note added after first publication

This article replaces the version published on 14th August 2026, correcting the reference list order for ref. 92–102.

## Supplementary Material

SC-017-D5SC10046A-s001

SC-017-D5SC10046A-s002

## Data Availability

CCDC 2483368 (for 3a), 2483369 (for 3b), 2483374 (for 3e), 2483376 (for 3i), 2483378 (for 3m), 2483422 (for 3q), 2483381 (for 3r), 2483405 (for 3t), 2492591 (for 4g), 2483390 (for 4j), 2483491 (for 4j′), 2483482 (for 4n) and 2483411 (for 4q) contain the supplementary crystallographic data for this paper.^92*a*–*m*^ Supplementary information (SI): experimental details and characterization of new compounds. See DOI: https://doi.org/10.1039/d5sc10046a.
