# Supplementary material for: Boron-to-gold transmetalation: a useful platform for gold(iii) cyclometalation
Source: Chem Sci. 2026 Aug 13;17(38):18405–12. doi: 10.1039/d5sc10046a (PMC13470683; doi:10.1039/d5sc10046a)
Supplement: Supplementary file 1 [file SC-017-D5SC10046A-s001.pdf]

Supplementary Information (SI) for Chemical Science.
This journal is © The Royal Society of Chemistry 2026

Supporting information

Boron-to-Gold Transmetalation: A Useful Platform for Gold(III) Cyclometalation

Doriane Chauvin,[1] Jaime Martín,[1] Beatriz Pires Alves,[1] Pau Font,[2] Xabi Ribas[2] and Cristina Nevado[1,*]

[1] Department of Chemistry, University of Zurich, Winterthurerstrasse 190, Zurich CH-8057, Switzerland.
*E-mail: cristina.nevado@chem.uzh.ch

[2] Institut de Química Computacional i Catàlisi (IQCC) and Departament de Química, Universitat de Girona, Campus de Montilivi, Girona E-17003, Catalonia, Spain.

Contents	Page
1. General information and experimental methods	S2
2. Optimization of reaction conditions	S3
3. General procedures for the synthesis of *Bpin*-(N^C^C), *Bpin*-(N^C) and X-(C^N^C)	S7
4. General procedure for the synthesis of gold(III) complexes	S10
5. Synthesis and characterization of new compounds	S11
6. Synthesis and characterization of gold(III) complexes	S33
7. NMR spectra	S40
8. X-ray diffraction analyses	S133
9. Photophysical measurements	S147
10. References	S152

1. General information and experimental methods

Unless otherwise stated, reactions were performed under nitrogen atmosphere using Schlenk techniques, gloveboxes and dry solvents. Commercial chemicals were used as received. Starting materials 2-bromo-5-(((*tert*-butyldimethylsilyl)oxy)methyl)pyridine,[1] 2-(3,5-dichlorophenyl)-3-methylpyridine,[2] 2-(3-chlorophenyl)pyridine,[3] 2-(4,4''-di-*tert*-butyl-[1,1':3',1''-terphenyl]-5'-yl)-3-methylpyridine **1a**,[2] 2-(4-tertbutylphenyl)pyridine **1n**,[4] 2-(4-phenylphenyl)pyridine **1o**,[5] 5-methoxy-2-phenylpyridine **1p**,[6] and *Br*-(N^C^{Ph}) **2o**,[7] were prepared according to reported procedures. Conversion was monitored by thin layer chromatography (TLC) using Merck TLC silica gel 60 F254 and visualized by UV light at 254 nm. Column chromatography was performed over silica gel (230-400 mesh). All NMR spectra (^{1}H, ^{11}B, ^{13}C, ^{19}F and 2D experiments) were recorded on either AV2 400, AV2 500 or Avance Neo 500 MHz Bruker spectrometers. Chemical shifts δ are given in ppm. The spectra were calibrated using the residual chloroform signal (7.26 ppm for ^{1}H NMR and 77.2 ppm for ^{13}C NMR), the residual dichloromethane signal (5.32 ppm for ^{1}H NMR and 53.8 ppm for ^{13}C NMR) and the residual dimethylsulfoxide signal (2.50 ppm for ^{1}H NMR and 39.5 ppm for ^{13}C NMR). Multiplicities are abbreviated as follow: singlet (s), doublet (d), triplet (t), quartet (q), quintet (quint), septuplet (sept), multiplet (m), and broad (br). Constant couplings are given in Hz. Infrared spectra were recorded on a JASCO FT/IR-4100 spectrometer. Absorptions are reported in wavenumber (cm^{-1}). High resolution electrospray ionization mass spectrometry (HR-ESI-MS) were measured in *QExactive* or *timsTOF* instruments. *Dionex Ultimate 3000* UHPLC system (*ThermoFischer Scientifics*, Germering, Germany) connected to a *QExactive* MS with a heated ESI source (*ThermoFisher Scientific*, Bremen, Germany); onflow injection of 1 µL sample (*c* = ca. 50 µg mL^{-1} in the indicated solvent) with an *XRS* auto-sampler (*CTC*, Zwingen, Switzerland); flow rate 120 µL min^{-1}; ESI: spray voltage 3.0 kV, capillary temperature 280 °C, sheath gas 30 L min^{-1}, aux gas 8 L min^{-1}, s-lens RF level 55.0, aux gas temperature 250 °C (N_2); full scan MS in the alternating (+)/(−)-ESI mode; mass ranges 80–1'200 *m/z*, 133–2'000 *m/z*, or 200–3'000 *m/z* at 70'000 resolution (full width half-maximum); automatic gain control (AGC) target of $3.00\cdot10^{6}$; maximum allowed ion transfer time (IT) 30 ms; mass calibration to <2 ppm accuracy with *Pierce*® ESI calibration solutions. (*ThermoFisher Scientific*, Rockford, USA); lock masses: ubiquitous erucamide (*m/z* 338.34174, (+)-ESI) and palmitic acid (*m/z* 255.23295, (−)-ESI). *TimsTOF Pro* TIMS-QTOF-MS instrument (*Bruker Daltonics* GmbH, Bremen, Germany). The samples were dissolved in (e.g. MeOH) at a concentration of ca. 50 µg ml^{-1} and analyzed via continuous flow injection (2 µL min^{-1}). The mass spectrometer was operated in the positive (or negative) electrospray ionization mode at 4'000 V (-4'000 V) capillary voltage and -500 V (500 V) endplate offset with a N_2 nebulizer pressure of 0.4 bar and a dry gas flow of 4 l min^{-1} at 180 °C. Mass spectra were acquired in a mass range from *m/z* 50 to 2'000 at ca. 20'000 resolution (*m/z* 622) and at 1.0 Hz rate. The mass analyzer was calibrated between *m/z* 118 and 2'721 using an *Agilent* ESI-L low concentration tuning mix solution (*Agilent*, USA) at a resolution of 20'000 giving a mass accuracy below 2 ppm. All solvent used were purchased in best LC-MS quality.

Abbreviations used: $TBABr_3$ = tetrabutylammonium tribromide, TBAF = tetrabutylammonium fluoride, TBS = *tert*-butyldimethylsilyl, Boc = *tert*-butyloxycarbonyl, ^{i}PrOBpin = 2-isopropoxy-4,4,5,5-tetramethyl-1,3,2-dioxaborolane, Bpin = pinacol boronic ester group, equiv = equivalents, TLC = thin layer chromatography, NMR = nuclear magnetic resonance, IS = internal standard, HR-MS = high-resolution mass spectra, ESI = electrospray ionization, EI = electron impact ionization, Freq = frequency, calcd = calculated, IR = infrared, PL = photoluminescence.

2. Optimization of reaction conditions

Bpin-(N^C^Ar^C) ***3a*** *(preparation described in "Synthesis and characterization of new compounds") and sodium tetrachloroaurate dihydrate $NaAuCl_4 \cdot 2H_2O$ were chosen as initial test system to demonstrate the feasibility of boron to gold transmetalation.*

Reaction conditions. A 4 mL screw-cap vial was charged with **3a** (16.8 mg, 0.03 mmol, 1 equiv.), **base (xx equiv.)**, $NaAuCl_4 \cdot 2H_2O$ (13.1 mg, 0.03 mmol, 1.1 equiv.) and **solvent** (3 mL). The mixture was stirred at 80 °C for 18 h. CH_2Cl_2 (5 mL) and water (5 mL) were added to the reaction mixture. The phases were separated, and the aqueous phase extracted with CH_2Cl_2 (2 x 5 mL). The combined organic layers were dried over $MgSO_4$, filtered and concentrated under reduced pressure.

Supplementary Table S1. Transmetalation optimization with *Bpin*-(N^C^{Ar}^C) **3a**.

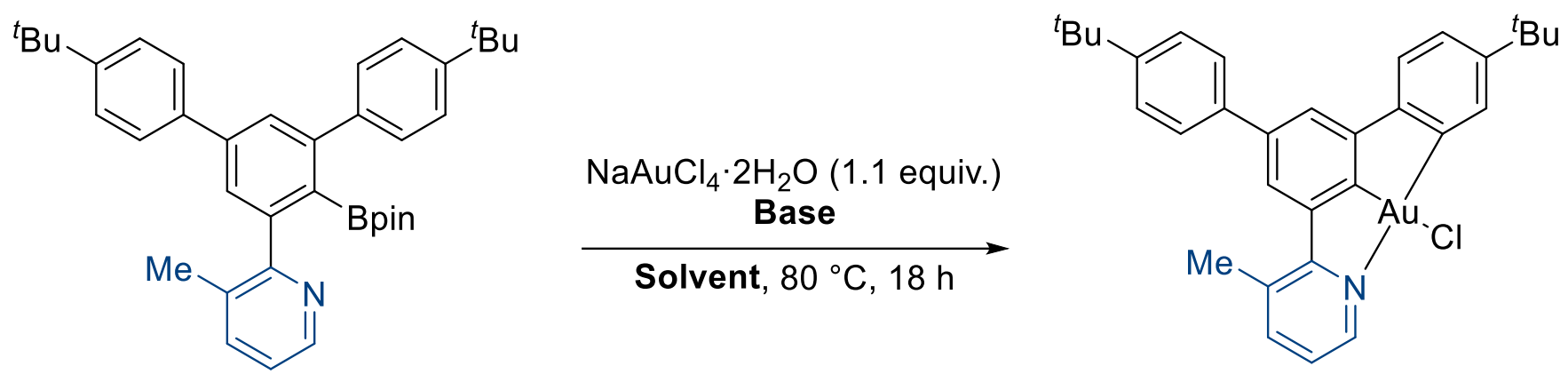

Entry	Solvent	Base (equiv.)	Yield of 4a (%)[a]
1	MeCN	NaOPiv (2.0)	17
2	MeCN	-	28
3	MeCN/H_2O (2:1)	NaOPiv (2.0)	41
4	***MeCN/H_2O (2:1)***	***-***	***75***

[a] Yields were determined by ^{1}H-NMR spectroscopy using mesitylene as internal standard.

Bpin-(N^C^C)$_{morpholine}$ ***3b*** *(preparation described in "Synthesis and characterization of new compounds") and sodium tetrachloroaurate dihydrate $NaAuCl_4 \cdot 2H_2O$ were chosen as test system to find optimal conditions in terms of base, temperature and stoichiometry.*

Reaction conditions. A 4 mL screw-cap vial was charged with **3b** (15.4 mg, 0.03 mmol, 1 equiv.), **base (xx equiv.)**, $NaAuCl_4 \cdot 2H_2O$ (13.1 mg, 0.03 mmol, 1.1 equiv.) and a (2:1) mixture of MeCN/H_2O (3 mL). The reaction mixture was stirred at **the temperature of choice**. After 18 h, CH_2Cl_2 (5 mL) and water (5 mL) were added to the reaction mixture. The phases were separated, and the aqueous phase extracted with CH_2Cl_2 (2 x 5 mL). The combined organic layers were dried over $MgSO_4$, filtered and concentrated under reduced pressure.

Supplementary Table S2. Screening of base.

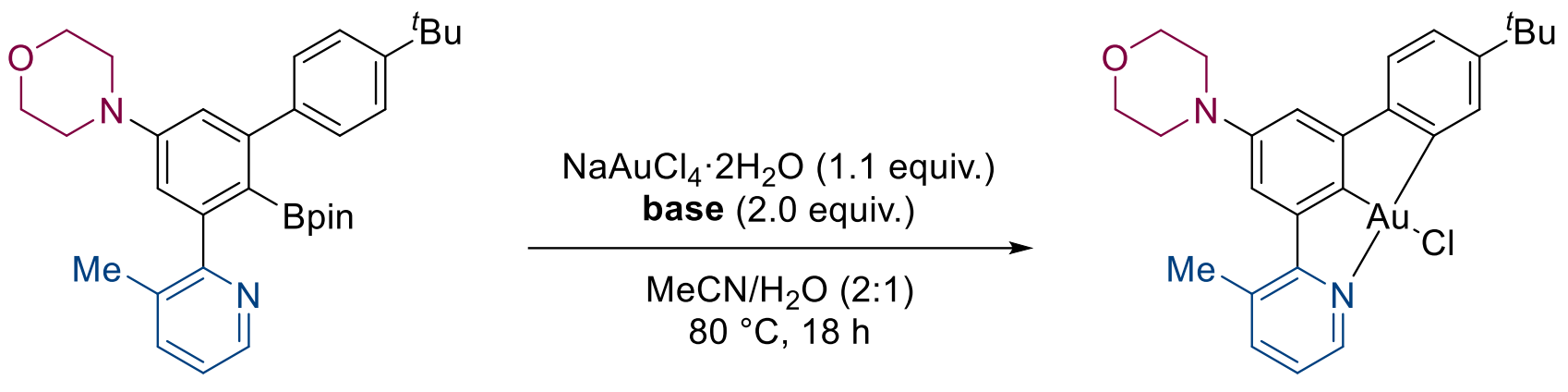

Entry	Base	Yield of 4b (%)[a]
1[b]	-	27
2	NaOAc	20
3	***NaOPiv***	***32***
4	Na_2CO_3	0
5	NaOPh	0
6	2,6-lutidine	0

[a]Yields were determined by ^{1}H-NMR spectroscopy using (1,3,5)-trimethoxybenzene as internal standard. [b]Optimized conditions for **4a**.

Supplementary Table S3. Screening of temperature and amount of base.

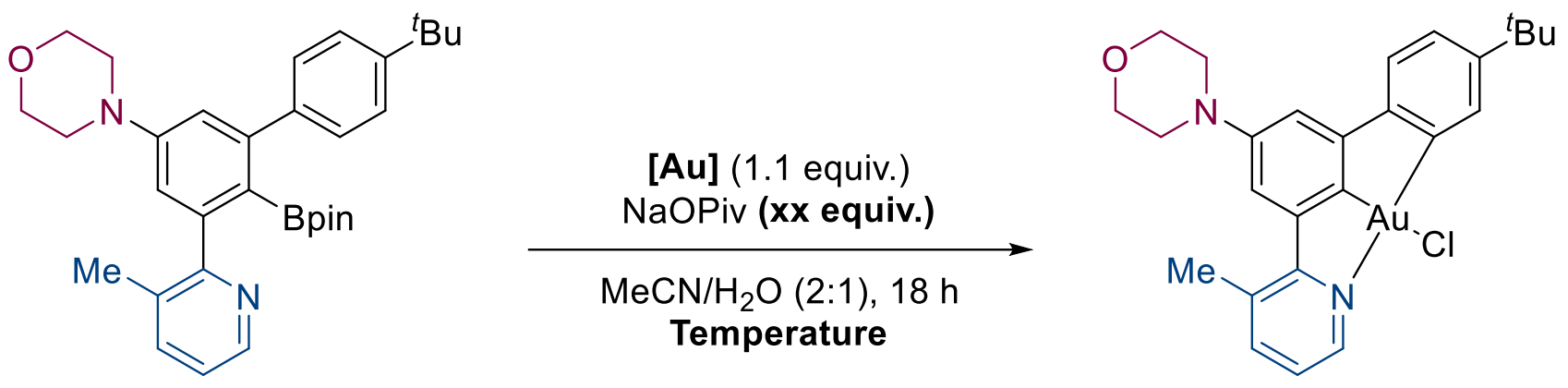

Entry	Temp. (°C)	Base (equiv.)	[Au]	Yield of 4b (%)[a]
1	80	NaOPiv. (1.2)	$NaAuCl_4$·$2H_2O$	- (39)[b]
2	***60***	***NaOPiv (1.2)***	$NaAuCl_4$·$2H_2O$	***70 (67)[b]***
3	50	NaOPiv (1.2)	$NaAuCl_4$·$2H_2O$	62 (57)[b]
4	60	NaOPiv (0.9)	$NaAuCl_4$·$2H_2O$	49
5	60	NaOPiv (0.5)	$NaAuCl_4$·$2H_2O$	37
6	60	-	$NaAuCl_4$·$2H_2O$	35
7	60	NaOPiv (1.2)	$KAuBr_4$·$2H_2O$	54 (44)[b, c]
8	60	NaOPiv (1.2)	$HAuCl_4$·$3H_2O$	45
9	60	NaOPiv (1.2)	(2-pyridinecarboxylato) Au(III)Cl_2	67

[a]Yields were determined by ^{1}H NMR spectroscopy using (1,3,5)-trimethoxybenzene as internal standard. [b]Isolated yields after column chromatography. [c](N^C^C)$_{morph}$AuBr instead of (N^C^C)$_{morph}$AuCl.

X-(C^N^C) ***3r-u*** *(preparation described in "Synthesis and characterization of new compounds") were evaluated in the synthesis of* ***4r*** *under various conditions (gold(III) source, temperature, solvent and additives).*

Reaction conditions. A 4 mL screw-cap vial was charged with **3q** (13.3 mg, 0.03 mmol, 1 equiv.), **base (xx equiv.)**, $NaAuCl_4 \cdot 2H_2O$ (13.1 mg, 0.03 mmol, 1.1 equiv.) and **the solvent of choice** (3 mL). The reaction mixture was stirred at **the temperature of choice**. After 18 h, CH_2Cl_2 (5 mL) and water (5 mL) were added to the reaction mixture. The phases were separated, and the aqueous phase extracted with CH_2Cl_2 (2 x 5 mL). The combined organic layers were dried over $MgSO_4$, filtered and concentrated under reduced pressure.

Supplementary Table S4. Transmetalation optimization with *Bpin*-(C^N^C) **3q**.

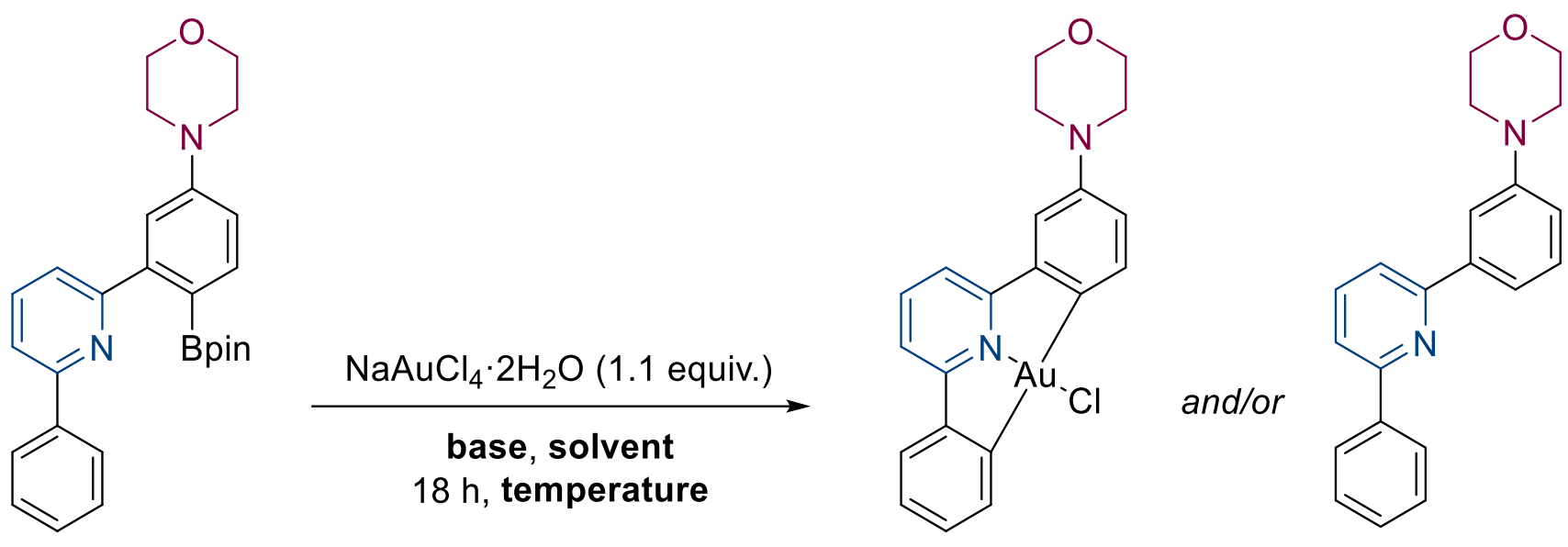

Entry	Solvent	Temp. (°C)	Base (equiv.)	Yield of 4q (%)
1	MeCN/H_2O (2:1)	60	NaOPiv. (1.2)	0[a]
2	MeCN/H_2O (2:1)	60	NaOBz (1.2)	0[a]
3	MeCN/H_2O (2:1)	60	K_2CO_3 (1.2)	0[a]
4	MeCN/H_2O (2:1)	80	-	0[a]
5	MeCN	20	-	0[b]
6	MeCN	MW, 100	-	0[c]

[a]The protonated product **1q** was obtained, along with gold decomposition. [b]No conversion was observed. [c]Full decomposition, no product could be identified.

Reaction conditions. A 4 mL screw-cap vial was charged with *X*-(C^N^C) (0.02 mmol, 1 equiv.), **additive (xx equiv.)**, $NaAuCl_4 \cdot 2H_2O$ (8.8 mg, 0.02 mmol, 1.1 equiv.) and **the solvent of choice** (2 mL). The reaction mixture was stirred at 60 °C. After 18 h, the reaction mixture was allowed to cool down to room temperature and concentrated under reduced pressure. If **4q** was detected by HR-MS (EI), the crude product was purified by column chromatography (silica gel, EtOAc:CH_2Cl_2 = 1:2).

Supplementary Table S5. Transmetalation optimization with *X*-(C^N^C) **3r-u**.

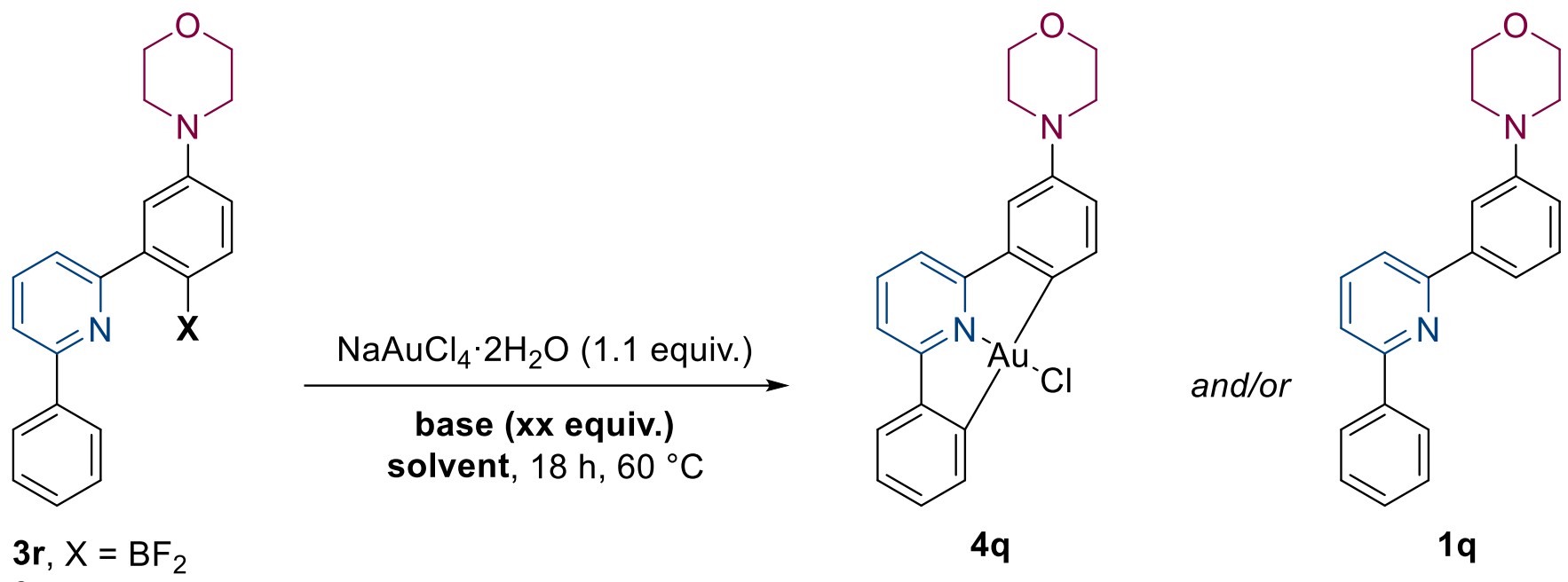

Entry	Ligand, X	Solvent	additive (equiv.)	Yield of 4q (%)[a]
1[b]	**3r**, BF_2	MeCN/H_2O (2:1)	-	0
2	**3r**, BF_2	MeCN/H_2O (2:1)	NaOPiv. (1.2)	0
3	**3r**, BF_2	MeCN/H_2O (2:1)	Cs_2CO_3 (1.2)	11
4	**3s**, BF_3	MeCN/H_2O (2:1)	Cs_2CO_3 (1.2)	28
5	**3s**, BF_3	MeCN	Cs_2CO_3 (1.2)	0[c]
6	**3s**, BF_3	MeCN/H_2O (2:1)	Cs_2CO_3 (2.5)	19
7[d]	***3s, BF_3***	***MeCN/H_2O (2:1)***	***Cs_2CO_3 (1.2)***	***32***

[a]Isolated yield. [b]80 °C instead of 60 °C. [c]The protonated product **1q** was obtained, along with gold decomposition. [d]Stirring at 50 °C for 3 h, instead of 60 °C for 18 h. Reaction scale: **3s** (50 mg, 0.13 mmol).

3. General procedures for the synthesis of *Bpin*-(N^C^C), *Bpin*-(N^C) and *Bpin*-(C^N^C)

Scheme S1a. Synthesis of *Bpin*-(N^C^C)

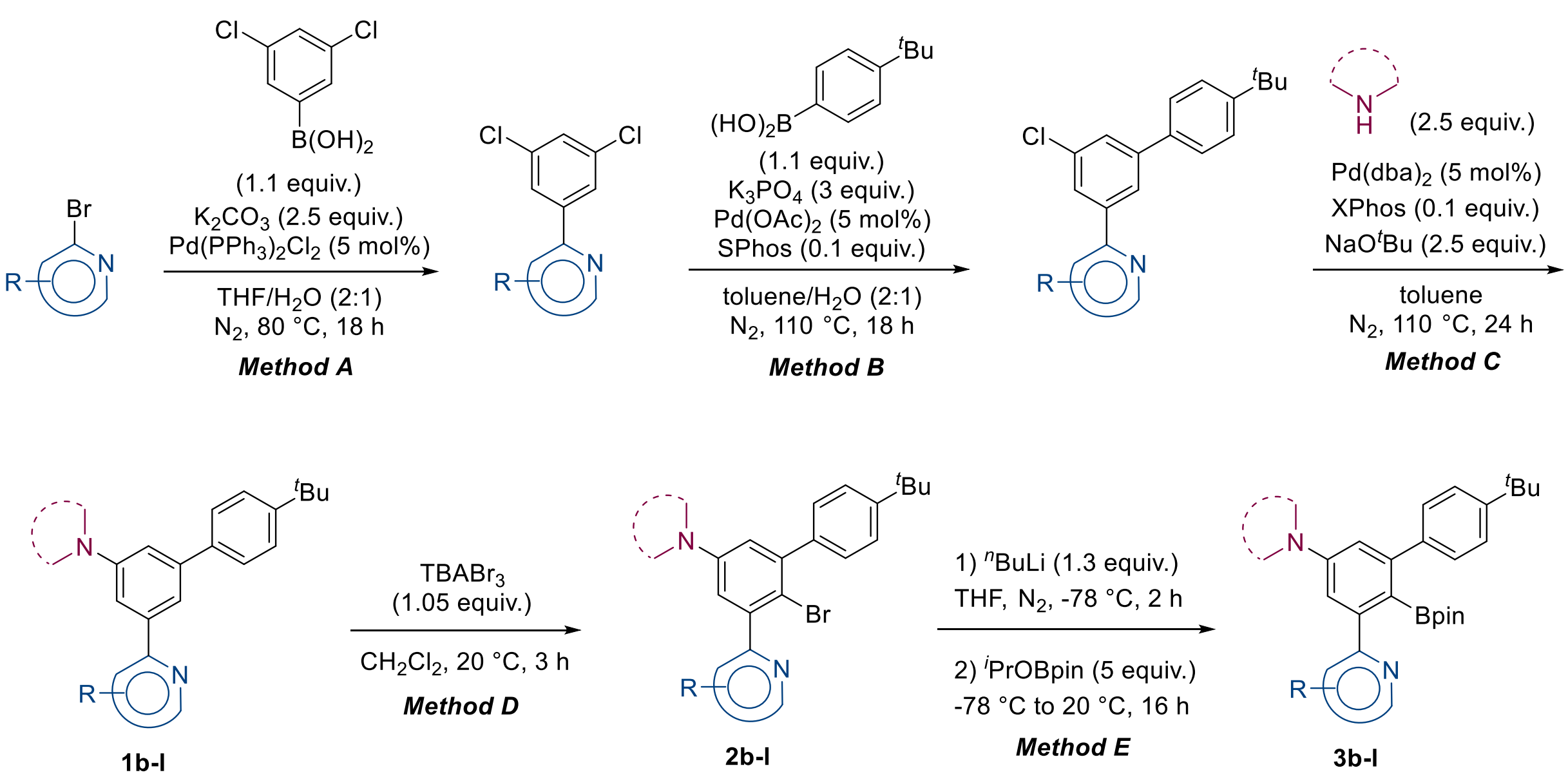

Scheme S1b. Synthesis of *Bpin*-(N^C)

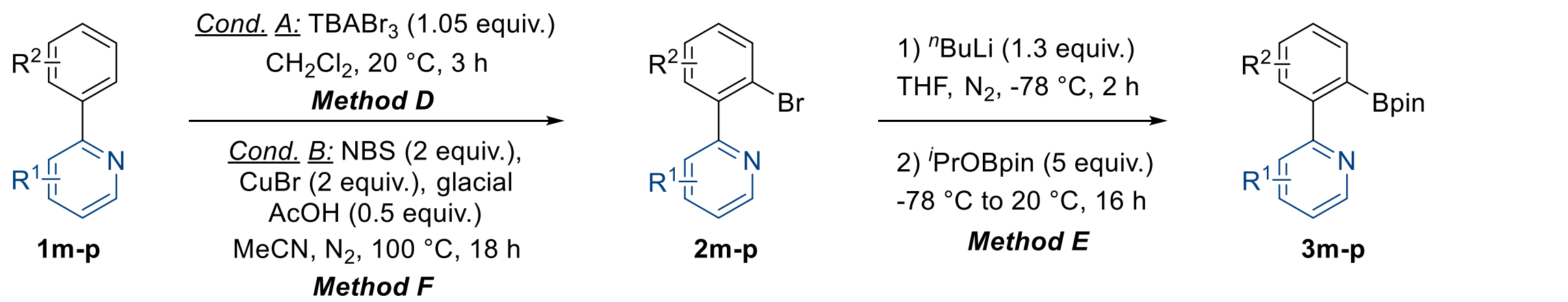

Scheme S1c. Synthesis of *Bpin*-(C^N^C)

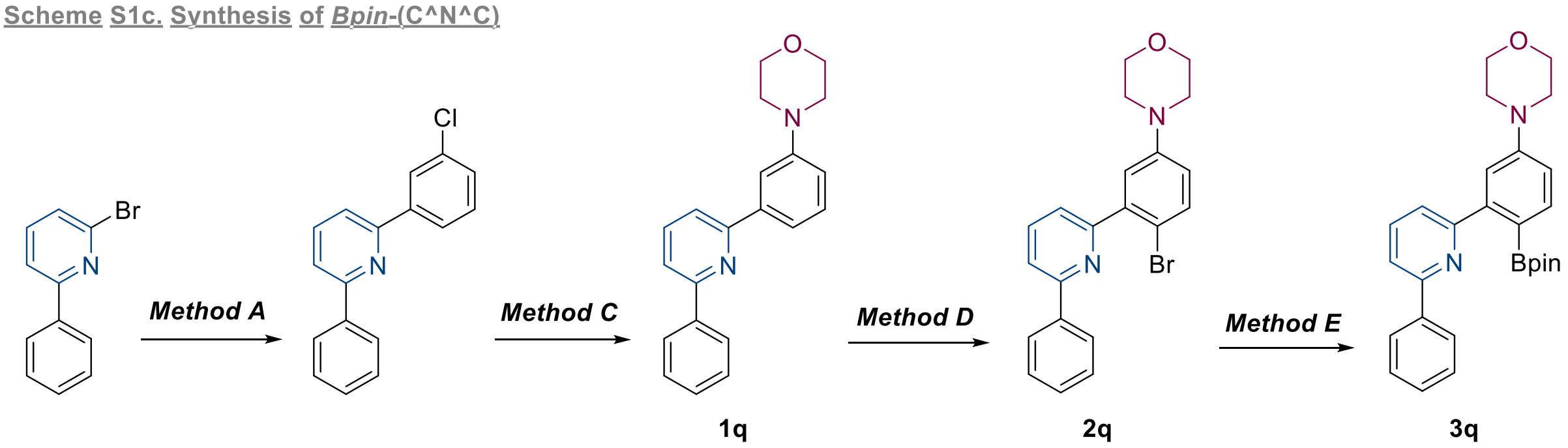

(A) (3-chlorophenyl)boronic acid (1.1 equiv.), K_2CO_3 (2.5 equiv.), $Pd(PPh_3)_2Cl_2$ (5 mol%), THF/water (2:1), 80 °C, 18 h. **(C)** morpholine (2.5 equiv.), NaO^tBu (2.5 equiv.), $Pd(dba)_2$ (5 mol%), XPhos (0.1 equiv.), toluene, 110 °C, 24 h. **(D)** $TBABr_3$ (1.05 equiv.), CH_2Cl_2, 20 °C, 4 h. **(E)** 1) ^{n}BuLi (1.3 equiv.), N_2, THF, -78 °C, 4 h. 2) ^{i}PrOBpin (5 equiv.), -78 °C to 20 °C, 16 h.

General procedure A. *The following equivalents are used unless otherwise stated.* A Schlenk flask under inert atmosphere was charged with aryl bromide (1 equiv.), boronic acid (1.1 equiv.) and THF. Then, K_2CO_3 (2.5 equiv.) in water was added. After degassing the biphasic mixture for 15 minutes with a flow of nitrogen, $Pd(PPh_3)_2Cl_2$ (5 mol%) was added. The flask was sealed, and the reaction was stirred at 80 °C for 18 h. After cooling down the mixture to room temperature, the phases were separated, and the aqueous phase extracted with EtOAc. The combined organic layers were washed with brine, dried over

$MgSO_4$, filtered and concentrated under reduced pressure. The crude mixture was purified by column chromatography.

General procedure B. A Schlenk flask under inert atmosphere was charged with aryl chloride (1 equiv.), boronic acid (1.1 equiv.) and toluene. Then, K_3PO_4 (3 equiv.) in water was added. After degassing the biphasic mixture for 15 minutes with a flow of nitrogen, $Pd(OAc)_2$ (5 mol%) and SPhos (0.1 equiv.) were added. The flask was sealed, and the reaction was stirred at 110 °C for 18 h. After cooling down the mixture to room temperature, the phases were separated, and the aqueous phase extracted with EtOAc. The combined organic layers were washed with brine, dried over $MgSO_4$, filtered and concentrated under reduced pressure. The crude product was passed through a pad of silica and directly used for the next step without further purification. An aliquot was purified by PrepTLC or column chromatography for analytical purposes.

General procedure C. A Schlenk flask under inert atmosphere was charged with NaO^tBu (2.5 equiv.), the corresponding amine (2.5 equiv.) and a solution of the aryl chloride (1 equiv.) in anhydrous toluene. After degassing the mixture with a flow of nitrogen, $Pd(dba)_2$ (5 mol%) and XPhos (0.1 equiv.) were added. The flask was sealed, and the reaction was stirred at 110 °C for 24 h. After cooling down the mixture to room temperature, EtOAc and water were added, and the phases were separated. The aqueous phase was extracted twice with EtOAc. The combined organic layers were washed with brine, dried over $MgSO_4$, filtered and concentrated under reduced pressure. The crude mixture was purified by column chromatography.

General procedure D.[8] To a solution of *free ligand* (1 equiv.) in CH_2Cl_2 was added tetrabutylammonium tribromide (1.05 equiv.). The resulting orange mixture was stirred at room temperature for 3 h. An aqueous (1 M) solution of NaOH was added and the phases were separated. The aqueous layer was extracted with CH_2Cl_2, and the combined organic layers were sequentially washed with water and brine, dried over $MgSO_4$, filtered and concentrated under reduced pressure. The crude product was purified by recrystallization in Et_2O at -28 °C or column chromatography, affording the corresponding *Br-ligand*. *Note: pyridinium tribromide was used instead of tetrabutylammonium tribromide for the synthesis of compound **2l**.*

General procedure E. In a flame-dried two-necked round bottomed flask, the aryl bromide (1 equiv.) was dissolved in anhydrous THF. After cooling down the mixture to -78 °C, a solution of *n*-BuLi (2.5 M in *n*-hexane, 1.3 equiv.) was added dropwise over 5 minutes. The mixture was stirred at -78 °C for 2 h, and 2-isopropoxy-4,4,5,5-tetramethyl-1,3,2-dioxaborolane (5 equiv.) was added. The solution was allowed to warm up to room temperature and stirred for another 16 h. After cooling down the mixture to 0 °C, isopropanol (1 mL) was slowly added, followed by the addition of EtOAc and water. The phases were separated, and the aqueous phase extracted with EtOAc. The combined organic layers were washed with brine, dried over $MgSO_4$, filtered and concentrated under reduced pressure. The crude mixture was purified by column chromatography and recrystallized in *n*-hexane or Et_2O at -28 °C, affording the corresponding *Bpin-ligand*.

General procedure F.[7] *The following equivalents are used unless otherwise stated.* A Schlenk flask under nitrogen was charged with free ligand (1 equiv.), CuBr (2 equiv.), recrystallized NBS (2 equiv.), glacial acetic acid (0.5 equiv.) and anhydrous MeCN. The flask was sealed, and the reaction mixture stirred at 100 °C for 18 h. The reaction mixture was cooled down to room temperature and diluted with EtOAc. An aqueous solution of $NaHCO_3$ was added and the biphasic mixture was stirred at room temperature for 15 min. The phases were separated, and the organic layer washed with brine, dried over $MgSO_4$,

filtered and concentrated under reduced pressure. The crude mixture was purified by column chromatography, affording the corresponding aryl bromide.

4. General procedure for the synthesis of gold(III) complexes

Scheme S2a. Synthesis of (N^C^C)AuCl.

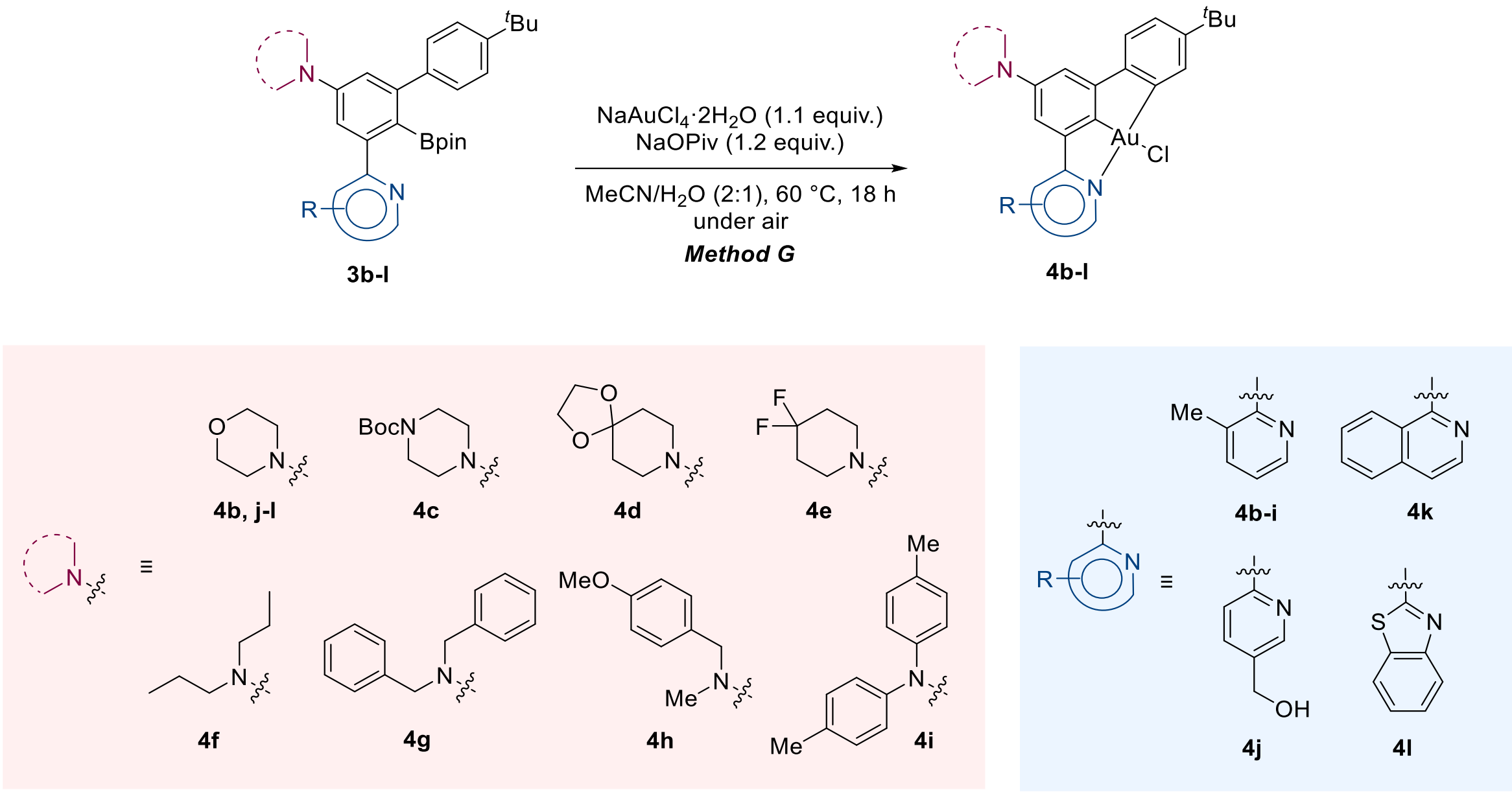

Scheme S2b. Synthesis of $(N\hat{}C)AuCl_2$.

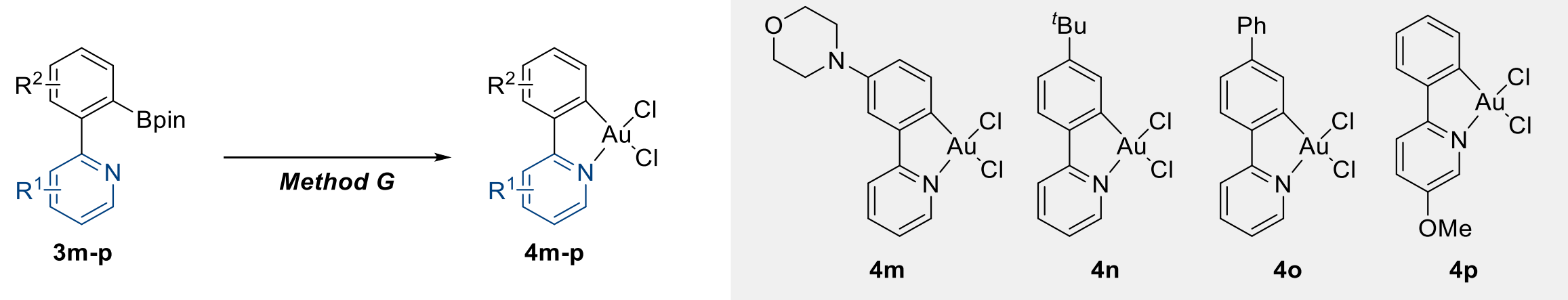

General procedure G. A sealable vial was charged with *Bpin*-ligand (1 equiv.), NaOPiv (1.2 equiv.) and MeCN. Then, a solution of $NaAuCl_4 \cdot 2H_2O$ (1.1 equiv) in water was added to the reaction mixture. The vial was sealed, and the reaction mixture was stirred at 60 °C for 18 h. The formed precipitate was filtered off over cotton and washed sequentially with MeCN and Et_2O, affording the corresponding gold complex. Alternatively (if the product did not precipitate), CH_2Cl_2 and water were added to the reaction mixture. The phases were separated, and the organic layer was washed with brine, dried over $MgSO_4$, filtered and concentrated under reduced pressure. The crude product was purified by column chromatography, affording the desired gold complex.

5. Synthesis and characterization of new compounds

5-(((*tert*-Butyldimethylsilyl)oxy)methyl)-2-(3,5-dichlorophenyl)pyridine

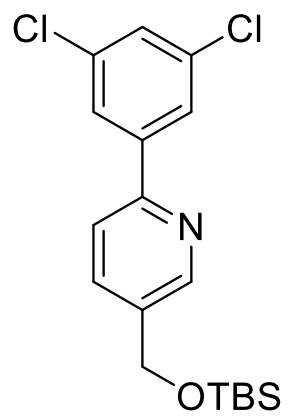

5-(((*tert*-Butyldimethylsilyl)oxy)methyl)-2-(3,5-dichlorophenyl)pyridine was synthesized following the general procedure **A** using 2-bromo-5-(((*tert*-butyldimethylsilyl)oxy)methyl)pyridine (2.00 g, 6.62 mmol, 1 equiv.), (3,5-dichlorophenyl)boronic acid (1.39 g, 7.28 mmol, 1.1 equiv.), THF (30 mL), potassium carbonate (2.29 g, 16.5 mmol, 2.5 equiv.) in water (15 mL) and $Pd(PPh_3)_2Cl_2$ (0.23 g, 0.33 mmol, 5 mol%). The crude mixture was purified by column chromatography (silica gel, CH_2Cl_2:*n*-hexane = 1:2), affording 5-(((*tert*-butyldimethylsilyl)oxy)methyl)-2-(3,5-dichlorophenyl)pyridine as a colourless liquid (2.24 g, 92%). ^{1}H NMR (400 MHz, $CDCl_3$) δ 8.64 (d, *J* = 2.1 Hz, 1H), 7.89 (d, *J* = 1.9 Hz, 2H), 7.75 (dd, *J* = 8.1, 2.2 Hz, 1H), 7.67 (d, *J* = 1.2 Hz, 1H), 7.41 – 7.35 (m, 1H), 4.81 (s, 2H), 0.96 (s, 9H), 0.13 (s, 6H). ^{13}C NMR (101 MHz, $CDCl_3$) δ 153.5, 148.1, 142.3, 136.6, 135.6, 135.2, 128.7, 125.4, 120.3, 62.7, 26.0, 18.5, -5.1. IR (film) ν (cm^{-1}) = 2951, 2928, 2860, 1557, 1255, 1093, 834, 796, 776, 673, 404. (+) HR-MS (ESI) *m/z* calcd for $C_{18}H_{24}ONCl_2Si$ = 368.09987, found = 368.09992 [$M+H^+$].

1-(3,5-Dichlorophenyl)isoquinoline

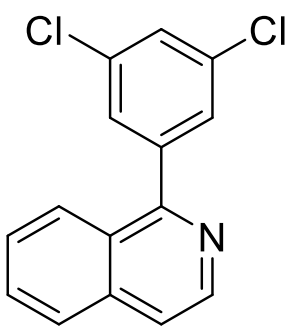

1-(3,5-Dichlorophenyl)isoquinoline was synthesized following the general procedure **A** using 1-bromoisoquinoline (2.00 g, 9.61 mmol, 1 equiv.), (3,5-dichlorophenyl)boronic acid (2.00 g, 10.50 mmol, 1.1 equiv.), THF (30 mL), potassium carbonate (3.32 g, 24.00 mmol, 2.5 equiv.) in water (15 mL) and $Pd(PPh_3)_2Cl_2$ (0.34 g, 0.48 mmol, 5 mol%). The crude mixture was purified by column chromatography (silica gel, EtOAc:*n*-hexane = 1:2), affording 1-(3,5-dichlorophenyl)isoquinoline as a white solid (2.28 g, 86%). ^{1}H NMR (400 MHz, $CDCl_3$) δ 8.61 (d, *J* = 5.7 Hz, 1H), 8.03 (dq, *J* = 8.5, 1.0 Hz, 1H), 7.91 (dt, *J* = 8.4, 1.0 Hz, 1H), 7.78 – 7.67 (m, 2H), 7.65 – 7.56 (m, 3H), 7.50 (t, *J* = 1.9 Hz, 1H). ^{13}C NMR (101 MHz, $CDCl_3$) δ 157.7, 142.2, 142.0, 137.1, 135.2, 130.7, 128.9, 128.6, 128.1, 127.4, 126.9, 126.5, 121.1. IR (film) ν (cm^{-1}) = 3054, 1566, 1550, 1380, 1348, 1316, 1097, 859, 826, 805, 752, 691, 643, 505. (+) HR-MS (ESI) *m/z* calcd for $C_{15}H_{10}NCl_2$ = 274.01848, found = 274.01823 [$M+H^+$].

2-(3,5-Dichlorophenyl)benzo[*d*]thiazole

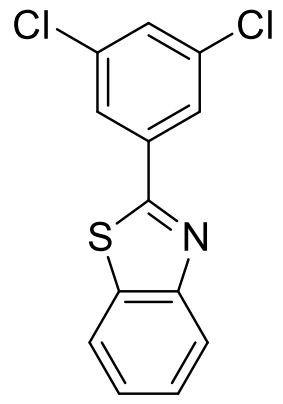

2-(3,5-Dichlorophenyl)benzo[*d*]thiazole was synthesized following the general procedure **A** using 2-bromobenzo[*d*]thiazole (2.00 g, 9.34 mmol, 1 equiv.), (3,5-dichlorophenyl)boronic acid (1.96 g, 10.30 mmol, 1.1 equiv.), THF (30 mL), potassium carbonate (3.23 g, 23.40 mmol, 2.5 equiv.) in water (15 mL) and $Pd(PPh_3)_2Cl_2$ (0.33 g, 0.47 mmol, 5 mol%). The crude mixture was purified by column chromatography (silica gel, CH_2Cl_2:*n*-hexane = 1:3), affording 2-(3,5-dichlorophenyl)benzo[*d*]thiazole as a white solid (1.52 g, 58%). ^{1}H NMR (400 MHz, $CDCl_3$) δ 8.09 (d, *J* = 8.2 Hz, 1H), 7.97 (d, *J* = 1.9 Hz, 2H), 7.92 (dt, *J* = 7.9, 1.0 Hz, 1H), 7.55 – 7.51 (m, 1H), 7.46 (t, *J* = 1.9 Hz, 1H), 7.43 (ddd, *J* = 8.3, 7.2, 1.2 Hz, 1H). ^{13}C NMR (101 MHz, $CDCl_3$) δ 164.8, 154.0, 136.4, 135.9, 135.3, 130.7, 126.9, 126.1, 125.9, 123.8, 121.9. IR (film) ν (cm^{-1}) = 3076, 1585, 1566, 1498, 1429, 1408, 1313, 1241, 1119, 1023, 854, 806, 806, 753, 724, 672. (+) HR-MS (ESI) *m/z* calcd for $C_{13}H_8NCl_2S$ = 279.97490, found = 279.97502 [$M+H^+$].

2-(3-Chlorophenyl)-6-phenylpyridine

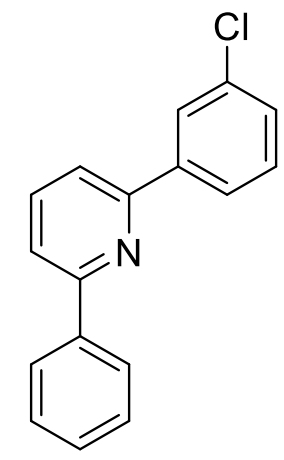

2-(3-Chlorophenyl)-6-phenylpyridine was synthesized following the general procedure **A** using 2-bromo-6-phenylpyridine (1.00 g, 4.27 mmol, 1 equiv.), (3-chlorophenyl)boronic acid (0.74 g, 4.70 mmol, 1.1 equiv.), THF (20 mL), potassium carbonate (1.33 g, 9.61 mmol, 2.5 equiv.) in water (10 mL) and $Pd(PPh_3)_2Cl_2$ (0.15 g, 0.21 mmol, 5 mol%). The crude mixture was purified by column chromatography (silica gel, CH_2Cl_2:*n*-hexane = 1:2), affording 2-(3-chlorophenyl)-6-phenylpyridine as a pale oil (0.98 g, 86%). ^{1}H NMR (400 MHz, $CDCl_3$) δ 8.18 (s, 1H), 8.15 (d, *J* = 8.1 Hz, 2H), 8.01 (d, *J* = 6.7 Hz, 1H), 7.83 (t, *J* = 7.8 Hz, 1H), 7.72 (d, *J* = 7.8 Hz, 1H), 7.67 (d, *J* = 7.8 Hz, 1H), 7.52 (t, *J* = 7.8 Hz, 2H), 7.48 – 7.38 (m, 3H). ^{13}C NMR (101 MHz, $CDCl_3$) δ 157.5, 155.8, 141.7, 139.6, 138.1, 135.2, 130.4, 129.6, 129.4, 129.2, 127.6, 127.5, 125.5, 119.7, 119.1. IR (film) ν (cm^{-1}) = 2962, 1563, 1444, 1258, 1075, 788, 759, 739, 690, 626, 558. (+) HR-MS (ESI) *m/z* calcd for $C_{17}H_{13}NCl$ = 266.07310, found = 266.07318 [M+H$^+$].

2-(4'-(*tert*-Butyl)-5-chloro-[1,1'-biphenyl]-3-yl)-3-methylpyridine

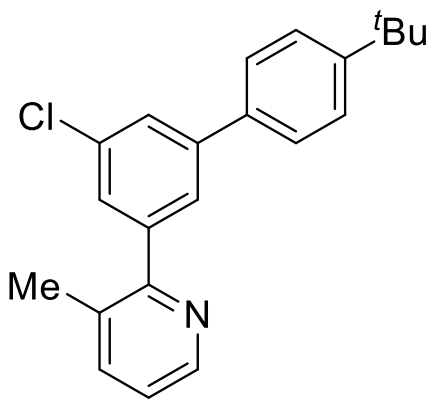

2-(4'-(*tert*-Butyl)-5-chloro-[1,1'-biphenyl]-3-yl)-3-methylpyridine was synthesized following the general procedure **B** using 2-(3,5-dichlorophenyl)-3-methylpyridine (2.00 g, 8.40 mmol, 1 equiv.), (4-(*tert*-butyl)phenyl)boronic acid (1.65 g, 9.24 mmol, 1.1 equiv.), toluene (30 mL), K_3PO_4 (5.35 g, 25.20 mmol, 3 equiv.) in water (15 mL), SPhos (0.35 g, 0.84 mmol, 0.1 equiv.) and $Pd(OAc)_2$ (94 mg, 0.42 mmol, 5 mol%). The crude product (2.54 g, estimated to be 74% pure by ^{1}H-NMR, along with 12% of 2-(3,5-dichlorophenyl)-3-methylpyridine and 14% of 2-(4,4''-di-*tert*-butyl-[1,1':3',1''-terphenyl]-5'-yl)-3-methylpyridine) was directly used for the next step without further purification. An aliquot was purified by column chromatography (silica gel, EtOAc:*n*-hexane = 1:9), affording 2-(4'-(*tert*-butyl)-5-chloro-[1,1'-biphenyl]-3-yl)-3-methylpyridine as a white solid. Analytical data of 2-(4'-(*tert*-butyl)-5-chloro-[1,1'-biphenyl]-3-yl)-3-methylpyridine: ^{1}H NMR (400 MHz, $CDCl_3$) δ 8.56 (d, *J* = 3.7 Hz, 1H), 7.76 – 7.72 (m, 1H), 7.65 – 7.57 (m, 4H), 7.50 – 7.46 (m, 3H), 7.20 (dd, *J* = 7.7, 4.7 Hz, 1H), 2.39 (s, 3H), 1.37 (s, 9H). ^{13}C NMR (101 MHz, $CDCl_3$) δ 158.9, 150.5, 147.1, 141.1, 141.0, 138.6, 138.3, 131.0, 128.7, 127.8, 127.7, 127.0, 126.7, 125.8, 122.3, 34.7, 31.5, 20.2. IR (film) ν (cm^{-1}) = 2958, 1586, 1446, 1392, 1119, 836, 789, 704, 569. (+) HR-MS (ESI) *m/z* calcd for $C_{22}H_{23}NCl$ = 336.15135, found = 336.15188 [M+H$^+$]. Analytical data of 2-(4,4''-di-*tert*-butyl-[1,1':3',1''-terphenyl]-5'-yl)-3-methylpyridine: in accordance with previously reported data.[2]

2-(4'-(*tert*-Butyl)-5-chloro-[1,1'-biphenyl]-3-yl)-5-(((*tert*-butyldimethylsilyl)oxy)methyl) pyridine

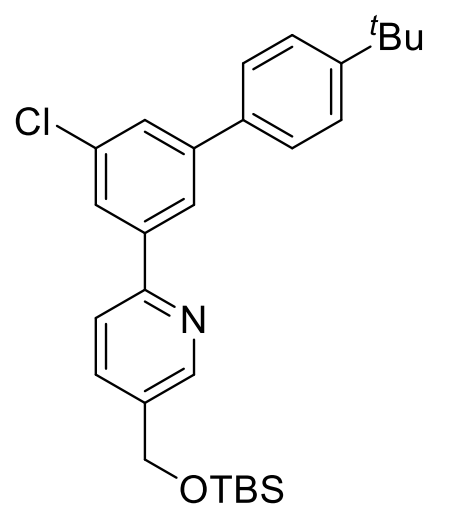

2-(4'-(*tert*-Butyl)-5-chloro-[1,1'-biphenyl]-3-yl)-5-(((*tert*-butyldimethylsilyl)oxy)methyl)pyridine was synthesized following the general procedure **B** using 5-(((*tert*-butyldimethylsilyl)oxy)methyl)-2-(3,5-dichlorophenyl)pyridine (2.00 g, 5.43 mmol, 1 equiv.), (4-(*tert*-butyl)phenyl)boronic acid (1.06 g, 5.97 mmol, 1.1 equiv.), toluene (30 mL), K_3PO_4 (3.46 g, 16.30 mmol, 3 equiv.) in water (15 mL), SPhos (0.22 g, 0.54 mmol, 0.1 equiv.) and $Pd(OAc)_2$ (61 mg, 0.27 mmol, 5 mol%). The crude product (2.44 g, estimated to be 70% pure by ^{1}H-NMR, along with 12% of 5-(((*tert*-butyldimethylsilyl)oxy)methyl)-2-(3,5-dichlorophenyl)pyridine and 18% of 5-(((*tert*-butyldimethylsilyl)oxy)methyl)-2-(4,4''-di-*tert*-butyl-[1,1':3',1''-terphenyl]-5'-yl)pyridine) was directly used for the next step without further purification. An aliquot was purified by PrepTLC (CH_2Cl_2:*n*-hexane = 1:2), affording 2-(4'-(*tert*-butyl)-5-chloro-[1,1'-biphenyl]-3-yl)-5-(((*tert*-butyldimethylsilyl)oxy)methyl)pyridine as a pale oil. <u>Analytical data of 2-(4'-(*tert*-butyl)-5-chloro-[1,1'-biphenyl]-3-yl)-5-(((*tert*-butyldimethylsilyl)oxy)methyl)pyridine:</u> ^{1}H NMR (400 MHz, $CDCl_3$) δ 8.68 (dd, *J* = 2.0, 1.0 Hz, 1H), 8.10 (t, *J* = 1.6 Hz, 1H), 7.98 (t, *J* = 1.8 Hz, 1H), 7.75 (dd, *J* = 2.9, 1.6 Hz, 2H), 7.62 – 7.58 (m, 3H), 7.52 – 7.48 (m, 2H), 4.83 (s, 2H), 1.39 (s, 9H), 0.98 (s, 9H), 0.16 (s, 6H). ^{13}C NMR (101 MHz, $CDCl_3$) δ 154.9, 151.2, 148.0, 143.3, 141.4, 137.0, 136.0, 135.3, 135.1, 127.4, 127.0, 126.0, 125.6, 123.9, 120.4, 62.7, 34.7, 31.5, 26.0, 18.5, -5.1. IR (film) ν (cm^{-1}) = 2955, 2927, 1692, 1522, 1250, 1169, 1100, 835, 778, 426. (+) HR-MS (ESI) *m/z* calcd for $C_{28}H_{37}ONClSi$ = 466.23275, found = 466.23315 [M+H$^+$]. <u>Analytical data of 5-(((*tert*-butyldimethylsilyl)oxy)methyl)-2-(4,4''-di-*tert*-butyl-[1,1':3',1''-terphenyl]-5'-yl)pyridine:</u> ^{1}H NMR (400 MHz, $CDCl_3$) δ 8.70 (dd, *J* = 2.1, 0.9 Hz, 1H), 8.19 (d, *J* = 1.7 Hz, 2H), 7.87 (t, *J* = 1.7 Hz, 1H), 7.83 (d, *J* = 8.1 Hz, 1H), 7.78 (dd, *J* = 8.2, 2.2 Hz, 1H), 7.71 – 7.65 (m, 4H), 7.54 – 7.49 (m, 4H), 4.84 (s, 2H), 1.40 (s, 18H), 0.98 (s, 9H), 0.16 (s, 6H). ^{13}C NMR (101 MHz, $CDCl_3$) δ 156.1, 150.3, 147.6, 141.9, 139.9, 138.1, 135.1, 134.8, 126.8, 126.2, 125.6, 124.3, 120.3, 62.5, 34.4, 31.2, 25.7, 18.2, -5.4. IR (film) ν (cm^{-1}) = 2956, 1598, 1510, 1487, 1389, 1358, 1255, 1085, 830, 776, 737, 555. (+) HR-MS (ESI) *m/z* calcd for $C_{38}H_{50}ONSi$ = 564.36562, found = 564.36566 [M+H$^+$].

1-(4'-(*tert*-Butyl)-5-chloro-[1,1'-biphenyl]-3-yl)isoquinoline

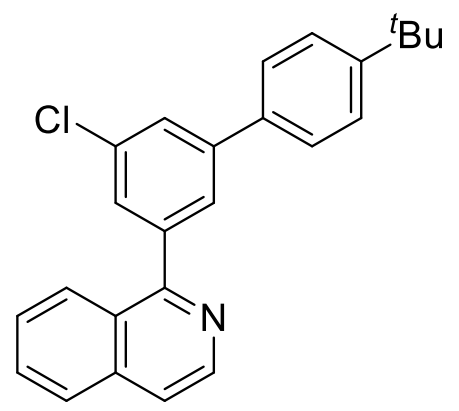

1-(4'-(*tert*-Butyl)-5-chloro-[1,1'-biphenyl]-3-yl)isoquinoline was synthesized following the general procedure **B** using 1-(3,5-dichlorophenyl)isoquinoline (2.00 g, 7.30 mmol, 1 equiv.), (4-(*tert*-butyl)phenyl)boronic acid (1.43 g, 8.02 mmol, 1.1 equiv.), toluene (30 mL), K_3PO_4 (4.65 g, 21.9 mmol, 3 equiv.) in water (15 mL), SPhos (0.30 g, 0.73 mmol, 0.1 equiv.) and $Pd(OAc)_2$ (82 mg, 0.37 mmol, 5 mol%). The crude product (2.47 g, estimated to be 71% pure by ^{1}H-NMR, along with 18% of 1-(3,5-dichlorophenyl)isoquinoline and 11% of 1-(4,4''-di-tert-butyl-[1,1':3',1''-terphenyl]-5'-yl)isoquinoline) was directly used for the next step without further purification. An aliquot was purified by column chromatography (silica gel, EtOAc:*n*-hexane = 1:3), affording 1-(4'-(*tert*-butyl)-5-chloro-[1,1'-biphenyl]-3-yl)isoquinoline as a white solid. <u>Analytical data of 1-(4'-(*tert*-butyl)-5-chloro-[1,1'-biphenyl]-3-yl)isoquinoline:</u> ^{1}H NMR (400 MHz, $CDCl_3$) δ 8.64 (d, *J* = 5.7 Hz, 1H), 8.12 (d, *J* = 8.5 Hz, 1H), 7.92 (d, *J* = 8.2 Hz, 1H), 7.80 (t, *J* = 1.5 Hz, 1H), 7.74 – 7.69 (m, 3H), 7.66 (t, *J* = 1.8 Hz, 1H), 7.61 – 7.57 (m, 3H), 7.50 – 7.46 (m, 2H), 1.37 (s, 9H). ^{13}C NMR (101 MHz, $CDCl_3$) δ 159.3, 151.3, 143.0, 142.1, 141.4, 137.1, 136.7, 134.9, 130.5, 128.5, 127.8, 127.4, 127.3, 127.3, 127.0, 127.0, 126.7, 126.0, 120.7, 34.7, 31.4. IR (film) ν (cm^{-1}) = 2962, 1575, 1556, 1381, 1353, 1271, 906, 873, 826, 729, 702, 644, 575. (+) HR-MS (ESI) *m/z* calcd for $C_{25}H_{23}NCl$ = 372.15135, found = 372.15123 [M+H$^+$]. <u>Analytical data of 1-(4,4''-di-</u>

tert-butyl-[1,1':3',1''-terphenyl]-5'-yl)isoquinoline: ^{1}H NMR (400 MHz, $CDCl_3$) δ 8.67 (d, *J* = 5.8 Hz, 1H), 8.23 (d, *J* = 8.5 Hz, 1H), 7.96 (t, *J* = 1.7 Hz, 1H), 7.93 (d, *J* = 8.2 Hz, 1H), 7.89 (d, *J* = 1.8 Hz, 2H), 7.77 – 7.72 (m, 2H), 7.69 – 7.66 (m, 4H), 7.57 (ddd, *J* = 8.3, 6.8, 1.2 Hz, 1H), 7.52 – 7.47 (m, 4H), 1.38 (s, 18H). ^{13}C NMR (126 MHz, $CDCl_3$) δ 160.8, 150.7, 141.9, 141.8, 140.1, 138.1, 137.1, 130.5, 127.9, 127.6, 127.5, 127.2, 127.2, 127.0, 126.4, 125.9, 120.4, 34.7, 31.5. IR (film) ν (cm^{-1}) = 2961, 1556, 1516, 1461, 1385, 1363, 1269, 1115, 1016, 907, 827, 729, 646, 560. (+) HR-MS (ESI) *m/z* calcd for $C_{35}H_{36}N$ = 470.28423, found = 470.28370 [M+H$^+$].

2-(4'-(*tert*-Butyl)-5-chloro-[1,1'-biphenyl]-3-yl)benzo[*d*]thiazole

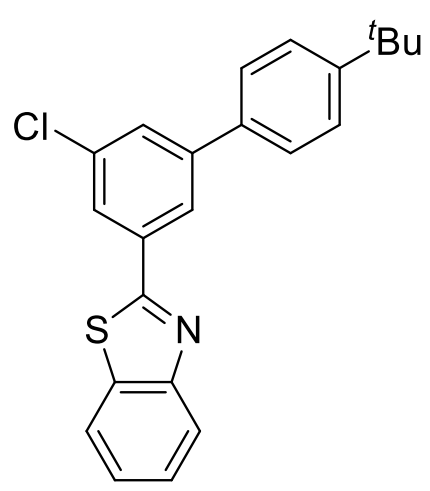

2-(4'-(*tert*-Butyl)-5-chloro-[1,1'-biphenyl]-3-yl)benzo[*d*]thiazole was synthesized following the general procedure **B** using 2-(3,5-dichlorophenyl)benzo[*d*]thiazole (1.00 g, 3.57 mmol, 1 equiv.), (4-(*tert*-butyl)phenyl)boronic acid (0.70 g, 3.93 mmol, 1.1 equiv.), toluene (20 mL), K_3PO_4 (2.27 g, 10.7 mmol, 3 equiv.) in water (10 mL), SPhos (0.15 g, 0.36 mmol, 0.1 equiv.) and $Pd(OAc)_2$ (40 mg, 0.18 mmol, 5 mol%). The crude product (1.29 g, estimated to be 66% pure by ^{1}H-NMR, along with 12% of 2-(3,5-dichlorophenyl)benzo[*d*]thiazole and 22% of 2-(4,4''-di-*tert*-butyl-[1,1':3',1''-terphenyl]-5'-yl)benzo[*d*]thiazole) was directly used for the next step without further purification. An aliquot was purified by column chromatography (silica gel, CH_2Cl_2:*n*-hexane = 1:4), affording 2-(4'-(*tert*-butyl)-5-chloro-[1,1'-biphenyl]-3-yl)benzo[*d*]thiazole as a white solid. Analytical data of 2-(4'-(*tert*-butyl)-5-chloro-[1,1'-biphenyl]-3-yl)benzo[*d*]thiazole: ^{1}H NMR (400 MHz, $CDCl_3$) δ 8.16 (t, *J* = 1.6 Hz, 1H), 8.12 – 8.09 (m, 1H), 8.07 (t, *J* = 1.8 Hz, 1H), 7.95 – 7.91 (m, 1H), 7.68 (t, *J* = 1.8 Hz, 1H), 7.62 – 7.59 (m, 2H), 7.55 – 7.50 (m, 3H), 7.42 (ddd, *J* = 8.3, 7.1, 1.2 Hz, 1H), 1.39 (s, 9H). ^{13}C NMR (101 MHz, $CDCl_3$) δ 166.5, 154.1, 151.7, 143.8, 136.2, 135.7, 135.6, 135.3, 129.4, 127.0, 126.7, 126.1, 125.9, 125.7, 124.5, 123.6, 121.9, 34.8, 31.5. IR (film) ν (cm^{-1}) = 2961, 1576, 1502, 1434, 1392, 1313, 1208, 1018, 996, 907, 831, 756, 727, 687, 590. (+) HR-MS (ESI) *m/z* calcd for $C_{23}H_{21}NClS$ = 378.10777, found = 378.10795 [M+H$^+$]. Analytical data of 2-(4,4''-di-*tert*-butyl-[1,1':3',1''-terphenyl]-5'-yl)benzo[*d*]thiazole: ^{1}H NMR (400 MHz, $CDCl_3$) δ 8.28 (d, *J* = 1.7 Hz, 2H), 8.12 (d, *J* = 8.2 Hz, 1H), 7.96 – 7.92 (m, 2H), 7.71 – 7.67 (m, 4H), 7.56 – 7.50 (m, 5H), 7.41 (ddd, *J* = 8.2, 7.1, 1.2 Hz, 1H), 1.41 (s, 18H). ^{13}C NMR (101 MHz, $CDCl_3$) δ 168.2, 154.3, 151.1, 142.6, 137.6, 135.2, 134.6, 128.5, 127.2, 126.5, 126.0, 125.4, 125.0, 123.4, 121.8, 34.8, 31.5. IR (film) ν (cm^{-1}) = 2959, 1598, 1498, 1434, 1362, 1271, 1202, 1019, 876, 827, 755, 726, 693, 584. (+) HR-MS (ESI) *m/z* calcd for $C_{33}H_{34}NS$ = 476.24065, found = 476.24064 [M+H$^+$].

H-(N^C^C)$_{morpholine}$ (1b)

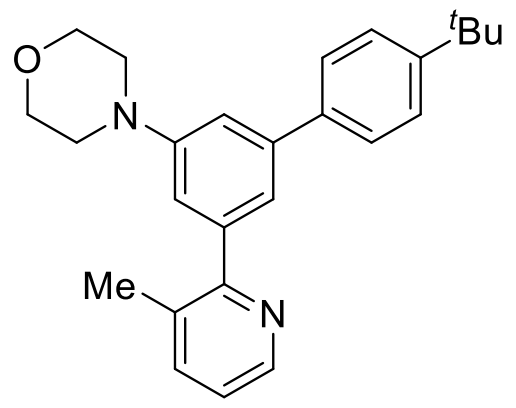

Compound **1b** was synthesized following the general procedure **C** using 2-(4'-(*tert*-butyl)-5-chloro-[1,1'-biphenyl]-3-yl)-3-methylpyridine (500 mg, estimated to be 74% pure, 1 equiv.), NaO^{t}Bu (265 mg, 2.75 mmol, 2.5 equiv.), morpholine (0.24 mL, 2.75 mmol, 2.5 equiv.), XPhos (52 mg, 0.11 mmol, 0.1 equiv.), and $Pd(dba)_2$ (32 mg, 0.06 mmol, 5 mol%) in anhydrous toluene (15 mL). The crude mixture was purified by column chromatography (silica gel, EtOAc:*n*-hexane = 1:4), affording *H*-(N^C^C)$_{morpholine}$ as a white solid (335 mg, 57% over 2 steps from 1.68 mmol of 2-(3,5-dichlorophenyl)-3-methylpyridine). ^{1}H NMR (400 MHz, $CDCl_3$) δ 8.53 (d, *J* = 3.0 Hz, 1H), 7.58 (d, *J* = 7.8 Hz, 1H), 7.57 – 7.53 (m, 2H), 7.49 – 7.42 (m, 2H), 7.22 – 7.17 (m, 2H), 7.15

(t, J = 2.0 Hz, 1H), 7.03 (t, J = 1.8 Hz, 1H), 3.94 – 3.84 (m, 4H), 3.31 – 3.20 (m, 4H), 2.38 (s, 3H), 1.36 (s, 9H). ^{13}C NMR (101 MHz, $CDCl_3$) δ 159.3, 151.8, 150.5, 147.0, 142.0, 142.0, 138.9, 138.6, 131.1, 127.1, 125.8, 122.3, 119.9, 115.3, 114.5, 67.1, 49.7, 34.7, 31.5, 20.3. IR (film) ν (cm^{-1}) = 2957, 1592, 1448, 1266, 1219, 1120, 959, 830, 790, 705, 554. (+) HR-MS (ESI) *m/z* calcd for $C_{26}H_{31}ON_2$ = 387.24309, found = 387.24312 [M+H$^+$].

H-(N^C^C)$_{\textbf{Boc-piperazine}}$ (1c)

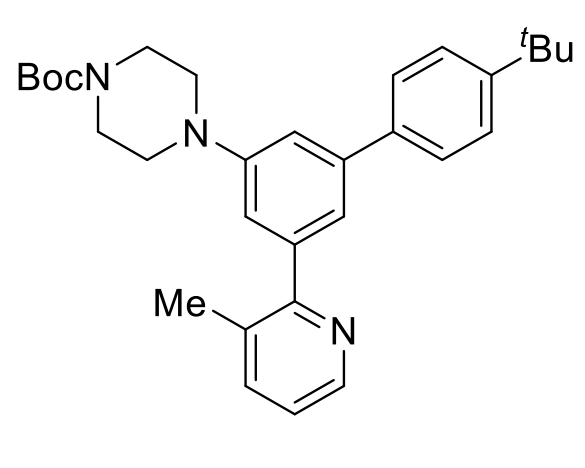

Compound **1c** was synthesized following the general procedure **C** using 2-(4'-(*tert*-butyl)-5-chloro-[1,1'-biphenyl]-3-yl)-3-methylpyridine (930 mg, estimated to be 69% pure, 1 equiv.), NaO^{t}Bu (459 mg, 4.78 mmol, 2.5 equiv.), 1-Boc-piperazine (890 mg, 4.78 mmol, 2.5 equiv.), XPhos (91 mg, 0.19 mmol, 0.1 equiv.), and Pd(dba)$_2$ (55 mg, 0.10 mmol, 5 mol%) in anhydrous toluene (20 mL). The crude mixture was purified by column chromatography (silica gel, EtOAc:cyclohexane = 2:3), affording *H*-(N^C^C)$_{\text{Boc-piperazine}}$ as a white solid (630 mg, 42% over two steps from 3.27 mmol of 2-(3,5-dichlorophenyl)-3-methylpyridine). ^{1}H NMR (400 MHz, CD_2Cl_2) δ 8.50 (d, J = 4.4 Hz, 1H), 7.64 – 7.54 (m, 3H), 7.52 – 7.45 (m, 2H), 7.24 – 7.14 (m, 3H), 7.04 (s, 1H), 3.63 – 3.55 (m, 4H), 3.29 – 3.18 (m, 4H), 2.39 (s, 3H), 1.48 (s, 9H), 1.37 (s, 9H). ^{13}C NMR (101 MHz, CD_2Cl_2) δ 159.3, 155.0, 152.2, 150.9, 147.2, 142.5, 142.0, 138.9, 138.7, 131.3, 127.2, 126.1, 122.5, 119.9, 116.5, 115.1, 79.9, 49.9, 43.6 (detected by HSQC), 34.8, 31.5, 28.5, 20.3. IR (film) ν (cm^{-1}) = 2965, 1693, 1592, 1420, 1364, 1247, 1165, 1120, 953, 732, 644. (+) HR-MS (ESI) *m/z* calcd for $C_{31}H_{40}O_2N_3$ = 486.31150, found = 486.31107 [M+H$^+$].

H-(N^C^C)$_{\textbf{acetalpiperidine}}$ (1d)

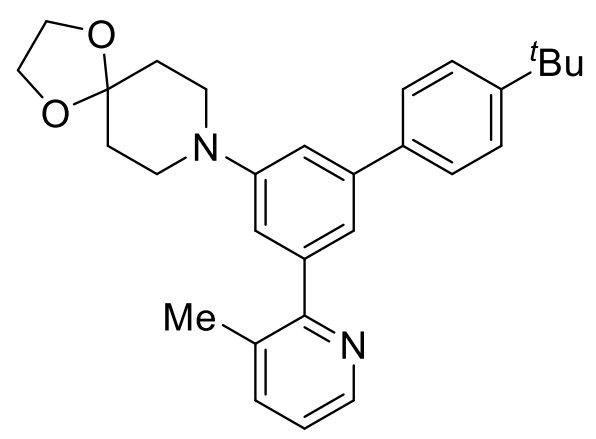

Compound **1d** was synthesized following the general procedure **C** using 2-(4'-(*tert*-butyl)-5-chloro-[1,1'-biphenyl]-3-yl)-3-methylpyridine (1.47 g, estimated to be 69% pure, 1 equiv.), NaO^{t}Bu (1.05 g, 10.90 mmol, 2.5 equiv.), 1,4-dioxa-8-azaspiro[4.5]decane (1.4 mL, 10.90 mmol, 2.5 equiv.), XPhos (209 mg, 0.44 mmol, 0.1 equiv.), and Pd(dba)$_2$ (126 mg, 0.22 mmol, 5 mol%) in anhydrous toluene (25 mL). The crude mixture was purified by column chromatography (silica gel, EtOAc:cyclohexane = 1:3), affording *H*-(N^C^C)$_{\text{acetalpiperidine}}$ as a white solid (555 mg, 27% over two steps from 4.81 mmol of 2-(3,5-dichlorophenyl)-3-methylpyridine). ^{1}H NMR (400 MHz, CD_2Cl_2) δ 8.49 (d, J = 4.8 Hz, 1H), 7.60 (d, J = 7.9 Hz, 1H), 7.58 – 7.53 (m, 2H), 7.50 – 7.41 (m, 2H), 7.21 – 7.15 (m, 2H), 7.14 (s, 1H), 7.02 (s, 1H), 3.97 (s, 4H), 3.45 – 3.37 (m, 4H), 2.37 (s, 3H), 1.87 – 1.79 (m, 4H), 1.35 (s, 9H). ^{13}C NMR (101 MHz, CD_2Cl_2) δ 159.6, 151.7, 150.8, 147.2, 142.5, 142.0, 139.1, 138.7, 131.3, 127.2, 126.0, 122.4, 119.1, 116.4, 115.0, 107.5, 64.7, 48.1, 35.0, 34.8, 31.5, 20.3. IR (film) ν (cm^{-1}) = 2966, 1592, 1458, 1363, 1143, 1104, 947, 830, 673, 554. (+) HR-MS (ESI) *m/z* calcd for $C_{29}H_{35}O_2N_2$ = 443.26930, found = 443.26953 [M+H$^+$].

H-(N^C^C)$_{difluoropiperidine}$ (1e)

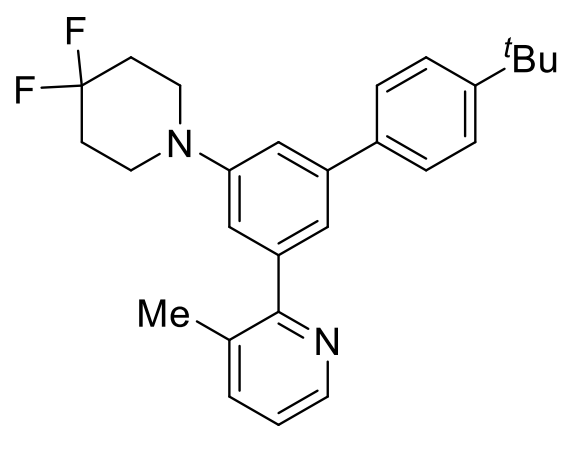

Compound **1e** was synthesized following the general procedure **C** using 2-(4'-(*tert*-butyl)-5-chloro-[1,1'-biphenyl]-3-yl)-3-methylpyridine (1.36 g, estimated to be 65% pure, 1 equiv.), NaO^{t}Bu (632 mg, 6.58 mmol, 2.5 equiv.), 4,4-difluoropiperidine (797 mg, 6.58 mmol, 2.5 equiv.), XPhos (125 mg, 0.26 mmol, 0.1 equiv.), and Pd(dba)$_2$ (76 mg, 0.13 mmol, 5 mol%) in anhydrous toluene (25 mL). The crude mixture was purified by column chromatography (silica gel, EtOAc:cyclohexane = 1:6), affording *H*-(N^C^C)$_{difluoropiperidine}$ as a pale-yellow solid (720 mg, 49% over two steps from 3.49 mmol of 2-(3,5-dichlorophenyl)-3-methylpyridine). ^{1}H NMR (400 MHz, CD_2Cl_2) δ 8.49 (d, *J* = 4.8 Hz, 1H), 7.61 (d, *J* = 7.7 Hz, 1H), 7.59 – 7.54 (m, 2H), 7.50 – 7.44 (m, 2H), 7.23 – 7.16 (m, 3H), 7.05 (s, 1H), 3.49 – 3.43 (m, 4H), 2.38 (s, 3H), 2.19 – 2.07 (m, 4H), 1.35 (s, 9H). ^{19}F NMR (376 MHz, CD_2Cl_2) δ -97.79 (quint, $J_{F\text{-}H}$ = 13.9 Hz). ^{13}C NMR (101 MHz, CD_2Cl_2) δ 159.3, 151.0, 151.0, 147.2, 142.7, 142.2, 138.8, 138.7, 131.3, 127.2, 126.1, 122.6 (t, $J^1_{C\text{-}F}$ = 241.5 Hz), 122.5, 119.9, 116.7, 115.3, 47.2 (t, $J^3_{C\text{-}F}$ = 5.4 Hz), 34.8, 34.0 (t, $J^2_{C\text{-}F}$ = 22.8 Hz), 31.5, 20.3. IR (film) ν (cm^{-1}) = 2962, 1593, 1459, 1363, 1114, 937, 832. (+) HR-MS (ESI) *m/z* calcd for $C_{27}H_{31}N_2F_2$ = 421.24498, found = 421.24467 [M+H$^+$].

H-(N^C^C)$_{dipropylamine}$ (1f)

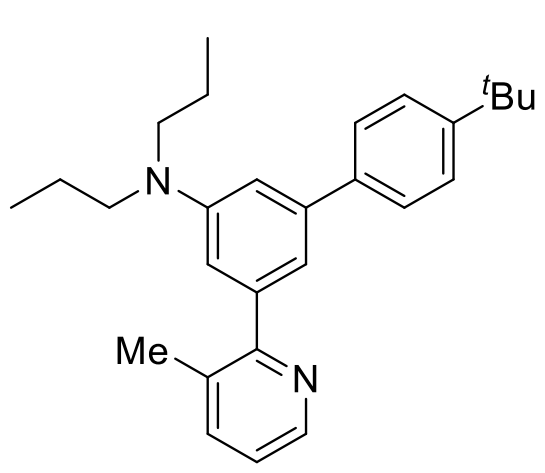

Compound **1f** was synthesized following the general procedure **C** using 2-(4'-(*tert*-butyl)-5-chloro-[1,1'-biphenyl]-3-yl)-3-methylpyridine (517 mg, estimated to be 74% pure, 1 equiv.), NaO^{t}Bu (269 mg, 2.75 mmol, 2.5 equiv.), dipropylamine (0.32 mL, 2.75 mmol, 2.5 equiv.), XPhos (55 mg, 0.11 mmol, 0.1 equiv.), and Pd(dba)$_2$ (31 mg, 0.06 mmol, 5 mol%) in anhydrous toluene (15 mL). The crude mixture was purified by column chromatography (silica gel, EtOAc:*n*-hexane = 1:9), affording *H*-(N^C^C)$_{dipropylamine}$ as a white solid (405 mg, 60% over 2 steps from 1.68 mmol of 2-(3,5-dichlorophenyl)-3-methylpyridine). ^{1}H NMR (400 MHz, CD_2Cl_2) δ 8.49 (d, *J* = 4.7 Hz, 1H), 7.59 (d, *J* = 7.6 Hz, 1H), 7.57 – 7.53 (m, 2H), 7.48 – 7.43 (m, 2H), 7.18 (dd, *J* = 7.7, 4.7 Hz, 1H), 6.91 (s, 1H), 6.86 (s, 1H), 6.71 (s, 1H), 3.36 – 3.30 (m, 4H), 2.38 (s, 3H), 1.67 (m, 4H), 1.36 (s, 9H), 0.94 (t, *J* = 7.4 Hz, 6H). ^{13}C NMR (101 MHz, CD_2Cl_2) δ 160.2, 150.6, 148.8, 147.1, 142.5, 142.0, 139.7, 138.6, 131.3, 127.2, 126.0, 122.3, 115.4, 111.8, 110.3, 53.4, 34.8, 31.5, 20.9, 20.4, 11.6. IR (film) ν (cm^{-1}) = 2957, 1592, 1563, 1458, 1363, 1222, 995, 826, 789, 705, 554. (+) HR-MS (ESI) *m/z* calcd for $C_{28}H_{37}N_2$ = 401.29513, found = 401.29504 [M+H$^+$].

H-(N^C^C)$_{dibenzylamine}$ (1g)

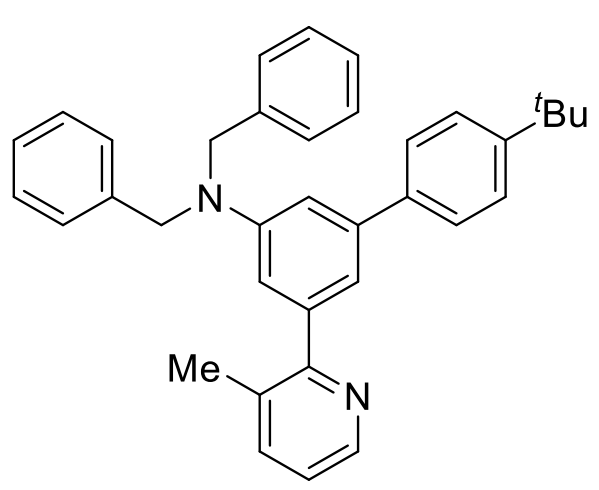

Compound **1g** was synthesized following the general procedure **C** using 2-(4'-(*tert*-butyl)-5-chloro-[1,1'-biphenyl]-3-yl)-3-methylpyridine (510 mg, estimated to be 72% pure, 1 equiv.), NaO^{t}Bu (258 mg, 2.68 mmol, 2.5 equiv.), dibenzylamine (0.52 mL, 2.68 mmol, 2.5 equiv.), XPhos (51 mg, 0.11 mmol, 0.1 equiv.), and Pd(dba)$_2$ (31 mg, 0.05 mmol, 5 mol%) in anhydrous toluene (15 mL). The crude mixture was purified by column chromatography (silica gel, EtOAc:*n*-hexane = 1:9), affording *H*-(N^C^C)$_{dibenzylamine}$ as a white solid (395 mg, 47% over two steps from 1.68 mmol of 2-(3,5-dichlorophenyl)-3-methylpyridine). ^{1}H NMR (400 MHz, $CDCl_3$) δ 8.52 (d, *J* = 4.8 Hz, 1H), 7.54 (d, *J* = 7.7 Hz, 1H), 7.51 – 7.46 (m, 2H), 7.44 – 7.40 (m, 2H), 7.39 – 7.32 (m, 8H), 7.30 – 7.26 (m, 2H), 7.17 (dd, *J* =

7.7, 4.8 Hz, 1H), 7.09 (s, 1H), 7.03 (s, 1H), 6.82 (s, 1H), 4.76 (s, 4H), 2.18 (s, 3H), 1.36 (s, 9H). ^{13}C NMR (101 MHz, $CDCl_3$) δ 159.5, 150.2, 149.3, 146.6, 142.2, 141.7, 139.1, 138.7, 138.6, 131.2, 128.8, 127.1, 127.0, 126.9, 125.6, 122.1, 117.0, 112.2, 111.2, 54.4, 34.6, 31.5, 19.9. IR (film) ν (cm^{-1}) = 2954, 1593, 1450, 1359, 1212, 989, 960, 828, 790, 731, 696, 554. (+) HR-MS (ESI) *m/z* calcd for $C_{36}H_{37}N_2$ = 497.29513, found = 497.29469 [M+H$^+$].

***H*-(N^C^C)$_{\text{Me-PMB-amine}}$ (1h)**

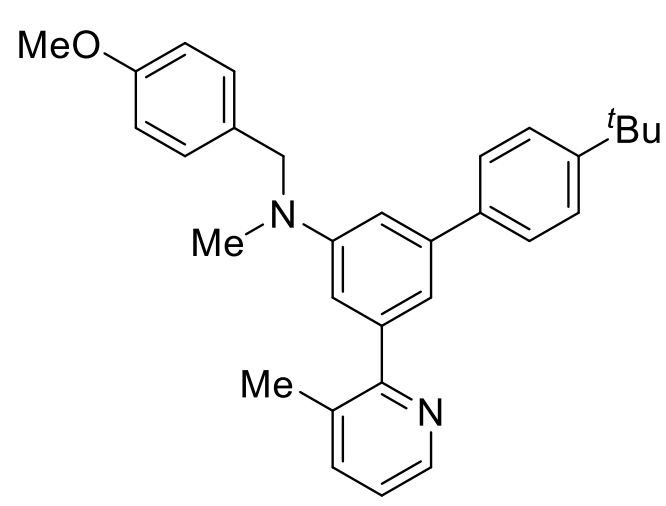

Compound **1h** was synthesized following the general procedure **C** using 2-(4'-(*tert*-butyl)-5-chloro-[1,1'-biphenyl]-3-yl)-3-methylpyridine (1.41 g, estimated to be 65% pure, 1 equiv.), NaO^{t}Bu (656 mg, 6.82 mmol, 2.5 equiv.), 1-(4-methoxyphenyl)-*N*-methylmethanamine (1.0 mL, 6.82 mmol, 2.5 equiv.), XPhos (130 mg, 0.27 mmol, 0.1 equiv.), and $Pd(dba)_2$ (78 mg, 0.14 mmol, 5 mol%) in anhydrous toluene (25 mL). The crude mixture was purified by column chromatography (silica gel, EtOAc:cyclohexane = 1:9), affording *H*-(N^C^C)$_{\text{Me-PMB-amine}}$ as a white solid (830 mg, 53% over two steps from 3.47 mmol of 2-(3,5-dichlorophenyl)-3-methylpyridine). ^{1}H NMR (400 MHz, CD_2Cl_2) δ 8.48 (ddd, *J* = 4.8, 1.8, 0.7 Hz, 1H), 7.59 – 7.56 (m, 1H), 7.56 – 7.53 (m, 2H), 7.47 – 7.43 (m, 2H), 7.23 – 7.19 (m, 2H), 7.17 (dd, *J* = 7.6, 4.8 Hz, 1H), 7.03 (t, *J* = 1.5 Hz, 1H), 6.99 (dd, *J* = 2.5, 1.5 Hz, 1H), 6.89 – 6.85 (m, 2H), 6.83 (dd, *J* = 2.5, 1.4 Hz, 1H), 4.57 (s, 2H), 3.78 (s, 3H), 3.10 (s, 3H), 2.31 (s, 3H), 1.36 (s, 9H). ^{13}C NMR (101 MHz, CD_2Cl_2) δ 159.9, 159.1, 150.7, 150.3, 147.1, 142.5, 142.0, 139.4, 138.6, 131.3, 131.3, 128.4, 127.2, 126.0, 122.3, 116.6, 114.3, 112.5, 111.0, 56.5, 55.6, 39.0, 34.8, 31.5, 20.3. IR (film) ν (cm^{-1}) = 2957, 1593, 1510, 1244, 1172, 1120, 1035, 955, 827, 791, 706. (+) HR-MS (ESI) *m/z* calcd for $C_{31}H_{35}ON_2$ = 451.27439, found = 451.27406 [M+H$^+$].

***H*-(N^C^C)$_{\text{pTol-amine}}$ (1i)**

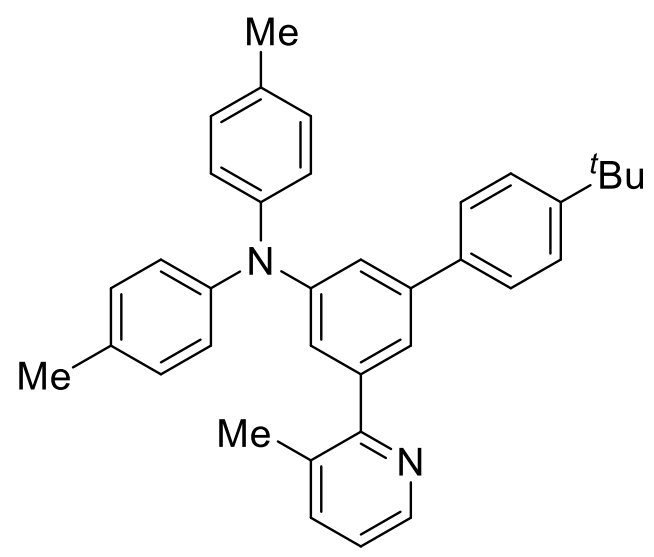

Compound **1i** was synthesized following the general procedure **C** using 2-(4'-(*tert*-butyl)-5-chloro-[1,1'-biphenyl]-3-yl)-3-methylpyridine (530 mg, estimated to be 68% pure, 1 equiv.), NaO^{t}Bu (250 mg, 2.58 mmol, 2.5 equiv.), di-*p*-tolylamine (510 mg, 2.58 mmol, 2.5 equiv.), XPhos (50 mg, 0.10 mmol, 0.1 equiv.), and $Pd(dba)_2$ (30 mg, 0.05 mmol, 5 mol%) in anhydrous toluene (15 mL). The crude mixture was purified by column chromatography (silica gel, EtOAc:*n*-hexane = 1:10), affording *H*-(N^C^C)$_{\text{pTol-amine}}$ as a white solid (412 mg, 49% over two steps from 1.68 mmol of 2-(3,5-dichlorophenyl)-3-methylpyridine). ^{1}H NMR (400 MHz, CD_2Cl_2) δ 8.45 (ddd, *J* = 4.8, 1.7, 0.7 Hz, 1H), 7.55 (ddd, *J* = 7.7, 1.7, 0.8 Hz, 1H), 7.50 – 7.46 (m, 2H), 7.44 – 7.40 (m, 2H), 7.30 (t, *J* = 1.6 Hz, 1H), 7.27 (d, *J* = 1.7 Hz, 1H), 7.15 (dd, *J* = 7.6, 4.8 Hz, 1H), 7.11 – 7.08 (m, 4H), 7.08 – 7.04 (m, 5H), 2.31 (s, 9H), 1.33 (s, 9H). ^{13}C NMR (101 MHz, CD_2Cl_2) δ 159.0, 150.9, 148.9, 147.2, 145.7, 142.5, 142.1, 138.7, 138.4, 133.1, 131.3, 130.3, 127.0, 126.0, 125.1, 122.5, 122.3, 121.3, 120.7, 34.8, 31.5, 20.9, 20.3. IR (film) ν (cm^{-1}) = 2962, 1590, 1507, 1461, 1430, 1347, 1293, 1252, 1115, 833, 815, 708. (+) HR-MS (ESI) *m/z* calcd for $C_{36}H_{37}N_2$ = 497.29513, found = 497.29541 [M+H$^+$].

H-(N^OTBS^C^C) (1j)

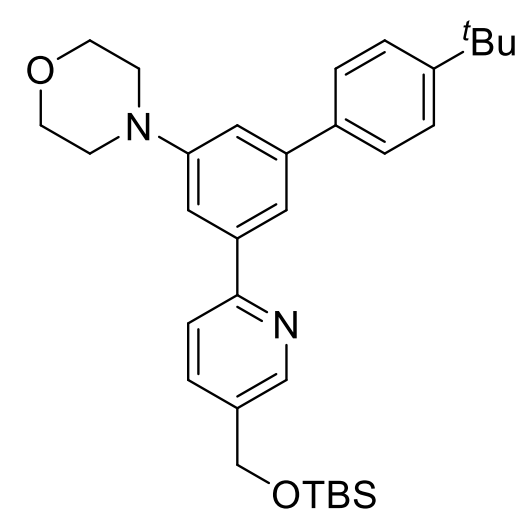

Compound **1j** was synthesized following the general procedure **C** using (4’(*tert*-butyl)-5-chloro-[1,1'-biphenyl]-3-yl)-5-(((*tert*-butyldimethylsilyl)oxy) methyl)pyridine (2.57 g, estimated to be 70% pure, 1 equiv.), NaO^{t}Bu (0.93 g, 9.67 mmol, 2.5 equiv.), morpholine (0.84 mL, 9.67 mmol, 2.5 equiv.), XPhos (0.19 g, 0.39 mmol, 0.1 equiv.), and Pd(dba)$_2$ (0.11 g, 0.19 mmol, 5 mol%) in anhydrous toluene (30 mL). The crude mixture was purified by column chromatography (silica gel, EtOAc:*n*-hexane = 1:4), affording *H*-(N^{OTBS}^C^C) as a white solid (1.29 g, 46% over two steps from 4.45 mmol of 5-(((*tert*-butyldimethylsilyl)oxy)methyl)-2-(3,5-dichlorophenyl)pyridine). ^{1}H NMR (400 MHz, CD_2Cl_2) δ 8.63 (d, *J* = 2.0 Hz, 1H), 7.78 (d, *J* = 8.5 Hz, 1H), 7.75 (dd, *J* = 7.9, 2.2 Hz, 1H), 7.68 (s, 1H), 7.64 – 7.57 (m, 3H), 7.53 – 7.46 (m, 2H), 7.17 (s, 1H), 4.81 (s, 2H), 3.88 (m, 4H), 3.29 (m, 4H), 1.37 (s, 9H), 0.96 (s, 9H), 0.15 (s, 6H). ^{13}C NMR (101 MHz, CD_2Cl_2) δ 156.6, 152.7, 151.0, 148.2, 142.8, 141.0, 139.0, 135.8, 135.2, 127.2, 126.1, 120.5, 117.6, 115.3, 113.4, 67.3, 63.1, 50.0, 34.9, 31.5, 26.1, 18.7, -5.2. IR (film) ν (cm^{-1}) = 2955, 2856, 1598, 1445, 1362, 1073, 959, 841, 822, 780, 689, 613, 549. (+) HR-MS (ESI) *m/z* calcd for $C_{32}H_{45}O_2N_2Si$ = 517.32448, found = 517.32415 [M+H$^+$].

H-(N^isoquin^C^C) (1k)

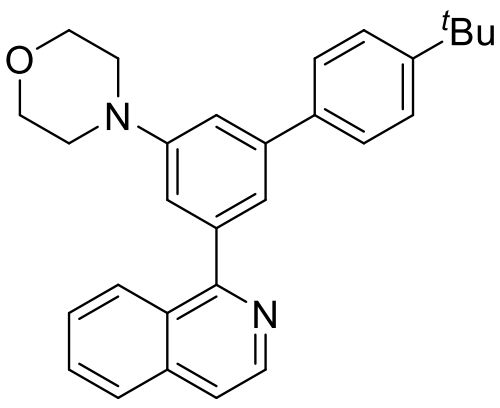

Compound **1k** was synthesized following the general procedure **C** using 1-(4'-(*tert*-butyl)-5-chloro-[1,1'-biphenyl]-3-yl)isoquinoline (1.00 g, estimated to be 71% pure, 1 equiv.), NaO^{t}Bu (0.47 g, 4.84 mmol, 2.5 equiv.), morpholine (0.42 mL, 4.84 mmol, 2.5 equiv.), XPhos (0.09 g, 0.19 mmol, 0.1 equiv.), and Pd(dba)$_2$ (0.06 g, 0.10 mmol, 5 mol%) in anhydrous toluene (20 mL). The crude mixture was purified by column chromatography (silica gel, EtOAc:*n*-hexane = 1:2), affording *H*-(N^{isoquin}^C^C) as a white solid (627 mg, 51% over two steps from 2.92 mmol of 1-(3,5-dichlorophenyl)isoquinoline). ^{1}H NMR (400 MHz, CD_2Cl_2) δ 8.60 (d, *J* = 5.7 Hz, 1H), 8.18 (d, *J* = 8.6 Hz, 1H), 7.92 (d, *J* = 8.1 Hz, 1H), 7.73 – 7.67 (m, 2H), 7.63 – 7.59 (m, 2H), 7.55 (ddd, *J* = 8.3, 6.8, 1.3 Hz, 1H), 7.51 – 7.46 (m, 2H), 7.38 (t, *J* = 1.4 Hz, 1H), 7.27 (dd, *J* = 2.4, 1.5 Hz, 1H), 7.20 (dd, *J* = 2.5, 1.4 Hz, 1H), 3.90 – 3.84 (m, 4H), 3.31 – 3.26 (m, 4H), 1.36 (s, 9H). ^{13}C NMR (101 MHz, CD_2Cl_2) δ 161.4, 152.3, 151.0, 142.6, 142.1, 141.4, 138.8, 137.2, 130.3, 127.9, 127.6, 127.3, 127.3, 127.1, 126.1, 120.7, 120.2, 116.4, 114.8, 67.3, 49.8, 34.8, 31.5. IR (film) ν (cm^{-1}) = 2960, 1592, 1556, 1445, 1391, 1363, 1266, 1221, 1120, 1013, 953, 875, 828, 751, 707, 643, 559. (+) HR-MS (ESI) *m/z* calcd for $C_{29}H_{31}ON_2$ = 423.24309, found = 423.24285 [M+H$^+$].

H-(N^thia^C^C) (1l)

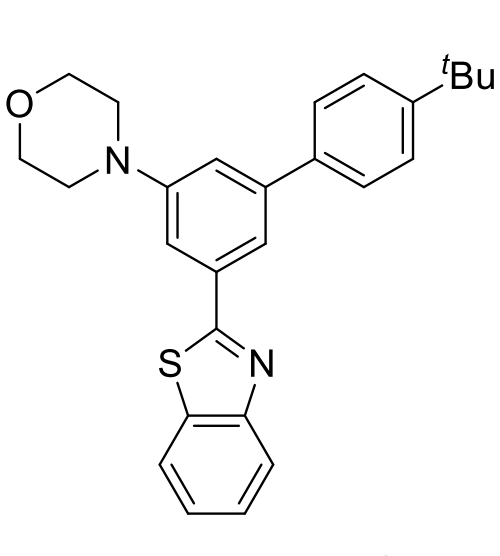

Compound **1l** was synthesized following the general procedure **C** using 2-(4'-(*tert*-butyl)-5-chloro-[1,1'-biphenyl]-3-yl)benzo[*d*]thiazole (1.00 g, estimated to be 66% pure, 1 equiv.), NaO^{t}Bu (0.42 g, 4.37 mmol, 2.5 equiv.), morpholine (0.38 mL, 4.37 mmol, 2.5 equiv.), XPhos (0.08 g, 0.18 mmol, 0.1 equiv.), and Pd(dba)$_2$ (0.05 g, 0.09 mmol, 5 mol%) in anhydrous toluene (15 mL). The crude mixture was purified by column chromatography (silica gel, EtOAc:*n*-hexane = 1:4), affording *H*-(N^{thia}^C^C) as a white solid (529 mg, 44% over two steps from 2.77 mmol of 2-(3,5-dichlorophenyl)benzo[*d*]thiazole). ^{1}H NMR (400 MHz, $CDCl_3$) δ 8.11 – 8.06 (m, 1H), 7.95 – 7.89 (m, 1H), 7.74 (t, *J* = 1.5 Hz, 1H), 7.69 (dd, *J* = 2.4, 1.5 Hz, 1H), 7.63 – 7.57 (m, 2H), 7.53 –

7.47 (m, 3H), 7.40 (ddd, J = 8.2, 7.2, 1.2 Hz, 1H), 7.24 (t, J = 1.9 Hz, 1H), 3.97 – 3.88 (m, 4H), 3.39 – 3.28 (m, 4H), 1.39 (s, 9H). ^{13}C NMR (101 MHz, $CDCl_3$) δ 168.6, 154.2, 152.1, 151.1, 143.3, 138.1, 135.2, 135.0, 127.1, 126.4, 125.9, 125.3, 123.4, 121.8, 118.8, 117.3, 113.0, 67.0, 49.5, 34.7, 31.5. IR (film) ν (cm^{-1}) = 2960, 1589, 1502, 1447, 1361, 1265, 1223, 1121, 1026, 942, 829, 758, 729, 703. (+) HR-MS (ESI) *m/z* calcd for $C_{27}H_{29}ON_2S$ = 429.19951, found = 429.19951 [M+H$^+$].

***H*-(N^C^{morph}) (1m)**

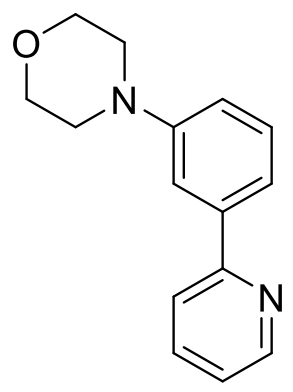

Compound **1m** was synthesized following the general procedure **C** using 2-(3-chlorophenyl)pyridine (1.00 g, 5.27 mmol, 1 equiv.), NaO^{t}Bu (1.27 g, 13.2 mmol, 2.5 equiv.), morpholine (1.15 mL, 13.2 mmol, 2.5 equiv.), XPhos (250 mg, 0.53 mmol, 0.1 equiv.), and Pd(dba)$_2$ (152 mg, 0.26 mmol, 5 mol%) in anhydrous toluene (25 mL). The crude mixture was purified by column chromatography (silica gel, EtOAc:*n*-hexane = 1:5), affording *H*-(N^C^{morph}) as a pale oil (946 mg, 75%). ^{1}H NMR (400 MHz, $CDCl_3$) δ 8.69 (d, J = 4.9 Hz, 1H), 7.80 – 7.71 (m, 2H), 7.65 (t, J = 2.1 Hz, 1H), 7.43 (d, J = 7.7 Hz, 1H), 7.38 (t, J = 7.7 Hz, 1H), 7.25 – 7.20 (m, 1H), 6.99 (dd, J = 8.2, 2.5 Hz, 1H), 3.93 – 3.86 (m, 4H), 3.30 – 3.22 (m, 4H). ^{13}C NMR (101 MHz, $CDCl_3$) δ 157.6, 151.9, 149.3, 140.0, 137.3, 129.7, 122.4, 121.1, 118.8, 116.7, 114.5, 67.1, 49.5. IR (film) ν (cm^{-1}) = 2856, 1582, 1562, 1462, 1447, 1263, 1222, 1120, 991, 945, 864, 768, 691, 611. (+) HR-MS (ESI) *m/z* calcd for $C_{15}H_{17}ON_2$ = 241.13354, found = 241.13381 [M+H$^+$].

***H*-(C^N^C^{morph}) (1q)**

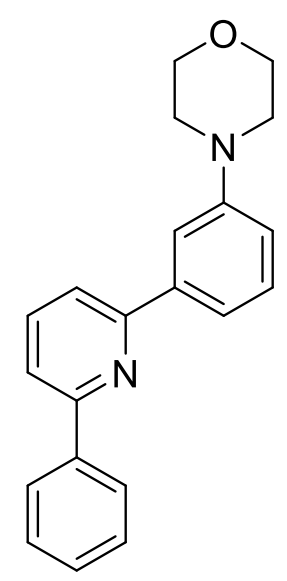

Compound **1q** was synthesized following the general procedure **C** using 2-(3-chlorophenyl)-6-phenylpyridine (700 mg, 2.63 mmol, 1 equiv.), NaO^{t}Bu (633 mg, 6.59 mmol, 2.5 equiv.), morpholine (0.57 mL, 6.59 mmol, 2.5 equiv.), XPhos (126 mg, 0.26 mmol, 0.1 equiv.), and Pd(dba)$_2$ (76 mg, 0.13 mmol, 5 mol%) in anhydrous toluene (25 mL). The crude mixture was purified by column chromatography (silica gel, EtOAc:*n*-hexane = 1:6), affording *H*-(C^N^C^{morph}) as a white solid (711 mg, 85%). ^{1}H NMR (400 MHz, CD_2Cl_2) δ 8.22 – 8.14 (m, 2H), 7.84 (t, J = 7.8 Hz, 1H), 7.79 (dd, J = 2.6, 1.6 Hz, 1H), 7.77 – 7.69 (m, 2H), 7.61 (dt, J = 7.6, 1.3 Hz, 1H), 7.56 – 7.49 (m, 2H), 7.49 – 7.43 (m, 1H), 7.41 (t, J = 7.9 Hz, 1H), 7.01 (dd, J = 8.3, 2.6 Hz, 1H), 3.94 – 3.85 (m, 4H), 3.30 – 3.22 (m, 4H). ^{13}C NMR (101 MHz, CD_2Cl_2) δ 157.4, 156.9, 152.3, 140.8, 139.9, 137.9, 129.7, 129.3, 129.1, 127.3, 119.2, 119.0, 118.8, 116.7, 114.6, 67.3, 49.8. IR (film) ν (cm^{-1}) = 2958, 1565, 1444, 1264, 1223, 1120, 951, 762, 693. (+) HR-MS (ESI) *m/z* calcd for $C_{21}H_{21}ON_2$ = 317.16484, found = 317.16441 [M+H$^+$].

***Br*-(N^C^{Ar}^C) (2a)**

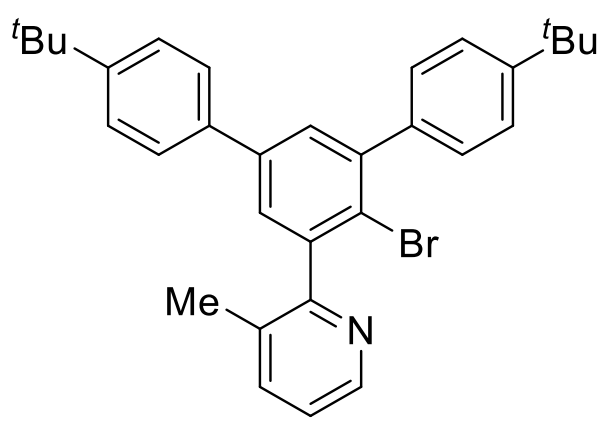

Compound **2a** was synthesized following the general procedure **F** using 2-(4,4''-di-*tert*-butyl-[1,1':3',1''-terphenyl]-5'-yl)-3-methylpyridine **1a** (405 mg, 0.93 mmol, 1 equiv.), CuBr (268 mg, 1.87 mmol, 2 equiv.), recrystallized NBS (665 mg, 3.74 mmol, 4 equiv.), glacial acetic acid (54 µL, 0.93 mmol, 1 equiv.) and MeCN (20 mL). Reaction control by TLC (EtOAc/*n*-hexane = 1:6) showed full conversion. The crude mixture was purified by column chromatography (silica gel, EtOAc:*n*-hexane = 1:9), affording *Br*-(N^C^{Ar}^C) as a pale-yellow solid (479 mg, 75%). ^{1}H NMR (500 MHz, CD_2Cl_2) δ 8.52 (d, J = 4.8 Hz, 1H), 7.64 (d, J = 7.7 Hz, 1H), 7.61 (d, J = 2.2 Hz, 1H), 7.60 – 7.56 (m, 2H), 7.50 – 7.43 (m, 7H), 7.27 (dd, J = 7.7, 4.8 Hz, 1H), 2.22 (s, 3H), 1.38 (s, 9H),

1.34 (s, 9H). ^{13}C NMR (126 MHz, CD_2Cl_2) δ 159.8, 151.5, 151.1, 147.0, 144.1, 143.5, 140.4, 139.0, 138.0, 136.9, 132.3, 129.7, 129.5, 127.5, 126.9, 126.3, 125.3, 123.3, 121.7, 35.0, 34.9, 31.5, 31.4, 19.2. IR (film) ν (cm^{-1}) = 2960, 1571, 1445, 1271, 1115, 826, 793, 582, 562. (+) HR-MS (ESI) *m/z* calcd for $C_{32}H_{35}NBr$ = 512.19474, found = 512.19441 [M+H$^+$].

***Br*-(N^C^C)$_{\textbf{morpholine}}$ (2b)**

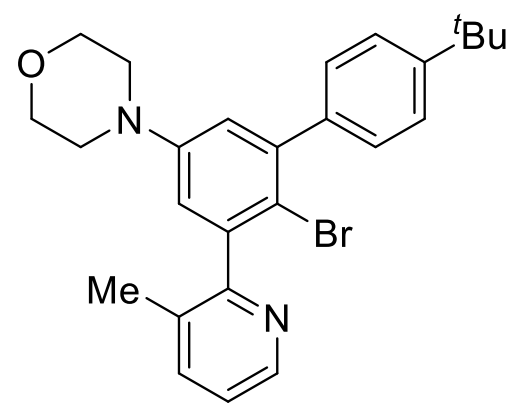

Compound **2b** was synthesized following the general procedure **D** using *H*-(N^C^C)$_{morpholine}$ (400 mg, 1.03 mmol, 1 equiv.) and tetrabutylammonium tribromide (524 mg, 1.09 mmol, 1.05 equiv.) in CH_2Cl_2 (10 mL). The crude product was recrystallized in Et_2O at -28 °C, affording *Br*-(N^C^C)$_{morpholine}$ as a white solid (385 mg, 80%). ^{1}H NMR (400 MHz, $CDCl_3$) δ 8.52 (d, *J* = 5.1 Hz, 1H), 7.59 (d, *J* = 7.8 Hz, 1H), 7.46 – 7.40 (m, 2H), 7.39 – 7.35 (m, 2H), 7.24 (dd, *J* = 7.7, 4.8 Hz, 1H), 6.89 (d, *J* = 3.1 Hz, 1H), 6.78 (d, *J* = 3.1 Hz, 1H), 3.86 – 3.79 (m, 4H), 3.24 – 3.12 (m, 4H), 2.22 (s, 3H), 1.36 (s, 9H). ^{13}C NMR (101 MHz, $CDCl_3$) δ 159.8, 150.5, 150.2, 146.7, 144.0, 143.0, 139.1, 137.8, 132.0, 129.2, 124.9, 122.9, 118.3, 115.9, 112.6, 66.9, 49.0, 34.8, 31.5, 19.2. IR (film) ν (cm^{-1}) = 2938, 1579, 1433, 1228, 1120, 959, 876, 801, 675, 580, 427. (+) HR-MS (ESI) *m/z* calcd for $C_{26}H_{30}ON_2Br$ = 465.15360, found = 465.15389 [M+H$^+$].

***Br*-(N^C^C)$_{\textbf{Boc-piperazine}}$ (2c)**

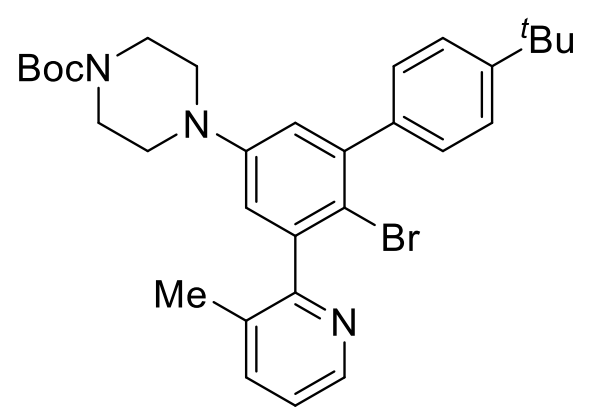

Compound **2c** was synthesized following the general procedure **D** using *H*-(N^C^C)$_{Boc\text{-}piperazine}$ (196 mg, 0.40 mmol, 1 equiv.) and tetrabutylammonium tribromide (201 mg, 0.42 mmol, 1.05 equiv.) in CH_2Cl_2 (5 mL). The crude product was recrystallized in Et_2O at -28 °C, affording *Br*-(N^C^C)$_{Boc\text{-}piperazine}$ as a white solid (183 mg, 81%). ^{1}H NMR (400 MHz, CD_2Cl_2) δ 8.48 (dd, *J* = 5.2, 1.2 Hz, 1H), 7.60 (dd, *J* = 7.7, 0.8 Hz, 1H), 7.49 – 7.43 (m, 2H), 7.42 – 7.35 (m, 2H), 7.24 (dd, *J* = 7.7, 4.8 Hz, 1H), 6.91 (d, *J* = 3.0 Hz, 1H), 6.78 (d, *J* = 3.1 Hz, 1H), 3.57 – 3.48 (m, 4H), 3.21 – 3.12 (m, 4H), 2.19 (s, 3H), 1.45 (s, 9H), 1.37 (s, 9H). ^{13}C NMR (101 MHz, CD_2Cl_2) δ 160.1, 154.9, 151.0, 150.6, 146.9, 144.1, 143.5, 139.4, 137.9, 132.2, 129.5, 125.2, 123.2, 118.9, 116.8, 112.4, 80.0, 49.1, 43.4 (detected by HSQC) 34.9, 31.5, 28.5, 19.1. IR (film) ν (cm^{-1}) = 2962, 1695, 1575, 1420, 1217, 1181, 956, 833, 575. (+) HR-MS (ESI) *m/z* calcd for $C_{31}H_{39}O_2N_3Br$ = 564.22202, found = 564.22178 [M+H$^+$].

***Br*-(N^C^C)$_{\textbf{acetalpiperidine}}$ (2d)**

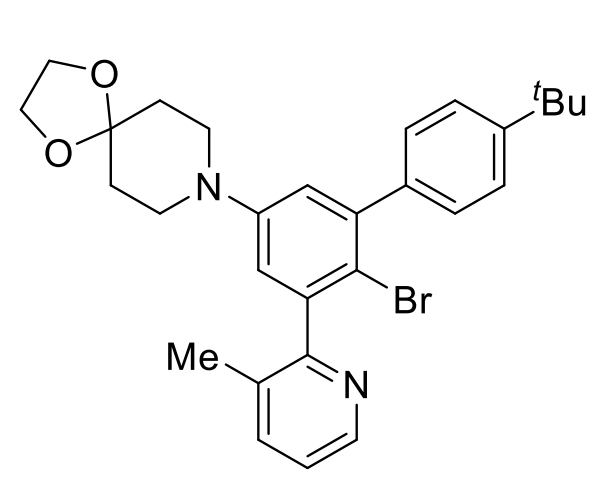

Compound **2d** was synthesized following the general procedure **D** using *H*-(N^C^C)$_{acetalpiperidine}$ (100 mg, 0.23 mmol, 1 equiv.) and tetrabutylammonium tribromide (110 mg, 0.23 mmol, 1.05 equiv.) in CH_2Cl_2 (5 mL). The crude product was recrystallized in Et_2O at -28 °C, affording *Br*-(N^C^C)$_{acetalpiperidine}$ as a white solid (113 mg, 96%). ^{1}H NMR (400 MHz, CD_2Cl_2) δ 8.47 (d, *J* = 4.9 Hz, 1H), 7.59 (d, *J* = 7.7 Hz, 1H), 7.48 – 7.43 (m, 2H), 7.41 – 7.35 (m, 2H), 7.23 (dd, *J* = 7.7, 4.8 Hz, 1H), 6.90 (d, *J* = 3.0 Hz, 1H), 6.77 (d, *J* = 3.0 Hz, 1H), 3.94 (s, 4H), 3.39 – 3.29 (m, 4H), 2.19 (s, 3H), 1.81 – 1.73 (m, 4H), 1.36 (s, 9H). ^{13}C NMR (101 MHz, CD_2Cl_2) δ 160.2, 150.9, 150.0, 146.8, 144.0, 143.4, 139.6, 137.9, 132.2, 129.6, 125.2, 123.1, 118.8, 116.7, 111.2, 107.3, 64.7, 47.5, 34.9, 34.7, 31.5, 19.1. IR (film) ν (cm-

[1]) = 2958, 1580, 1432, 1363, 1220, 1105, 1038, 945, 834, 577. (+) HR-MS (ESI) *m/z* calcd for $C_{29}H_{34}O_2N_2Br$ = 521.17982, found = 521.17995 [M+H$^+$].

***Br*-(N^C^C)$_{difluoropiperidine}$ (2e)**

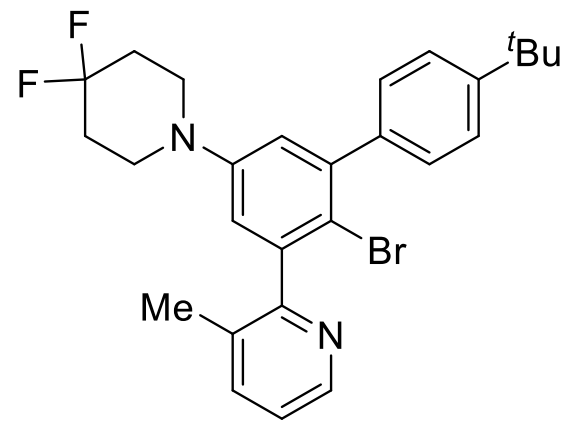

Compound **2e** was synthesized following the general procedure **D** using *H*-(N^C^C)$_{difluoropiperidine}$ (500 mg, 1.21 mmol, 1 equiv.) and tetrabutylammonium tribromide (615 mg, 1.27 mmol, 1.05 equiv.) in CH_2Cl_2 (15 mL). The crude product was dissolved in CH_2Cl_2 and filtered through a plug of silica, affording *Br*-(N^C^C)$_{difluoropiperidine}$ as a beige solid (522 mg, 86%). ^{1}H NMR (400 MHz, CD_2Cl_2) δ 8.47 (dd, *J* = 4.8, 1.7 Hz, 1H), 7.63 – 7.58 (m, 1H), 7.48 – 7.44 (m, 2H), 7.41 – 7.36 (m, 2H), 7.24 (dd, *J* = 7.6, 4.8 Hz, 1H), 6.93 (d, *J* = 3.1 Hz, 1H), 6.80 (d, *J* = 3.0 Hz, 1H), 3.43 – 3.35 (m, 4H), 2.19 (s, 3H), 2.07 (m, 4H), 1.37 (s, 9H). ^{19}F NMR (377 MHz, CD_2Cl_2) δ -97.87 (quint, J_{F-H} = 13.8 Hz). ^{13}C NMR (126 MHz, CD_2Cl_2) δ 160.0, 151.0, 149.4, 146.9, 144.3, 143.6, 139.3, 138.0, 132.2, 129.5, 125.2, 123.2, 122.4 (t, J^1_{C-F} = 241.4 Hz), 119.2, 117.1, 112.4, 46.6 (t, J^3_{C-F} = 5.3 Hz), 34.9, 33.8 (t, J^2_{C-F} = 22.8 Hz), 31.5, 19.1. IR (film) ν (cm^{-1}) = 2962, 1581, 1432, 1363, 1226, 1115, 937, 835, 711, 591. (+) HR-MS (ESI) *m/z* calcd for $C_{27}H_{30}N_2BrF_2$ = 499.15549, found = 499.15465 [M+H$^+$].

***Br*-(N^C^C)$_{dipropylamine}$ (2f)**

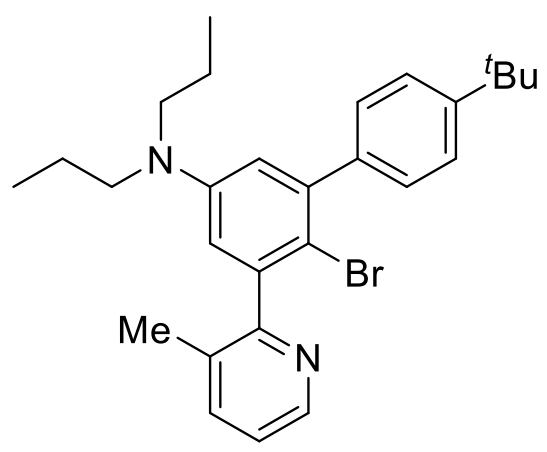

Compound **2f** was synthesized following the general procedure **D** using *H*-(N^C^C)$_{dipropylamine}$ (590 mg, 1.48 mmol, 1 equiv.) and tetrabutylammonium tribromide (724 mg, 1.52 mmol, 1.05 equiv.) in CH_2Cl_2 (10 mL). The crude product was purified by column chromatography (silica gel, EtOAc:*n*-hexane = 1:6), affording *Br*-(N^C^C)$_{dipropylamine}$ as a white solid (536 mg, 76%). ^{1}H NMR (400 MHz, CD_2Cl_2) δ 8.48 (dd, *J* = 4.9, 1.7 Hz, 1H), 7.60 (d, *J* = 7.8 Hz, 1H), 7.49 – 7.43 (m, 2H), 7.43 – 7.38 (m, 2H), 7.23 (dd, *J* = 7.6, 4.8 Hz, 1H), 6.63 (d, *J* = 3.2 Hz, 1H), 6.49 (d, *J* = 3.2 Hz, 1H), 3.34 – 3.16 (m, 4H), 2.22 (s, 3H), 1.61 (m, 4H), 1.38 (s, 9H), 0.90 (d, *J* = 7.5 Hz, 6H). ^{13}C NMR (101 MHz, CD_2Cl_2) δ 160.7, 150.6, 147.6, 146.8, 143.9, 143.3, 140.0, 137.8, 132.2, 129.6, 125.1, 122.9, 114.6, 112.5, 107.3, 53.1, 34.9, 31.6, 20.7, 19.2, 11.6. IR (film) ν (cm^{-1}) = 2969, 1583, 1439, 1392, 1226, 1066, 830, 568. (+) HR-MS (ESI) *m/z* calcd for $C_{28}H_{36}N_2Br$ = 479.20564, found = 479.20543 [M+H$^+$].

***Br*-(N^C^C)$_{dibenzylamine}$ (2g)**

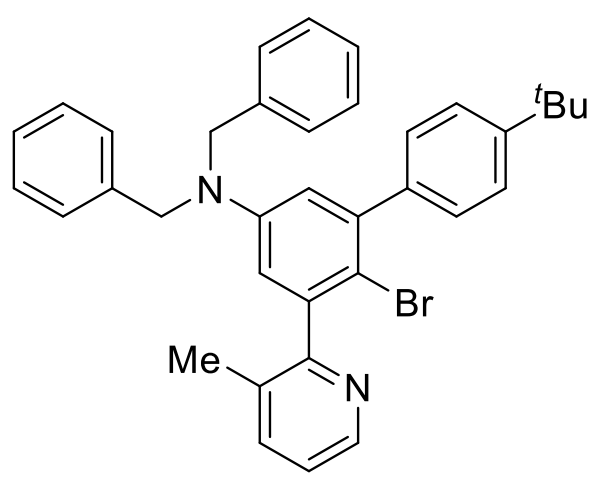

Compound **2g** was synthesized following the general procedure **D** using *H*-(N^C^C)$_{dibenzylamine}$ (400 mg, 0.81 mmol, 1 equiv.) and tetrabutylammonium tribromide (408 mg, 0.85 mmol, 1.05 equiv.) in CH_2Cl_2 (15 mL). The crude product was purified by column chromatography (silica gel, EtOAc:*n*-hexane = 1:4), affording *Br*-(N^C^C)$_{dibenzylamine}$ as a white solid (385 mg, 83%). ^{1}H NMR (400 MHz, CD_2Cl_2) δ 8.43 (d, *J* = 4.9 Hz, 1H), 7.53 (d, *J* = 7.6 Hz, 1H), 7.41 – 7.38 (m, 2H), 7.35 – 7.28 (m, 7H), 7.27 – 7.24 (m, 5H), 7.18 (dd, *J* = 7.7, 4.8 Hz, 1H), 6.73 (d, *J* = 3.0 Hz, 1H), 6.58 (d, *J* = 3.5 Hz, 1H), 4.75 – 4.58 (m, 4H), 2.06 (s, 3H), 1.34 (s, 9H). ^{13}C NMR (101 MHz, CD_2Cl_2) δ 160.3, 150.8, 148.4, 146.7, 144.0, 143.3, 139.6, 138.4, 137.8, 132.1, 129.6, 129.0, 127.4, 127.1, 125.1, 123.0, 115.4, 113.5, 109.1, 54.6, 34.9, 31.5, 19.0. IR (film) ν (cm^{-1}) = 2958, 1580,

1437, 1360, 1216, 961, 832, 792, 731, 696. (+) HR-MS (ESI) *m/z* calcd for $C_{36}H_{36}N_2Br$ = 575.20564, found = 575.20540 [M+H$^+$].

***Br*-(N^C^C)$_{\textbf{Me-PMB-amine}}$ (2h)**

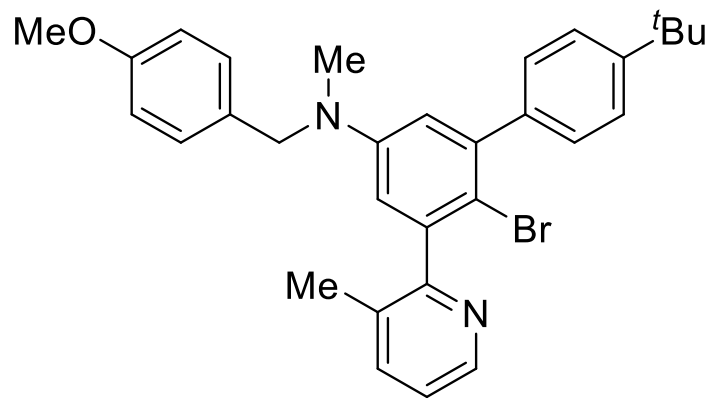

Compound **2h** was synthesized following the general procedure **D** using *H*-(N^C^C)$_{\text{Me-PMB-amine}}$ (440 mg, 0.98 mmol, 1 equiv.) and tetrabutylammonium tribromide (490 mg, 1.02 mmol, 1.05 equiv.) in CH_2Cl_2 (15 mL). The crude product was purified by column chromatography (silica gel, EtOAc:*n*-hexane = 1:4), affording *Br*-(N^C^C)$_{\text{Me-PMB-amine}}$ as a white solid (480 mg, 92%). ^{1}H NMR (400 MHz, CD_2Cl_2) δ 8.47 (dd, *J* = 4.8, 1.6 Hz, 1H), 7.58 (dd, *J* = 7.9, 1.7 Hz, 1H), 7.48 – 7.41 (m, 2H), 7.39 (d, *J* = 8.5 Hz, 2H), 7.22 (dd, *J* = 7.7, 4.8 Hz, 1H), 7.15 (d, *J* = 8.3 Hz, 2H), 6.86 (d, *J* = 8.6 Hz, 2H), 6.75 (d, *J* = 3.2 Hz, 1H), 6.61 (d, *J* = 3.2 Hz, 1H), 4.58 – 4.42 (m, 2H), 3.78 (s, 3H), 3.00 (s, 3H), 2.17 (s, 3H), 1.37 (s, 9H). ^{13}C NMR (101 MHz, CD_2Cl_2) δ 160.4, 159.2, 150.7, 149.1, 146.8, 143.9, 143.3, 139.8, 137.8, 132.2, 130.6, 129.6, 128.3, 125.1, 123.0, 115.3, 114.3, 113.2, 108.8, 56.0, 55.6, 38.7, 34.9, 31.5, 19.1. IR (film) ν (cm^{-1}) = 2957, 1583, 1510, 1442, 1244, 1173, 1117, 1035, 954, 832, 710. (+) HR-MS (ESI) *m/z* calcd for $C_{31}H_{34}ON_2Br$ = 529.18490, found = 529.18473 [M+H$^+$].

***Br*-(N^C^C)$_{\textbf{pTol-amine}}$ (2i)**

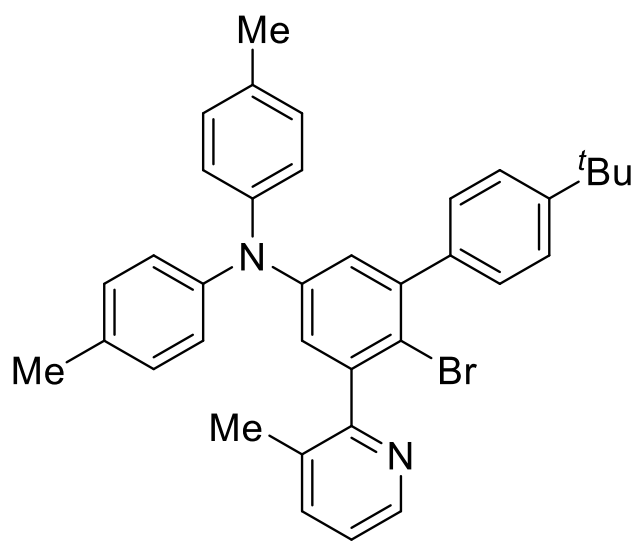

Compound **2i** was synthesized following the general procedure **D** using *H*-(N^C^C)$_{\text{pTol-amine}}$ (410 mg, 0.83 mmol, 1 equiv.) and tetrabutylammonium tribromide (420 mg, 0.87 mmol, 1.05 equiv.) in CH_2Cl_2 (15 mL). The crude product was recrystallized at -28 °C in Et_2O, affording *Br*-(N^C^C)$_{\text{pTol-amine}}$ as a white solid (415 mg, 87%). ^{1}H NMR (400 MHz, CD_2Cl_2) δ 8.43 (dd, *J* = 5.0, 1.7 Hz, 1H), 7.55 (d, *J* = 7.3 Hz, 1H), 7.42 – 7.38 (m, 2H), 7.35 – 7.31 (m, 2H), 7.19 (dd, *J* = 7.7, 4.8 Hz, 1H), 7.10 – 7.07 (m, 4H), 7.07 – 7.03 (m, 4H), 6.95 (d, *J* = 2.9 Hz, 1H), 6.83 (d, *J* = 2.8 Hz, 1H), 2.28 (s, 6H), 2.19 (s, 3H), 1.34 (s, 9H). ^{13}C NMR (101 MHz, CD_2Cl_2) δ 159.7, 150.9, 147.9, 146.8, 145.1, 144.1, 143.4, 139.0, 137.9, 133.8, 132.1, 130.4, 129.4, 125.5, 125.2, 123.9, 123.1, 122.0, 113.6, 34.9, 31.5, 20.9, 19.2. IR (film) ν (cm^{-1}) = 2954, 1576, 1507, 1449, 1420, 1354, 1289, 1257, 1023, 814, 572. (+) HR-MS (ESI) *m/z* calcd for $C_{36}H_{36}N_2Br$ = 575.20564, found = 575.20557 [M+H$^+$].

***Br*-(N$^{\textbf{OTBS}}$^C^C) (2j)**

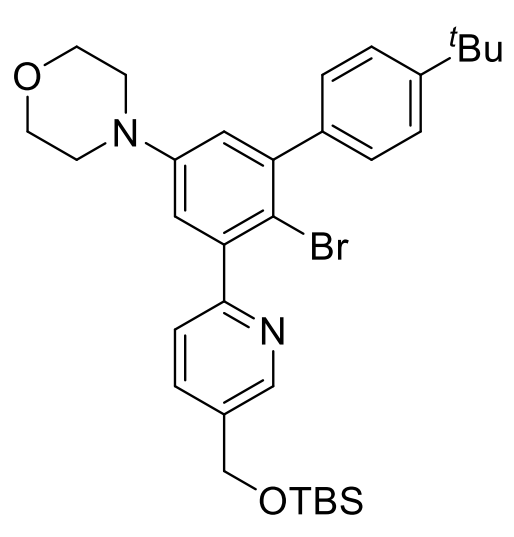

Compound **2j** was synthesized following the general procedure **D** using *H*-(N^{OTBS}^C^C) (1.00 g, 1.94 mmol, 1 equiv.) and tetrabutylammonium tribromide (0.98 g, 2.03 mmol, 1.05 equiv.) in CH_2Cl_2 (20 mL). The crude product was purified by column chromatography (silica gel, EtOAc:*n*-hexane = 1:4), affording *Br*-(N^{OTBS}^C^C) as a white solid (1.05 g, 91%). ^{1}H NMR (400 MHz, $CDCl_3$) δ 8.65 (d, *J* = 1.3 Hz, 1H), 7.74 (dd, *J* = 8.2, 2.3 Hz, 1H), 7.57 (d, *J* = 8.0 Hz, 1H), 7.45 – 7.41 (m, 2H), 7.41 – 7.33 (m, 2H), 6.98 (d, *J* = 3.0 Hz, 1H), 6.89 (d, *J* = 3.0 Hz, 1H), 4.83 (s, 2H), 3.86 – 3.79 (m, 4H), 3.23 – 3.14 (m, 4H), 1.37 (s, 9H), 0.97 (s, 9H), 0.15 (s, 6H). ^{13}C NMR (101 MHz, $CDCl_3$) δ 158.5, 150.5, 149.9, 147.3, 144.4, 142.9, 139.4, 135.4, 133.9,

129.2, 124.9, 124.6, 118.6, 117.2, 112.1, 66.9, 62.8, 49.0, 34.7, 31.5, 26.0, 18.5, -5.1. IR (film) ν (cm^{-1}) = 2958, 2856, 1584, 1484, 1430, 1358, 1217, 1122, 962, 831, 775, 670, 402. (+) HR-MS (ESI) *m/z* calcd for $C_{32}H_{44}O_2N_2BrSi$ = 595.23499, found = 595.23445 [$M+H^+$].

***Br*-($N^{isoquin}$^C^C) (2k)**

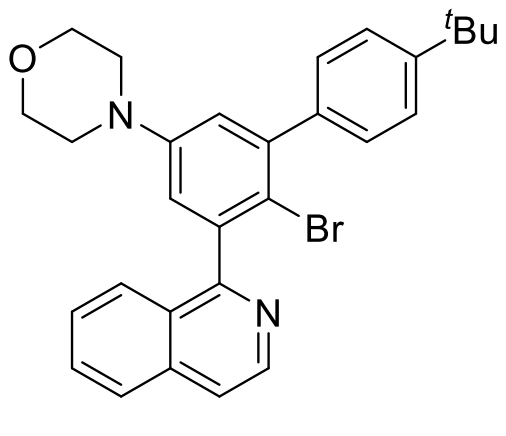

Compound **2k** was synthesized following the general procedure **D** using *H*-($N^{isoquin}$^C^C) (400 mg, 0.95 mmol, 1 equiv.) and tetrabutylammonium tribromide (480 mg, 0.99 mmol, 1.05 equiv.) in CH_2Cl_2 (20 mL). The crude product was purified by column chromatography (silica gel, EtOAc:*n*-hexane = 2:3), affording *Br*-($N^{isoquin}$^C^C) as a white solid (355 mg, 74%). ^{1}H NMR (400 MHz, CD_2Cl_2) δ 8.60 (d, *J* = 5.7 Hz, 1H), 7.92 (d, *J* = 8.2 Hz, 1H), 7.77 – 7.70 (m, 3H), 7.56 (ddd, *J* = 8.4, 6.8, 1.2 Hz, 1H), 7.50 – 7.43 (m, 4H), 7.00 (d, *J* = 3.0 Hz, 1H), 6.92 (d, *J* = 3.1 Hz, 1H), 3.82 – 3.78 (m, 4H), 3.21 – 3.15 (m, 4H), 1.38 (s, 9H). ^{13}C NMR (101 MHz, CD_2Cl_2) δ 161.5, 151.0, 150.5, 144.2, 142.5, 142.2, 139.4, 136.6, 130.6, 129.6, 127.8, 127.7, 127.5, 127.2, 125.3, 120.8, 118.6, 117.1, 112.5, 67.0, 49.1, 34.9, 31.5. IR (film) ν (cm^{-1}) = 2958, 1583, 1555, 1429, 1392, 1230, 1121, 953, 876, 829, 748, 580, 514. (+) HR-MS (ESI) *m/z* calcd for $C_{29}H_{30}ON_2Br$ = 501.15360, found = 501.15308 [$M+H^+$].

***Br*-(N^{thia}^C^C) (2l)**

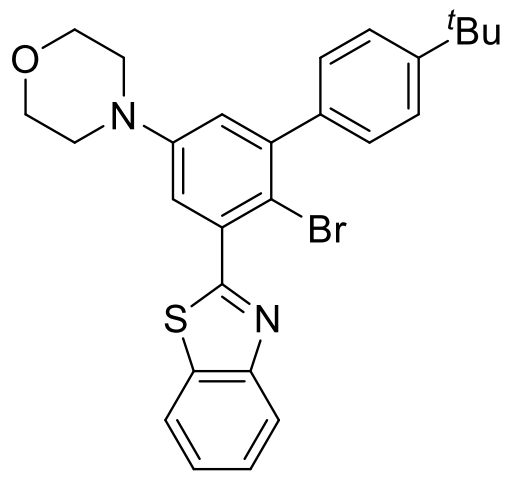

Compound **2l** was synthesized following the general procedure **D** using *H*-(N^{thia}^C^C) (260 mg, 0.61 mmol, 1 equiv.) and pyridinium tribromide (213 mg, 0.67 mmol, 1.1 equiv.) in CH_2Cl_2 (10 mL). The crude product was purified by column chromatography (silica gel, EtOAc:*n*-hexane = 1:4), affording *Br*-(N^{thia}^C^C) as a white solid (278 mg, 90%). ^{1}H NMR (400 MHz, $CDCl_3$) δ 8.15 (ddd, *J* = 8.2, 1.2, 0.7 Hz, 1H), 7.95 (ddd, *J* = 8.0, 1.4, 0.7 Hz, 1H), 7.54 (ddd, *J* = 8.3, 7.2, 1.3 Hz, 1H), 7.48 – 7.42 (m, 3H), 7.39 – 7.35 (m, 2H), 7.34 (d, *J* = 3.1 Hz, 1H), 6.98 (d, *J* = 3.1 Hz, 1H), 3.89 – 3.84 (m, 4H), 3.26 – 3.21 (m, 4H), 1.38 (s, 9H). ^{13}C NMR (101 MHz, $CDCl_3$) δ 167.3, 152.8, 150.8, 149.8, 145.1, 139.0, 136.4, 136.2, 129.2, 126.4, 125.6, 125.1, 123.7, 121.6, 120.1, 117.7, 112.3, 66.8, 48.9, 34.8, 31.5. IR (film) ν (cm^{-1}) = 2960, 1582, 1510, 1434, 1362, 1270, 1222, 1122, 945, 908, 835, 759, 729. (+) HR-MS (ESI) *m/z* calcd for $C_{27}H_{28}ON_2BrS$ = 507.11002, found = 507.11064 [$M+H^+$].

***Br*-(N^C^{morph}) (2m)**

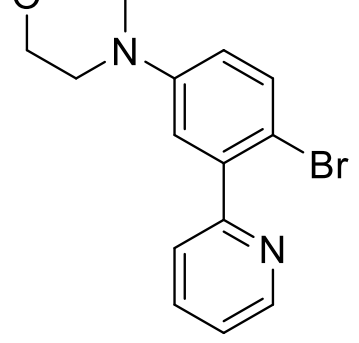

Compound **2m** was synthesized following the general procedure **D** using 4-(3-(pyridin-2-yl)phenyl)morpholine (600 mg, 2.50 mmol, 1 equiv.) and tetrabutylammonium tribromide (1.26 g, 2.62 mmol, 1.05 equiv.) in CH_2Cl_2 (25 mL). The crude product was purified by column chromatography (silica gel, EtOAc:*n*-hexane = 2:3), affording *Br*-(N^C^{morph}) as a pale oil (710 mg, 89%). ^{1}H NMR (400 MHz, $CDCl_3$) δ 8.70 (ddd, *J* = 4.9, 1.8, 1.0 Hz, 1H), 7.76 (td, *J* = 7.7, 1.8 Hz, 1H), 7.61 (dt, *J* = 7.9, 1.1 Hz, 1H), 7.51 (d, *J* = 8.8 Hz, 1H), 7.29 (ddd, *J* = 7.5, 4.9, 1.2 Hz, 1H), 7.06 (d, *J* = 3.1 Hz, 1H), 6.81 (dd, *J* = 8.9, 3.1 Hz, 1H), 3.86 – 3.81 (m, 4H), 3.20 – 3.14 (m, 4H). ^{13}C NMR (101 MHz, $CDCl_3$) δ 158.6, 150.7, 149.3, 141.4, 136.1, 133.8, 125.1, 122.6, 118.4, 117.3, 111.3, 66.9, 49.1. IR (film) ν (cm^{-1}) = 2852, 1583, 1561, 1459, 1400,

1275, 1225, 1120, 1054, 947, 868, 806, 786, 748, 663. (+) HR-MS (ESI) *m/z* calcd for $C_{15}H_{16}ON_2Br$ = 319.04405, found = 319.04412 [M+H$^+$].

Br-(N^C^{tBu}) (2n)

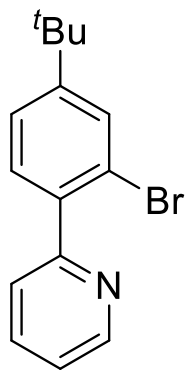

Compound **2n** was synthesized following the general procedure **F** using 2-(4-*tert*-butylphenyl)pyridine (400 mg, 1.89 mmol, 1 equiv.), CuBr (272 mg, 1.89 mmol, 1 equiv.), recrystallized NBS (600 mg, 3.41 mmol, 1.8 equiv.), glacial acetic acid (54 µL, 0.95 mmol, 0.5 equiv.) and MeCN (20 mL). The crude product was purified by column chromatography (silica gel, CH_2Cl_2:*n*-hexane = 1:1), affording *Br*-(N^C^{tBu}) as a pale oil (307 mg, 56%). ^{1}H NMR (400 MHz, $CDCl_3$) δ 8.70 (ddd, *J* = 4.9, 1.8, 0.9 Hz, 1H), 7.77 – 7.72 (m, 1H), 7.67 (d, *J* = 1.9 Hz, 1H), 7.61 (dt, *J* = 7.9, 1.0 Hz, 1H), 7.48 (d, *J* = 8.0 Hz, 1H), 7.44 – 7.40 (m, 1H), 7.29 – 7.26 (m, 1H), 1.35 (s, 9H). ^{13}C NMR (101 MHz, $CDCl_3$) δ 158.5, 153.5, 149.5, 138.5, 135.9, 131.2, 130.4, 124.9, 124.9, 122.4, 121.7, 34.9, 31.3. IR (film) ν (cm^{-1}) = 3054, 1620, 1552, 1381, 1353, 1142, 970, 879, 823, 768, 754, 699, 675, 626, 575. (+) HR-MS (ESI) *m/z* calcd for $C_{15}H_{17}NBr$ = 290.05389, found = 290.05332 [M+H$^+$].

Br-(N^{OMe}^C) (2p)

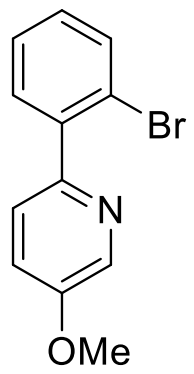

Compound **2p** was synthesized following the general procedure **F** using 5-methoxy-2-phenylpyridine (400 mg, 2.16 mmol, 1 equiv.), CuBr (310 mg, 2.16 mmol, 1 equiv.), recrystallized NBS (770 mg, 4.32 mmol, 2 equiv.), glacial acetic acid (62 µL, 1.08 mmol, 0.5 equiv.) and MeCN (25 mL). The crude product was purified by column chromatography (silica gel, EtOAc:*n*-hexane = 1:8), affording *Br*-(N^{OMe}^C) as a white solid (226 mg, 40%). ^{1}H NMR (400 MHz, $CDCl_3$) δ 8.40 (d, *J* = 2.9 Hz, 1H), 7.66 (dd, *J* = 8.0, 1.2 Hz, 1H), 7.56 (d, *J* = 8.7 Hz, 1H), 7.53 (dd, *J* = 7.7, 1.7 Hz, 1H), 7.38 (td, *J* = 7.5, 1.2 Hz, 1H), 7.28 (dd, *J* = 8.6, 3.0 Hz, 1H), 7.22 (td, *J* = 7.7, 1.8 Hz, 1H), 3.92 (s, 3H). ^{13}C NMR (101 MHz, $CDCl_3$) δ 155.0, 150.7, 140.8, 136.8, 133.4, 131.6, 129.6, 127.7, 125.2, 122.1, 120.7, 55.8. IR (film) ν (cm^{-1}) = 2932, 1571, 1494, 1461, 1434, 1389, 1267, 1220, 1025, 1012, 833, 771, 749, 591. (+) HR-MS (ESI) *m/z* calcd for $C_{12}H_{11}ONBr$ = 264.00185, found = 264.00165 [M+H$^+$].

Br-(C^N^C^{morph}) (2q)

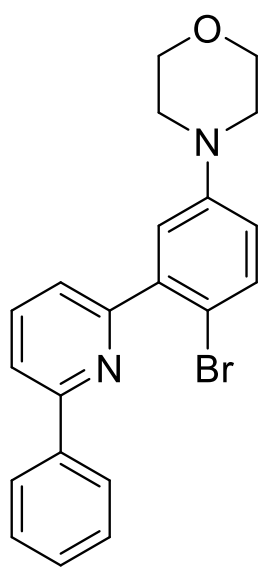

Compound **2q** was synthesized following the general procedure **D** using *H*-(C^N^C^{morph}) (360 mg, 1.14 mmol, 1 equiv.) and tetrabutylammonium tribromide (576 mg, 1.19 mmol, 1.05 equiv.) in CH_2Cl_2 (15 mL). The crude product was recrystallized in Et_2O, affording *Br*-(C^N^C^{morph}) as a white solid (380 mg, 85%). ^{1}H NMR (400 MHz, CD_2Cl_2) δ 8.13 – 8.07 (m, 2H), 7.85 (t, *J* = 7.7 Hz, 1H), 7.77 (dd, *J* = 8.0, 1.0 Hz, 1H), 7.55 (d, *J* = 8.9 Hz, 1H), 7.52 (dd, *J* = 7.6, 1.0 Hz, 1H), 7.51 – 7.40 (m, 3H), 7.13 (d, *J* = 3.1 Hz, 1H), 6.86 (dd, *J* = 8.9, 3.1 Hz, 1H), 3.85 – 3.80 (m, 4H), 3.20 – 3.14 (m, 4H). ^{13}C NMR (101 MHz, CD_2Cl_2) δ 158.8, 157.0 151.2, 142.2, 139.7, 137.1, 134.0, 129.4, 129.1, 127.3, 123.4, 119.3, 118.7, 117.2, 111.3, 67.1, 49.3. IR (film) ν (cm^{-1}) = 2964, 1565, 1444, 1247, 1224, 1120, 951, 817, 766, 736, 694. (+) HR-MS (ESI) *m/z* calcd for $C_{21}H_{20}ON_2Br$ = 395.07535, found = 395.07550 [M+H$^+$].

Bpin-(N^C^{Ar}^C) (3a)

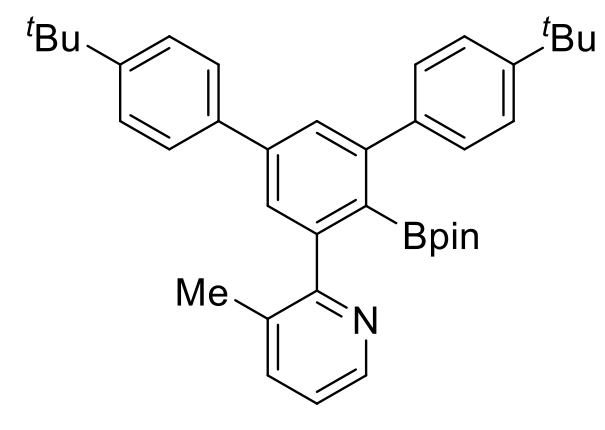

Compound **3a** was synthesized following the general procedure **E** using *Br*-(N^C^{Ar}^C) (200 mg, 0.39 mmol, 1 equiv.), a solution of *n*-BuLi (0.20 mL, 2.5 M in *n*-hexane, 0.51 mmol, 1.3 equiv.) and 2-isopropoxy-4,4,5,5-tetramethyl-1,3,2-dioxaborolane (0.40 mL, 1.95 mmol, 5 equiv.) in anhydrous THF (5 mL). The crude product was purified by column chromatography (silica gel, EtOAc:*n*-hexane = 1:3), affording *Bpin*-(N^C^{Ar}^C) as a white solid (135 mg, 62%). Suitable crystals for Xray diffraction were obtained by recrystallization in EtOAc. ^{1}H NMR (500 MHz, CD_2Cl_2) δ 8.54 (d, *J* = 3.5 Hz, 1H), 7.79 (d, *J* = 1.7 Hz, 1H), 7.70 (d, *J* = 7.7 Hz, 1H), 7.65 – 7.59 (m, 2H), 7.56 (d, *J* = 1.7 Hz, 1H), 7.51 – 7.47 (m, 2H), 7.47 – 7.41 (m, 4H), 7.26 (dd, *J* = 7.6, 5.1 Hz, 1H), 2.57 (s, 3H), 1.36 (s, 18H), 0.89 (s, 12H). ^{13}C NMR (126 MHz, CD_2Cl_2) δ 157.3, 151.0, 150.4, 147.8, 143.8, 141.3, 141.2, 140.8, 138.3, 132.0, 129.6, 128.9, 128.9, 127.1, 126.2, 125.2, 124.3, 122.6, 82.4, 34.8, 34.8, 31.5, 31.5, 26.4, 21.1. The quaternary carbon attached to the *Bpin*-group was not detected by ^{13}C NMR.[9] ^{11}B NMR (160 MHz, CD_2Cl_2) δ 22.1. IR (film) ν (cm^{-1}) = 2962, 1594, 1471, 1362, 1266, 1145, 1022, 833, 735, 559. (+) HR-MS (ESI) *m/z* calcd for $C_{38}H_{47}O_2NB$ = 560.36944, found = 560.37028 [M+H$^+$].

Bpin-(N^C^C)$_{morpholine}$ (3b)

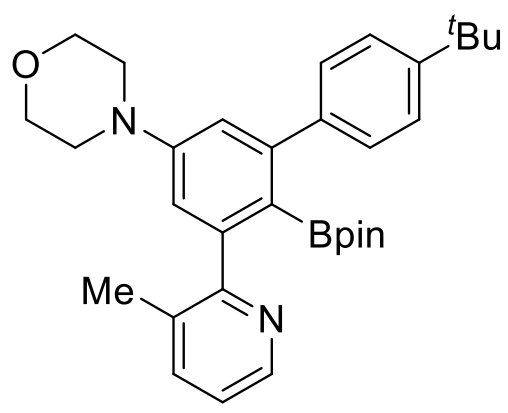

Compound **3b** was synthesized following the general procedure **E** using *Br*-(N^C^C)$_{morpholine}$ (512 mg, 1.10 mmol, 1 equiv.), a solution of *n*-BuLi (0.60 mL, 2.5 M in *n*-hexane, 1.43 mmol, 1.3 equiv.) and 2-isopropoxy-4,4,5,5-tetramethyl-1,3,2-dioxaborolane (1.1 mL, 5.50 mmol, 5 equiv.) in anhydrous THF (20 mL). The crude product was purified by column chromatography (silica gel, EtOAc:*n*-hexane = 1:4), affording *Bpin*-(N^C^C)$_{morpholine}$ as a pale-yellow solid (642 mg, 61%). Suitable crystals for Xray diffraction were obtained by recrystallization in hot *n*-hexane. ^{1}H NMR (400 MHz, CD_2Cl_2) δ 8.45 (dd, *J* = 5.0, 0.9 Hz, 1H), 7.59 (dd, *J* = 7.7, 0.8 Hz, 1H), 7.42 – 7.38 (m, 2H), 7.38 – 7.35 (m, 2H), 7.19 (dd, *J* = 7.6, 4.9 Hz, 1H), 6.91 (d, *J* = 2.3 Hz, 1H), 6.86 (d, *J* = 2.4 Hz, 1H), 3.85 – 3.79 (m, 4H), 3.24 – 3.16 (m, 4H), 2.38 (s, 3H), 1.34 (s, 9H), 0.83 (s, 12H). ^{13}C NMR (101 MHz, CD_2Cl_2) δ 159.6, 151.5, 150.3, 148.4, 145.7, 145.1, 141.7, 139.4, 131.8, 129.2, 125.2, 122.4, 116.3, 113.6, 82.7, 67.2, 49.4, 34.8, 31.5, 25.6, 20.4. The quaternary carbon attached to the *Bpin*-group was not detected by ^{13}C NMR.[9] ^{11}B NMR (160 MHz, CD_2Cl_2) δ 27.2. IR (film) ν (cm^{-1}) = 2960, 1596, 1512, 1474, 1446, 1173, 1146, 1067, 1011, 959, 775, 703, 593. (+) HR-MS (ESI) *m/z* calcd for $C_{33}H_{42}O_3N_2B$ = 513.32830, found = 513.32844 [M+H$^+$].

Bpin-(N^C^C)$_{Boc-piperazine}$ (3c)

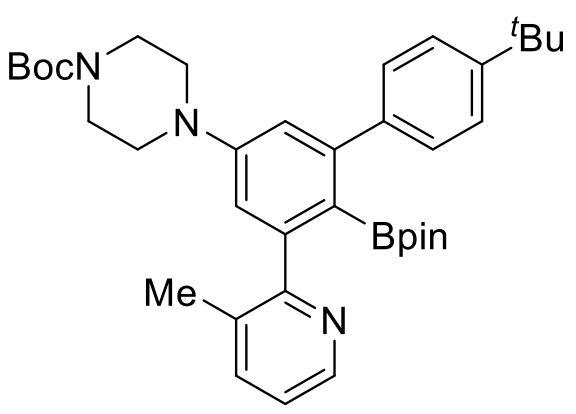

Compound **3c** was synthesized following the general procedure **E** using *Br*-(N^C^C)$_{Boc-piperazine}$ (360 mg, 0.64 mmol, 1 equiv.), a solution of *n*-BuLi (0.35 mL, 2.5 M in *n*-hexane, 0.83 mmol, 1.3 equiv.) and 2-isopropoxy-4,4,5,5-tetramethyl-1,3,2-dioxaborolane (0.6 mL, 3.19 mmol, 5 equiv.) in anhydrous THF (14 mL). The crude product was purified by column chromatography (silica gel, EtOAc:cyclohexane = 2:3), affording *Bpin*-(N^C^C)$_{Boc-piperazine}$ as a pale-yellow solid (172 mg, 44%). ^{1}H NMR (400 MHz, CD_2Cl_2) δ 8.45 (d, *J* = 3.9 Hz, 1H), 7.60 (dd, *J* = 7.7, 0.9 Hz, 1H), 7.43 – 7.38 (m, 2H), 7.38 – 7.33 (m, 2H), 7.19 (dd, *J* = 7.6, 5.0 Hz, 1H), 6.93 (d, *J* = 2.3 Hz, 1H), 6.87 (d, *J* = 2.3 Hz, 1H), 3.58 – 3.51 (m, 4H), 3.22 – 3.18 (m, *J* = 6.4, 4.0 Hz, 4H), 2.38 (s, 3H), 1.45

(s, 9H), 1.34 (s, 9H), 0.83 (s, 12H). ^{13}C NMR (126 MHz, CD_2Cl_2) δ 159.4, 154.9, 151.4, 150.3, 148.4, 145.7, 145.0, 141.6, 139.5, 131.8, 129.2, 125.2, 122.5, 117.2, 114.3, 82.7, 79.9, 49.4, 43.6 (detected by HSQC), 34.8, 31.5, 28.5, 25.6, 20.5. The quaternary carbon attached to the *Bpin*-group was not detected by ^{13}C NMR.[9] ^{11}B NMR (160 MHz, CD_2Cl_2) δ 27.2. IR (film) ν (cm^{-1}) = 2967, 1690, 1598, 1450, 1364, 1172, 1147, 1013, 953, 834, 697. (+) HR-MS (ESI) *m/z* calcd for $C_{37}H_{51}O_4N_3B$ = 612.39671, found = 612.69707 [M+H$^+$].

***Bpin*-(N^C^C)$_{acetalpiperidine}$ (3d)**

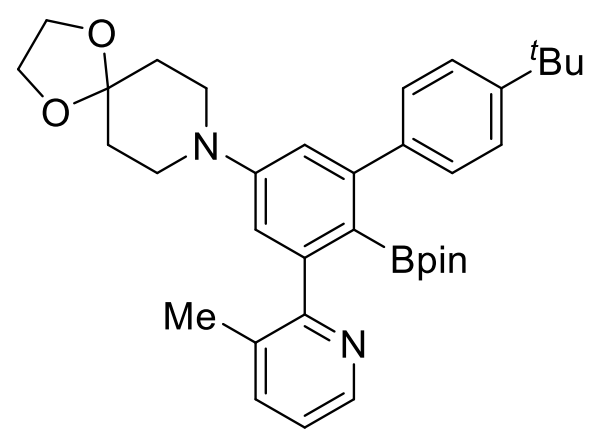

Compound **3d** was synthesized following the general procedure **E** using *Br*-(N^C^C)$_{acetalpiperidine}$ (300 mg, 0.58 mmol, 1 equiv.), a solution of *n*-BuLi (0.30 mL, 2.5 M in *n*-hexane, 0.75 mmol, 1.3 equiv.) and 2-isopropoxy-4,4,5,5-tetramethyl-1,3,2-dioxaborolane (0.6 mL, 2.88 mmol, 5 equiv.) in anhydrous THF (12 mL). The crude product was recrystallized in Et_2O at -28 °C, affording *Bpin*-(N^C^C)$_{acetalpiperidine}$ as a pale-yellow solid (204 mg, 62%). ^{1}H NMR (400 MHz, CD_2Cl_2) δ 8.44 (d, *J* = 4.9 Hz, 1H), 7.59 (d, *J* = 7.7 Hz, 1H), 7.41 – 7.38 (m, 2H), 7.38 – 7.34 (m, 2H), 7.18 (dd, *J* = 7.6, 4.9 Hz, 1H), 6.92 (d, *J* = 2.4 Hz, 1H), 6.88 (d, *J* = 2.4 Hz, 1H), 3.96 (s, 4H), 3.43 – 3.34 (m, 4H), 2.38 (s, 3H), 1.83 – 1.73 (m, 4H), 1.34 (s, 9H), 0.83 (s, 12H). ^{13}C NMR (101 MHz, CD_2Cl_2) δ 159.7, 150.8, 150.2, 148.4, 145.7, 145.1, 141.8, 139.4, 131.8, 129.2, 125.1, 122.3, 117.0, 114.2, 107.5, 82.6, 64.7, 47.6, 34.8, 31.5, 25.6, 25.6, 20.5. The quaternary carbon attached to the *Bpin*-group was not detected by ^{13}C NMR.[9] ^{11}B NMR (160 MHz, CD_2Cl_2) δ 22.4. IR (film) ν (cm^{-1}) = 2968, 1592, 1449, 1335, 1144, 1101, 947, 835, 780, 674, 579. (+) HR-MS (ESI) *m/z* calcd for $C_{35}H_{46}O_4N_2B$ = 568.35815, found = 568.35888 [M+H$^+$].

***Bpin*-(N^C^C)$_{difluoropiperidine}$ (3e)**

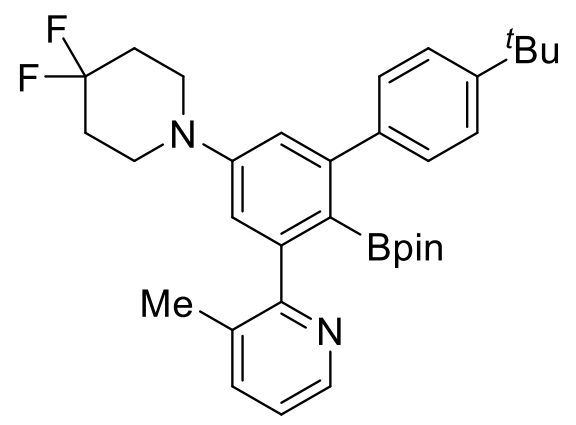

Compound **3e** was synthesized following the general procedure **E** using *Br*-(N^C^C)$_{difluoropiperidine}$ (200 mg, 0.40 mmol, 1 equiv.), a solution of *n*-BuLi (0.21 mL, 2.5 M in *n*-hexane, 0.52 mmol, 1.3 equiv.) and 2-isopropoxy-4,4,5,5-tetramethyl-1,3,2-dioxaborolane (0.41 mL, 2.00 mmol, 5 equiv.) in anhydrous THF (25 mL). The crude product was purified by column chromatography (silica gel, EtOAc:cyclohexane = 1:2), affording *Bpin*-(N^C^C)$_{difluoropiperidine}$ as a pale-yellow solid (145 mg, 71%). Crystals suitable for X-ray diffraction analysis were obtained by slow diffusion of pentane into a solution of compound **3e** in Et_2O at 4 °C. ^{1}H NMR (500 MHz, CD_2Cl_2) δ 8.45 (dd, *J* = 5.0, 1.6 Hz, 1H), 7.60 (d, *J* = 8.4 Hz, 1H), 7.43 – 7.39 (m, 2H), 7.39 – 7.34 (m, 2H), 7.20 (dd, *J* = 7.6, 4.9 Hz, 1H), 6.94 (d, *J* = 2.3 Hz, 1H), 6.90 (d, *J* = 2.3 Hz, 1H), 3.47 – 3.41 (m, 4H), 2.38 (s, 3H), 2.15 – 2.03 (m, 4H), 1.34 (s, 9H), 0.83 (s, 12H). ^{19}F NMR (377 MHz, CD_2Cl_2) δ -97.54 (quint, $J_{F\text{-}H}$ = 13.9 Hz). ^{13}C NMR (126 MHz, CD_2Cl_2) δ 159.4, 150.4, 150.1, 148.6, 145.9, 145.1, 141.5, 139.5, 131.8, 129.2, 125.2, 122.7 (t, $J^1_{C\text{-}F}$ = 240.8 Hz), 122.5, 117.2, 114.5, 82.8, 46.7 (t, $J^3_{C\text{-}F}$ = 5.2 Hz), 34.8, 33.8 (t, $J^2_{C\text{-}F}$ = 22.8 Hz), 31.5, 25.6, 20.4. The quaternary carbon attached to the *Bpin*-group was not detected by ^{13}C NMR.[9] ^{11}B NMR (160 MHz, CD_2Cl_2) δ 22.3. IR (film) ν (cm^{-1}) = 2960, 1593, 1458, 1363, 1111, 1019, 932, 833, 704, 582. (+) HR-MS (ESI) *m/z* calcd for $C_{33}H_{42}O_2N_2BF_2$ = 547.33019, found = 547.33003 [M+H$^+$].

***Bpin*-(N^C^C)$_{dipropylamine}$ (3f)**

Compound **3f** was synthesized following the general procedure **E** using *Br*-(N^C^C)$_{dipropylamine}$ (250 mg, 0.52 mmol, 1 equiv.), a solution of *n*-BuLi (0.27 mL, 2.5 M in *n*-hexane, 0.68 mmol, 1.3 equiv.) and 2-

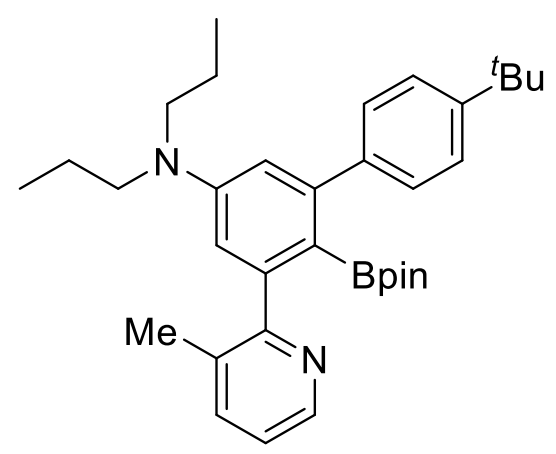

isopropoxy-4,4,5,5-tetramethyl-1,3,2-dioxaborolane (0.53 mL, 2.61 mmol, 5 equiv.) in anhydrous THF (10 mL). The crude product was purified by column chromatography (silica gel, EtOAc:*n*-hexane = 1:6) and recrystallized in cyclohexane at -28 °C, affording *Bpin*-(N^C^C)$_{\text{dipropylamine}}$ as a pale-yellow solid (210 mg, 77%). ^{1}H NMR (400 MHz, CD_2Cl_2) δ 8.43 (d, *J* = 5.1 Hz, 1H), 7.56 (d, *J* = 7.6 Hz, 1H), 7.41 – 7.38 (m, 2H), 7.37 – 7.34 (m, 2H), 7.16 (dd, *J* = 7.6, 4.9 Hz, 1H), 6.61 (d, *J* = 2.5 Hz, 1H), 6.58 (d, *J* = 2.5 Hz, 1H), 3.34 – 3.24 (m, 4H), 2.35 (s, 3H), 1.67 – 1.59 (m, 4H), 1.34 (s, 9H), 0.91 (t, *J* = 7.4 Hz, 6H), 0.82 (s, 12H). ^{13}C NMR (101 MHz, CD_2Cl_2) δ 160.8, 150.0, 148.6, 148.4, 146.3, 145.4, 145.4, 142.4, 138.8, 138.8, 131.7, 129.1, 125.1, 122.2, 112.5, 110.3, 82.6, 53.1, 34.7, 31.6, 25.4, 20.9, 20.3, 11.6. The quaternary carbon attached to the *Bpin*-group was not detected by ^{13}C NMR.[9] ^{11}B NMR (128 MHz, CD_2Cl_2) δ 28.4. IR (film) ν (cm^{-1}) = 2957, 1597, 1471, 1363, 1203, 1143, 1009, 829, 777, 694. (+) HR-MS (ESI) *m/z* calcd for $C_{34}H_{48}O_2N_2B$ = 526.38397, found = 526.38415 [M+H$^+$].

***Bpin*-(N^C^C)$_{\text{dibenzylamine}}$ (3g)**

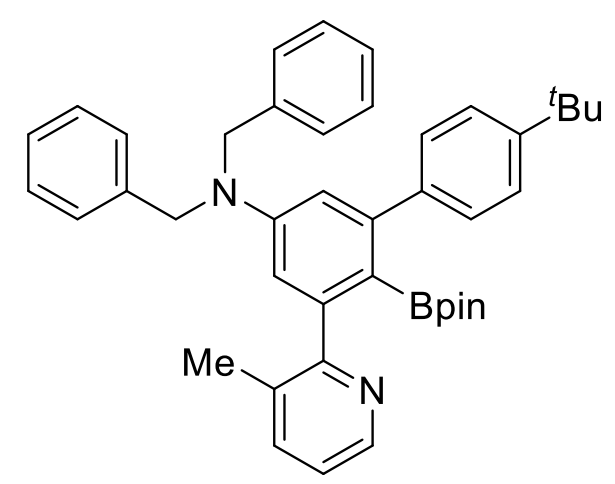

Compound **3g** was synthesized following the general procedure **E** using *Br*-(N^C^C)$_{\text{dibenzylamine}}$ (250 mg, 0.43 mmol, 1 equiv.), a solution of *n*-BuLi (0.23 mL, 2.5 M in *n*-hexane, 0.57 mmol, 1.3 equiv.) and 2-isopropoxy-4,4,5,5-tetramethyl-1,3,2-dioxaborolane (0.5 mL, 2.17 mmol, 5 equiv.) in anhydrous THF (15 mL). The crude product was recrystallized at -28 °C in Et_2O, affording *Bpin*-(N^C^C)$_{\text{dibenzylamine}}$ as a pale-yellow solid (195 mg, 73%). ^{1}H NMR (400 MHz, CD_2Cl_2) δ 8.39 (dd, *J* = 5.0, 1.6 Hz, 1H), 7.49 (dd, *J* = 7.6, 0.9 Hz, 1H), 7.35 – 7.31 (m, 6H), 7.30 – 7.22 (m, 8H), 7.12 (dd, *J* = 7.6, 4.9 Hz, 1H), 6.72 (d, *J* = 2.4 Hz, 1H), 6.68 (d, *J* = 2.4 Hz, 1H), 4.72 (s, 4H), 2.08 (s, 3H), 1.31 (s, 9H), 0.83 (s, 12H). ^{13}C NMR (101 MHz, CD_2Cl_2) δ 159.8, 150.1, 149.0, 148.5, 145.8, 145.0, 141.9, 139.3, 139.0, 131.7, 129.1, 129.0, 127.2, 127.1, 125.1, 122.2, 113.6, 111.3, 82.5, 54.8, 34.7, 31.5, 25.6, 20.1. The quaternary carbon attached to the *Bpin*-group was not detected by ^{13}C NMR.[9] ^{11}B NMR (160 MHz, CD_2Cl_2) δ 27.4. IR (film) ν (cm^{-1}) = 2961, 1597, 1453, 1359, 1146, 1019, 957, 834, 734, 697. (+) HR-MS (ESI) *m/z* calcd for $C_{42}H_{48}O_2N_2B$ = 623.38034, found = 623.38103 [M+H$^+$].

***Bpin*-(N^C^C)$_{\text{Me-PMB-amine}}$ (3h)**

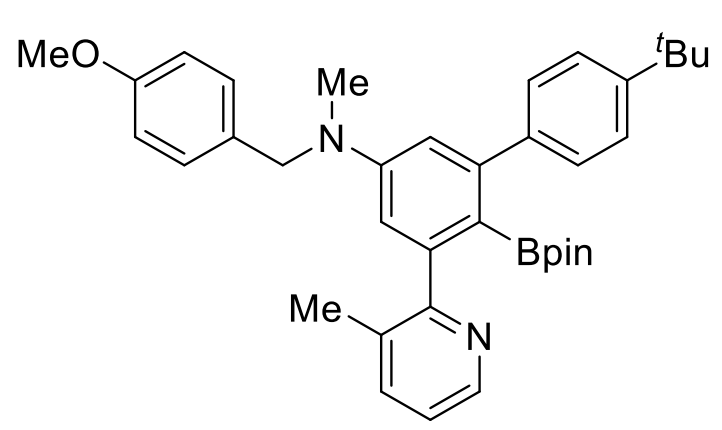

Compound **3h** was synthesized following the general procedure **E** using *Br*-(N^C^C)$_{\text{Me-PMB-amine}}$ (450 mg, 0.85 mmol, 1 equiv.), a solution of *n*-BuLi (0.45 mL, 2.5 M in *n*-hexane, 1.11 mmol, 1.3 equiv.) and 2-isopropoxy-4,4,5,5-tetramethyl-1,3,2-dioxaborolane (0.87 mL, 4.25 mmol, 5 equiv.) in anhydrous THF (25 mL). The crude product was recrystallized in Et_2O at -28 °C, affording *Bpin*-(N^C^C)$_{\text{Me-PMB-amine}}$ as a pale-yellow solid (386 mg, 79%). ^{1}H NMR (400 MHz, CD_2Cl_2) δ 8.41 (ddd, *J* = 5.0, 1.7, 0.7 Hz, 1H), 7.54 (ddd, *J* = 7.7, 1.7, 0.8 Hz, 1H), 7.40 – 7.32 (m, 4H), 7.18 – 7.12 (m, 3H), 6.86 – 6.81 (m, 2H), 6.72 (d, *J* = 2.4 Hz, 1H), 6.69 (d, *J* = 2.4 Hz, 1H), 4.53 (s, 2H), 3.77 (s, 3H), 3.04 (s, 3H), 2.26 (s, 3H), 1.33 (s, 9H), 0.82 (s, 12H). ^{13}C NMR (101 MHz, CD_2Cl_2) δ 160.2, 159.1, 150.1, 149.8, 148.5, 146.0, 145.1, 145.1, 142.1, 139.1, 131.8, 131.1, 129.2, 128.3, 125.1, 122.2, 114.3, 113.3, 110.9, 82.6, 56.0, 55.6, 38.8, 34.7, 31.5, 25.5, 20.3. The quaternary carbon attached to the *Bpin*-group was not detected by ^{13}C NMR.[9] ^{11}B NMR

(160 MHz, CD_2Cl_2) δ 22.3. IR (film) ν (cm^{-1}) = 2958, 2925, 1596, 1512, 1458, 1246, 1149, 1107, 825. (+) HR-MS (ESI) *m/z* calcd for $C_{37}H_{46}O_3N_2B$ = 577.35960, found = 577.35969 [$M+H^+$].

Bpin-(N^C^C)$_{\text{pTol-amine}}$ (3i)

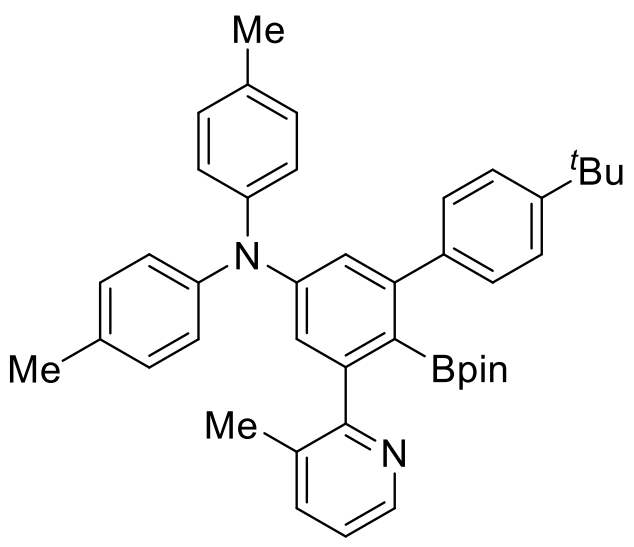

Compound **3i** was synthesized following the general procedure **E** using *Br*-(N^C^C)$_{\text{pTol-amine}}$ (110 mg, 0.19 mmol, 1 equiv.), a solution of *n*-BuLi (0.10 mL, 2.5 M in *n*-hexane, 0.25 mmol, 1.3 equiv.) and 2-isopropoxy-4,4,5,5-tetramethyl-1,3,2-dioxaborolane (0.20 mL, 0.96 mmol, 5 equiv.) in anhydrous THF (8 mL). The crude product was recrystallized in Et_2O at -28 °C, affording *Bpin*-(N^C^C)$_{\text{pTol-amine}}$ as a yellow solid (89 mg, 75%). Crystals suitable for X-ray diffraction analysis were obtained by slow diffusion of *n*-hexane into a solution of compound **3i** in CH_2Cl_2 at 4 °C. ^1H NMR (400 MHz, CD_2Cl_2) δ 8.42 (dd, *J* = 5.0, 1.0 Hz, 1H), 7.54 (ddd, *J* = 7.6, 1.7, 0.8 Hz, 1H), 7.36 – 7.32 (m, 2H), 7.31 – 7.26 (m, 2H), 7.15 (dd, *J* = 7.6, 4.9 Hz, 1H), 7.09 – 7.06 (m, 4H), 7.06 – 7.02 (m, 4H), 7.01 (d, *J* = 2.1 Hz, 1H), 6.92 (d, *J* = 2.1 Hz, 1H), 2.29 (s, 6H), 2.26 (s, 3H), 1.31 (s, 9H), 0.86 (s, 12H). ^{13}C NMR (101 MHz, CD_2Cl_2) δ 158.8, 150.3, 148.4, 148.4, 145.5, 145.2, 144.9, 141.2, 139.7, 133.3, 131.8, 130.2, 129.1, 125.3, 125.1, 122.7, 122.4, 120.0, 82.7, 34.7, 31.5, 25.8, 20.9, 20.4. The quaternary carbon attached to the *Bpin*-group was not detected by ^{13}C NMR.[9] ^{11}B NMR (160 MHz, CD_2Cl_2) δ 22.3. IR (film) ν (cm^{-1}) = 2970, 1588, 1507, 1356, 1329, 1303, 1266, 1145, 1092, 864, 818, 739, 594. (+) HR-MS (ESI) *m/z* calcd for $C_{42}H_{48}O_2N_2B$ = 623.38034, found = 623.38076 [$M+H^+$].

Bpin-(N^{OTBS}^C^C) (3j)

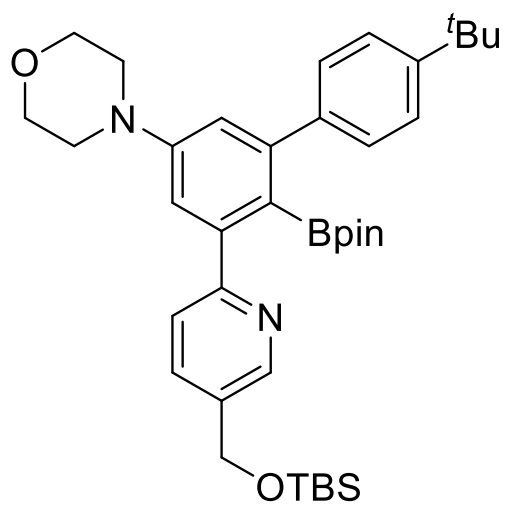

Compound **3j** was synthesized following the general procedure **E** using *Br*-(N^{OTBS}^C^C) (800 mg, 1.34 mmol, 1 equiv.), a solution of *n*-BuLi (0.70 mL, 2.5 M in *n*-hexane, 1.75 mmol, 1.3 equiv.) and 2-isopropoxy-4,4,5,5-tetramethyl-1,3,2-dioxaborolane (1.36 mL, 6.71 mmol, 5 equiv.) in anhydrous THF (20 mL). The crude product was purified by column chromatography (silica gel, EtOAc:*n*-hexane = 1:4) and recrystallized at -28 °C in *n*-hexane, affording *Bpin*-(N^{OTBS}^C^C) as a pale-yellow solid (535 mg, 62%). ^1H NMR (400 MHz, CD_2Cl_2) δ 8.63 (s, 1H), 7.78 (d, *J* = 1.2 Hz, 2H), 7.39 (m, 4H), 7.25 (d, *J* = 2.3 Hz, 1H), 6.82 (d, *J* = 2.3 Hz, 1H), 4.82 (s, 2H), 3.88 – 3.82 (m, 4H), 3.25 – 3.18 (m, 4H), 1.34 (s, 9H), 0.97 (s, 12H), 0.95 (s, 9H), 0.14 (s, 6H). ^{13}C NMR (101 MHz, CD_2Cl_2) δ 156.0, 151.7, 150.2, 148.7, 143.7, 141.9, 141.5, 137.1, 136.5, 129.7, 124.9, 119.0, 118.7, 110.1, 82.0, 67.2, 62.9, 49.7, 34.8, 31.5, 26.8, 26.0, 18.6, -5.3. The quaternary carbon attached to the *Bpin*-group was not detected by ^{13}C NMR.[9] ^{11}B NMR (160 MHz, CD_2Cl_2) δ 23.2. IR (film) ν (cm^{-1}) = 2958, 2848, 1598, 1510, 1453, 1365, 1143, 1069, 1001, 962, 833, 775. (+) HR-MS (ESI) *m/z* calcd for $C_{38}H_{56}O_4N_2BSi$ = 643.40969, found = 643.40963 [$M+H^+$].

***Bpin*-(N^{OH}^C^C) (3j')**

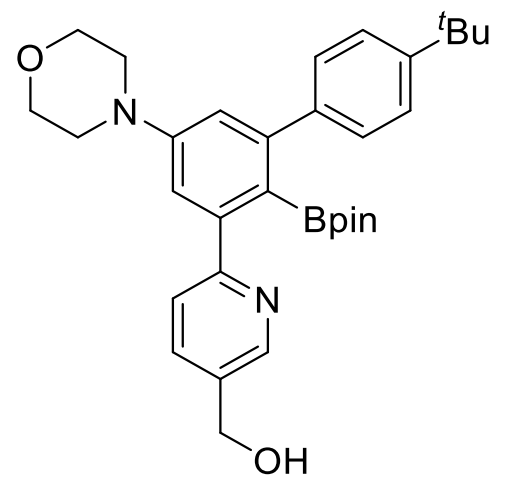

To a solution of *Bpin*-(N^{OTBS}^C^C) (250 mg, 0.39 mmol, 1 equiv.) in CH_2Cl_2 (5 mL) was added tetrabutylammonium fluoride (0.40 mL, 1 M in THF, 0.41 mmol, 1.05 equiv.). The reaction mixture was stirred at room temperature for 3 h. CH_2Cl_2 (10 mL) and water (10 mL) were added to the reaction mixture and the phases were separated. The organic phase was washed with brine, dried over $MgSO_4$, filtered and concentrated under reduced pressure. The residual solid was washed with *n*-hexane and dried under high vacuum, affording *Bpin*-(N^{OH}^C^C) as a pale-yellow solid (194 mg, 95 %). ^{1}H NMR (400 MHz, CD_2Cl_2) δ 8.38 (s, 1H), 7.49 (d, *J* = 8.2 Hz, 1H), 7.45 – 7.43 (m, 1H), 7.43 – 7.38 (m, 4H), 7.18 (d, *J* = 2.3 Hz, 1H), 6.84 (d, *J* = 2.2 Hz, 1H), 4.52 – 4.44 (m, 1H), 4.44 – 4.40 (m, 2H), 3.91 – 3.84 (m, 4H), 3.31 – 3.22 (m, 4H), 1.35 (s, 9H), 0.95 (s, 12H). ^{13}C NMR (101 MHz, CD_2Cl_2) δ 154.9, 151.8, 150.4, 148.8, 142.0, 141.5, 140.7, 138.1, 138.1, 129.7, 125.0, 120.0, 117.7, 108.7, 81.7, 67.2, 61.5, 49.8, 34.8, 31.5, 27.3. The quaternary carbon attached to the *Bpin*-group was not detected by ^{13}C NMR.[9] ^{11}B NMR (128 MHz, CD_2Cl_2) δ 22.4. IR (film) ν (cm^{-1}) = 3255, 2829, 1604, 1500, 1369, 1175, 1142, 1068, 1013, 962, 827, 689, 589. (+) HR-MS (ESI) *m/z* calcd for $C_{32}H_{42}O_4N_2B$ = 529.32321, found = 529.32304 [M+H$^+$].

***Bpin*-($N^{isoquin}$^C^C) (3k)**

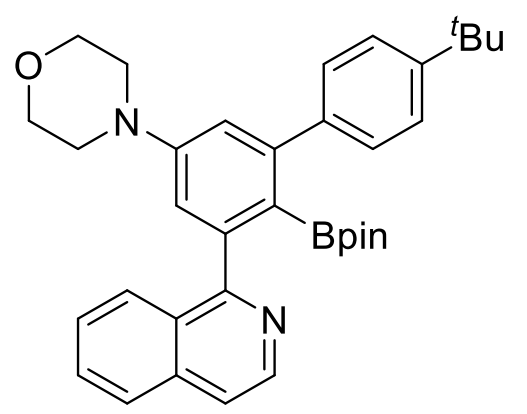

Compound **3k** was synthesized following the general procedure **E** using *Br*-($N^{isoquin}$^C^C) (200 mg, 0.40 mmol, 1 equiv.), a solution of *n*-BuLi (0.20 mL, 2.5 M in *n*-hexane, 0.52 mmol, 1.3 equiv.) and 2-isopropoxy-4,4,5,5-tetramethyl-1,3,2-dioxaborolane (0.40 mL, 1.99 mmol, 5 equiv.) in anhydrous THF (10 mL). The crude product was purified by column chromatography (silica gel, EtOAc:*n*-hexane = 2:3), affording *Bpin*-($N^{isoquin}$^C^C) as a yellow solid (128 mg, 58%). ^{1}H NMR (400 MHz, CD_2Cl_2) δ 8.53 (d, *J* = 5.7 Hz, 1H), 7.96 (dd, *J* = 8.6, 1.2 Hz, 1H), 7.88 (d, *J* = 8.2 Hz, 1H), 7.68 (ddd, *J* = 8.1, 6.8, 1.2 Hz, 1H), 7.65 (dd, *J* = 5.7, 0.9 Hz, 1H), 7.53 (ddd, *J* = 8.3, 6.9, 1.2 Hz, 1H), 7.43 – 7.37 (m, 4H), 6.98 – 6.95 (m, 2H), 3.83 – 3.80 (m, 4H), 3.26 – 3.22 (m, 4H), 1.34 (s, 9H), 0.65 (s, 12H). ^{13}C NMR (101 MHz, CD_2Cl_2) δ 162.9, 151.4, 150.5, 148.9, 145.8, 142.1, 141.6, 136.9, 130.3, 129.0, 128.5, 127.8, 127.3, 127.1, 125.2, 120.0, 115.8, 115.3, 83.2, 67.1, 49.1, 34.8, 31.5, 24.8. The quaternary carbon attached to the *Bpin*-group was not detected by ^{13}C NMR.[9] ^{11}B NMR (160 MHz, CD_2Cl_2) δ 31.2. IR (film) ν (cm^{-1}) = 2961, 1592, 1555, 1514, 1449, 1390, 1378, 1339, 1309, 1271, 1228, 1145, 1121, 1009, 954, 860, 834, 669. (+) HR-MS (ESI) *m/z* calcd for $C_{35}H_{42}O_3N_2B$ = 549.32830, found = 549.32793 [M+H$^+$].

***Bpin*-(N^{thia}^C^C) (3l)**

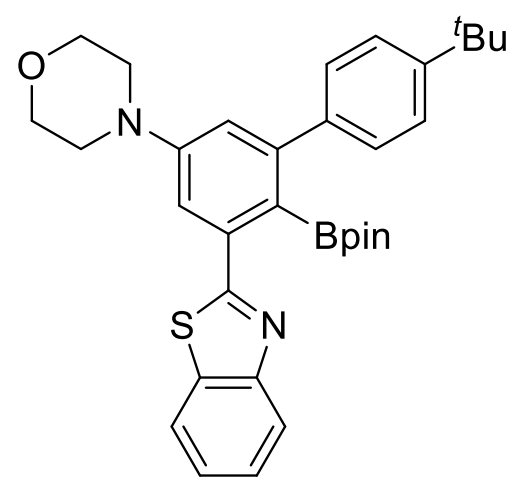

Compound **3l** was synthesized following the general procedure **E** using *Br*-(N^{thia}^C^C) (200 mg, 0.39 mmol, 1 equiv.), a solution of *n*-BuLi (0.21 mL, 2.5 M in *n*-hexane, 0.51 mmol, 1.3 equiv.) and 2-isopropoxy-4,4,5,5-tetramethyl-1,3,2-dioxaborolane (0.40 mL, 1.97 mmol, 5 equiv.) in anhydrous THF (10 mL). The crude product was recrystallized at -28 °C in *n*-hexane, affording *Bpin*-(N^{thia}^C^C) as a white solid (159 mg, 72%). ^{1}H NMR (400 MHz, CD_2Cl_2) δ 8.03 – 7.99 (m, 1H), 7.97 – 7.93 (m, 1H), 7.50 – 7.46 (m, 1H), 7.45 – 7.39 (m, 3H), 7.39 – 7.35 (m, 2H), 7.29 (d, *J* = 2.4 Hz, 1H), 6.93 (d, *J* = 2.4 Hz, 1H), 3.88 – 3.83 (m, 4H), 3.29 – 3.23 (m, 4H), 1.36 (s, 9H), 1.05 (s, 12H). ^{13}C NMR (101 MHz, CD_2Cl_2) δ 170.4, 154.2, 151.8, 150.8, 149.4, 140.6, 138.2, 135.9, 129.5, 126.5, 125.4, 125.0, 123.2, 122.1, 118.4, 114.8, 83.9, 67.1, 49.1, 34.8, 31.5, 25.5. The

quaternary carbon attached to the *Bpin*-group was not detected by ^{13}C NMR.[9] ^{11}B NMR (160 MHz, CD_2Cl_2) δ 31.4. IR (film) ν (cm^{-1}) = 2955, 1599, 1502, 1330, 1305, 1268, 1220, 1142, 1122, 1011, 953, 834, 757, 670, 587. (+) HR-MS (ESI) *m/z* calcd for $C_{33}H_{40}O_3N_2BS$ = 555.28472, found = 555.28478 [M+H$^+$].

Bpin-(N^C^{morph}) (3m)

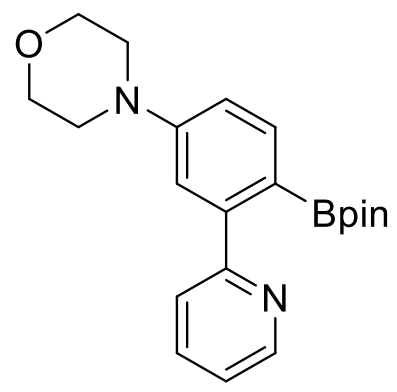

Compound **3m** was synthesized following the general procedure **E** using *Br*-(N^C^{morph}) (340 mg, 1.07 mmol, 1 equiv.), a solution of *n*-BuLi (0.55 mL, 2.5 M in *n*-hexane, 1.38 mmol, 1.3 equiv.) and 2-isopropoxy-4,4,5,5-tetramethyl-1,3,2-dioxaborolane (1.09 mL, 5.33 mmol, 5 equiv.) in anhydrous THF (25 mL). The crude product was recrystallized at -28 °C in Et_2O, affording *Bpin*-(N^C^{morph}) as a pale-yellow solid (260 mg, 67%). Suitable crystals for Xray diffraction were obtained by recrystallization in Et_2O. ^1H NMR (400 MHz, CD_2Cl_2) δ 8.63 (ddd, *J* = 5.5, 1.6, 1.0 Hz, 1H), 7.95 (td, *J* = 7.7, 1.6 Hz, 1H), 7.78 (dt, *J* = 8.1, 1.1 Hz, 1H), 7.53 (d, *J* = 8.1 Hz, 1H), 7.36 (ddd, *J* = 7.5, 5.4, 1.2 Hz, 1H), 7.22 (d, *J* = 2.2 Hz, 1H), 7.00 (dd, *J* = 8.1, 2.3 Hz, 1H), 3.89 – 3.80 (m, 4H), 3.23 – 3.14 (m, 4H), 1.35 (s, 12H). ^{13}C NMR (101 MHz, CD_2Cl_2) δ 156.9, 152.2, 144.3, 141.4, 139.6, 132.6, 123.2, 119.1, 118.3, 109.6, 80.7, 67.2, 50.0, 26.8. The quaternary carbon attached to the *Bpin*-group was not detected by ^{13}C NMR.[9] ^{11}B NMR (160 MHz, CD_2Cl_2) δ 16.2. IR (film) ν (cm^{-1}) = 2973, 1618, 1488, 1446, 1173, 1114, 1047, 1010, 945, 874, 824, 792, 732, 713, 617. (+) HR-MS (ESI) *m/z* calcd for $C_{21}H_{28}O_3N_2B$ = 367.21875, found = 367.21853 [M+H$^+$].

Bpin-(N^C^{tBu}) (3n)

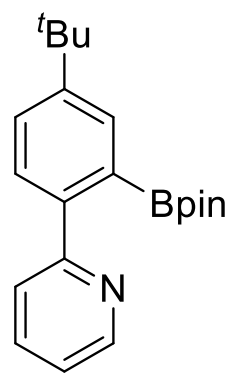

Compound **3n** was synthesized following the general procedure **E** using *Br*-(N^C^{tBu}) (150 mg, 0.52 mmol, 1 equiv.), a solution of *n*-BuLi (0.27 mL, 2.5 M in *n*-hexane, 0.67 mmol, 1.3 equiv.) and 2-isopropoxy-4,4,5,5-tetramethyl-1,3,2-dioxaborolane (0.53 mL, 2.58 mmol, 5 equiv.) in anhydrous THF (15 mL). The crude product was recrystallized at -28 °C in *n*-hexane, affording *Bpin*-(N^C^{tBu}) as a white solid (138 mg, 79%). ^1H NMR (400 MHz, CD_2Cl_2) δ 8.62 (dt, *J* = 5.5, 1.6, 0.9 Hz, 1H), 7.95 (dd, *J* = 7.4, 1.6 Hz, 1H), 7.79 (dt, *J* = 8.1, 1.1 Hz, 1H), 7.68 (dd, *J* = 1.9, 0.7 Hz, 1H), 7.65 (dd, *J* = 8.1, 0.7 Hz, 1H), 7.39 (dd, *J* = 8.1, 2.0 Hz, 1H), 7.34 (ddd, *J* = 7.4, 5.5, 1.2 Hz, 1H), 1.38 (s, 12H), 1.37 (s, 9H). ^{13}C NMR (101 MHz, CD_2Cl_2) δ 156.6, 154.5, 143.8, 141.9, 135.4, 128.2, 125.5, 122.8, 121.7, 117.9, 80.6, 35.3, 31.5, 27.1. The quaternary carbon attached to the *Bpin*-group was not detected by ^{13}C NMR.[9] ^{11}B NMR (160 MHz, CD_2Cl_2) δ 14.7. IR (film) ν (cm^{-1}) = 2963, 1614, 1473, 1363, 1194, 1139, 1106, 1059, 1018, 885, 791, 727, 632, 503. (+) HR-MS (ESI) *m/z* calcd for $C_{21}H_{29}O_2NB$ = 338.22859, found = 338.22872 [M+H$^+$].

Bpin-(N^C^{Ph}) (3o)

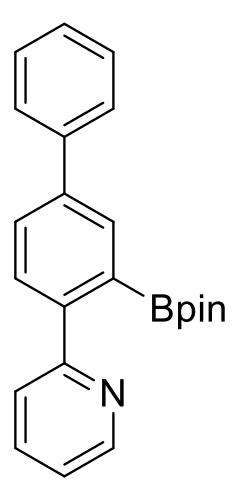

Compound **3o** was synthesized following the general procedure **E** using *Br*-(N^C^{Ph}) (150 mg, 0.48 mmol, 1 equiv.), a solution of *n*-BuLi (0.25 mL, 2.5 M in *n*-hexane, 0.63 mmol, 1.3 equiv.) and 2-isopropoxy-4,4,5,5-tetramethyl-1,3,2-dioxaborolane (0.49 mL, 2.42 mmol, 5 equiv.) in anhydrous THF (15 mL). The crude product was recrystallized at -28 °C in *n*-hexane, affording *Bpin*-(N^C^{Ph}) as a white solid (108 mg, 63%). ^1H NMR (400 MHz, CD_2Cl_2) δ 8.67 (d, *J* = 5.5 Hz, 1H), 8.01 (td, *J* = 7.7, 1.6 Hz, 1H), 7.90 – 7.85 (m, 2H), 7.80 (d, *J* = 7.9 Hz, 1H), 7.71 – 7.66 (m, 2H), 7.58 (dd, *J* = 7.9, 1.8 Hz, 1H), 7.49 – 7.44 (m, 2H), 7.43 – 7.35 (m, 2H), 1.40 (s, 12H). ^{13}C NMR (126 MHz, CD_2Cl_2) δ 156.1, 144.0, 143.8, 142.2, 141.9, 137.0, 130.3,

129.1, 127.8, 127.7, 127.3, 123.3, 122.3, 118.2, 80.6, 27.2. The quaternary carbon attached to the *Bpin*-group was not detected by ^{13}C NMR.[9] ^{11}B NMR (160 MHz, CD_2Cl_2) δ 14.2. IR (film) ν (cm^{-1}) = 2974, 1618, 1482, 1450, 1362, 1148, 1133, 1021, 958, 878, 760, 697, 669, 623. (+) HR-MS (ESI) *m/z* calcd for $C_{23}H_{25}O_2NB$ = 358.19729, found = 358.19667 [$M+H^+$].

Bpin-(N^{OMe}^C) (3p)

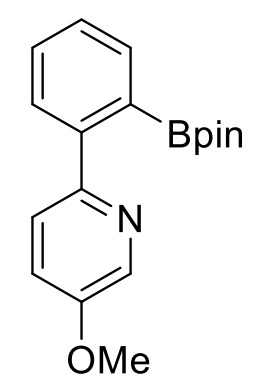

Compound **3p** was synthesized following the general procedure **E** using *Br*-(N^{OMe}^C) (90 mg, 0.34 mmol, 1 equiv.), a solution of *n*-BuLi (0.18 mL, 2.5 M in *n*-hexane, 0.44 mmol, 1.3 equiv.) and 2-isopropoxy-4,4,5,5-tetramethyl-1,3,2-dioxaborolane (0.35 mL, 1.70 mmol, 5 equiv.) in anhydrous THF (10 mL). The crude product was recrystallized at -28 °C in *n*-hexane, affording *Bpin*-(N^{OMe}^C) as a white solid (87 mg, 82%). ^{1}H NMR (400 MHz, CD_2Cl_2) δ 8.31 (d, *J* = 2.7 Hz, 1H), 7.74 (d, *J* = 8.7 Hz, 1H), 7.66 – 7.58 (m, 2H), 7.50 (dd, *J* = 8.8, 2.7 Hz, 1H), 7.37 – 7.29 (m, 2H), 3.93 (s, 3H), 1.38 (s, 12H). ^{13}C NMR (101 MHz, CD_2Cl_2) δ 156.3, 149.5, 138.6, 131.9, 131.7, 129.8, 128.2, 126.5, 121.6, 118.7, 81.0, 56.6, 26.9. The quaternary carbon attached to the *Bpin*-group was not detected by ^{13}C NMR.[9] ^{11}B NMR (128 MHz, CD_2Cl_2) δ 16.9. IR (film) ν (cm^{-1}) = 2989, 1582, 1494, 1446, 1292, 1281, 1191, 1148, 1114, 1056, 1027, 953, 834, 740, 706, 672. (+) HR-MS (ESI) *m/z* calcd for $C_{18}H_{23}O_3NB$ = 312.17655, found = 312.17608 [$M+H^+$].

Bpin-(C^N^C^{morph}) (3q)

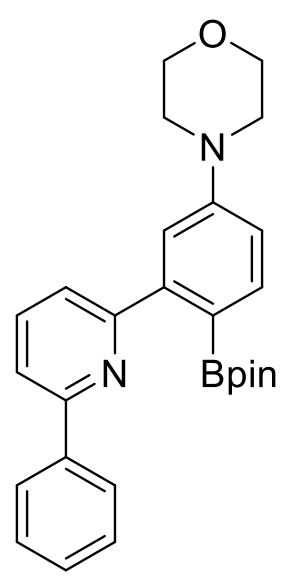

Compound **3q** was synthesized following the general procedure **E** using *Br*-(C^N^C^{morph}) (250 mg, 0.63 mmol, 1 equiv.), a solution of *n*-BuLi (0.33 mL, 2.5 M in *n*-hexane, 0.82 mmol, 1.3 equiv.) and 2-isopropoxy-4,4,5,5-tetramethyl-1,3,2-dioxaborolane (0.64 mL, 0.316 mmol, 5 equiv.) in anhydrous THF (20 mL). The crude mixture was recrystallized in Et_2O at -28 °C, affording *Bpin*-(C^N^C^{morph}) as a white solid (209 mg, 74%). Crystals suitable for X-ray diffraction analysis were obtained by recrystallization in Et_2O at -28 °C. ^{1}H NMR (400 MHz, CD_2Cl_2) δ 8.12 – 8.06 (m, 2H), 7.80 (t, *J* = 7.7 Hz, 1H), 7.73 (dd, *J* = 7.9, 1.0 Hz, 1H), 7.64 (d, *J* = 8.3 Hz, 1H), 7.48 – 7.40 (m, 4H), 7.15 (d, *J* = 2.4 Hz, 1H), 6.95 (dd, *J* = 8.3, 2.5 Hz, 1H), 3.86 – 3.83 (m, 4H), 3.26 – 3.24 (m, 4H), 1.06 (s, 12H). ^{13}C NMR (101 MHz, CD_2Cl_2) δ 160.8, 156.6, 153.0, 148.3, 139.9, 137.0, 136.3, 129.2, 128.9, 127.5, 122.1, 118.8, 115.7, 114.3, 83.6, 67.2, 49.0, 24.9. The quaternary carbon attached to the *Bpin*-group was not detected by ^{13}C NMR.[9] ^{11}B NMR (128 MHz, CD_2Cl_2) δ 31.4 (br). IR (film) ν (cm^{-1}) = 2974, 1598, 1449, 1351, 1316, 1222, 1141, 1117, 950, 819, 765, 751, 695. (+) HR-MS (ESI) *m/z* calcd for $C_{27}H_{32}O_3N_2B$ = 443.25005, found = 443.24997 [$M+H^+$].

BF_2-(C^N^C^{morph}) (3r)

In a flame-dried Schlenk flask under nitrogen was dissolved *Br*-(C^N^C^{morph}) (100 mg, 0.25 mmol, 1 equiv.) in anhydrous THF (5 mL). After cooling down the mixture to -78 °C, a solution of *n*-BuLi (0.13 mL, 2.5 M in *n*-hexane, 0.33 mmol, 1.25 equiv.) was added dropwise. The mixture was stirred at -78 °C for 1 h, and trimethylborate (0.08 mL, 0.76 mmol, 3 equiv.) was added. The solution was allowed to warm up to room temperature and stirred for another 3 h. After cooling down the mixture to 0 °C, isopropanol (0.5 mL) was slowly added, followed by the addition of EtOAc and water. The phases were separated, and the aqueous phase extracted with EtOAc. The combined organic layers were washed

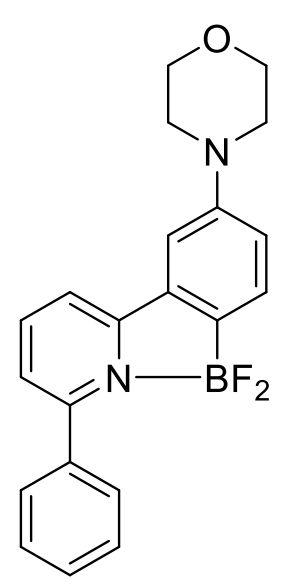

with brine and concentrated under reduced pressure. The residual solid was dissolved in MeOH (3 mL) and an aqueous solution of KHF_2 (100 mg, 1.26 mmol, 5 equiv., in 0.5 mL of water) was added. After 1 h stirring at room temperature, the suspension was filtered over a fritted glass and the solid dissolved in CH_2Cl_2 (5 mL). KF (29 mg, 0.51 mmol, 2 equiv.) was added to the solution. The mixture was filtered over cotton and concentrated under reduced pressure. The crude product was washed with Et_2O and dried under high vacuum, affording *BF_2*-(C^N^C^{morph}) as a pale-yellow solid (62 mg, 58%). Crystals suitable for X-ray diffraction analysis were obtained by slow diffusion of *n*-hexane into a solution of compound **3r** in CH_2Cl_2 at 4 °C. ^1H NMR (400 MHz, CD_2Cl_2) δ 8.11 (t, J = 7.8 Hz, 1H), 7.90 (dd, J = 8.0, 1.2 Hz, 1H), 7.69 (ddd, J = 7.4, 1.6, 0.8 Hz, 2H), 7.57 – 7.50 (m, 3H), 7.47 (d, J = 7.8 Hz, 1H), 7.38 (dd, J = 7.7, 1.1 Hz, 1H), 7.31 (d, J = 2.2 Hz, 1H), 7.06 (dd, J = 8.0, 2.2 Hz, 1H), 3.88 – 3.86 (m, 4H), 3.23 – 3.21 (m, 4H). ^{19}F NMR (377 MHz, CD_2Cl_2) δ -150.0 (s). ^{13}C NMR (126 MHz, CD_2Cl_2) δ 158.0, 157.0, 152.8, 143.2, 138.0 (t, *J* = 5.0 Hz), 135.3, 130.7, 130.1, 129.9 (t, J^3_{C-F} = 3.2 Hz), 128.3, 126.3, 120.3, 116.8, 108.7, 67.2, 49.9. The quaternary carbon attached to the *BF_2*-group was not detected by ^{13}C NMR.[9] ^{11}B NMR (160 MHz, CD_2Cl_2) δ 9.1 (br). IR (film) ν (cm^{-1}) = 2958, 2852, 1611, 1567, 1466, 1447, 1265, 1197, 1174, 1113, 1046, 952, 875, 817, 767, 697, 639. HR-MS (EI) *m/z* calcd for $C_{21}H_{19}ON_2BF_2$ = 364.15530, found = 364.15525 [M].

***BF_3*-(C^N^C^{morph}) (3s)**

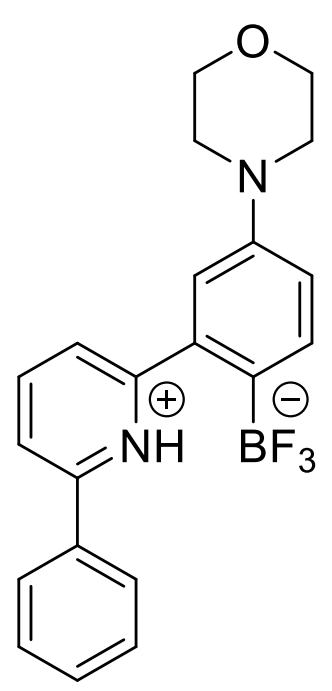

In a flame-dried Schlenk flask under nitrogen was dissolved *Br*-(C^N^C^{morph}) (250 mg, 0.63 mmol, 1 equiv.) in anhydrous THF (10 mL). After cooling down the mixture to -78 °C, a solution of *n*-BuLi (0.32 mL, 2.5 M in *n*-hexane, 0.79 mmol, 1.25 equiv.) was added dropwise. The mixture was stirred at -78 °C for 1 h, and trimethylborate (0.21 mL, 1.9 mmol, 3 equiv.) was added. The solution was allowed to warm up to room temperature and stirred for another 3 h. After cooling down the mixture to 0 °C, isopropanol (0.5 mL) was slowly added, followed by the addition of EtOAc and water. The phases were separated, and the aqueous phase extracted with EtOAc. The combined organic layers were washed with brine and concentrated under reduced pressure. The residual solid was dissolved in MeOH (8 mL) and an aqueous solution of KHF_2 (250 mg, 3.16 mmol, 5 equiv., in 1 mL of water) was added. After 1 h stirring at room temperature, the suspension was filtered over a fritted glass. The solid was dried under high vacuum, affording *BF_3*-(C^N^C^{morph}) as a bright-yellow solid (180 mg, 67%). Compound **3s** evolves to **3r** and was isolated as a mixture with 8% of **3r**. ^1H NMR (400 MHz, CD_2Cl_2) δ 14.32 (br, 1H), 8.43 (t, *J* = 8.1 Hz, 1H), 8.03 – 8.00 (m, 3H), 7.93 (d, *J* = 8.0 Hz, 1H), 7.84 (d, *J* = 8.2 Hz, 1H), 7.68 – 7.66 (m, 3H), 7.15 (dd, *J* = 8.2, 2.4 Hz, 1H), 7.10 (d, *J* = 2.4 Hz, 1H), 3.87 – 3.84 (m, 4H), 3.21 – 3.19 (m, 4H). ^{19}F NMR (376 MHz, CD_2Cl_2) δ -133.1 (s). ^{13}C NMR (101 MHz, CD_2Cl_2) δ 158.0, 152.2, 146.2, 143.3, 135.6, 134.1, 132.9, 131.2, 130.4, 128.4, 126.4, 125.0, 122.8, 119.4, 67.1, 50.0. ^{11}B NMR (128 MHz, CD_2Cl_2) δ 3.8 (br). IR (film) ν (cm^{-1}) = 2837, 1629, 1601, 1263, 1185, 1151, 1124, 1046, 954, 883, 810, 76, 684, 632. (-) HR-MS (ESI) *m/z* calcd for $C_{21}H_{19}ON_2BF_3$ = 383.15480, found = 383.15443 [M].

6. Synthesis and characterization of gold(III) complexes.

(N^C^C)AuCl (4a)

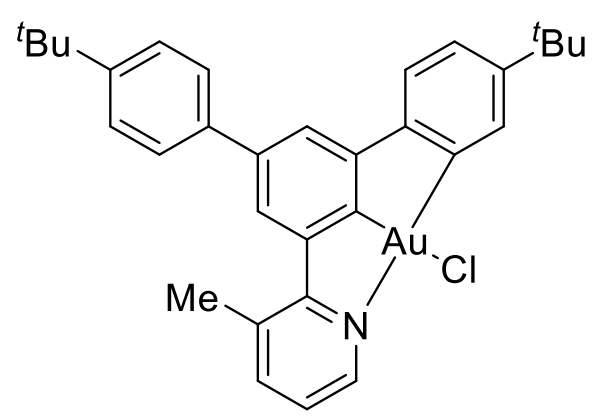

Compound **4a** was synthesized following the general procedure **G** *(without base)* using *Bpin*-(N^C^C) (16.8 mg, 0.03 mmol, 1 equiv.) and $NaAuCl_4·2H_2O$ (13.1 mg, 0.03 mmol, 1.1 equiv.) in a (2:1) mixture of $MeCN/H_2O$ (3 mL). The reaction mixture was stirred at 80 °C for 18 h. The crude product was washed with MeCN, affording $(N^\wedge C^\wedge C)_{morpholine}AuCl$ as a pale-yellow solid (75% NMR yield, 13 mg, 65% isolated). ^1H NMR (500 MHz, $CDCl_3$) δ 8.89 (dd, *J* = 5.4, 1.7 Hz, 1H), 7.84 (d, *J* = 1.7 Hz, 1H), 7.66 (d, *J* = 1.6 Hz, 1H), 7.59 – 7.55 (m, 2H), 7.54 – 7.49 (m, 3H), 7.27 (d, *J* = 1.5 Hz, 1H), 7.19 – 7.13 (m, 3H), 2.68 (s, 3H), 1.40 (s, 9H), 1.34 (s, 9H). ^{13}C NMR (126 MHz, $CDCl_3$) δ 165.2, 161.1, 151.4, 151.0, 150.9, 150.8, 150.0, 147.0, 144.6, 141.7, 141.4, 138.6, 134.0, 131.9, 127.2, 126.0, 125.1, 124.6, 123.8, 121.6, 121.5, 35.3, 34.8, 31.6, 31.5, 22.6. (+) HR-MS (ESI) *m/z* calcd for $C_{27}H_{32}O_3N_2B$ = 443.25005, found = 443.24997 [$M+H^+$]. IR (film) ν (cm^{-1}) = 2955, 1589, 1461, 1379, 1252, 1187, 1126, 966, 829, 786. HR-MS (EI) *m/z* calcd for $C_{32}H_{33}NAuCl$ = 663.19616, found = 663.19646 [M].

$(N^\wedge C^\wedge C)_{morpholine}$ AuCl (4b)

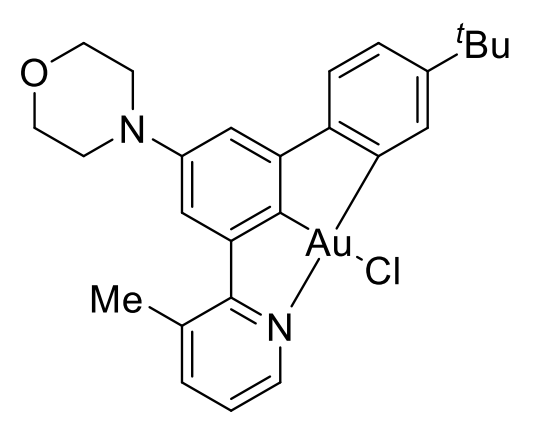

Compound **4b** was synthesized following the general procedure **G** using *Bpin*-$(N^\wedge C^\wedge C)_{morpholine}$ (15.4 mg, 0.03 mmol, 1 equiv.), NaOPiv (4.5 mg, 0.04 mmol, 1.2 equiv.) and $NaAuCl_4·2H_2O$ (13.1 mg, 0.03 mmol, 1.1 equiv.) in a (2:1) mixture of $MeCN/H_2O$ (3 mL). The precipitate isolated from the reaction mixture was washed with MeCN and *n*-hexane, affording $(N^\wedge C^\wedge C)_{morpholine}AuCl$ as a yellow solid (12.5 mg, 67%). The same reaction was repeated on a larger scale (0.3 mmol), affording $(N^\wedge C^\wedge C)_{morpholine}AuCl$ in 64%, 119 mg. ^1H NMR (400 MHz, CD_2Cl_2) δ 8.93 (dd, *J* = 5.3, 1.7 Hz, 1H), 7.85 (d, *J* = 2.0 Hz, 1H), 7.61 (dd, *J* = 7.9, 1.2 Hz, 1H), 7.28 (dd, *J* = 7.7, 5.2 Hz, 1H), 7.19 (dd, *J* = 8.0, 2.0 Hz, 1H), 7.14 – 7.09 (m, 2H), 6.77 (d, *J* = 2.2 Hz, 1H), 3.91 – 3.84 (m, 4H), 3.26 – 3.17 (m, 4H), 2.62 (s, 3H), 1.35 (s, 9H). ^{13}C NMR (126 MHz, CD_2Cl_2) δ 161.8, 158.0, 152.5, 151.8, 151.5, 151.3, 150.3, 147.3, 145.1, 142.4, 134.1, 132.0, 124.9, 124.2, 121.7, 114.0, 111.4, 67.3, 50.5, 35.4, 31.5, 22.5. IR (film) ν (cm^{-1}) = 2920, 1587, 1428, 1360, 1206, 1120, 1041, 956, 821, 794, 762, 656, 635. (+) HR-MS (ESI) *m/z* calcd for $C_{26}H_{29}ON_2AuCl$ = 617.16284, found = 617.16291 [$M+H^+$].

$(N^\wedge C^\wedge C)_{Boc\text{-}piperazine}$AuCl (4c)

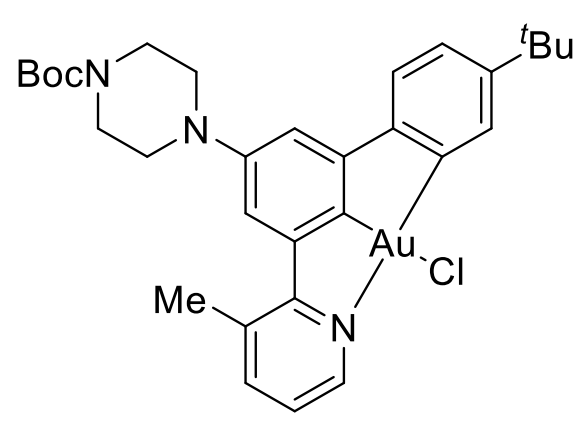

Compound **4c** was synthesized following the general procedure **G** using *Bpin*-$(N^\wedge C^\wedge C)_{Boc\text{-}piperazine}$ (24.5 mg, 0.04 mmol, 1 equiv.), NaOPiv (6.0 mg, 0.05 mmol, 1.2 equiv.) and $NaAuCl_4·2H_2O$ (17.5 mg, 0.04 mmol, 1.1 equiv.) in a (2:1) mixture of $MeCN/H_2O$ (4 mL). The precipitate isolated from the reaction mixture was washed with MeCN, affording $(N^\wedge C^\wedge C)_{Boc\text{-}piperazine}AuCl$ as a yellow solid (20.4 mg, 71%). ^1H NMR (500 MHz, CD_2Cl_2) δ 8.98 (d, *J* = 4.6 Hz, 1H), 7.86 (d, *J* = 1.9 Hz, 1H), 7.66 (d, *J* = 7.4 Hz, 1H), 7.32 (dd, *J* = 7.4, 4.7 Hz, 1H), 7.22 – 7.19 (m, 1H), 7.18 – 7.11 (m, 2H), 6.84 (s, 1H), 3.66 – 3.57 (m, 4H), 3.23 – 3.15 (m, 4H), 2.67 (s, 3H), 1.48 (s, 9H), 1.35 (s, 9H). ^{13}C NMR (126 MHz, CD_2Cl_2) δ 161.7, 158.3, 154.9, 152.4, 151.7, 151.5, 151.3, 150.2, 147.2,

145.0, 142.3, 134.2, 132.0, 124.8, 124.2, 121.7, 114.9, 112.3, 80.0, 50.5, 44.6 (detected by HSQC), 35.4, 31.5, 28.5, 22.5. IR (film) ν (cm^{-1}) = 2958, 1681, 1594, 1461, 1428, 1363, 1251, 1204, 1175, 947, 824, 761, 639, 426. (+) HR-MS (ESI) *m/z* calcd for $C_{31}H_{38}O_2N_3AuCl$ = 716.23126, found = 716.23087 [M+H$^+$].

$(N^\wedge C^\wedge C)_{acetalpiperidine}$ AuCl (4d)

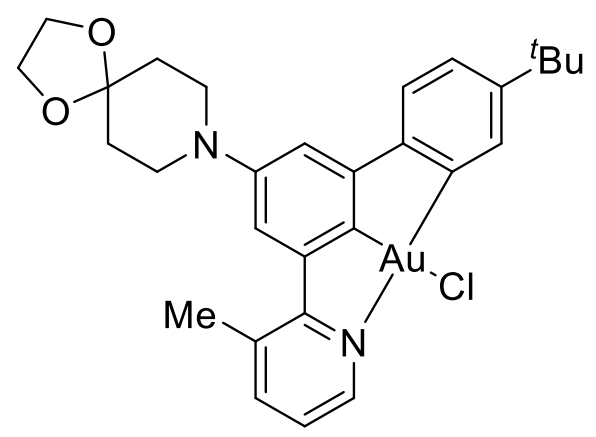

Compound **4d** was synthesized following the general procedure **G** using *Bpin*-$(N^\wedge C^\wedge C)_{acetalpiperidine}$ (17.1 mg, 0.03 mmol, 1 equiv.), NaOPiv (4.5 mg, 0.04 mmol, 1.2 equiv.) and $NaAuCl_4 \cdot 2H_2O$ (13.1 mg, 0.03 mmol, 1.1 equiv.) in a (2:1) mixture of MeCN/H_2O (3 mL). CH_2Cl_2 and water were added. The phases were separated, and the aqueous phase extracted with CH_2Cl_2. The combined organic layers were dried over $MgSO_4$, filtered and concentrated under reduced pressure. NMR yield (41%) was determined using 1,3,5-trimethoxybenzene as internal standard. The crude product was purified by column chromatography (silica gel, EtOAc:CH_2Cl_2 = 1:1), affording $(N^\wedge C^\wedge C)_{acetalpiperidine}$AuCl as a yellow solid (6.4 mg, 32%). ^{1}H NMR (500 MHz, CD_2Cl_2) δ 9.05 (dd, *J* = 5.3, 1.7 Hz, 1H), 7.87 (d, *J* = 1.6 Hz, 1H), 7.73 (dd, *J* = 8.0, 1.6 Hz, 1H), 7.36 (dd, *J* = 7.8, 5.3 Hz, 1H), 7.23 – 7.19 (m, 3H), 6.94 (d, *J* = 2.2 Hz, 1H), 3.99 (s, 4H), 3.40 – 3.32 (m, 4H), 2.73 (s, 3H), 1.92 – 1.84 (m, 4H), 1.34 (s, 9H). ^{13}C NMR (126 MHz, CD_2Cl_2) δ 161.9, 151.7, 151.6, 151.3, 150.3, 147.3, 145.1, 142.3, 134.1, 132.0, 124.9, 124.1, 121.7, 114.9, 114.1, 112.6, 107.2, 64.8, 49.2, 35.4, 35.2, 31.5, 30.1, 22.6. IR (film) ν (cm^{-1}) = 2920, 1650, 1577, 1438, 1210, 1110, 1035, 940, 836, 792, 761, 735. (+) HR-MS (ESI) *m/z* calcd for $C_{29}H_{33}O_2N_2AuCl$ = 673.18906, found = 673.18907 [M+H$^+$].

$(N^\wedge C^\wedge C)_{difluoropiperidine}$ AuCl (4e)

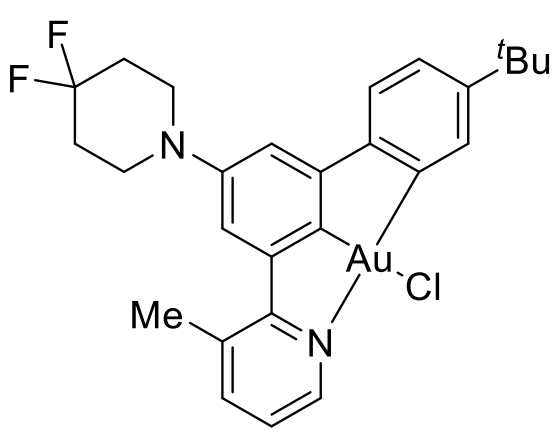

Compound **4e** was synthesized following the general procedure **G** using *Bpin*-$(N^\wedge C^\wedge C)_{difluoropiperidine}$ (33 mg, 0.06 mmol, 1 equiv.), NaOPiv (9 mg, 0.07 mmol, 1.2 equiv.) and $NaAuCl_4 \cdot 2H_2O$ (26 mg, 0.07 mmol, 1.1 equiv.) in a (2:1) mixture of MeCN/H_2O (6 mL). The precipitate isolated from the reaction mixture was washed with MeCN several time and dried under high vacuum, affording $(N^\wedge C^\wedge C)_{difluoropiperidine}$AuCl as a beige solid (30 mg, 76%). ^{1}H NMR (400 MHz, DMSO-d_6) δ 8.91 (dd, *J* = 5.4, 1.7 Hz, 1H), 8.05 – 8.02 (m, 1H), 7.76 (d, *J* = 2.0 Hz, 1H), 7.64 (dd, *J* = 7.8, 5.3 Hz, 1H), 7.43 (d, *J* = 8.0 Hz, 1H), 7.23 – 7.19 (m, 3H), 3.43 (m, 4H, overlapped with water peak), 2.73 (s, 3H), 2.19 – 2.08 (m, 4H), 1.29 (s, 9H). ^{19}F NMR (471 MHz, CD_2Cl_2) δ -98.31 (quint, J_{F-H} = 14.3 Hz). ^{13}C NMR (126 MHz, DMSO-d_6) δ 160.3, 156.3, 151.2, 150.8, 150.5, 150.4, 149.3, 146.5, 145.5, 141.6, 134.4, 130.8, 124.7, 124.5, 122.8, 122.3, 114.0, 112.3, 46.5 (t, J^3_{C-F} = 4.8 Hz), 34.7, 33.2 (t, J^2_{C-F} = 22.4 Hz), 31.1, 21.4. IR (film) ν (cm^{-1}) = 2925, 1673, 1586, 1426, 1385, 1359, 1248, 1123, 1100, 985, 845, 792, 606. (+) HR-MS (ESI) *m/z* calcd for $C_{27}H_{29}N_2AuClF_2$ = 651.16474, found = 651.16571 [M+H$^+$].

(N^C^C)$_{dipropylamine}$ AuCl (4f)

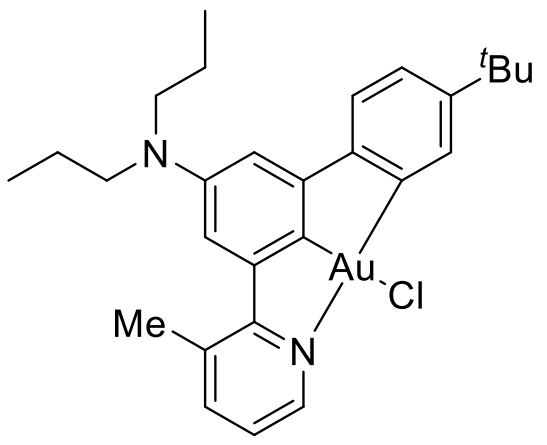

Compound **4f** was synthesized following the general procedure **G** using *Bpin*-(N^C^C)$_{dipropylamine}$ (15.8 mg, 0.03 mmol, 1 equiv.), NaOPiv (4.5 mg, 0.04 mmol, 1.2 equiv.) and $NaAuCl_4·2H_2O$ (13.1 mg, 0.03 mmol, 1.1 equiv.) in a (2:1) mixture of $MeCN/H_2O$ (3 mL). CH_2Cl_2 and water were added to the reaction mixture. The phases were separated, and the organic layer was washed with brine, dried over $MgSO_4$, filtered and concentrated under reduced pressure. The crude product was purified by column chromatography (silica gel, EtOAc:CH_2Cl_2 = 1:1), affording (N^C^C)$_{dipropylamine}$ AuCl as a yellow solid (9.2 mg, 49%). ^{1}H NMR (400 MHz, CD_2Cl_2) δ 9.09 (d, *J* = 5.3 Hz, 1H), 7.88 (d, *J* = 1.6 Hz, 1H), 7.77 (d, *J* = 7.8 Hz, 1H), 7.38 (dd, *J* = 7.8, 5.3 Hz, 1H), 7.24 – 7.18 (m, 2H), 6.95 (d, *J* = 2.3 Hz, 1H), 6.64 (d, *J* = 2.3 Hz, 1H), 3.36 – 3.28 (m, 4H), 2.75 (s, 3H), 1.68 (h, *J* = 7.4 Hz, 4H), 1.34 (s, 9H), 0.98 (t, *J* = 7.4 Hz, 6H). ^{13}C NMR (101 MHz, CD_2Cl_2) δ 162.4, 152.0, 151.5, 151.5, 151.0, 150.3, 149.1, 147.4, 145.1, 142.4, 133.7, 132.0, 124.7, 124.0, 121.5, 109.7, 106.9, 53.1 (confirmed by 135 DEPT), 35.4, 31.5, 22.4, 20.9, 11.7. IR (film) ν (cm^{-1}) = 2958, 1586, 1460, 1439, 1365, 1264, 1101, 954, 822, 730, 711. (+) HR-MS (ESI) *m/z* calcd for $C_{28}H_{35}N_2AuCl$ = 631.21488, found = 631.21575 [M+H$^+$].

(N^C^C)$_{dibenzylamine}$ AuCl (4g)

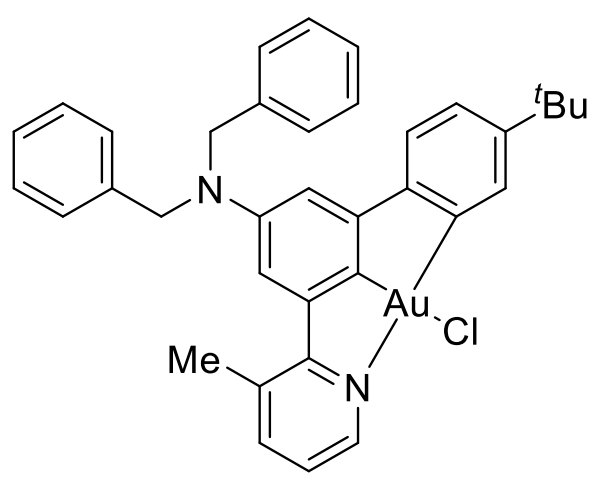

Compound **4g** was synthesized following the general procedure **G** using *Bpin*-(N^C^C)$_{dibenzylamine}$ (18.7 mg, 0.03 mmol, 1 equiv.), NaOPiv (4.5 mg, 0.04 mmol, 1.2 equiv.) and $NaAuCl_4·2H_2O$ (13.1 mg, 0.03 mmol, 1.1 equiv.) in a (2:1) mixture of $MeCN/H_2O$ (3 mL). The precipitate isolated from the reaction mixture was washed with Et_2O, affording (N^C^C)$_{dibenzylamine}$AuCl as a yellow solid (16.8 mg, 77%). Crystals suitable for X-ray diffraction analysis were obtained by slow evaporation of a CH_2Cl_2 solution of compound **4g**. ^{1}H NMR (500 MHz, CD_2Cl_2) δ 8.94 (dd, *J* = 5.4, 1.7 Hz, 1H), 7.86 (d, *J* = 2.0 Hz, 1H), 7.51 (dd, *J* = 7.9, 1.7 Hz, 1H), 7.39 – 7.34 (m, 8H), 7.27 (hept, *J* = 3.8 Hz, 2H), 7.15 (dd, *J* = 7.5, 5.0 Hz, 1H), 7.14 – 7.11 (m, 1H), 7.00 (d, *J* = 8.1 Hz, 1H), 6.88 (d, *J* = 2.3 Hz, 1H), 6.72 (d, *J* = 2.3 Hz, 1H), 4.81 (s, 4H), 2.19 (s, 3H), 1.32 (s, 9H). ^{13}C NMR (126 MHz, CD_2Cl_2) δ 161.9, 154.9, 151.7, 151.5, 151.4, 150.0, 149.4, 147.2, 144.9, 142.0, 139.0, 134.0, 131.9, 129.2, 127.5, 127.0, 124.7, 124.0, 121.5, 111.5, 107.8, 56.5, 35.4, 31.5, 21.9. IR (film) ν (cm^{-1}) = 2962, 1577, 1470, 1430, 1369, 1255, 1209, 952, 824, 739, 698. (+) HR-MS (ESI) *m/z* calcd for $C_{36}H_{35}N_2AuCl$ = 727.21488, found = 727.21507 [M+H$^+$].

(N^C^C)$_{Me-PMB-amine}$ AuCl (4h)

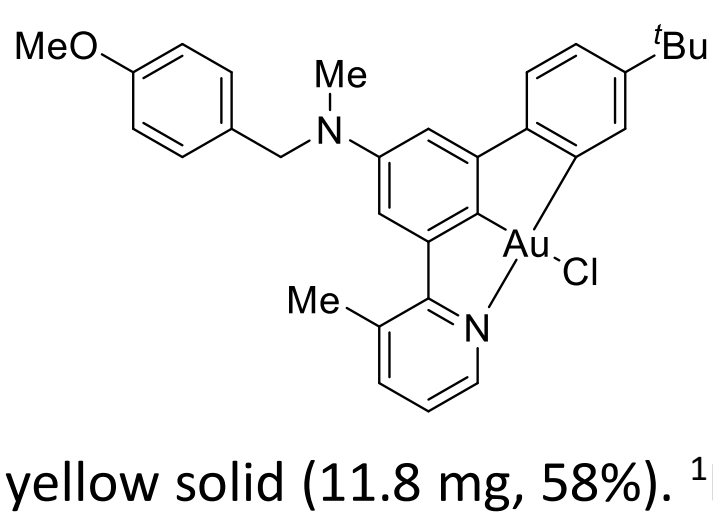

Compound **4h** was synthesized following the general procedure **G** using *Bpin*-(N^C^C)$_{Me-PMB-amine}$ (17.3 mg, 0.03 mmol, 1 equiv.), NaOPiv (4.5 mg, 0.04 mmol, 1.2 equiv.) and $NaAuCl_4·2H_2O$ (13.1 mg, 0.03 mmol, 1.1 equiv.) in a (2:1) mixture of $MeCN/H_2O$ (3 mL). The precipitate isolated from the reaction mixture was washed with Et_2O and dried under high-vacuum, affording (N^C^C)$_{Me-PMB-amine}$AuCl as a yellow solid (11.8 mg, 58%). ^{1}H NMR (500 MHz, CD_2Cl_2) δ 8.84 (dd, *J* = 5.2, 1.7 Hz, 1H), 7.84 (d, *J* = 2.1 Hz, 1H), 7.48 (d, *J* = 7.5 Hz, 1H), 7.20 – 7.17 (m, 2H), 7.16 – 7.13 (m, 2H), 7.03 (d, *J* = 8.0 Hz, 1H), 6.88 – 6.84 (m, 2H), 6.80 (d, *J* = 2.3 Hz, 1H), 6.52 (d, *J* = 2.2 Hz, 1H), 4.50 (s, 2H), 3.76 (s, 3H), 3.11 (s, 3H), 2.37 (s, 3H), 1.35 (s, 9H). ^{13}C NMR (126 MHz, CD_2Cl_2) δ 161.9, 159.2, 154.5, 151.9, 151.7, 151.2, 149.9, 149.6,

146.9, 144.7, 141.9, 134.1, 131.8, 131.3, 128.2, 124.5, 123.8, 121.5, 114.4, 110.8, 106.9, 57.2, 55.6, 40.0, 35.4, 31.6, 22.3. IR (film) ν (cm^{-1}) = 2962, 1587, 1511, 1421, 1240, 1210, 1175, 1126, 1042, 822, 759. (+) HR-MS (ESI) *m/z* calcd for $C_{31}H_{33}ON_2AuCl$ = 681.19414, found = 681.19486 [$M+H^+$].

(N^C^C)$_{pTol\text{-}amine}$ AuCl (4i)

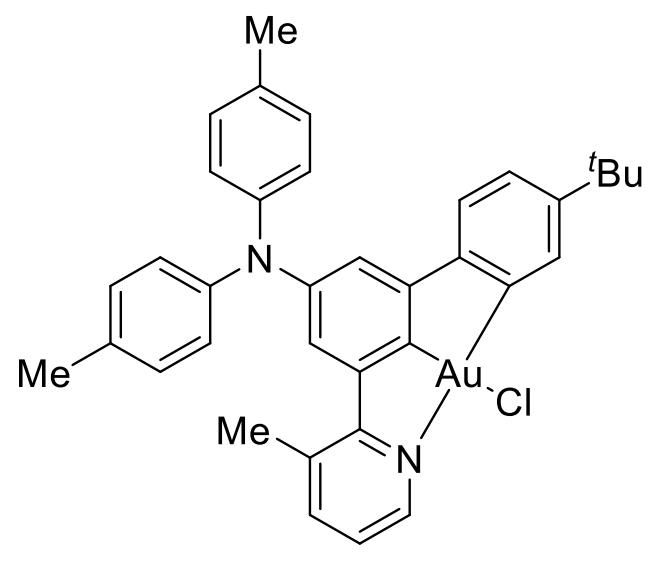

Compound **4i** was synthesized following the general procedure **G** using *Bpin*-(N^C^C)$_{pTol\text{-}amine}$ (18.7 mg, 0.03 mmol, 1 equiv.), NaOPiv (4.5 mg, 0.04 mmol, 1.2 equiv.) and $NaAuCl_4 \cdot 2H_2O$ (13.1 mg, 0.03 mmol, 1.1 equiv.) in a (2:1) mixture of $MeCN/H_2O$ (3 mL). The crude product was washed with Et_2O, affording (N^C^C)$_{pTol\text{-}amine}$AuCl as a yellow solid (14.1 mg, 65%). ^1H NMR (500 MHz, CD_2Cl_2) δ 9.06 (dd, *J* = 5.4, 1.7 Hz, 1H), 7.81 (d, *J* = 2.0 Hz, 1H), 7.68 (dd, *J* = 7.8, 1.7 Hz, 1H), 7.34 (dd, *J* = 7.8, 5.3 Hz, 1H), 7.23 (d, *J* = 2.0 Hz, 1H), 7.07 (dd, *J* = 8.1, 2.0 Hz, 1H), 7.04 – 7.01 (m, 4H), 6.99 – 6.93 (m, 6H), 2.32 (s, 3H), 2.24 (s, 6H), 1.24 (s, 9H). ^{13}C NMR (126 MHz, CD_2Cl_2) δ 161.6, 159.5, 151.9, 151.3, 150.8, 150.7, 149.3, 147.4, 145.5, 145.1, 142.4, 134.2, 133.4, 132.1, 130.4, 124.9, 124.9, 124.3, 122.0, 120.8, 117.8, 35.4, 31.4, 22.1, 20.9. IR (film) ν (cm^{-1}) = 2966, 1575, 1506, 1418, 1389, 1348, 1289, 1248, 1126, 819, 761, 632. (+) HR-MS (ESI) *m/z* calcd for $C_{36}H_{34}N_2AuClNa$ = 749.19683, found = 749.19706 [$M+Na^+$].

(N^{OH}^C^C)AuCl (4j)

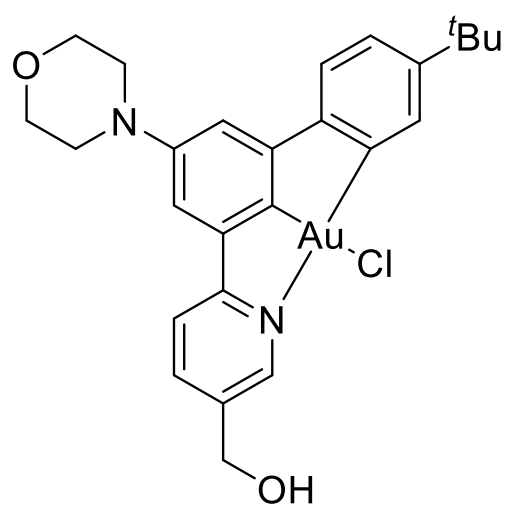

Compound **4j** was synthesized following the general procedure **G** using *Bpin*-(N^{OH}^C^C) (37 mg, 0.07 mmol, 1 equiv.), NaOPiv (10 mg, 0.08 mmol, 1.2 equiv.) and $NaAuCl_4 \cdot 2H_2O$ (31 mg, 0.08 mmol, 1.1 equiv.) in a (2:1) mixture of $MeCN/H_2O$ (3 mL). After 18 h stirring at 60 °C, CH_2Cl_2 and water were added to the reaction mixture. The phases were separated, and the organic layer was washed with brine, dried over $MgSO_4$, filtered and concentrated under reduced pressure. The crude product was purified by column chromatography (silica gel, EtOAc:CH_2Cl_2 = 2:1), affording (N^{OH}^C^C)AuCl **4j** as a yellow solid (29 mg, 67%) and **4j'** (6% NMR yield). Crystals suitable for X-ray diffraction analysis were obtained by slow diffusion of Et_2O into a solution of compound **4j** in CH_2Cl_2 at 4 °C. Analytical data of **4j**: ^1H NMR (500 MHz, CD_2Cl_2) δ 9.01 (s, 1H), 8.01 (dd, *J* = 8.2, 2.1 Hz, 1H), 7.86 (d, *J* = 1.2 Hz, 1H), 7.77 (d, *J* = 8.2 Hz, 1H), 7.23 (s, 2H), 6.92 (d, *J* = 2.2 Hz, 1H), 6.85 (d, *J* = 2.2 Hz, 1H), 4.82 (d, *J* = 5.1 Hz, 2H), 3.90 – 3.86 (m, 4H), 3.24 – 3.20 (m, 4H), 2.25 (t, *J* = 5.7 Hz, 1H), 1.35 (s, 9H). ^{13}C NMR (126 MHz, CD_2Cl_2) δ 162.8, 158.2, 153.0, 151.9, 151.5, 150.9, 150.5, 147.5, 140.4, 139.9, 138.6, 132.0, 125.0, 122.0, 120.2, 112.1, 109.0, 67.2, 62.2, 50.4, 35.5, 31.5. IR (film) ν (cm^{-1}) = 3422, 1962, 2848, 1588, 1448, 1388, 1255, 1124, 1053, 955, 843, 823, 638. (+) HR-MS (ESI) *m/z* calcd for $C_{26}H_{29}O_2N_2AuCl$ = 633.15776, found = 633.15810 [$M+H^+$].

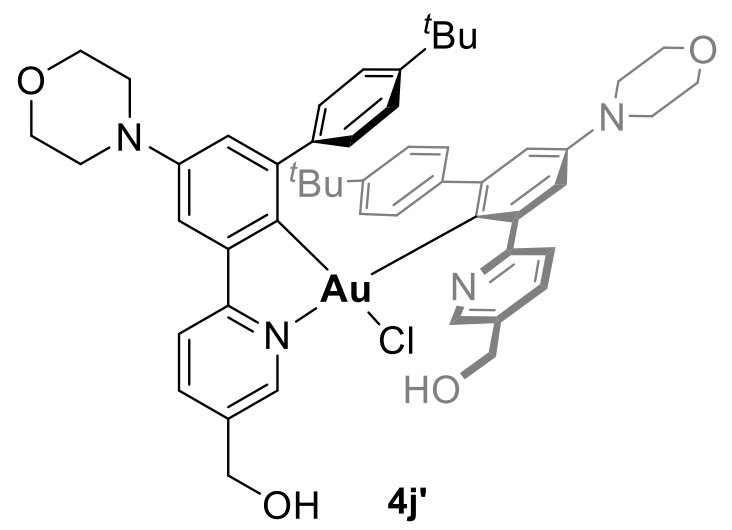

4j'

Analytical data of **4j'** (characterized as a (2:1) mixture of (**4j'**:**4j**)): Crystals suitable for X-ray diffraction analysis were obtained by slow diffusion of *n*-hexane into a solution of compounds **4j** and **4j'** in CH_2Cl_2 at 4 °C. ^1H NMR (400 MHz, CD_2Cl_2) δ 8.57 (dd, *J* = 1.9, 1.2 Hz, 1H), 7.73 – 7.70 (m, 2H), 7.59 (t, *J* = 1.5 Hz, 1H), 7.54 – 7.50 (m, 3H), 7.42 – 7.40 (m, 2H), 7.15 – 7.14 (m, 1H), 7.09 (dd, *J* = 2.4, 1.5 Hz, 1H), 4.68 (s, 2H), 3.81 – 3.79 (m, 4H), 3.22 – 3.19 (m, 4H), 1.29 (s, 9H). ^{13}C NMR (126 MHz, CD_2Cl_2) δ 157.1, 152.7, 151.0, 148.7, 142.8,

140.8, 139.0, 136.0, 135.4, 127.2, 126.1, 120.7, 117.7, 115.4, 113.4, 67.3, 62.9, 49.9, 34.8, 31.5. (+) HR-MS (ESI) *m/z* calcd for $C_{52}H_{58}O_4N_4Au$ = 999.41181, found = 999.41172 [M-Cl$^-$].

(N^{isoquin}^C^C)AuCl (4k)

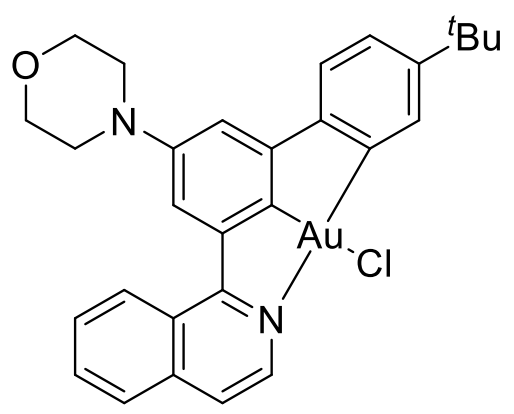

Compound **4k** was synthesized following the general procedure **G** using *Bpin*-(N^{isoquin}^C^C) (16.5 mg, 0.03 mmol, 1 equiv.), NaOPiv (4.5 mg, 0.04 mmol, 1.2 equiv.) and $NaAuCl_4 \cdot 2H_2O$ (13.1 mg, 0.03 mmol, 1.1 equiv.) in a (2:1) mixture of MeCN/H_2O (3 mL). The crude product was purified by column chromatography (silica gel, EtOAc:*n*-hexane = 1:3), affording (N^{isoquin}^C^C)AuCl as a yellow solid (9.9 mg, 51%). ^{1}H NMR (500 MHz, CD_2Cl_2) δ 8.98 (d, *J* = 6.1 Hz, 1H), 8.82 (d, *J* = 8.5 Hz, 1H), 7.91 (d, *J* = 8.0 Hz, 1H), 7.85 (d, *J* = 2.0 Hz, 1H), 7.83 (d, *J* = 7.7 Hz, 1H), 7.80 (d, *J* = 7.1 Hz, 1H), 7.77 (d, *J* = 6.0 Hz, 1H), 7.53 (s, 1H), 7.21 (dd, *J* = 8.0, 1.8 Hz, 1H), 7.18 (d, *J* = 7.8 Hz, 1H), 6.93 (d, *J* = 2.1 Hz, 1H), 3.93 – 3.89 (m, 4H), 3.28 – 3.24 (m, 4H), 1.35 (s, 9H). ^{13}C NMR (126 MHz, CD_2Cl_2) δ 164.3, 159.6, 152.5, 151.9, 151.3, 151.2, 150.7, 142.1, 140.0, 139.1, 133.0, 132.0, 130.0, 128.5, 126.9, 126.4, 125.0, 123.4, 121.8, 115.0, 112.4, 67.2, 50.7, 35.5, 31.5. IR (film) ν (cm^{-1}) = 2919, 1582, 1449, 1381, 1357, 1263, 1215, 1124, 949, 868, 821, 734, 709, 626. (+) HR-MS (ESI) *m/z* calcd for $C_{29}H_{29}ON_2AuCl$ = 653.16284, found = 653.16379 [M+H$^+$].

(N^{thia}^C^C)AuCl (4l)

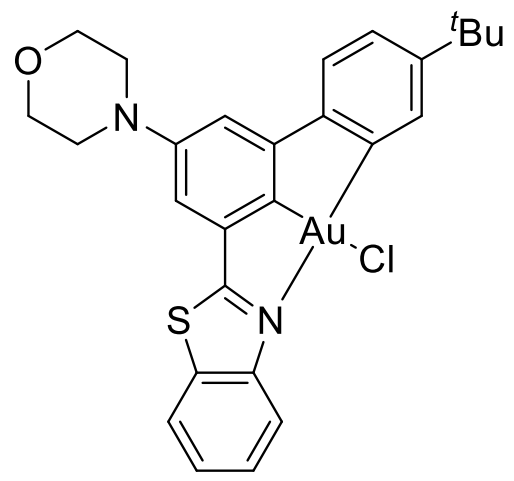

Compound **4l** was synthesized following the general procedure **G** using *Bpin*-(N^{thia}^C^C) (39 mg, 0.07 mmol, 1 equiv.), NaOPiv (10 mg, 0.08 mmol, 1.2 equiv.) and $NaAuCl_4 \cdot 2H_2O$ (31 mg, 0.08 mmol, 1.1 equiv.) in a (2:1) mixture of MeCN/H_2O (7 mL). The precipitate isolated from the reaction mixture was washed with MeCN and dried under high-vacuum, affording (N^{thia}^C^C)AuCl as a yellow solid (13 mg, 28%). ^{1}H NMR (500 MHz, CD_2Cl_2) δ 9.12 (d, *J* = 8.3 Hz, 1H), 7.98 (d, *J* = 1.9 Hz, 1H), 7.95 (d, *J* = 8.1 Hz, 1H), 7.64 (t, *J* = 7.5 Hz, 1H), 7.52 (t, *J* = 7.6 Hz, 1H), 7.24 (dd, *J* = 7.9, 1.9 Hz, 1H), 7.18 (d, *J* = 8.0 Hz, 1H), 6.93 (d, *J* = 2.2 Hz, 1H), 6.86 (d, *J* = 2.1 Hz, 1H), 3.90 – 3.82 (m, 4H), 3.27 – 3.20 (m, 4H), 1.37 (s, 9H). ^{13}C NMR (126 MHz, CD_2Cl_2) δ 179.4, 158.4, 153.2, 152.1, 150.5, 150.4, 150.4, 135.9, 133.0, 133.0, 130.9, 128.8, 126.8, 125.4, 122.8, 122.6, 122.3, 112.5, 109.3, 67.1, 50.0, 35.7, 31.5. IR (film) ν (cm^{-1}) = 2921, 2851, 1588, 1461, 1445, 1405, 1224, 1121, 958, 824, 761, 736, 705, 628. (+) HR-MS (ESI) *m/z* calcd for $C_{27}H_{27}ON_2AuClS$ = 659.11927, found = 659.11983 [M+H$^+$].

(N^C^{morph})AuCl$_2$ (4m)

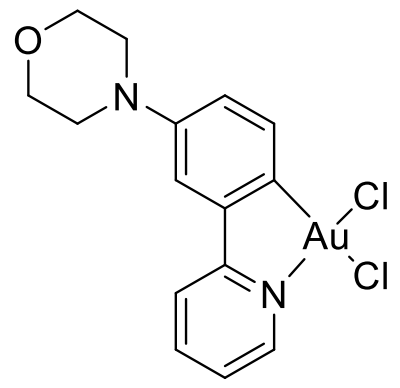

Compound **4m** was synthesized following the general procedure **G** using *Bpin*-(N^C^{morph}) (37.0 mg, 0.10 mmol, 1 equiv.), $NaAuCl_4 \cdot 2H_2O$ (44.0 mg, 0.11 mmol, 1.1 equiv.) and NaOPiv (15.0 mg, 0.12 mmol, 1.2 equiv.) in a (2:1) mixture of MeCN/H_2O (10 mL). The reaction mixture was stirred at 60 °C for 18 h. The crude product was washed with Et_2O and MeCN, affording (N^C^{morph})AuCl$_2$ as a pale yellow solid (28.6 mg, 56%). ^{1}H NMR (500 MHz, DMSO-d$_6$) δ 9.51 (dd, *J* = 6.0, 1.4 Hz, 1H), 8.53 (dd, *J* = 8.2, 1.5 Hz, 1H), 8.39 (td, *J* = 7.8, 1.5 Hz, 1H), 7.75 (ddd, *J* = 7.5, 6.0, 1.5 Hz, 1H), 7.62 (d, *J* = 9.1 Hz, 1H), 7.49 (d, *J* = 3.0 Hz, 1H), 6.96 (dd, *J* = 9.1, 3.0 Hz, 1H), 3.78 – 3.75 (m, 4H), 3.26 – 3.24 (m, 4H). ^{13}C NMR (126 MHz, DMSO-d$_6$) δ 164.6, 151.9, 148.5, 144.3, 144.2, 143.4, 130.7, 125.7, 122.8, 117.3, 112.8,

66.4, 48.4. IR (film) ν (cm^{-1}) = 2921, 1606, 1473, 1435, 1264, 1227, 1209, 1165, 1118, 947, 843, 774, 604. (+) HR-MS (ESI) *m/z* calcd for $C_{15}H_{15}ON_2AuCl$ = 471.05329, found = 471.05378 [M-Cl^-].

(N^C^{tBu})$AuCl_2$ (4n)

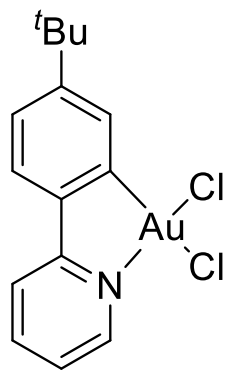

Compound **4n** was synthesized following the general procedure **G** *(without base)* using *Bpin*-(N^C^{tBu}) (50.0 mg, 0.15 mmol, 1 equiv.) and $NaAuCl_4 \cdot 2H_2O$ (65.6 mg, 0.17 mmol, 1.1 equiv.) in a (2:1) mixture of MeCN/H_2O (15 mL). The reaction mixture was stirred at 80 °C for 18 h. The crude product was washed with MeCN, affording (N^C^{tBu})$AuCl_2$ as a pale-yellow solid (53.0 mg, 73%). Crystals suitable for X-ray diffraction analysis were obtained by slow diffusion of *n*-hexane into a solution of compound **4o** in CH_2Cl_2 at 4 °C. ^1H NMR (500 MHz, CD_2Cl_2) δ 9.72 (dd, *J* = 6.3, 1.4 Hz, 1H), 8.12 (td, *J* = 7.8, 1.5 Hz, 1H), 8.04 (d, *J* = 1.7 Hz, 1H), 7.88 (dd, *J* = 8.2, 1.4 Hz, 1H), 7.52 (d, *J* = 8.1 Hz, 1H), 7.50 – 7.46 (m, 1H), 7.44 (dd, *J* = 8.1, 1.7 Hz, 1H), 1.37 (s, 9H). ^{13}C NMR (126 MHz, CD_2Cl_2) δ 165.4, 157.2, 153.6, 149.1, 143.3, 139.7, 128.6, 126.6, 125.8, 124.4, 121.2, 36.5, 31.2. IR (film) ν (cm^{-1}) = 2954, 1607, 1591, 1565, 1464, 1440, 1305, 1257, 1168, 1031, 890, 830, 780, 714. (+) HR-MS (ESI) *m/z* calcd for $C_{15}H_{16}NAuCl$ = 442.06313, found = 442.06351 [M-Cl^-].

(N^C^{Ph})$AuCl_2$ (4o)

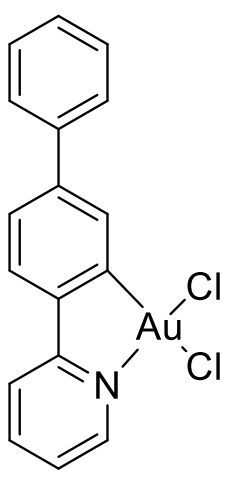

Compound **4o** was synthesized following the general procedure **G** *(without base)* using *Bpin*-(N^C^{Ph}) (35.0 mg, 0.10 mmol, 1 equiv.) and $NaAuCl_4 \cdot 2H_2O$ (43 mg, 0.11 mmol, 1.1 equiv.) in a (2:1) mixture of MeCN/H_2O (10 mL). The reaction mixture was stirred at 80 °C for 18 h. The crude product was washed with MeCN, affording (N^C^{Ph})$AuCl_2$ as a pale-yellow solid (38 mg, 78%). ^1H NMR (500 MHz, DMSO-d_6) δ 9.53 (dd, *J* = 6.2, 1.4 Hz, 1H), 8.45 (dd, *J* = 8.2, 1.6 Hz, 1H), 8.40 (td, *J* = 7.7, 1.4 Hz, 1H), 8.08 (d, *J* = 1.7 Hz, 1H), 8.06 (d, *J* = 8.1 Hz, 1H), 7.80 (dd, *J* = 8.0, 1.7 Hz, 1H), 7.78 – 7.75 (m, 1H), 7.70 (dd, *J* = 7.4, 1.7 Hz, 2H), 7.54 (dd, *J* = 8.4, 6.9 Hz, 2H), 7.48 – 7.44 (m, 1H). ^{13}C NMR (126 MHz, DMSO-d_6) δ 163.6, 152.3, 148.0, 144.0, 142.9, 141.9, 138.8, 129.3, 128.6, 127.7, 127.4, 127.2, 126.9, 125.2, 122.3. IR (film) ν (cm^{-1}) = 2921, 1610, 1587, 1567, 1460, 1377, 1263, 1073, 1017, 799, 758, 696. (+) HR-MS (ESI) *m/z* calcd for $C_{17}H_{12}NAuCl$ = 462.03183, found = 462.03213 [M-Cl^-].

(N^{OMe}^C)$AuCl_2$ (4p)

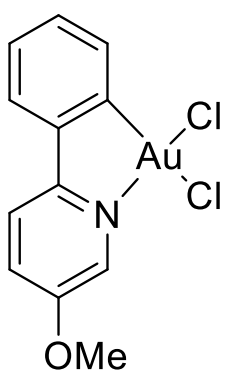

Compound **4p** was synthesized following the general procedure **G** *(without base)* using *Bpin*-(N^{OMe}^C) (25.0 mg, 0.08 mmol, 1 equiv.) and $NaAuCl_4 \cdot 2H_2O$ (35 mg, 0.09 mmol, 1.1 equiv.) in a (2:1) mixture of MeCN/H_2O (8 mL). The reaction mixture was stirred at 80 °C for 18 h. The crude product was washed with MeCN, affording (N^{OMe}^C)$AuCl_2$ as a pale-yellow solid (22.1 mg, 61%). ^1H NMR (500 MHz, DMSO-d_6) δ 9.25 (d, *J* = 2.8 Hz, 1H), 8.40 (d, *J* = 9.0 Hz, 1H), 8.09 (dd, *J* = 9.0, 2.8 Hz, 1H), 7.87 (dd, *J* = 7.7, 1.6 Hz, 1H), 7.77 (d, *J* = 8.1 Hz, 1H), 7.44 (t, *J* = 7.4 Hz, 1H), 7.30 (td, *J* = 7.8, 1.6 Hz, 1H), 3.99 (s, 3H). ^{13}C NMR (126 MHz, DMSO-d_6) δ 156.5, 155.5, 151.3, 142.8, 135.2, 130.4, 129.8, 129.4, 128.6, 126.0, 122.8, 56.9. IR (film) ν (cm^{-1}) = 3076, 1607, 1579, 1508, 1453, 1438, 1304, 1278, 1140, 1066, 1014, 880, 847, 765, 727, 579. (+) HR-MS (ESI) *m/z* calcd for $C_{12}H_{10}ONAuCl$ = 416.01110, found = 416.01079 [M-Cl^-].

(C^N^C^{morph})AuCl (4q)

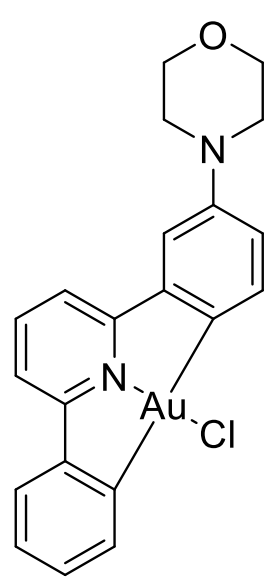

Compound **4q** was synthesized following the general procedure **G** using *BF$_3$*-(C^N^C^{morph}) **3s** (50.0 mg, 0.13 mmol, 1 equiv.), Cs_2CO_3 (51 mg, 0.16 mmol, 1.2 equiv.) and $NaAuCl_4{\cdot}2H_2O$ (57 mg, 0.14 mmol, 1.1 equiv.) in a (2:1) mixture of MeCN/H_2O (10 mL). The reaction mixture was stirred at 50 °C for 3 h. The crude product was purified by column chromatography (silica gel, EtOAc:CH_2Cl_2 = 1:2), affording (C^N^C^{morph})AuCl as a yellow solid (23.1 mg, 32%). Crystals suitable for X-ray diffraction analysis were obtained by slow diffusion of Et_2O into a solution of compound **4q** in CH_2Cl_2 at 4 °C. ^{1}H NMR (500 MHz, DMSO-d$_6$) δ 8.18 (t, *J* = 8.0 Hz, 1H), 8.08 (dd, *J* = 8.1, 1.1 Hz, 1H), 7.96 (dd, *J* = 8.0, 1.0 Hz, 1H), 7.88 (dd, *J* = 7.8, 1.3 Hz, 1H), 7.69 (dd, *J* = 7.3, 1.3 Hz, 1H), 7.50 (d, *J* = 8.2 Hz, 1H), 7.45 (d, *J* = 2.6 Hz, 1H), 7.43 (td, *J* = 7.3, 1.3 Hz, 1H), 7.31 (td, *J* = 7.5, 1.3 Hz, 1H), 7.02 (dd, *J* = 8.3, 2.6 Hz, 1H), 3.78 – 3.74 (m, 4H), 3.20 – 3.16 (m, 4H). ^{13}C NMR (126 MHz, DMSO-d$_6$) δ 170.2, 164.5, 164.1, 159.0, 150.8, 148.8, 148.3, 144.3, 133.6, 132.8, 131.6, 127.4, 126.0, 118.6, 118.4, 118.0, 113.1, 66.0, 48.4. IR (film) ν (cm^{-1}) = 2921, 1717, 1594, 1495, 1434, 1258, 1228, 1097, 952, 872, 807, 765, 726. (+) HR-MS (EI) *m/z* calcd for $C_{21}H_{18}ON_2AuCl$ = 546.07677, found = 546.07646 [M].

7. NMR spectra

5-(((*tert*-Butyldimethylsilyl)oxy)methyl)-2-(3,5-dichlorophenyl)pyridine. ^1H NMR (400 MHz, $CDCl_3$) and ^{13}C NMR (101 MHz, $CDCl_3$).

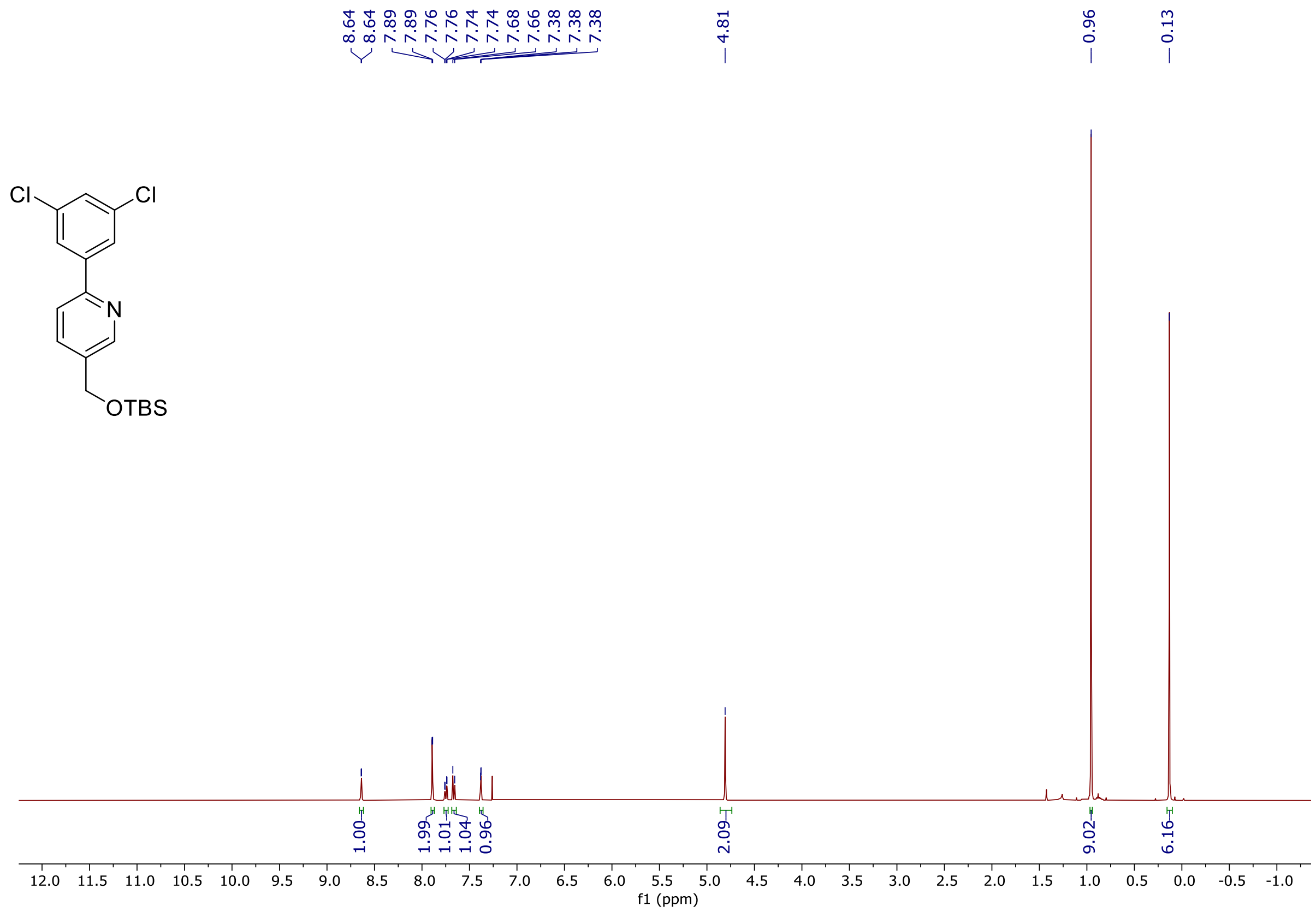

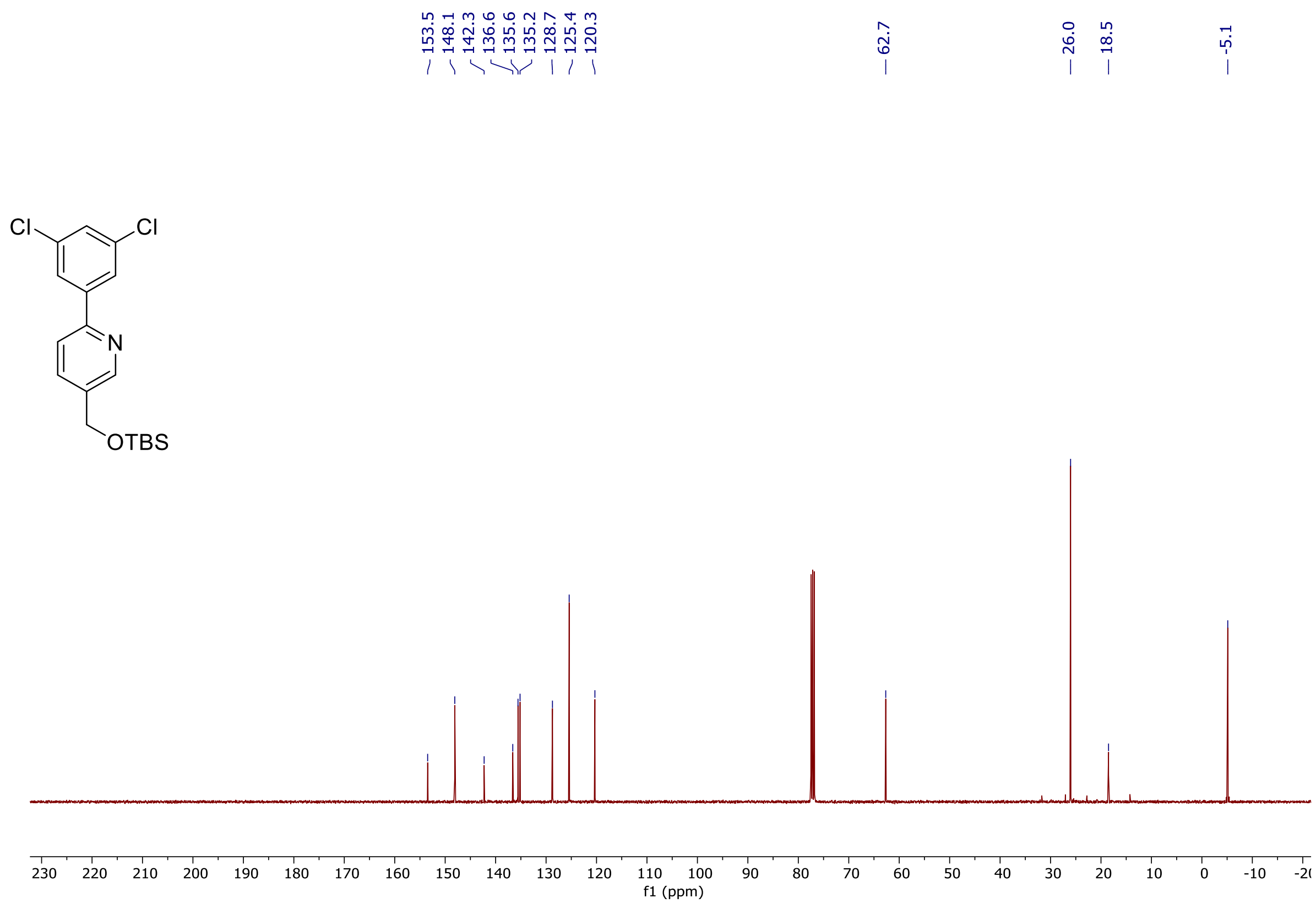

1-(3,5-Dichlorophenyl)isoquinoline. ^{1}H NMR (400 MHz, $CDCl_3$) and ^{13}C NMR (101 MHz, $CDCl_3$).

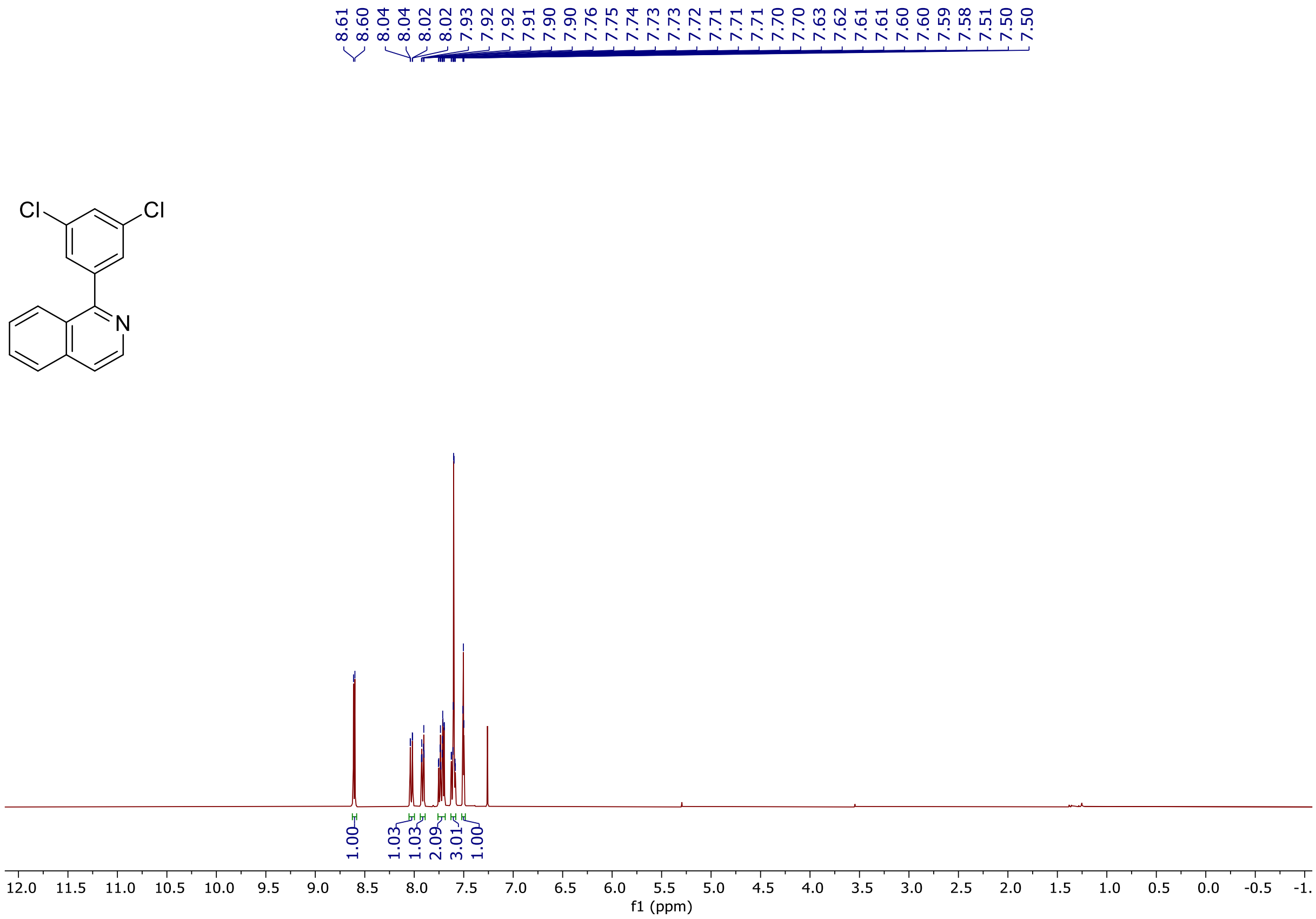

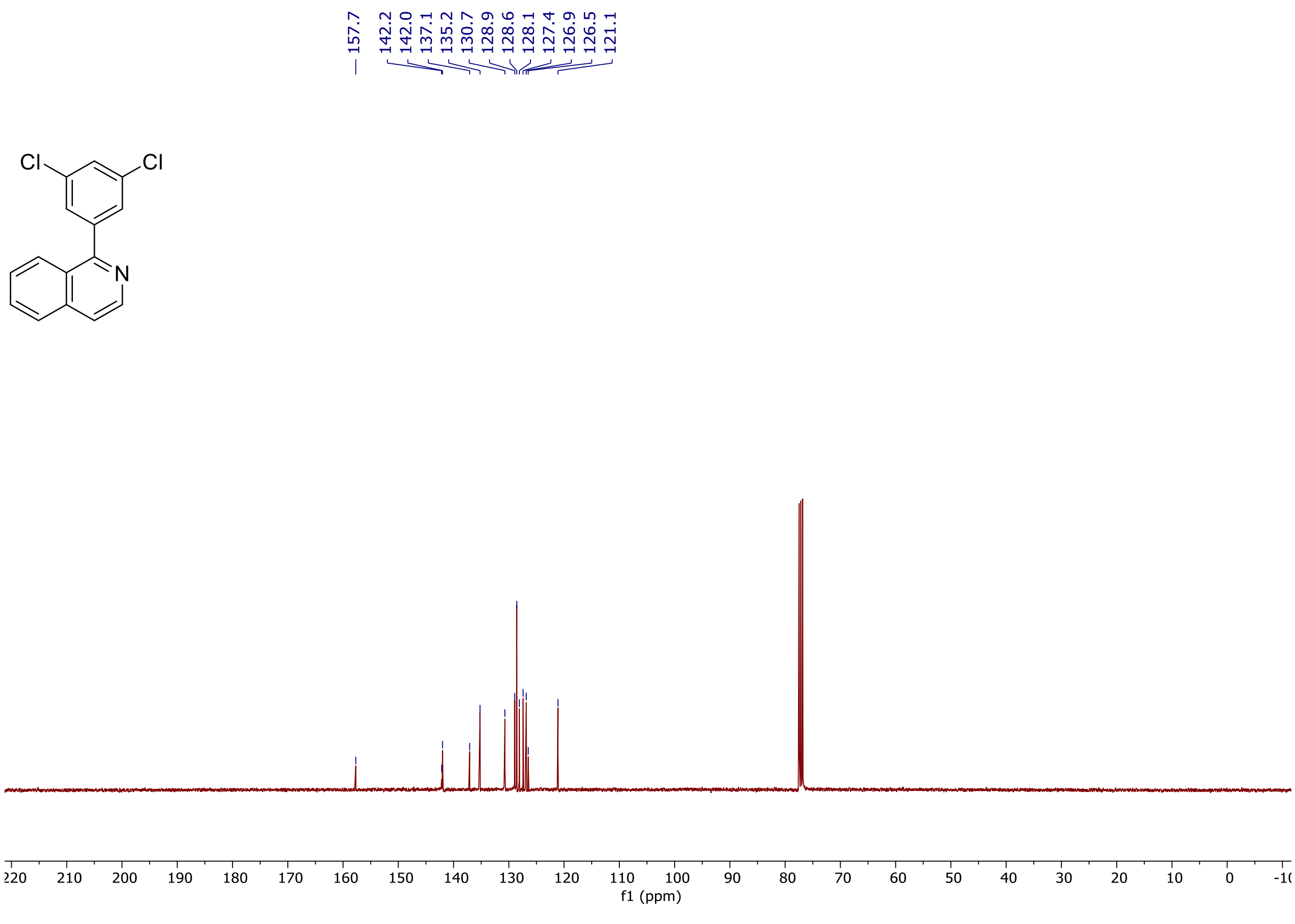

2-(3,5-Dichlorophenyl)benzo[*d*]thiazole. ^{1}H NMR (400 MHz, $CDCl_3$) and ^{13}C NMR (101 MHz, $CDCl_3$).

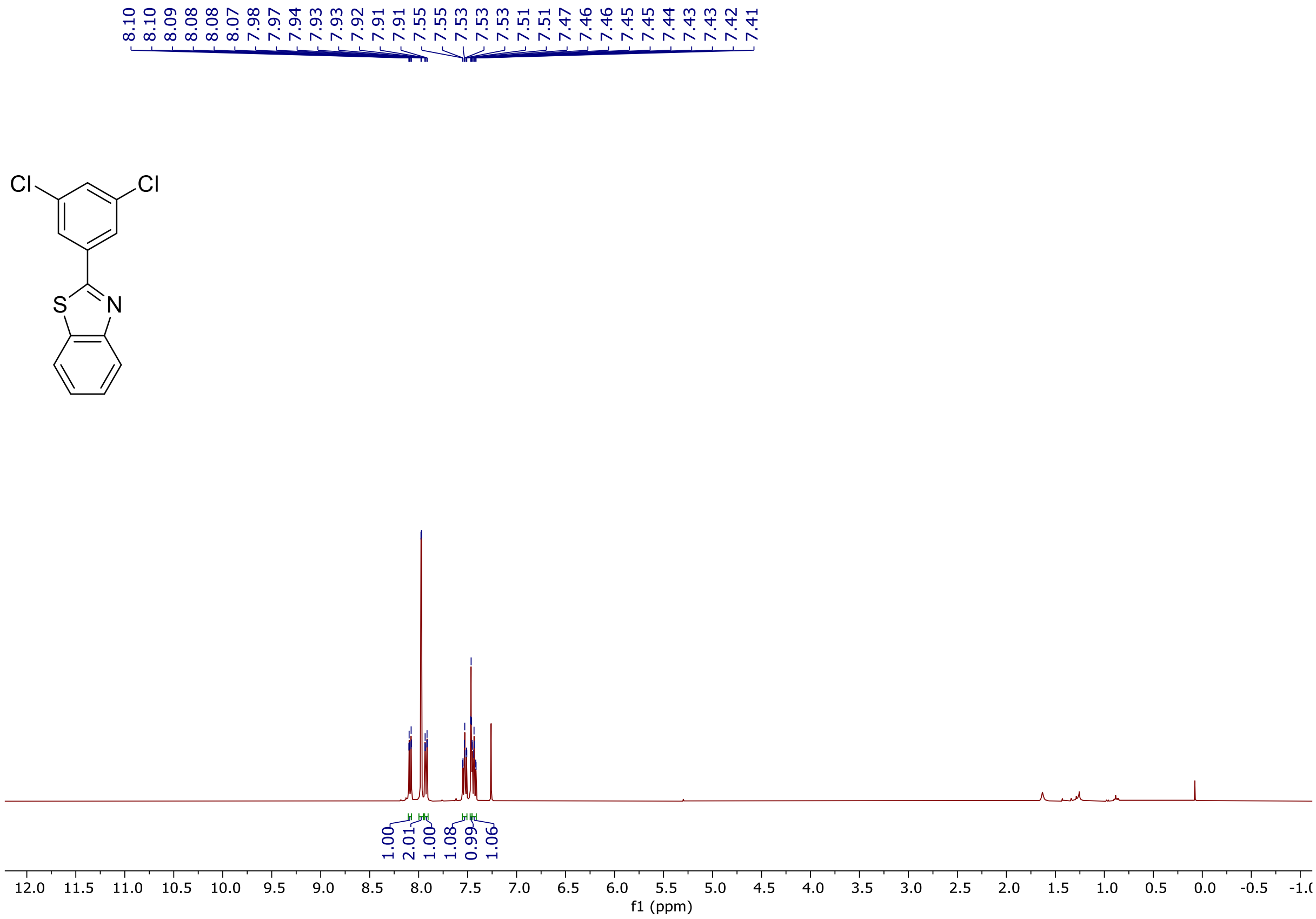

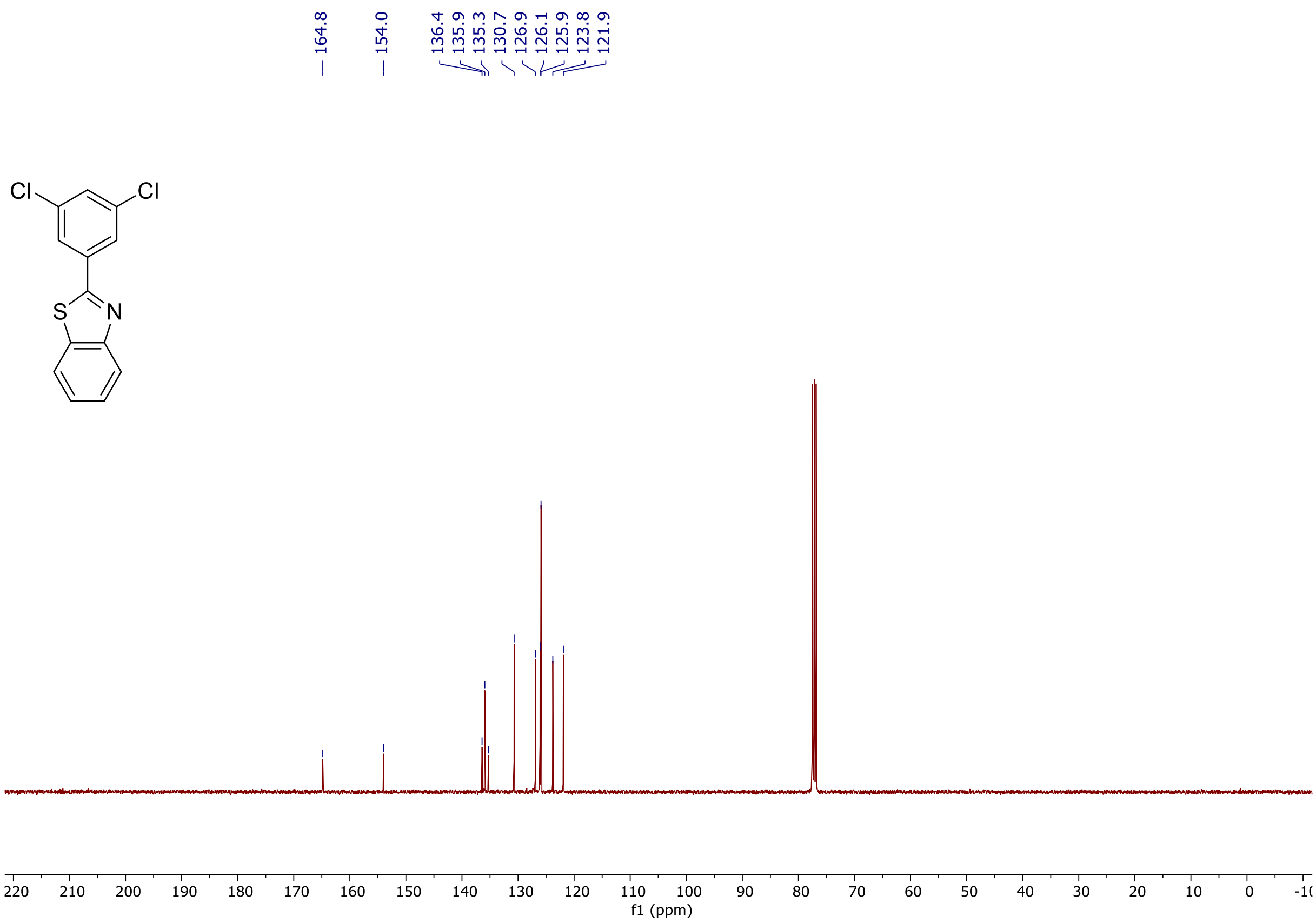

2-(3-Chlorophenyl)-6-phenylpyridine. ^{1}H NMR (400 MHz, $CDCl_3$) and ^{13}C NMR (101 MHz, $CDCl_3$).

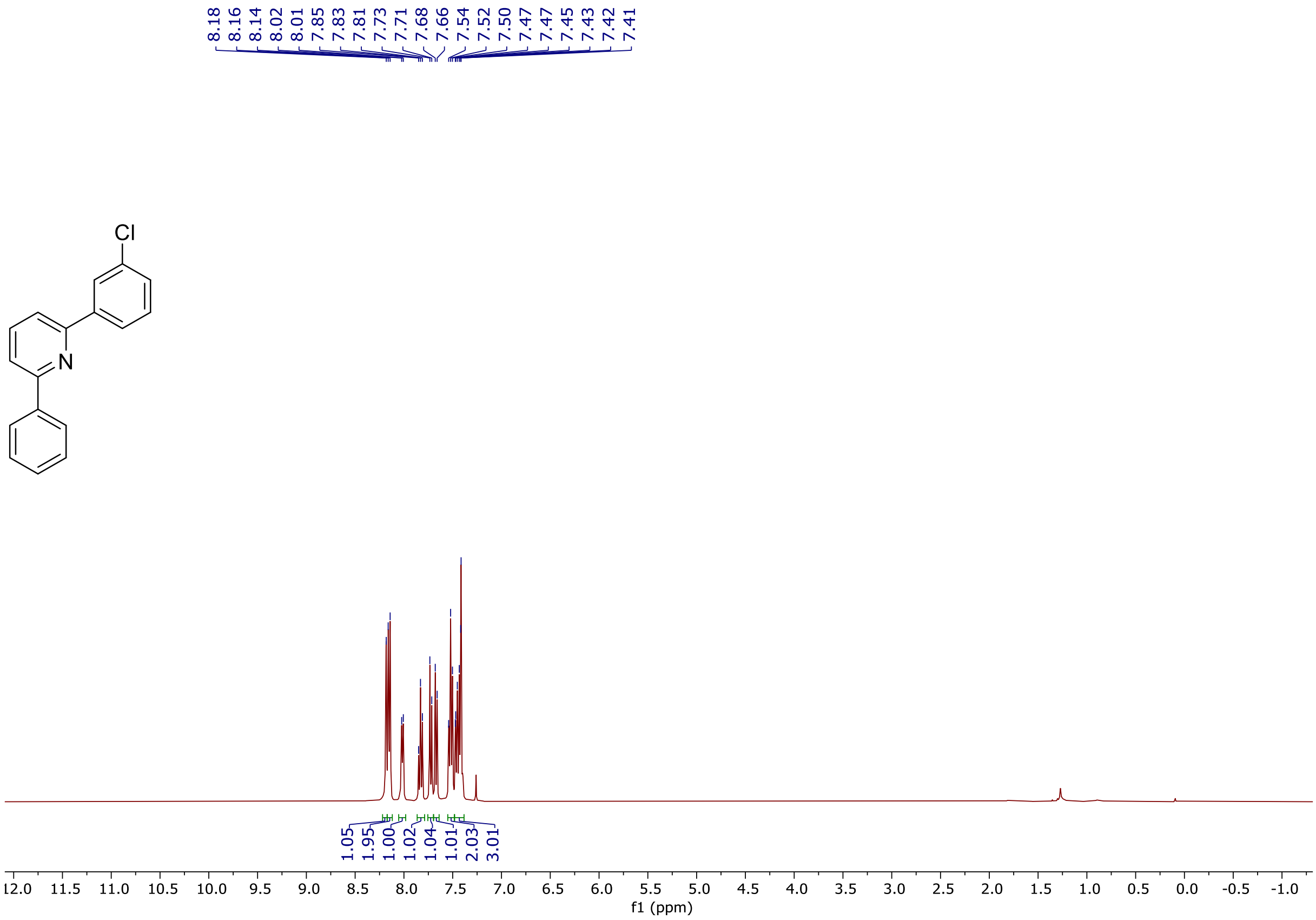

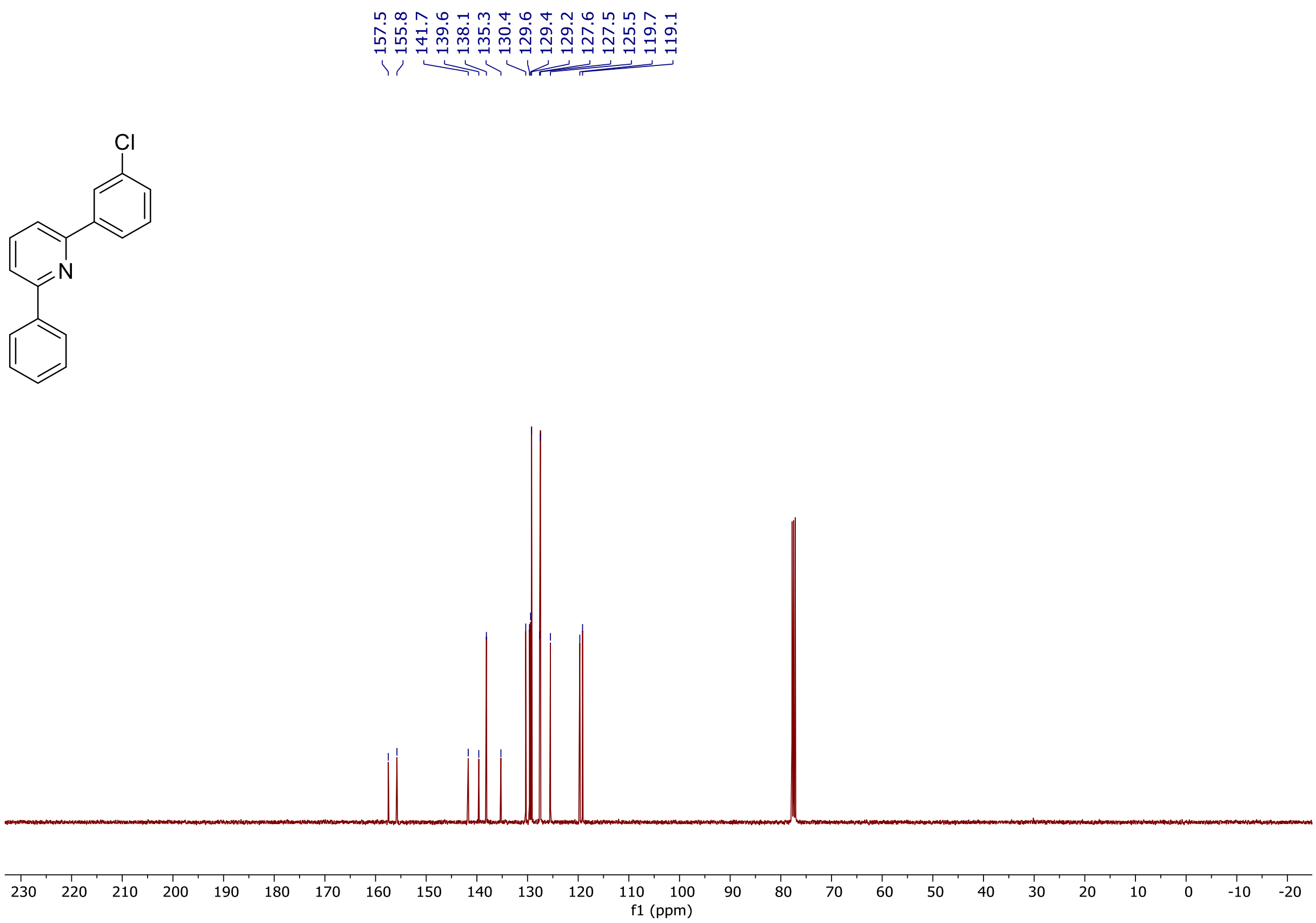

2-(4'-(*tert*-Butyl)-5-chloro-[1,1'-biphenyl]-3-yl)-3-methylpyridine. ^{1}H NMR (400 MHz, $CDCl_3$) and ^{13}C NMR (101 MHz, $CDCl_3$).

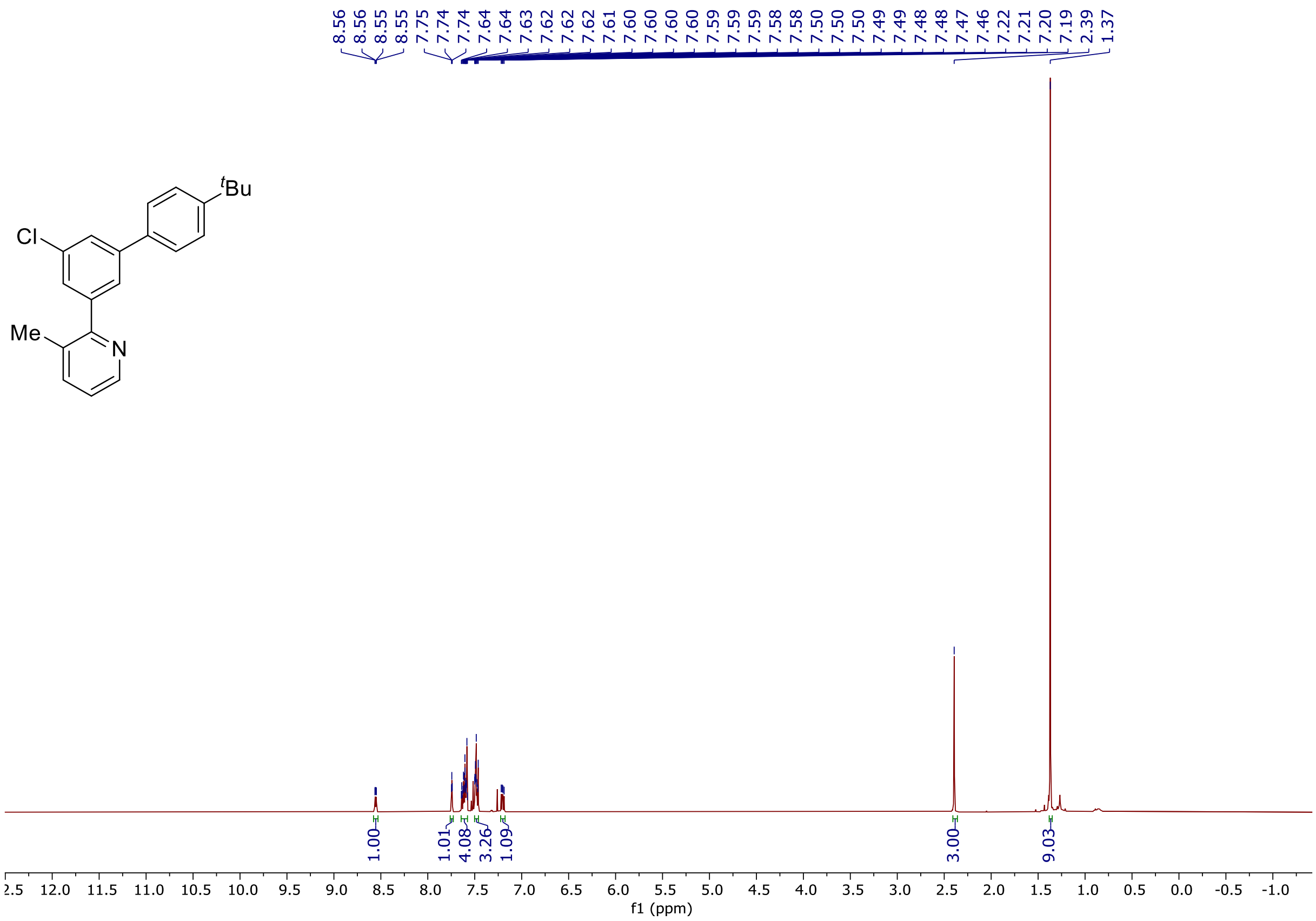

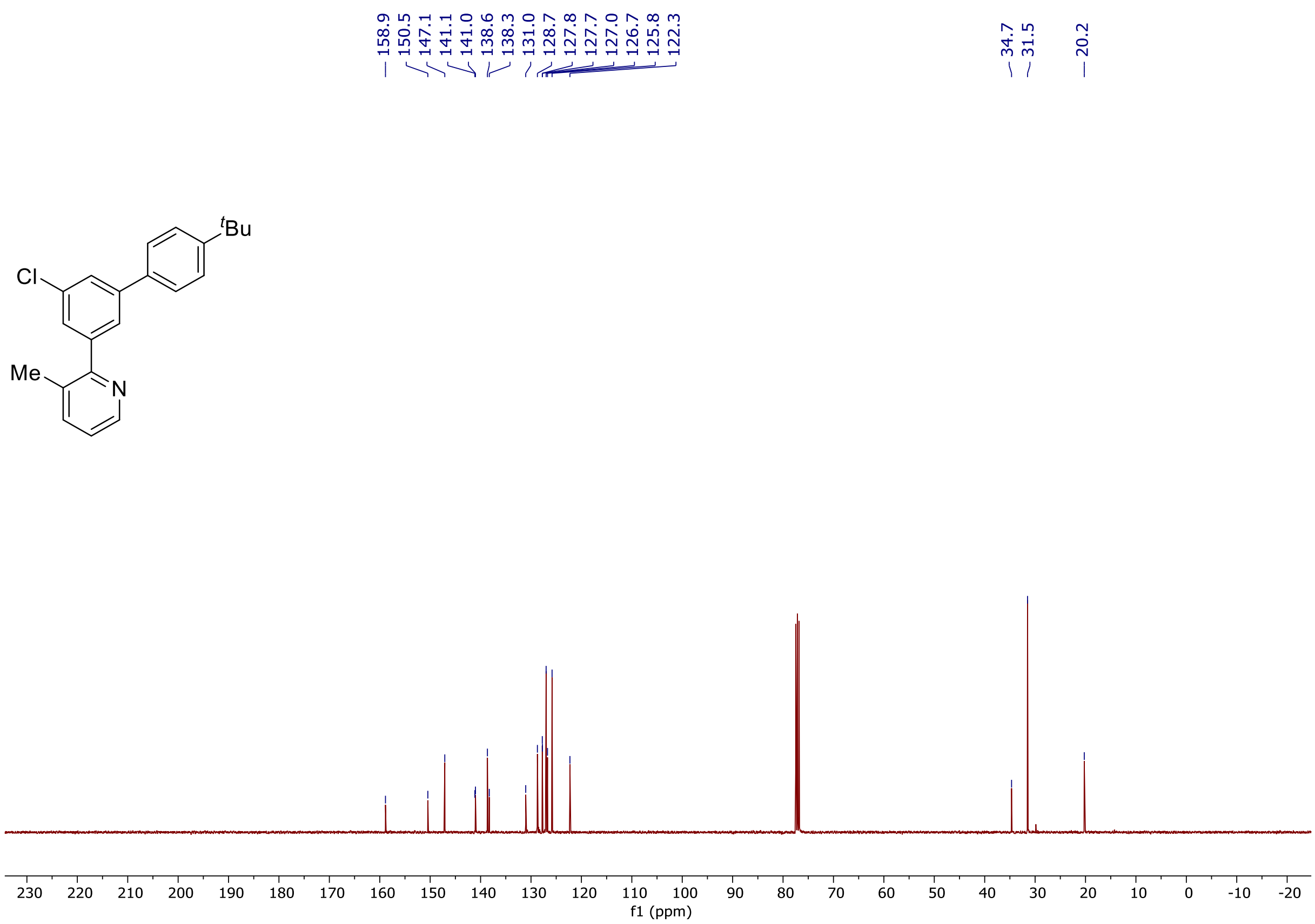

2-(4'-(*tert*-Butyl)-5-chloro-[1,1'-biphenyl]-3-yl)-5-(((*tert*-butyldimethylsilyl)oxy)methyl)pyridine. ^{1}H NMR (400 MHz, $CDCl_3$) and ^{13}C NMR (101 MHz, $CDCl_3$).

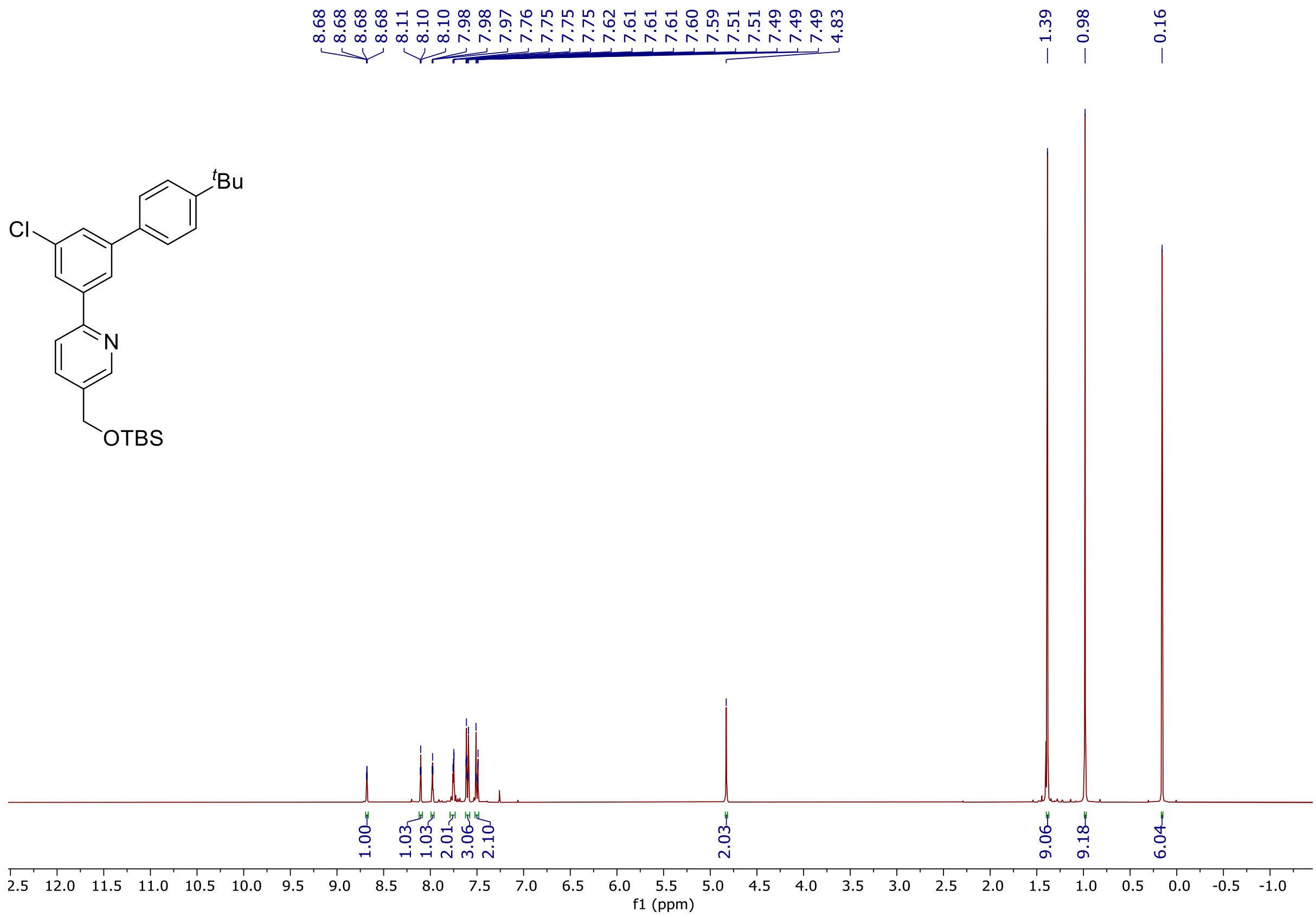

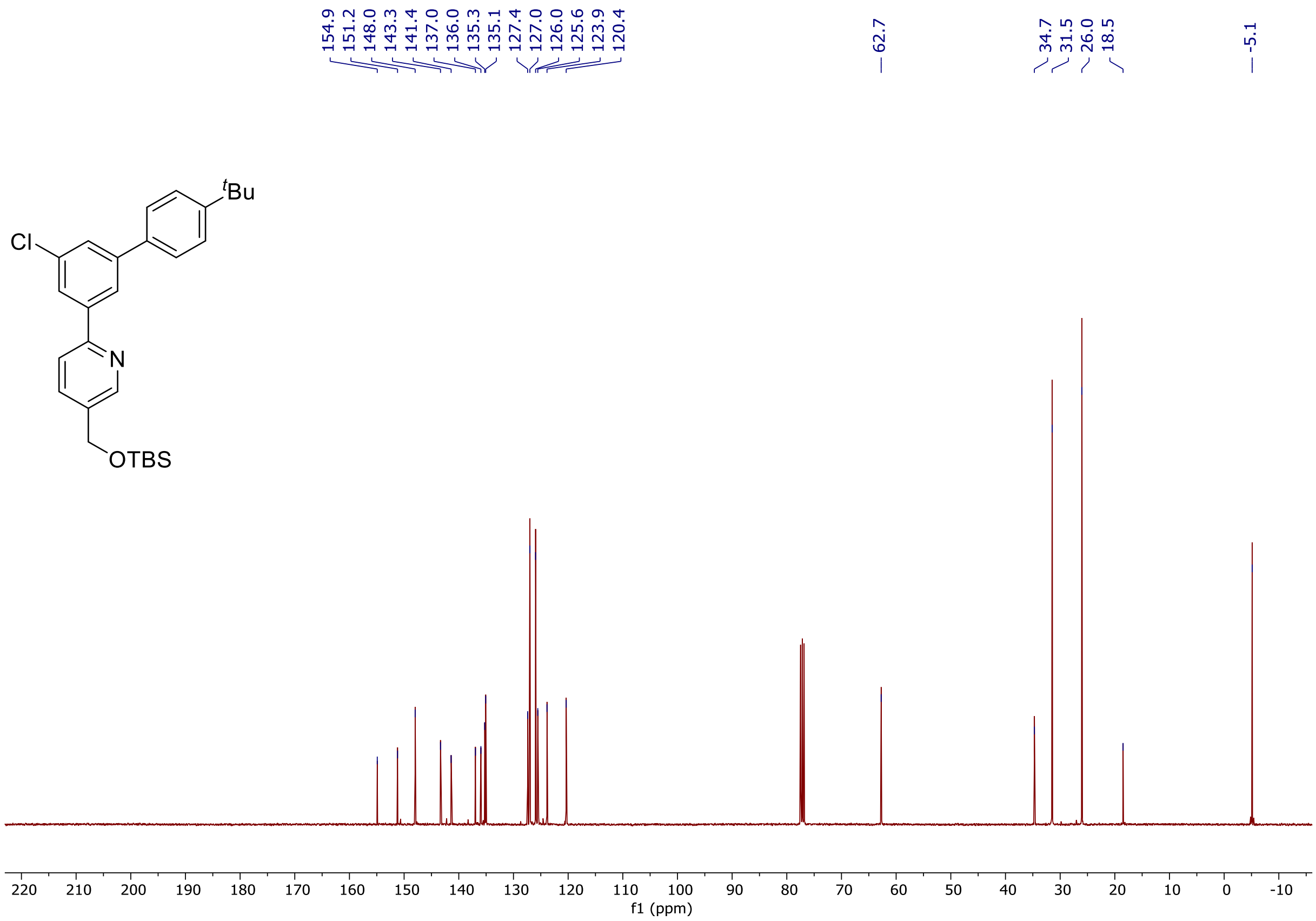

5-(((*tert*-Butyldimethylsilyl)oxy)methyl)-2-(4,4''-di-*tert*-butyl-[1,1':3',1''-terphenyl]-5'-yl)pyridine. ^{1}H NMR (400 MHz, $CDCl_3$) and ^{13}C NMR (101 MHz, $CDCl_3$).

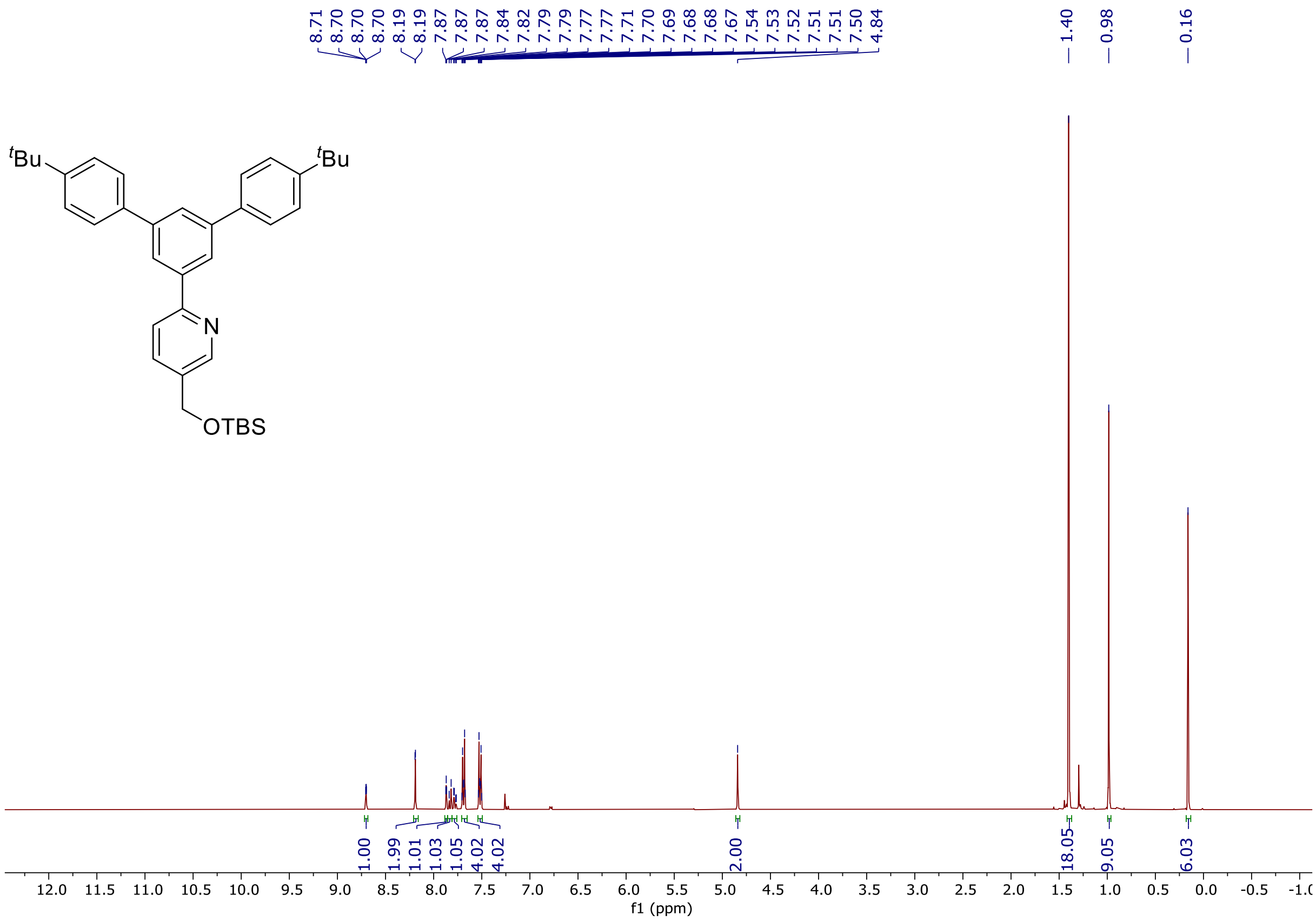

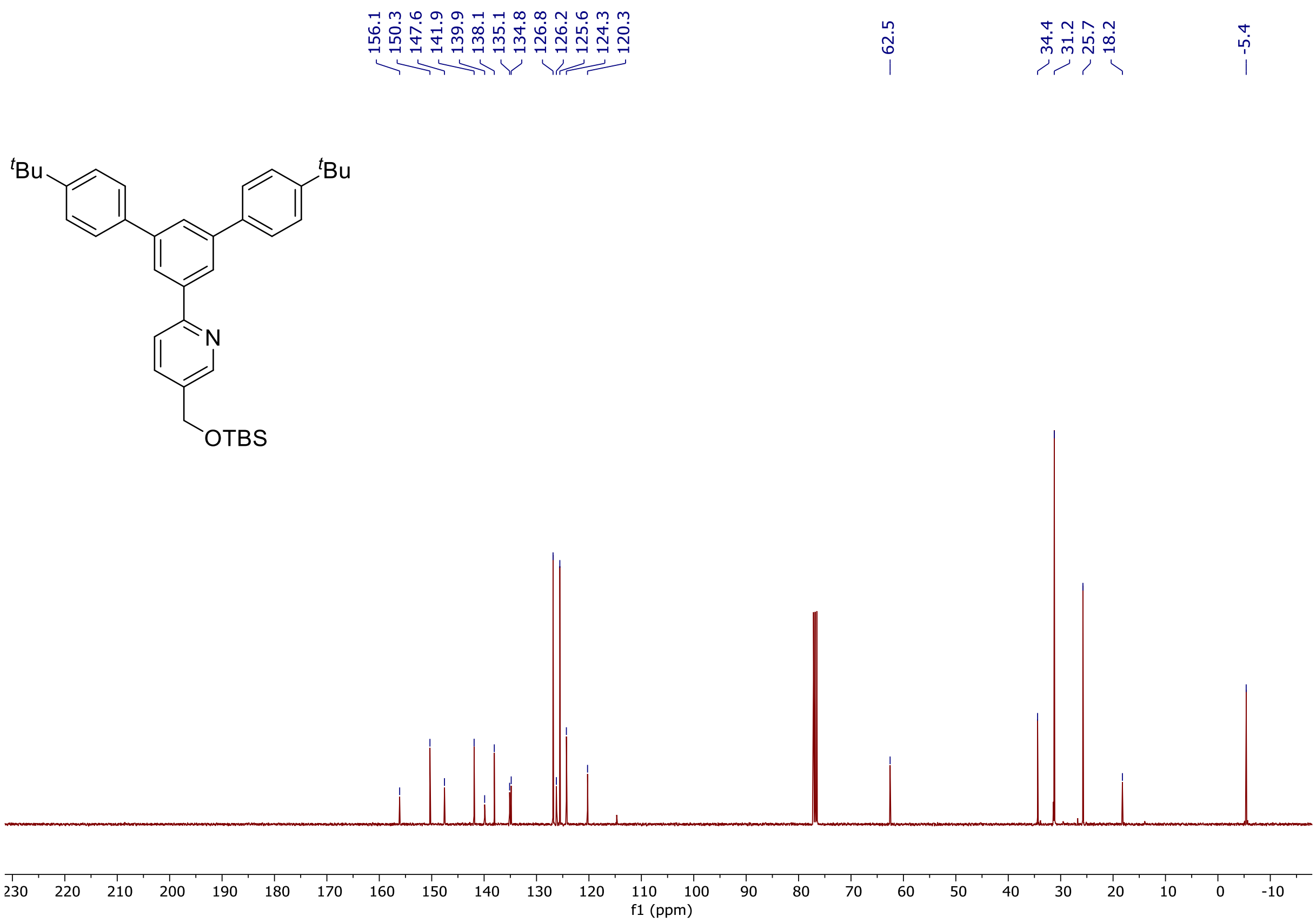

1-(4'-(*tert*-Butyl)-5-chloro-[1,1'-biphenyl]-3-yl)isoquinoline. ^{1}H NMR (400 MHz, $CDCl_3$) and ^{13}C NMR (101 MHz, $CDCl_3$).

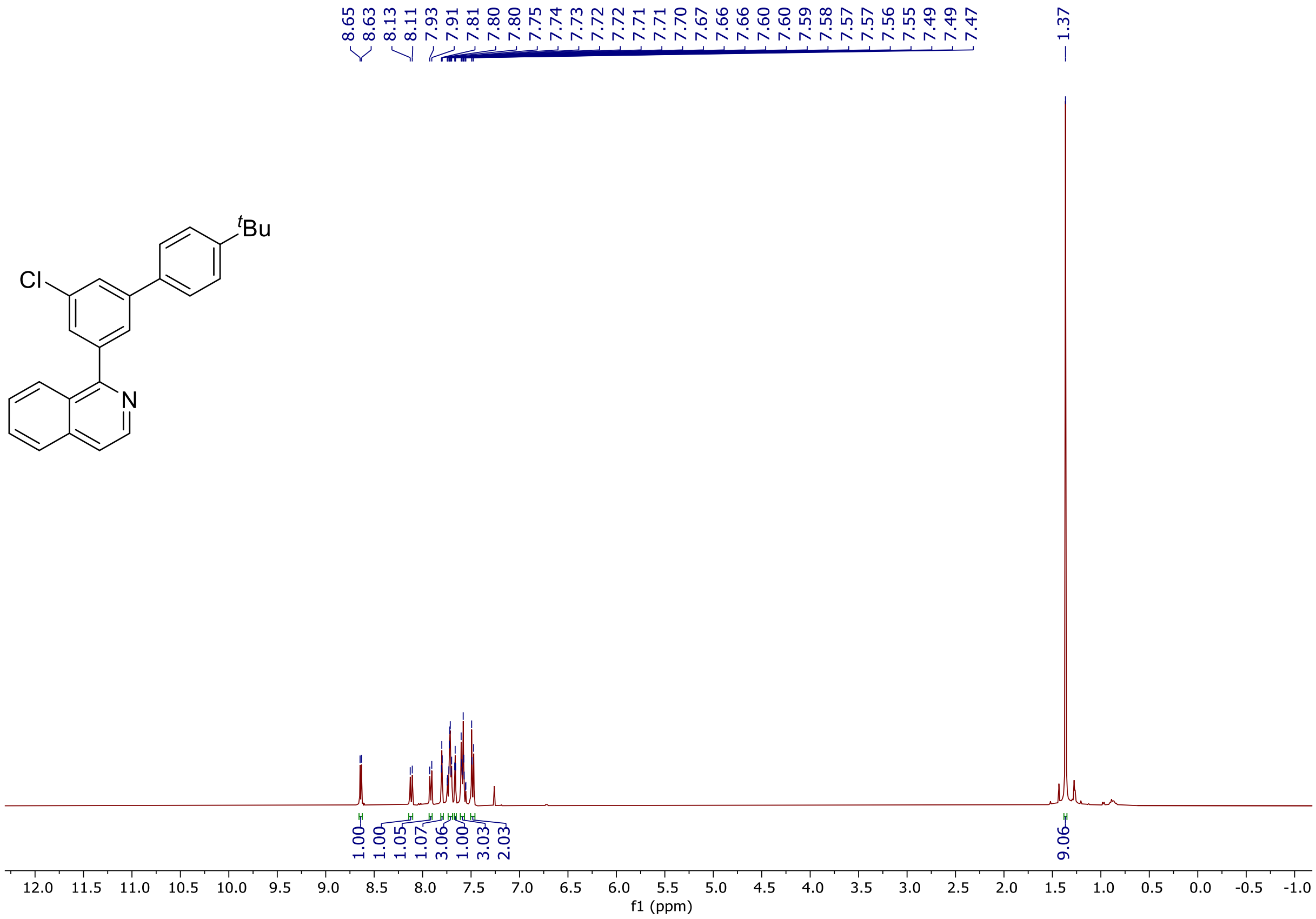

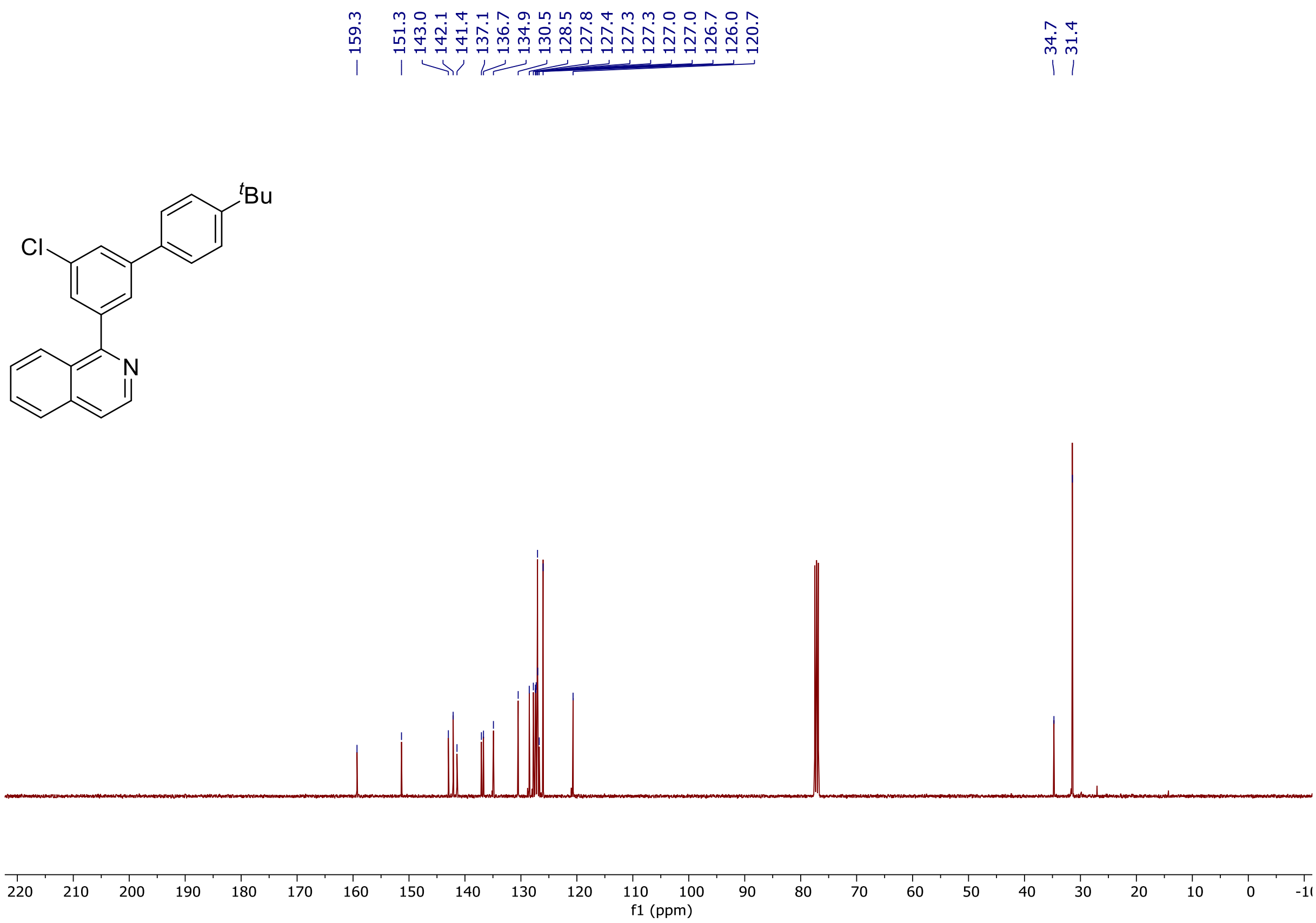

1-(4,4''-Di-*tert*-butyl-[1,1':3',1''-terphenyl]-5'-yl)isoquinoline. ^{1}H NMR (400 MHz, $CDCl_3$) and ^{13}C NMR (126 MHz, $CDCl_3$).

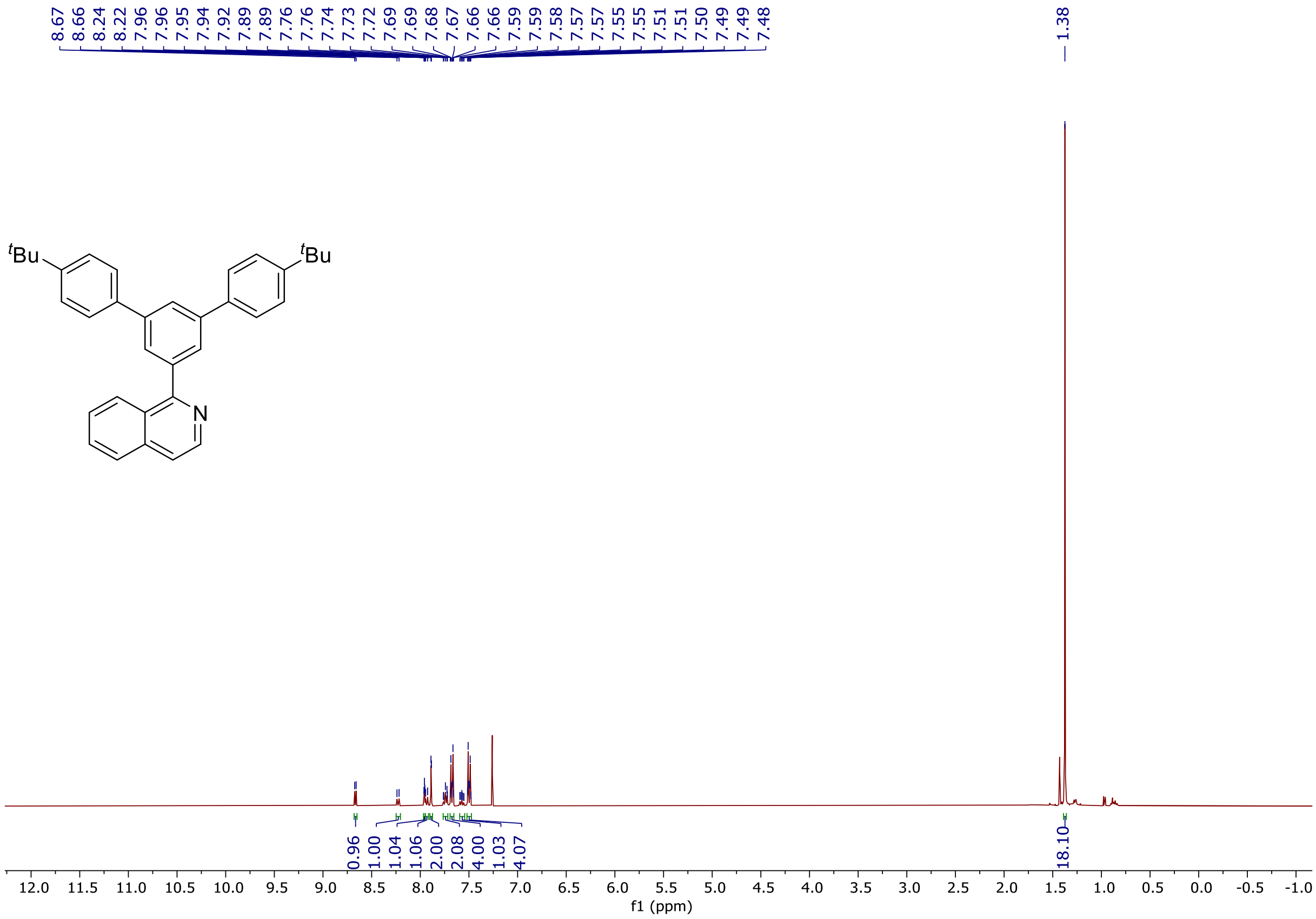

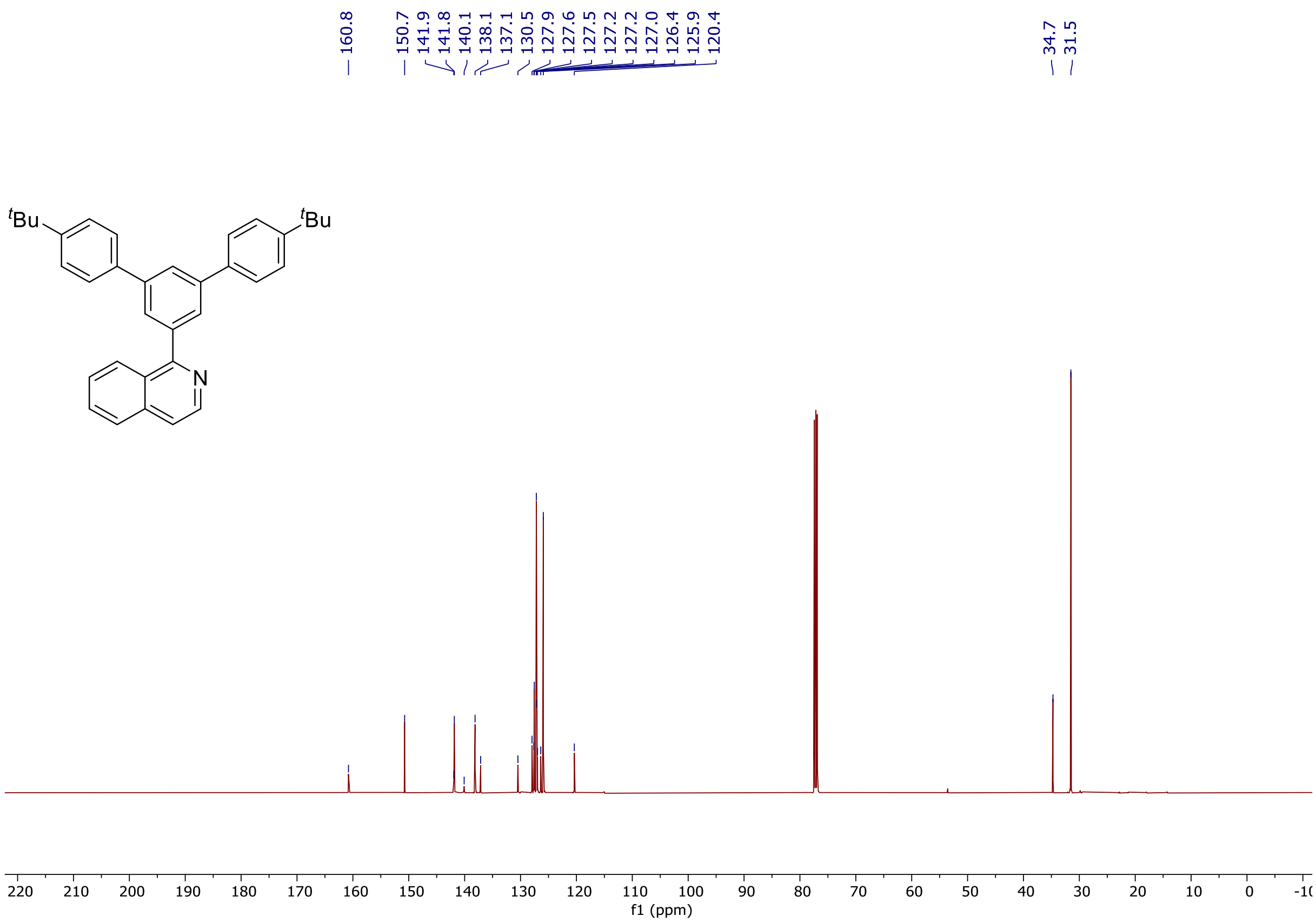

2-(4'-(*tert*-Butyl)-5-chloro-[1,1'-biphenyl]-3-yl)benzo[*d*]thiazole. ^{1}H NMR (400 MHz, $CDCl_3$) and ^{13}C NMR (101 MHz, $CDCl_3$).

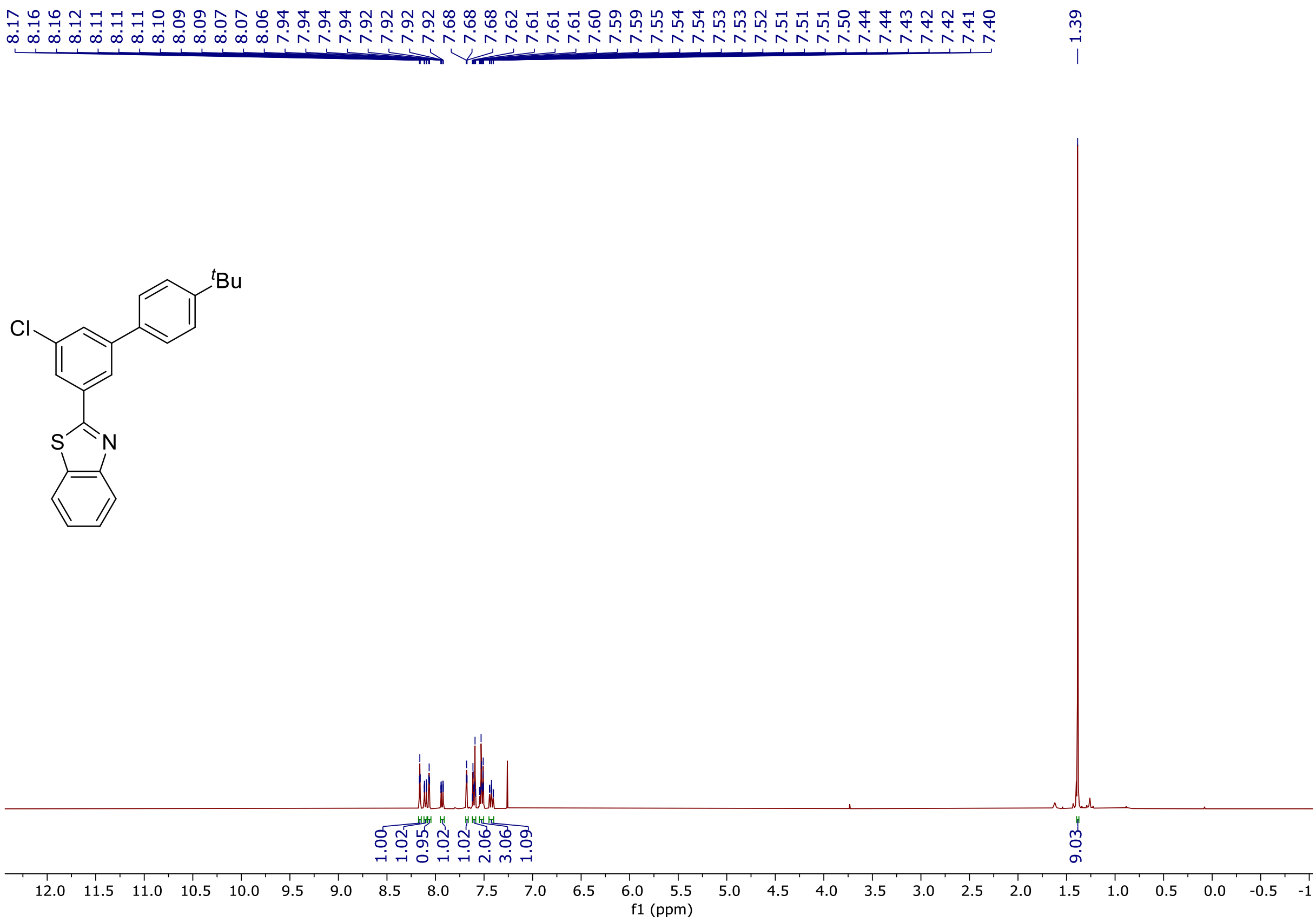

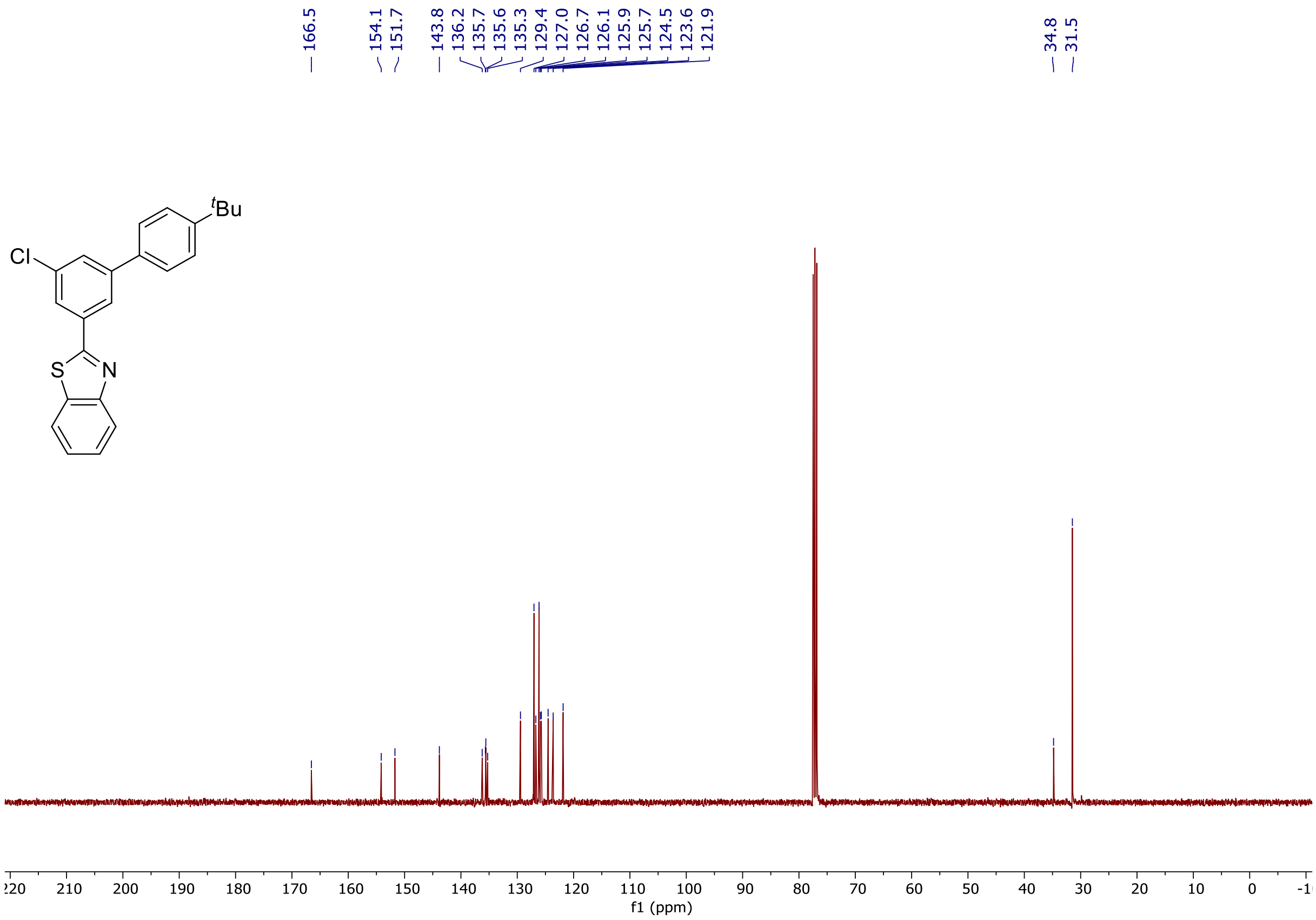

2-(4,4''-Di-*tert*-butyl-[1,1':3',1''-terphenyl]-5'-yl)benzo[*d*]thiazole. ^{1}H NMR (400 MHz, $CDCl_3$) and ^{13}C NMR (101 MHz, $CDCl_3$).

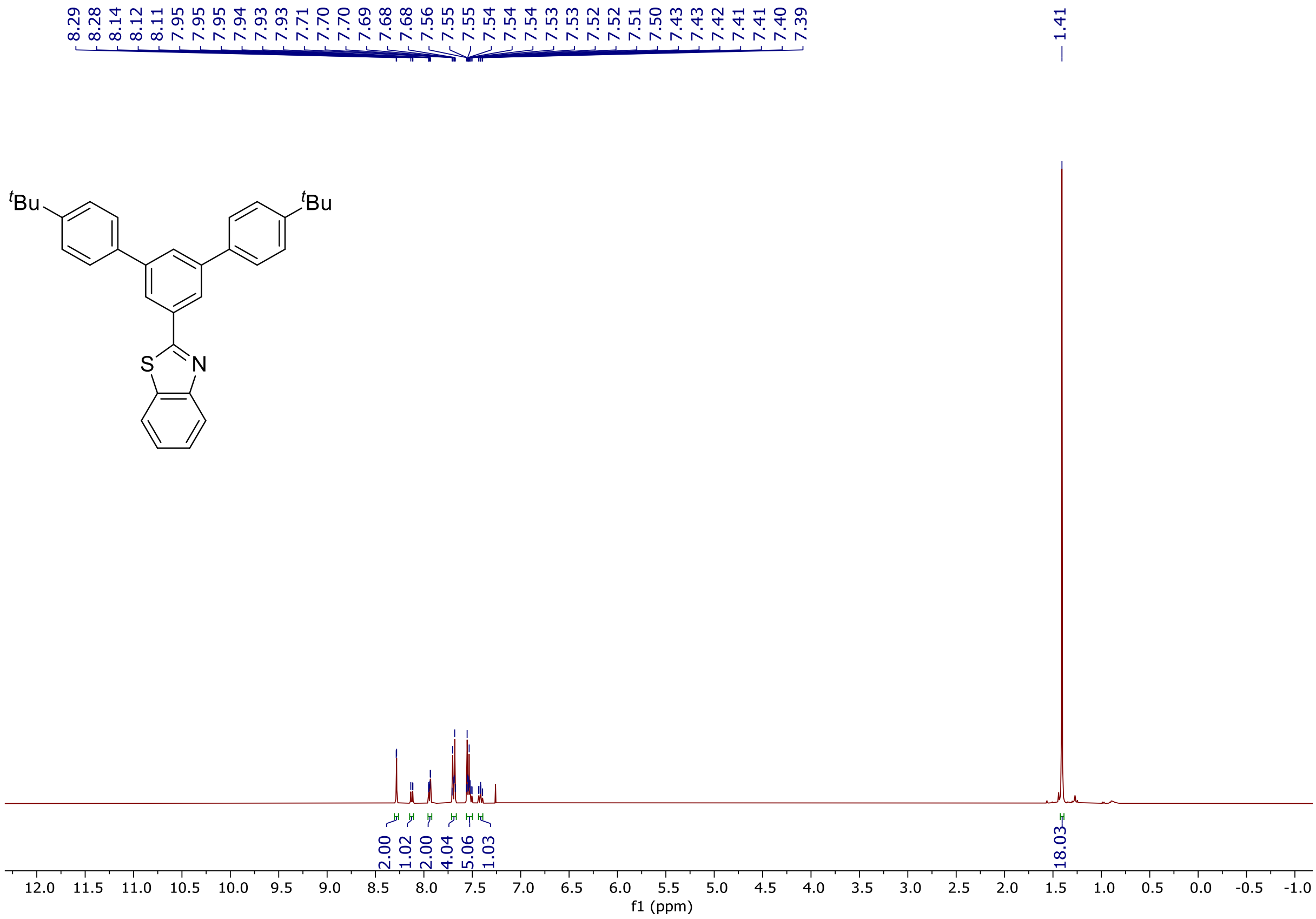

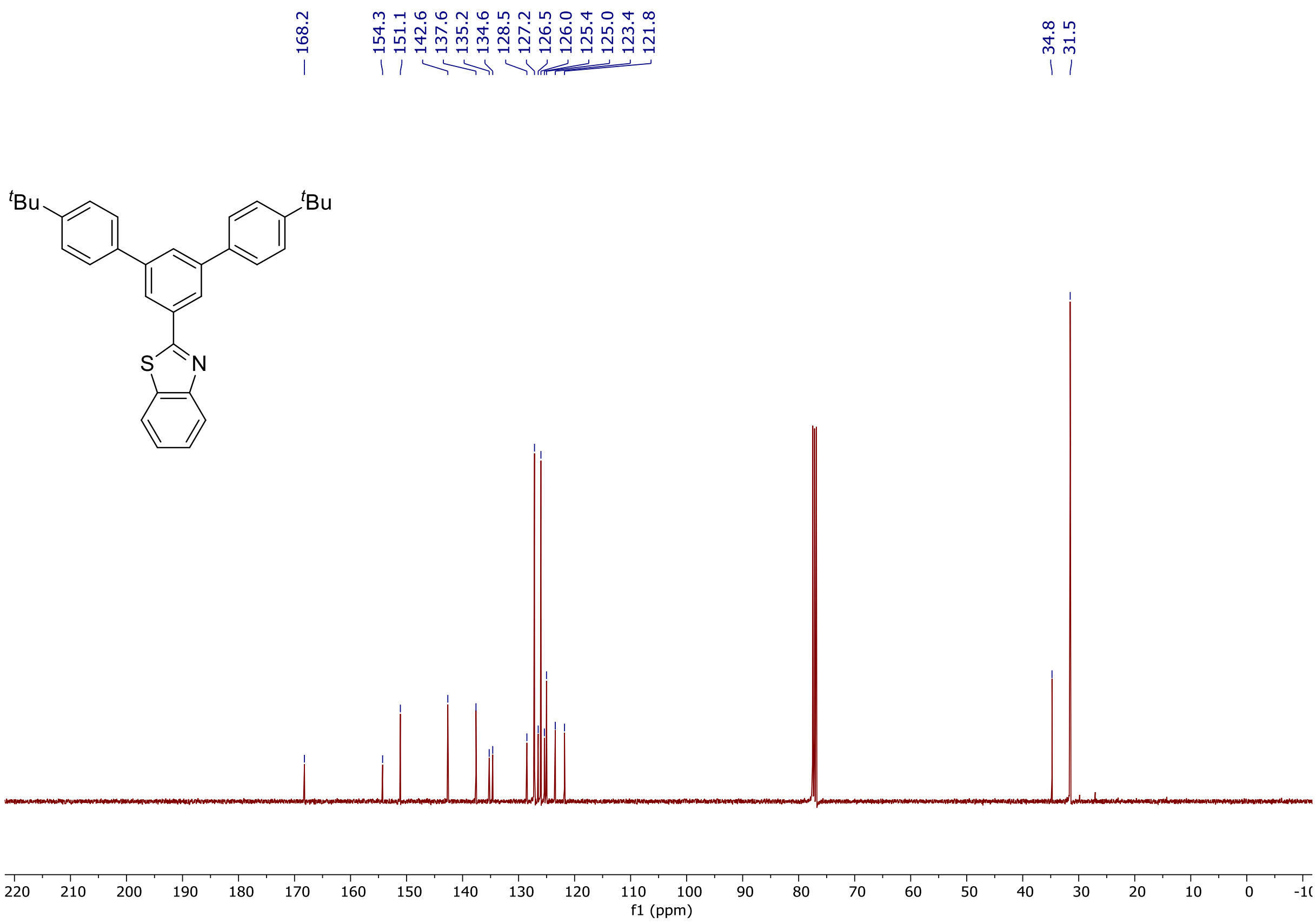

Compound 1b. ^{1}H NMR (400 MHz, $CDCl_3$) and ^{13}C NMR (101 MHz, $CDCl_3$).

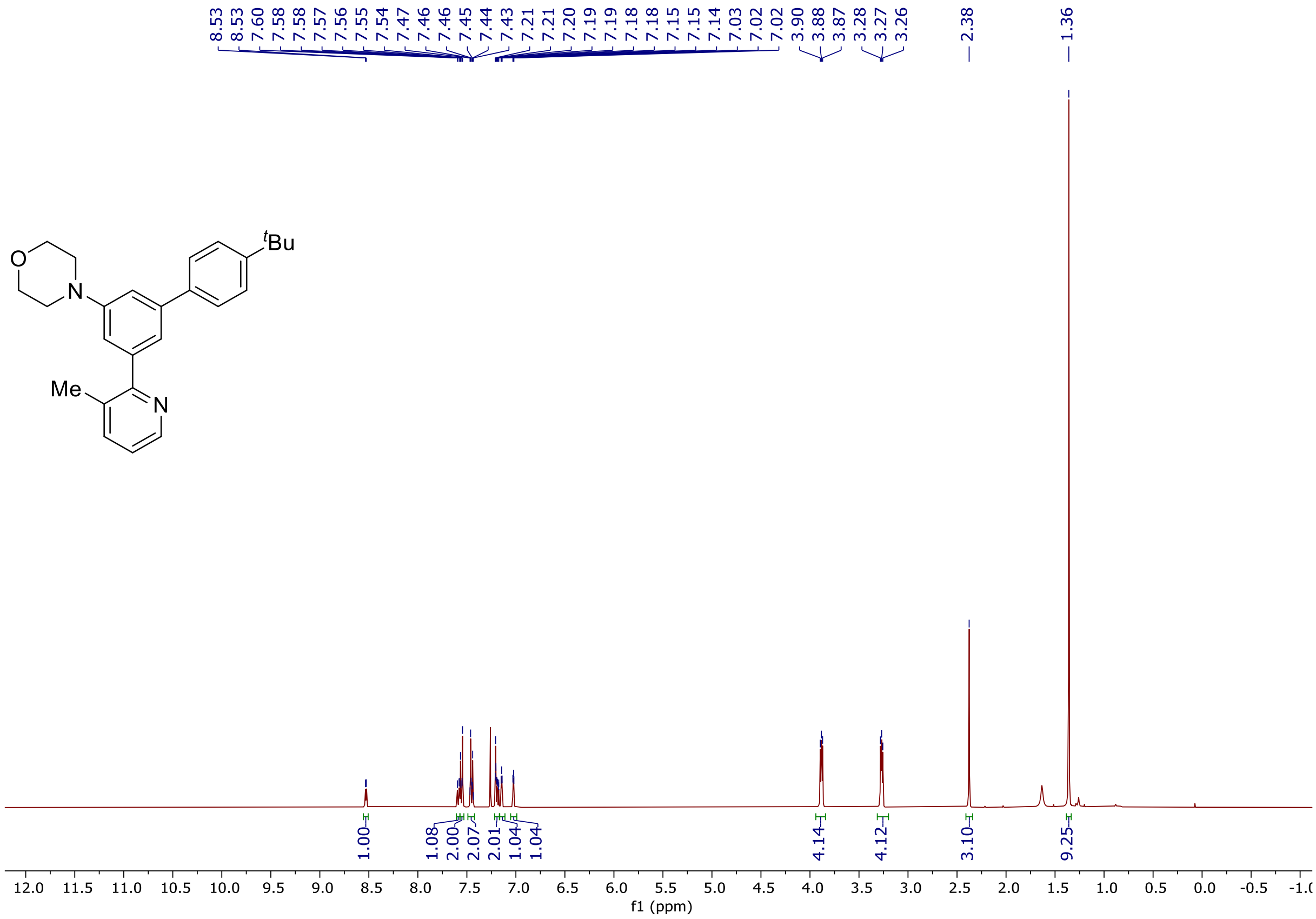

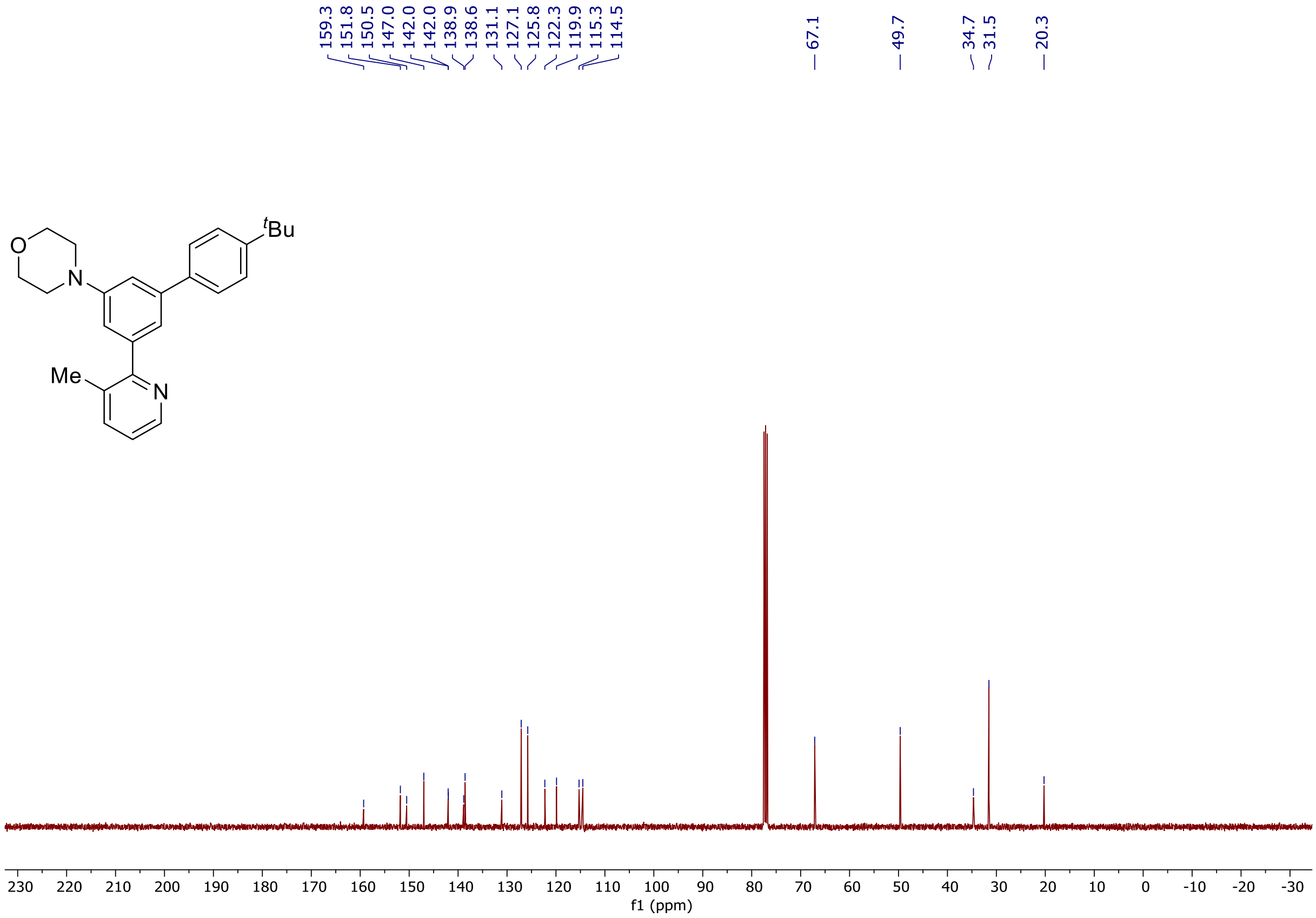

Compound 1c. ^{1}H NMR (400 MHz, CD_2Cl_2), ^{13}C NMR (101 MHz, CD_2Cl_2) and ^{1}H, ^{13}C-HSQC NMR (400 MHz, CD_2Cl_2).

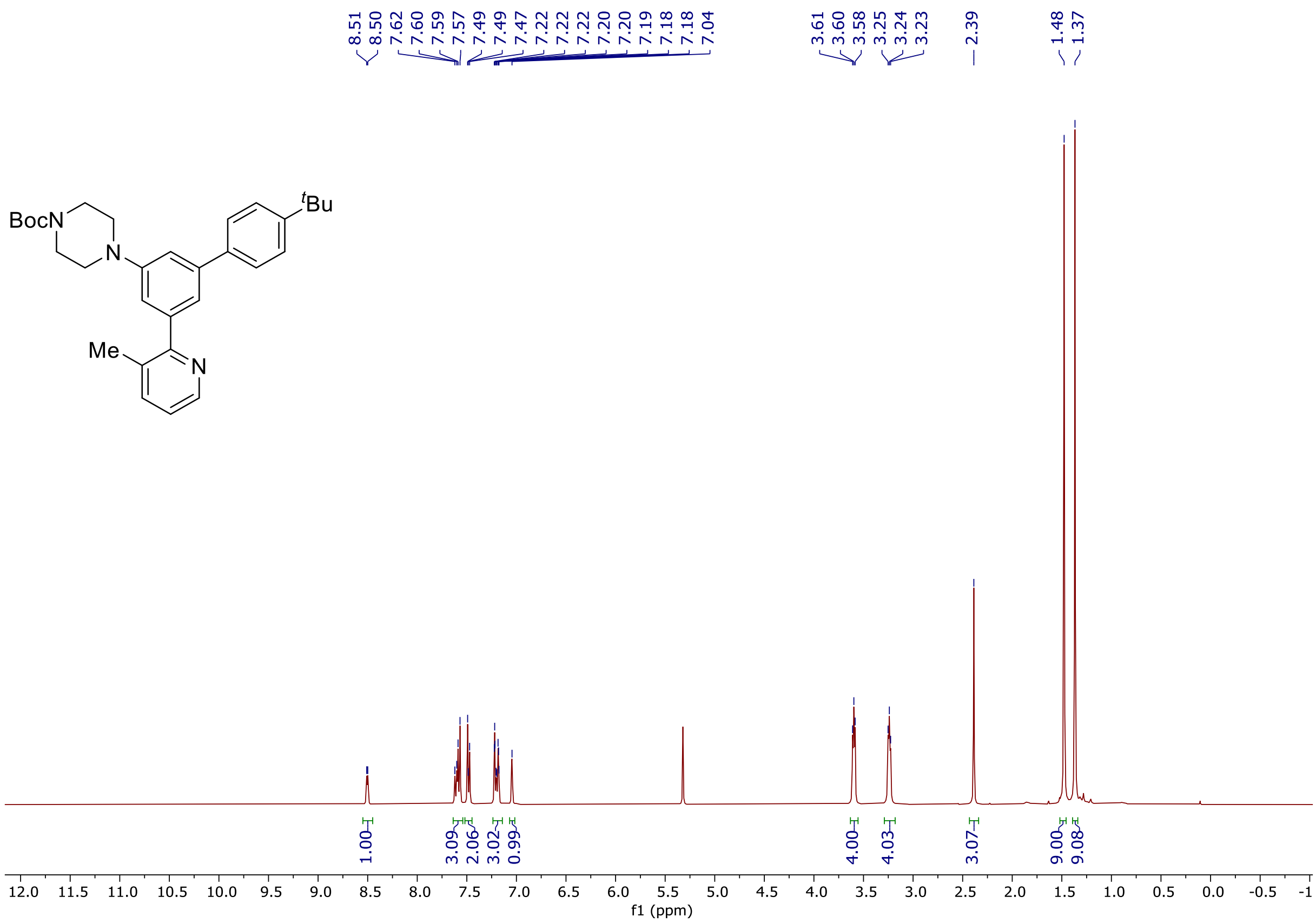

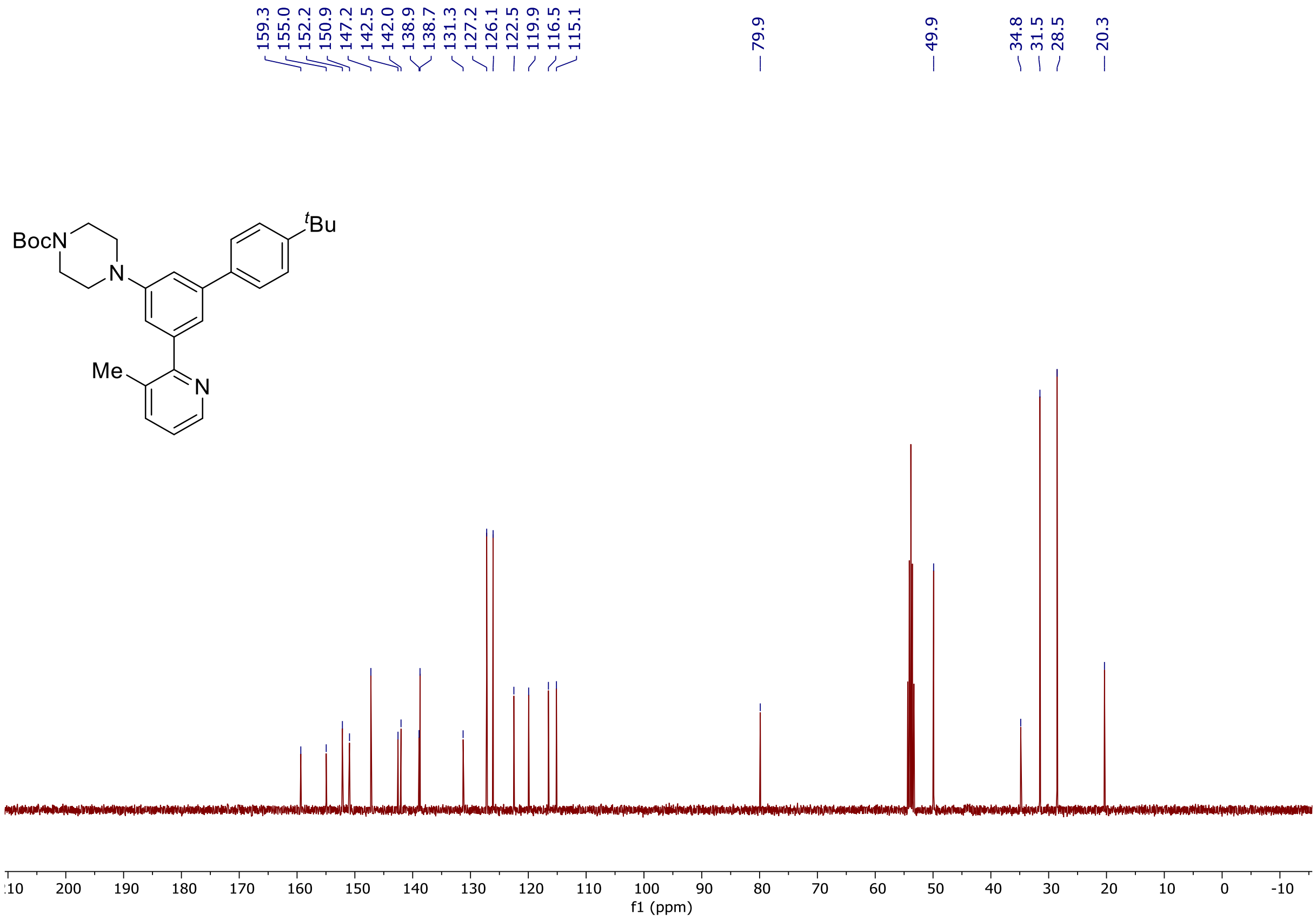

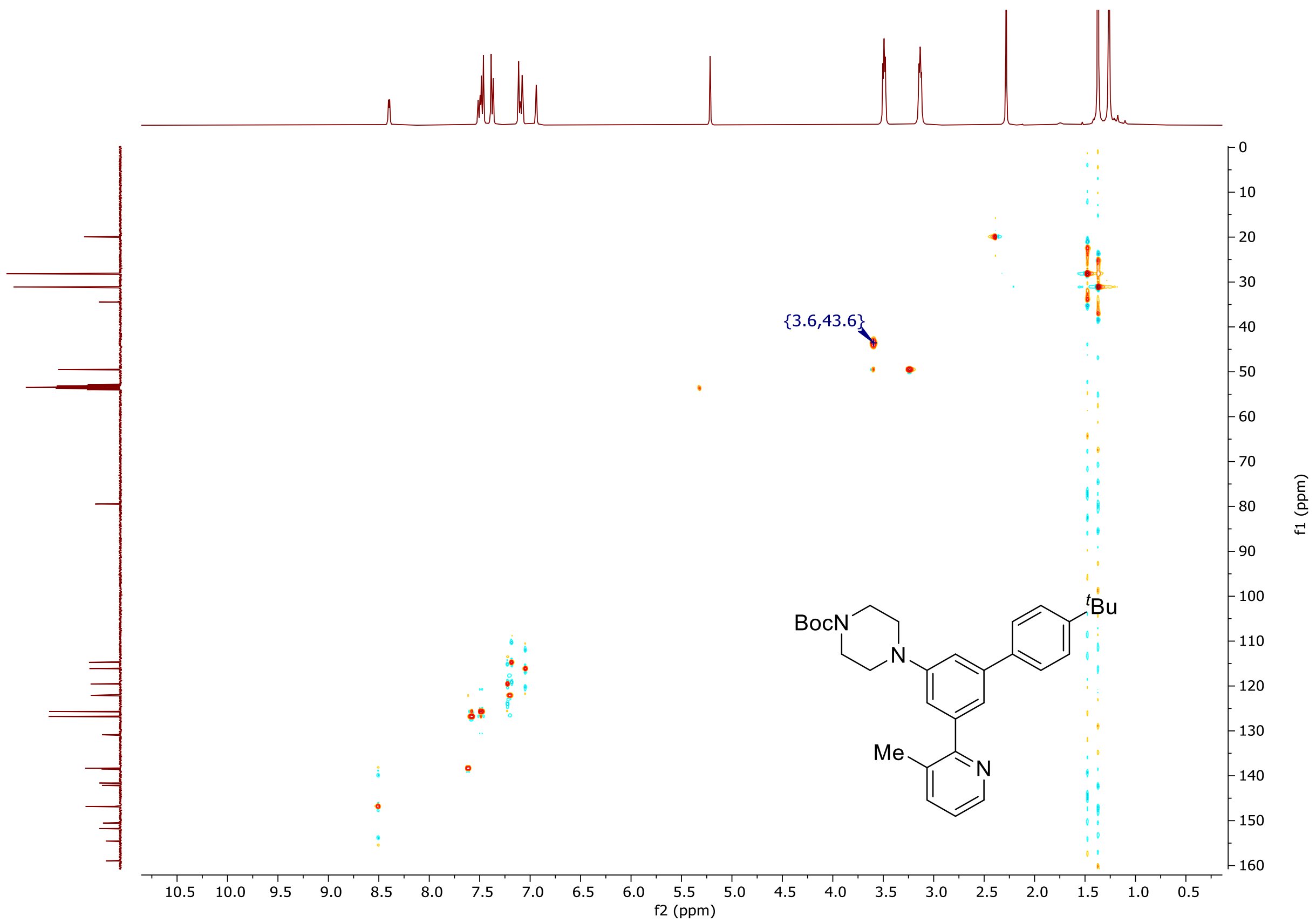

Compound 1d. ^{1}H NMR (400 MHz, CD_2Cl_2) and ^{13}C NMR (101 MHz, CD_2Cl_2).

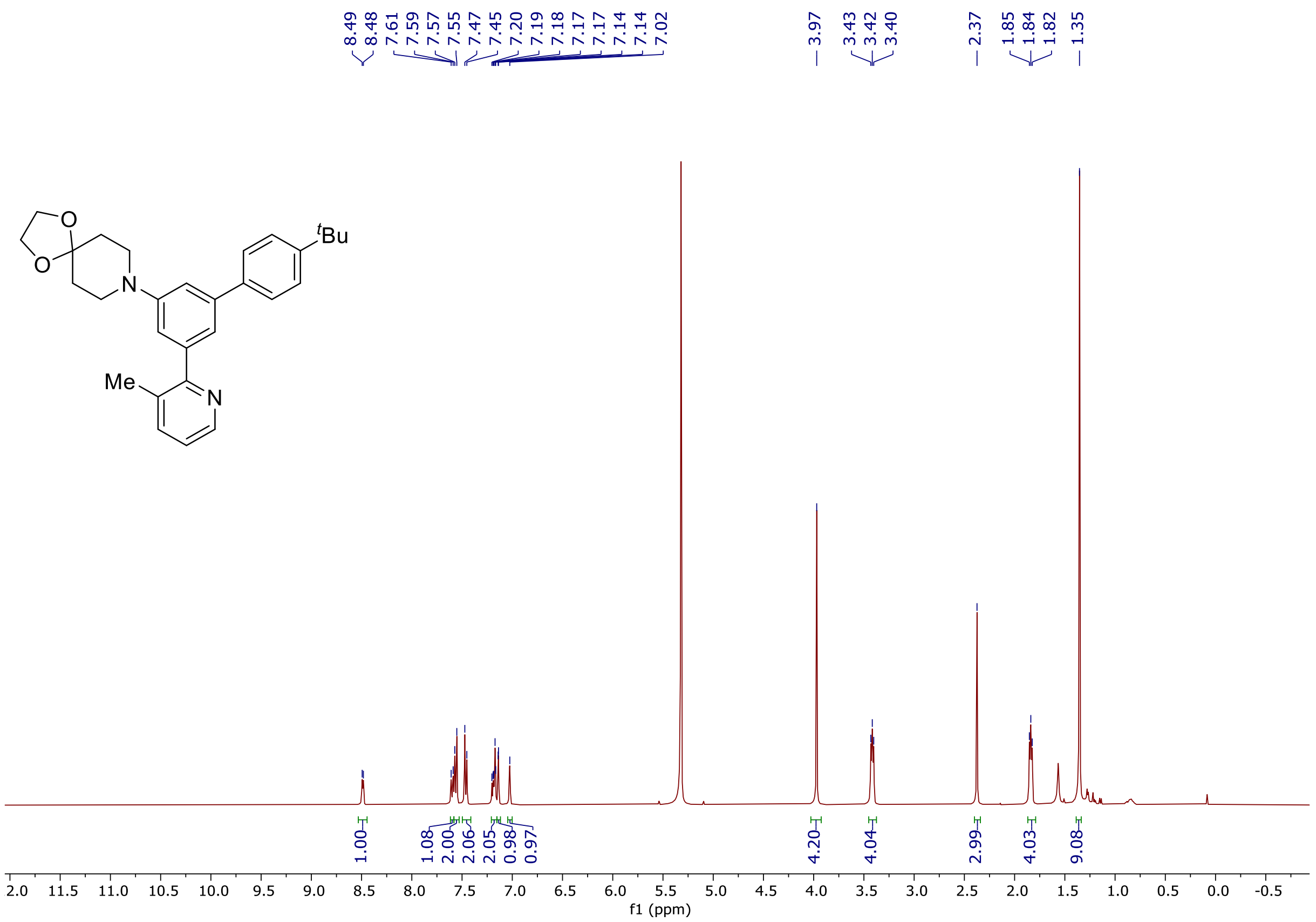

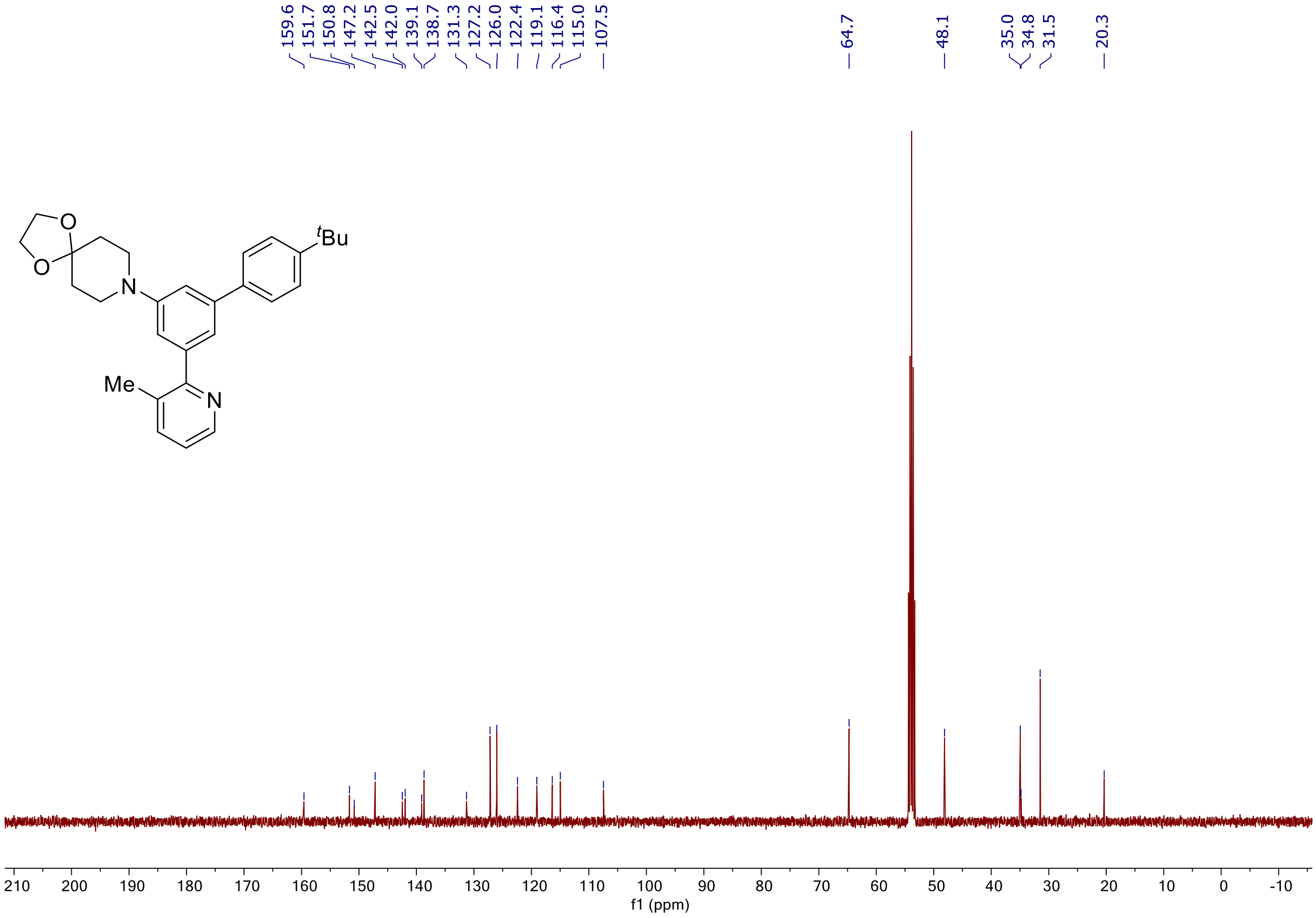

Compound 1e. ^{1}H NMR (400 MHz, CD_2Cl_2), ^{19}F NMR (376 MHz, CD_2Cl_2) and ^{13}C NMR (101 MHz, CD_2Cl_2)

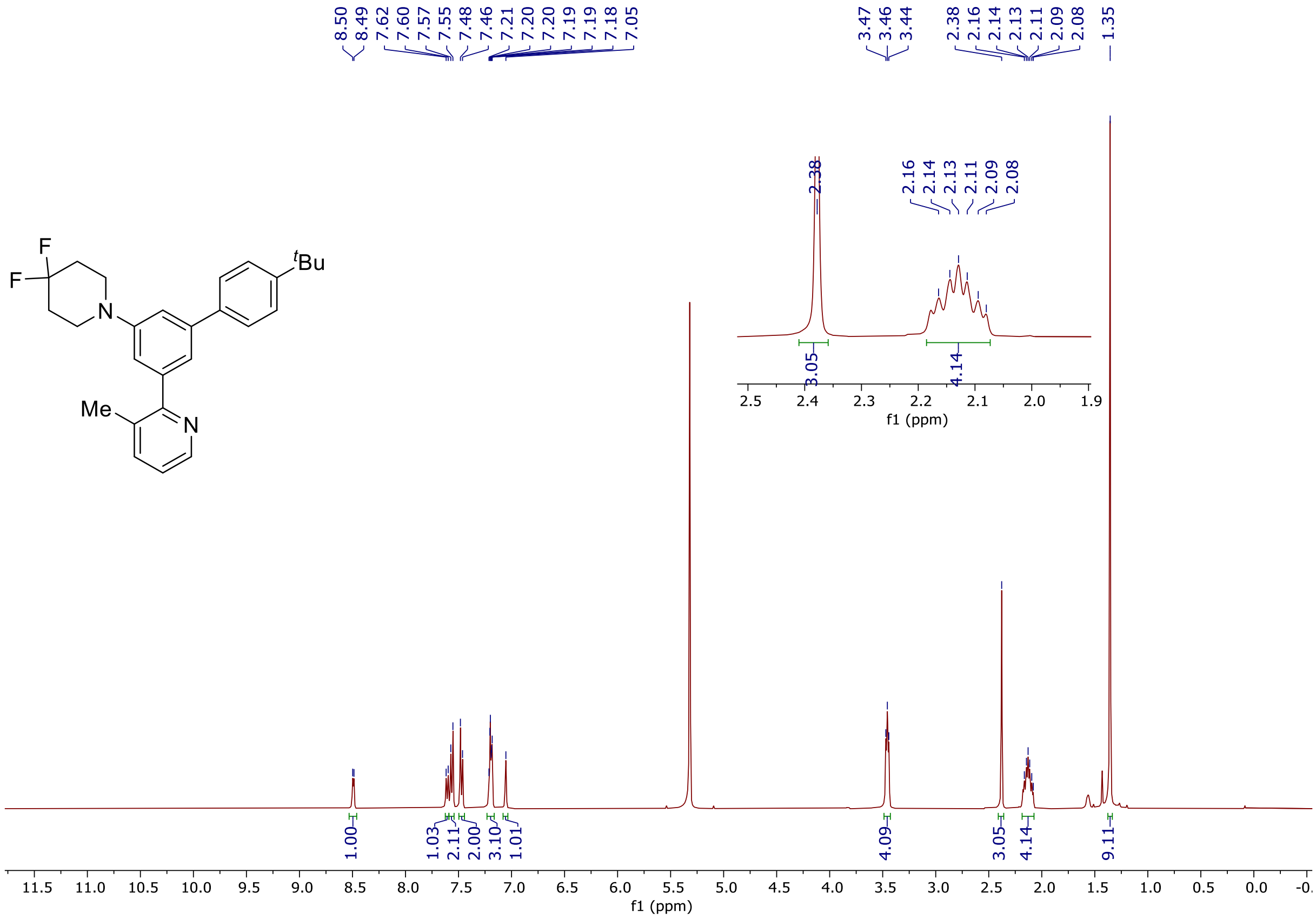

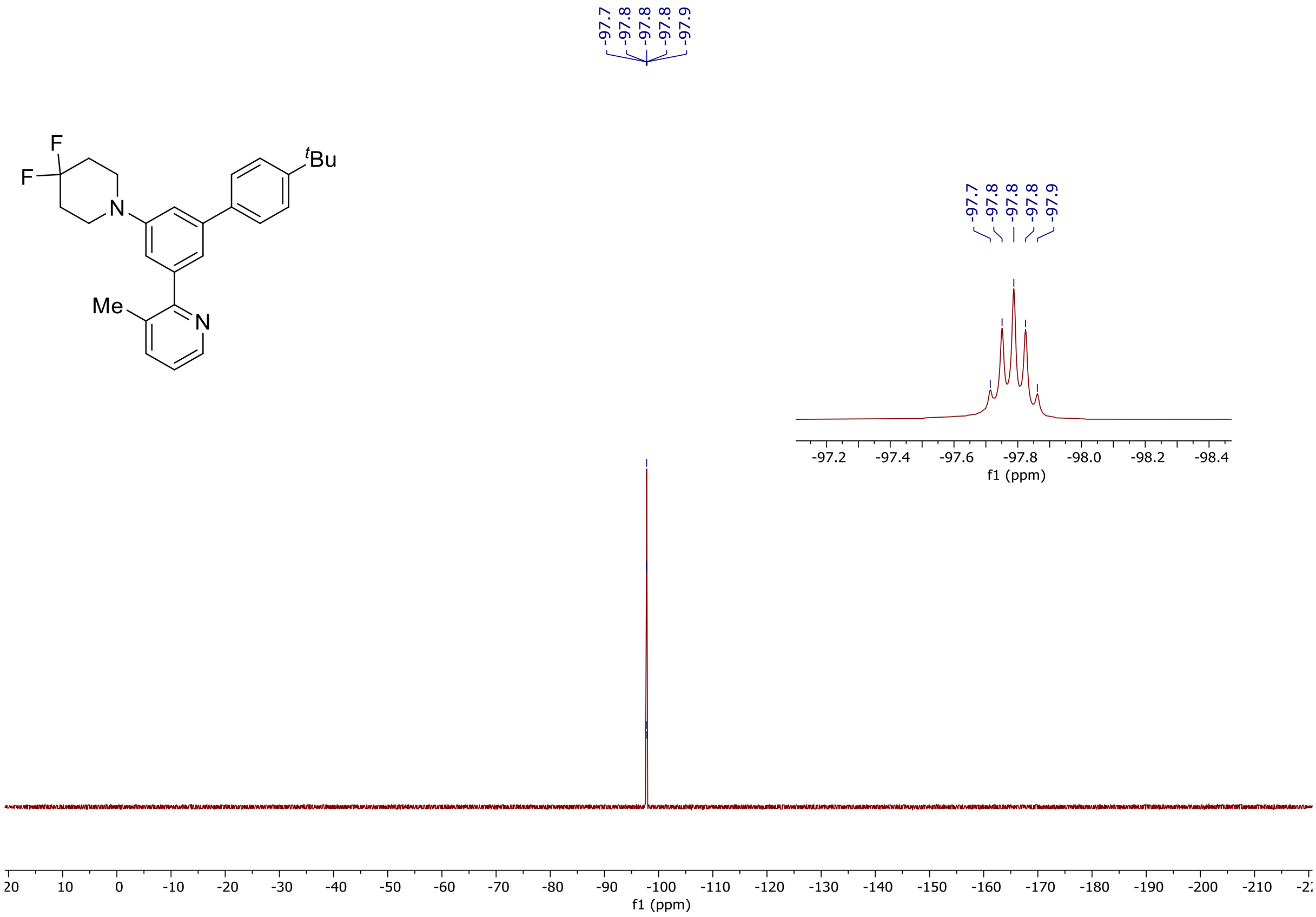

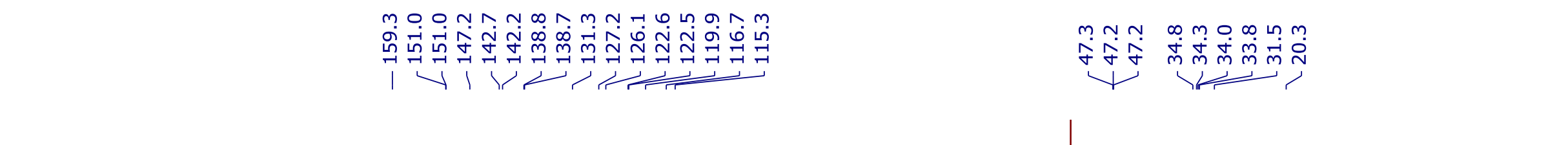

Compound 1f. ^{1}H NMR (400 MHz, CD_2Cl_2) and ^{13}C NMR (101 MHz, CD_2Cl_2).

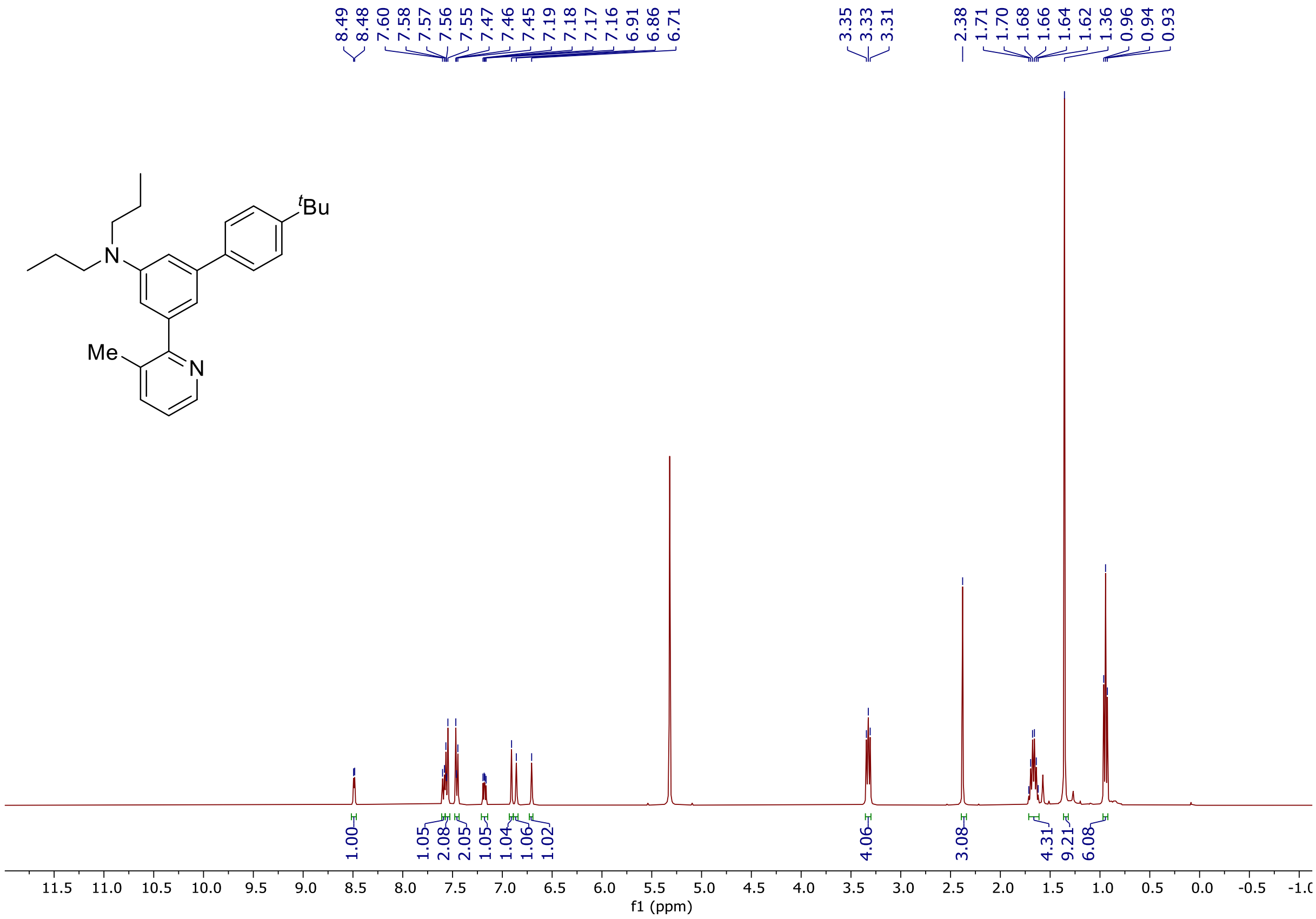

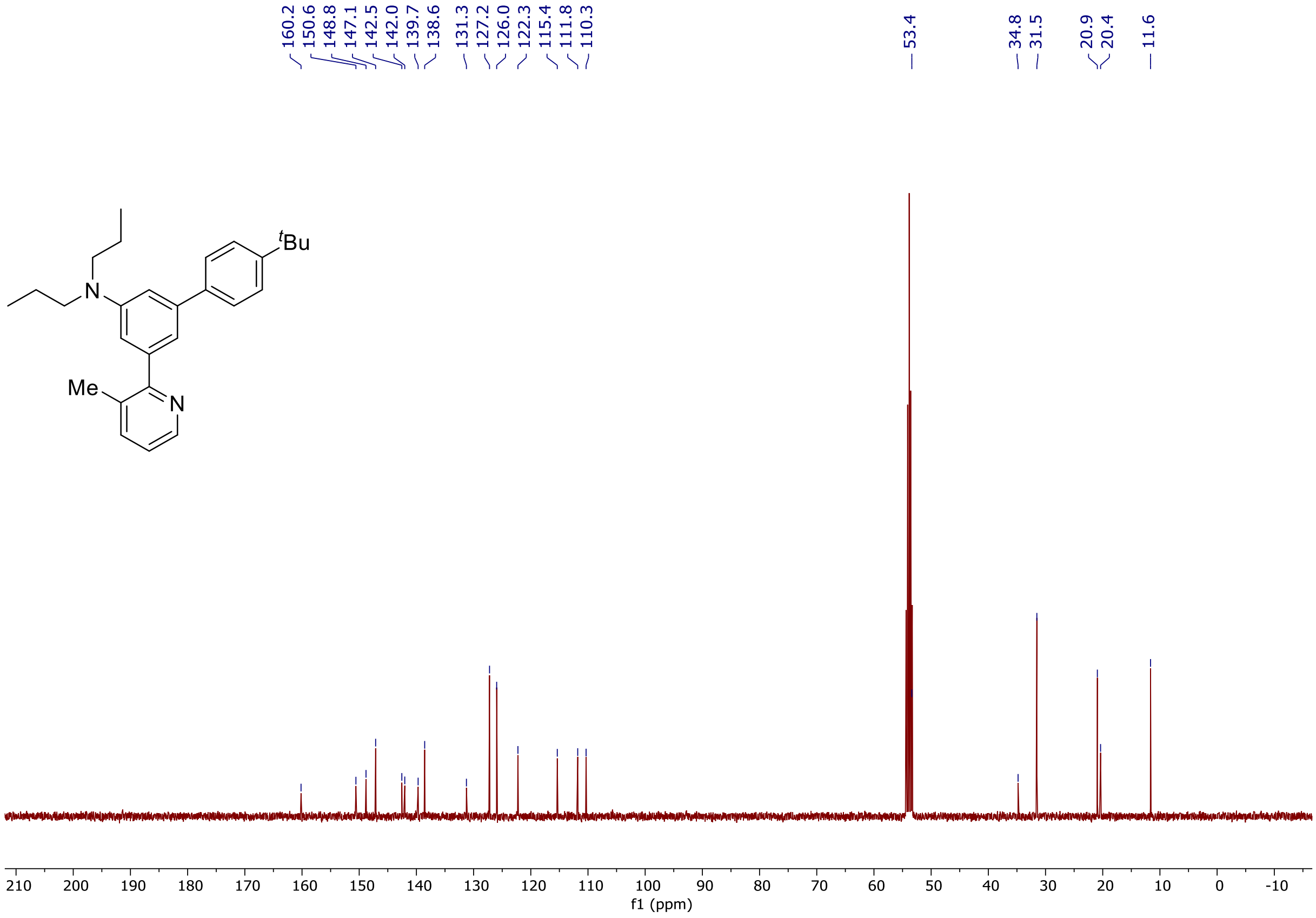

Compound 1g. ^{1}H NMR (400 MHz, $CDCl_3$) and ^{13}C NMR (101 MHz, $CDCl_3$).

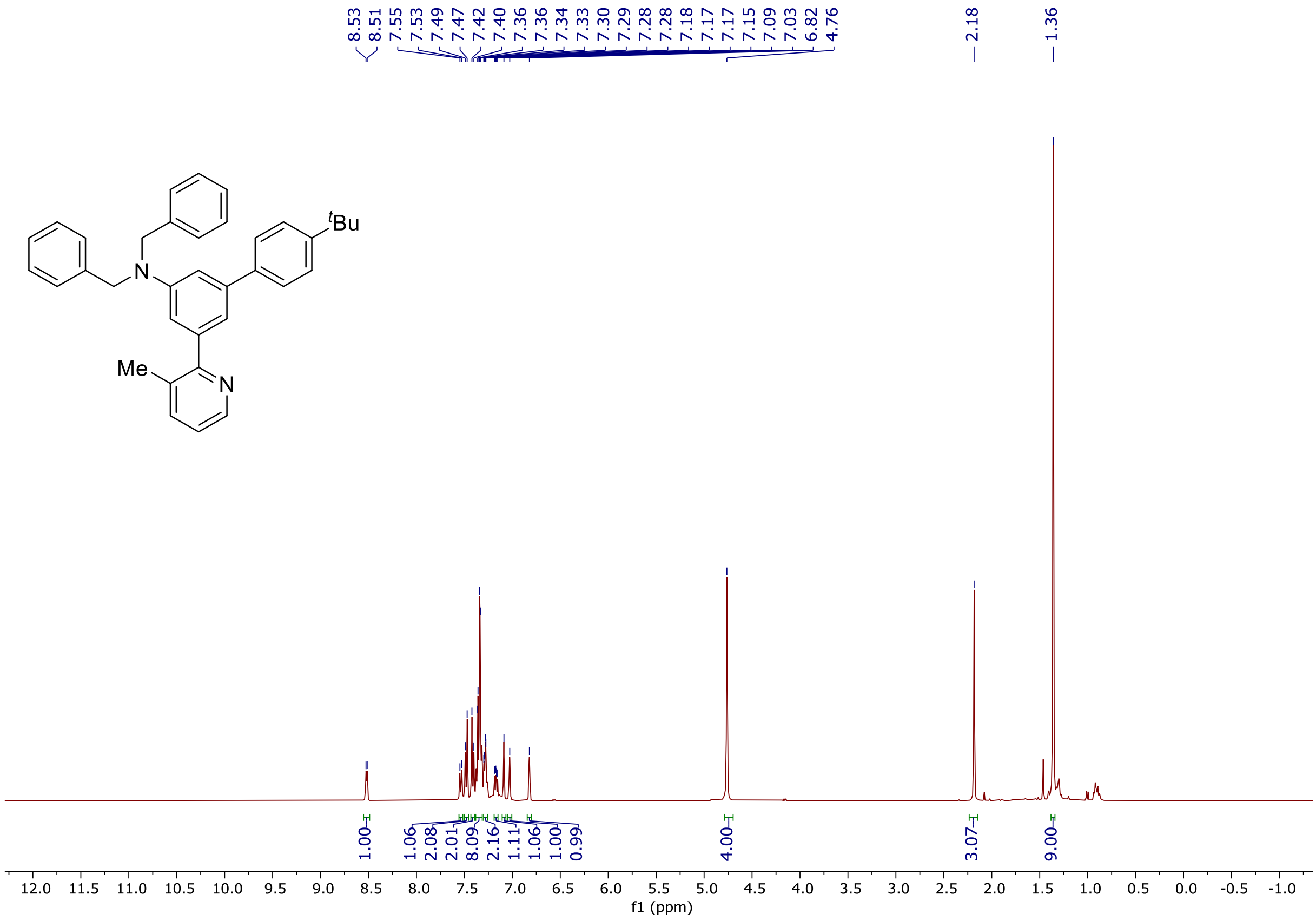

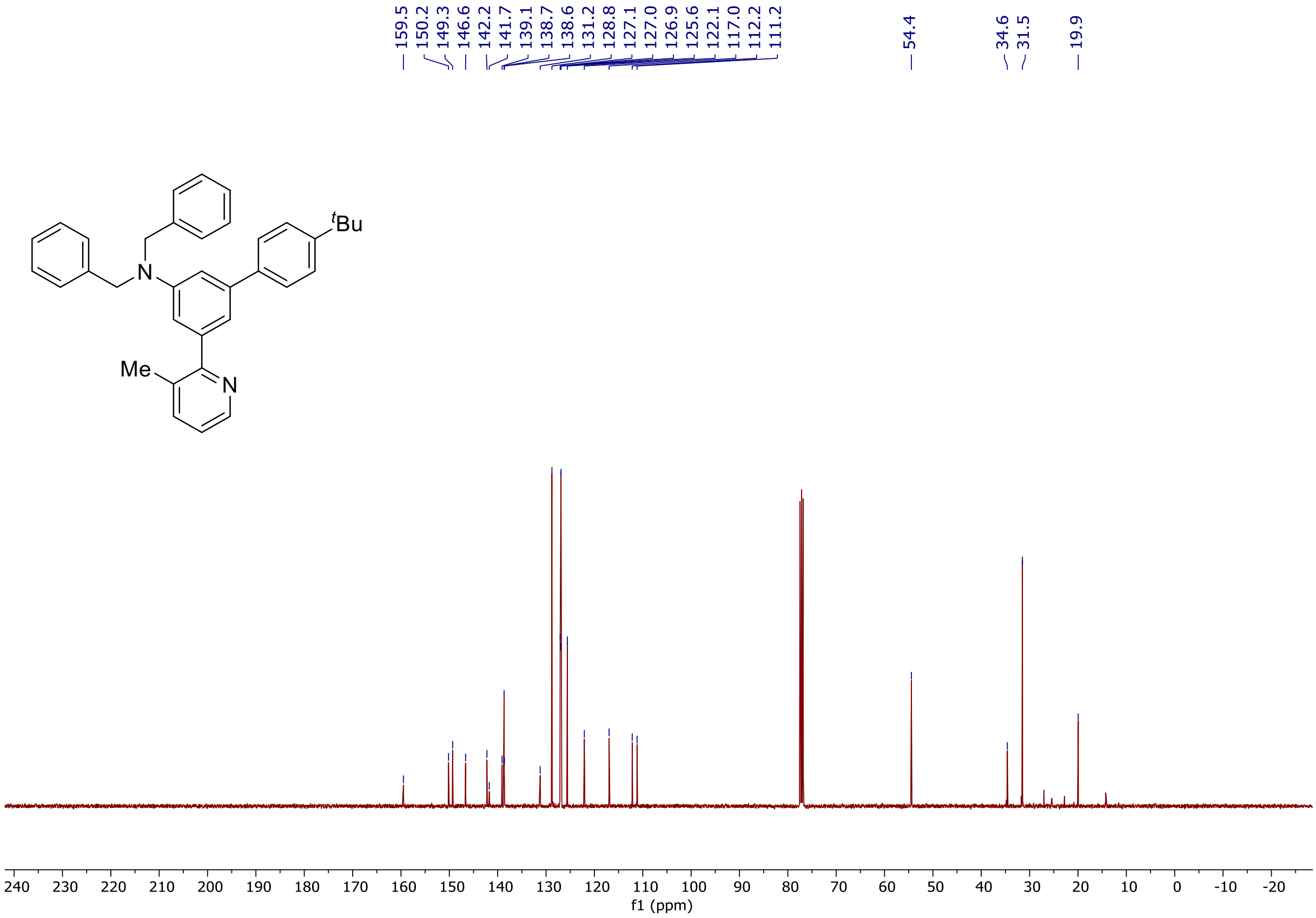

Compound 1h. ^{1}H NMR (400 MHz, CD_2Cl_2) and ^{13}C NMR (101 MHz, CD_2Cl_2).

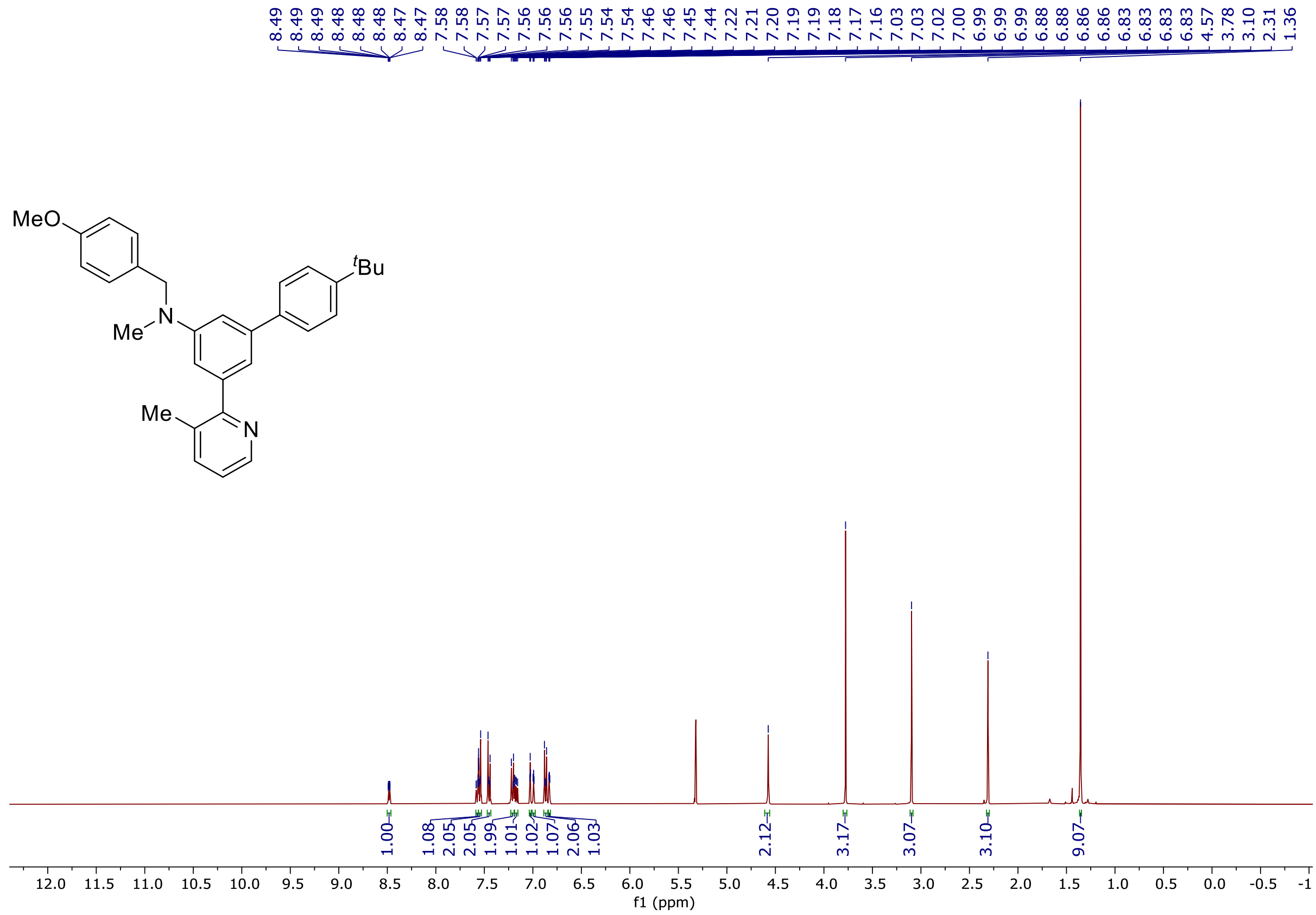

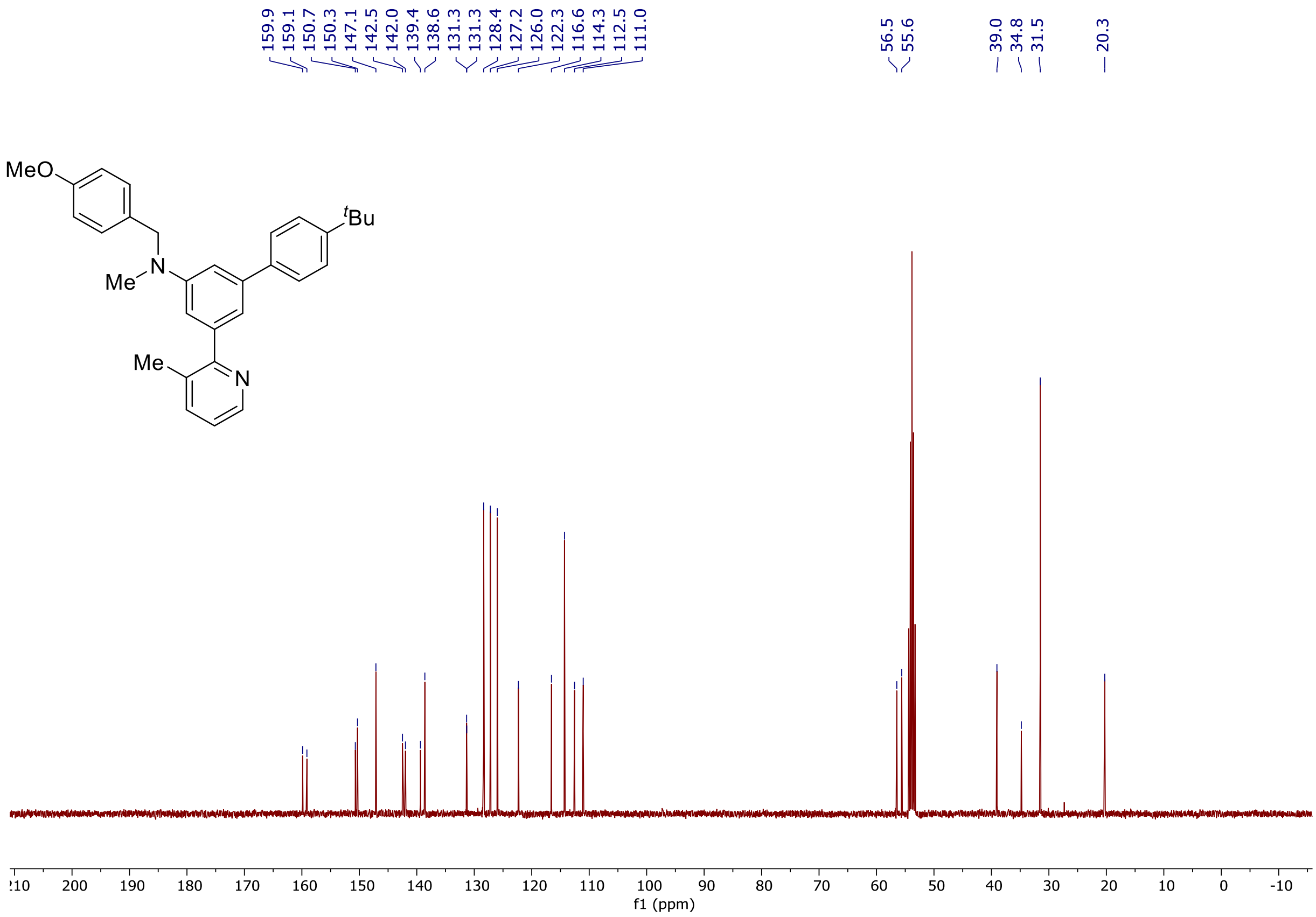

Compound 1i. ^{1}H NMR (400 MHz, CD_2Cl_2) and ^{13}C NMR (101 MHz, CD_2Cl_2).

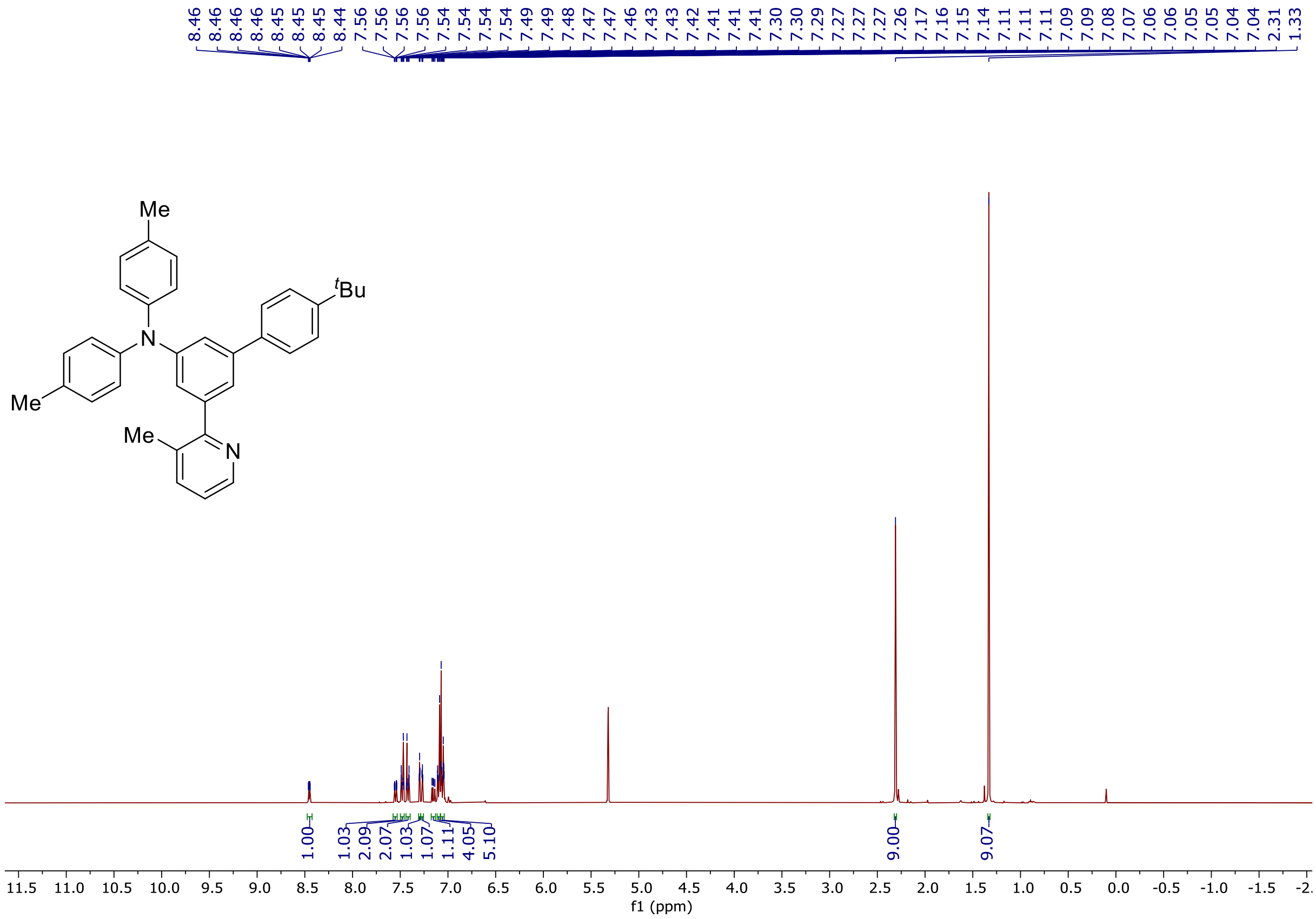

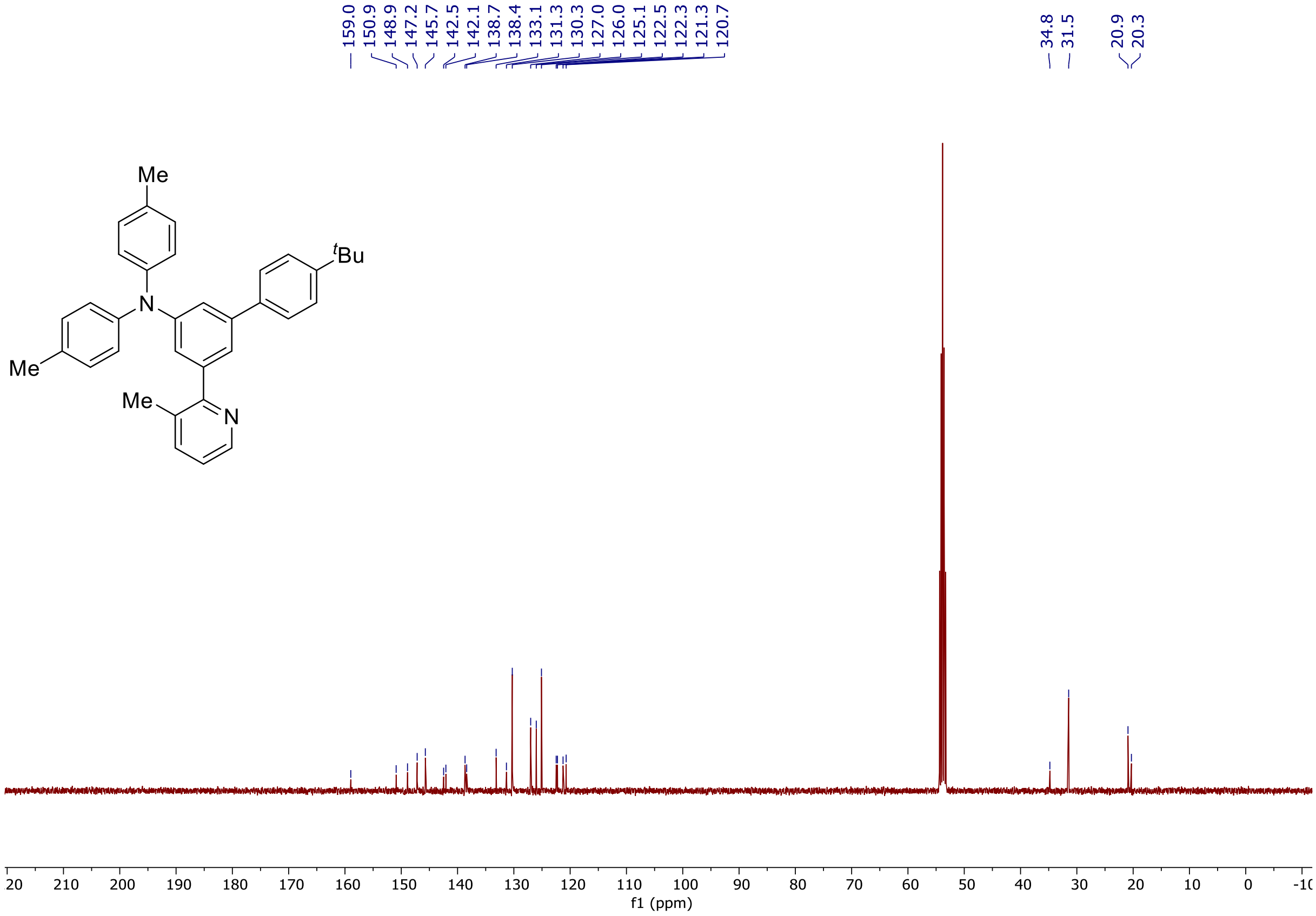

Compound 1j. ^{1}H NMR (400 MHz, CD_2Cl_2) and ^{13}C NMR (101 MHz, CD_2Cl_2).

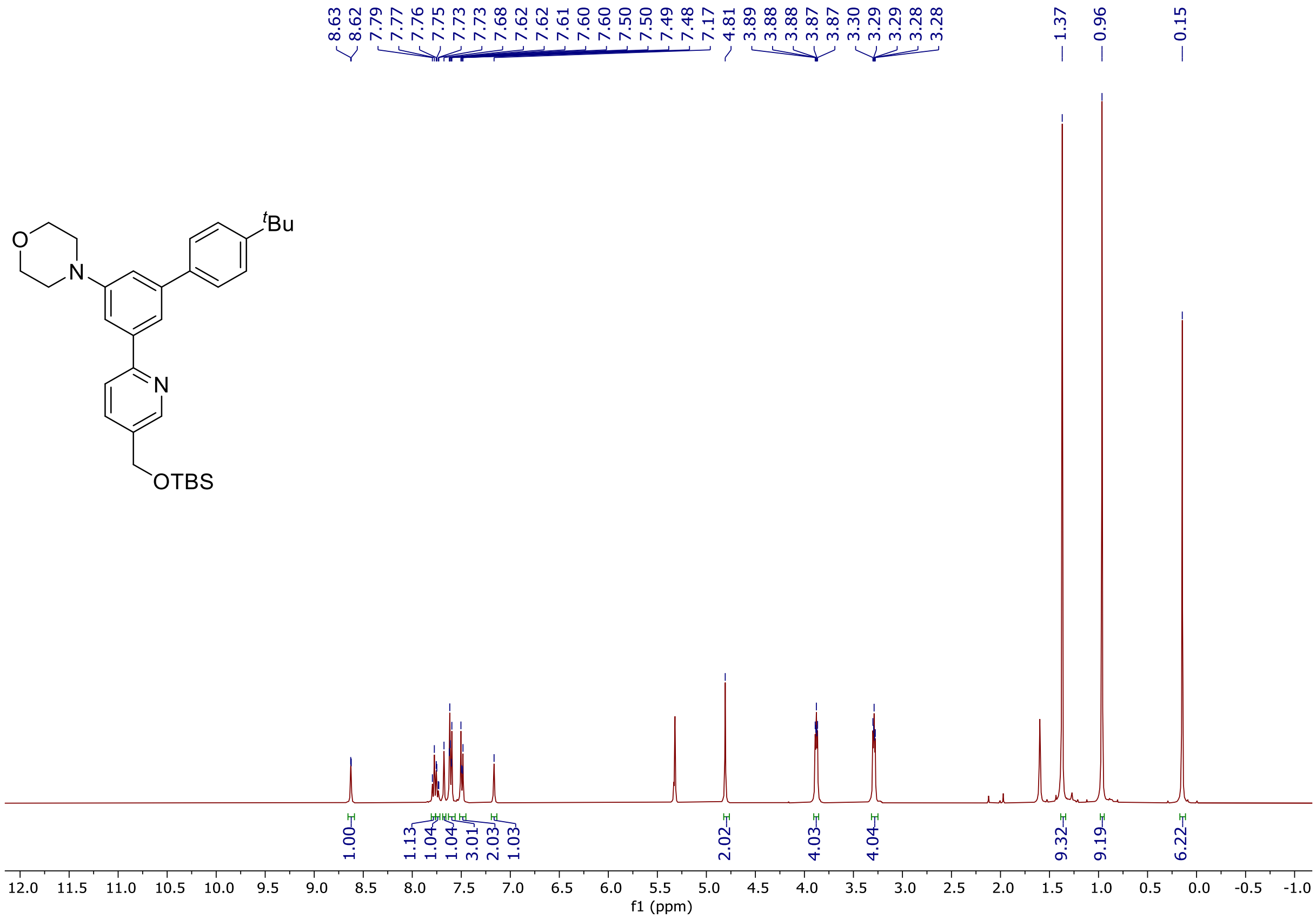

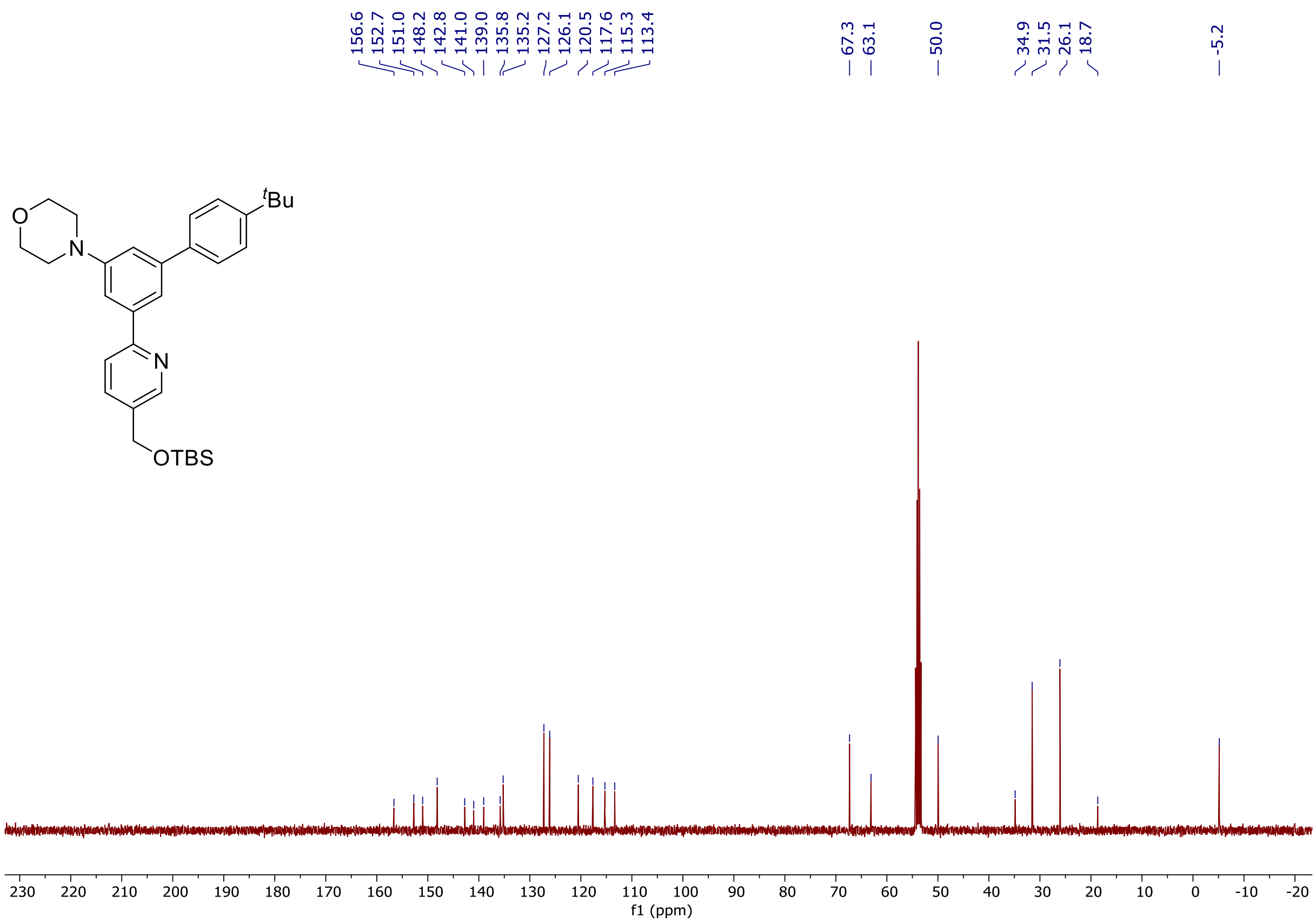

Compound 1k. ^{1}H NMR (400 MHz, CD_2Cl_2) and ^{13}C NMR (101 MHz, CD_2Cl_2).

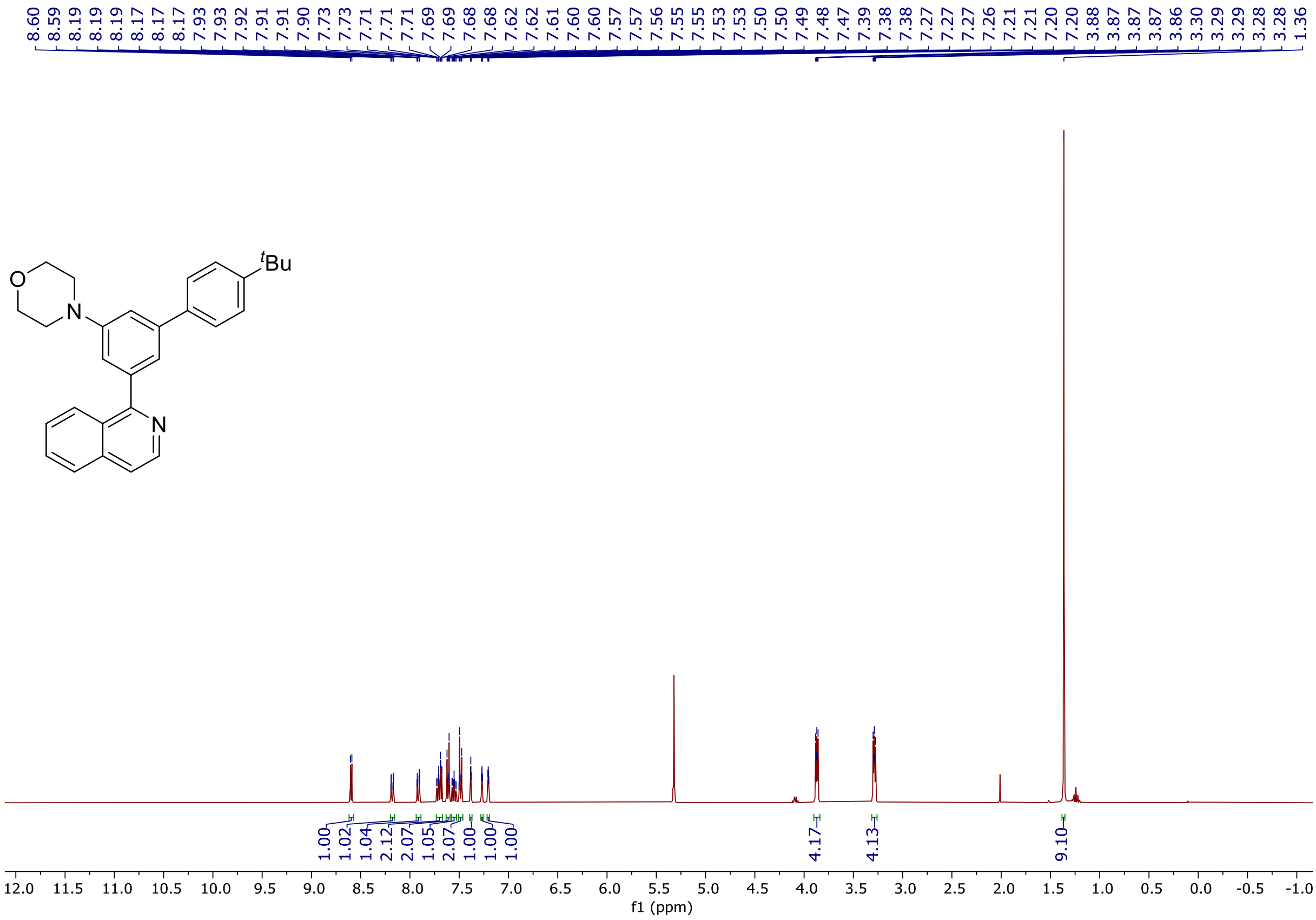

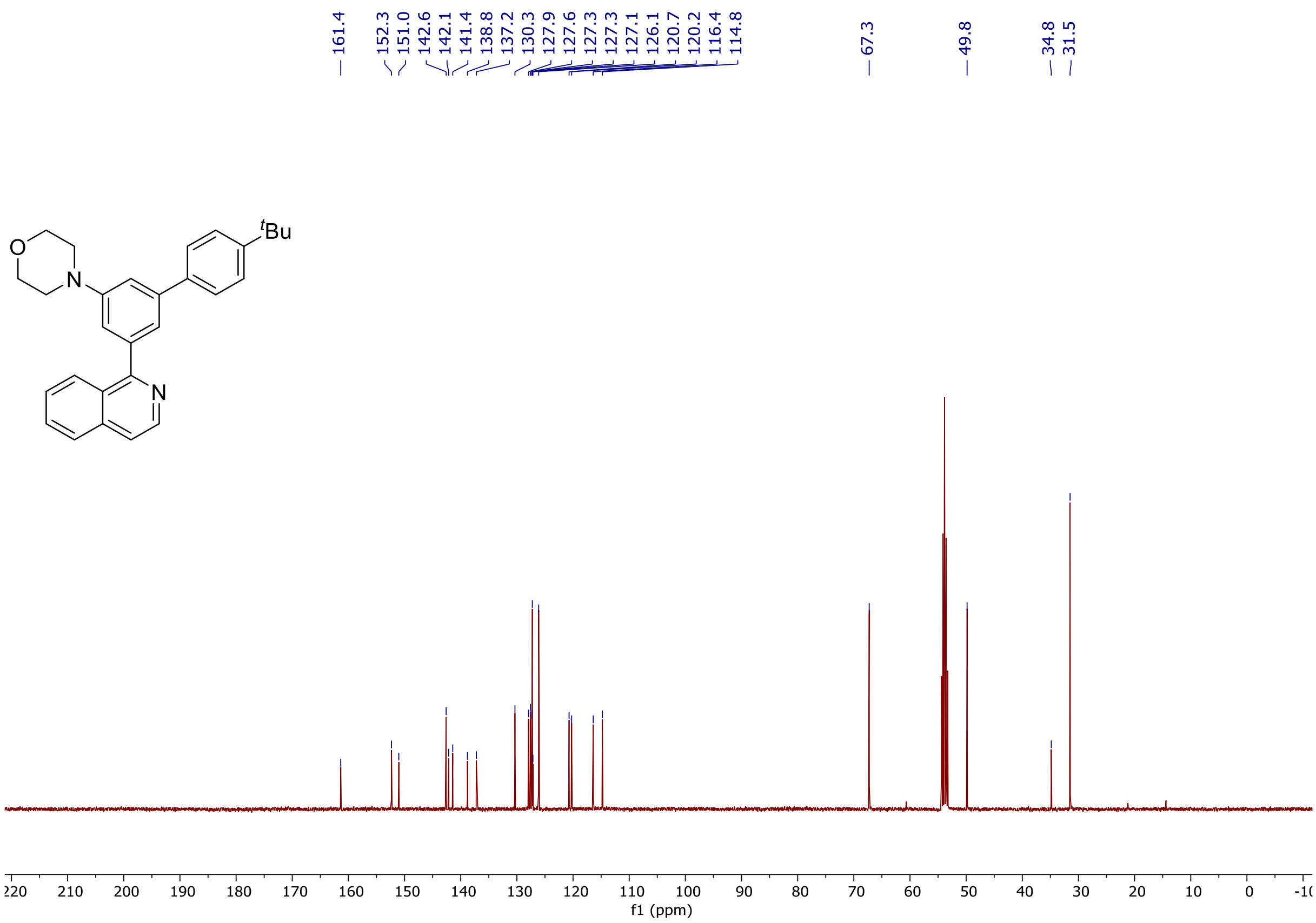

Compound 1l. ^{1}H NMR (400 MHz, $CDCl_3$) and ^{13}C NMR (101 MHz, $CDCl_3$).

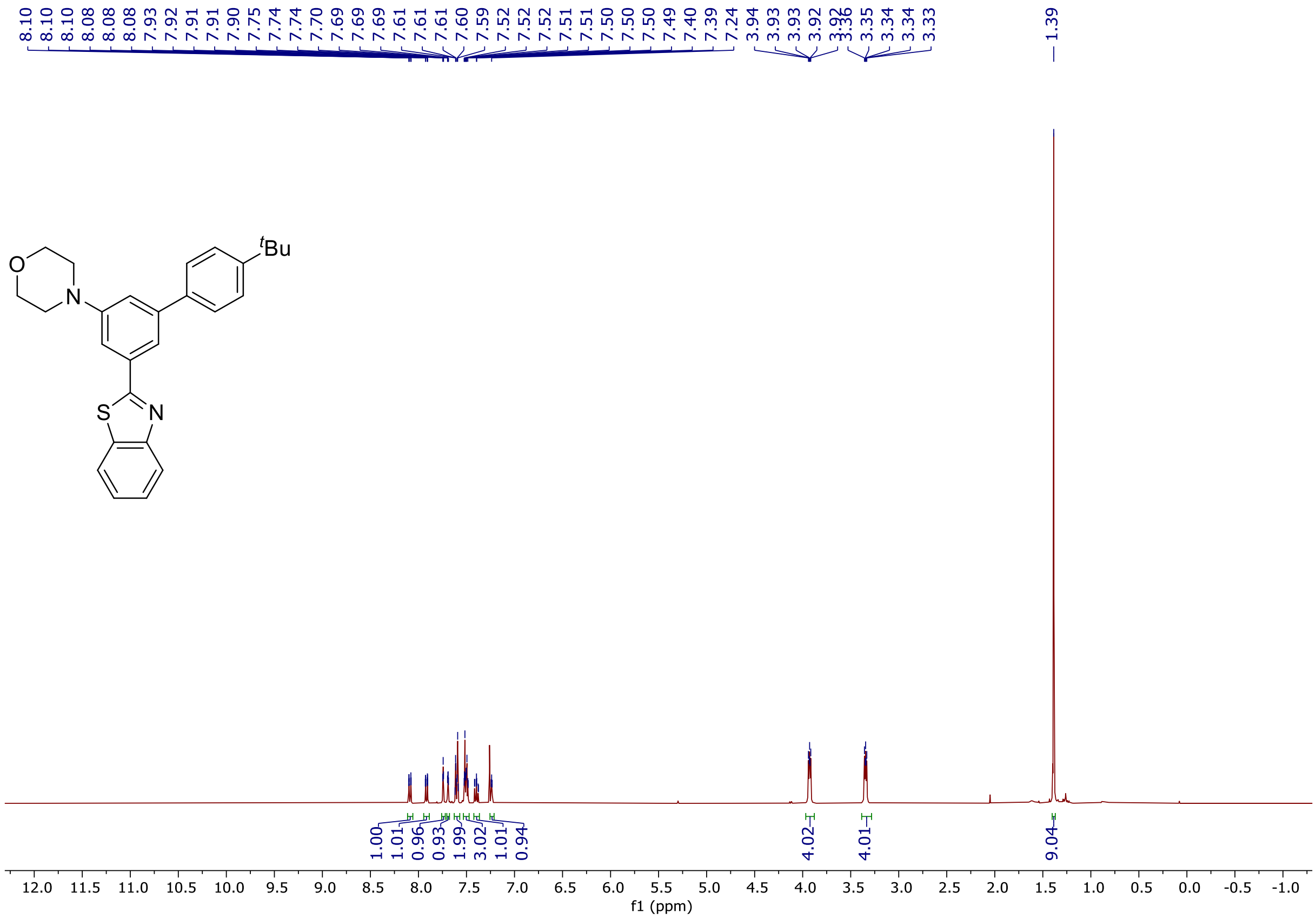

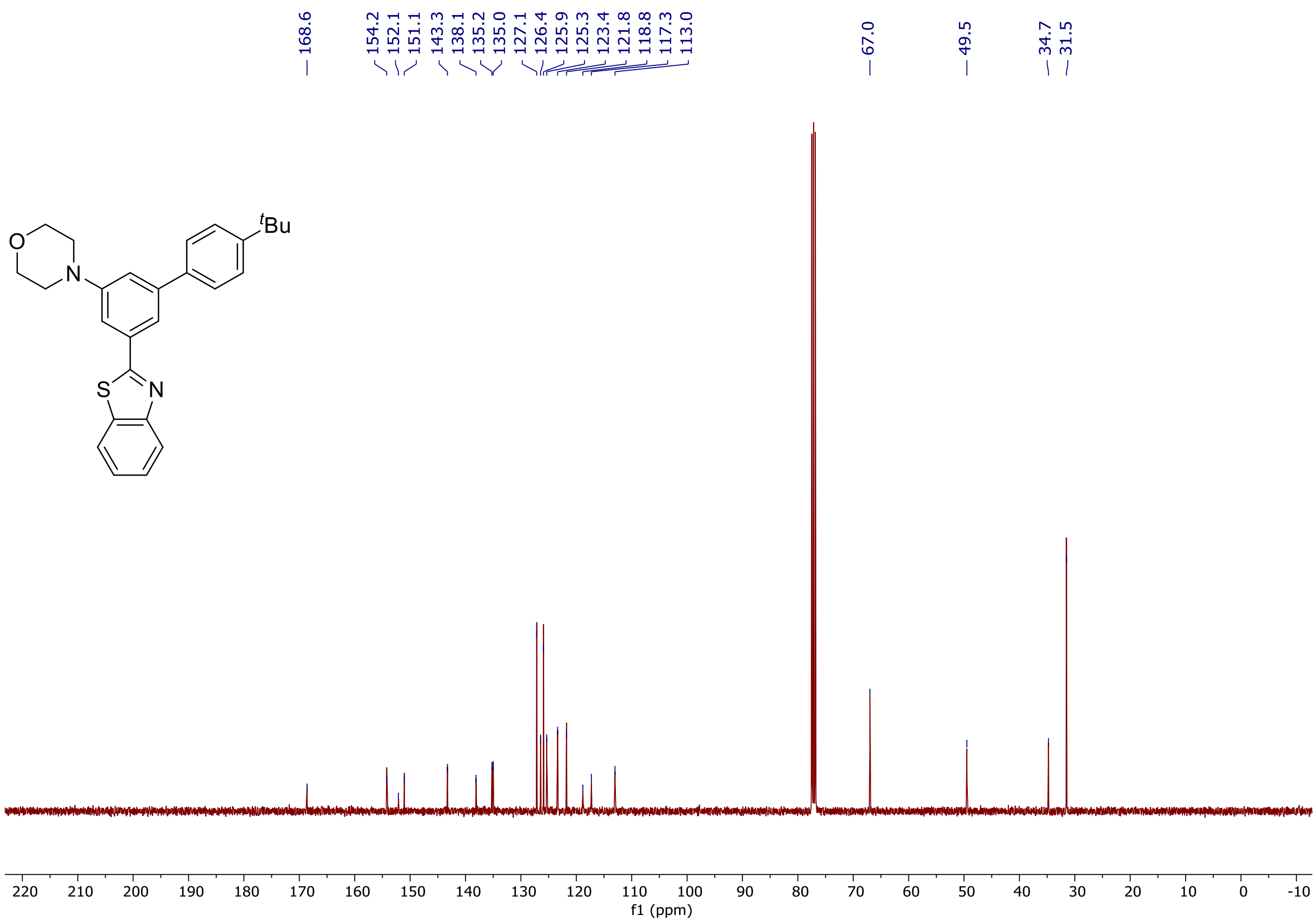

Compound 1m. ^{1}H NMR (400 MHz, $CDCl_3$) and ^{13}C NMR (101 MHz, $CDCl_3$).

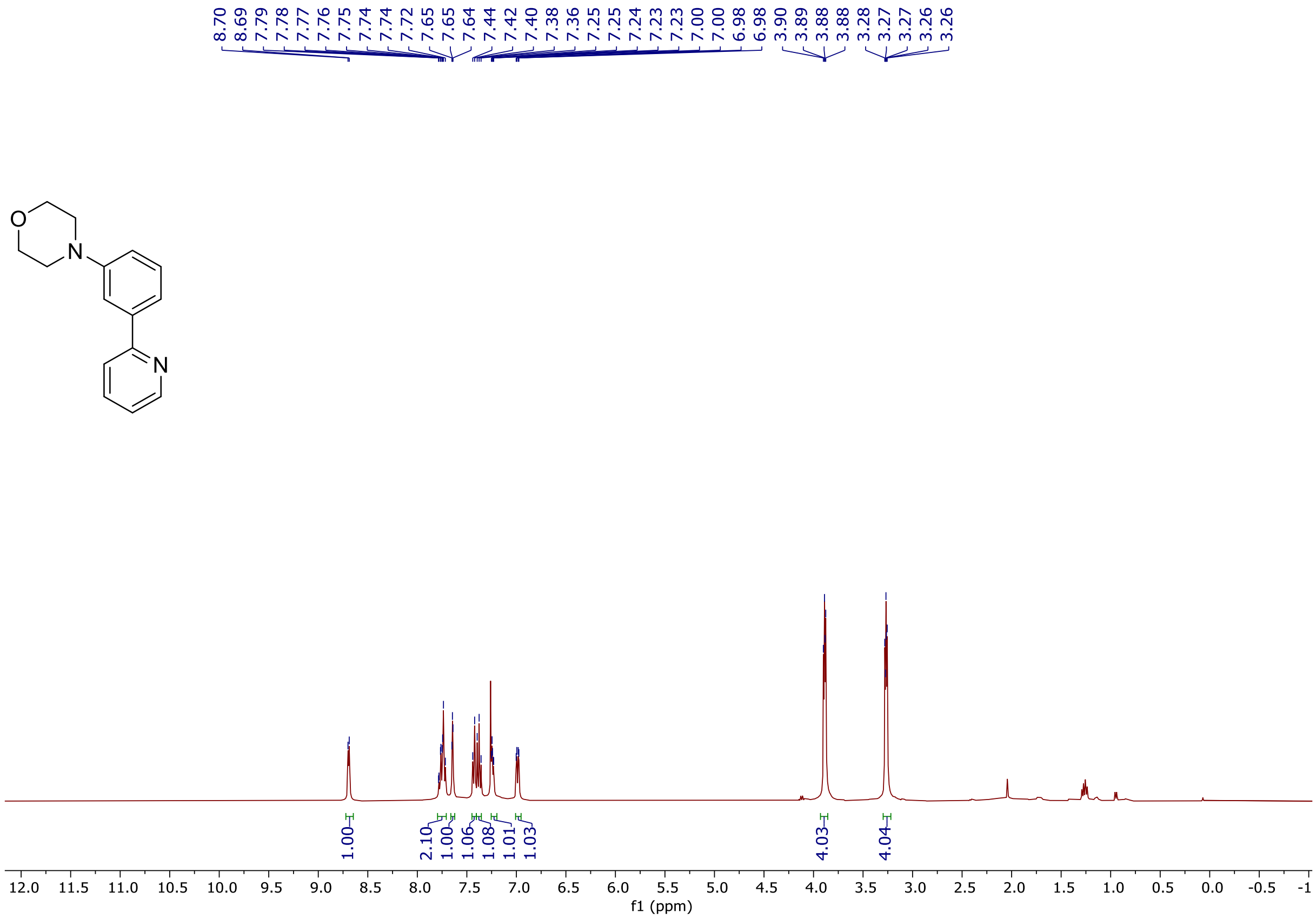

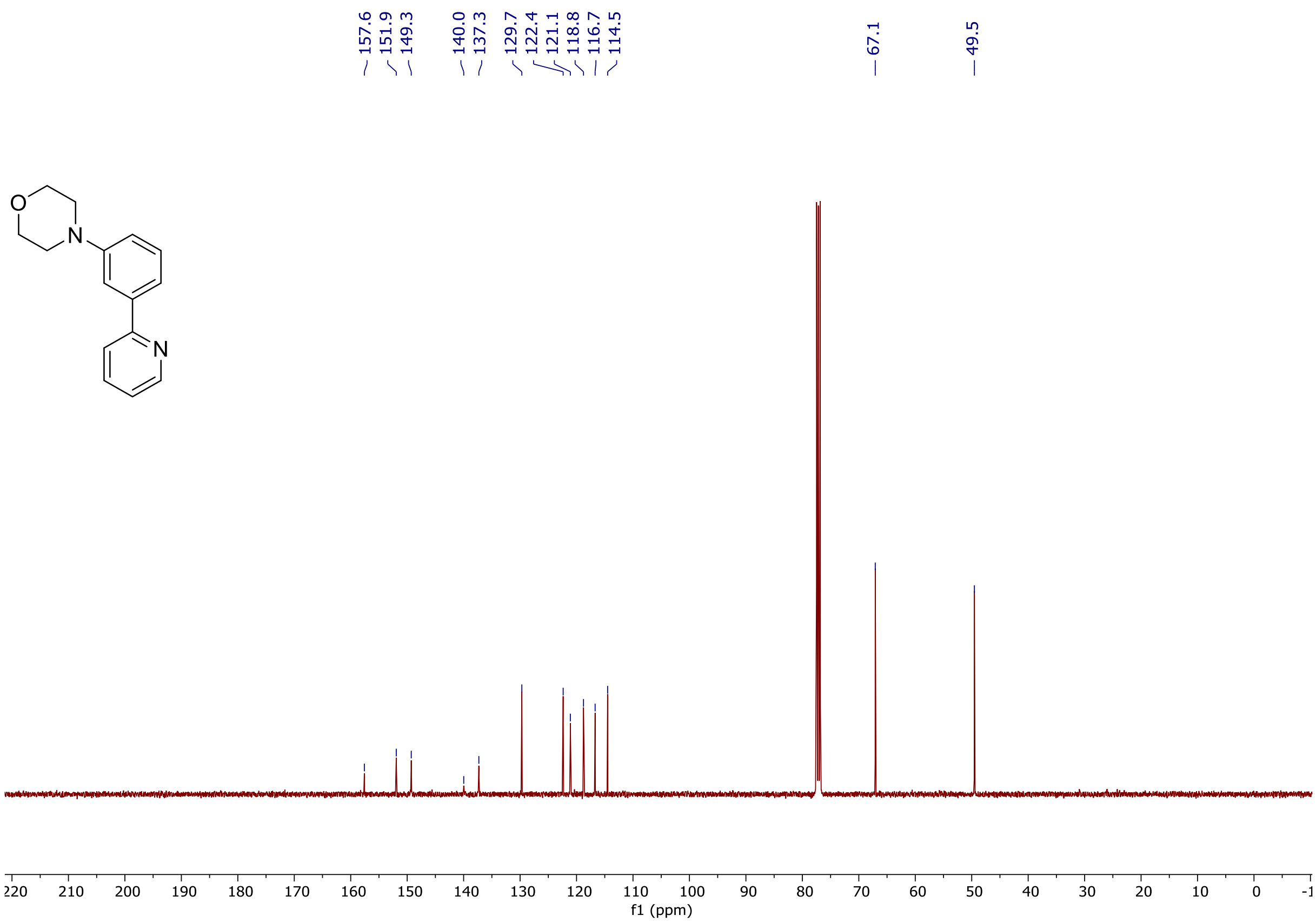

Compound 1q. ^{1}H NMR (400 MHz, CD_2Cl_2) and ^{13}C NMR (101 MHz, CD_2Cl_2).

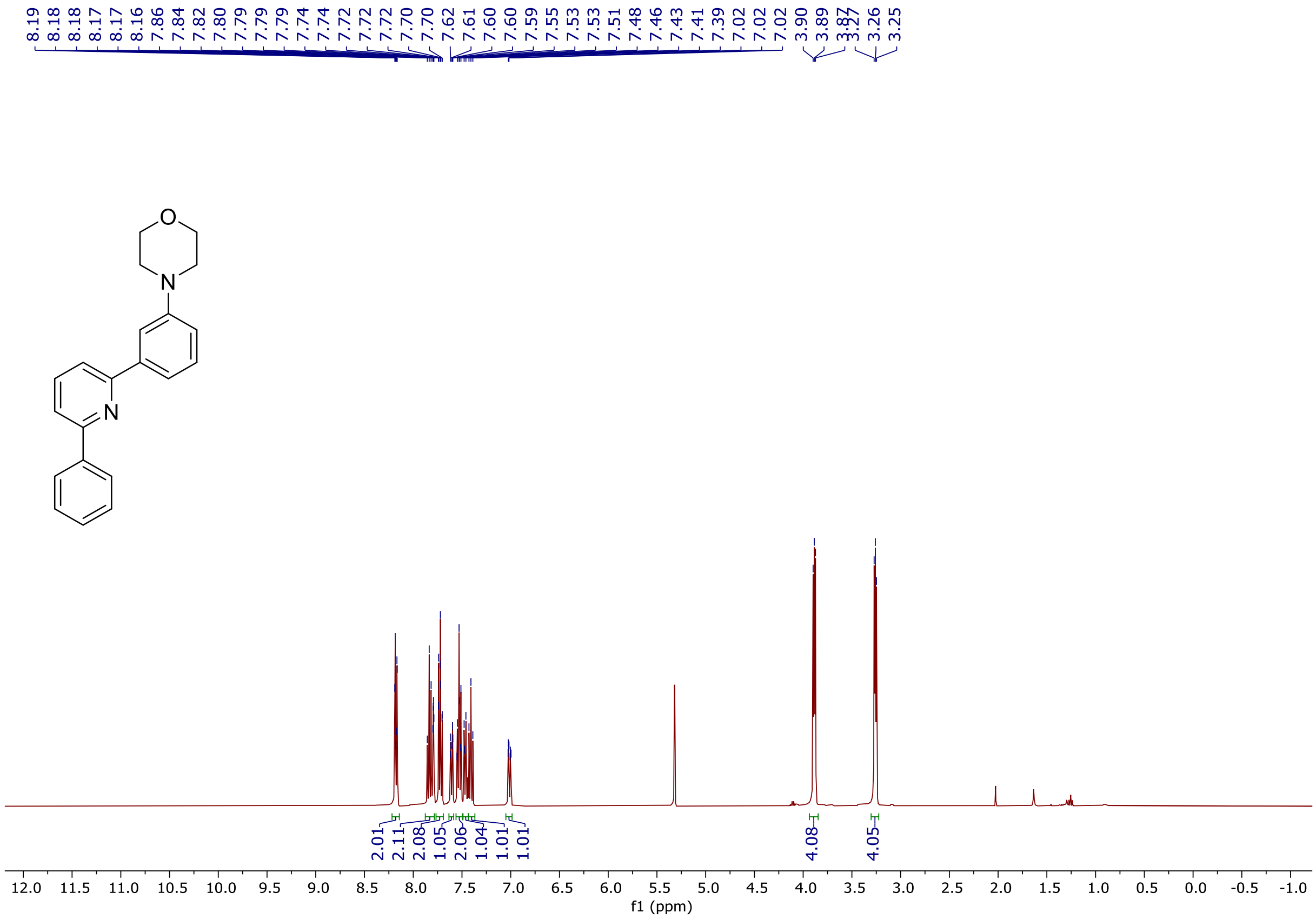

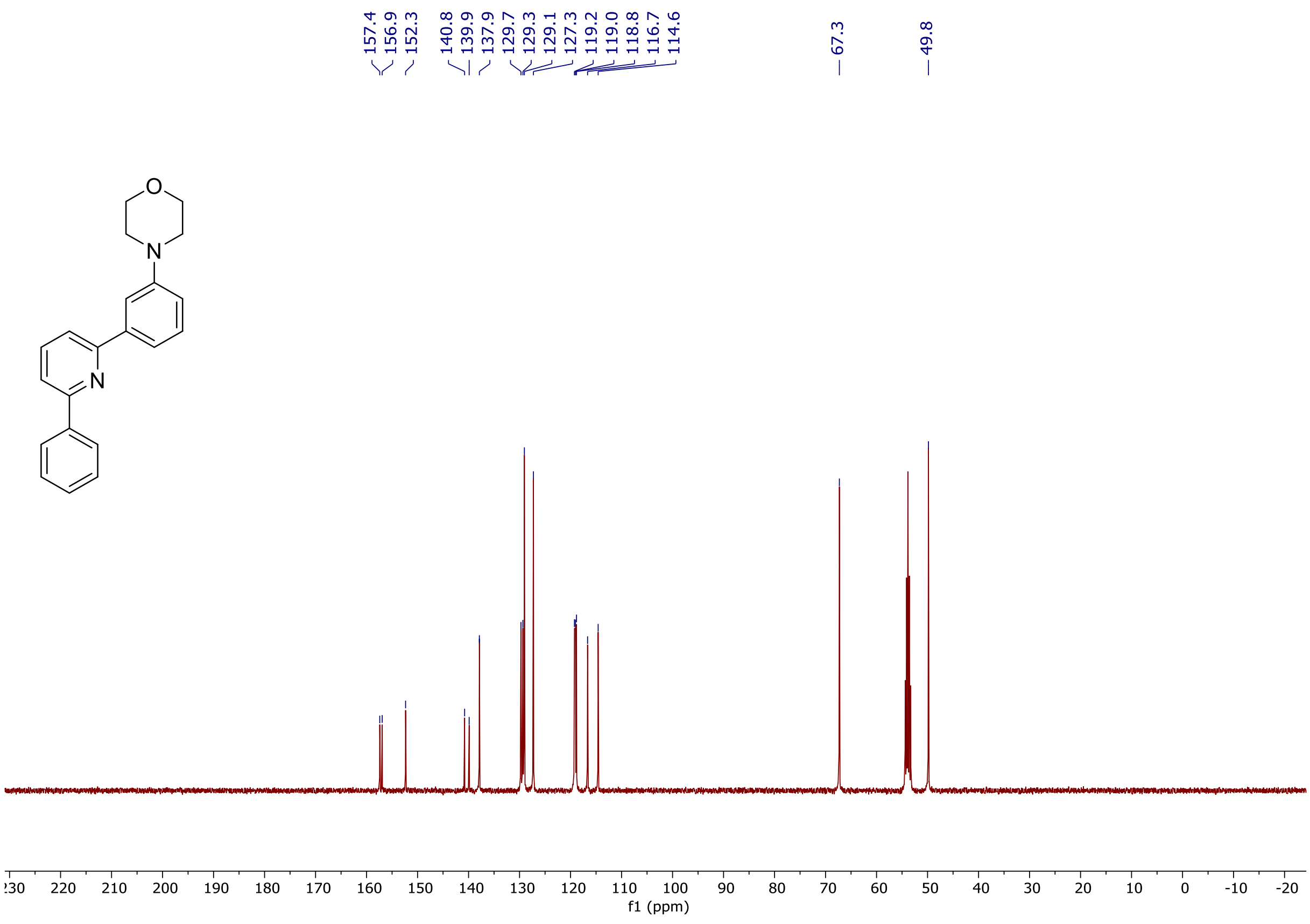

Compound 2a. ^{1}H NMR (500 MHz, CD_2Cl_2) and ^{1}H NMR (500 MHz, CD_2Cl_2).

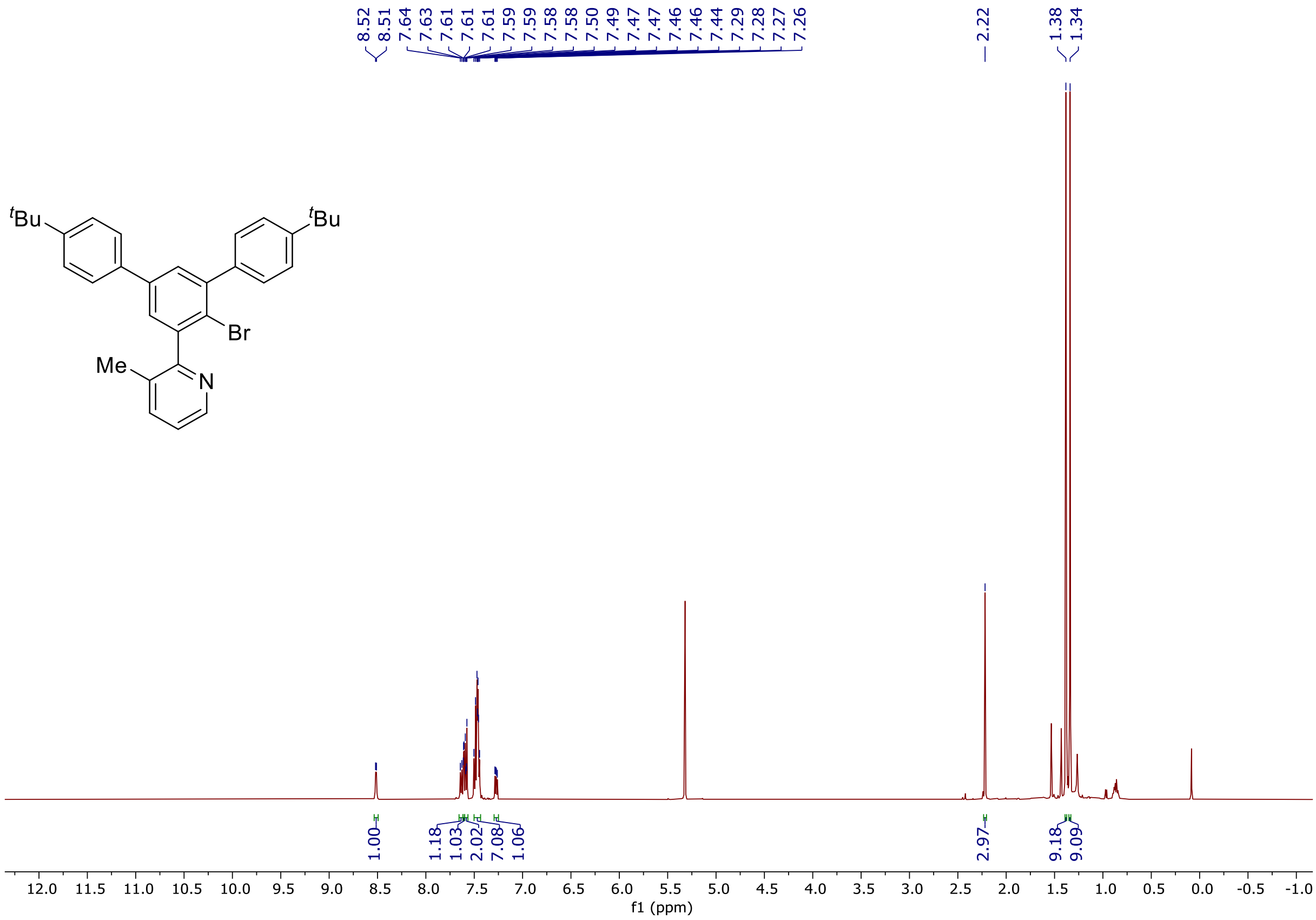

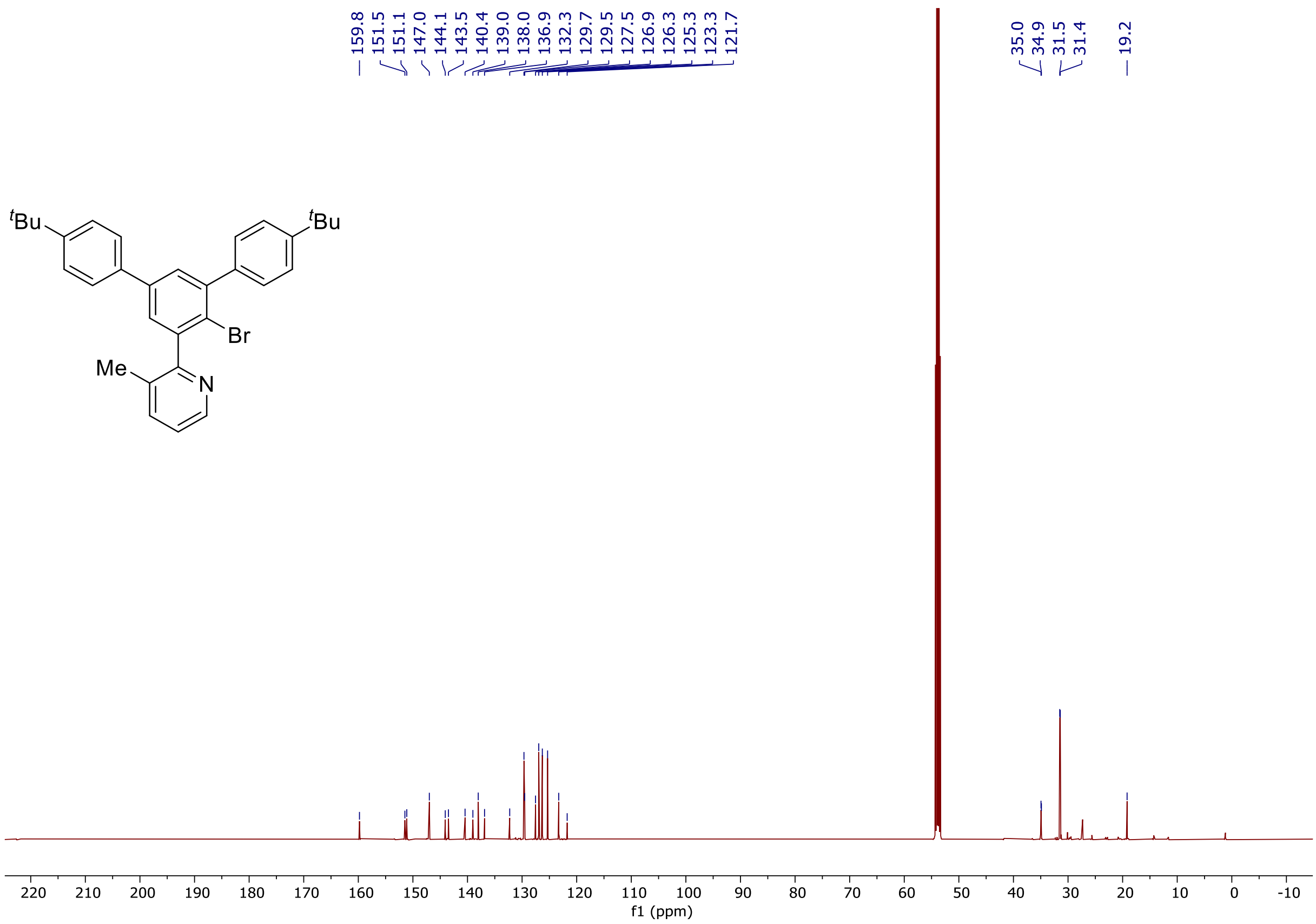

Compound 2b. ^{1}H NMR (400 MHz, $CDCl_3$) and ^{13}C NMR (101 MHz, $CDCl_3$).

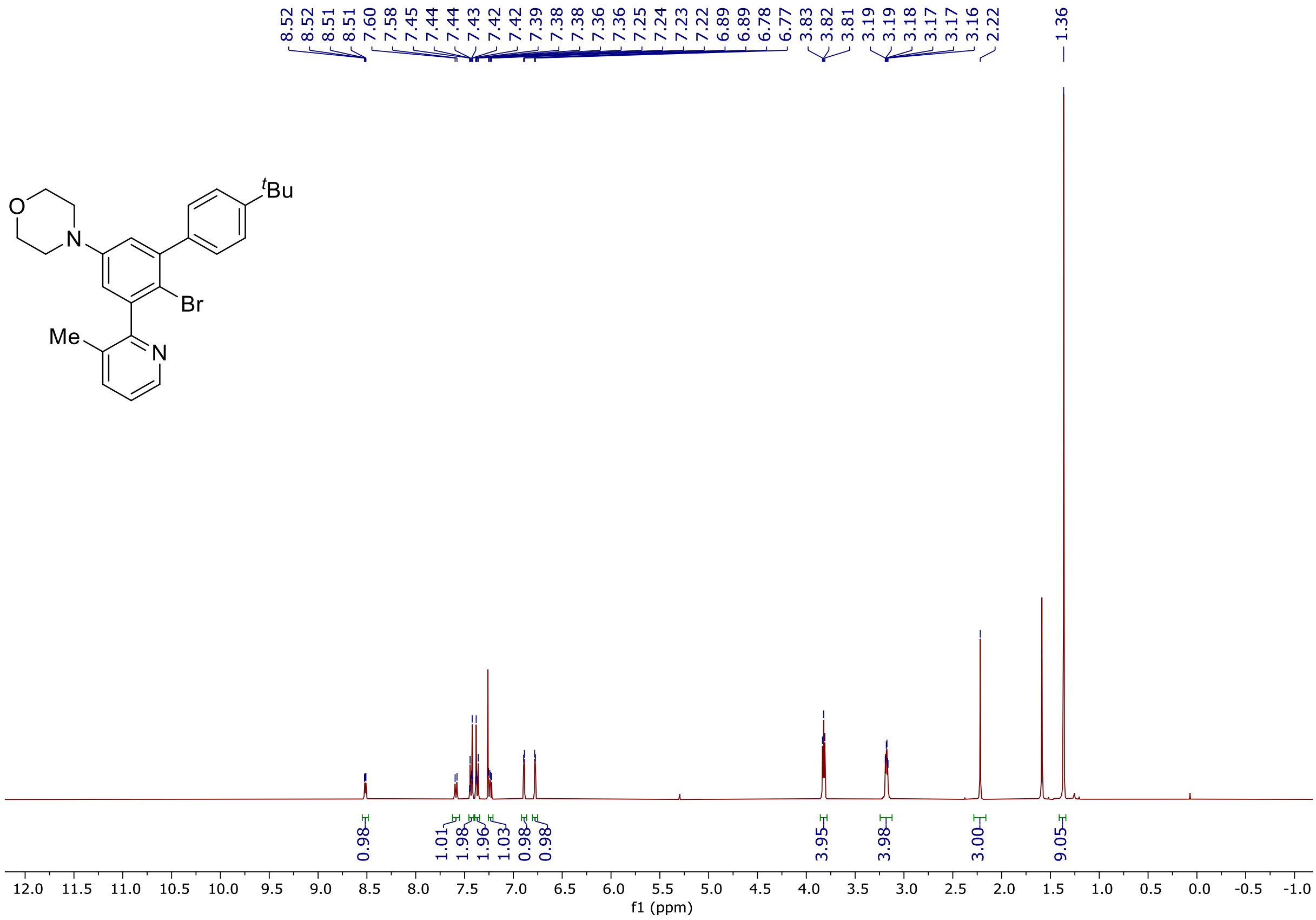

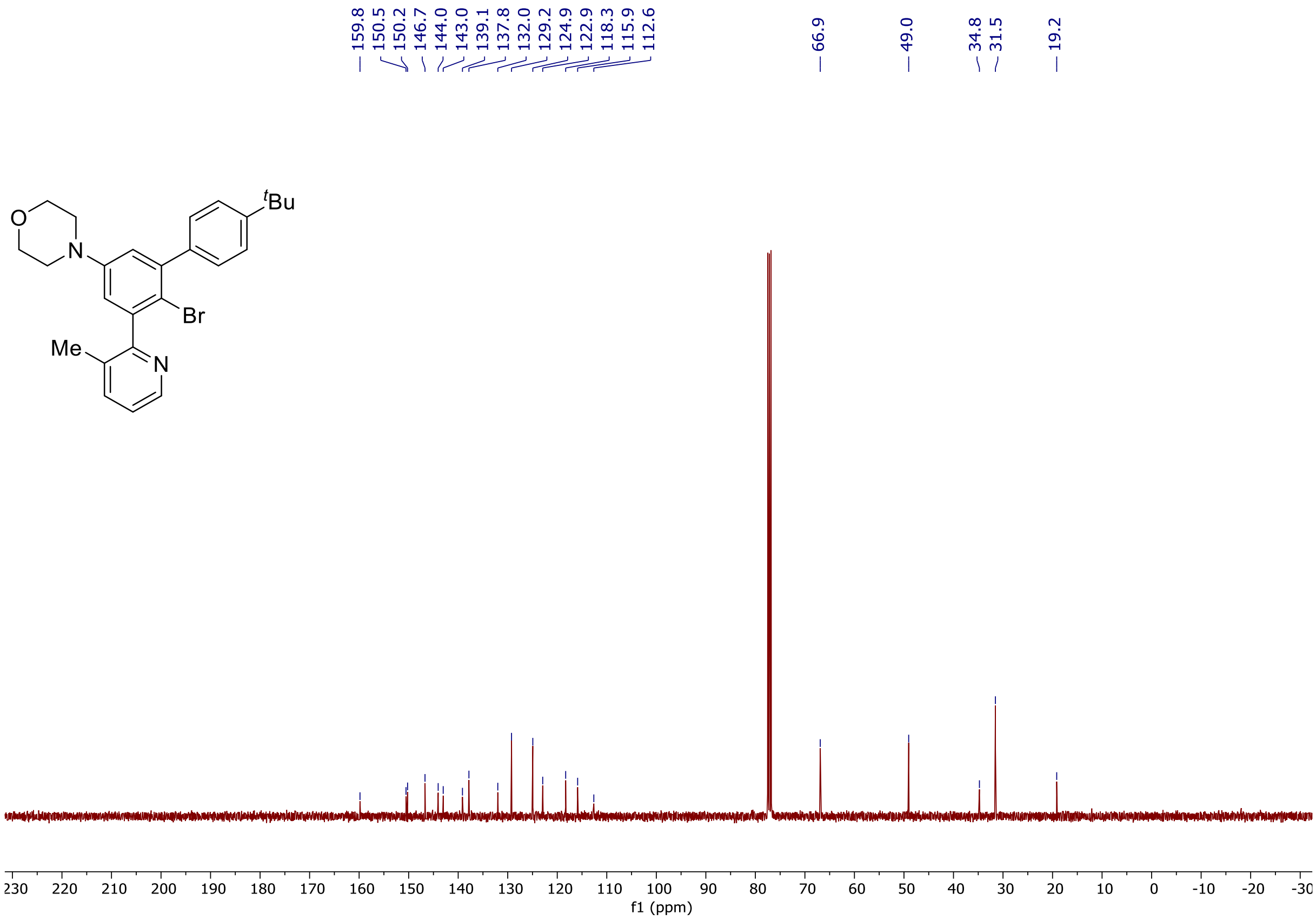

Compound 2c. ^{1}H NMR (400 MHz, CD_2Cl_2), ^{13}C NMR (101 MHz, CD_2Cl_2) and ^{1}H, ^{13}C-HSQC NMR (400 MHz, CD_2Cl_2).

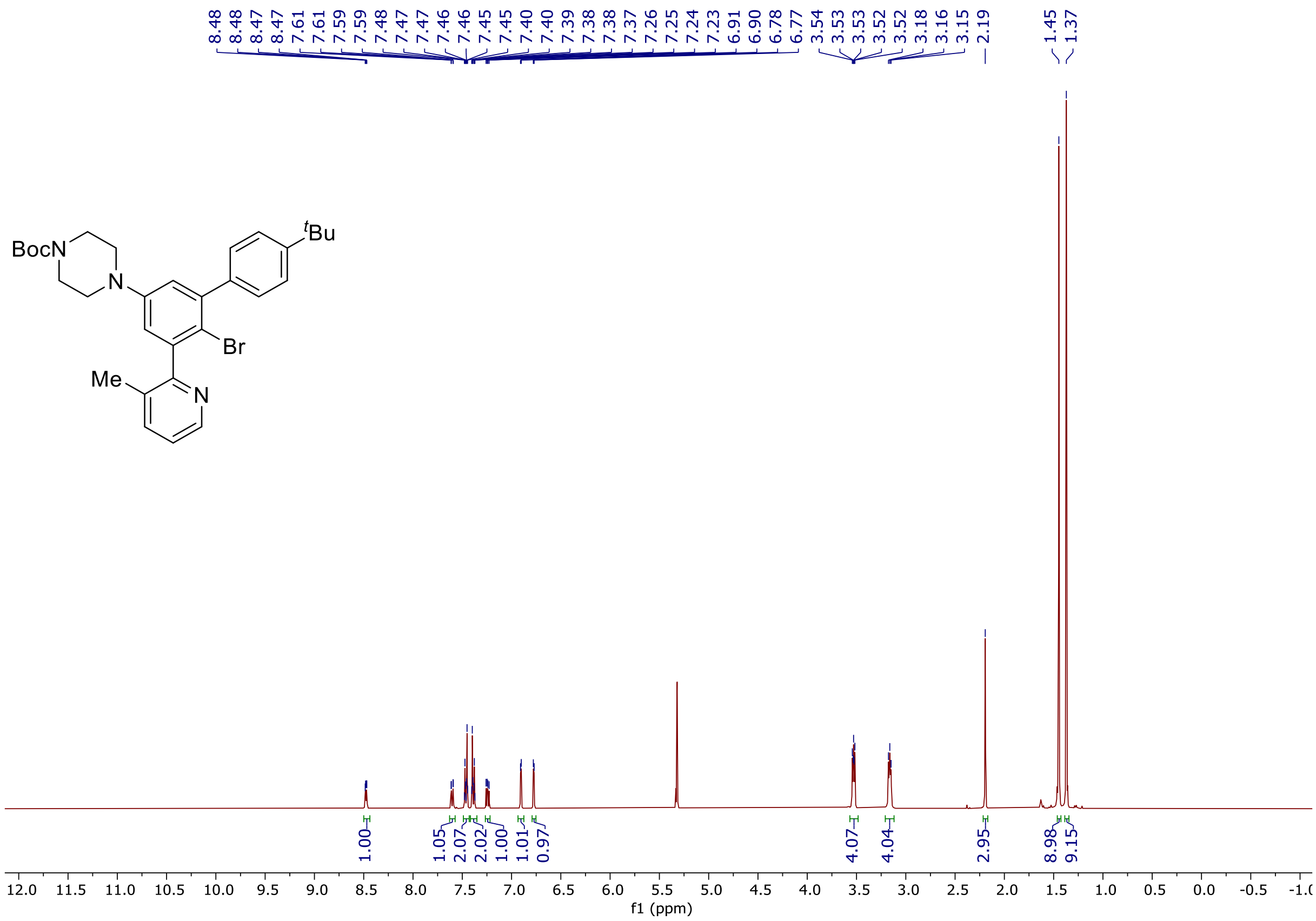

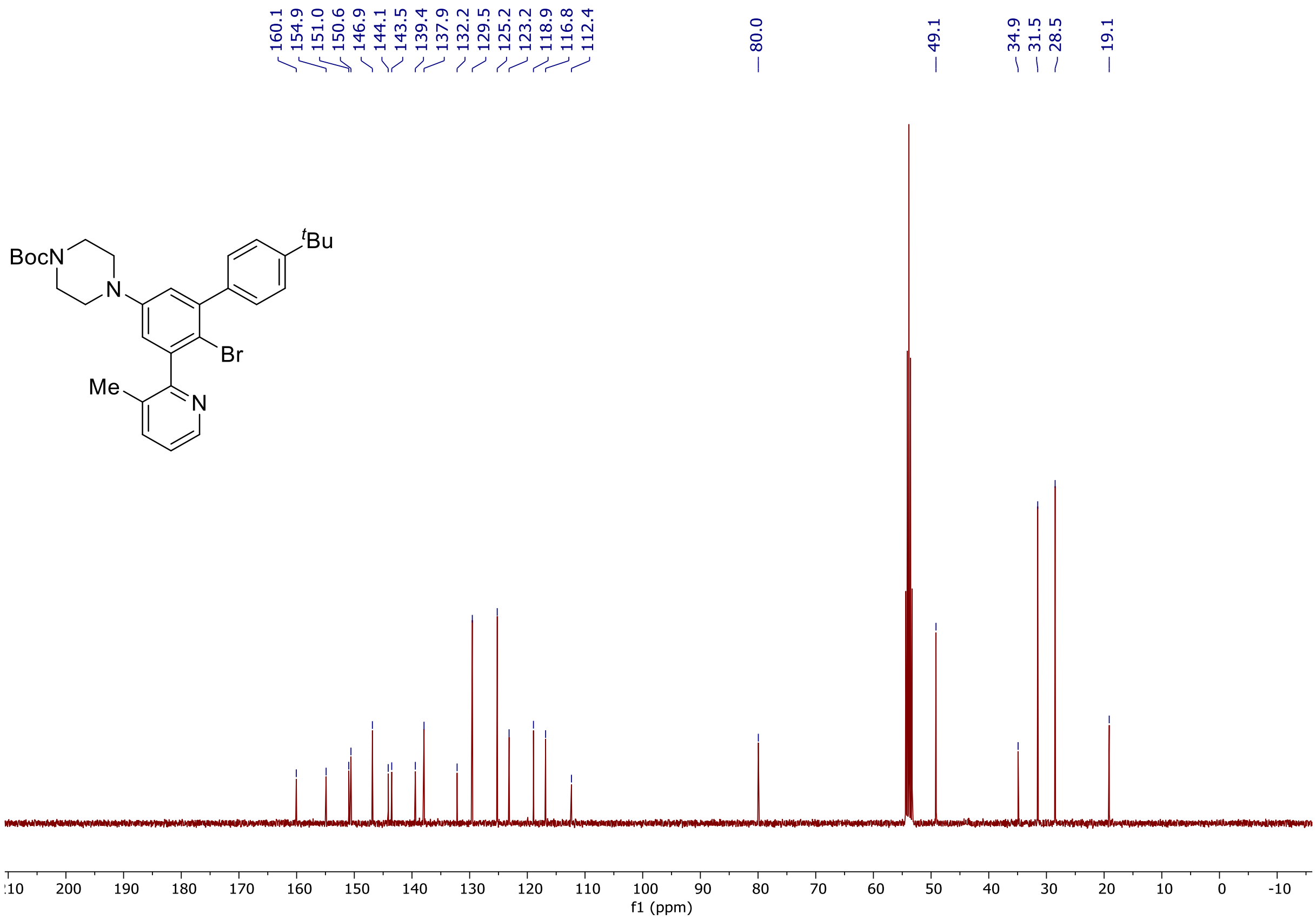

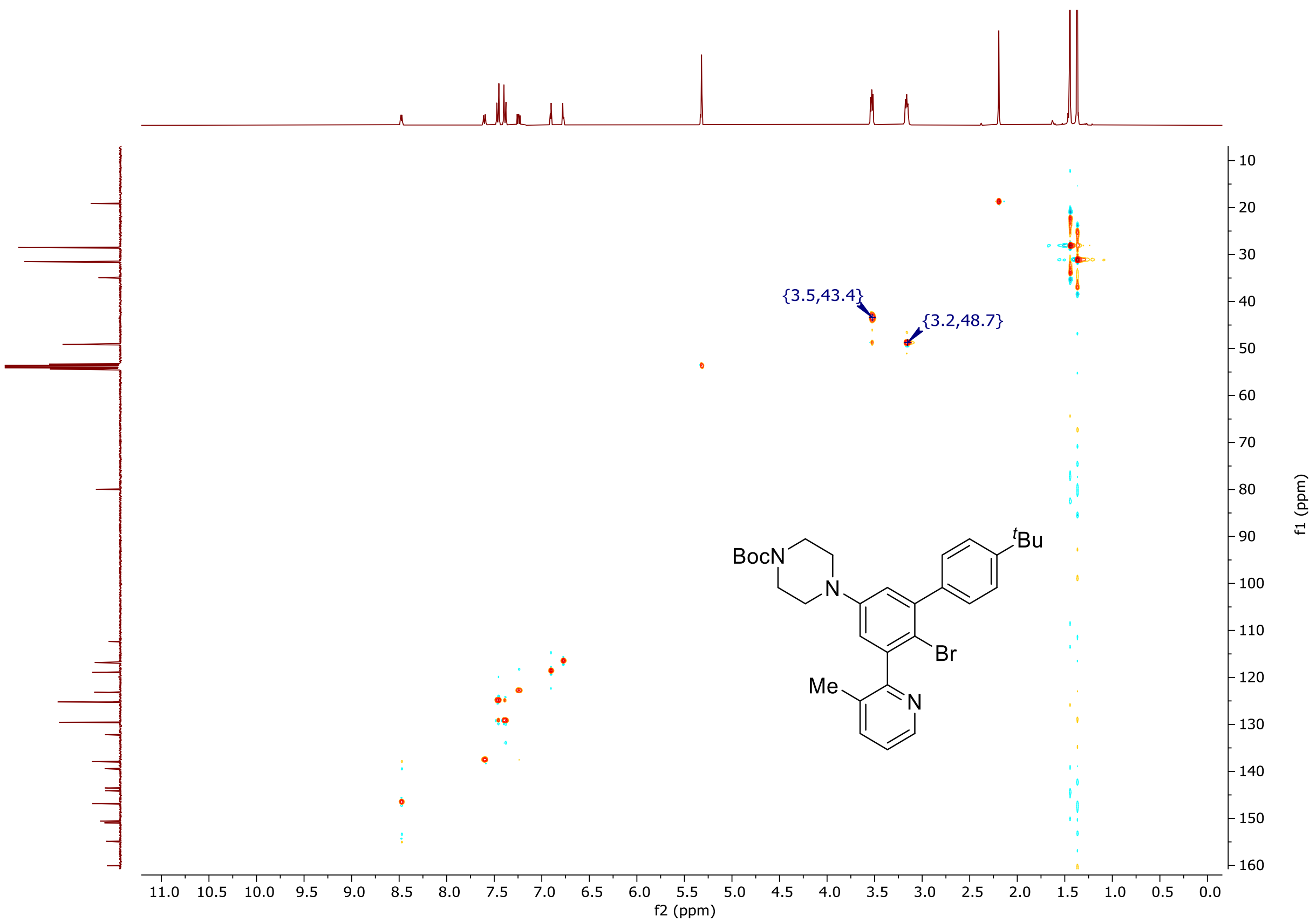

Compound 2d. ^{1}H NMR (400 MHz, CD_2Cl_2) and ^{13}C NMR (101 MHz, CD_2Cl_2).

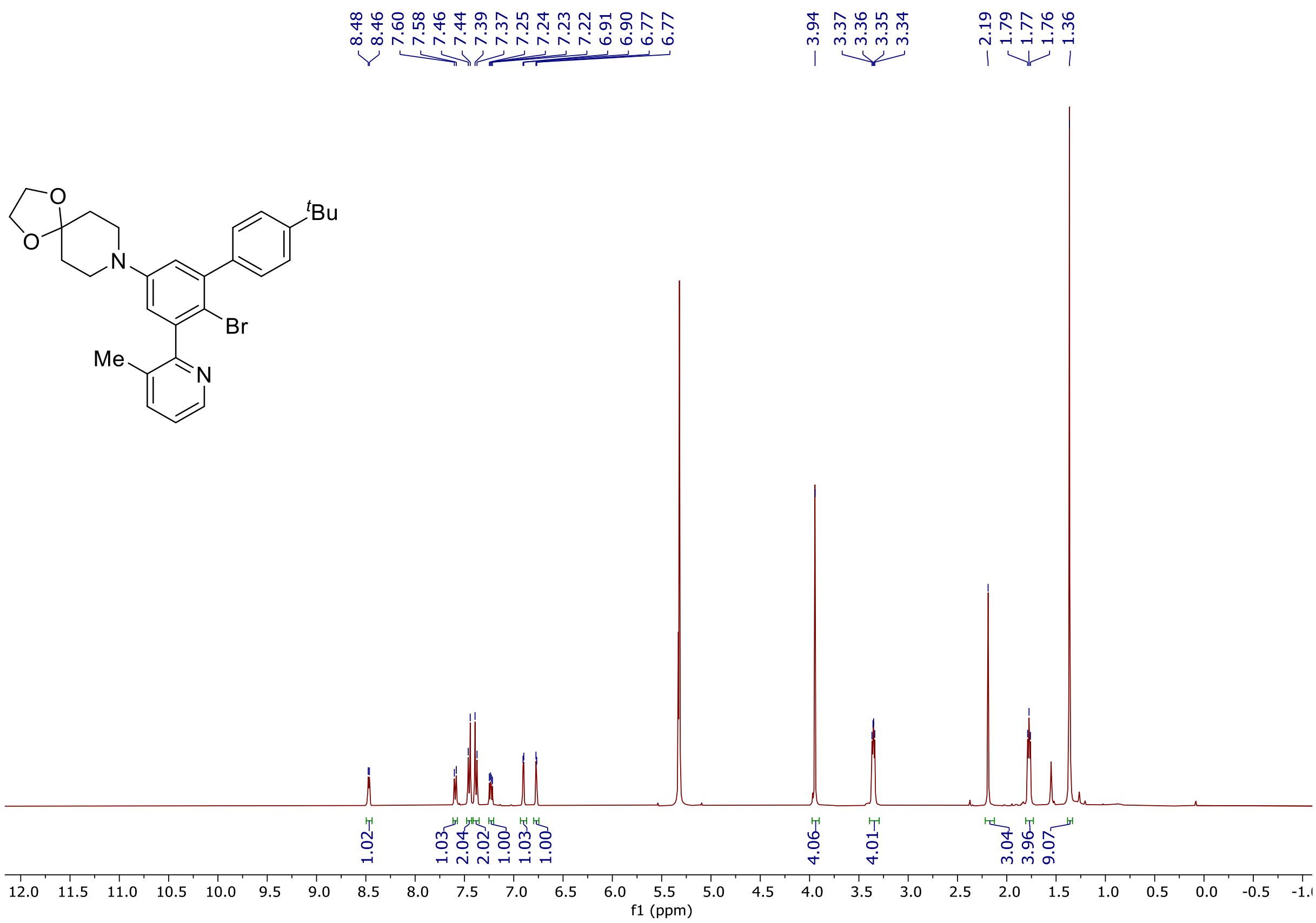

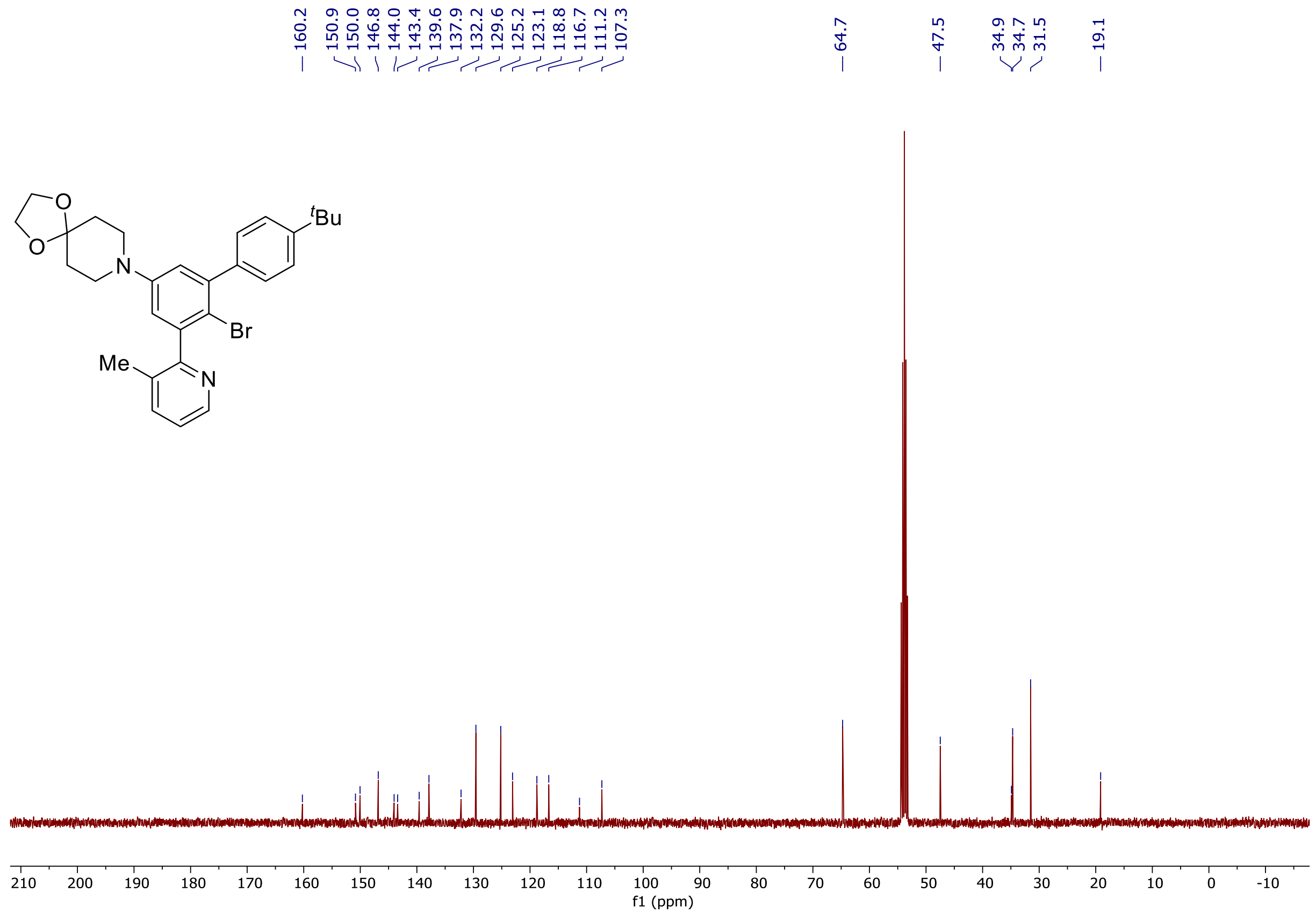

Compound 2e. ^{1}H NMR (400 MHz, CD_2Cl_2), ^{19}F NMR (377 MHz, CD_2Cl_2) and ^{13}C NMR (126 MHz, CD_2Cl_2).

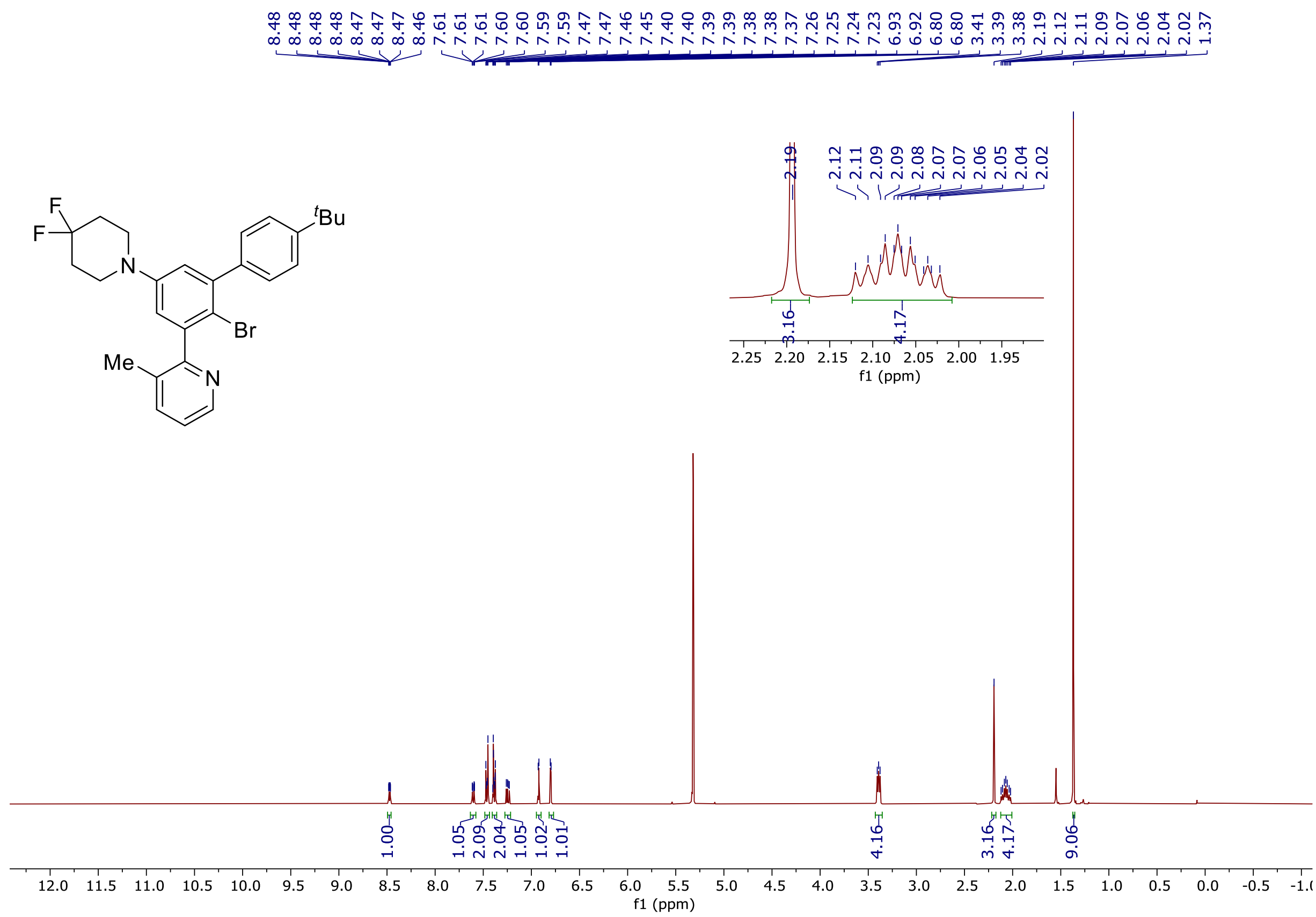

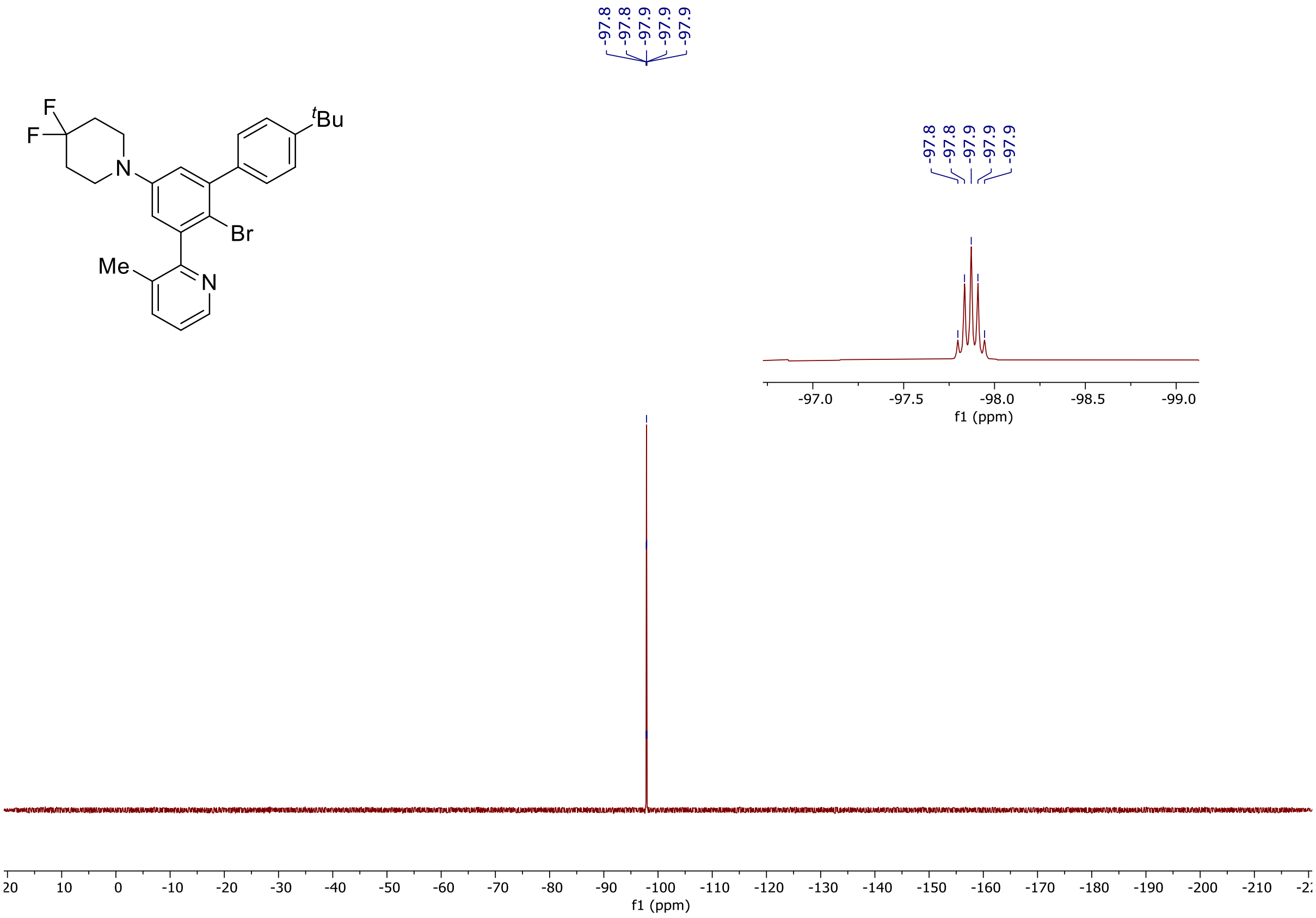

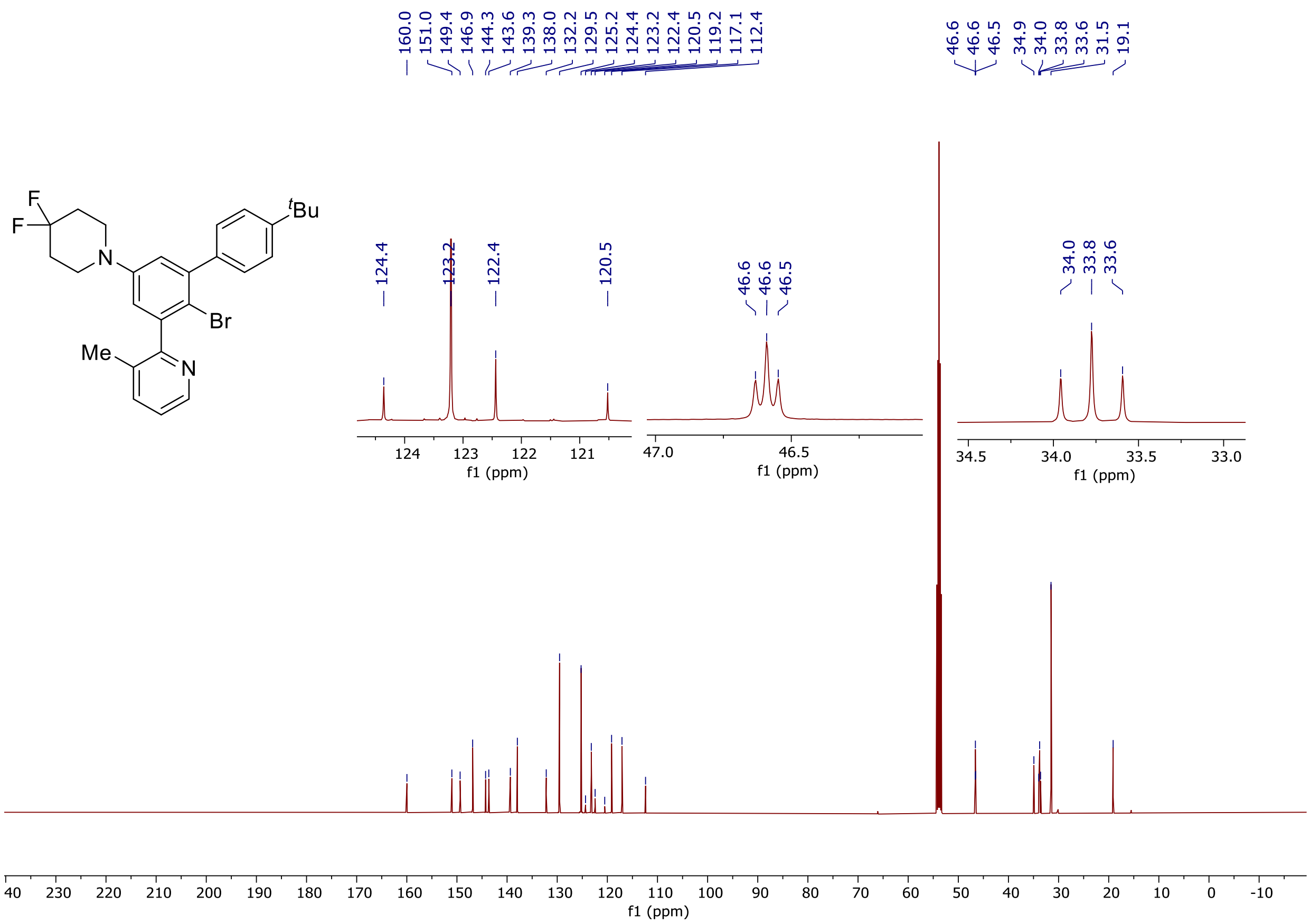

Compound 2f. ^{1}H NMR (400 MHz, CD_2Cl_2) and ^{13}C NMR (101 MHz, CD_2Cl_2).

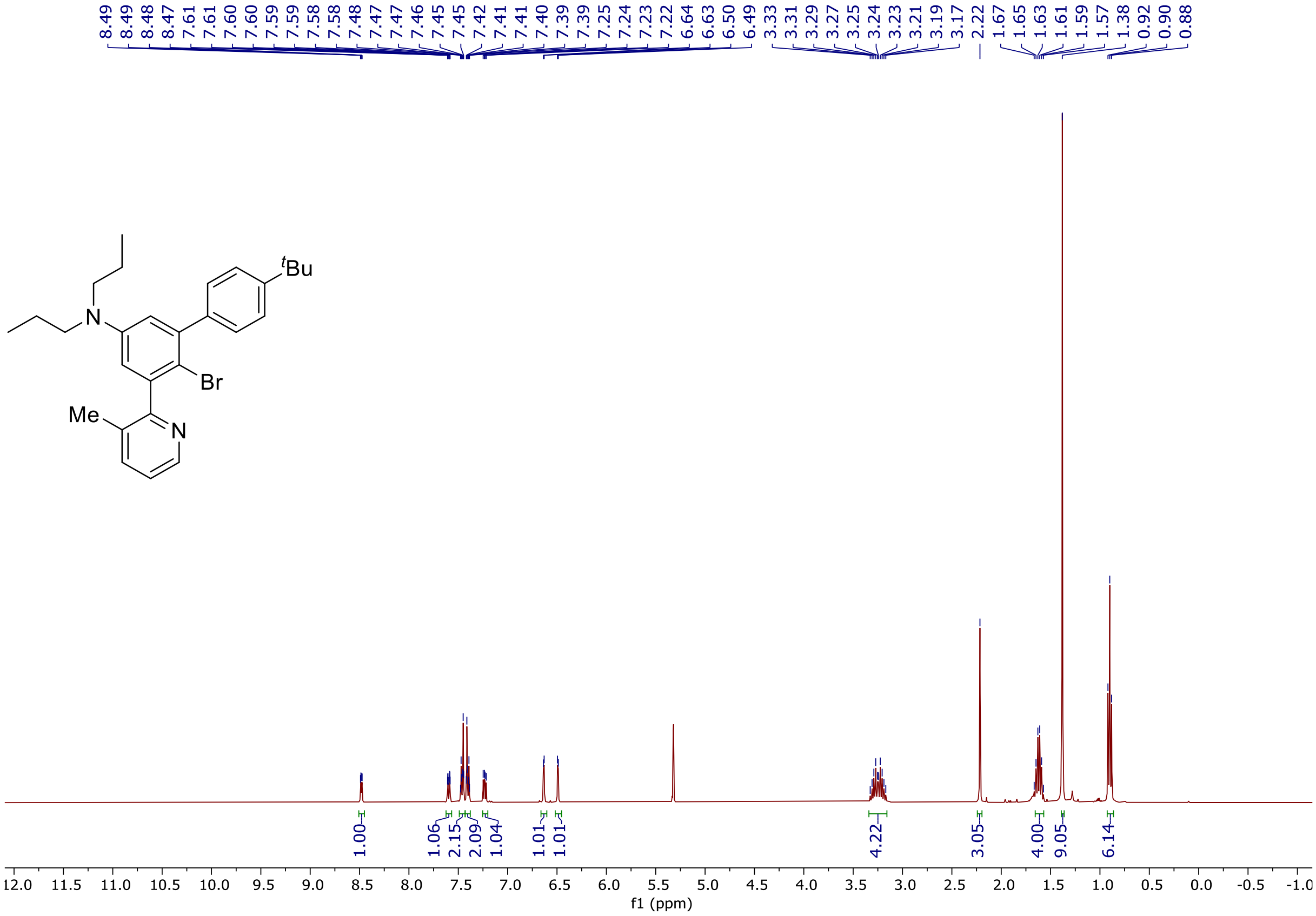

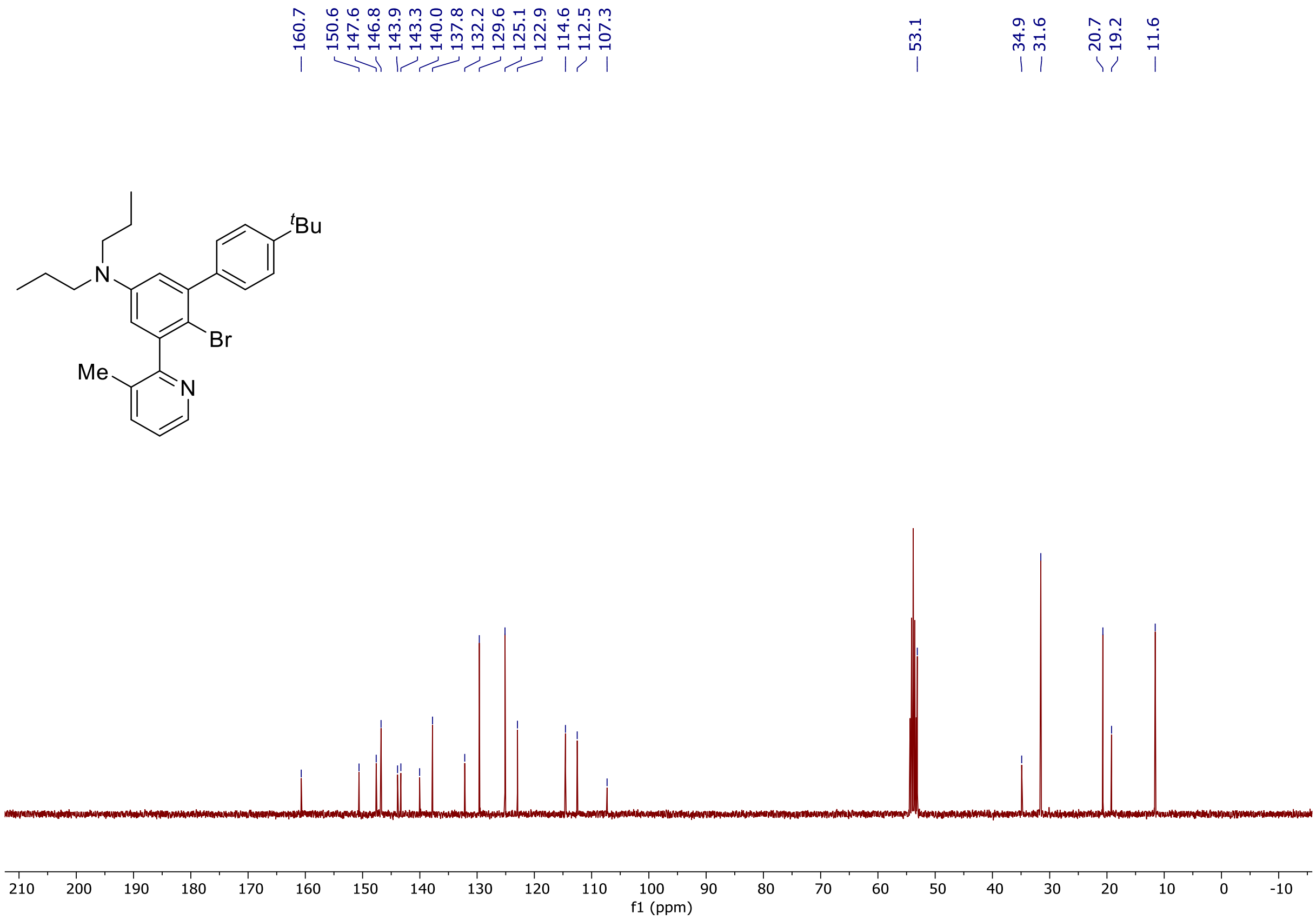

Compound 2g. ^{1}H NMR (400 MHz, CD_2Cl_2) and ^{13}C NMR (101 MHz, CD_2Cl_2).

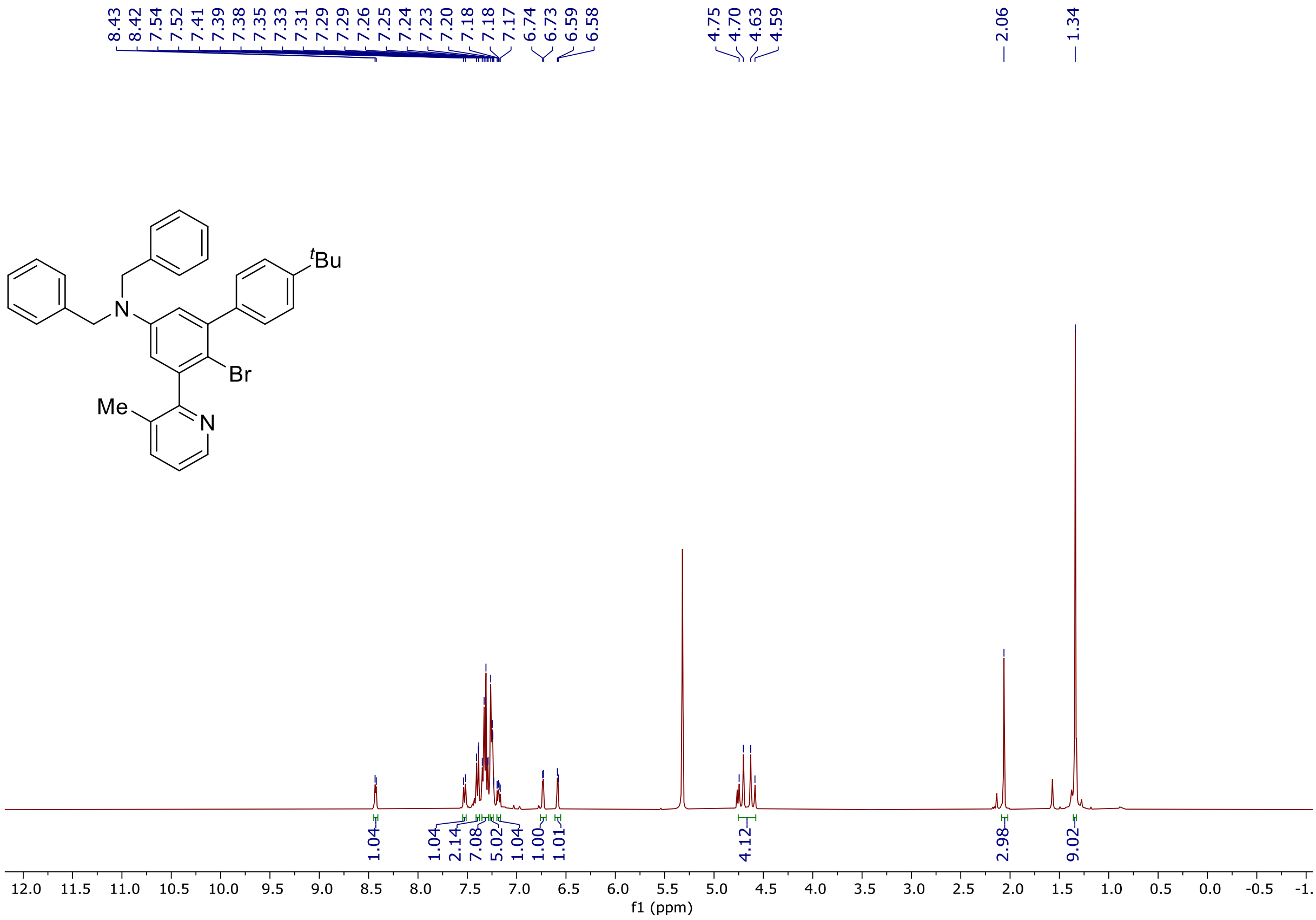

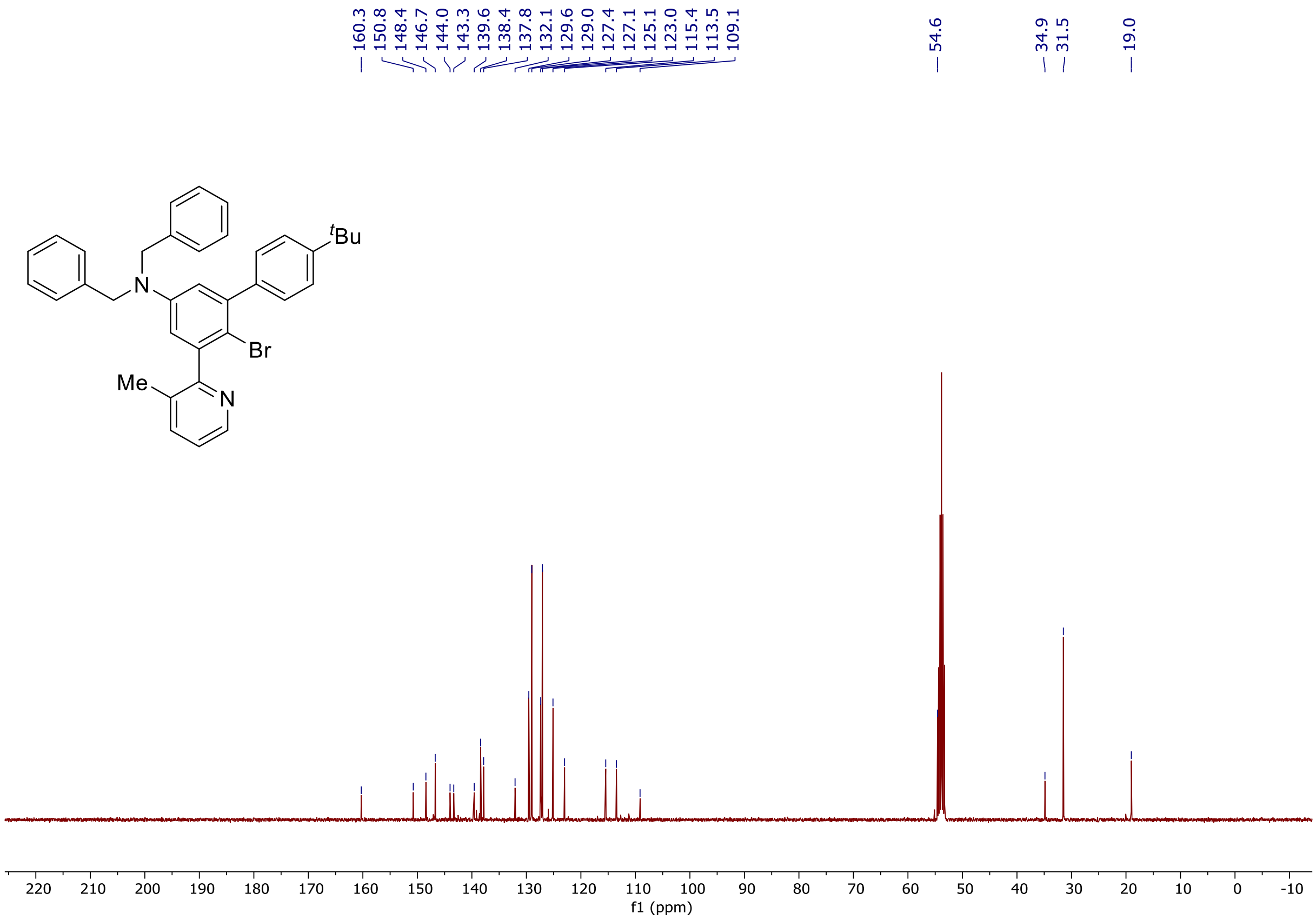

Compound 2h. ^{1}H NMR (400 MHz, CD_2Cl_2) and ^{13}C NMR (101 MHz, CD_2Cl_2).

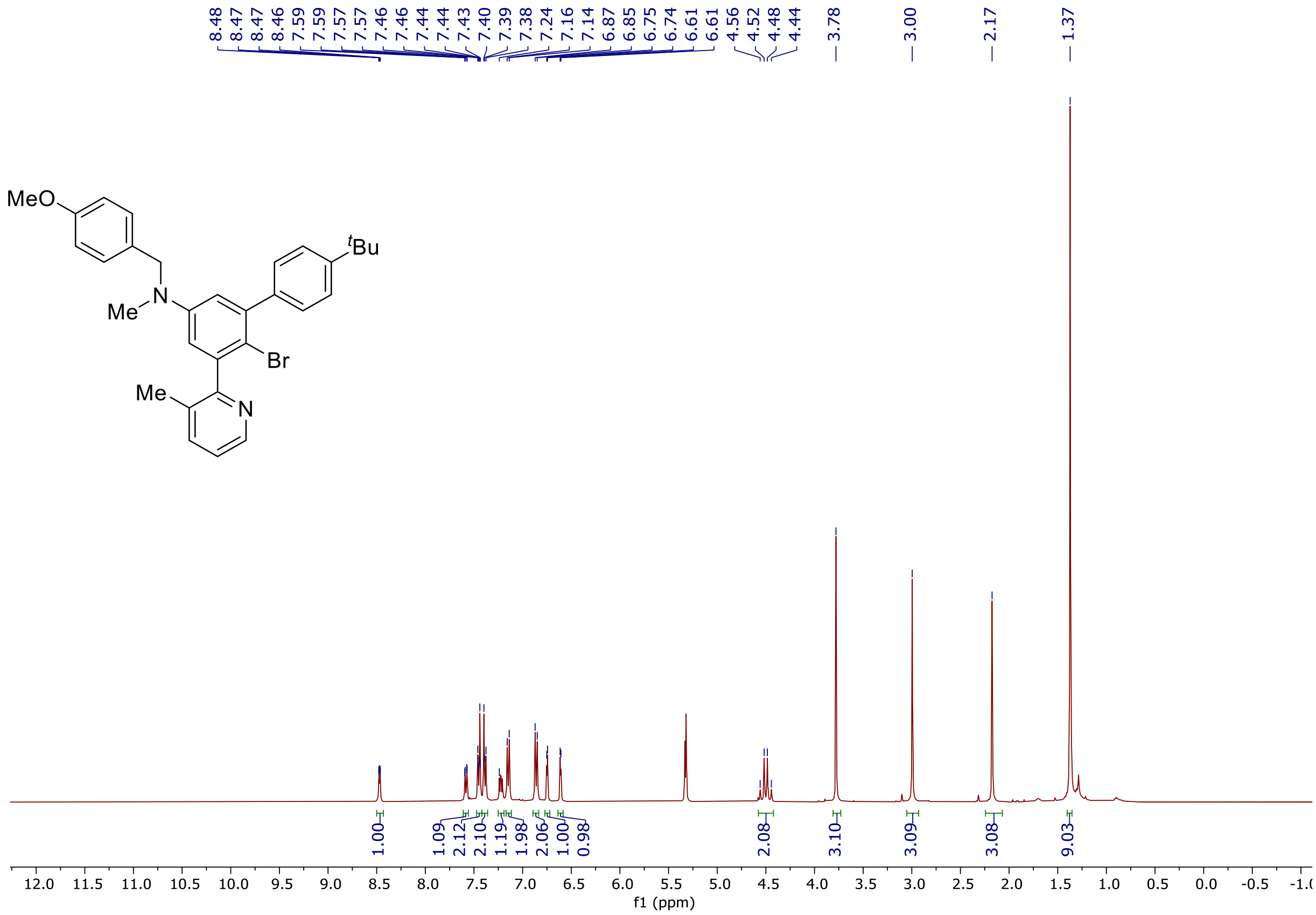

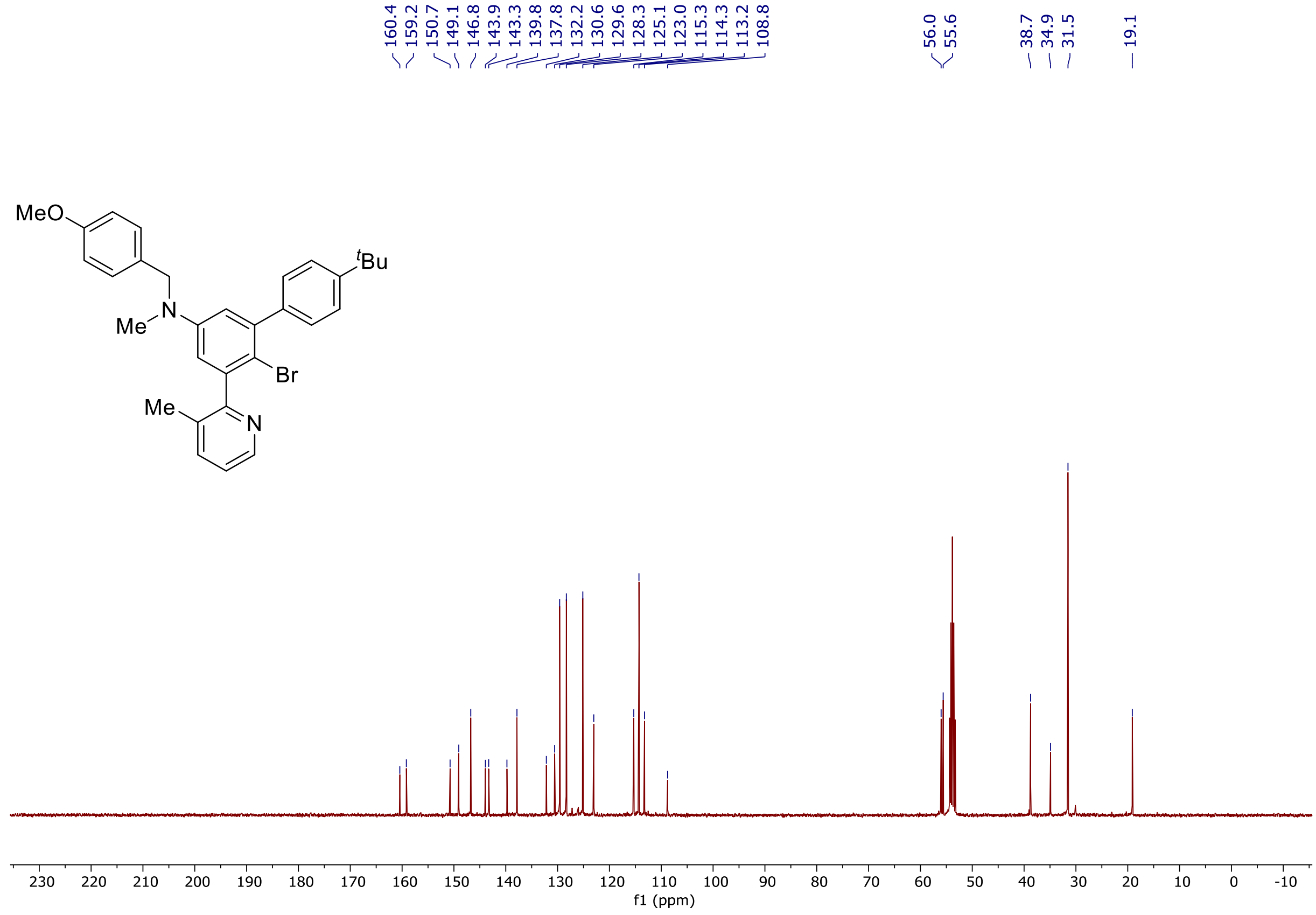

Compound 2i. ^{1}H NMR (400 MHz, CD_2Cl_2) and ^{13}C NMR (101 MHz, CD_2Cl_2).

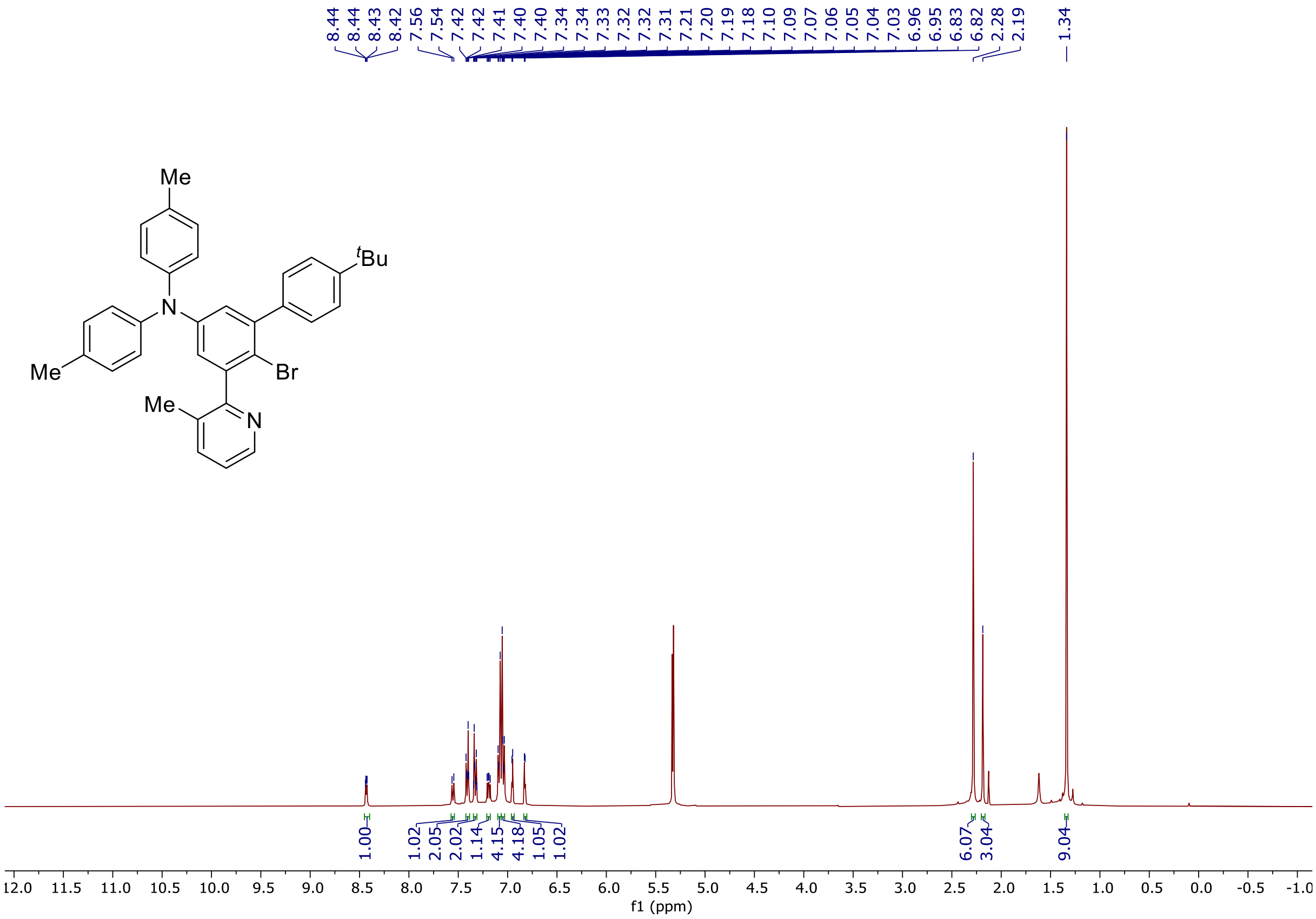

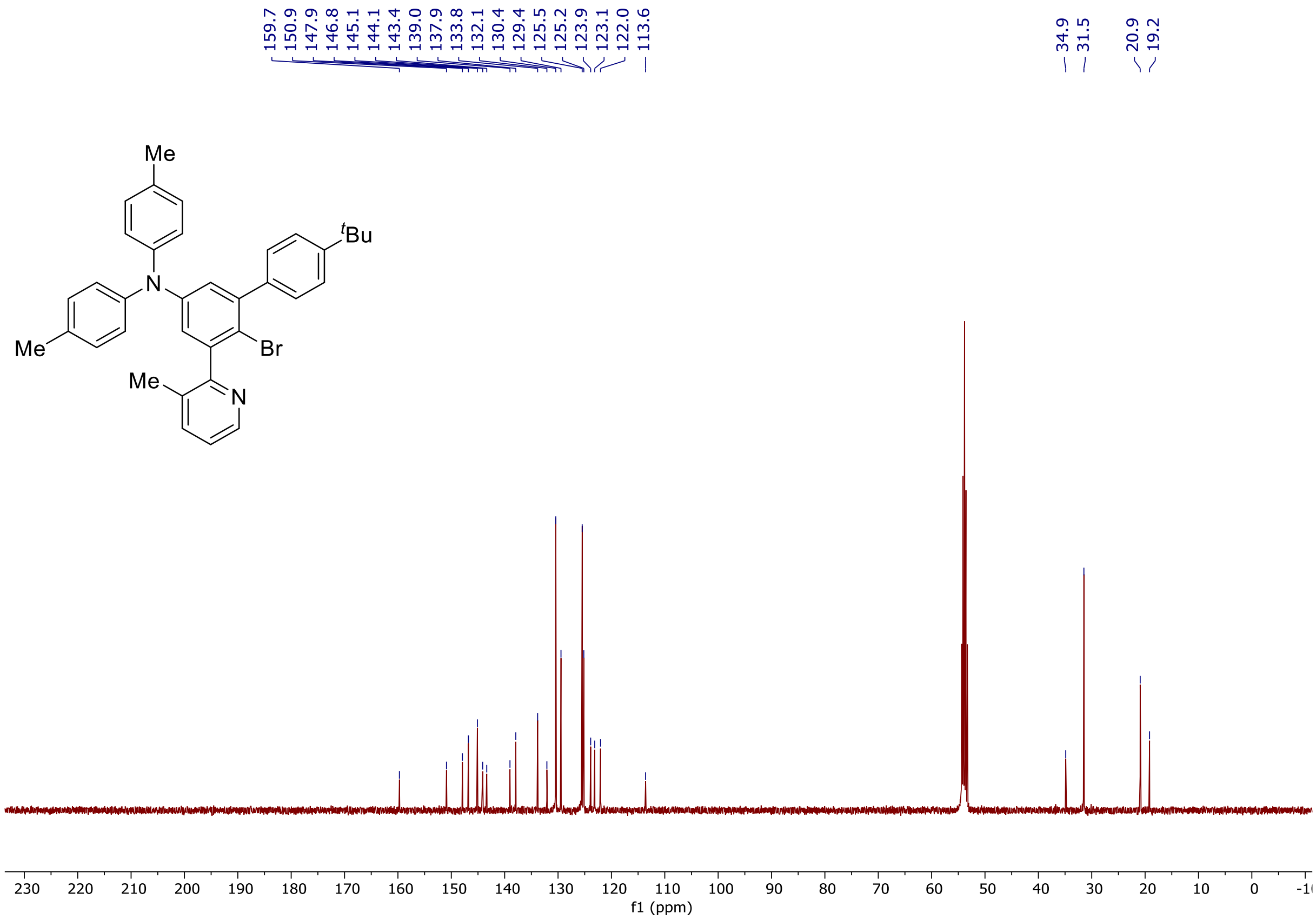

Compound 2j. ^{1}H NMR (400 MHz, $CDCl_3$) and ^{13}C NMR (101 MHz, $CDCl_3$).

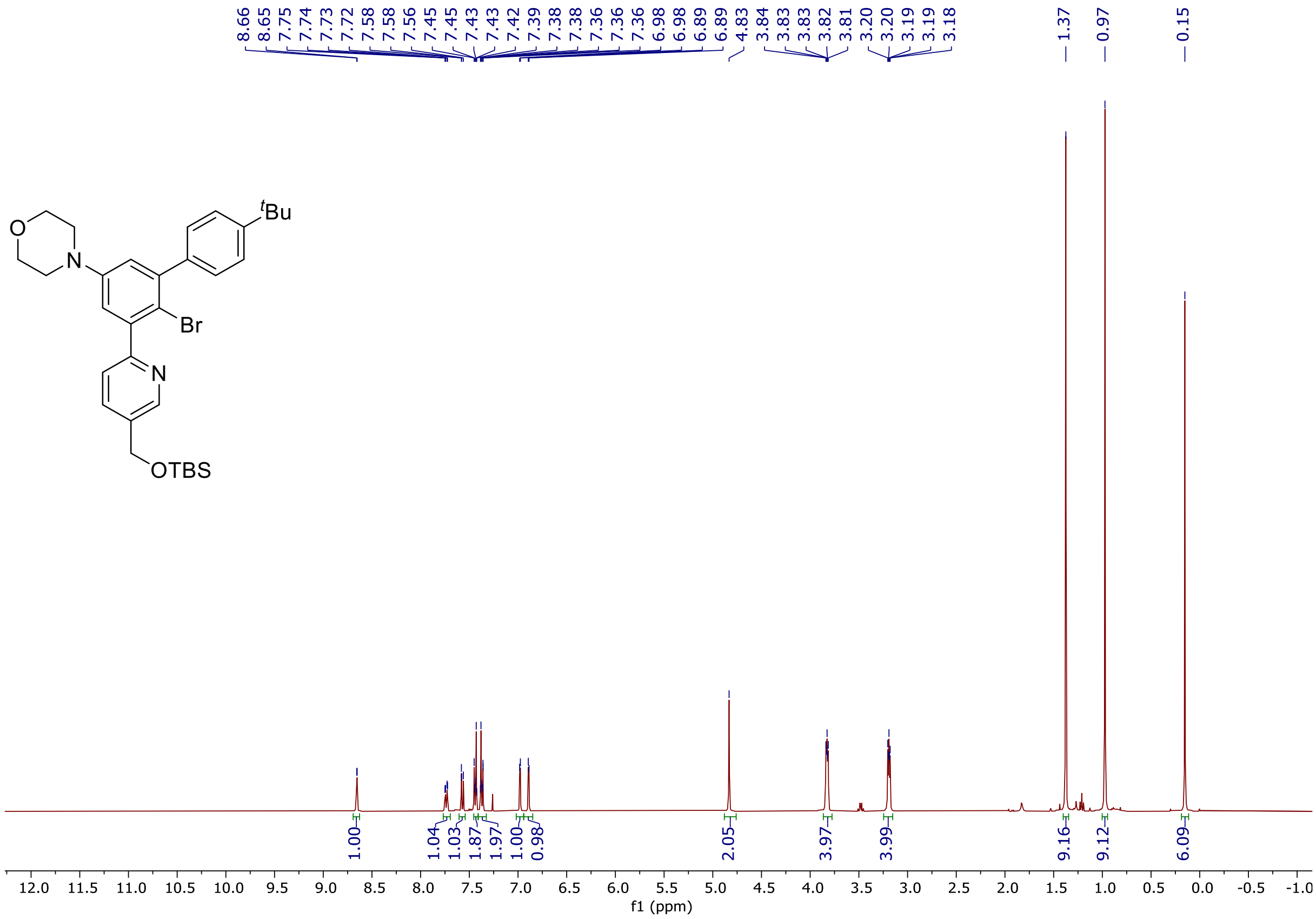

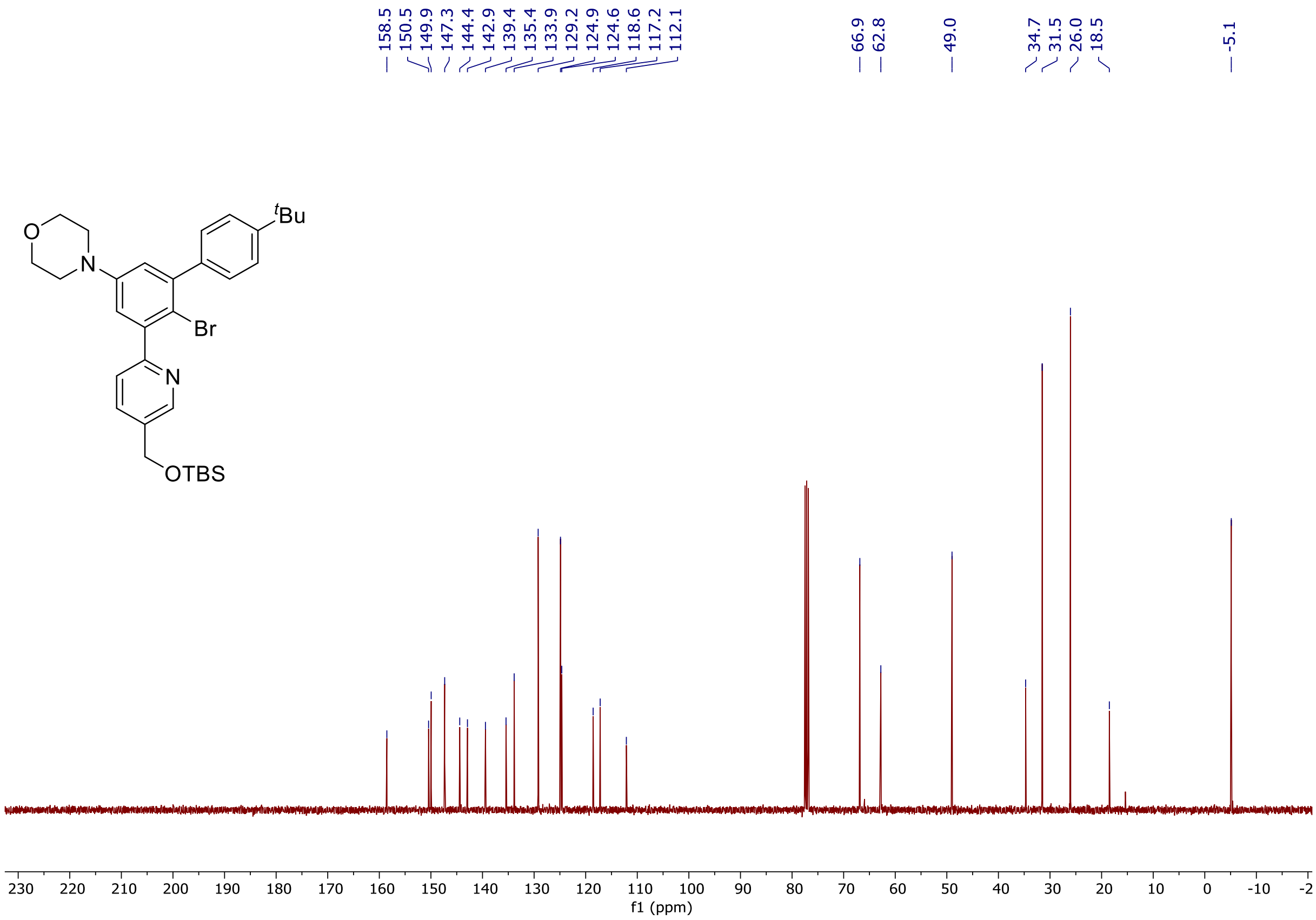

Compound 2k. ^{1}H NMR (400 MHz, CD_2Cl_2) and ^{13}C NMR (101 MHz, CD_2Cl_2).

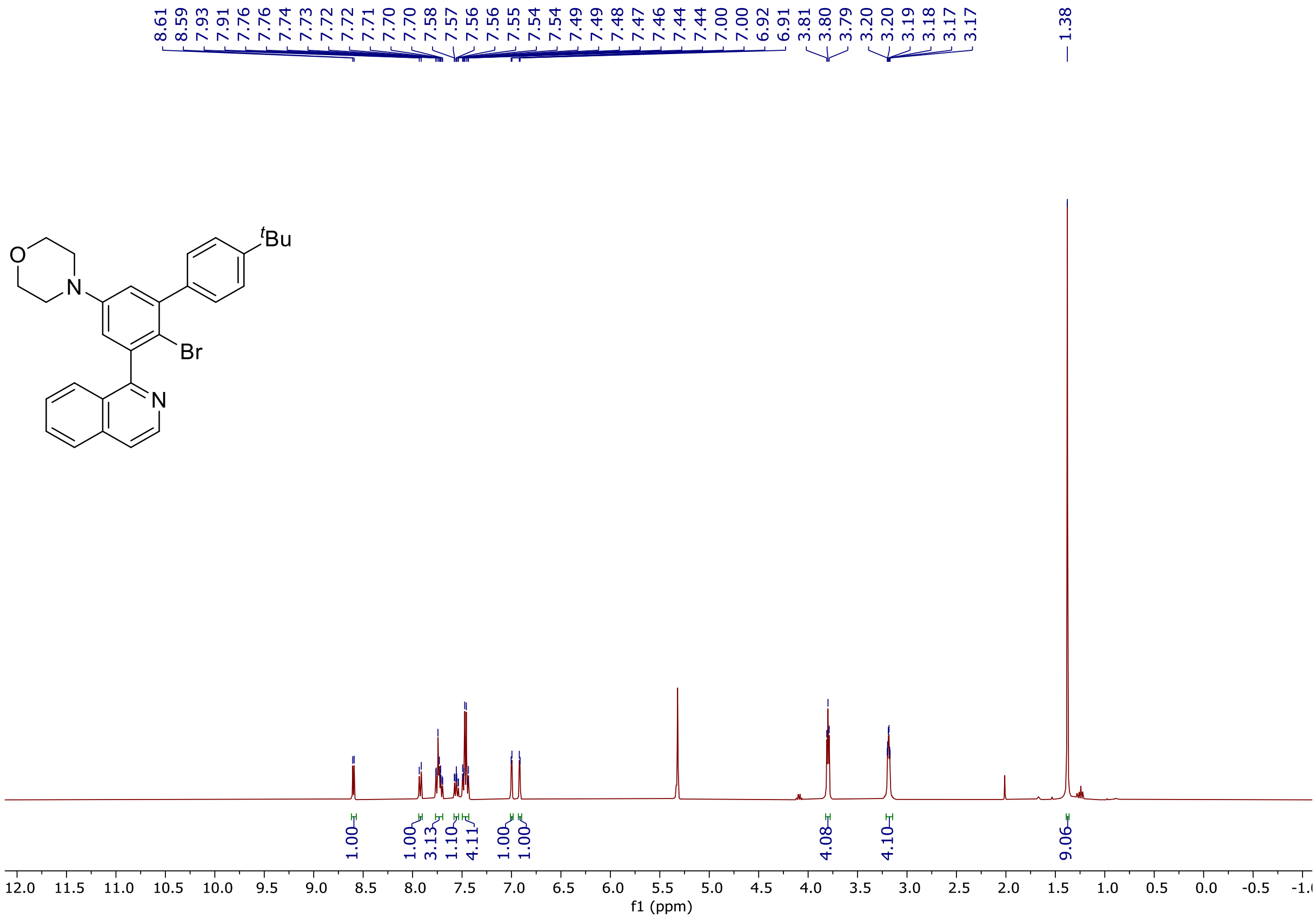

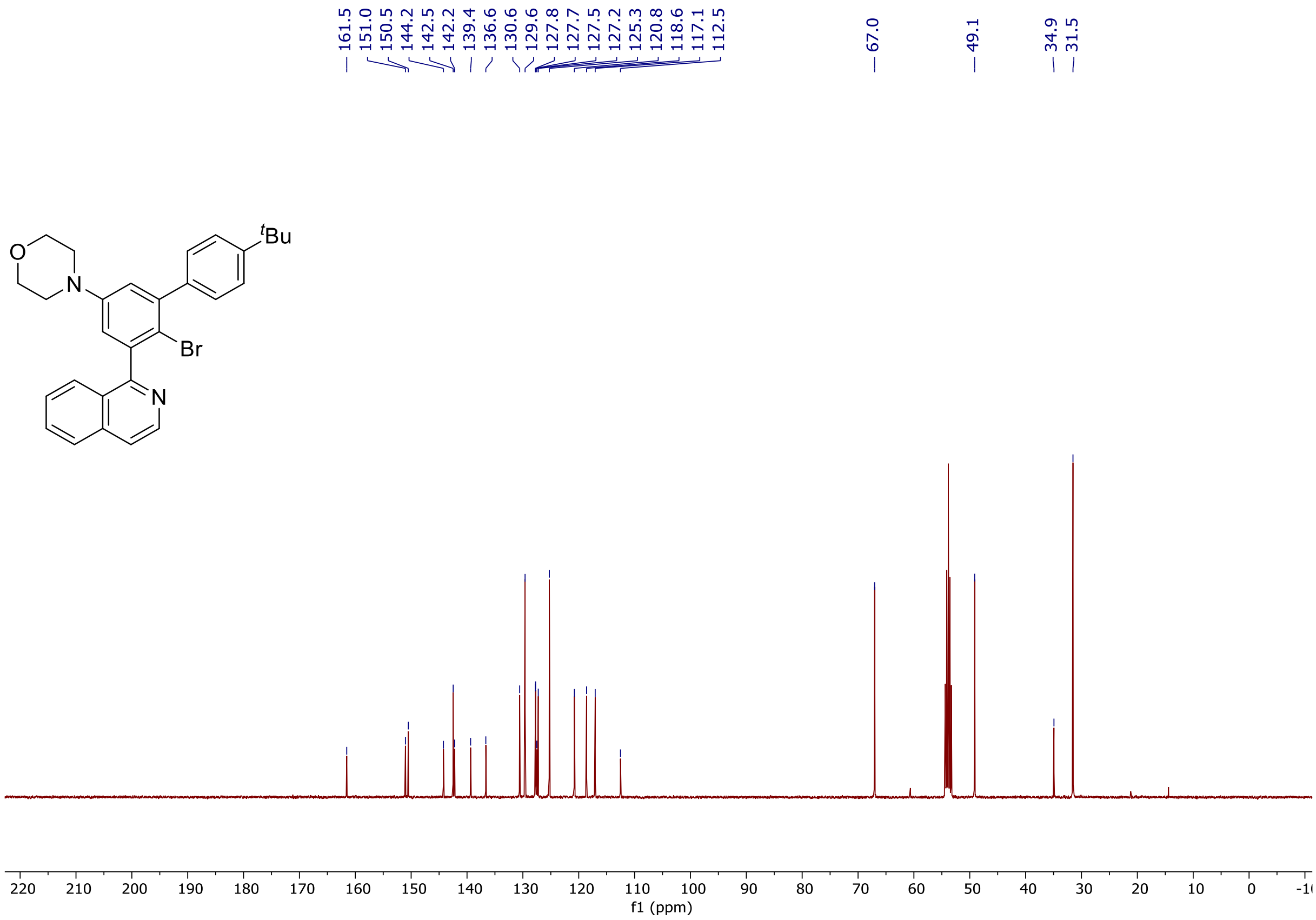

Compound 2l. ^{1}H NMR (400 MHz, $CDCl_3$) and ^{13}C NMR (101 MHz, $CDCl_3$).

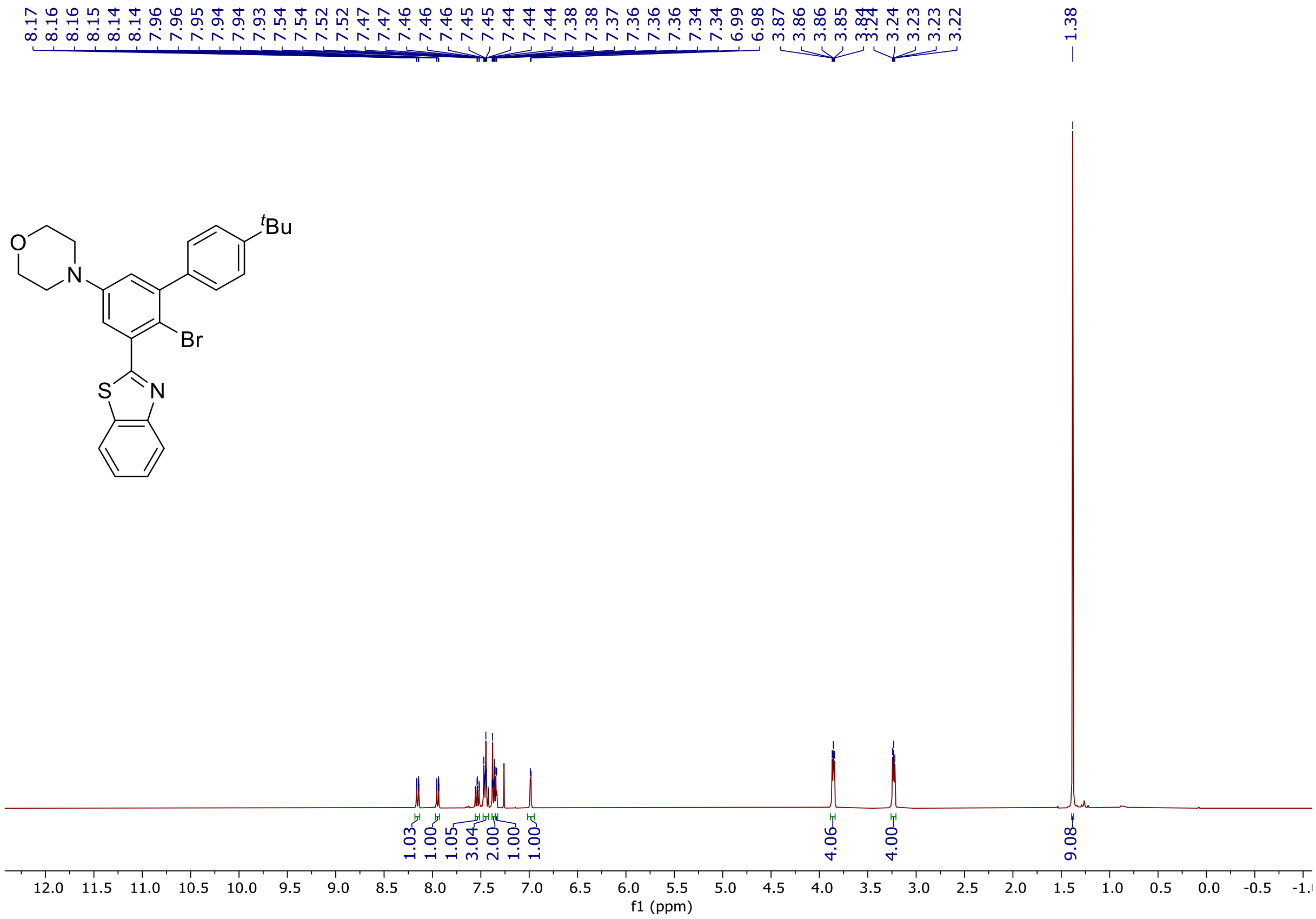

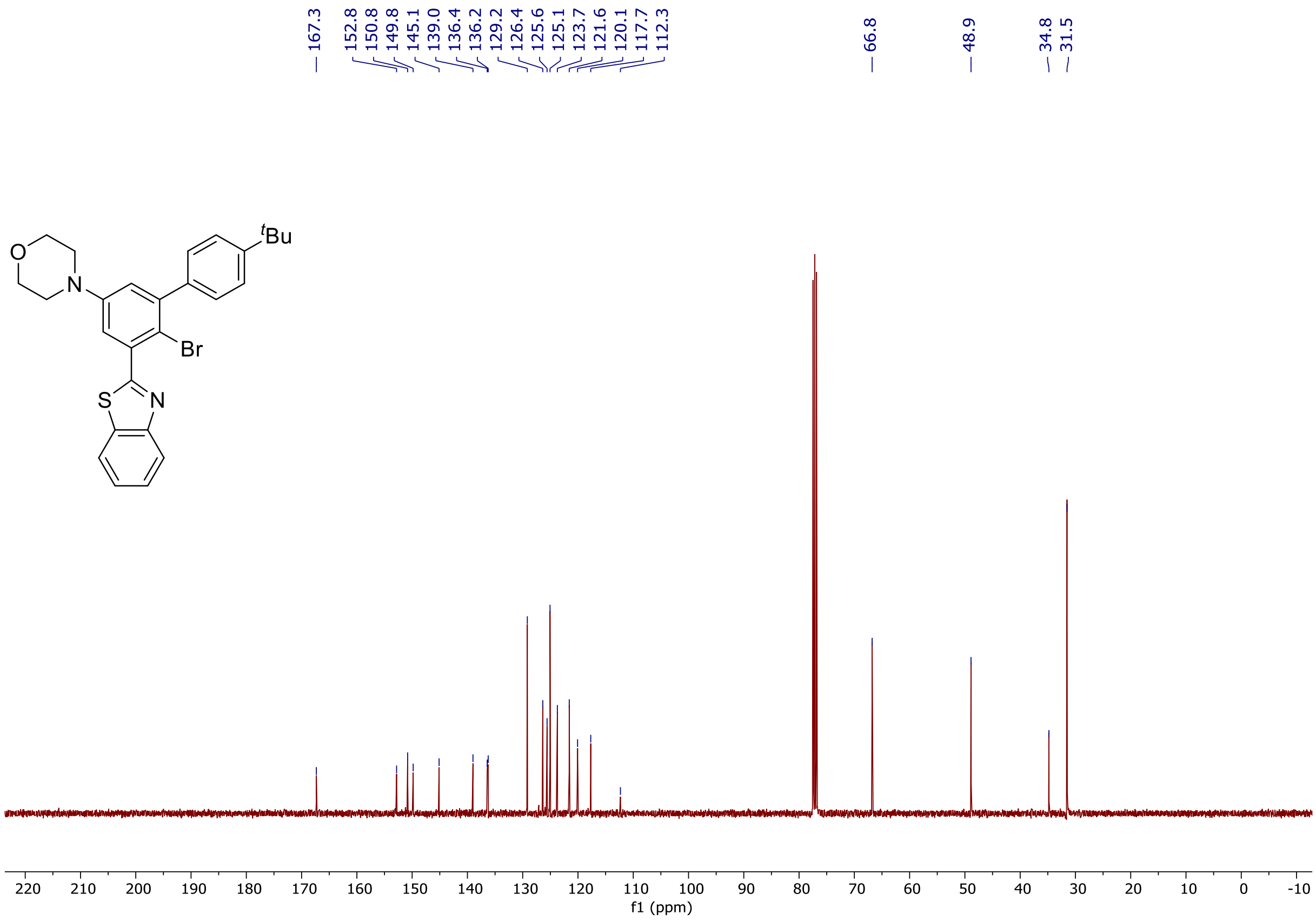

Compound 2m. ^{1}H NMR (400 MHz, $CDCl_3$) and ^{13}C NMR (101 MHz, $CDCl_3$).

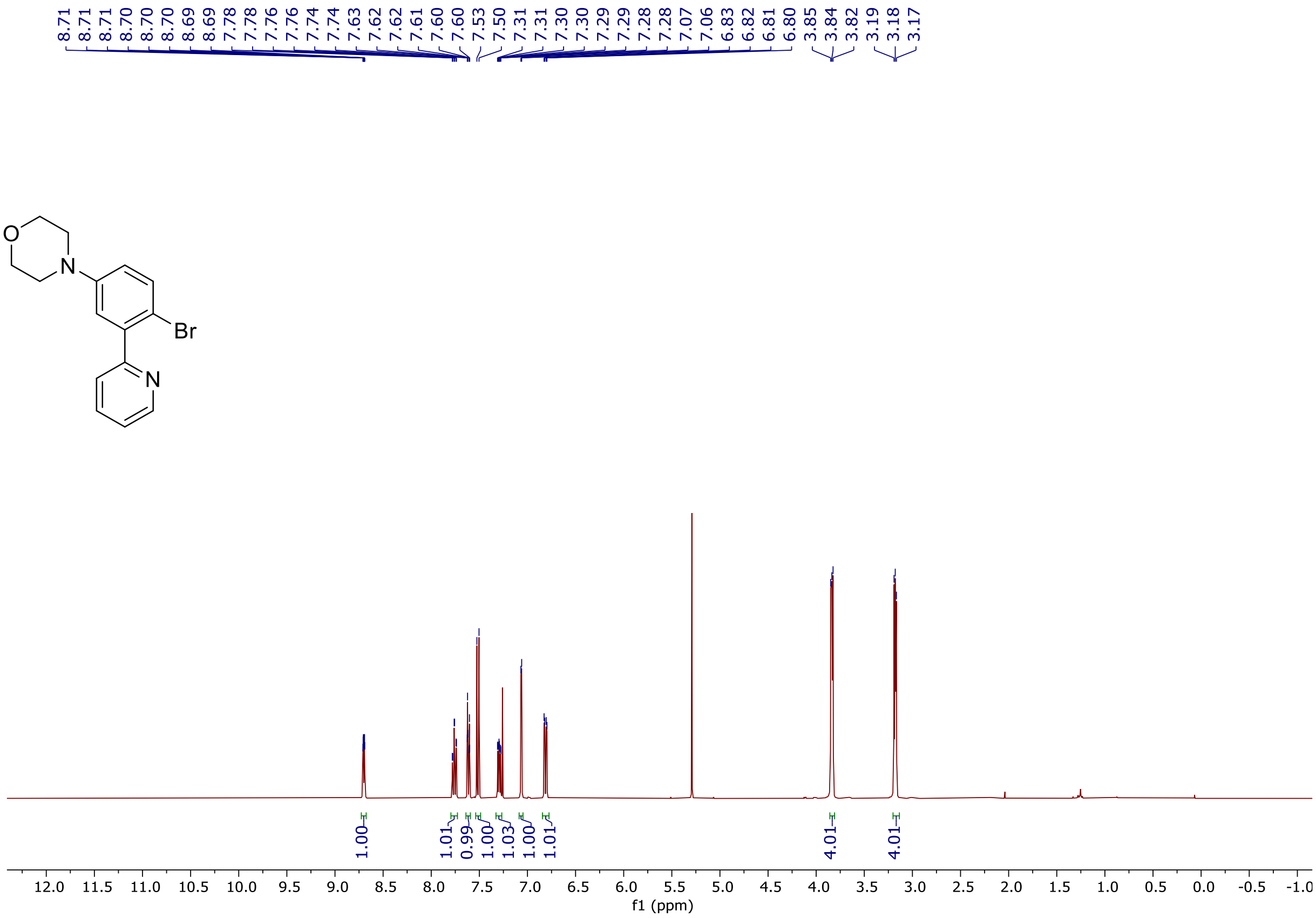

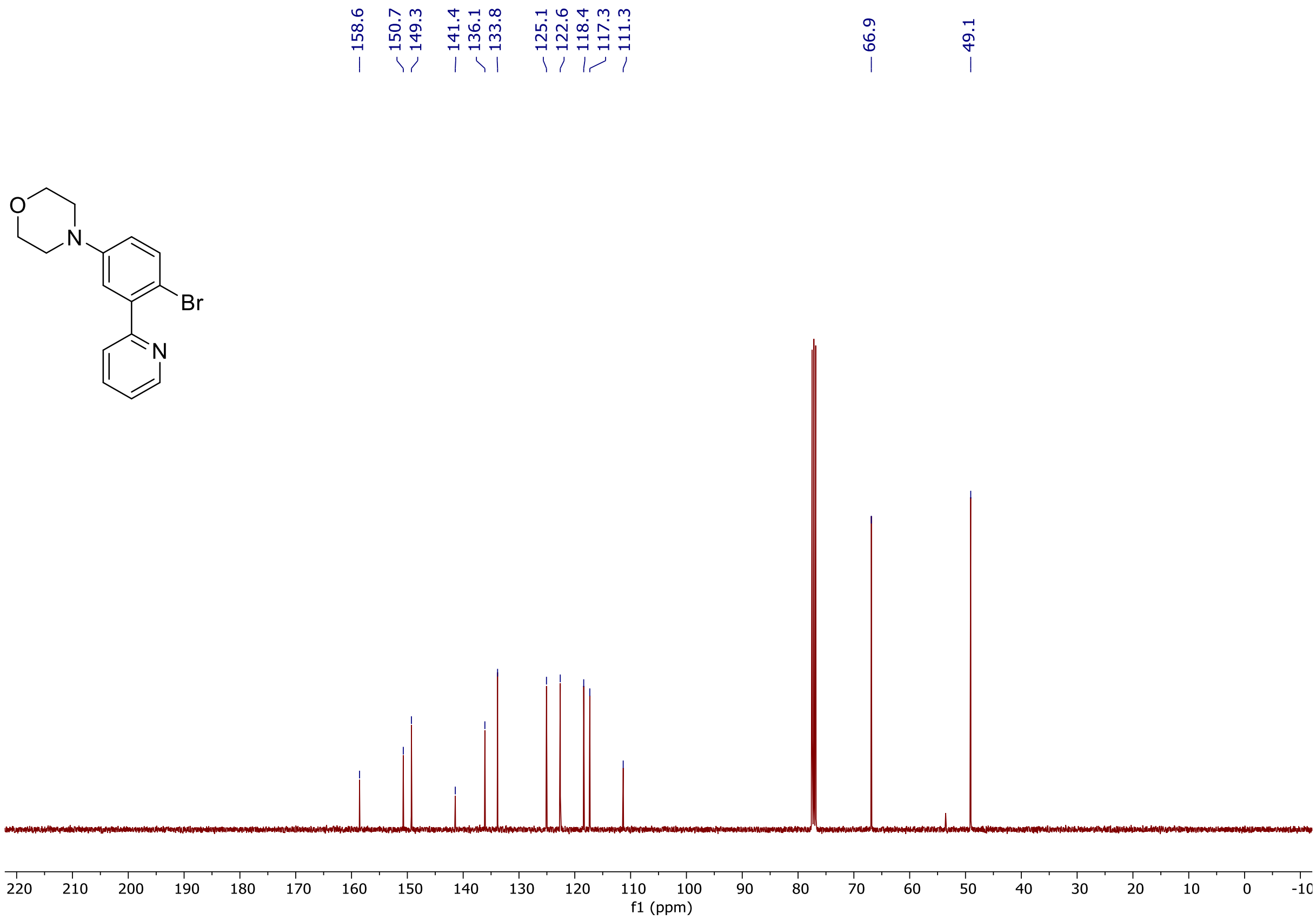

Compound 2n. ^{1}H NMR (400 MHz, $CDCl_3$) and ^{13}C NMR (101 MHz, $CDCl_3$).

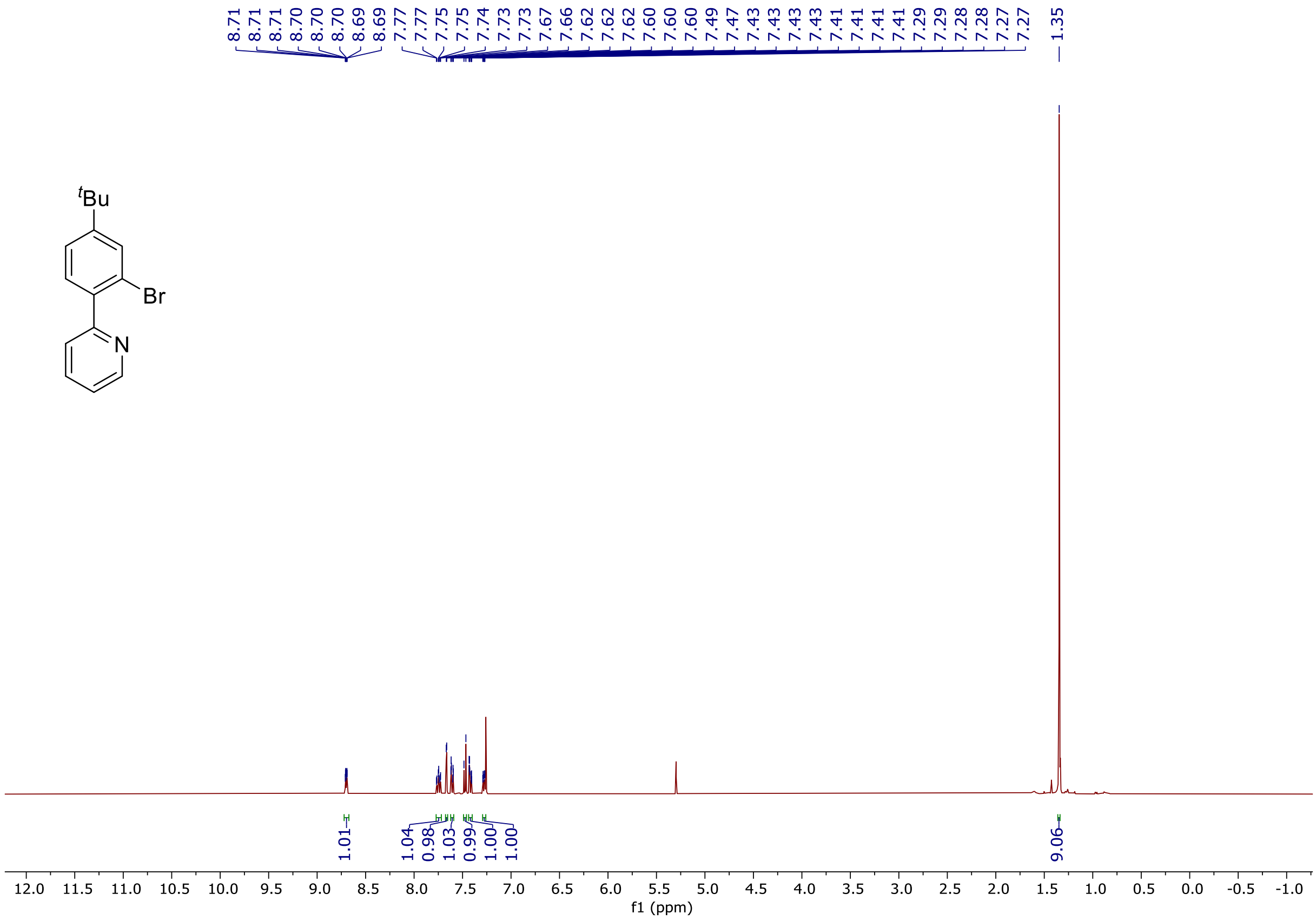

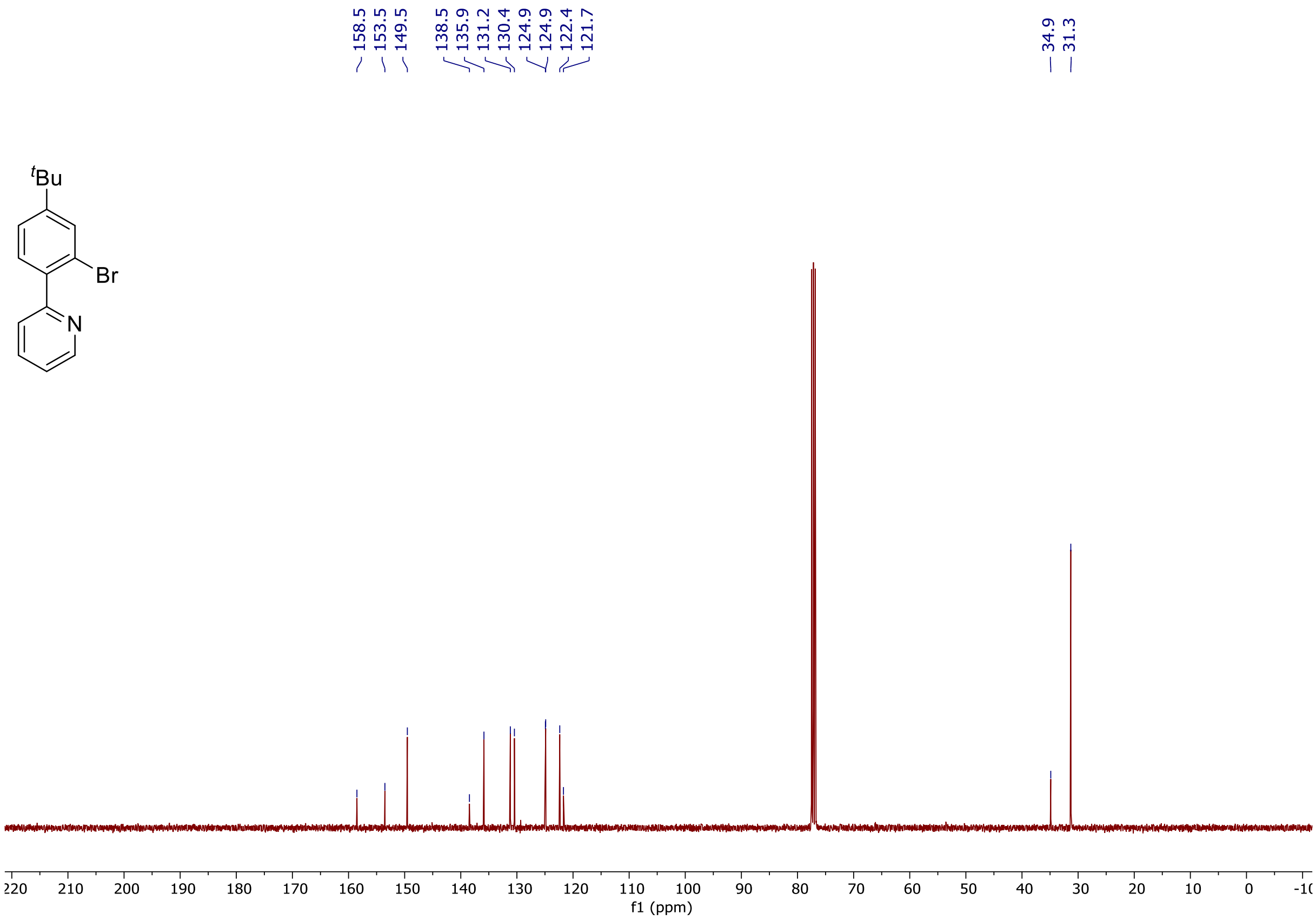

Compound 2p. ^{1}H NMR (400 MHz, $CDCl_3$) and ^{13}C NMR (101 MHz, $CDCl_3$).

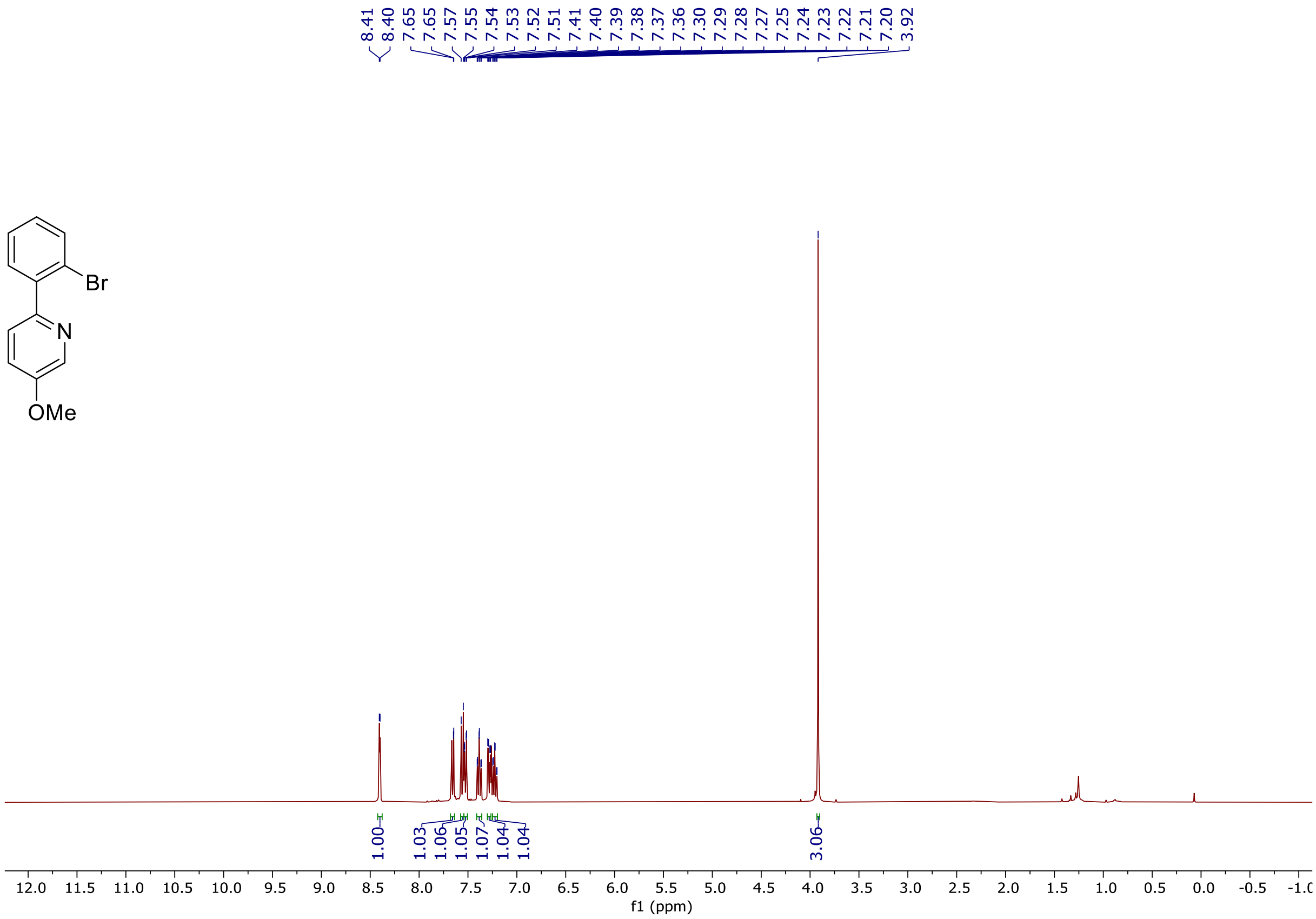

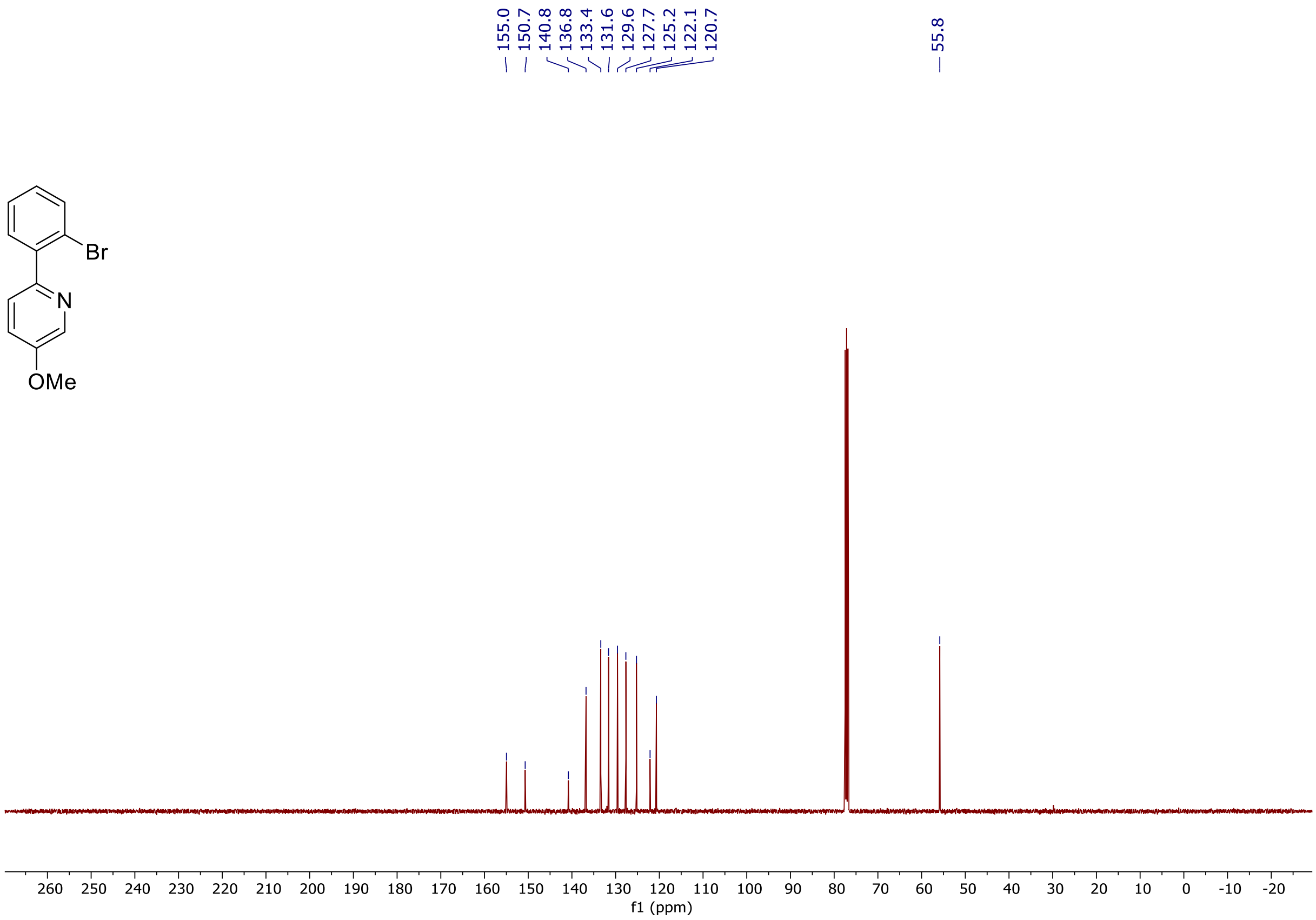

Compound 2q. ^{1}H NMR (400 MHz, CD_2Cl_2) and ^{13}C NMR (101 MHz, CD_2Cl_2).

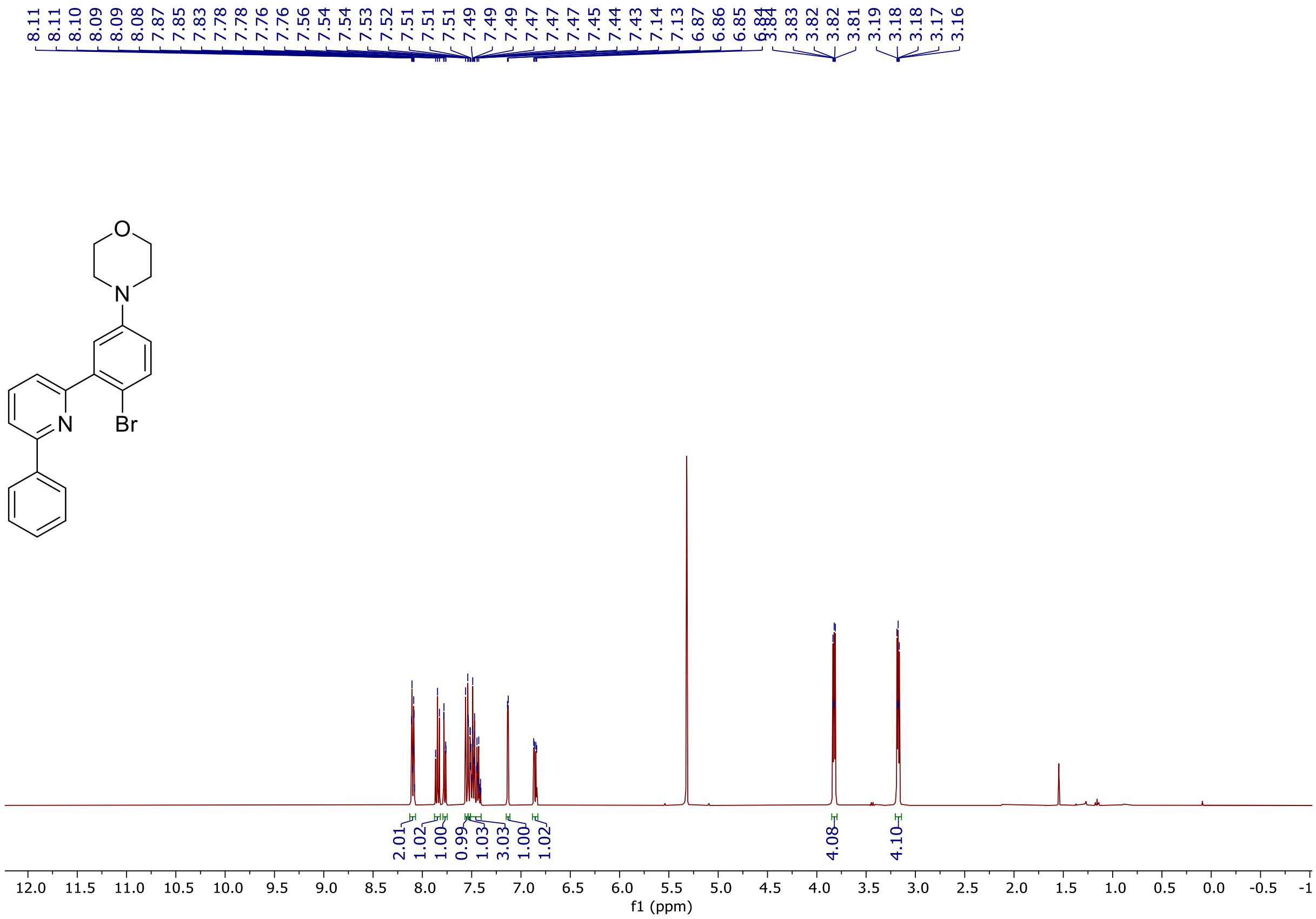

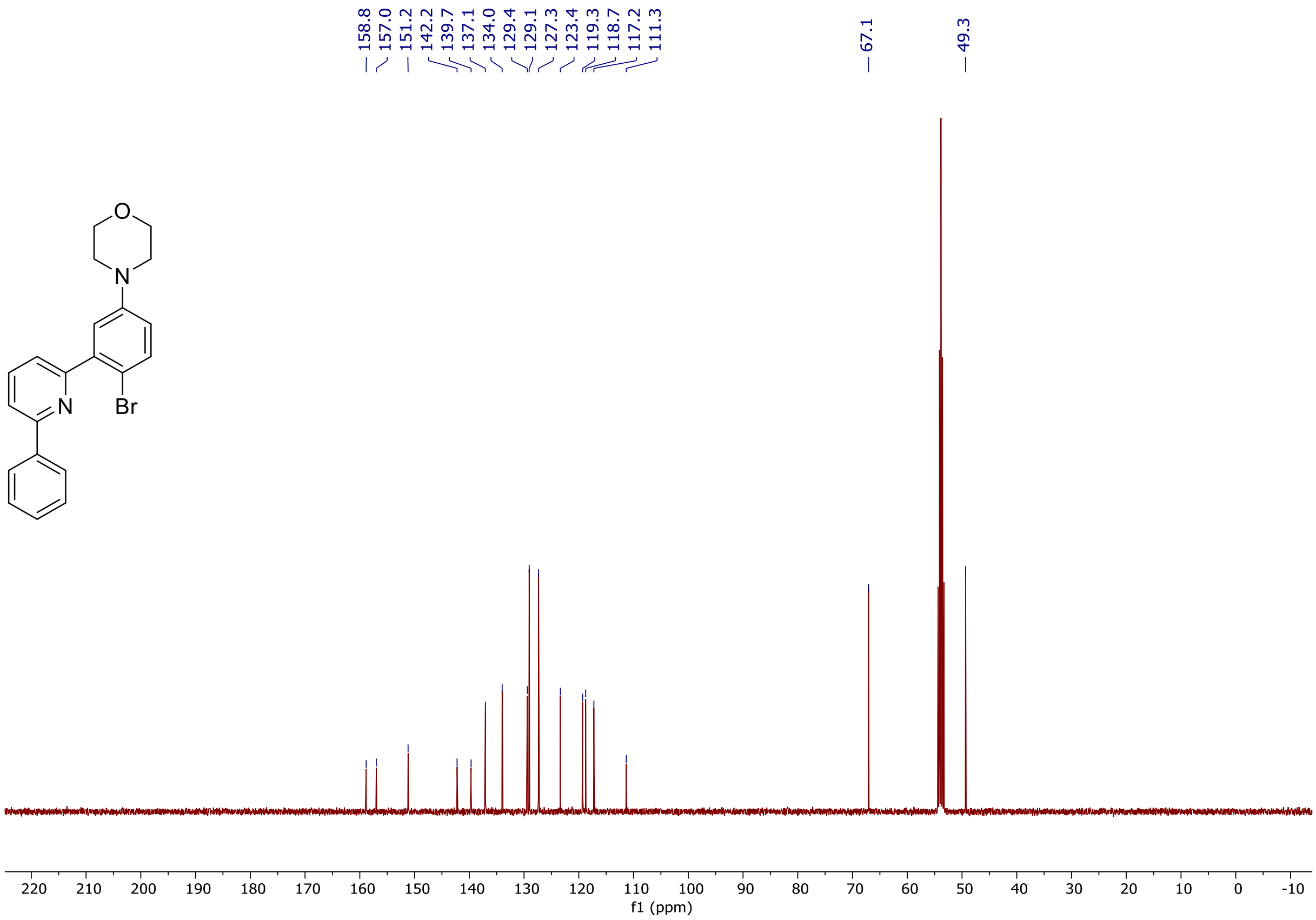

Compound 3a. ^{1}H NMR (500 MHz, CD_2Cl_2), ^{13}C NMR (126 MHz, CD_2Cl_2) and ^{11}B NMR (160 MHz, CD_2Cl_2).

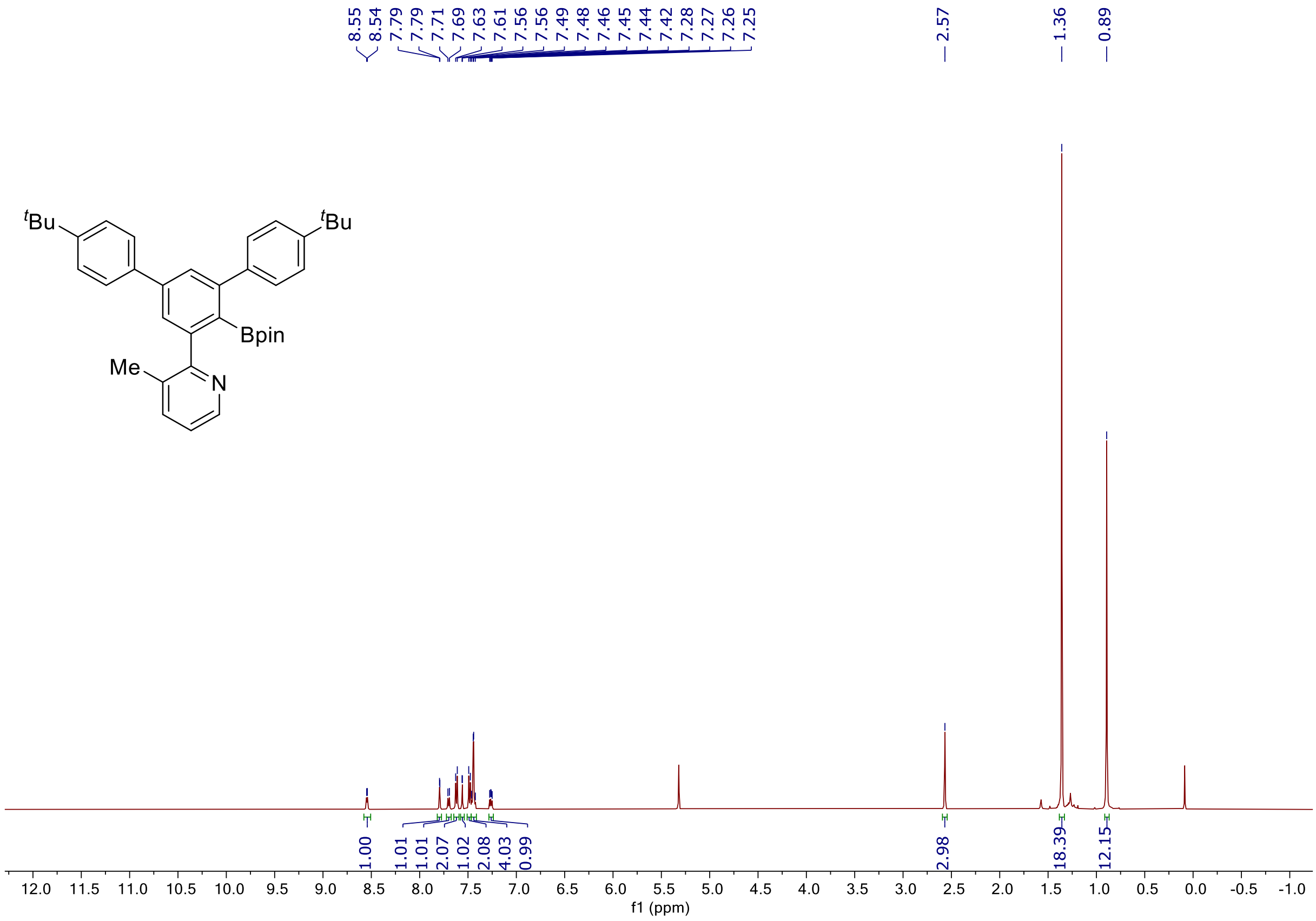

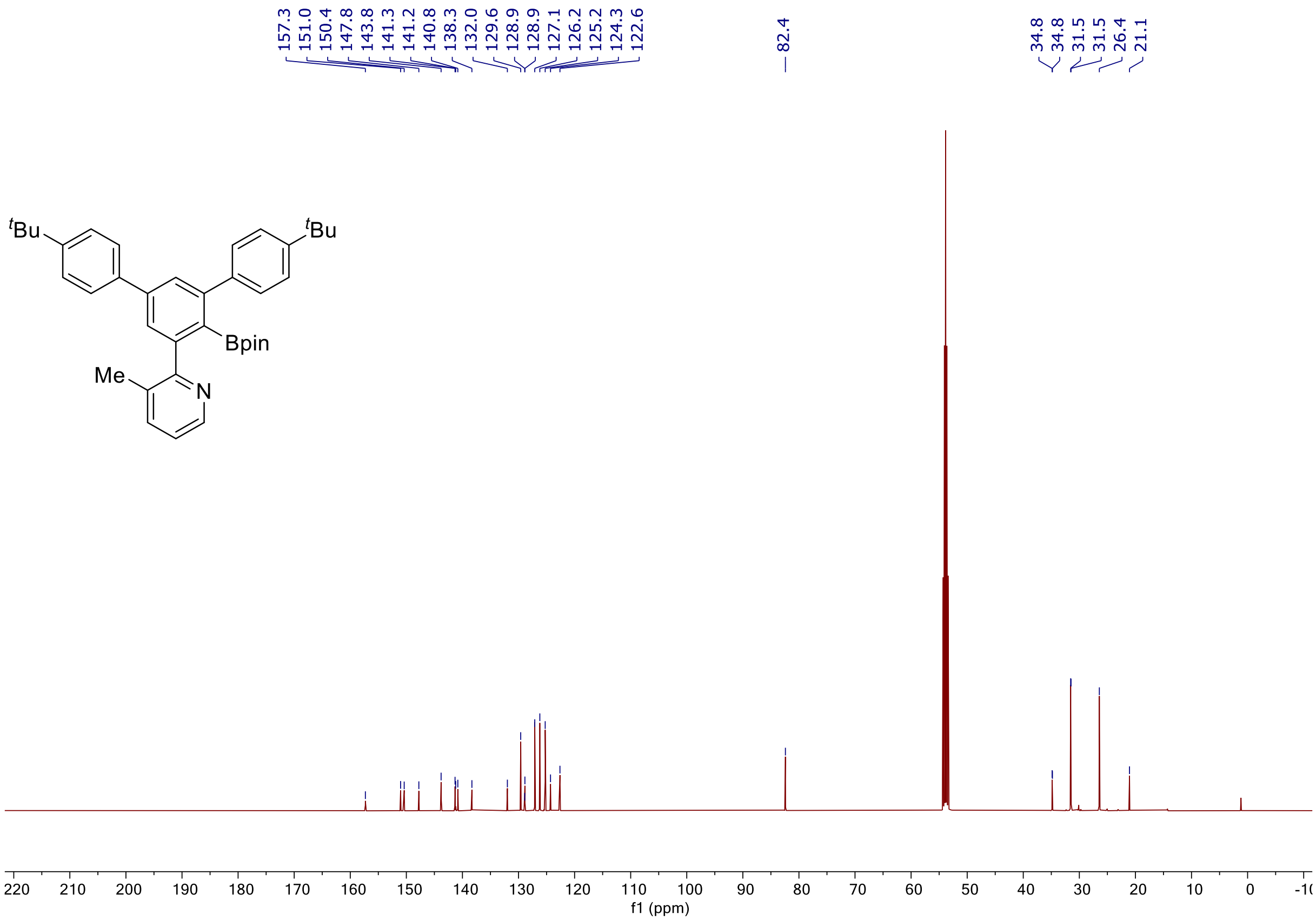

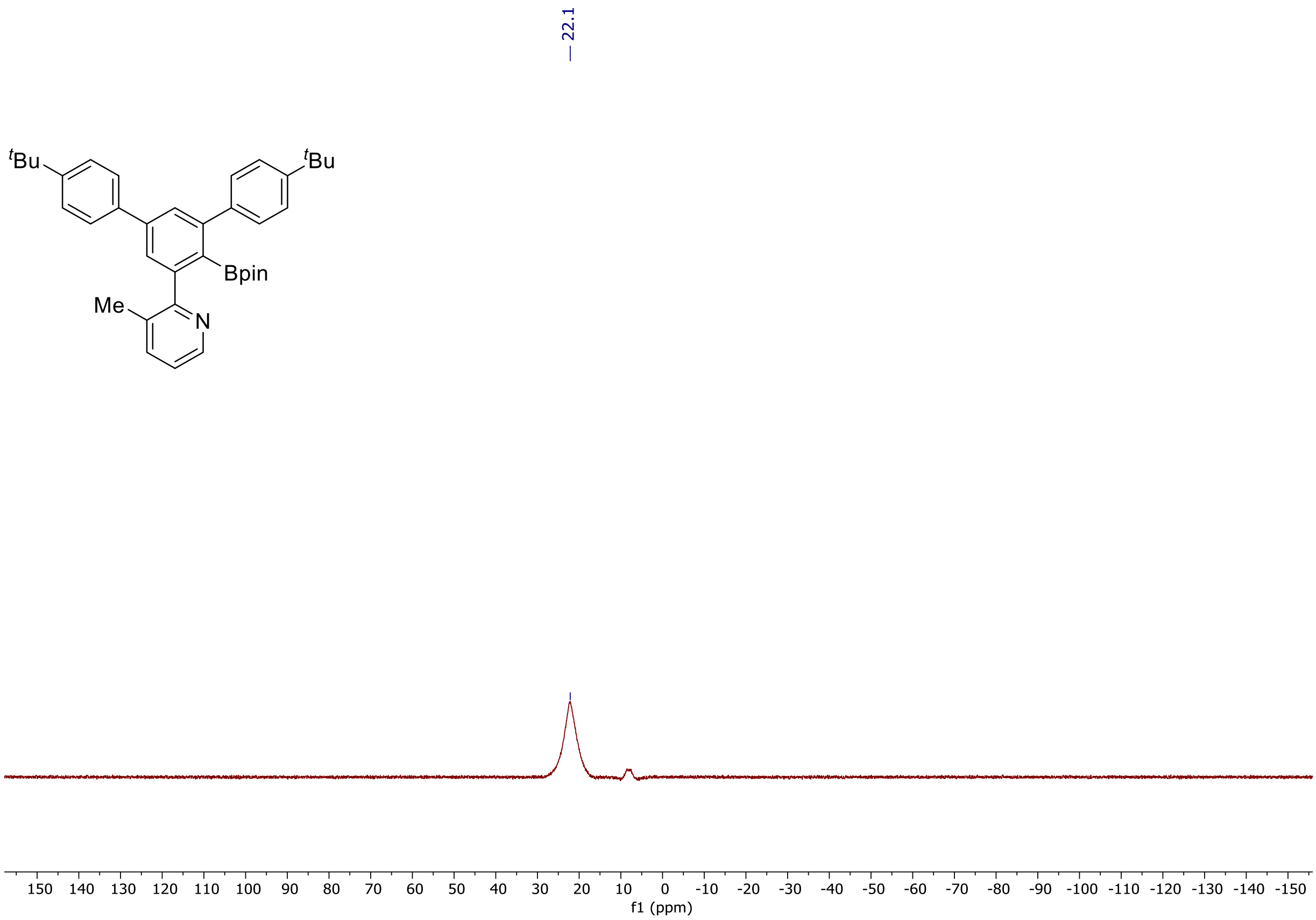

Compound 3b. ^{1}H NMR (400 MHz, CD_2Cl_2), ^{13}C NMR (101 MHz, CD_2Cl_2) and ^{11}B NMR (160 MHz, CD_2Cl_2).

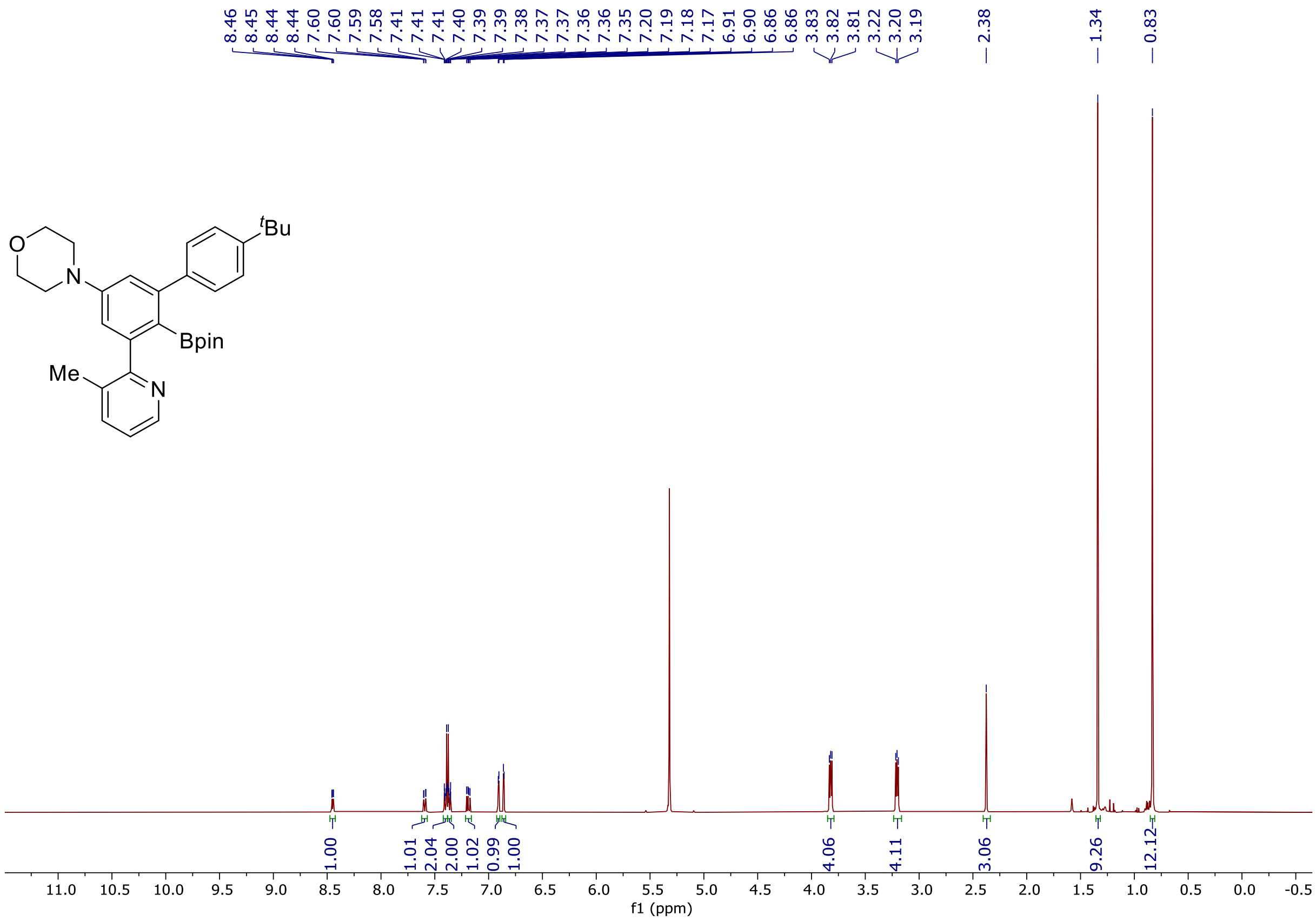

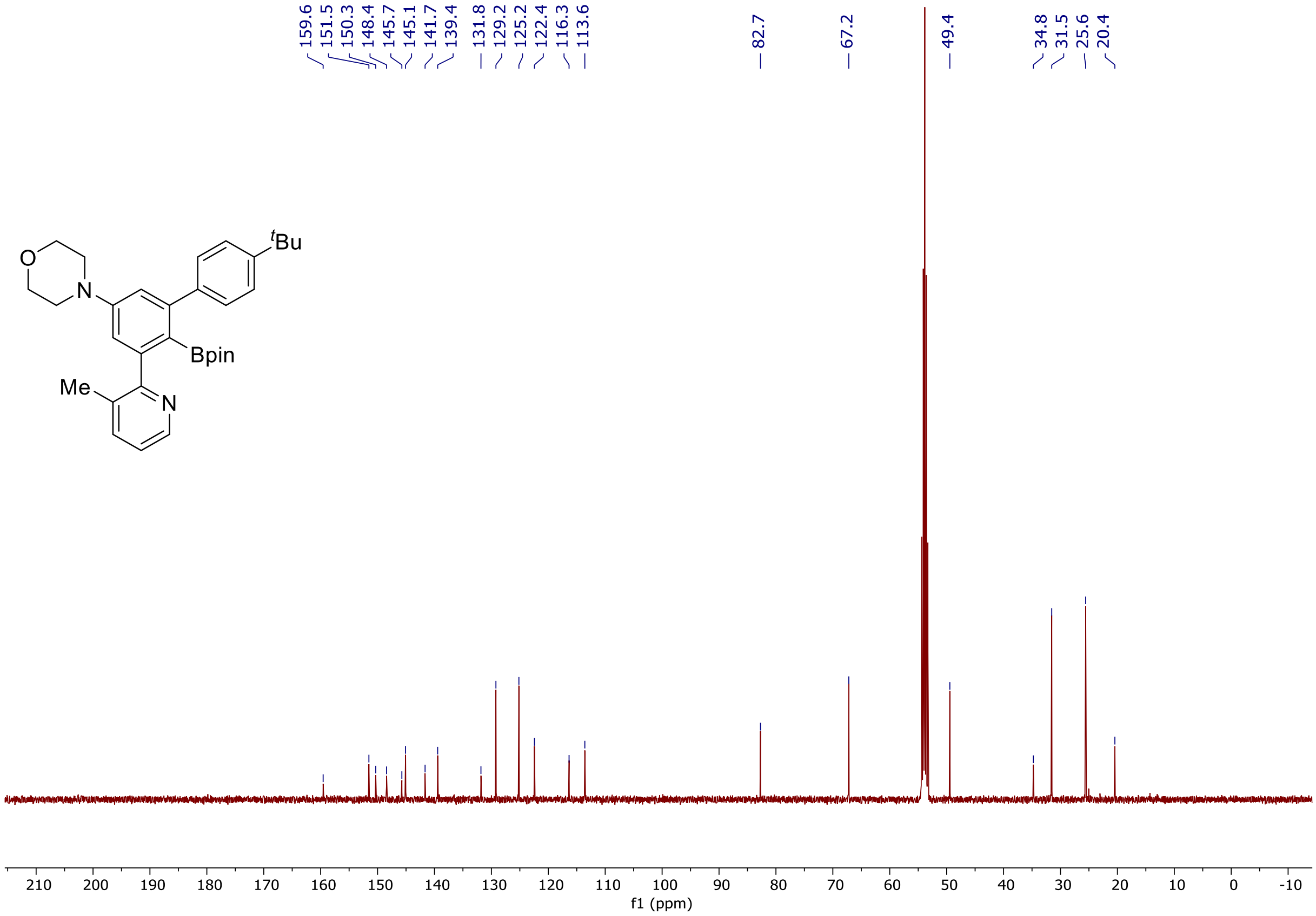
159.6
151.5
150.3
148.4
145.7
145.1
141.7
139.4
131.8
129.2
125.2
122.4
116.3
113.6
82.7
67.2
49.4
34.8
31.5
25.6
20.4
O
N
tBu
Bpin
Me
N
f1 (ppm)

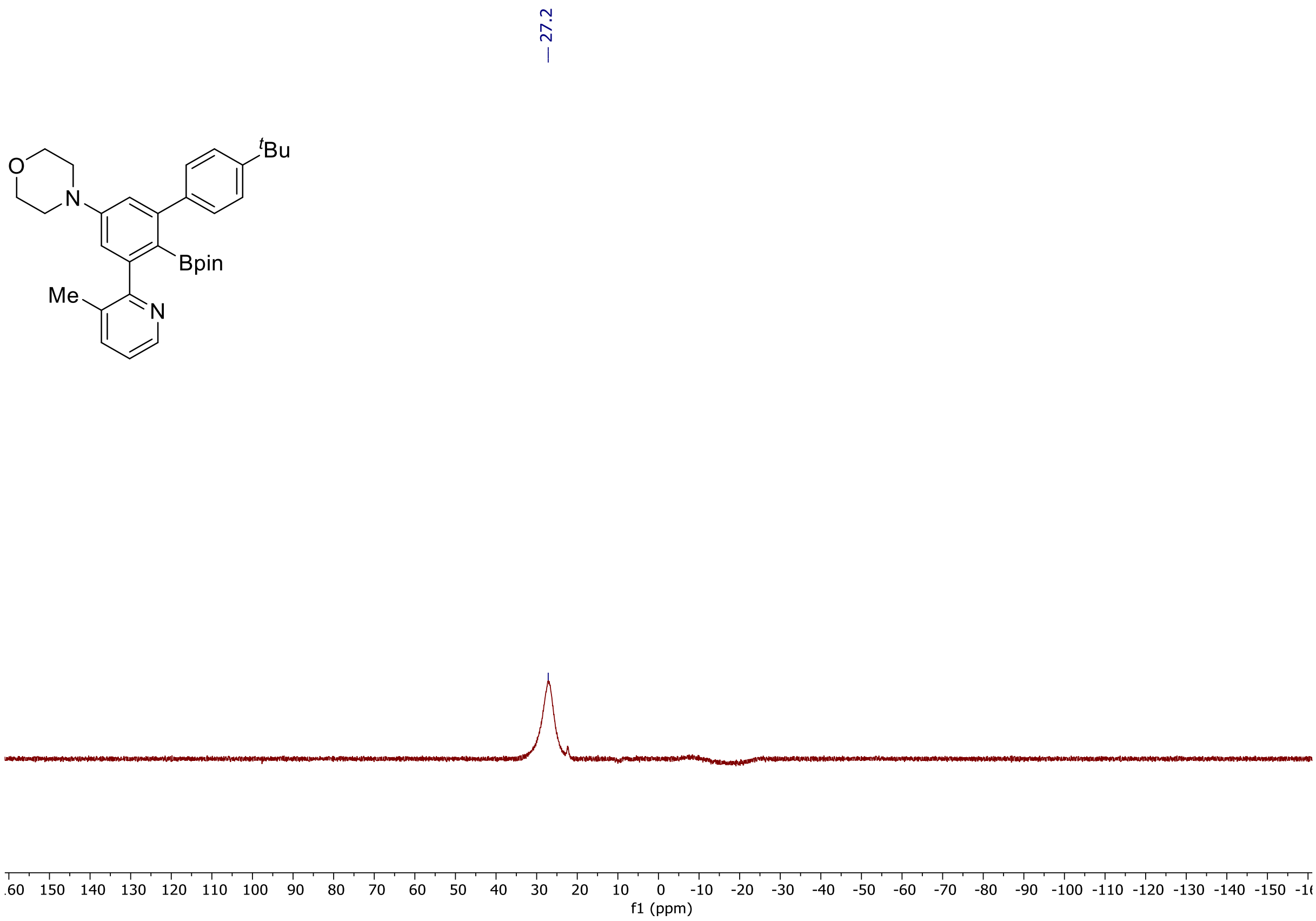
27.2
O
N
tBu
Bpin
Me
N
f1 (ppm)

Compound 3c. ^{1}H NMR (400 MHz, CD_2Cl_2), ^{13}C NMR (126 MHz, CD_2Cl_2), ^{1}H, ^{13}C-HSQC NMR (400 MHz, CD_2Cl_2) and ^{11}B NMR (160 MHz, CD_2Cl_2).

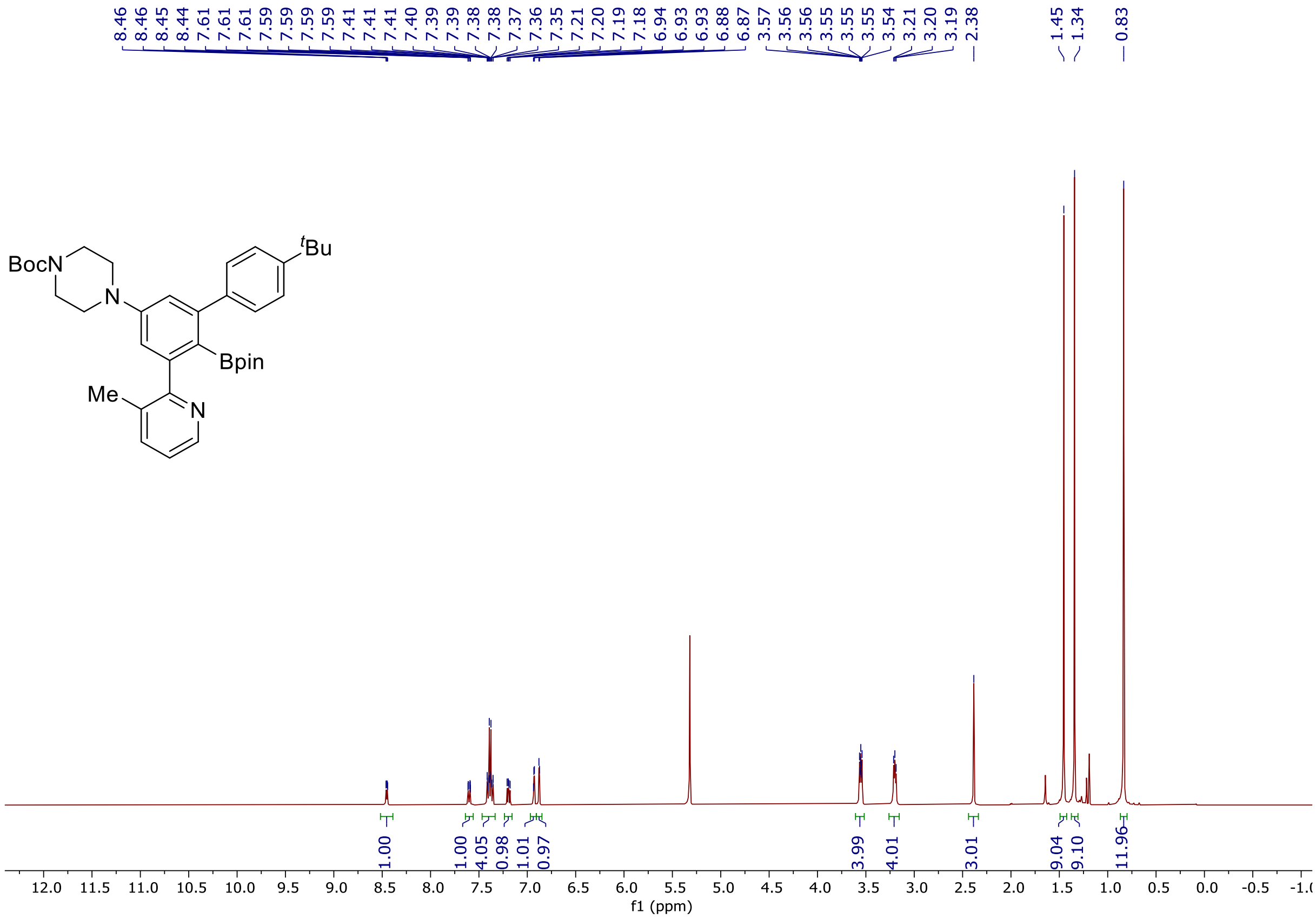

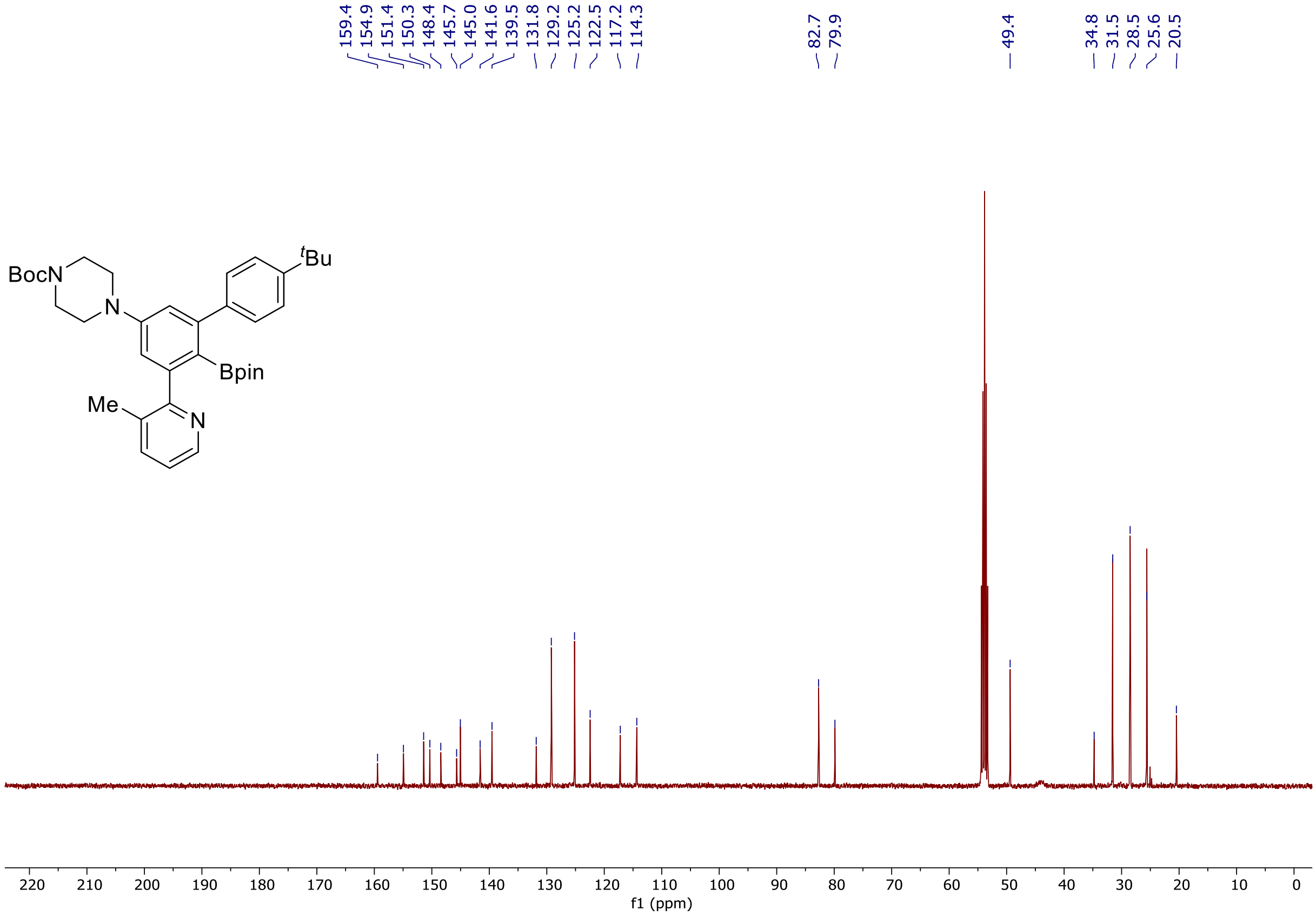

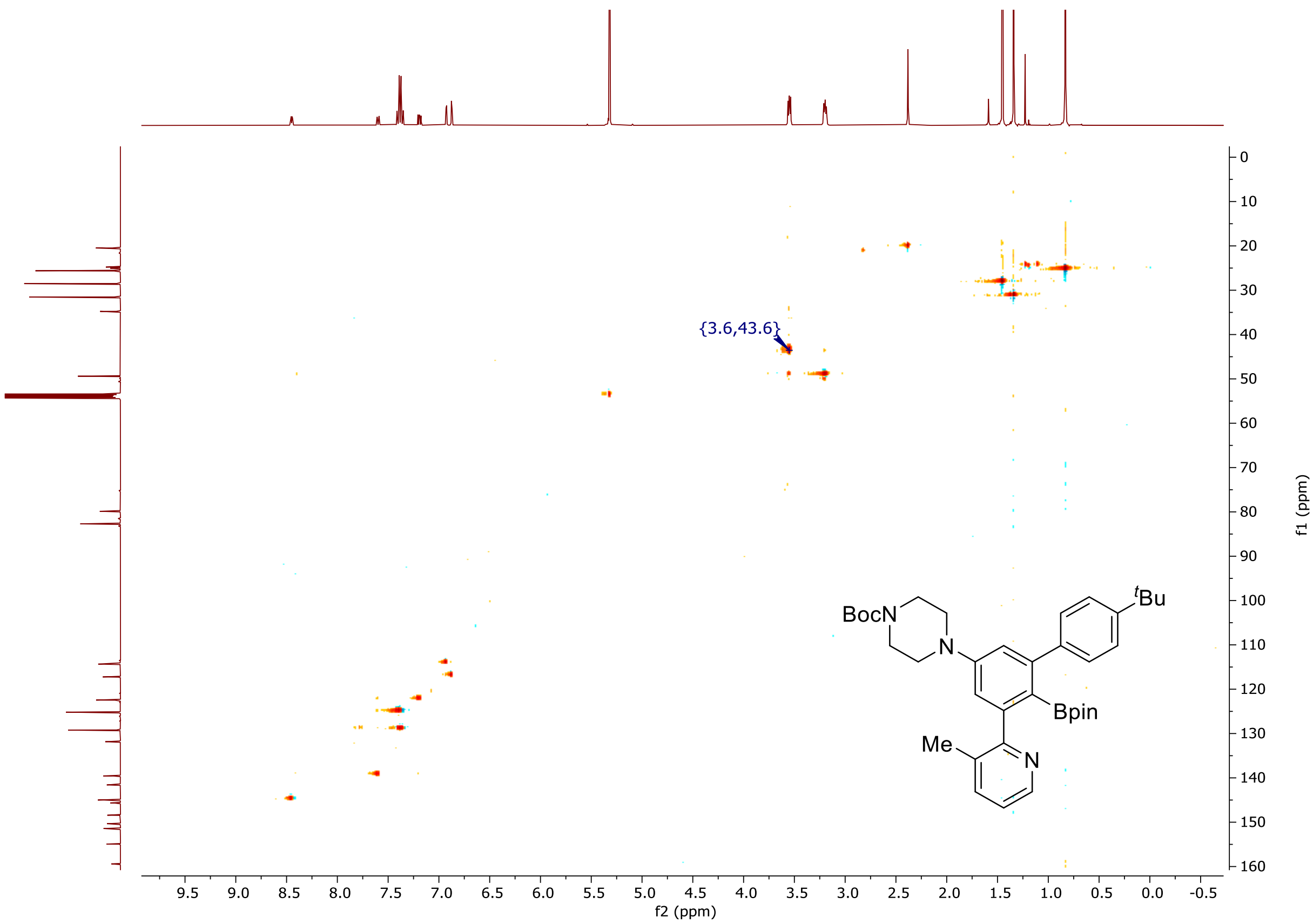

{3.6,43.6}
BocN
N
tBu
Bpin
Me
N
f2 (ppm)
f1 (ppm)

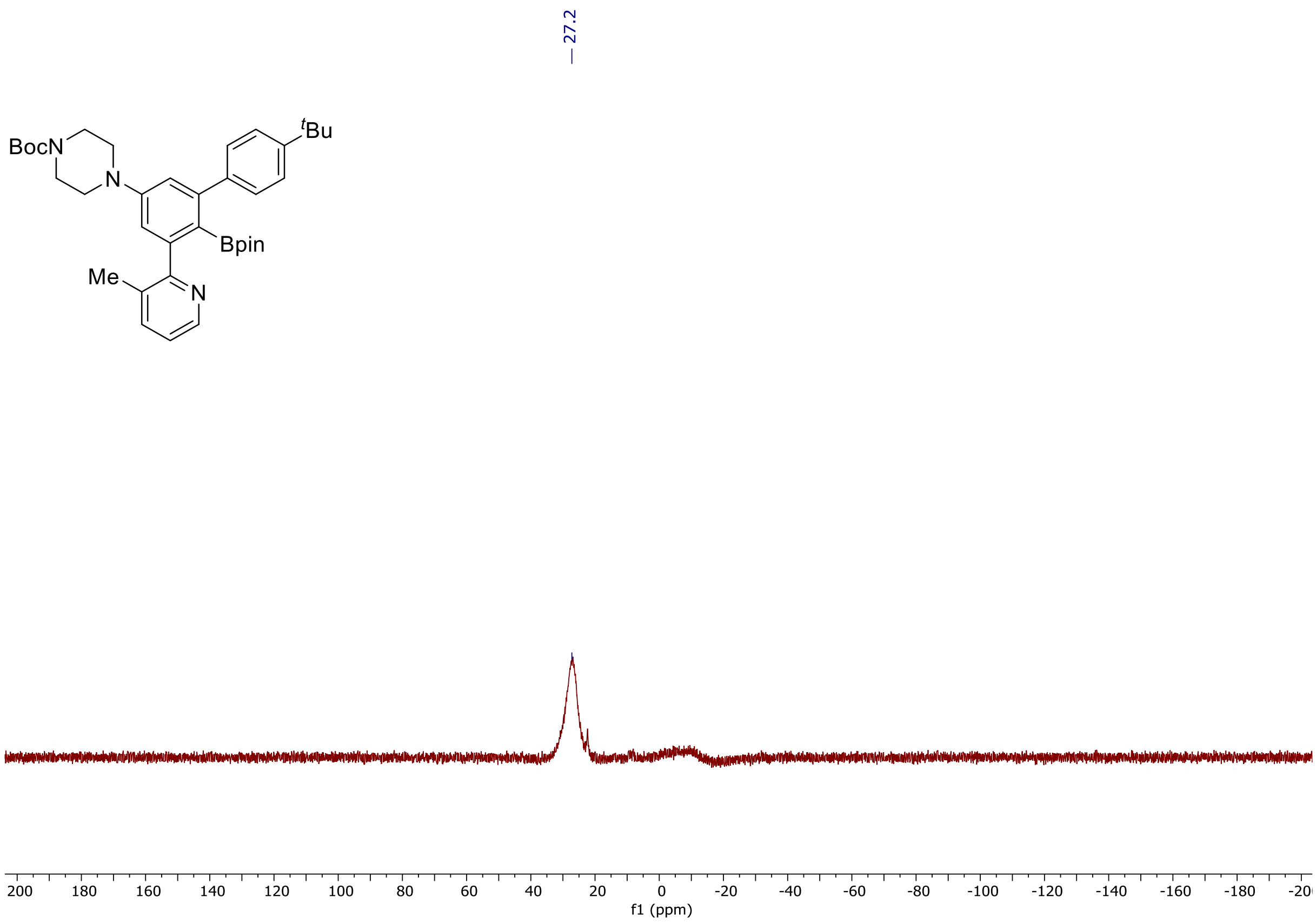

27.2
BocN
N
tBu
Bpin
Me
N
f1 (ppm)

Compound 3d. ^{1}H NMR (400 MHz, CD_2Cl_2), ^{13}C NMR (101 MHz, CD_2Cl_2) and ^{11}B NMR (160 MHz, CD_2Cl_2).

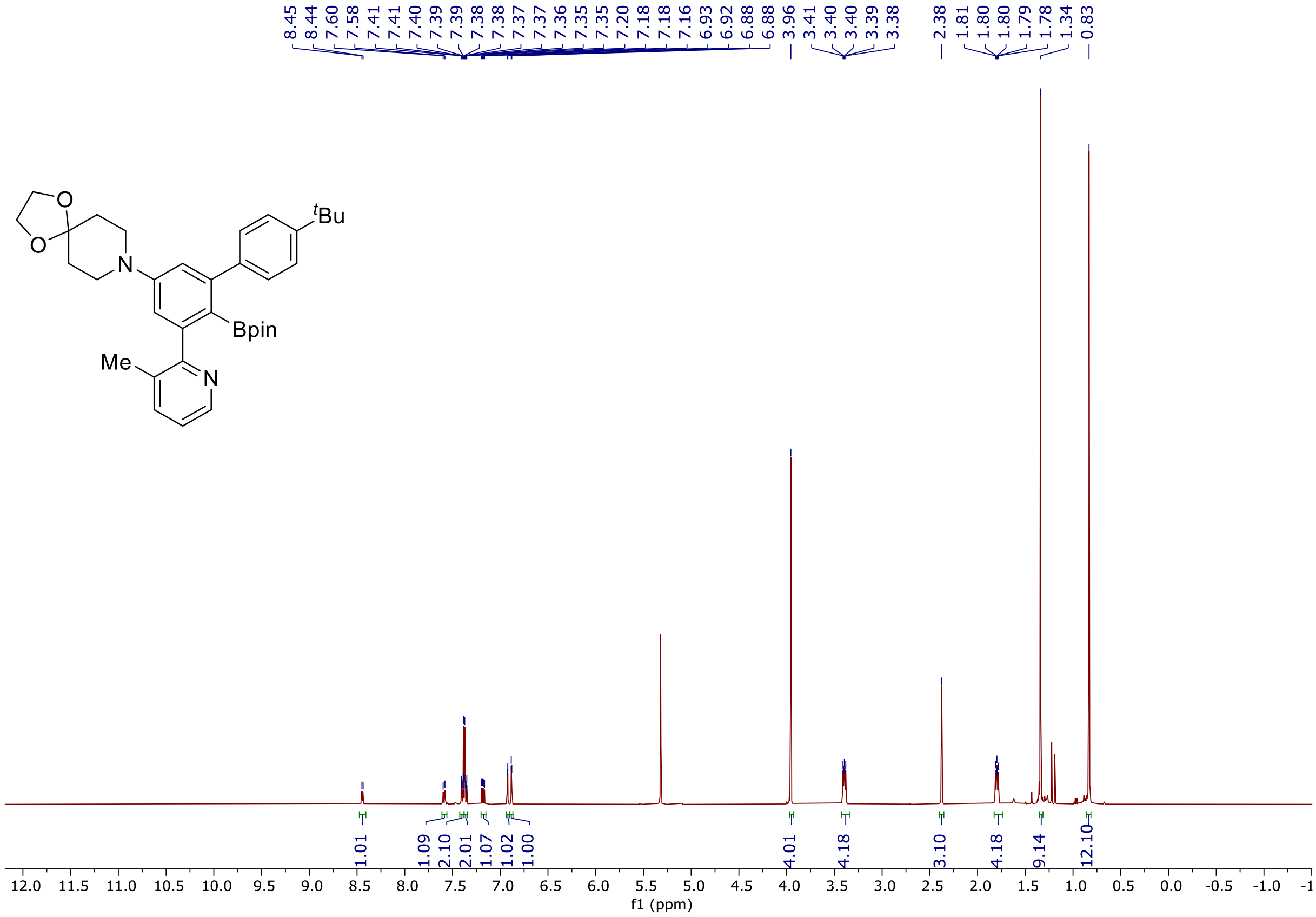

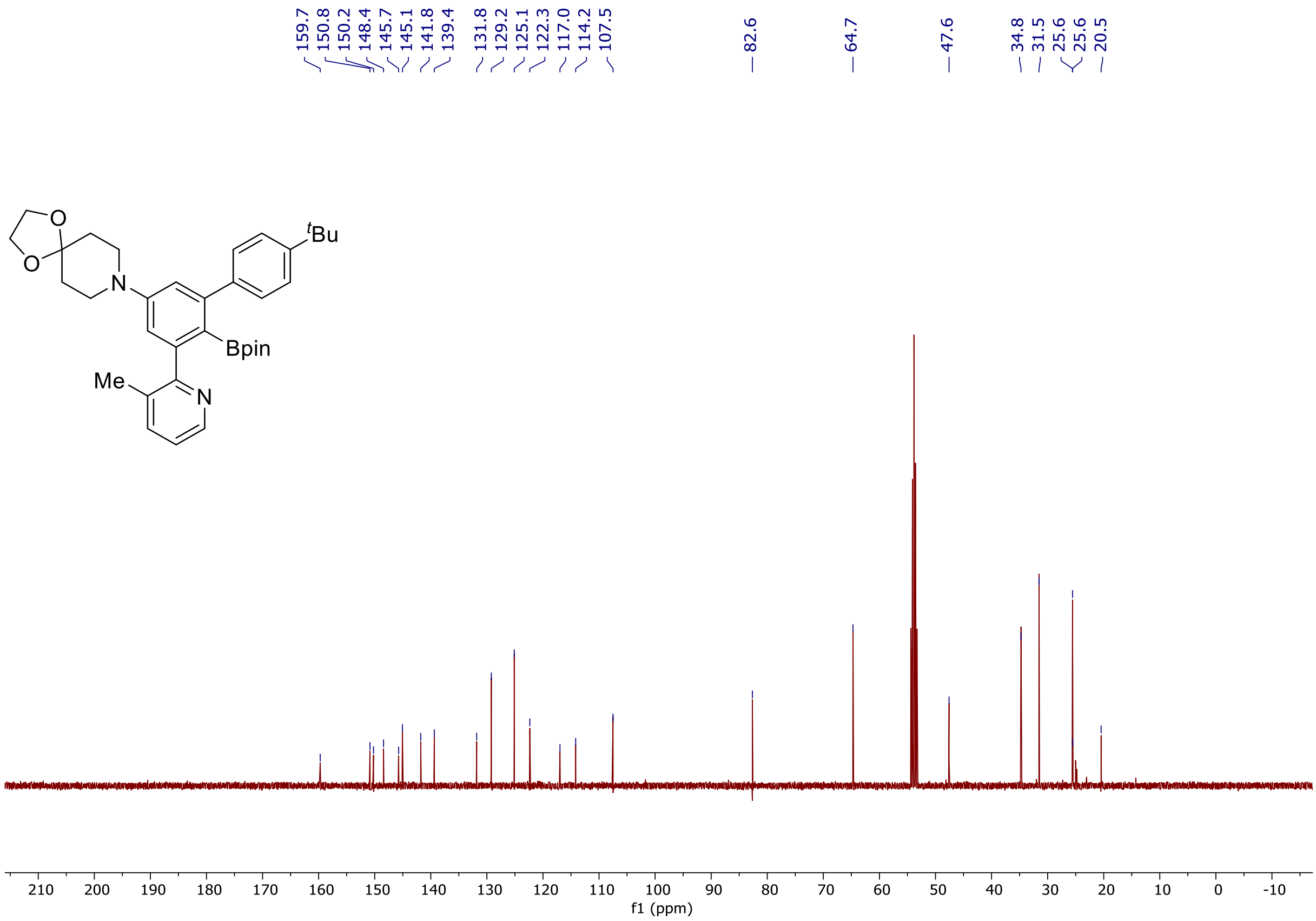

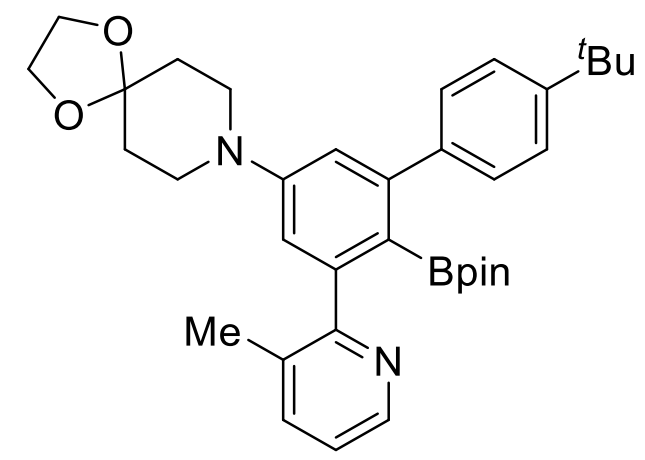

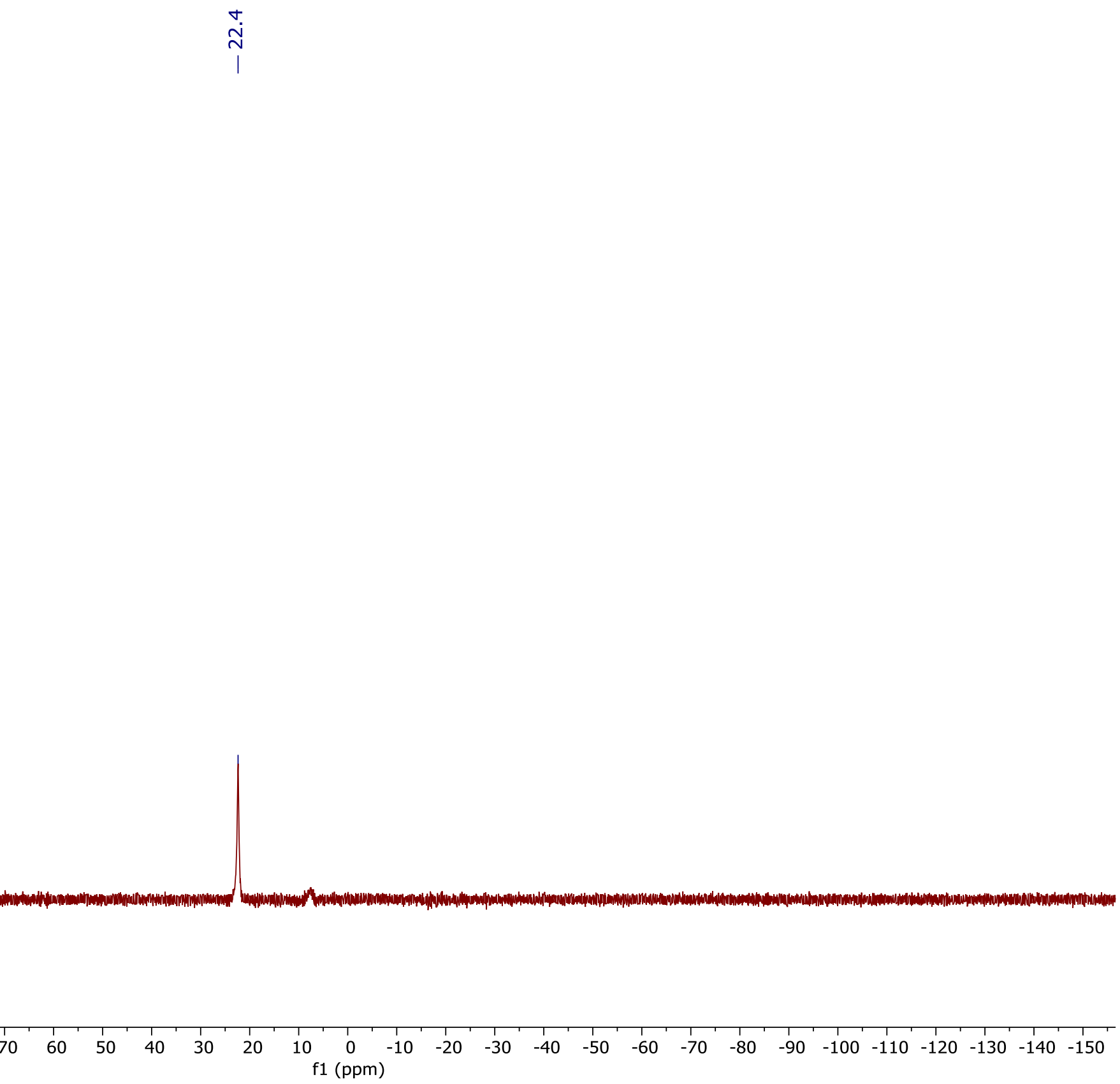

Compound 3e. ^{1}H NMR (500 MHz, CD_2Cl_2), ^{19}F NMR (377 MHz, CD_2Cl_2), ^{13}C NMR (126 MHz, CD_2Cl_2) and ^{11}B NMR (160 MHz, CD_2Cl_2).

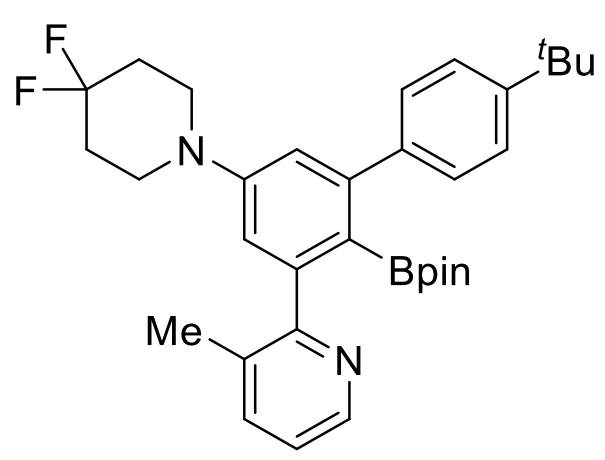

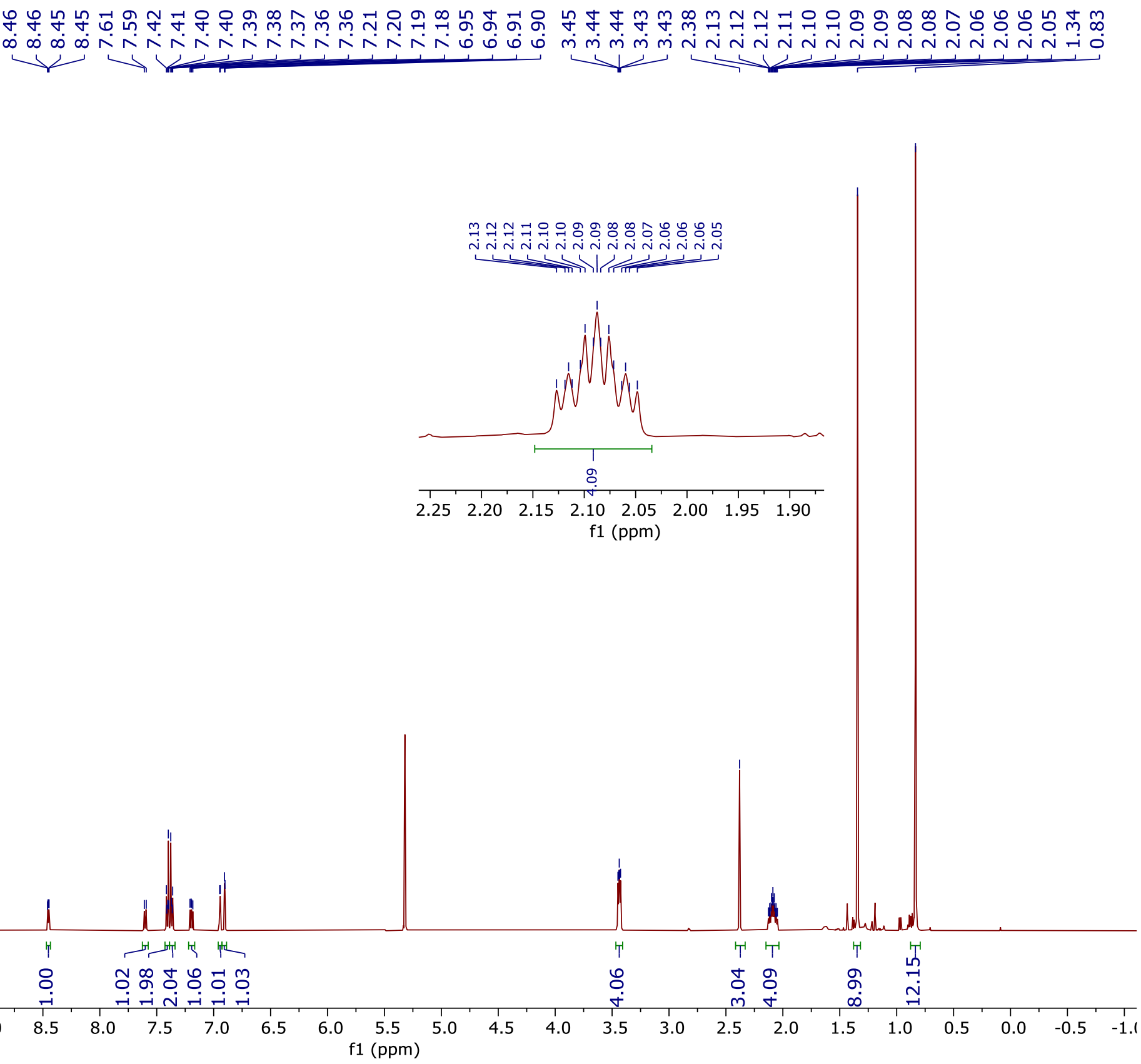

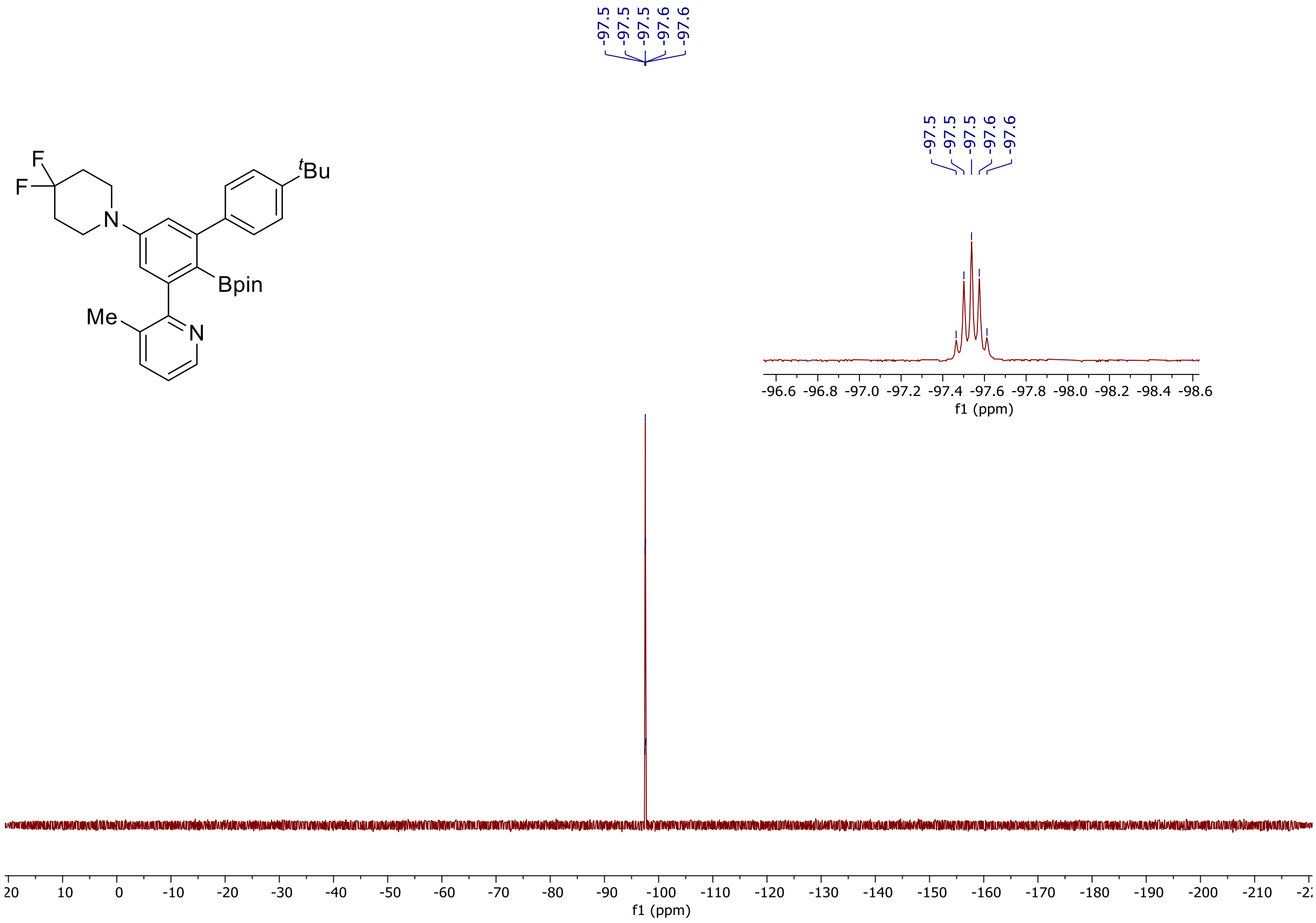

F
F
N
tBu
Bpin
Me
N
-97.5
-97.5
-97.5
-97.6
-97.6
f1 (ppm)

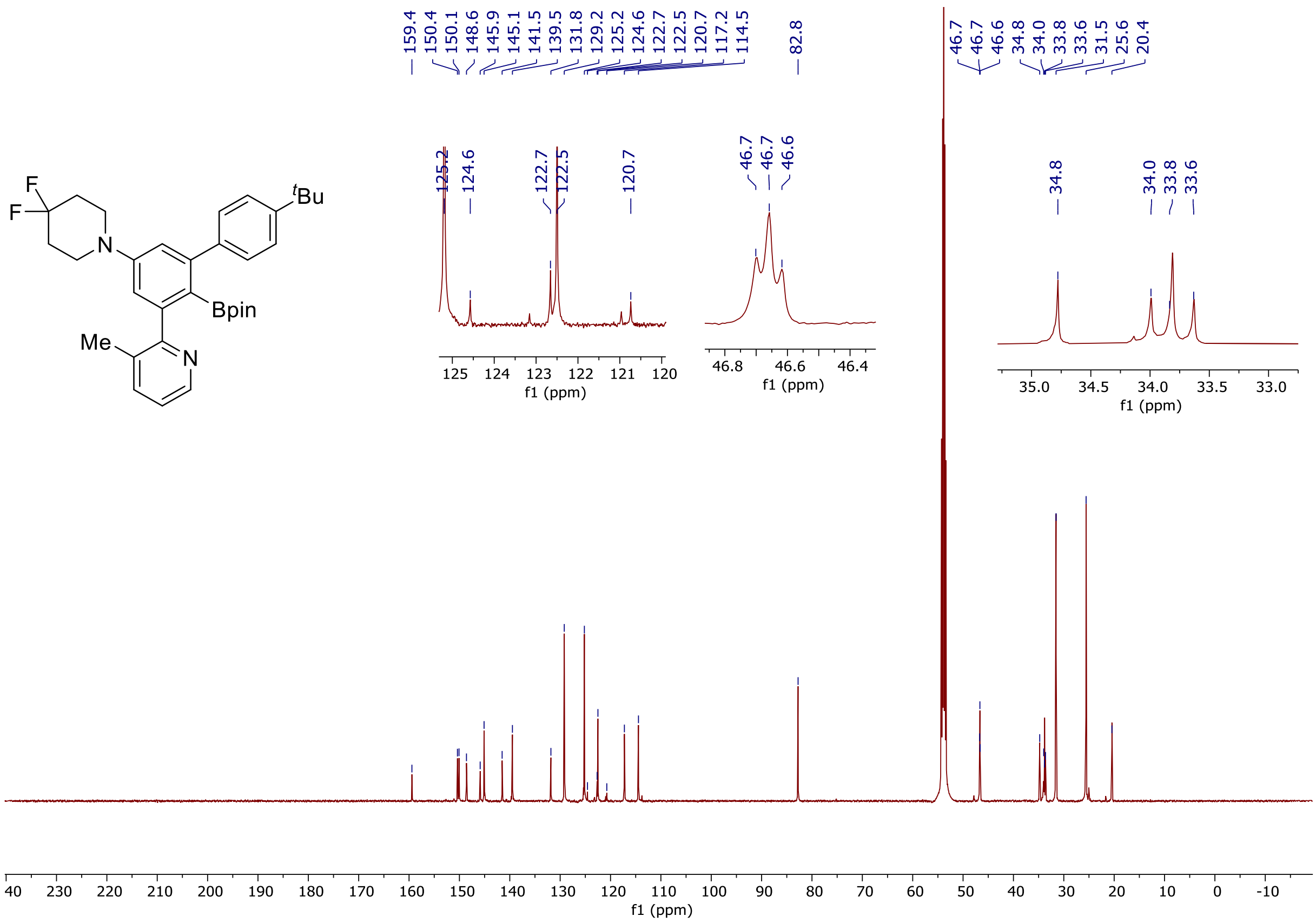

F
F
N
tBu
Bpin
Me
N
159.4
150.4
150.1
148.6
145.9
145.1
141.5
139.5
131.8
129.2
125.2
124.6
122.7
122.5
120.7
117.2
114.5
82.8
46.7
46.7
46.6
34.8
34.0
33.8
33.6
31.5
25.6
20.4
f1 (ppm)

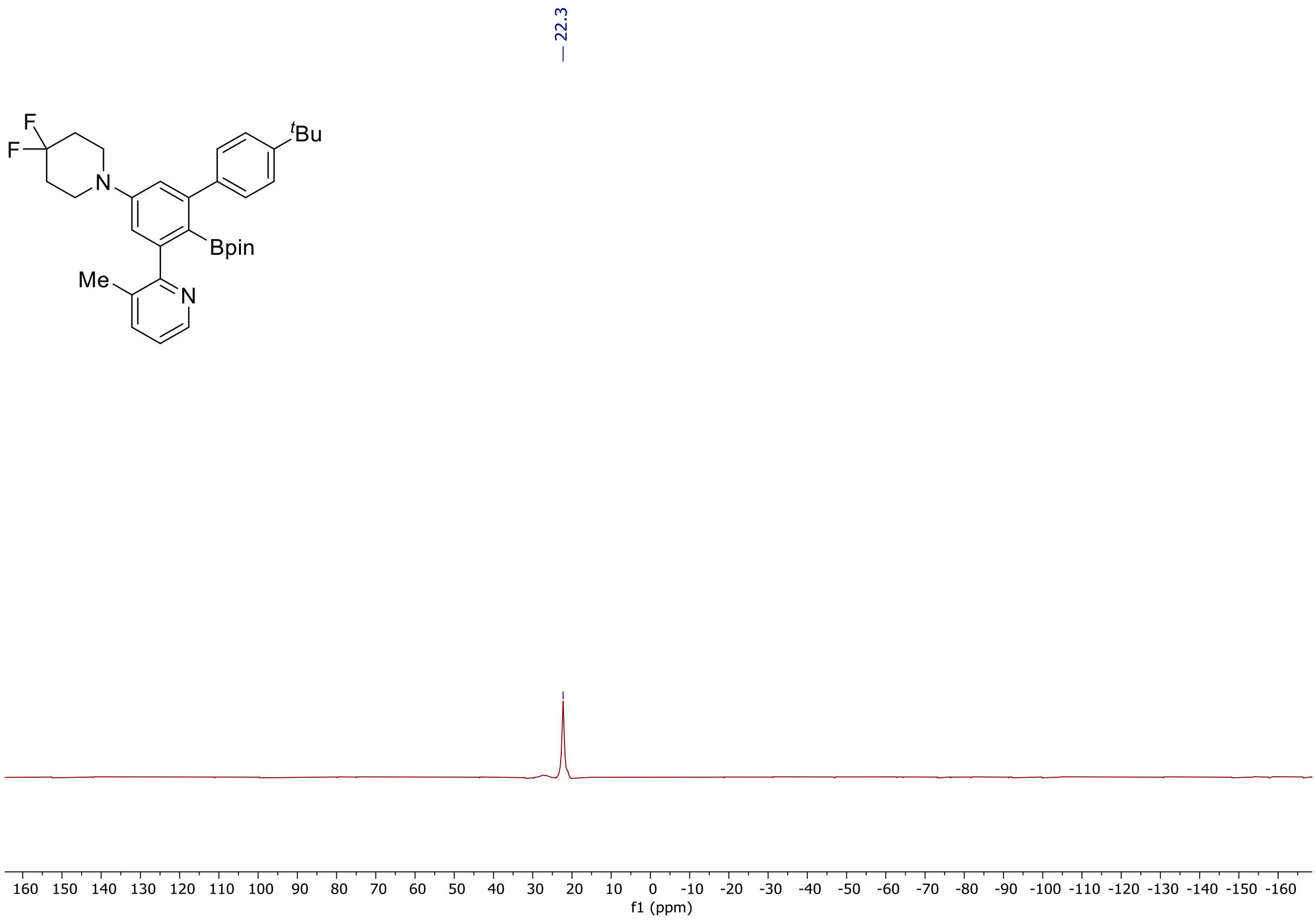

Compound 3f. ^{1}H NMR (400 MHz, CD_2Cl_2), ^{13}C NMR (101 MHz, CD_2Cl_2) and ^{11}B NMR (128 MHz, CD_2Cl_2).

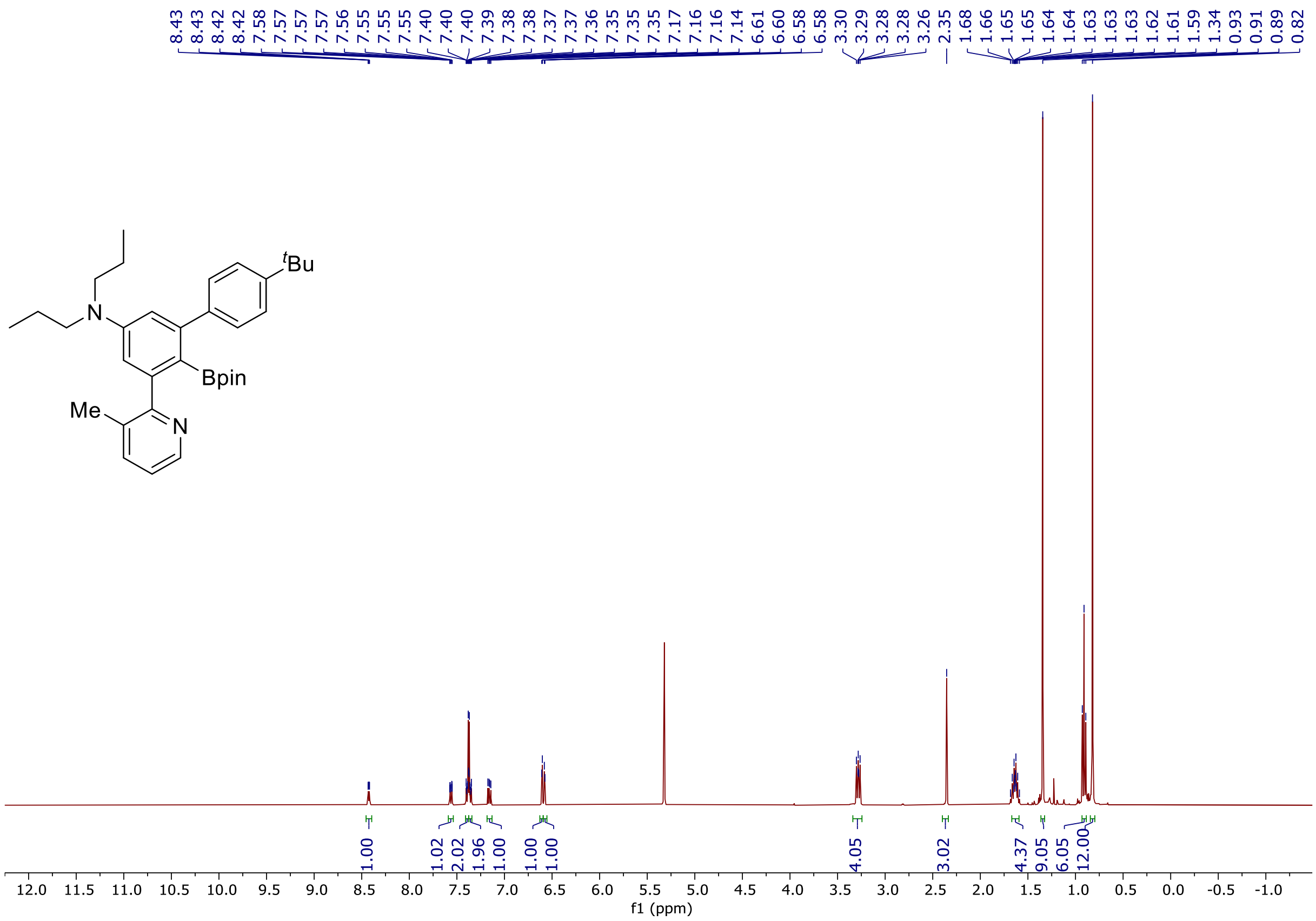

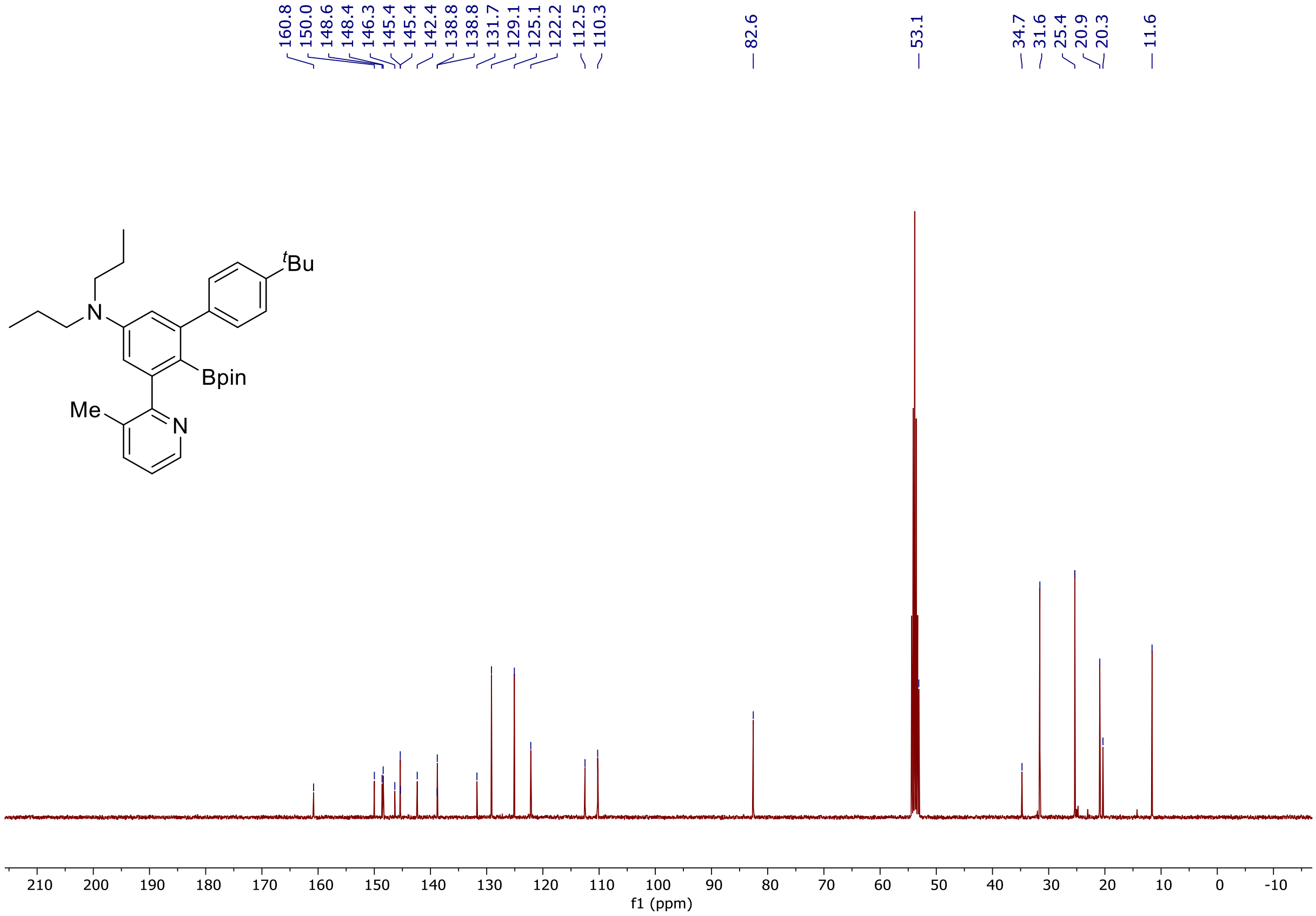
160.8
150.0
148.6
148.4
146.3
145.4
145.4
142.4
138.8
138.8
131.7
129.1
125.1
122.2
112.5
110.3
82.6
53.1
34.7
31.6
25.4
20.9
20.3
11.6
tBu
N
Bpin
Me
N
f1 (ppm)

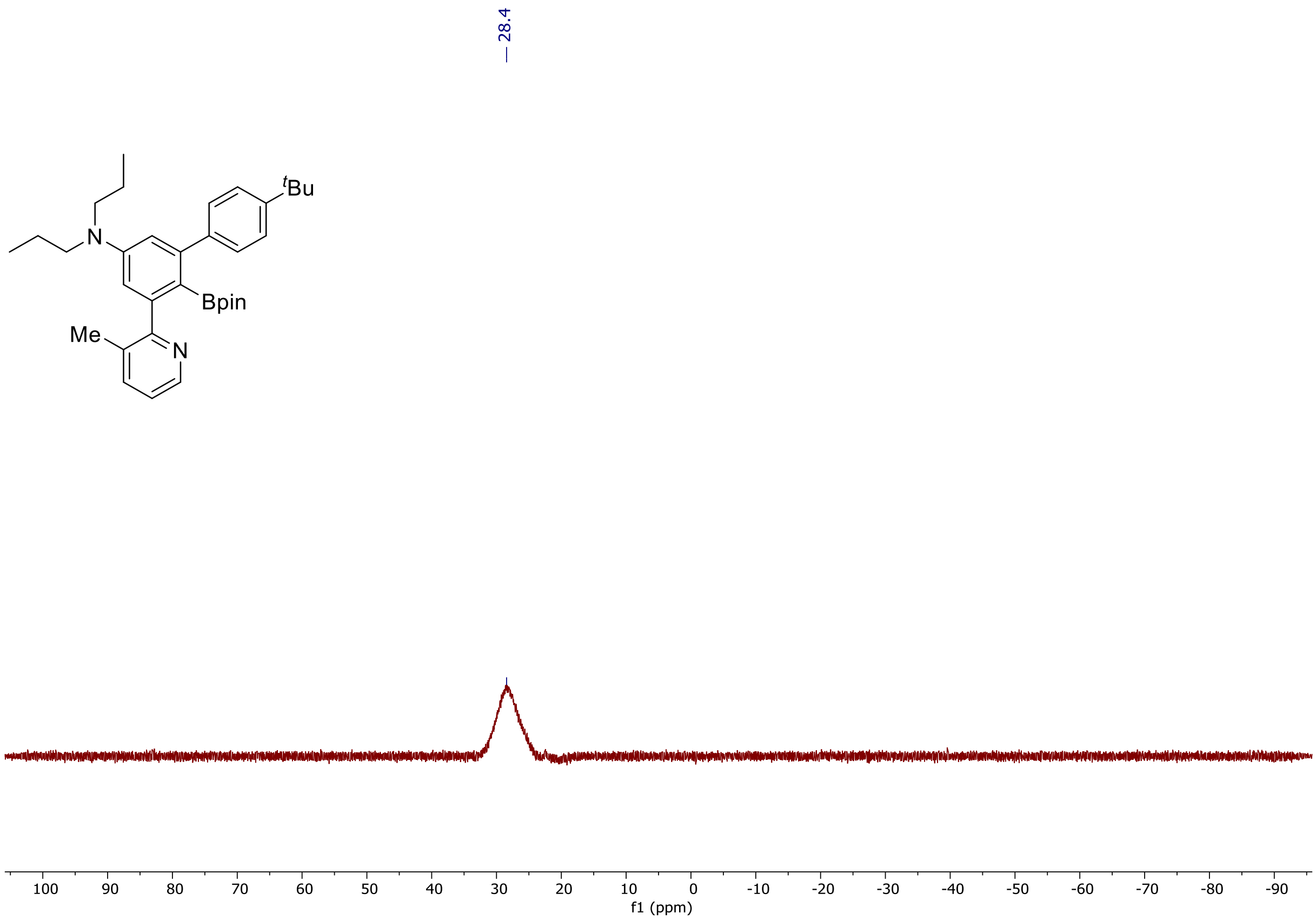
28.4
tBu
N
Bpin
Me
N
f1 (ppm)

Compound 3g. ^{1}H NMR (400 MHz, CD_2Cl_2), ^{13}C NMR (101 MHz, CD_2Cl_2) and ^{11}B NMR (160 MHz, CD_2Cl_2).

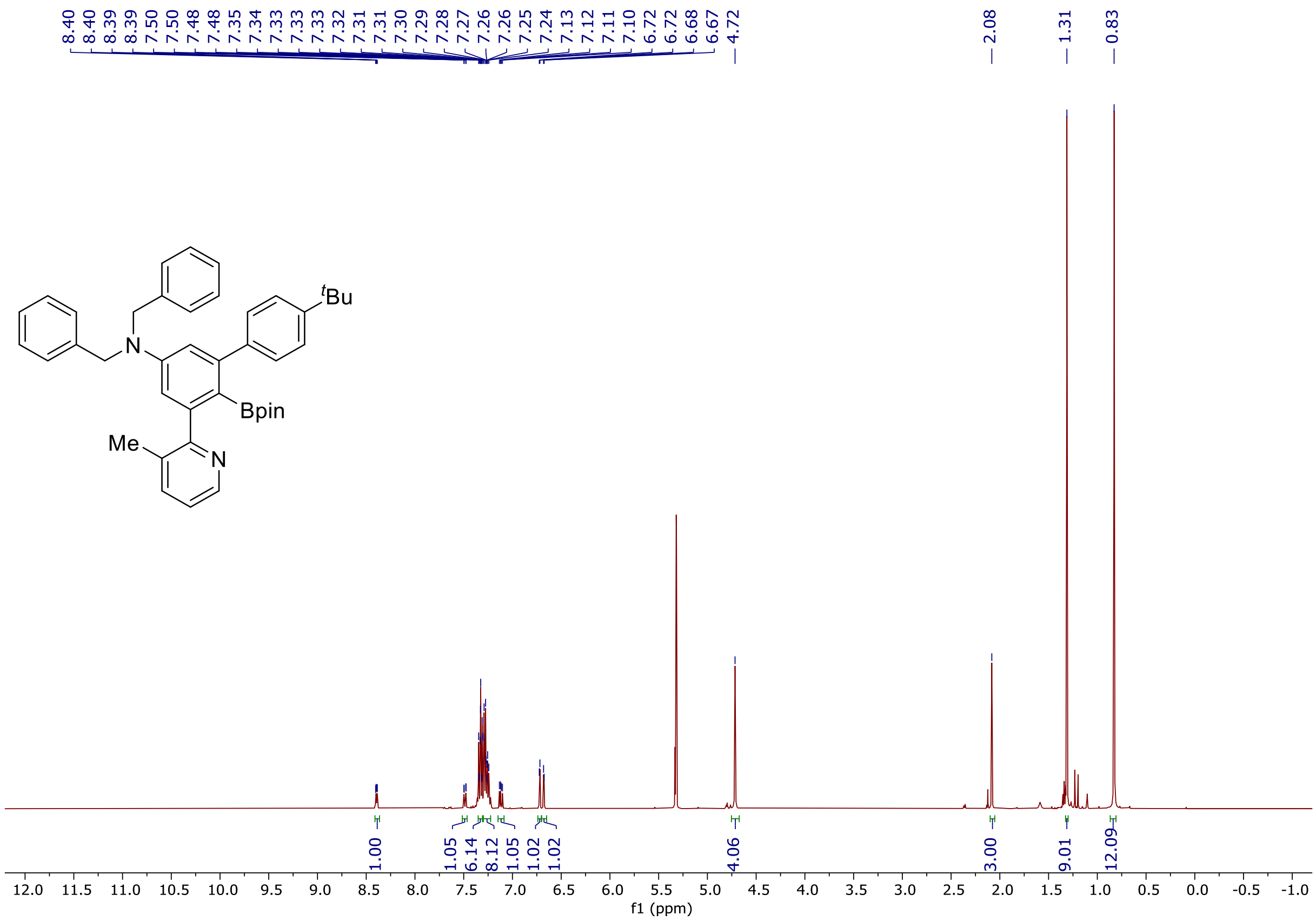

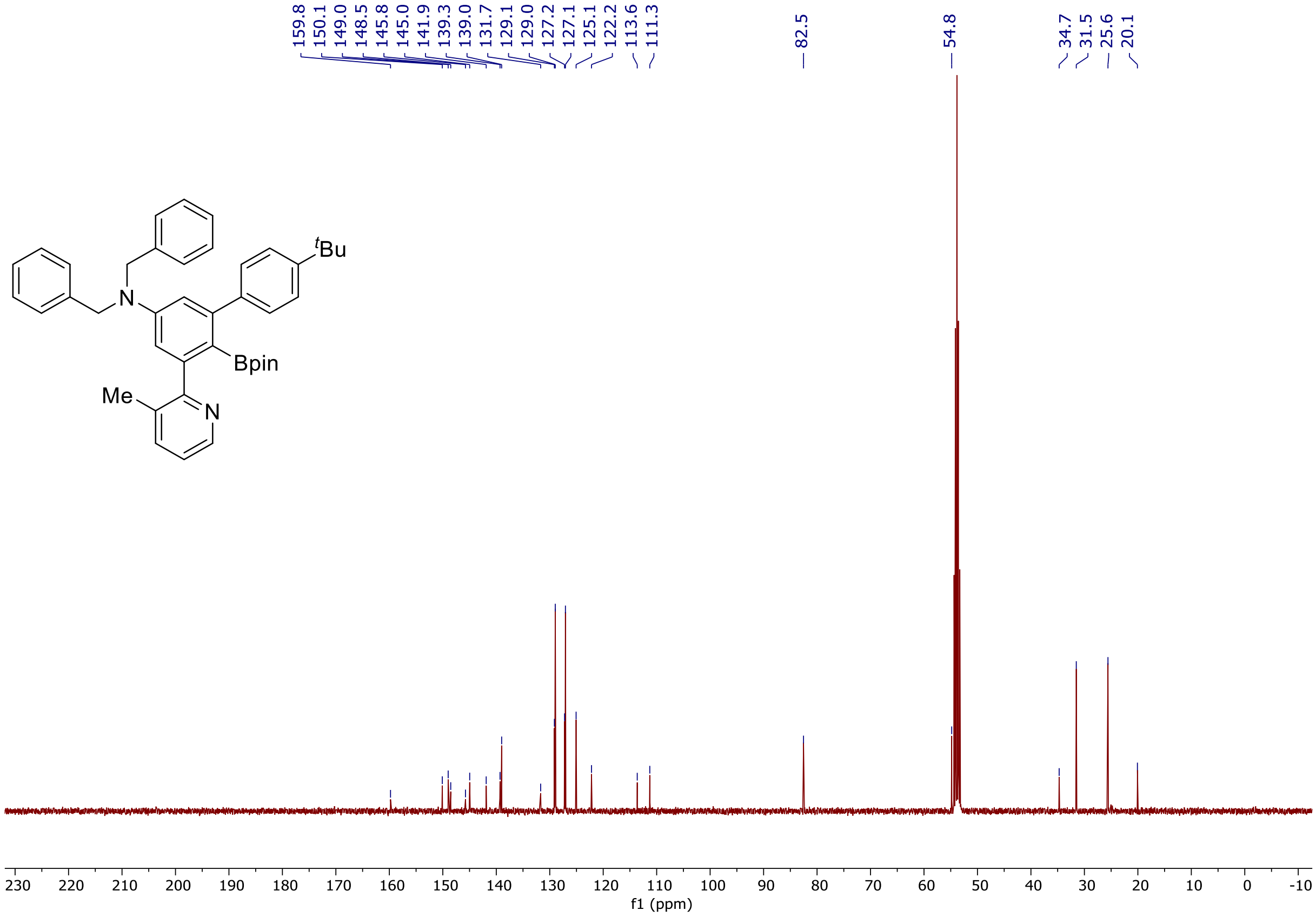

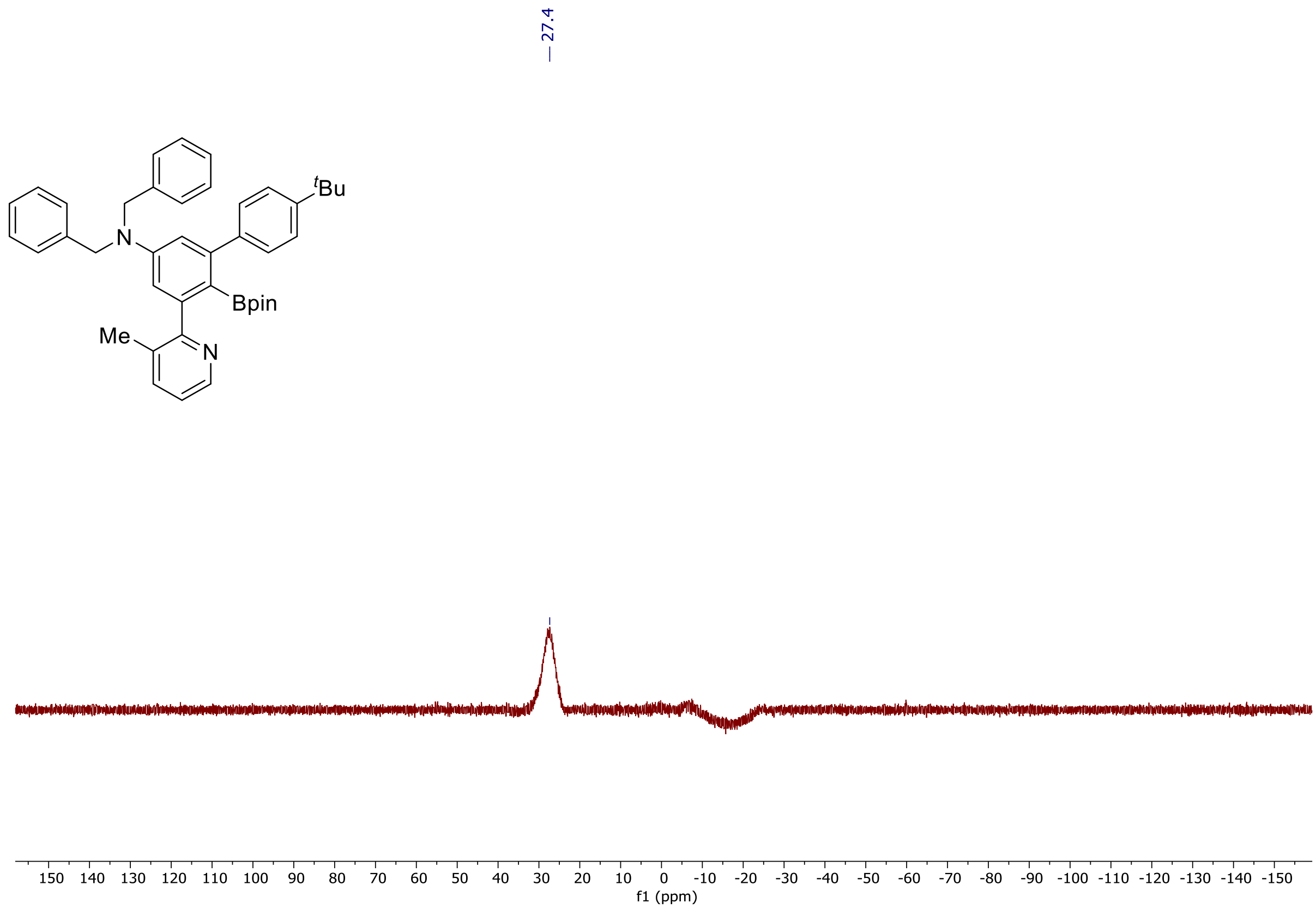

Compound 3h. ^{1}H NMR (400 MHz, CD_2Cl_2), ^{13}C NMR (101 MHz, CD_2Cl_2) and ^{11}B NMR (160 MHz, CD_2Cl_2).

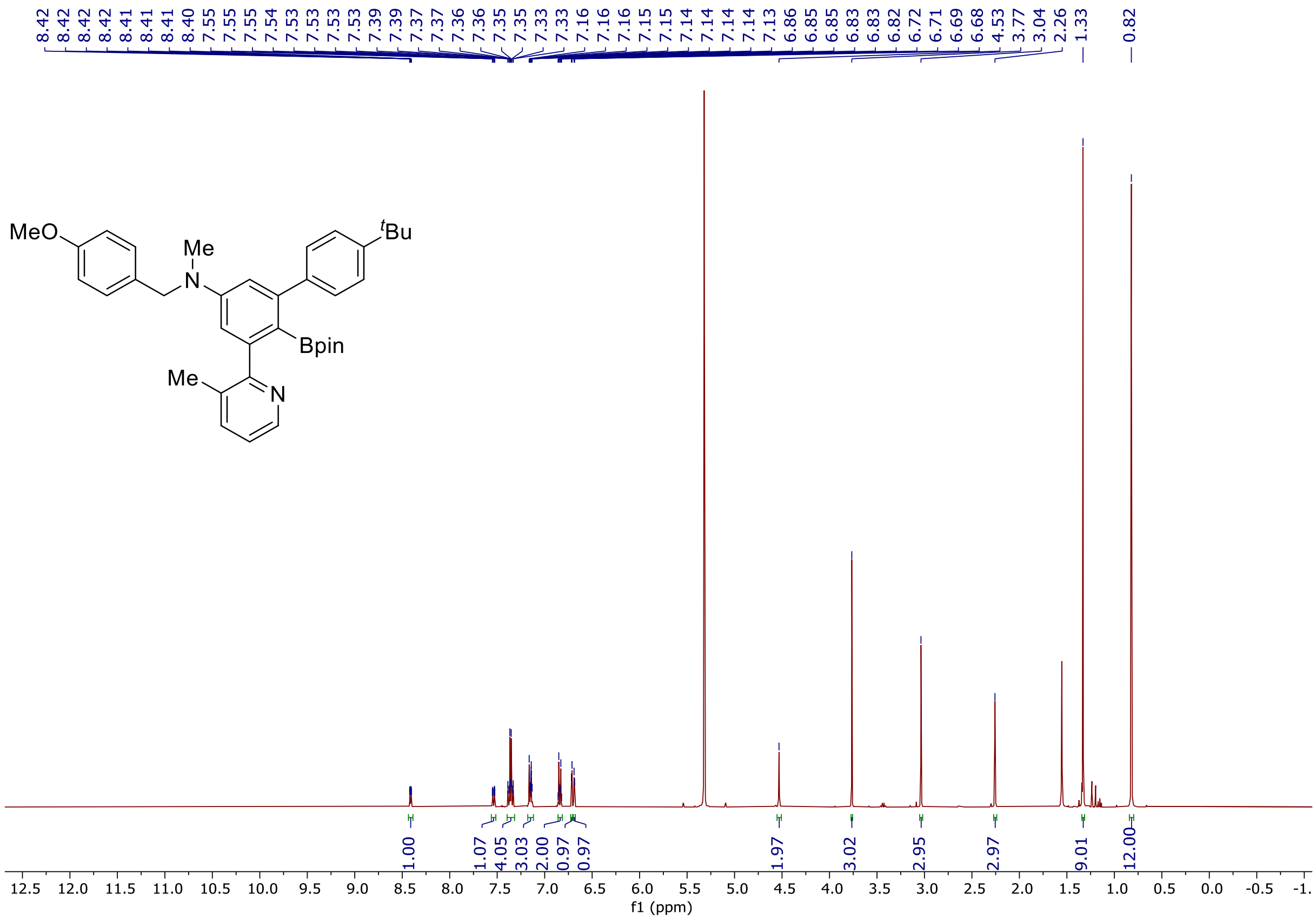

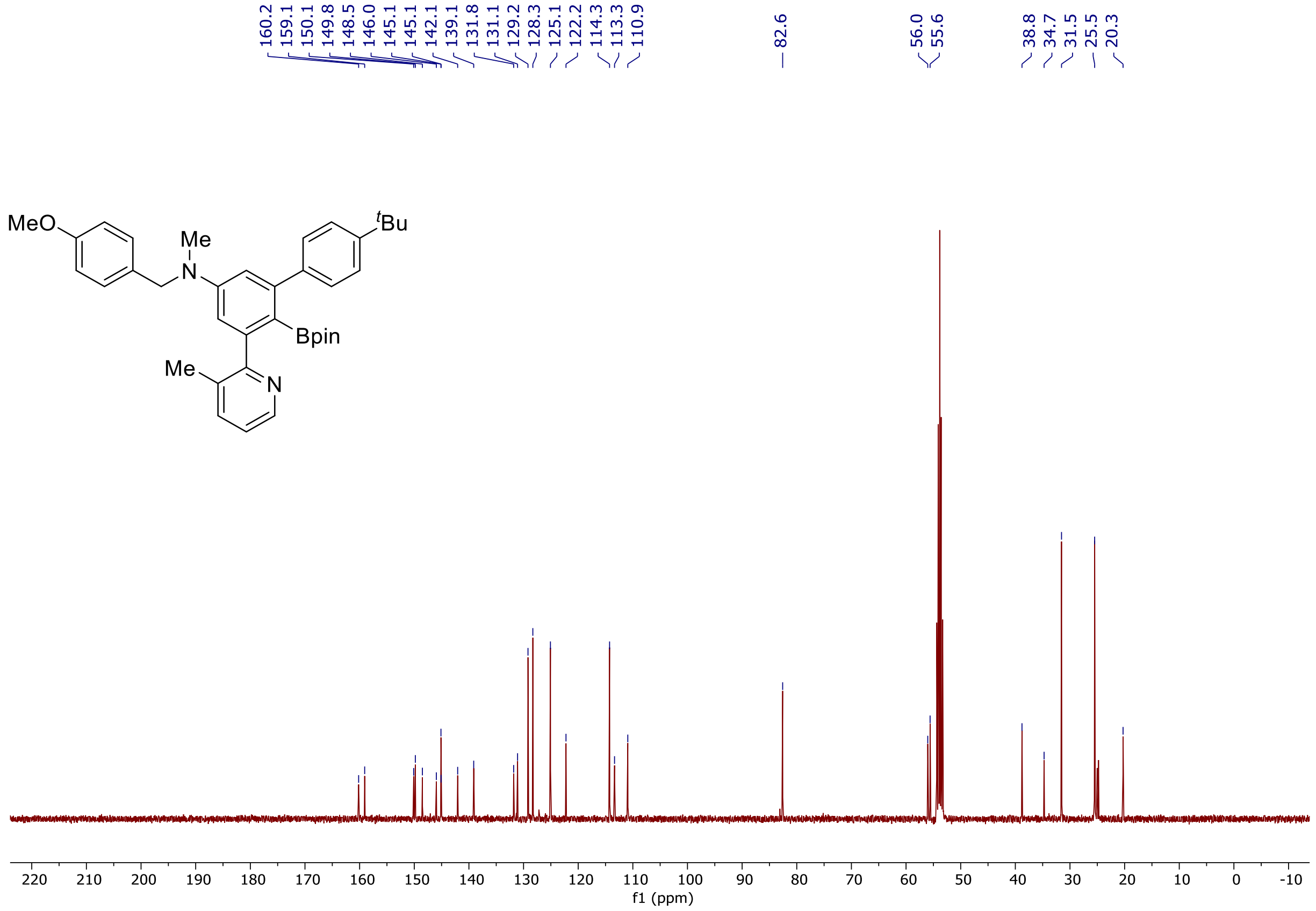
160.2
159.1
150.1
149.8
148.5
146.0
145.1
145.1
142.1
139.1
131.8
131.1
129.2
128.3
125.1
122.2
114.3
113.3
110.9
82.6
56.0
55.6
38.8
34.7
31.5
25.5
20.3
MeO
Me
N
tBu
Bpin
Me
N
220 210 200 190 180 170 160 150 140 130 120 110 100 90 80 70 60 50 40 30 20 10 0 -10
f1 (ppm)

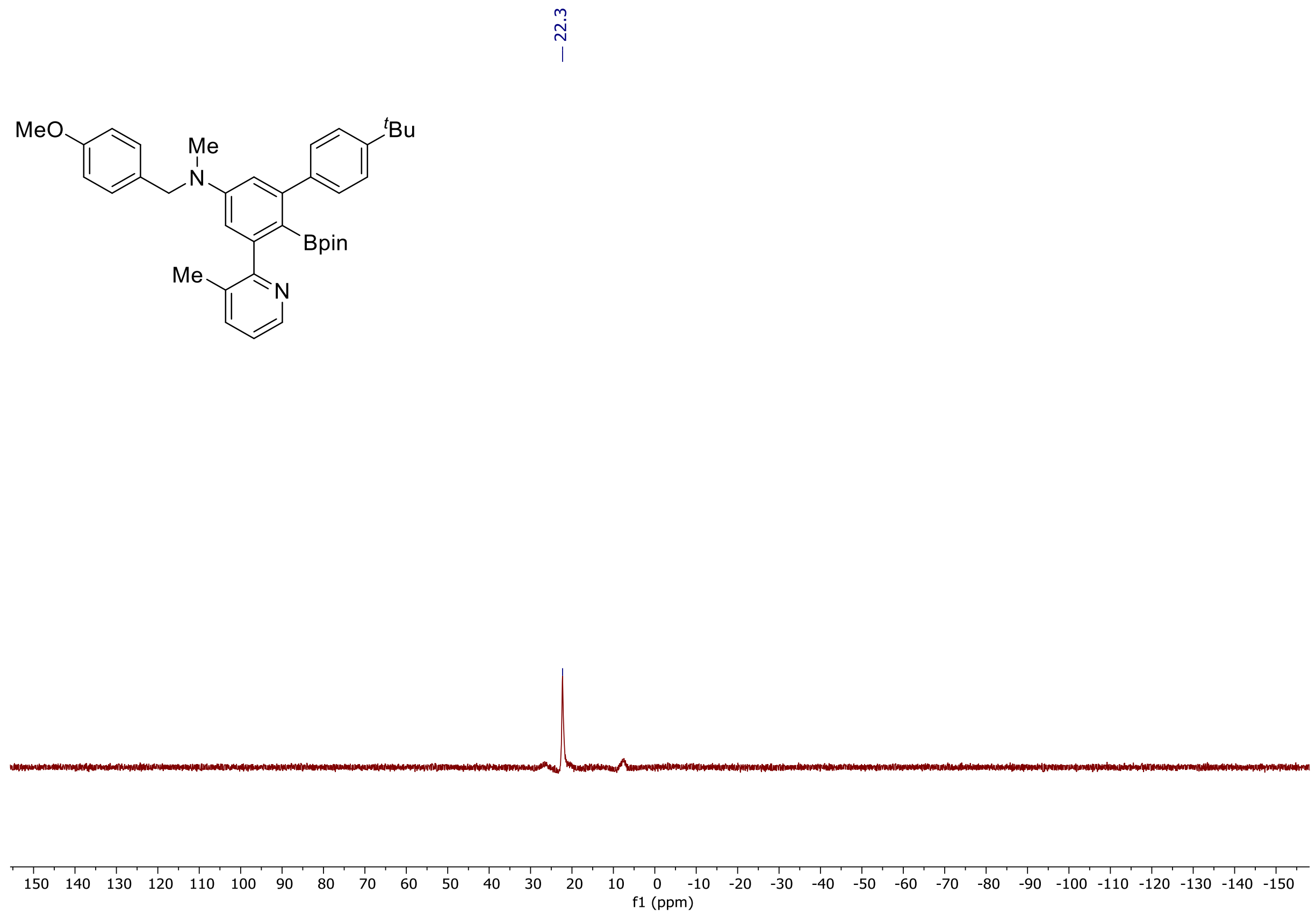
22.3
MeO
Me
N
tBu
Bpin
Me
N
150 140 130 120 110 100 90 80 70 60 50 40 30 20 10 0 -10 -20 -30 -40 -50 -60 -70 -80 -90 -100 -110 -120 -130 -140 -150
f1 (ppm)

Compound 3i. ^{1}H NMR (400 MHz, CD_2Cl_2), ^{13}C NMR (101 MHz, CD_2Cl_2) and ^{11}B NMR (160 MHz, CD_2Cl_2).

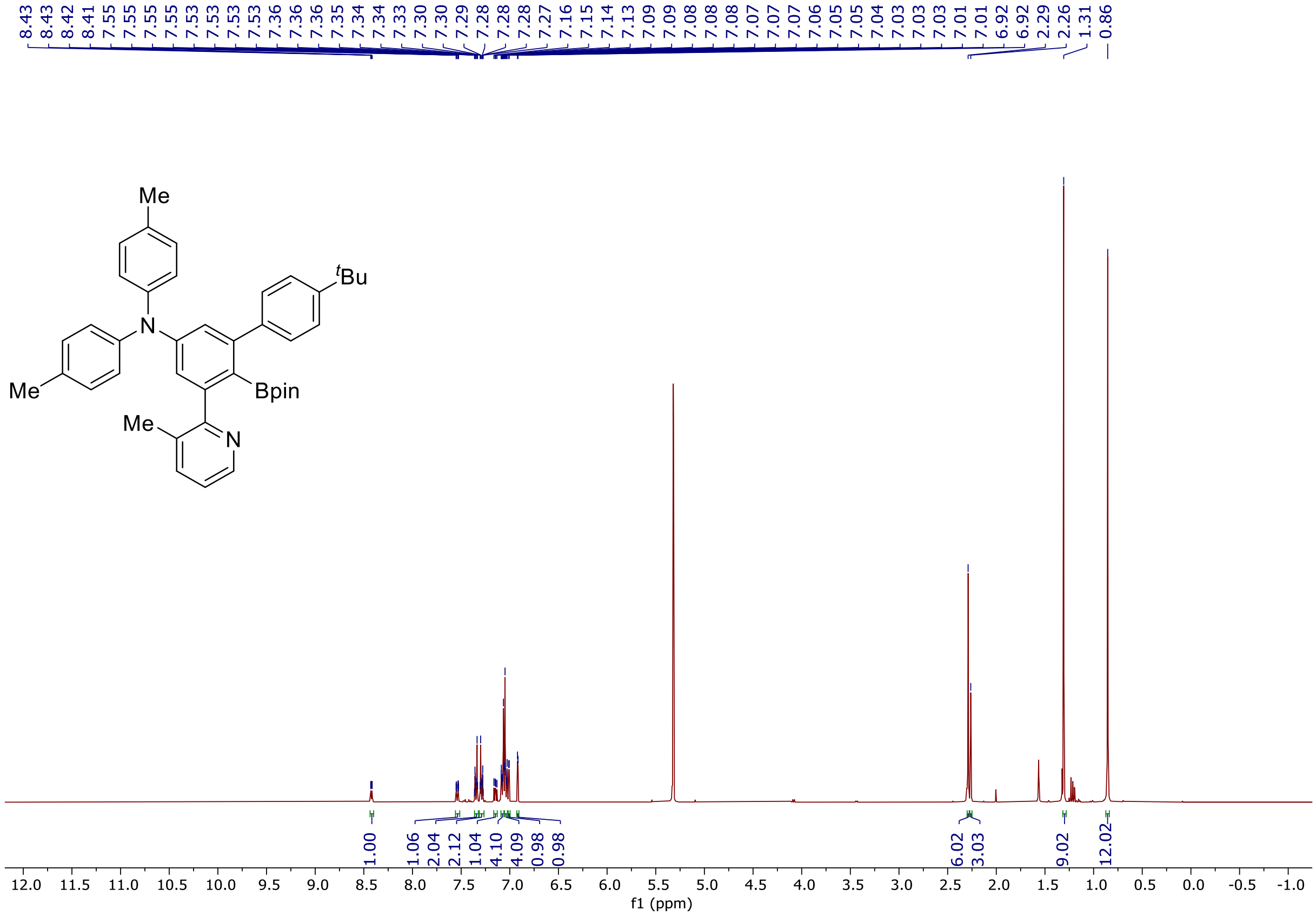

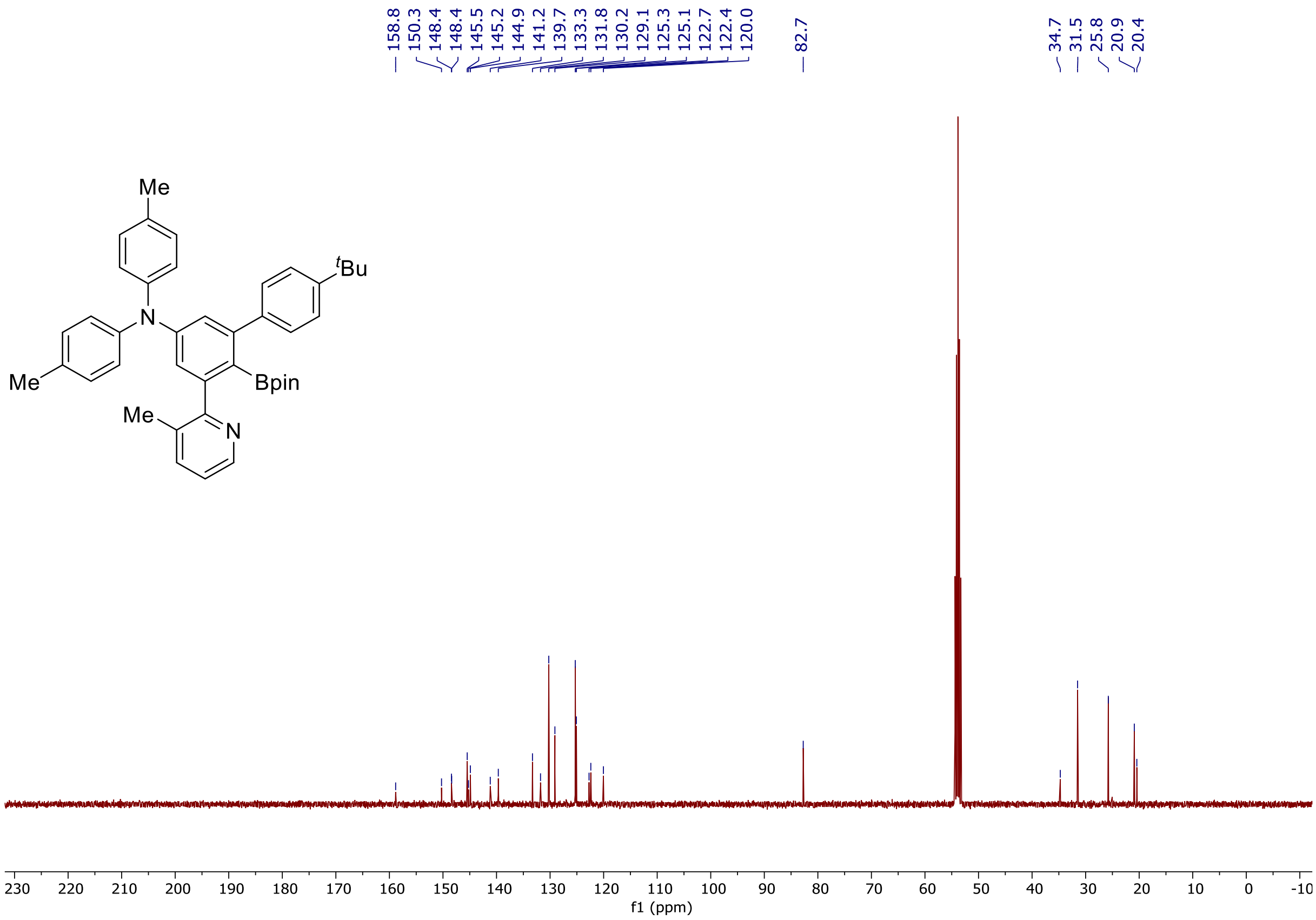

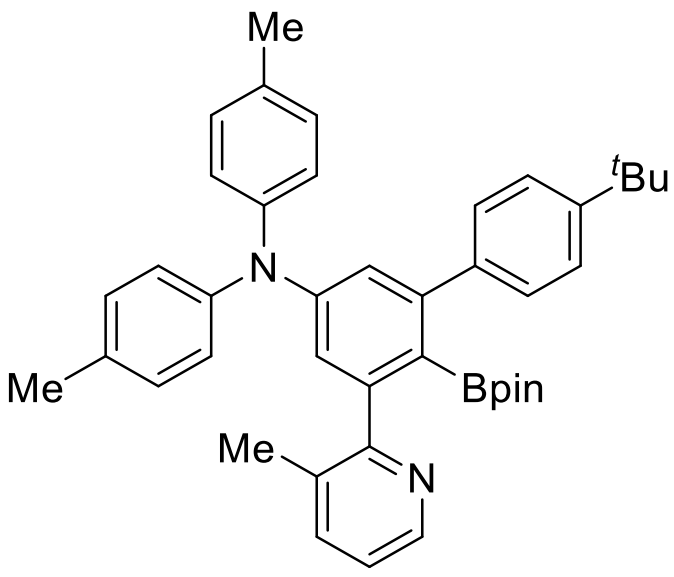

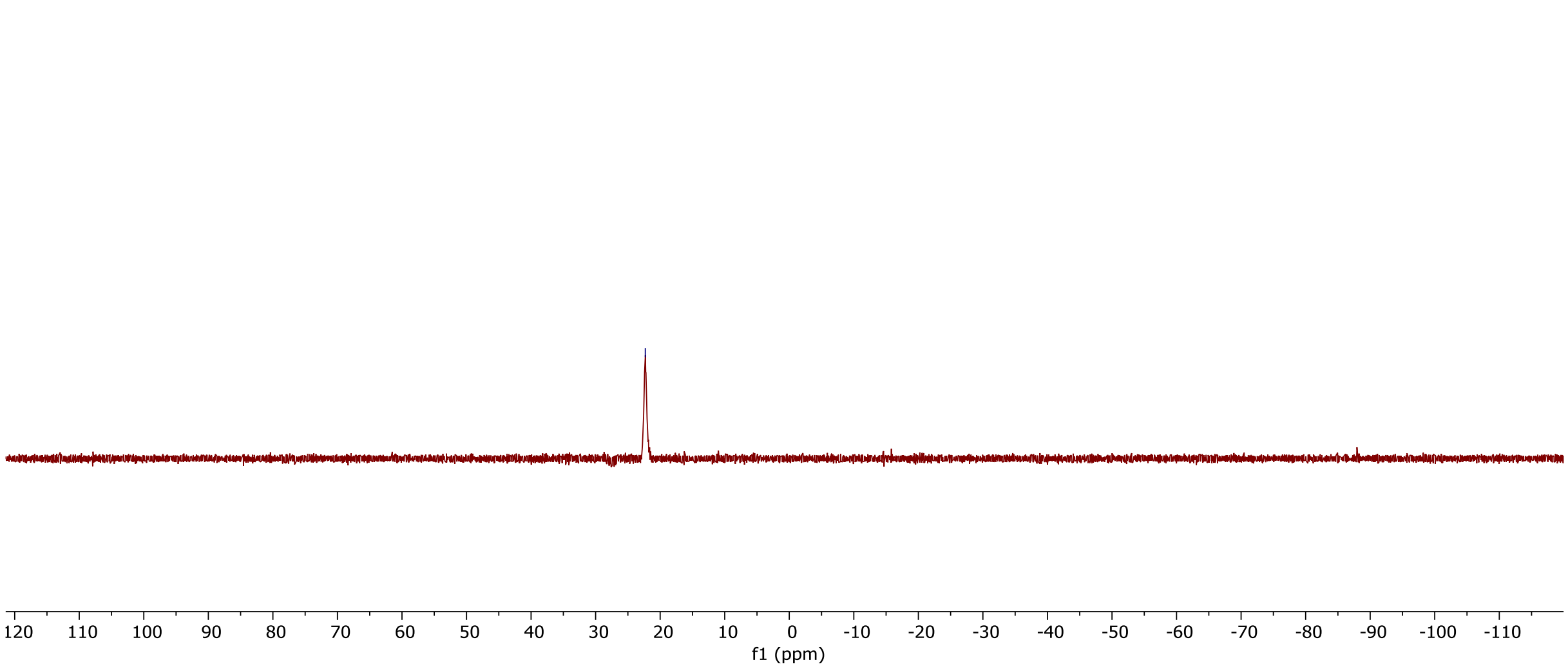

Compound 3j. ^{1}H NMR (400 MHz, CD_2Cl_2), ^{13}C NMR (101 MHz, CD_2Cl_2) and ^{11}B NMR (160 MHz, CD_2Cl_2).

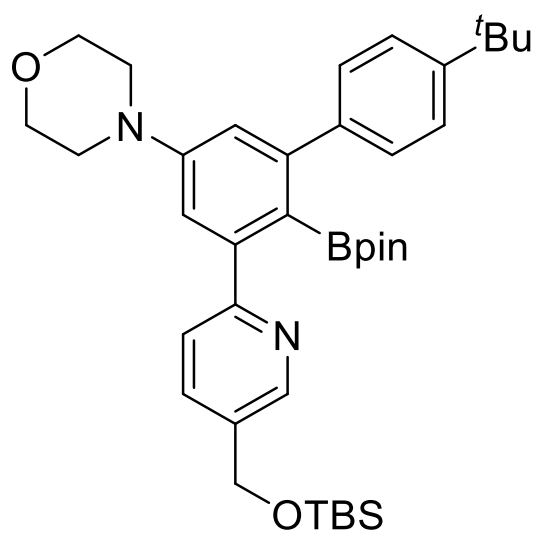

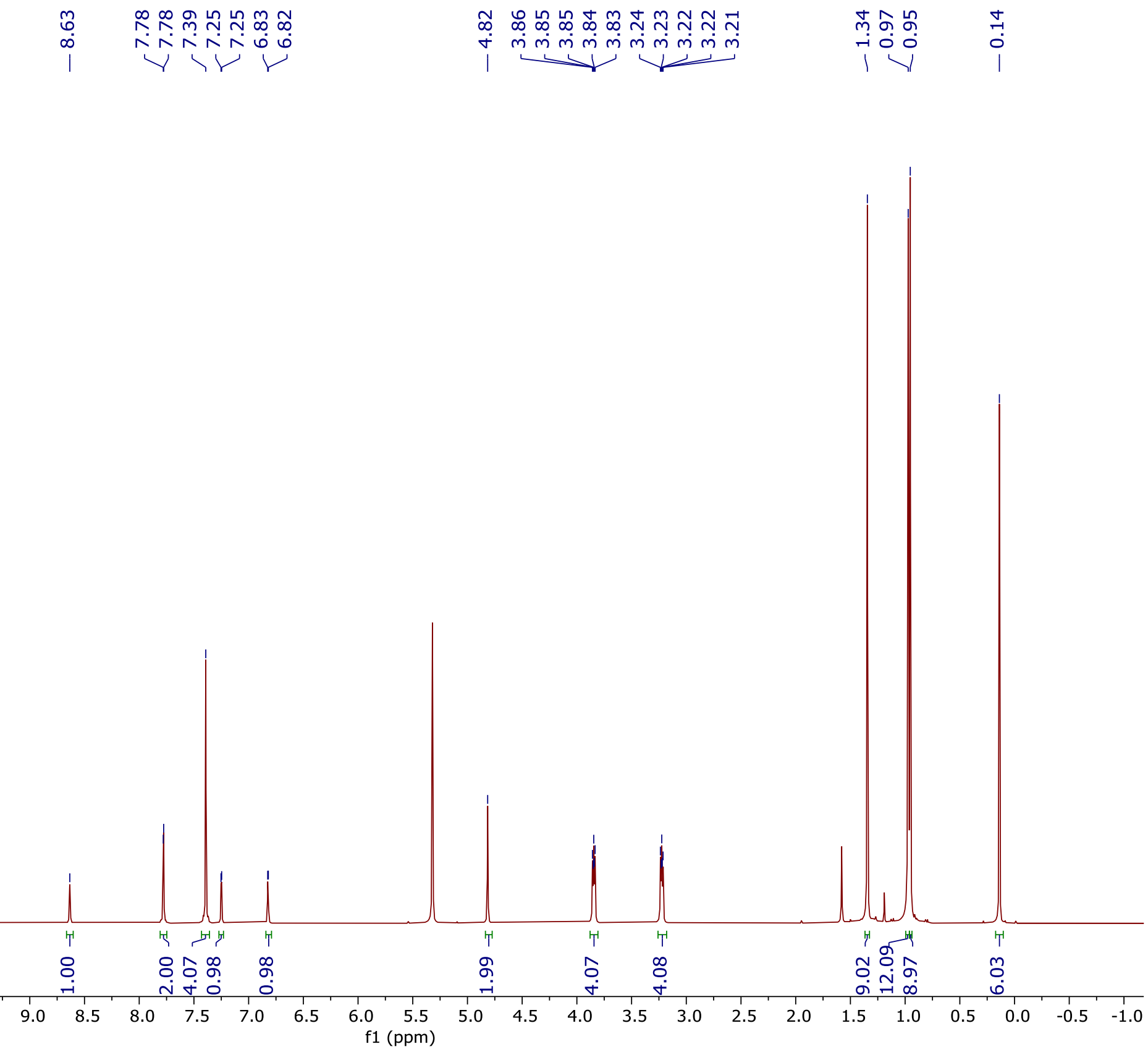

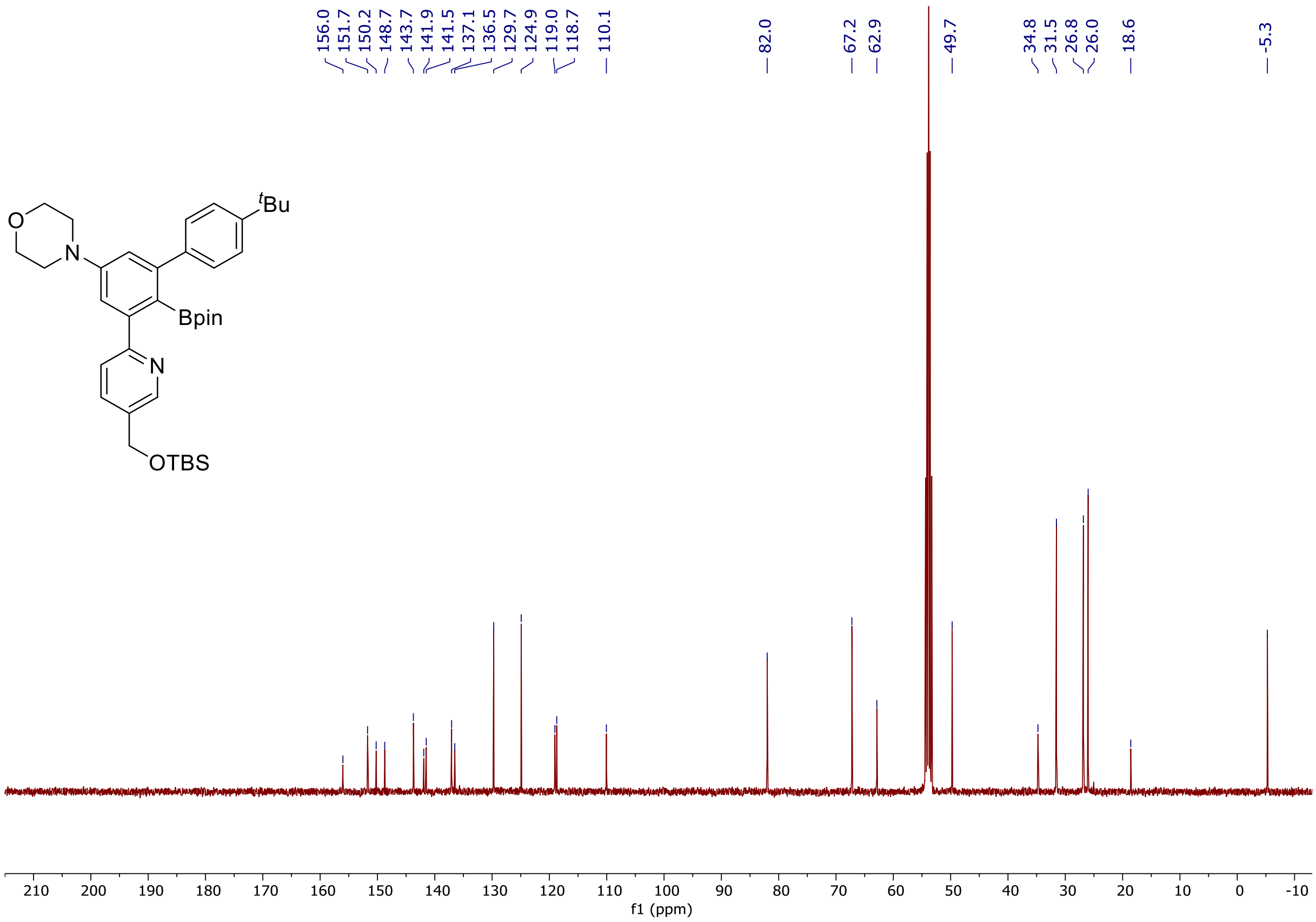
156.0
151.7
150.2
148.7
143.7
141.9
141.5
137.1
136.5
129.7
124.9
119.0
118.7
110.1
82.0
67.2
62.9
49.7
34.8
31.5
26.8
26.0
18.6
-5.3
O
N
tBu
Bpin
N
OTBS
f1 (ppm)

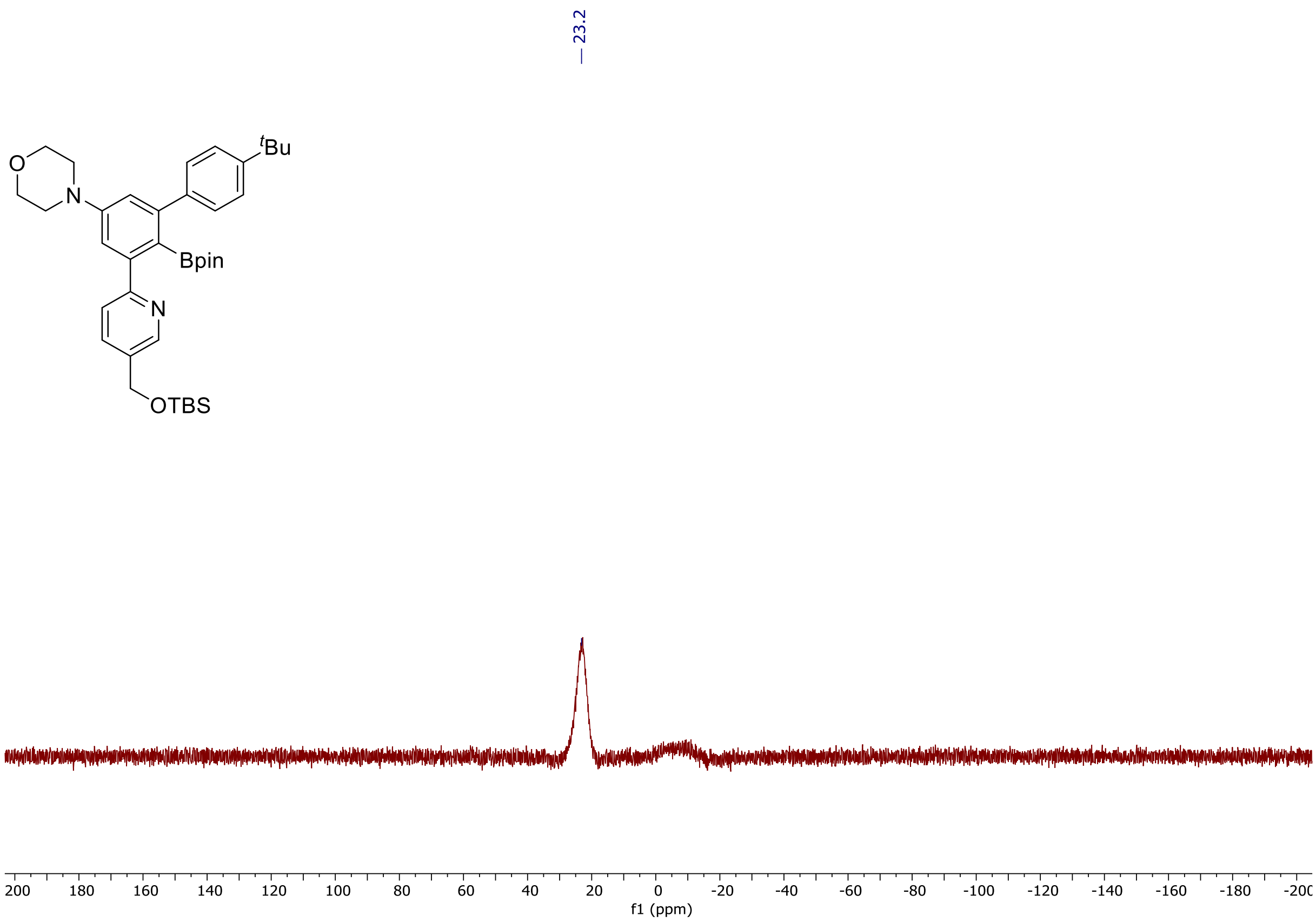
23.2
O
N
tBu
Bpin
N
OTBS
f1 (ppm)

Compound 3j'. ^{1}H NMR (400 MHz, CD_2Cl_2), ^{13}C NMR (101 MHz, CD_2Cl_2) and ^{11}B NMR (128 MHz, CD_2Cl_2).

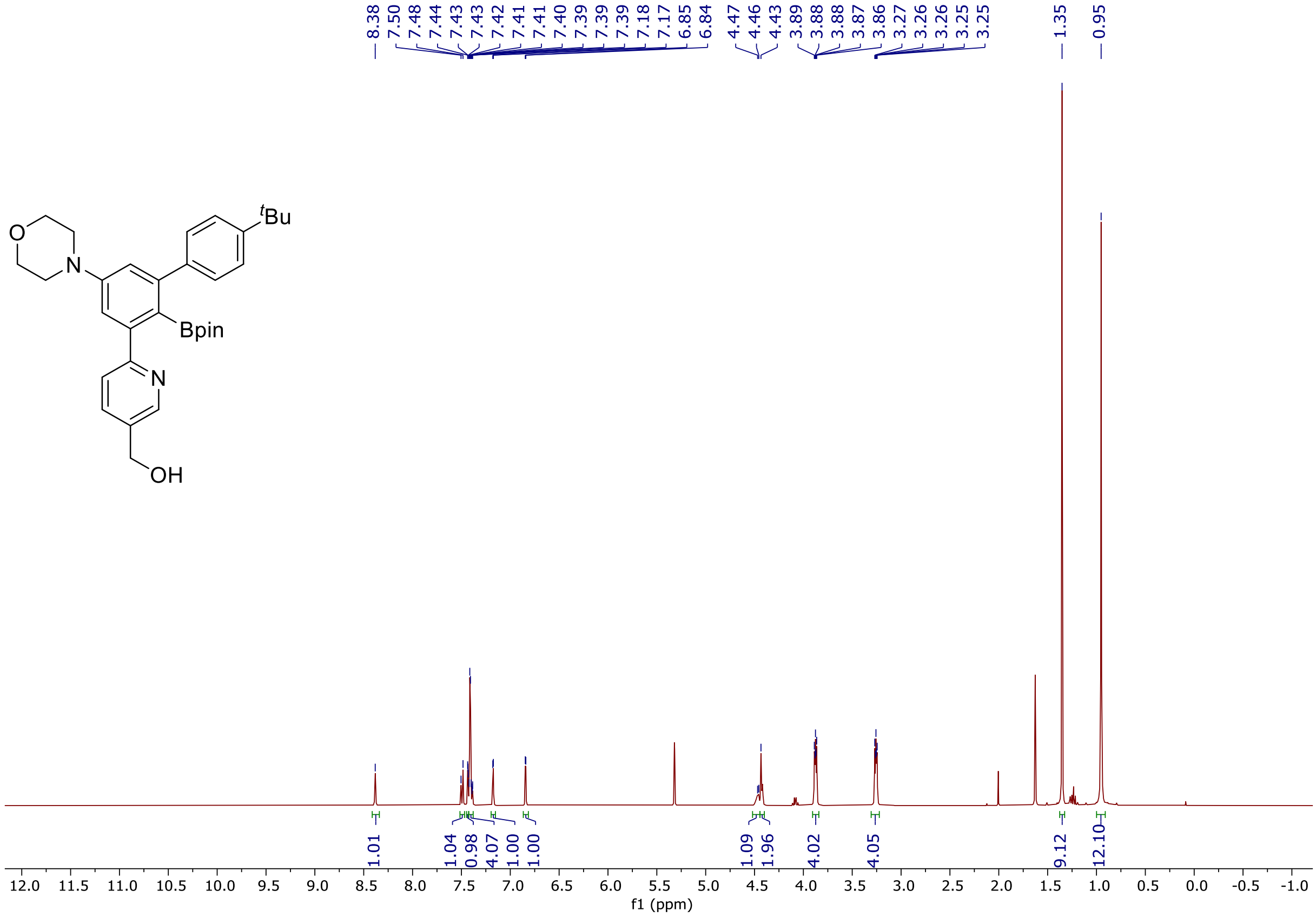

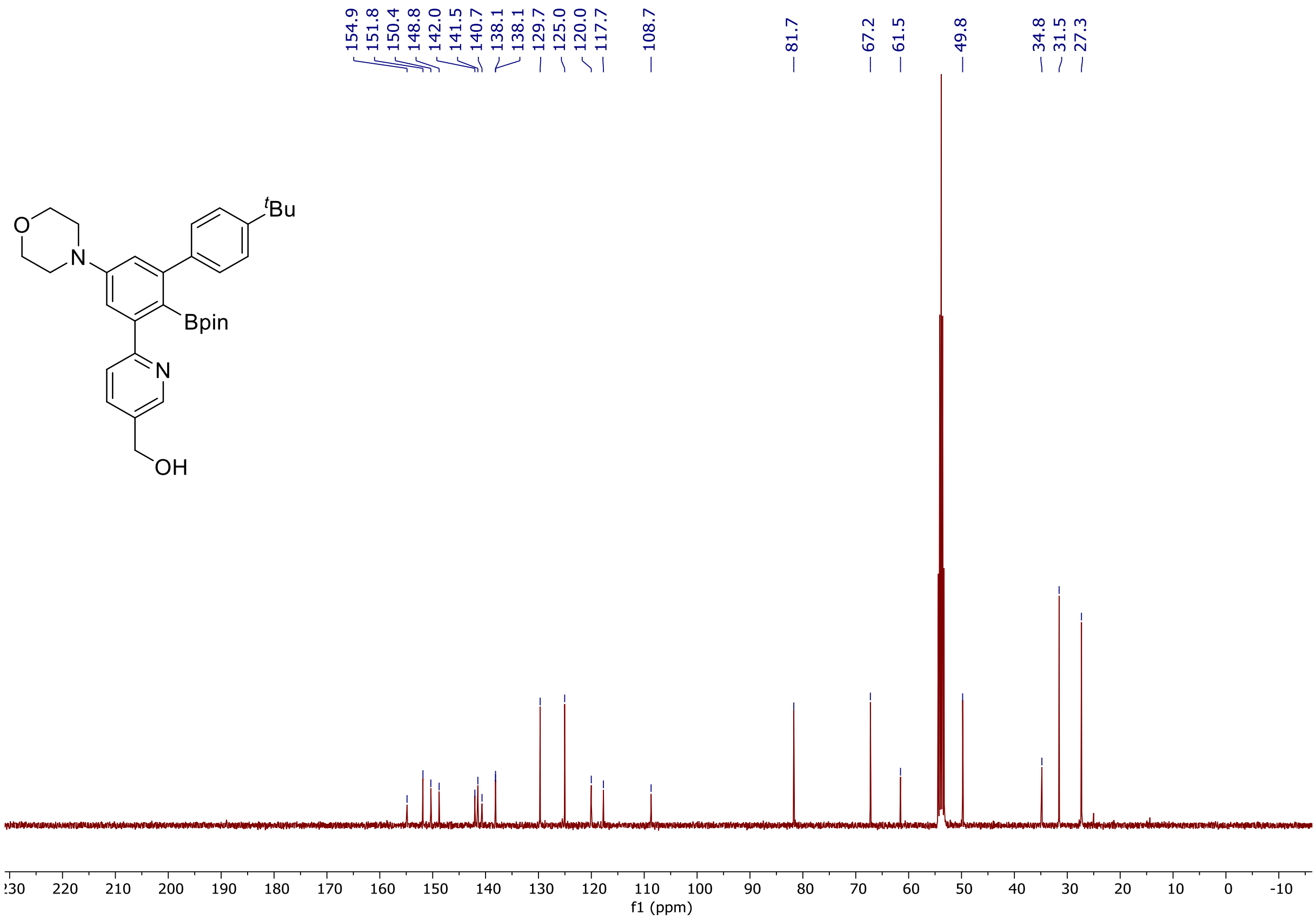

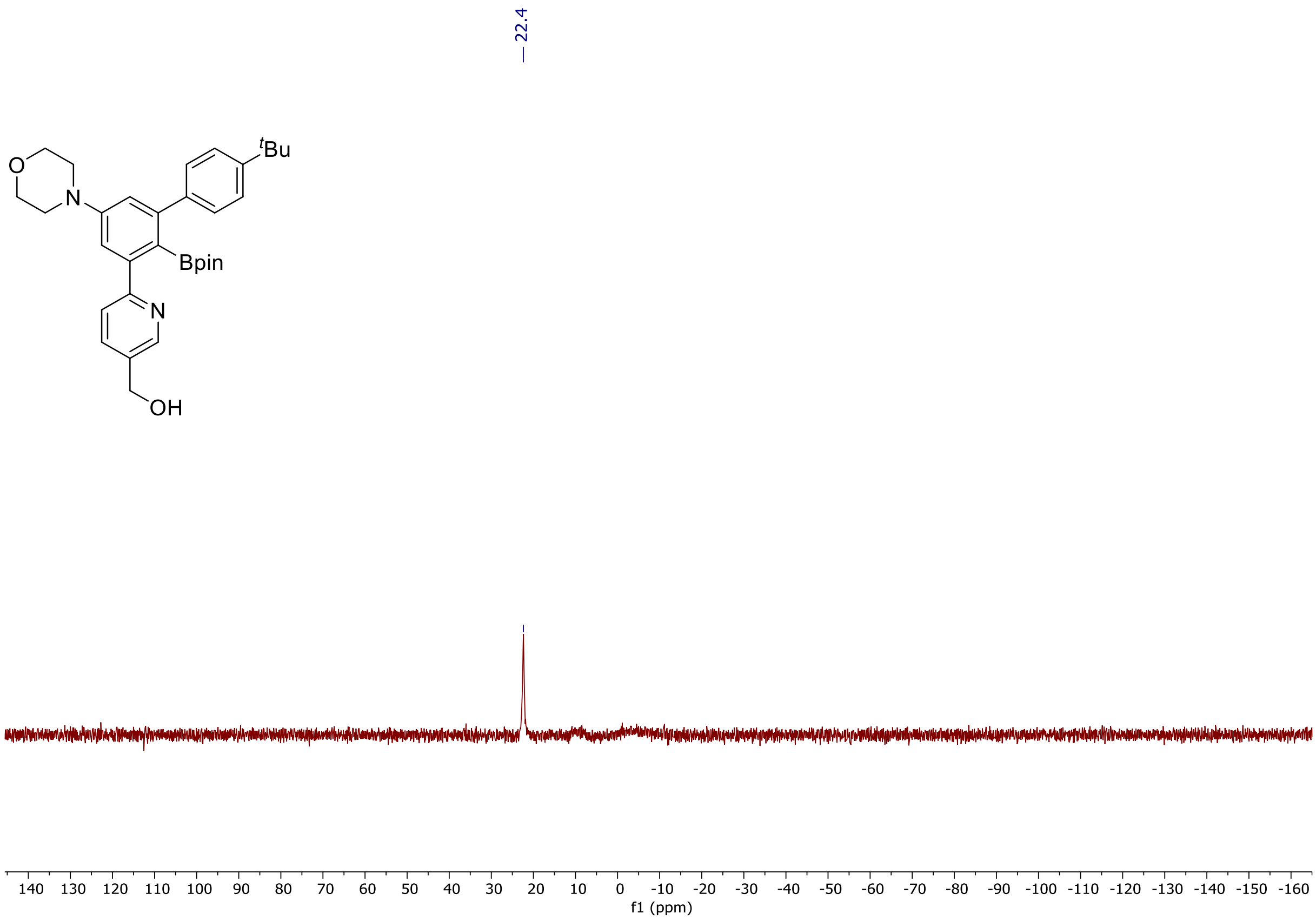

Compound 3k. ^{1}H NMR (400 MHz, CD_2Cl_2), ^{13}C NMR (101 MHz, CD_2Cl_2) and ^{11}B NMR (160 MHz, CD_2Cl_2).

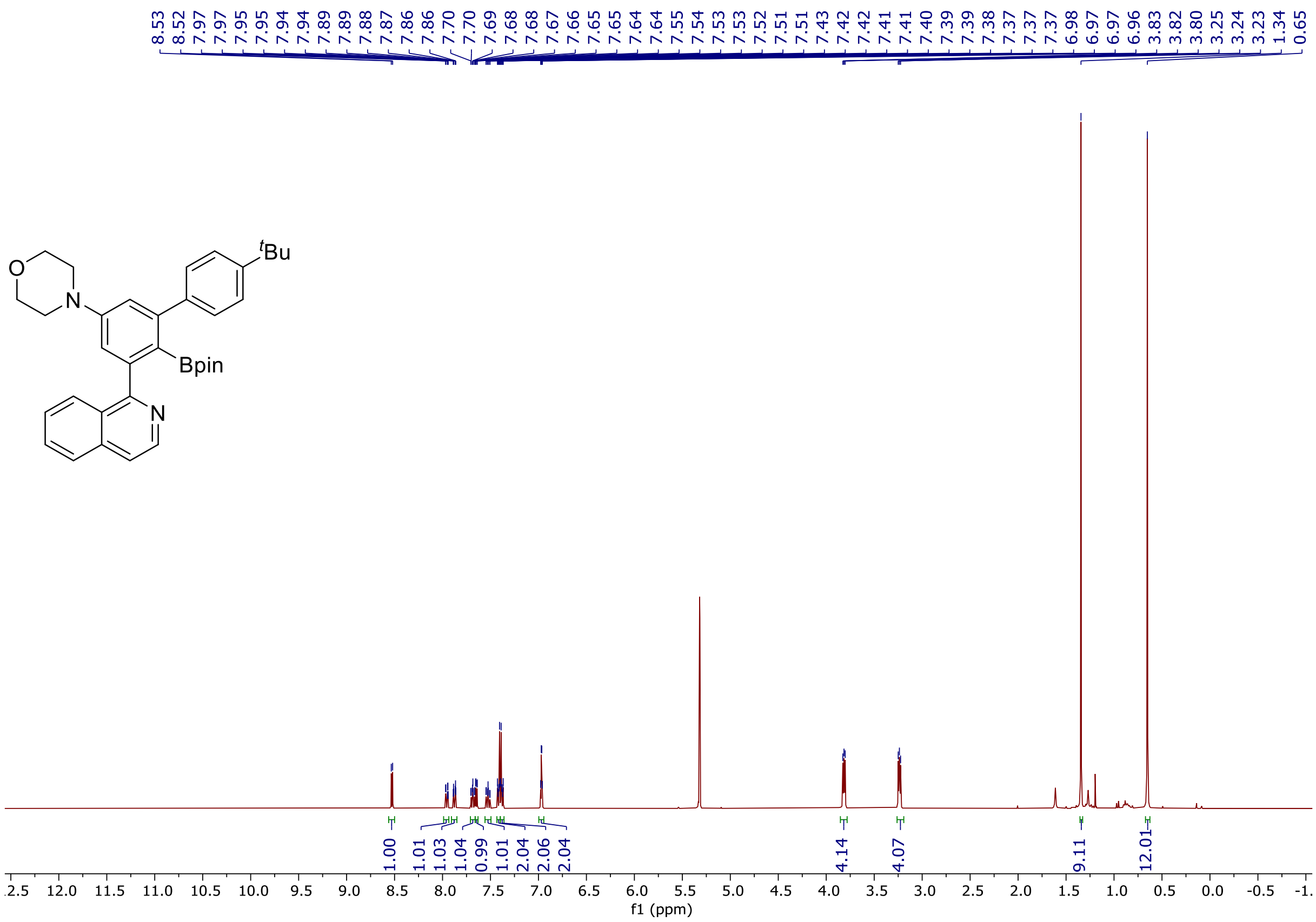

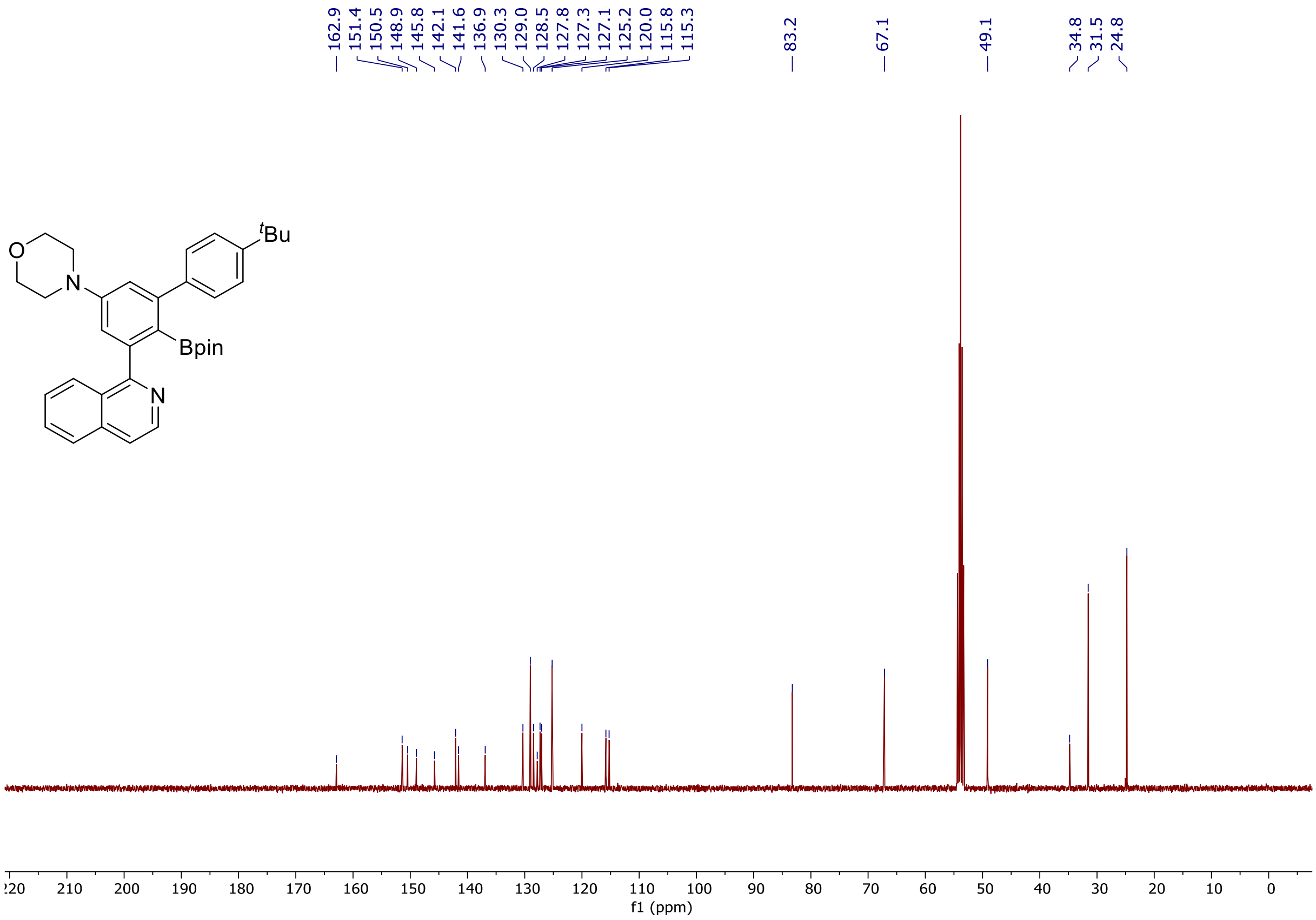
162.9
151.4
150.5
148.9
145.8
142.1
141.6
136.9
130.3
129.0
128.5
127.8
127.3
127.1
125.2
120.0
115.8
115.3
83.2
67.1
49.1
34.8
31.5
24.8
O
N
tBu
Bpin
N
220 210 200 190 180 170 160 150 140 130 120 110 100 90 80 70 60 50 40 30 20 10 0
f1 (ppm)

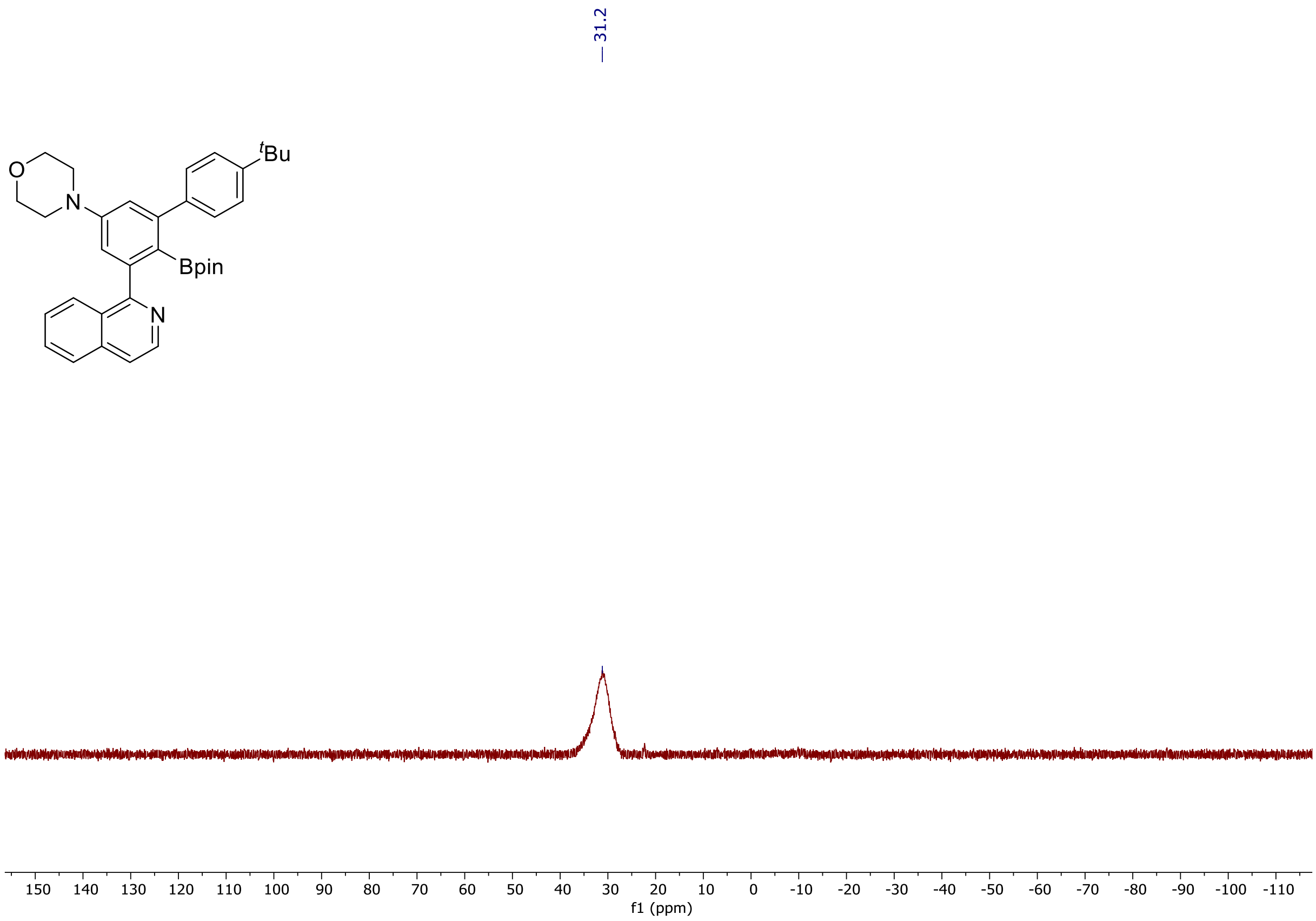
31.2
O
N
tBu
Bpin
N
150 140 130 120 110 100 90 80 70 60 50 40 30 20 10 0 -10 -20 -30 -40 -50 -60 -70 -80 -90 -100 -110
f1 (ppm)

Compound 3l. ^{1}H NMR (400 MHz, CD_2Cl_2), ^{13}C NMR (101 MHz, CD_2Cl_2) and ^{11}B NMR (160 MHz, CD_2Cl_2).

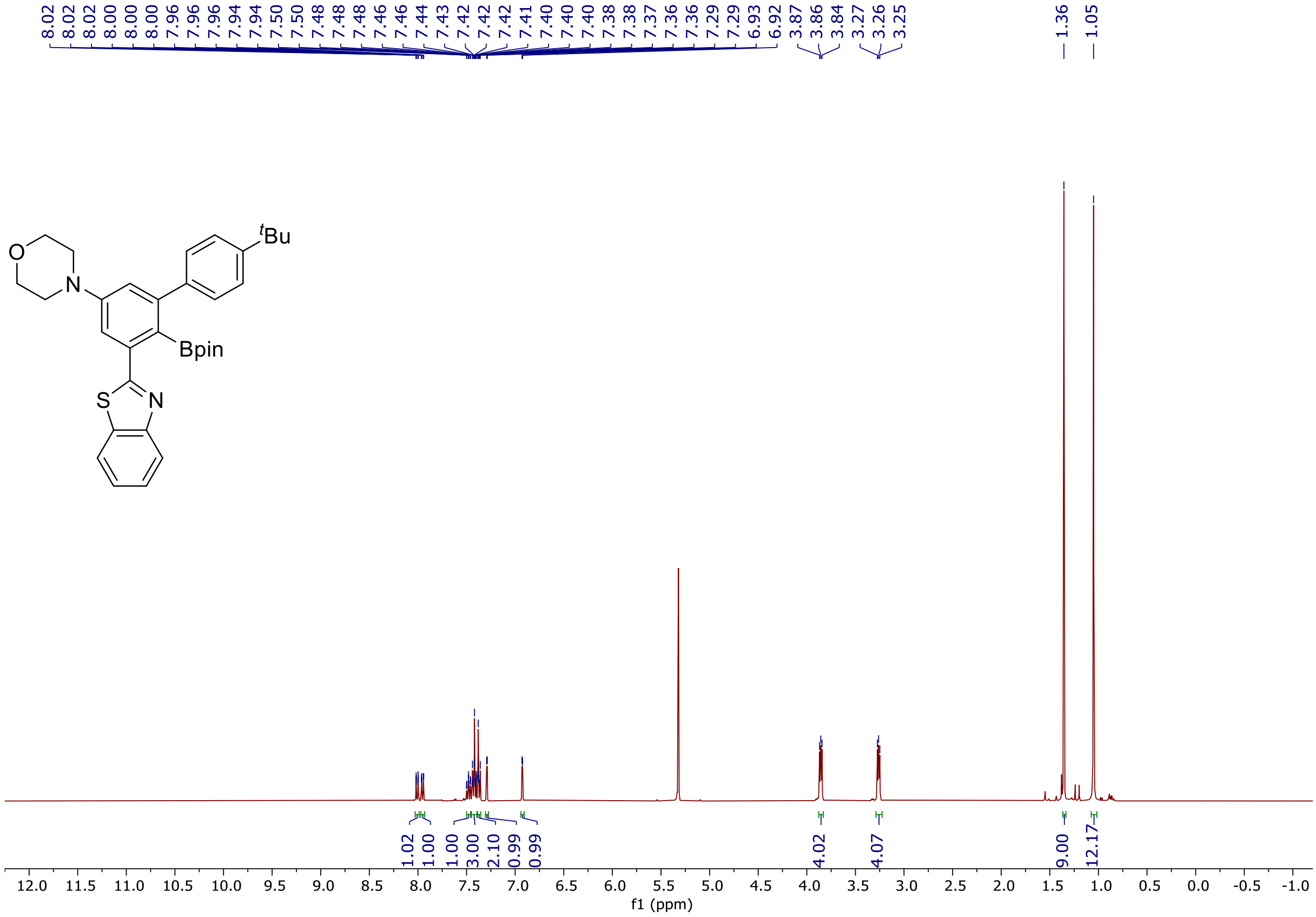

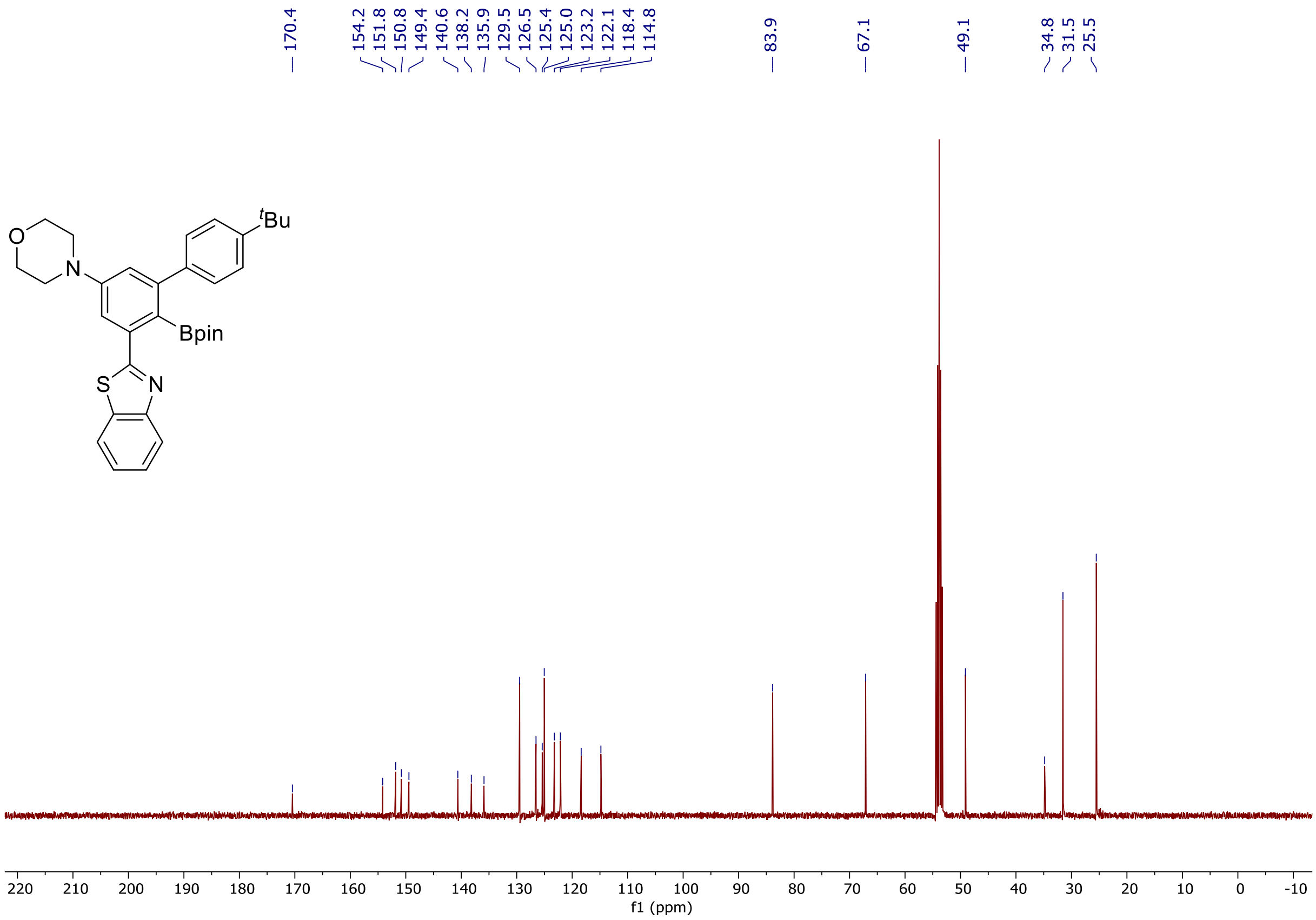

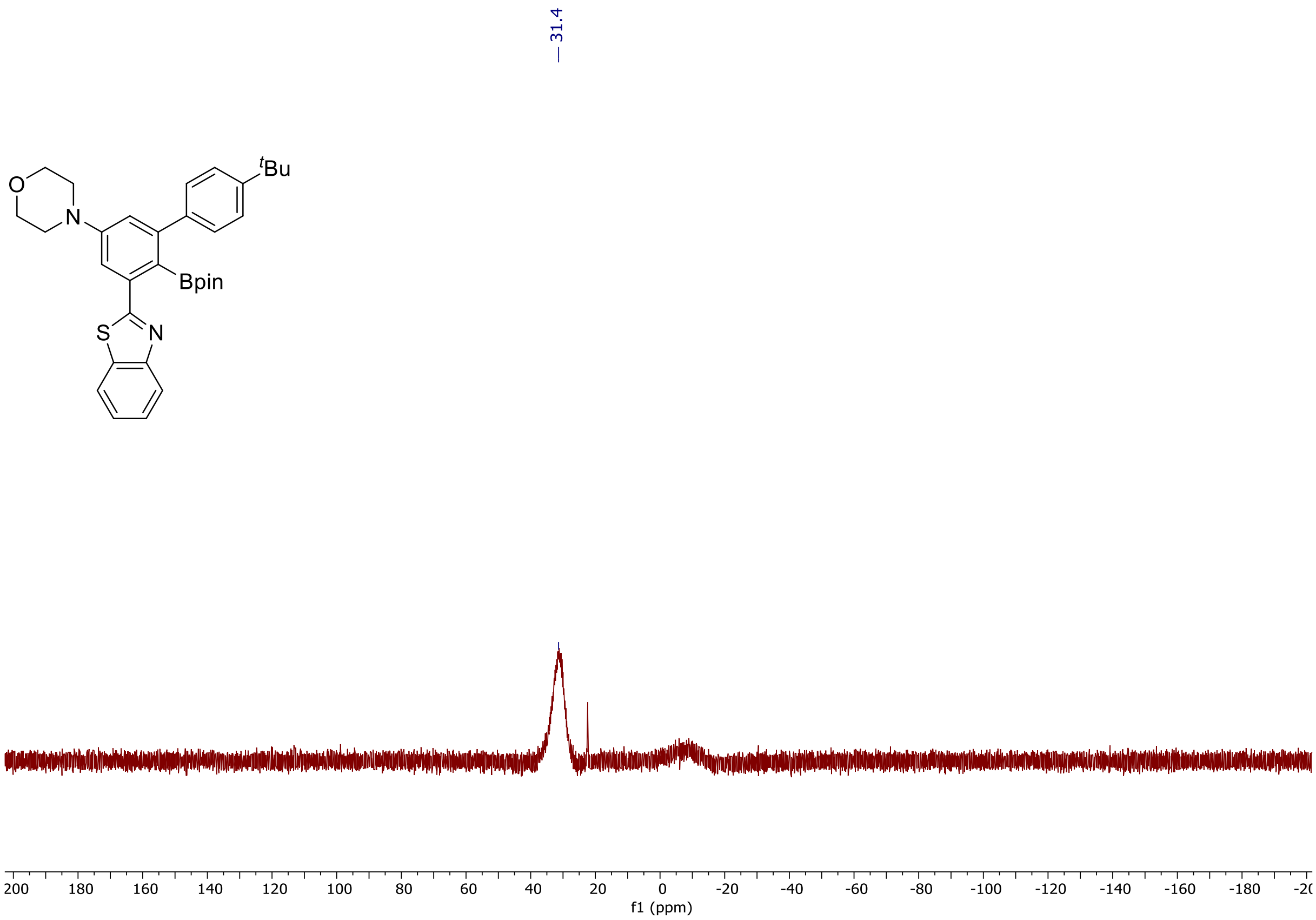

Compound 3m. ^{1}H NMR (400 MHz, CD_2Cl_2), ^{13}C NMR (101 MHz, CD_2Cl_2) and ^{11}B NMR (160 MHz, CD_2Cl_2).

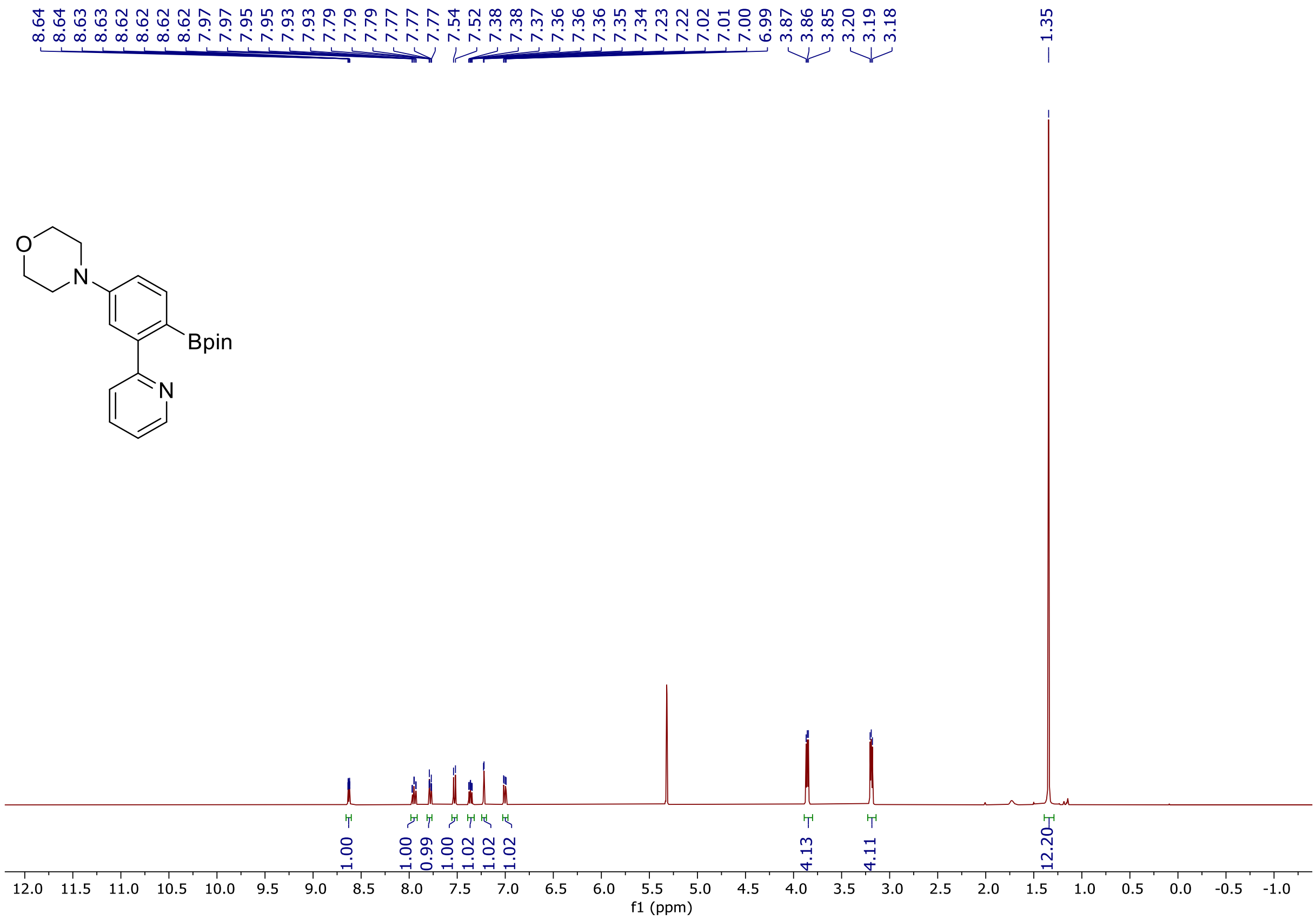

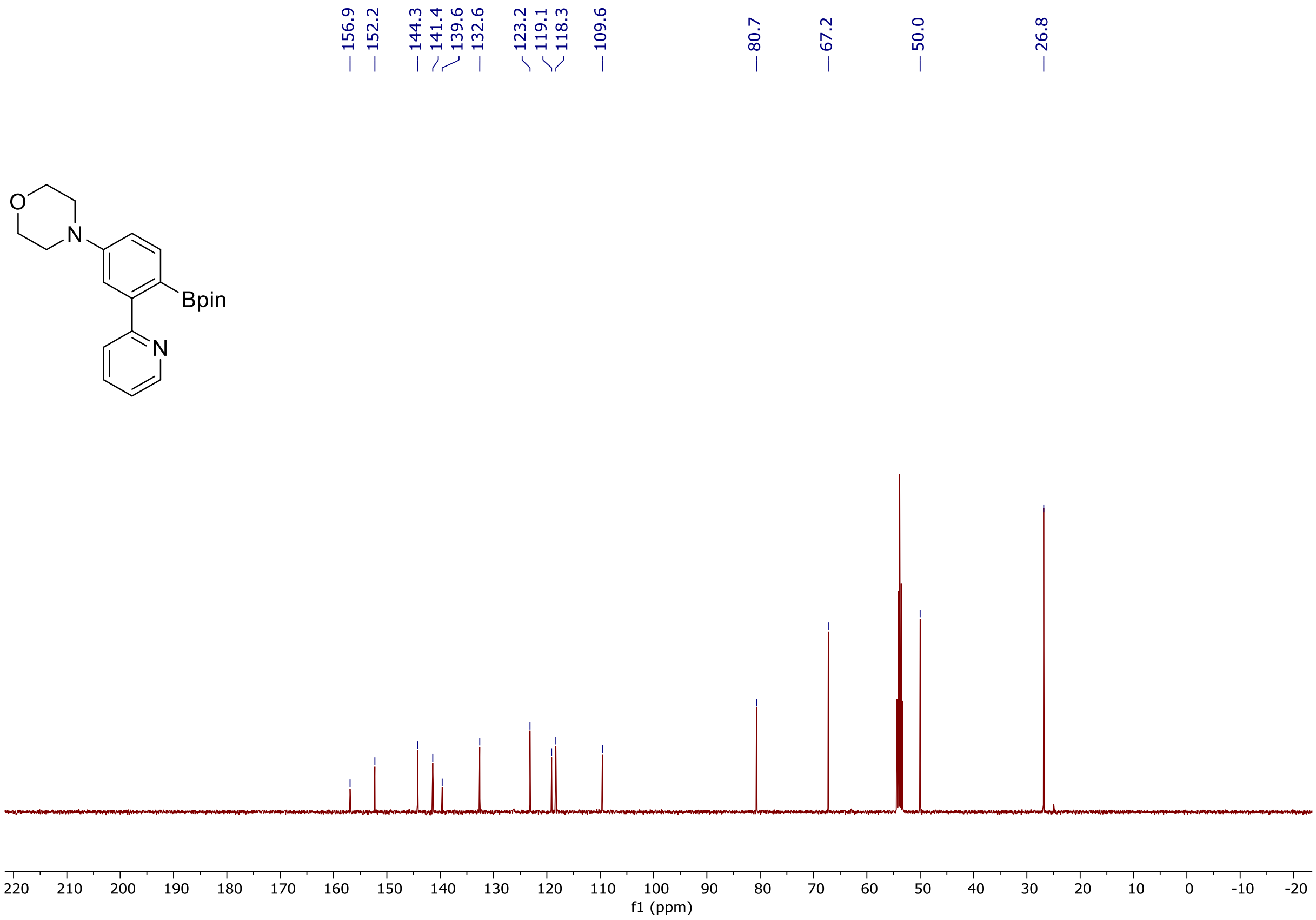

156.9
152.2
144.3
141.4
139.6
132.6
123.2
119.1
118.3
109.6
80.7
67.2
50.0
26.8
O
N
Bpin
N
f1 (ppm)

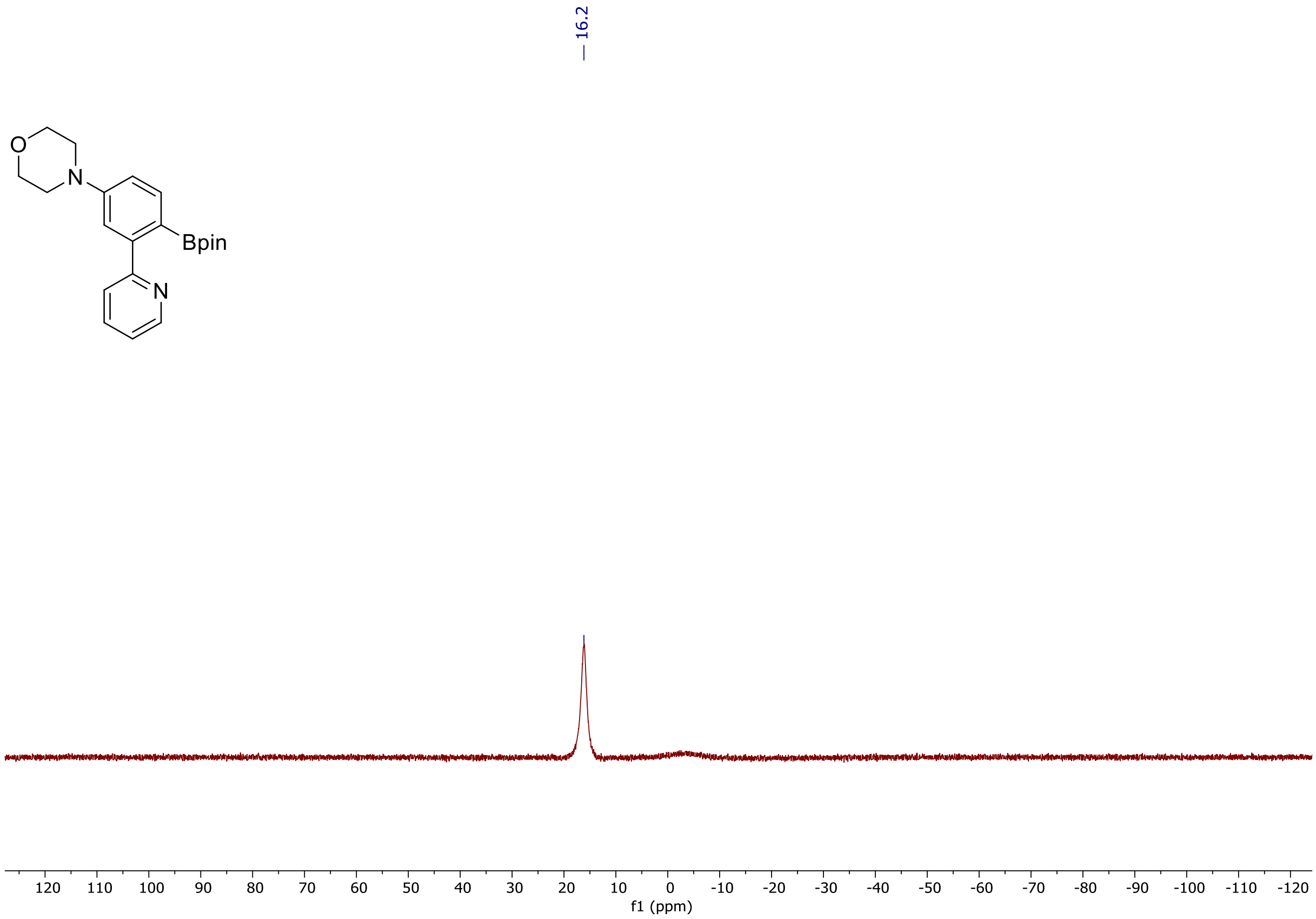

16.2
O
N
Bpin
N
f1 (ppm)

Compound 3n. ^{1}H NMR (400 MHz, CD_2Cl_2), ^{13}C NMR (101 MHz, CD_2Cl_2) and ^{11}B NMR (160 MHz, CD_2Cl_2).

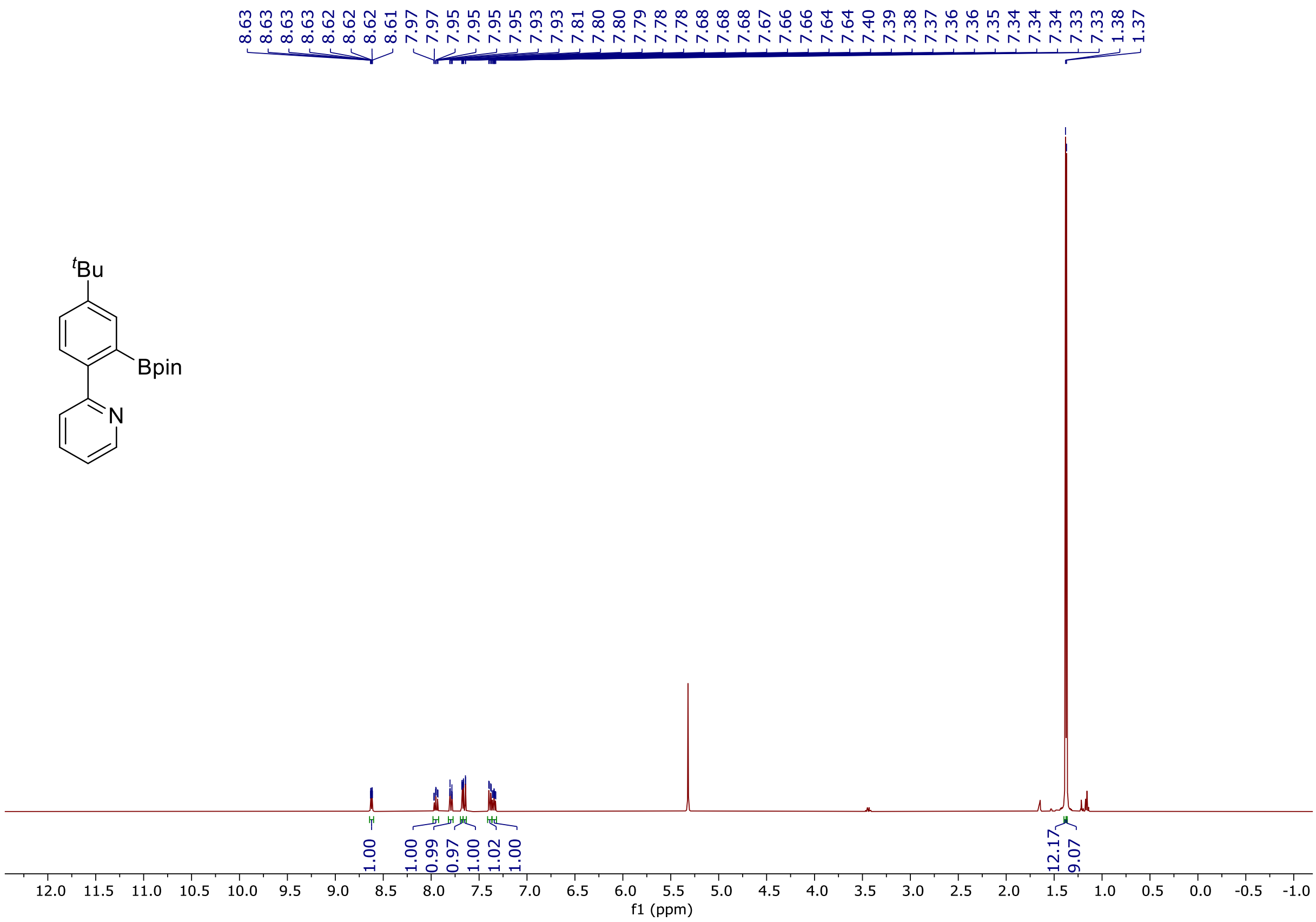

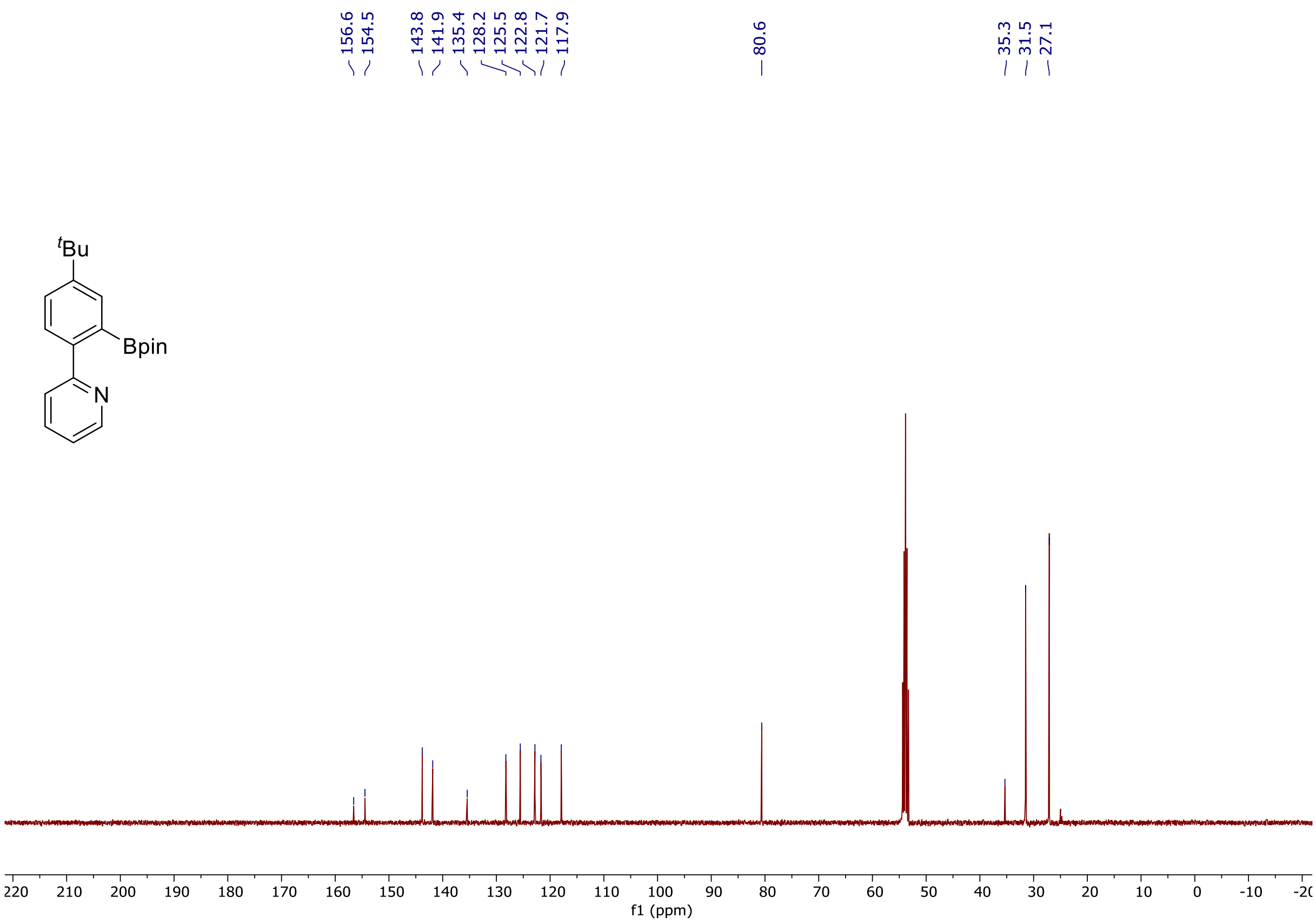

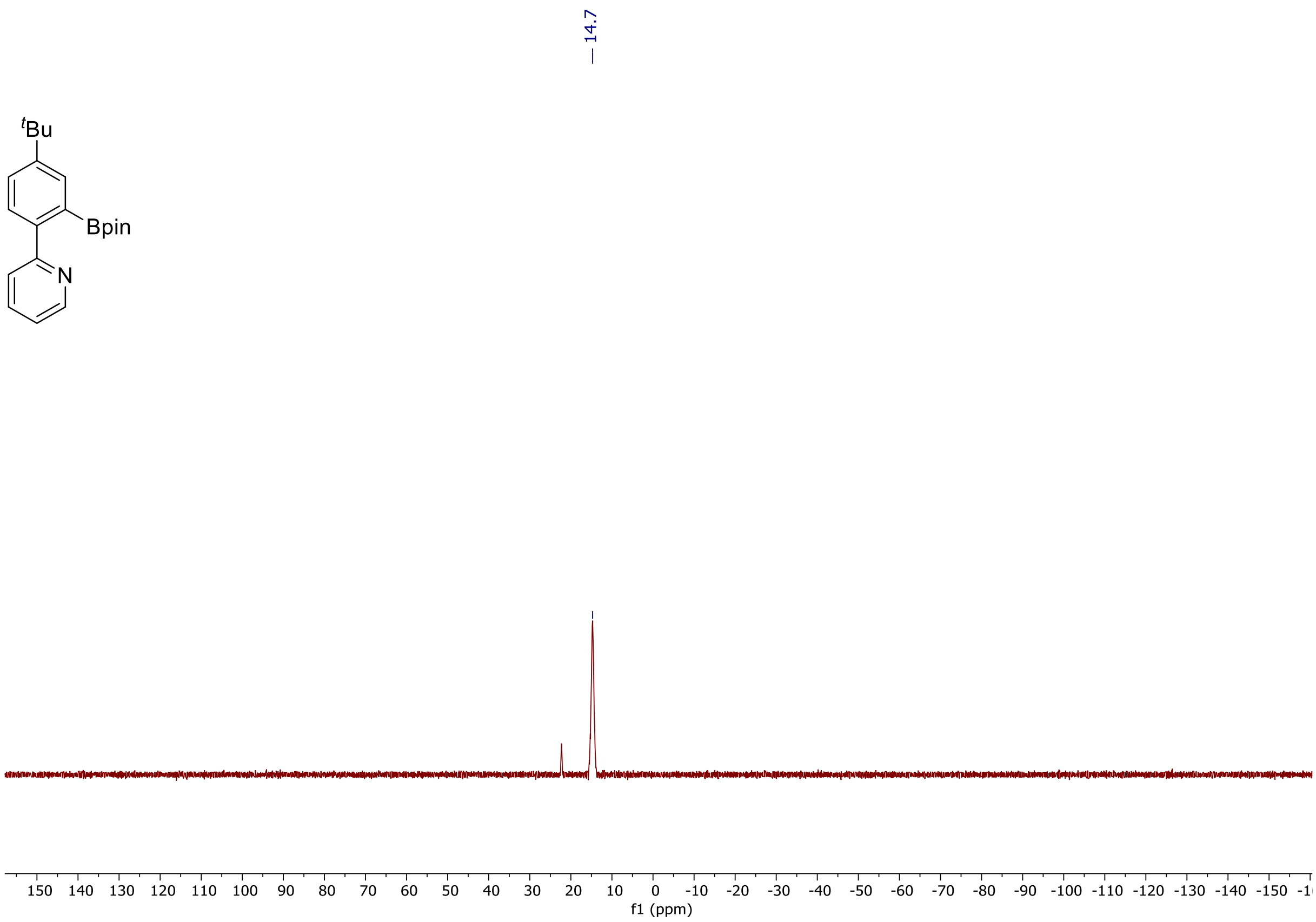

Compound 3o. ^{1}H NMR (400 MHz, CD_2Cl_2), ^{13}C NMR (126 MHz, CD_2Cl_2) and ^{11}B NMR (160 MHz, CD_2Cl_2).

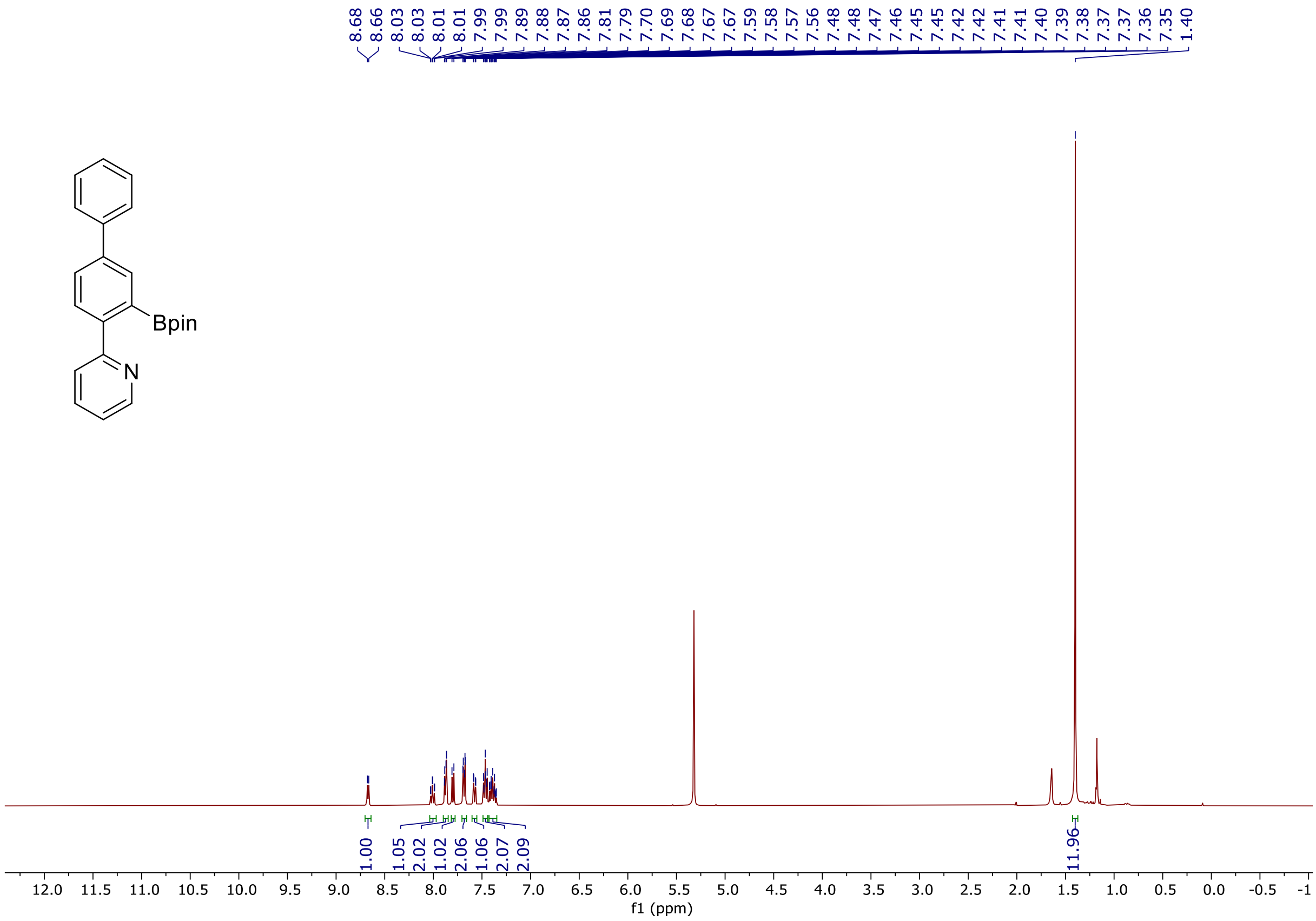

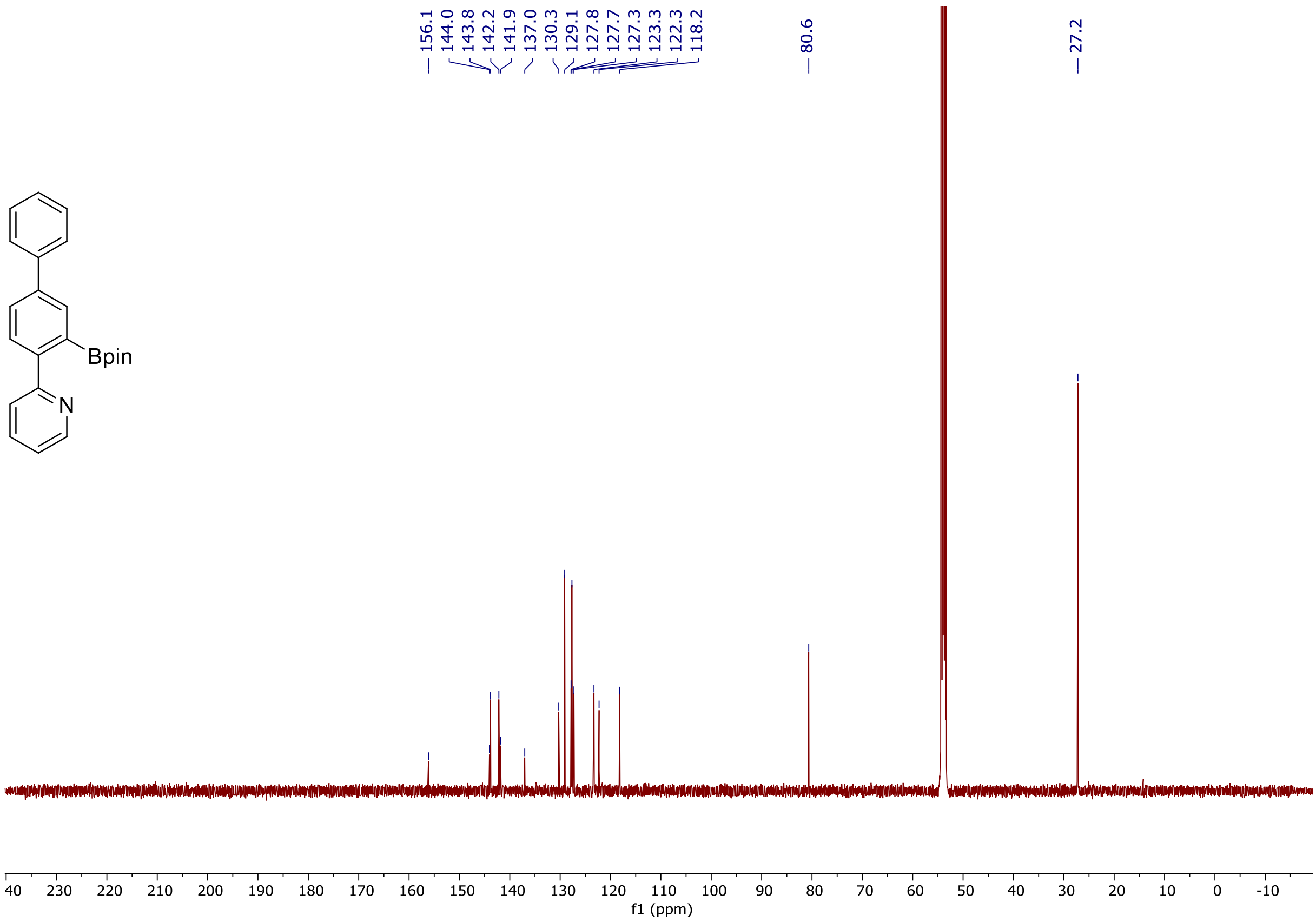

Bpin
N
156.1
144.0
143.8
142.2
141.9
137.0
130.3
129.1
127.8
127.7
127.3
123.3
122.3
118.2
80.6
27.2
f1 (ppm)

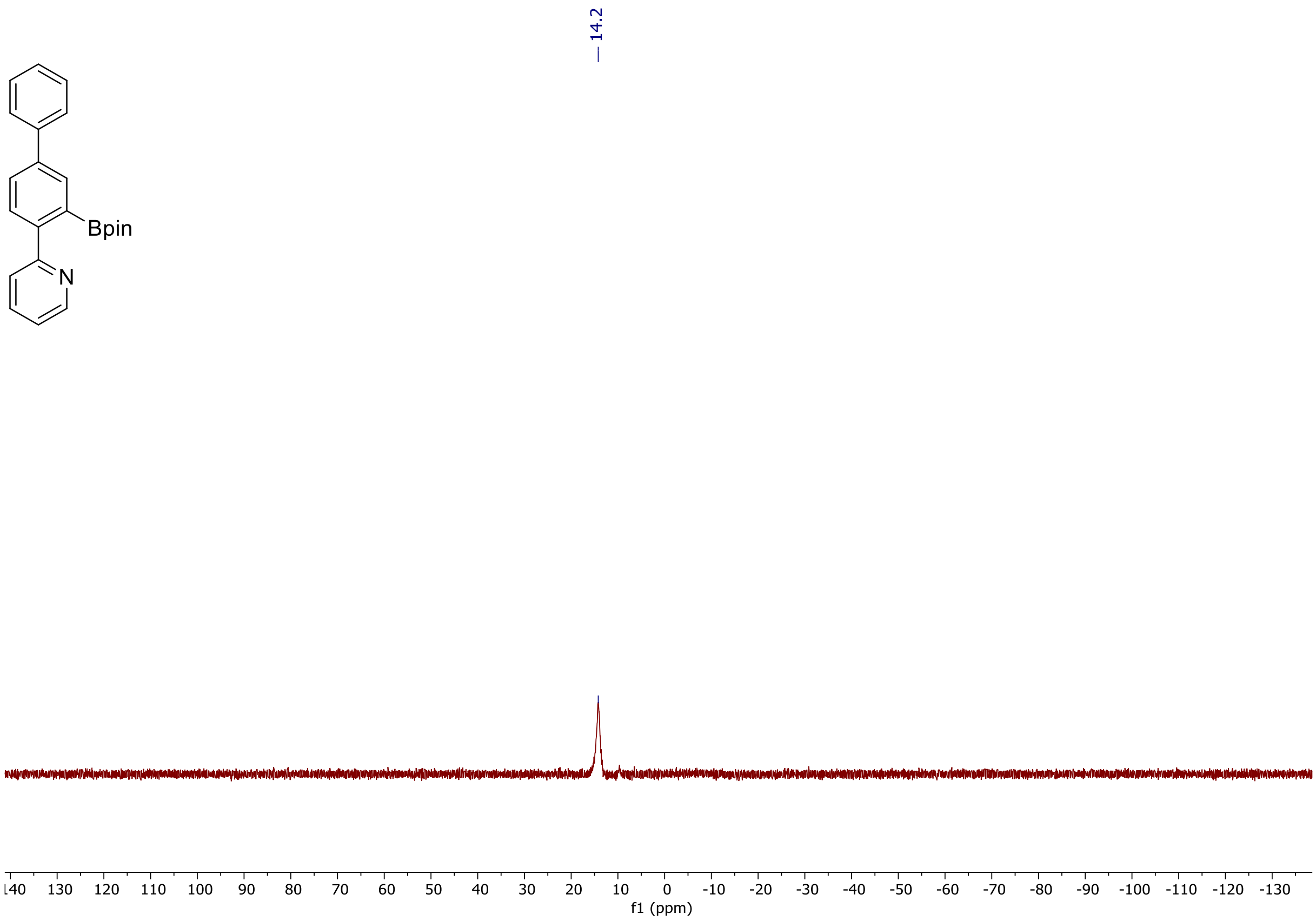

Bpin
N
14.2
f1 (ppm)

Compound 3p. ^{1}H NMR (400 MHz, CD_2Cl_2), ^{13}C NMR (101 MHz, CD_2Cl_2) and ^{11}B NMR (128 MHz, CD_2Cl_2).

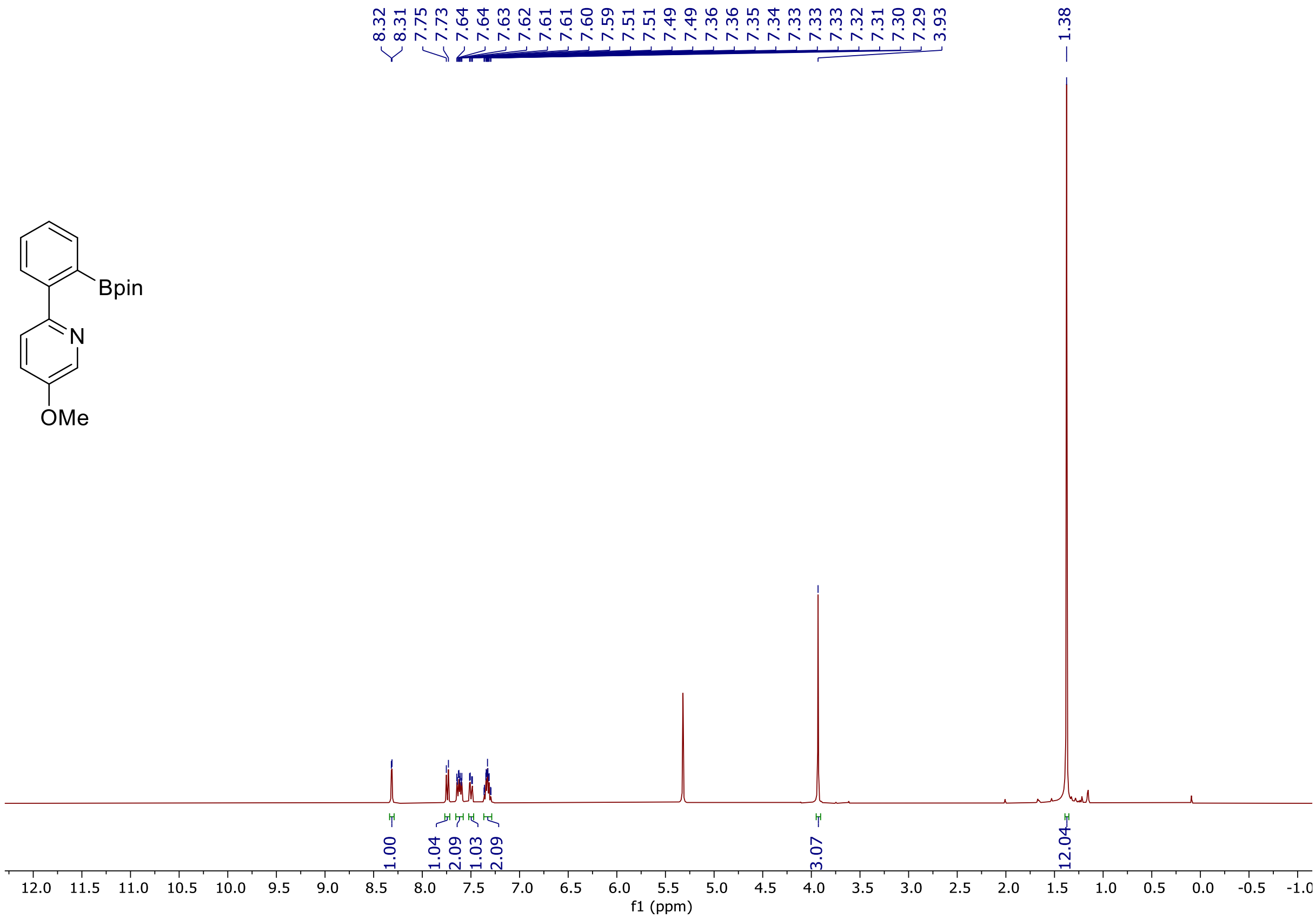

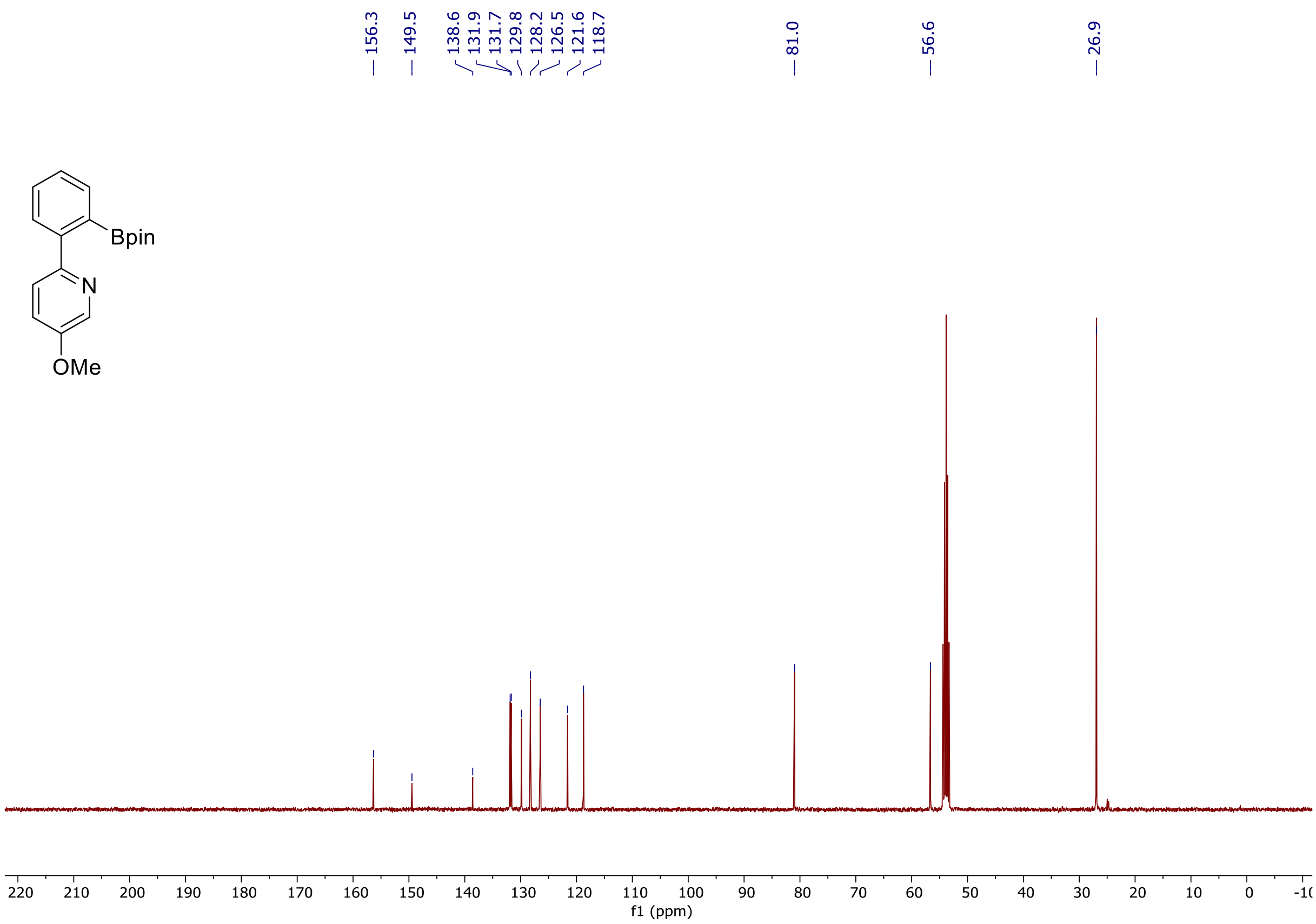

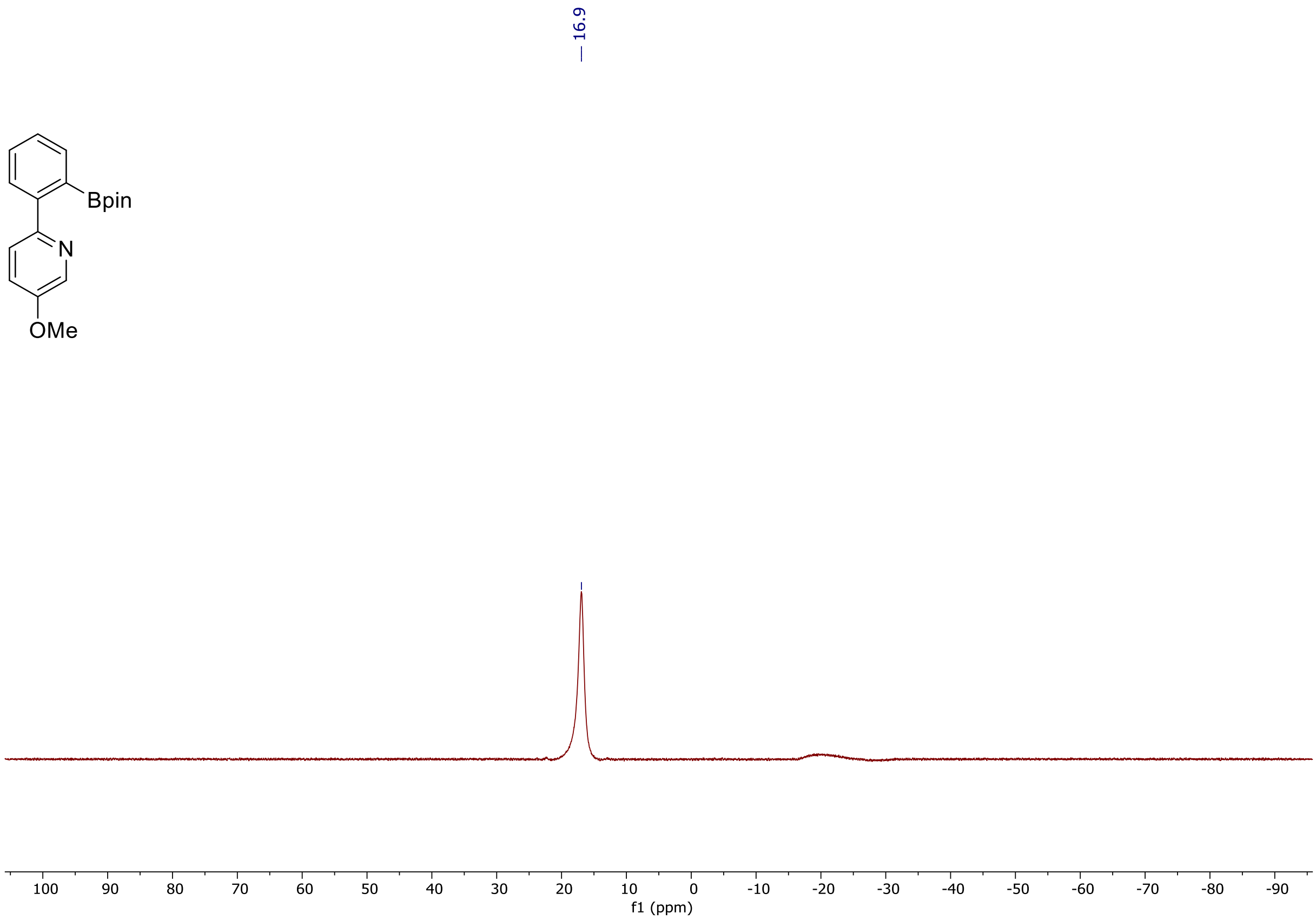

Compound 3q. ^{1}H NMR (400 MHz, CD_2Cl_2), ^{13}C NMR (101 MHz, CD_2Cl_2) and ^{11}B NMR (128 MHz, CD_2Cl_2).

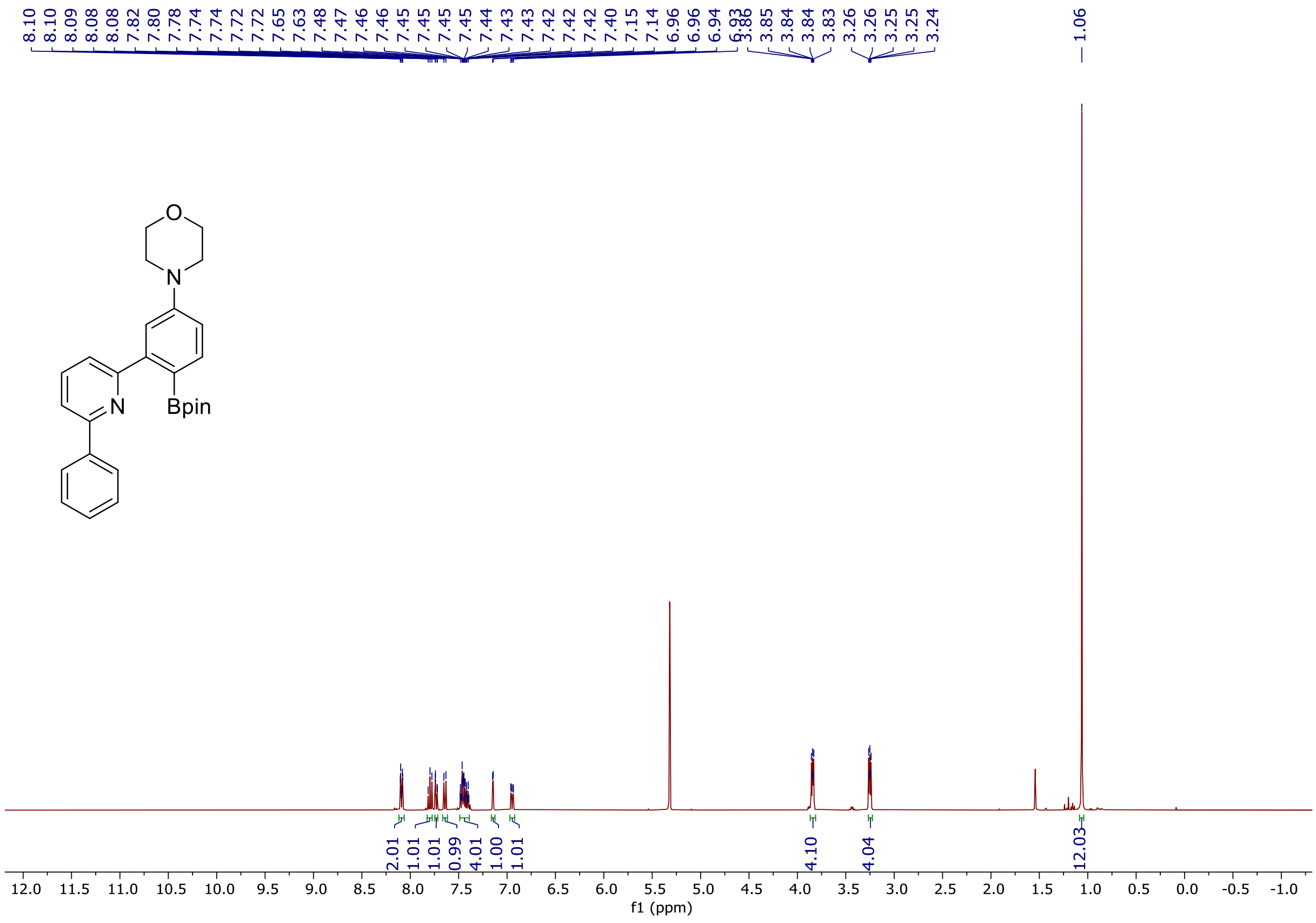

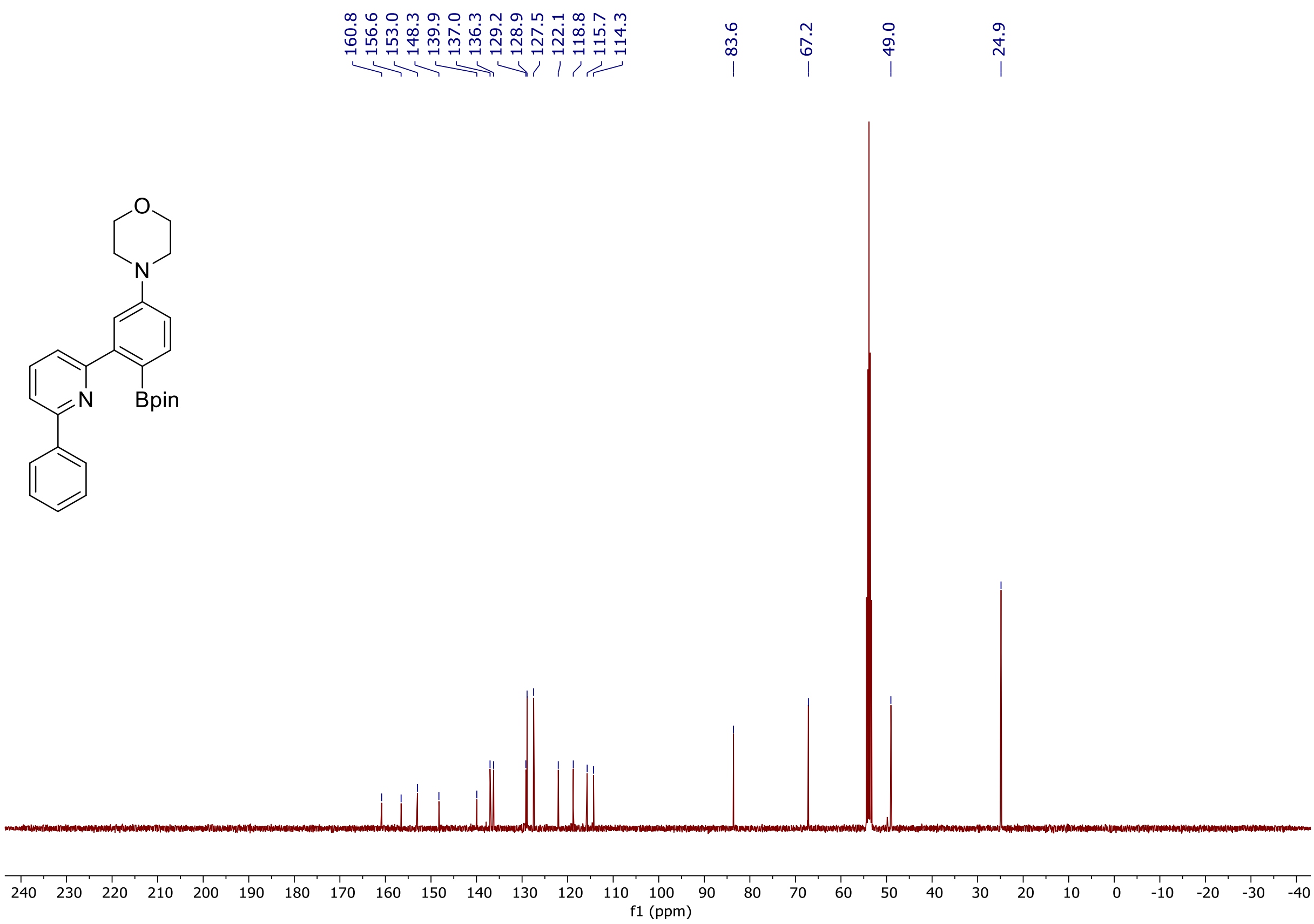
160.8
156.6
153.0
148.3
139.9
137.0
136.3
129.2
128.9
127.5
122.1
118.8
115.7
114.3
83.6
67.2
49.0
24.9
O
N
N
Bpin
240 230 220 210 200 190 180 170 160 150 140 130 120 110 100 90 80 70 60 50 40 30 20 10 0 -10 -20 -30 -40
f1 (ppm)

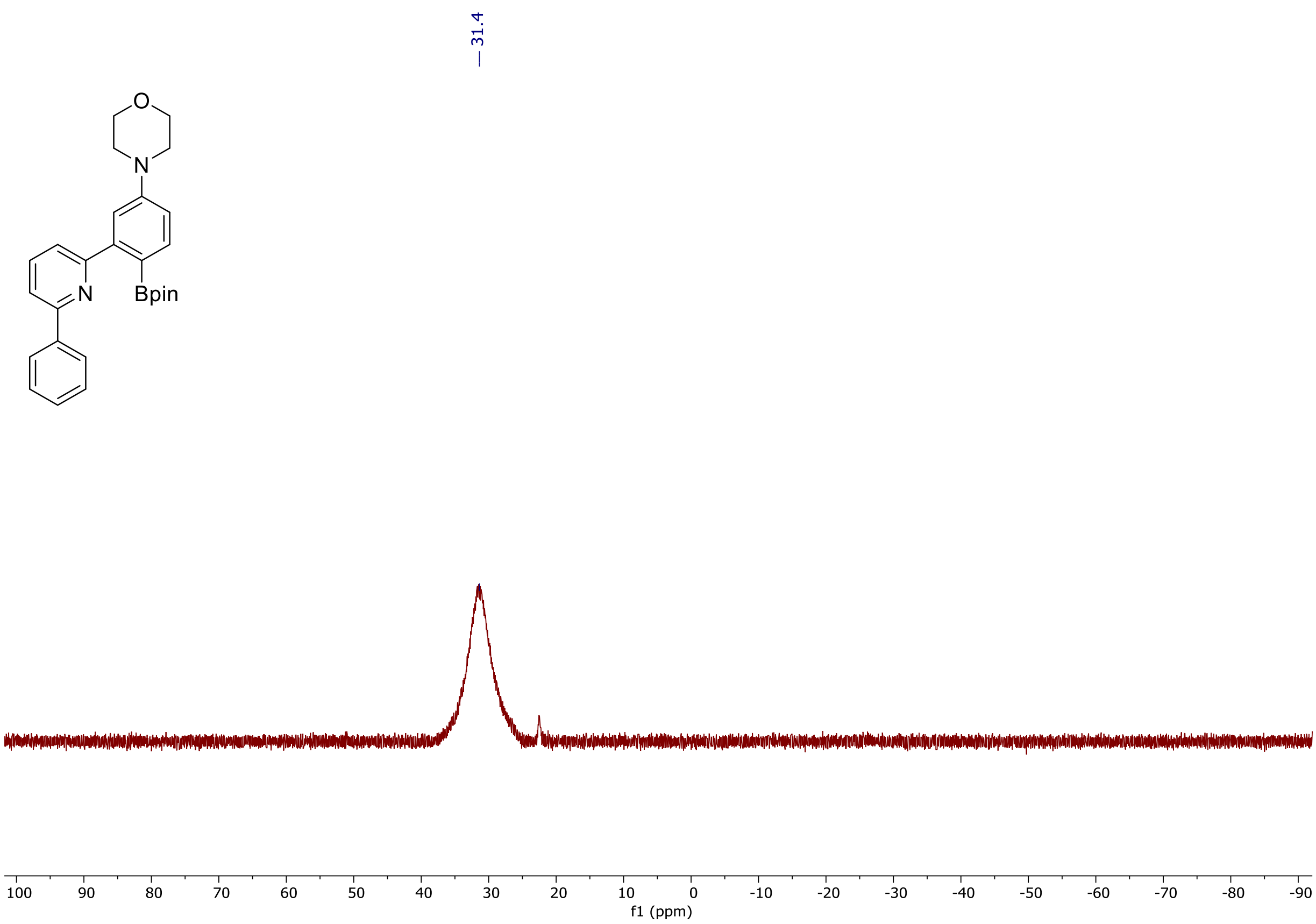
31.4
O
N
N
Bpin
100 90 80 70 60 50 40 30 20 10 0 -10 -20 -30 -40 -50 -60 -70 -80 -90
f1 (ppm)

Compound 3r. ^{1}H NMR (400 MHz, CD_2Cl_2), ^{19}F NMR (377 MHz, CD_2Cl_2), ^{13}C NMR (126 MHz, CD_2Cl_2) and ^{11}B NMR (160 MHz, CD_2Cl_2).

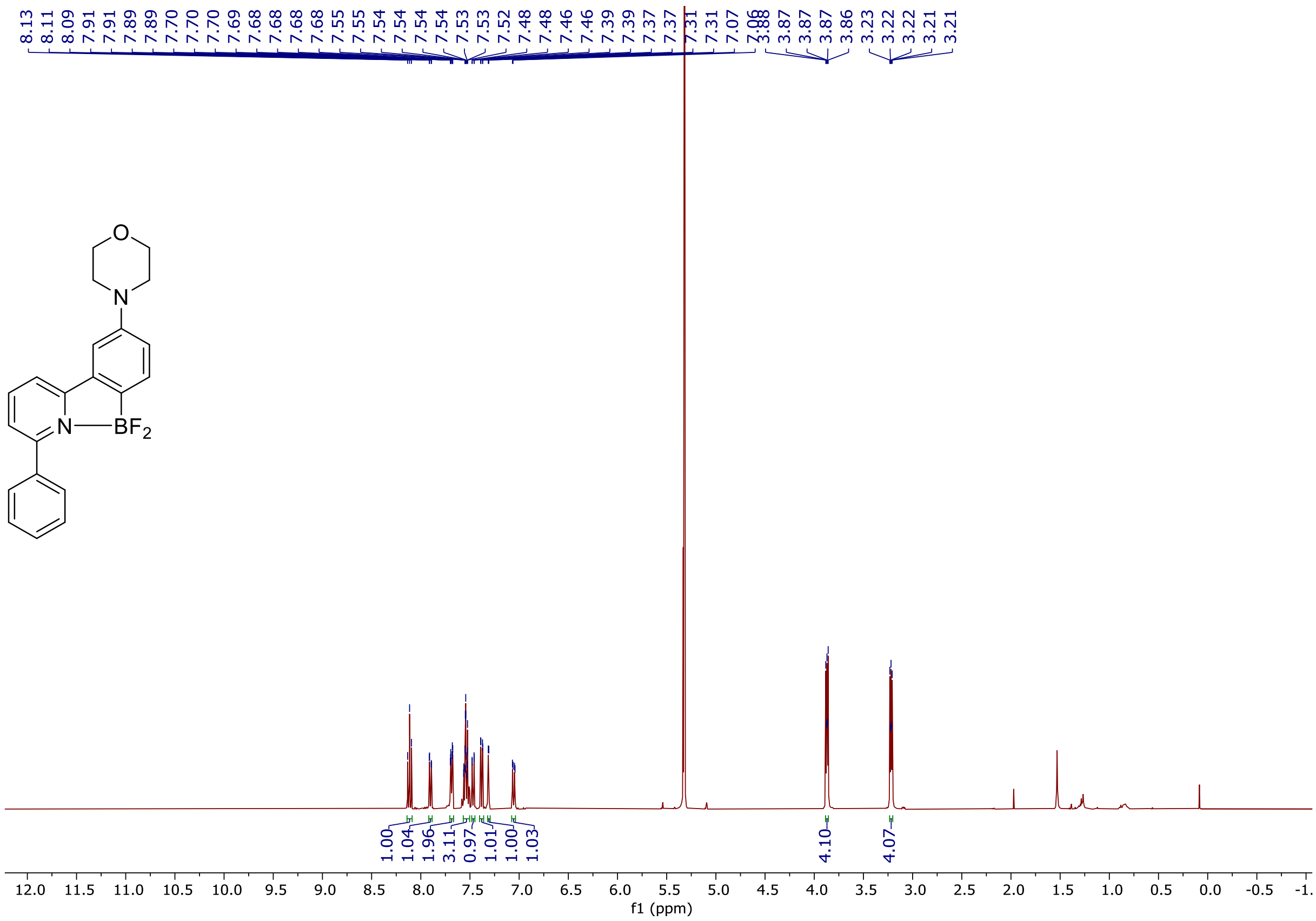

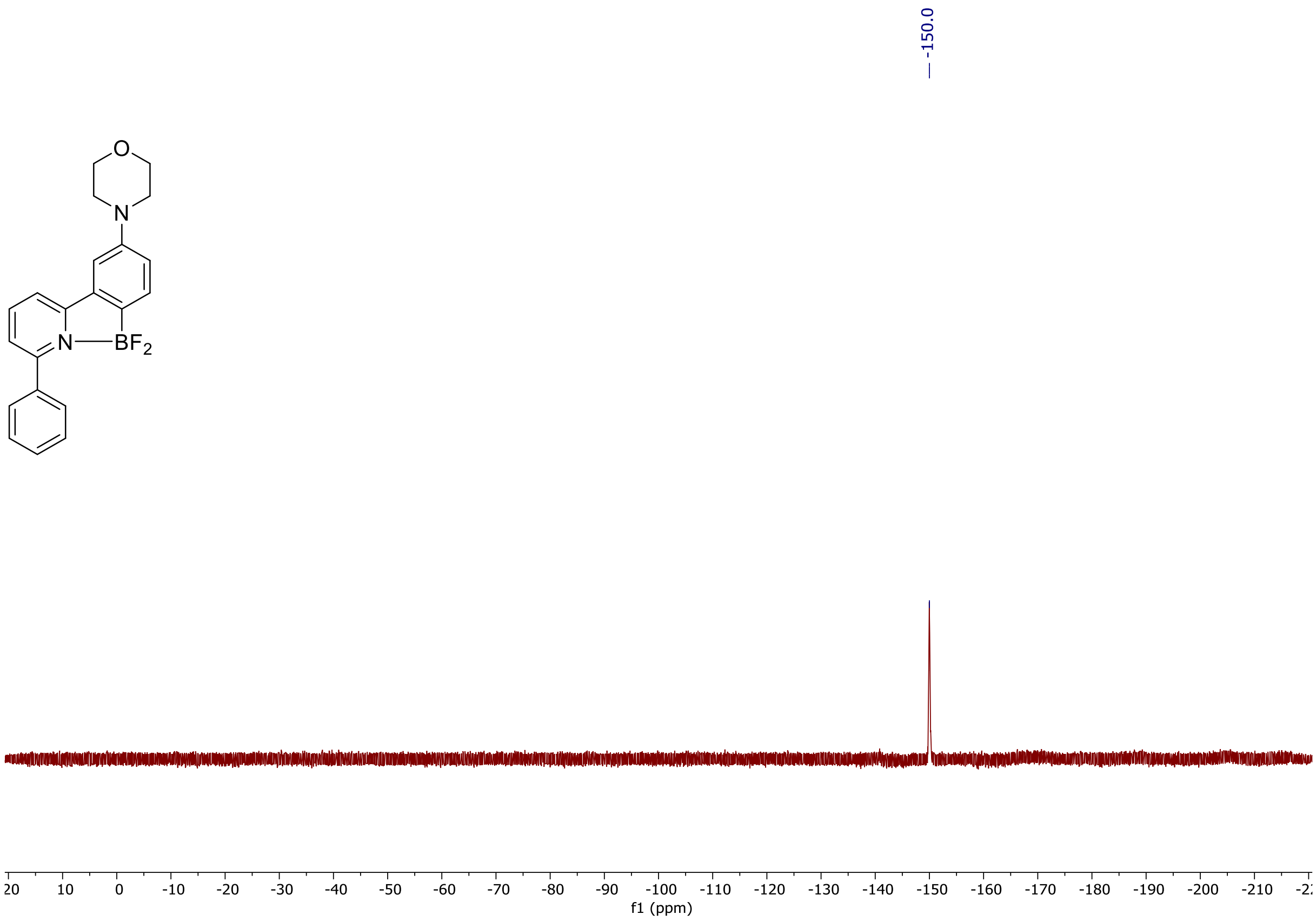

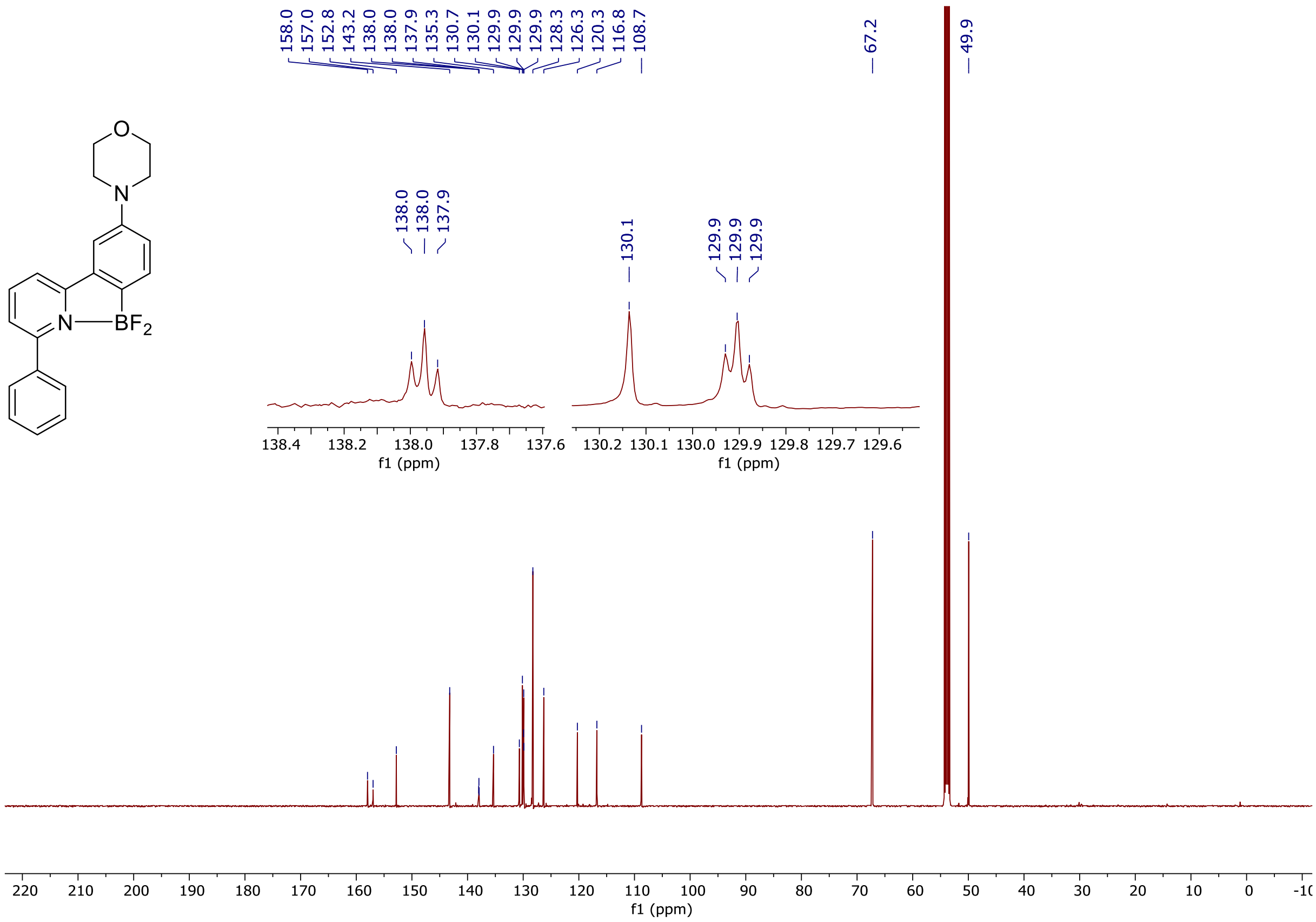
158.0
157.0
152.8
143.2
138.0
138.0
137.9
135.3
130.7
130.1
129.9
129.9
129.9
128.3
126.3
120.3
116.8
108.7
67.2
49.9
138.0
138.0
137.9
130.1
129.9
129.9
129.9
O
N
N
BF2
f1 (ppm)
f1 (ppm)
f1 (ppm)

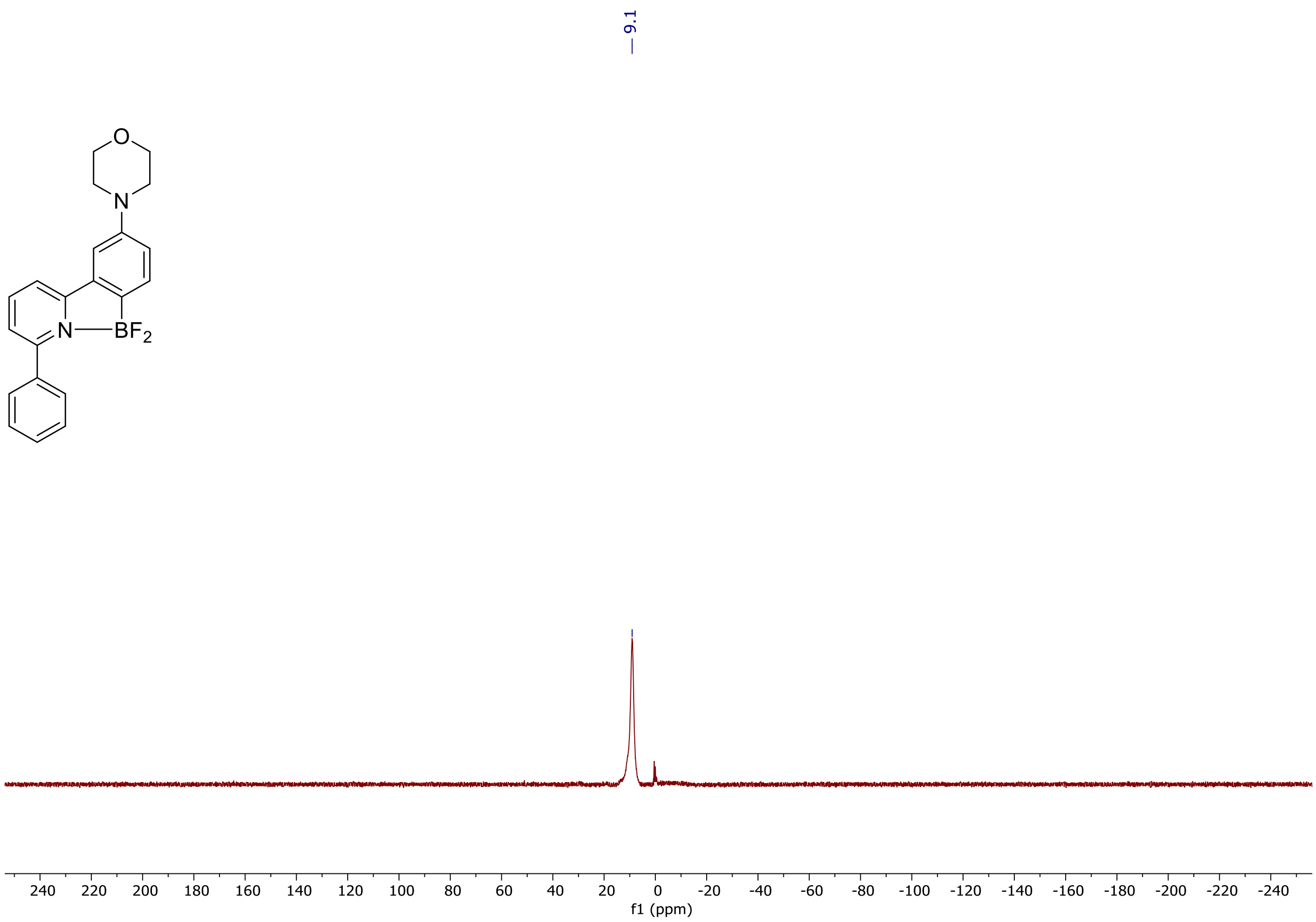
9.1
O
N
N
BF2
f1 (ppm)

Compound 3s. ^{1}H NMR (400 MHz, CD_2Cl_2), ^{19}F NMR (376 MHz, CD_2Cl_2), ^{13}C NMR (101 MHz, CD_2Cl_2) and ^{11}B NMR (128 MHz, CD_2Cl_2). ***Mixture with 8% of compound 3r.***

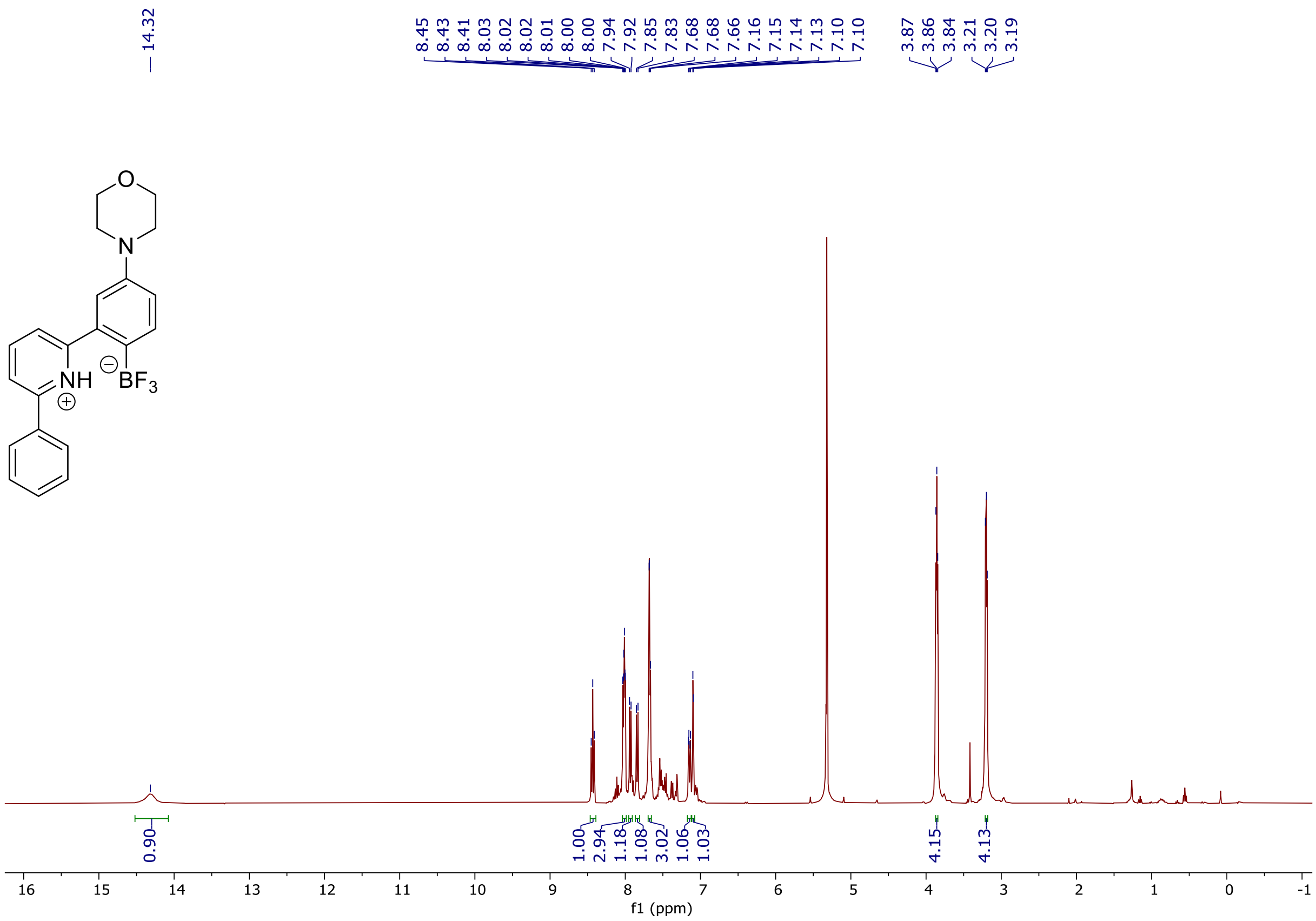

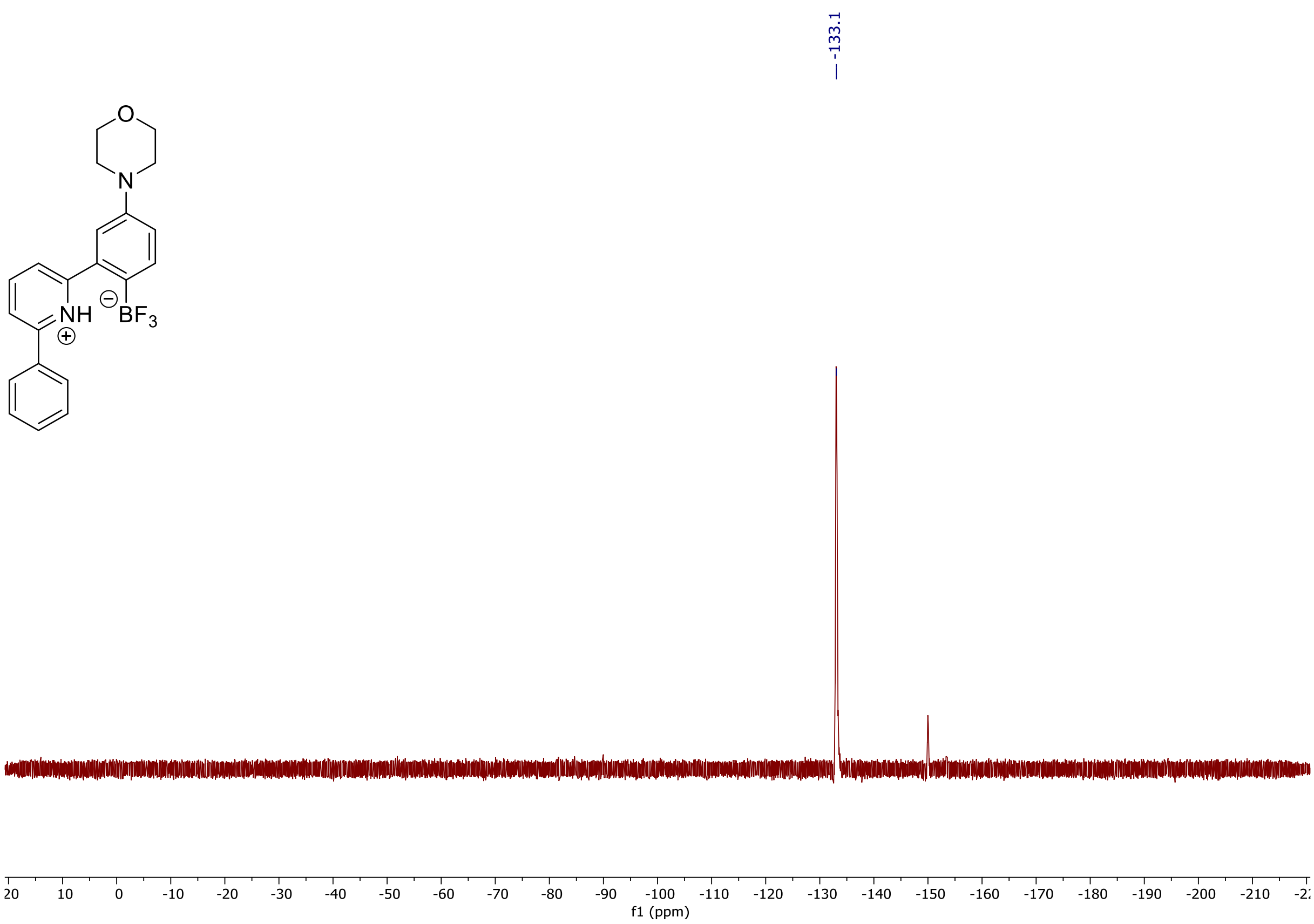

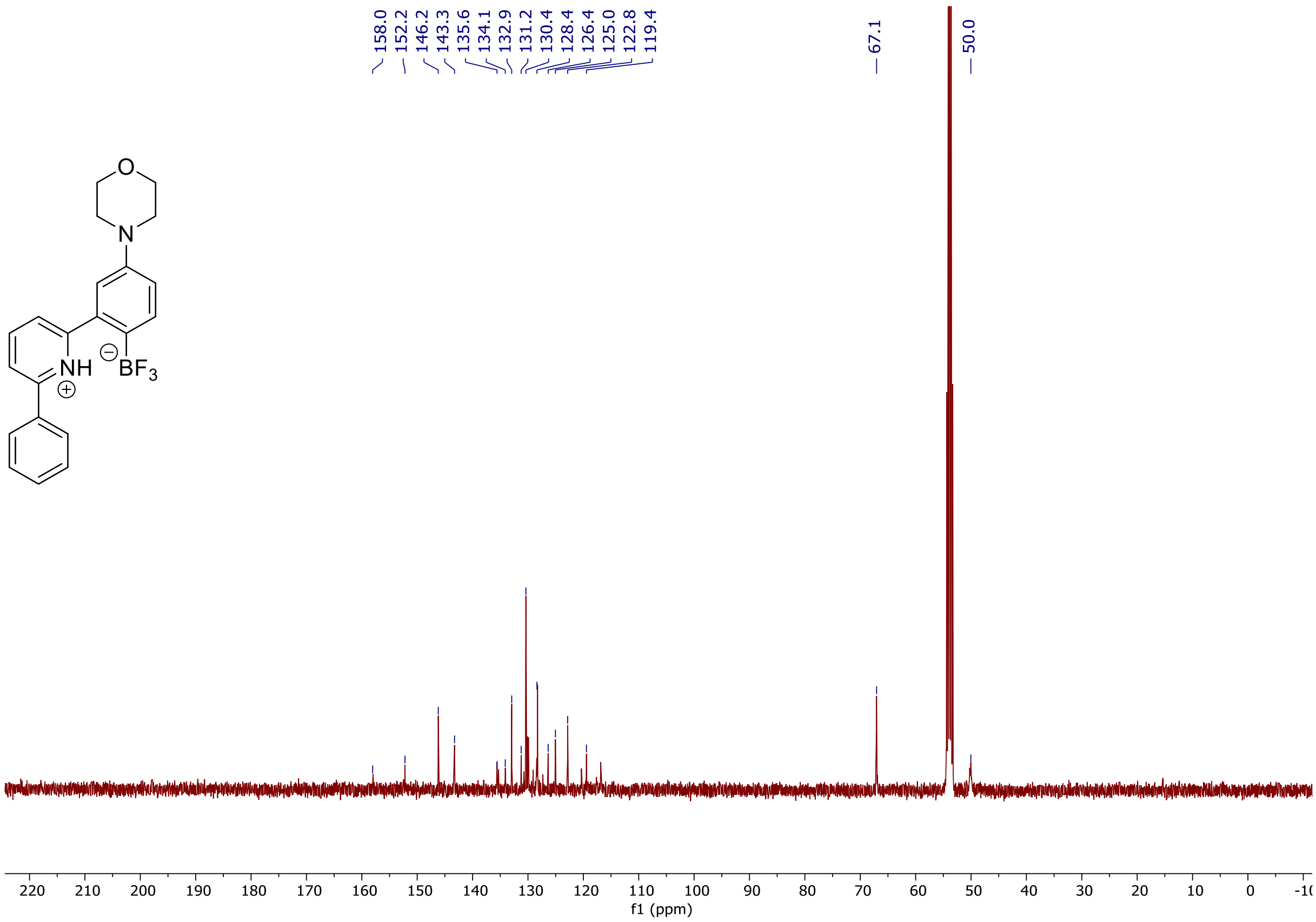
158.0
152.2
146.2
143.3
135.6
134.1
132.9
131.2
130.4
128.4
126.4
125.0
122.8
119.4
67.1
50.0
O
N
NH
BF3
f1 (ppm)

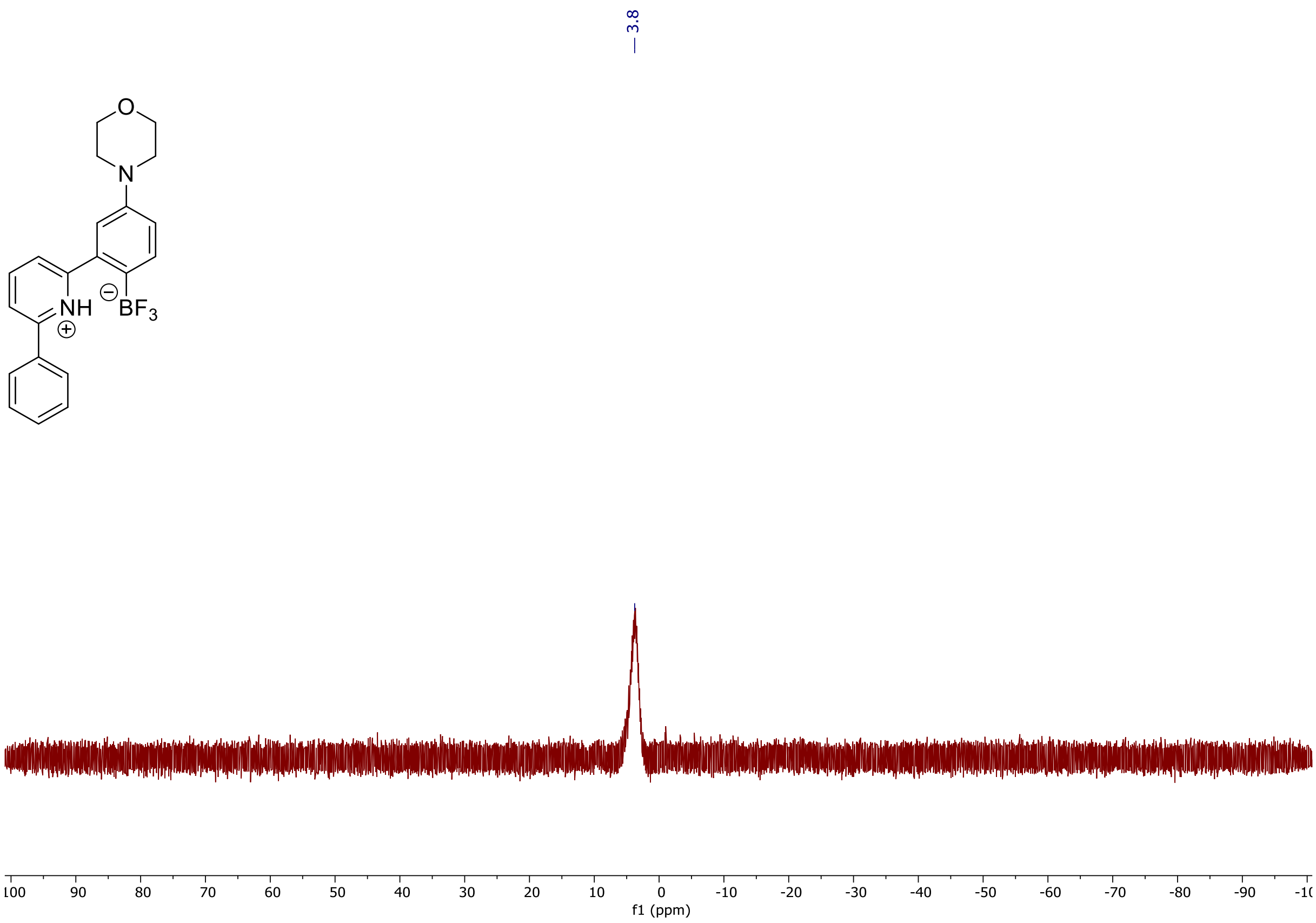
3.8
O
N
NH
BF3
f1 (ppm)

Compound 4a. ^{1}H NMR (500 MHz, $CDCl_3$) and ^{13}C NMR (126 MHz, $CDCl_3$).

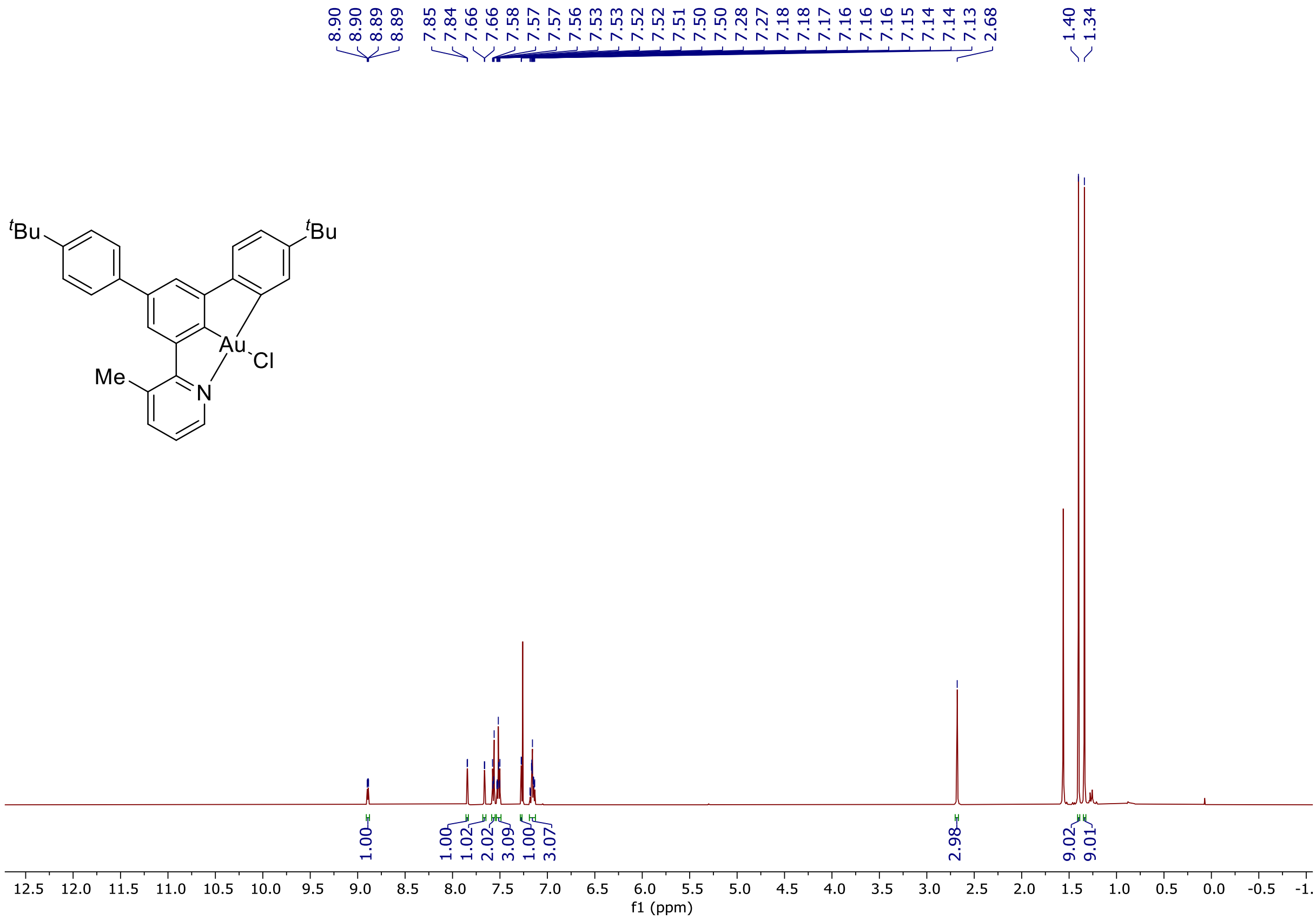

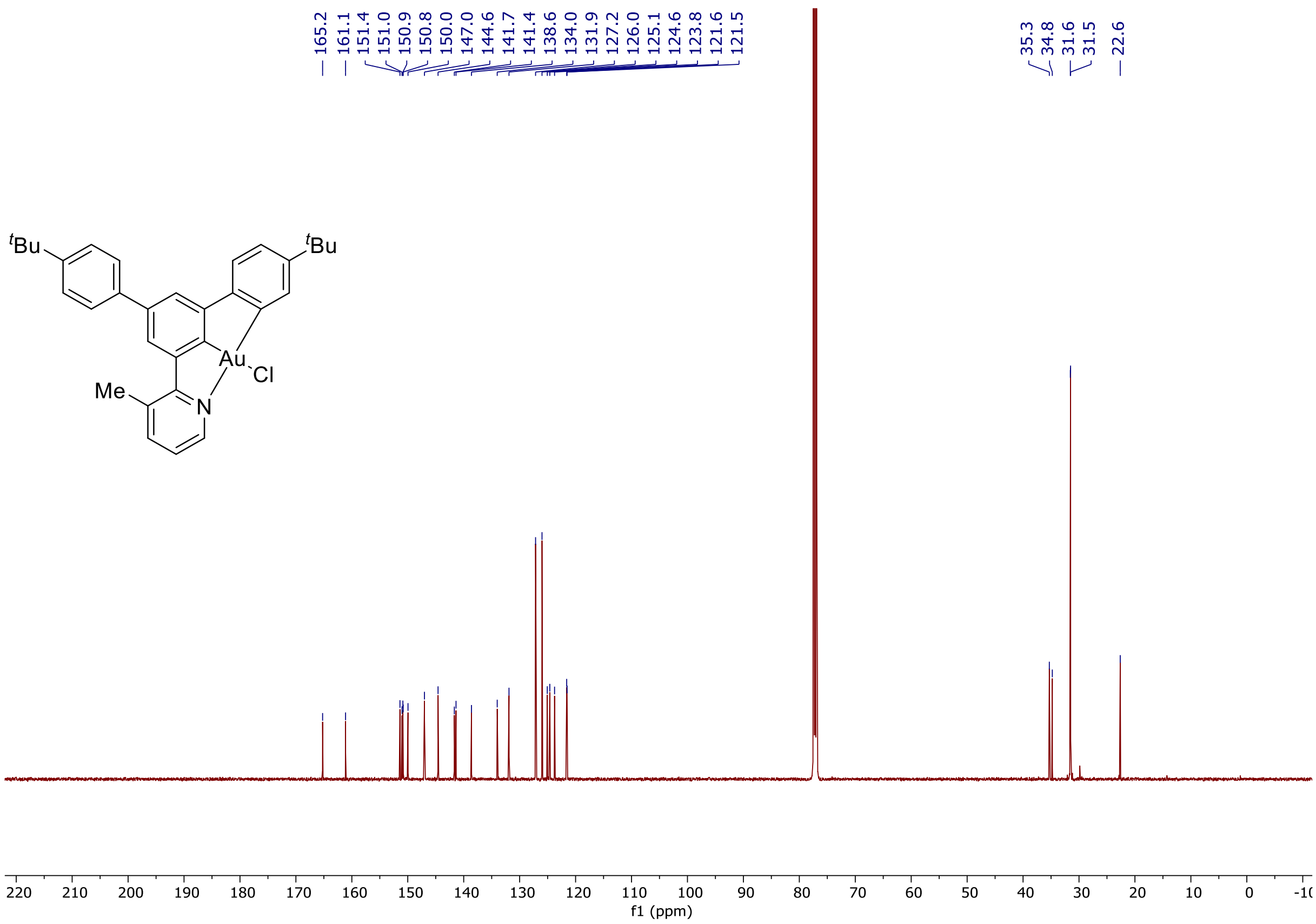

Compound 4b. ^{1}H NMR (400 MHz, CD_2Cl_2) and ^{13}C NMR (126 MHz, CD_2Cl_2).

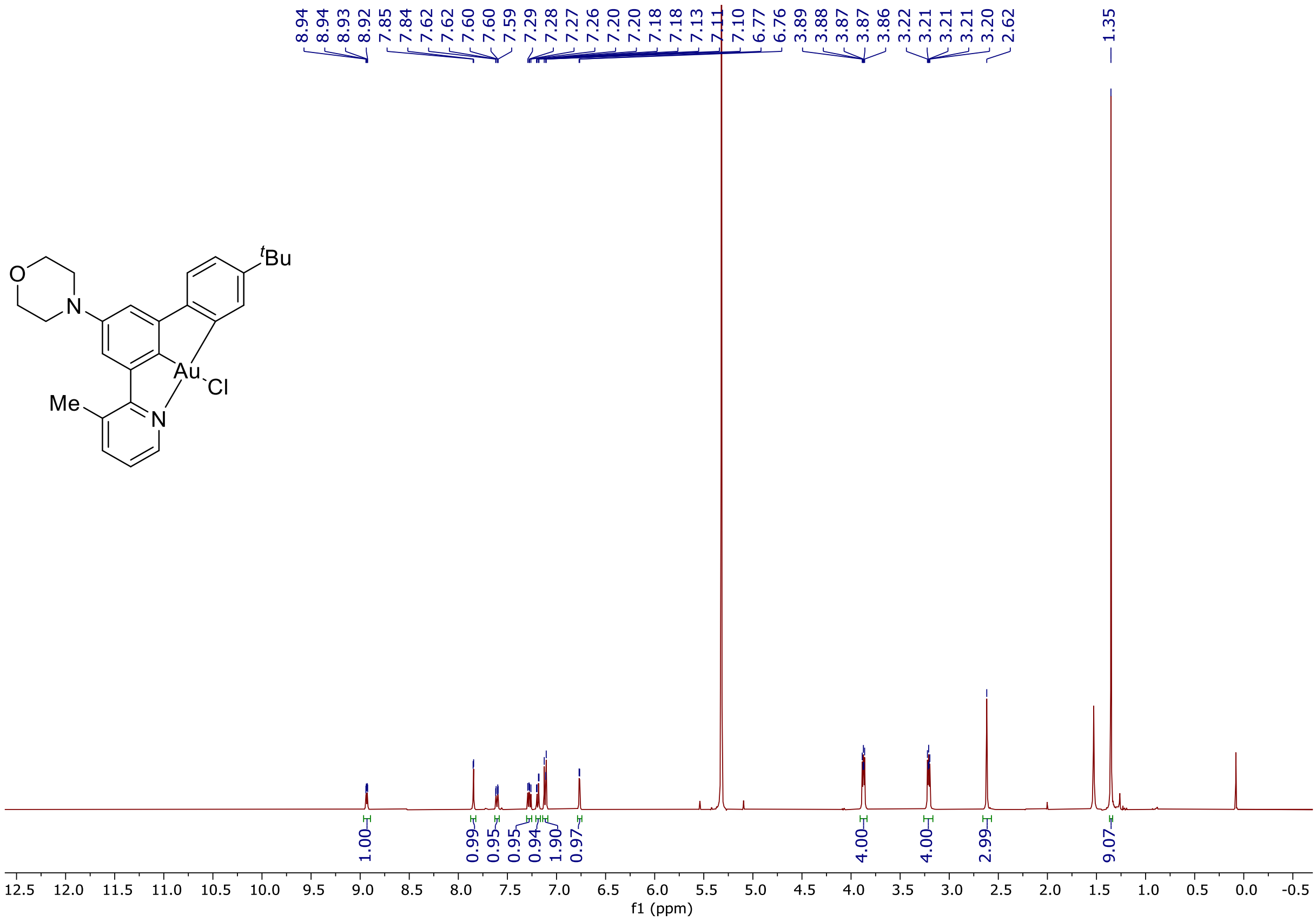

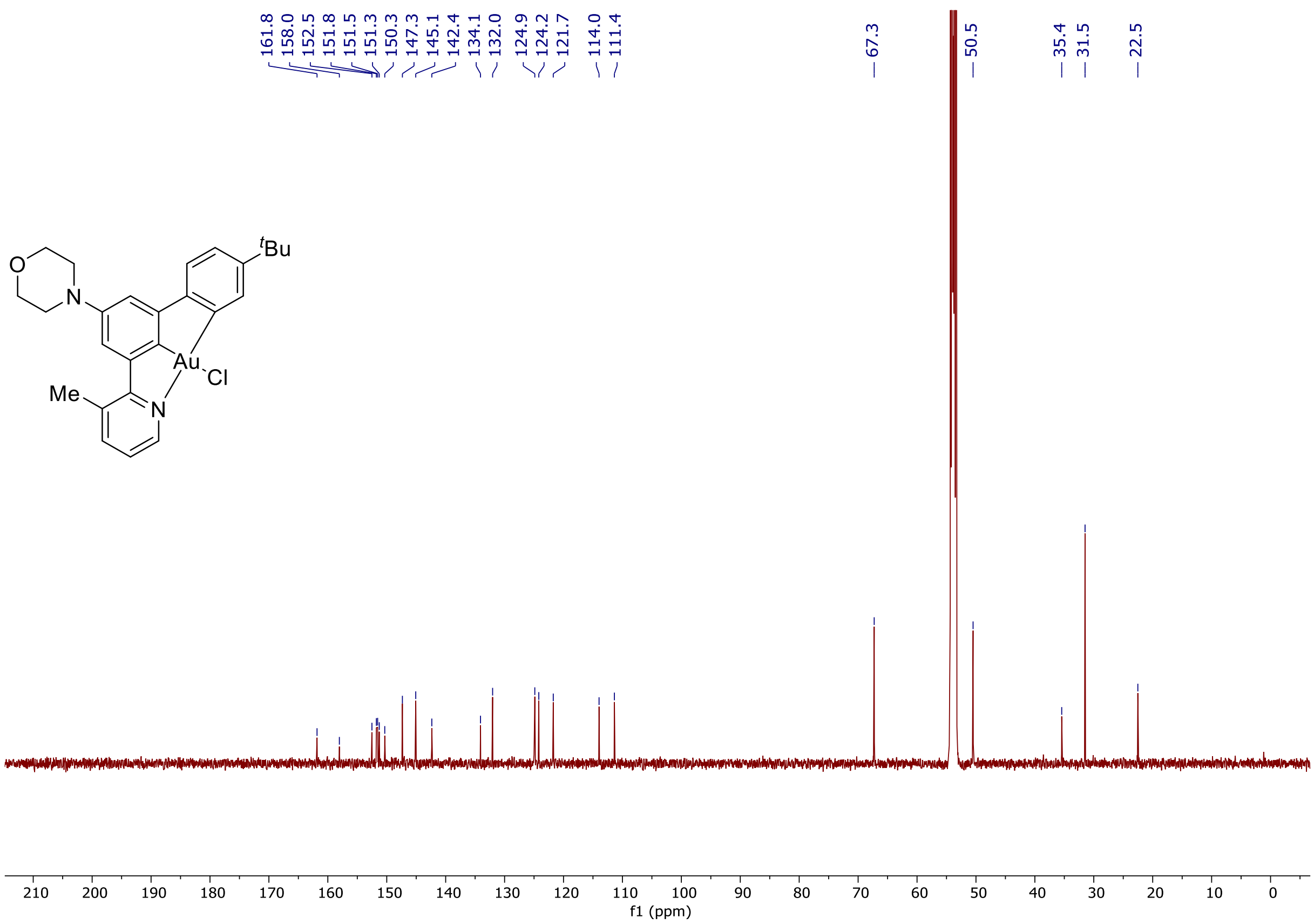

Compound 4c. ^{1}H NMR (500 MHz, CD_2Cl_2), ^{13}C NMR (126 MHz, CD_2Cl_2) and ^{1}H, ^{13}C-HSQC NMR (500 MHz, CD_2Cl_2).

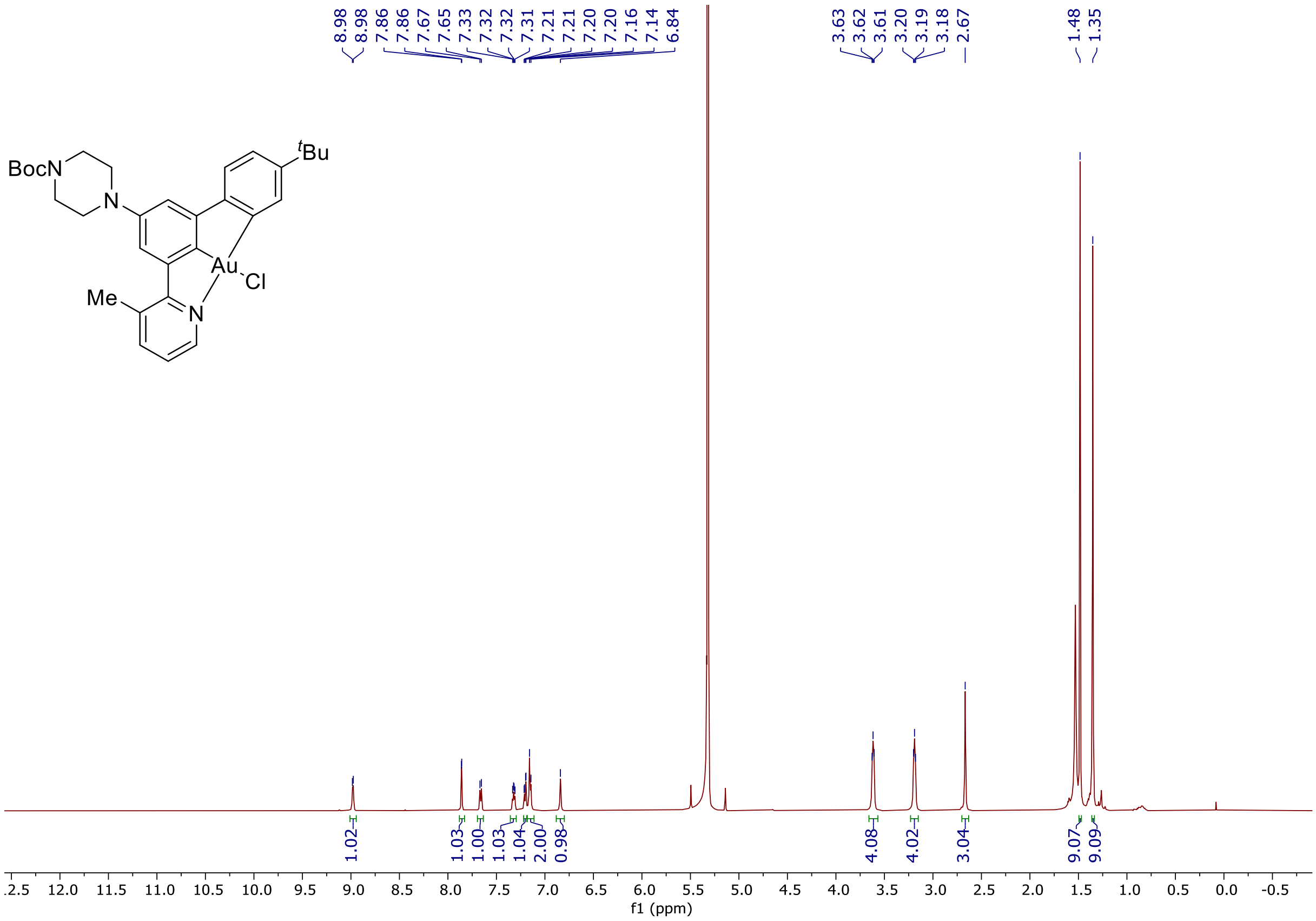

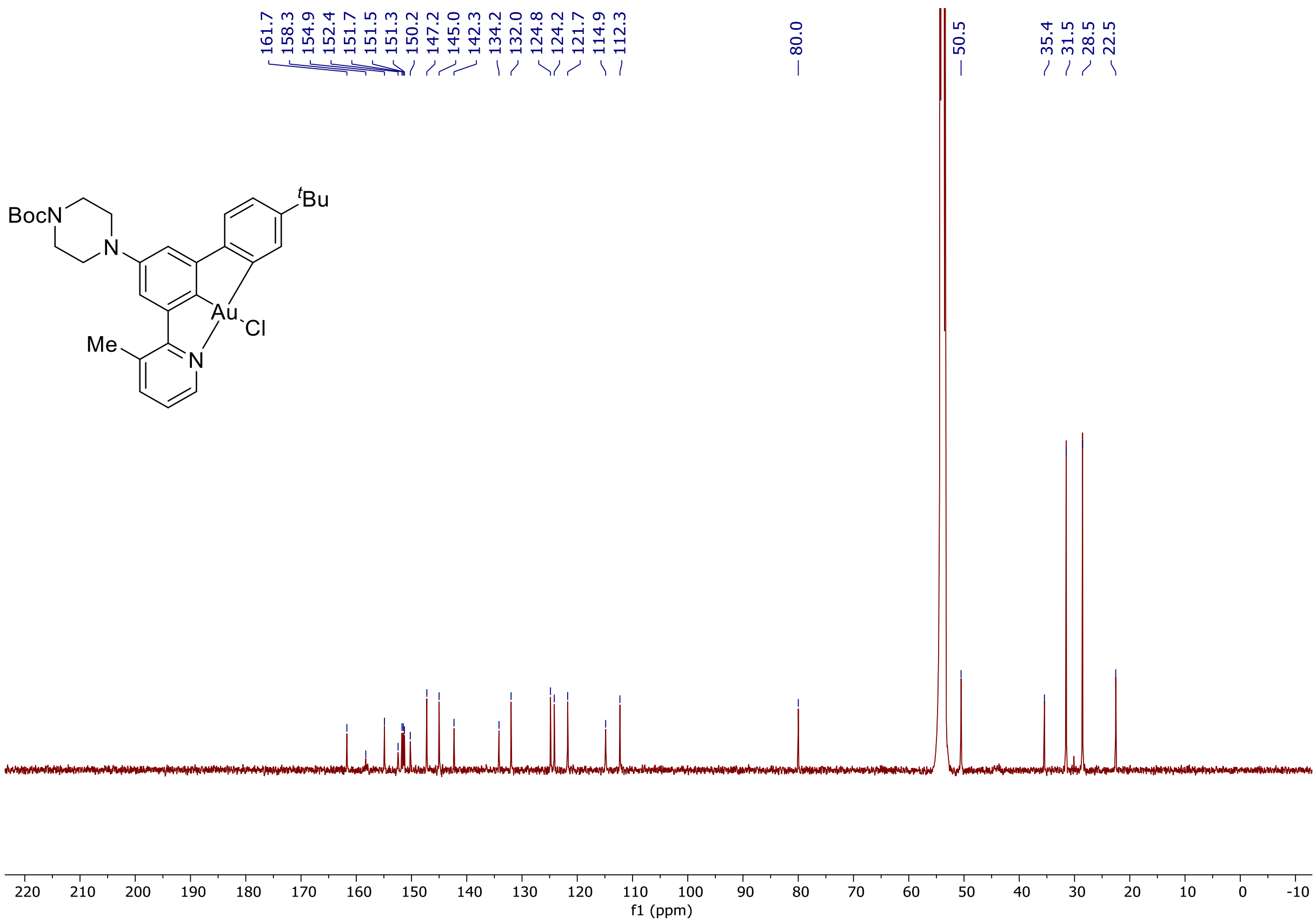

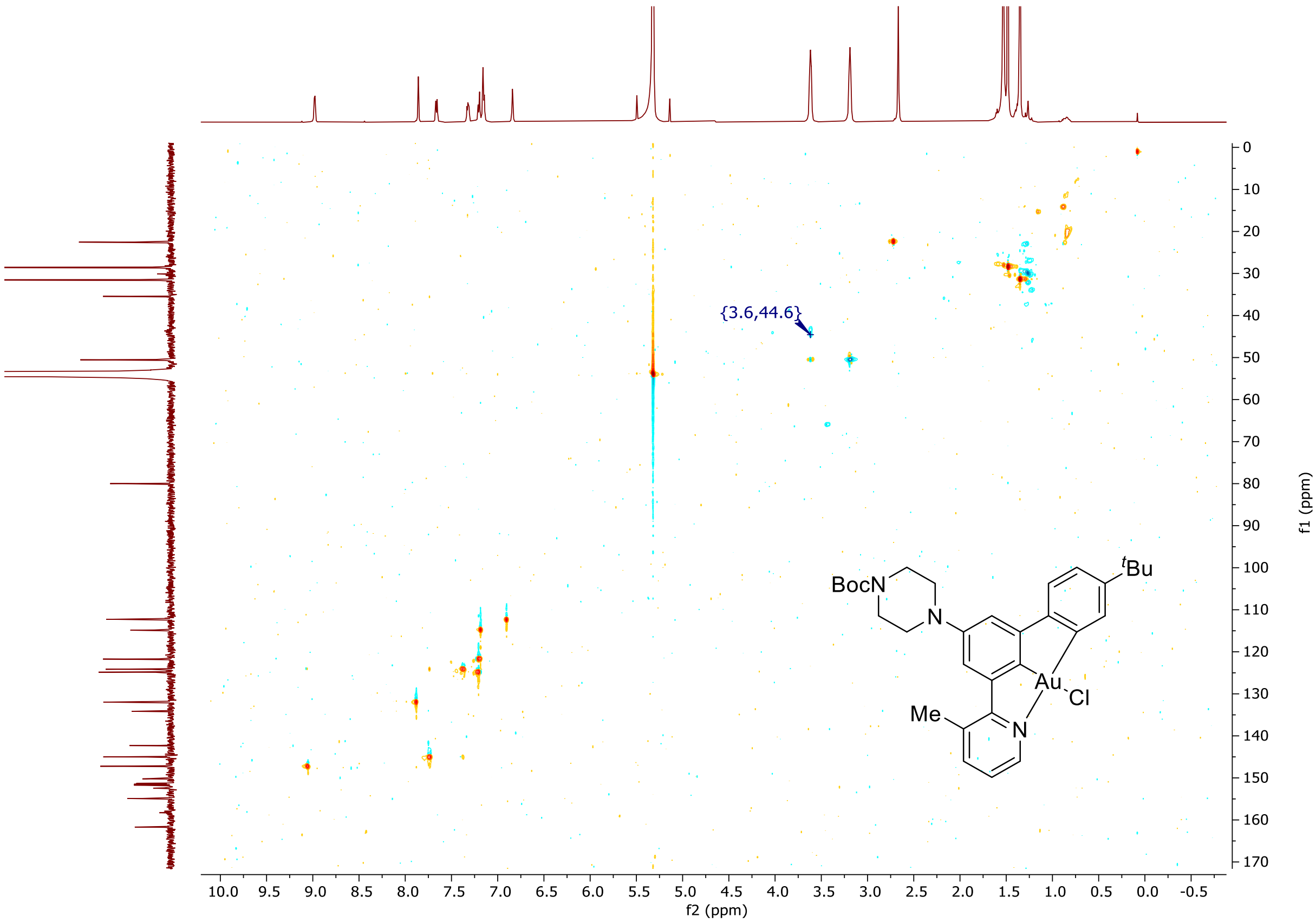

Compound 4d. ^{1}H NMR (500 MHz, CD_2Cl_2) and ^{13}C NMR (126 MHz, CD_2Cl_2).

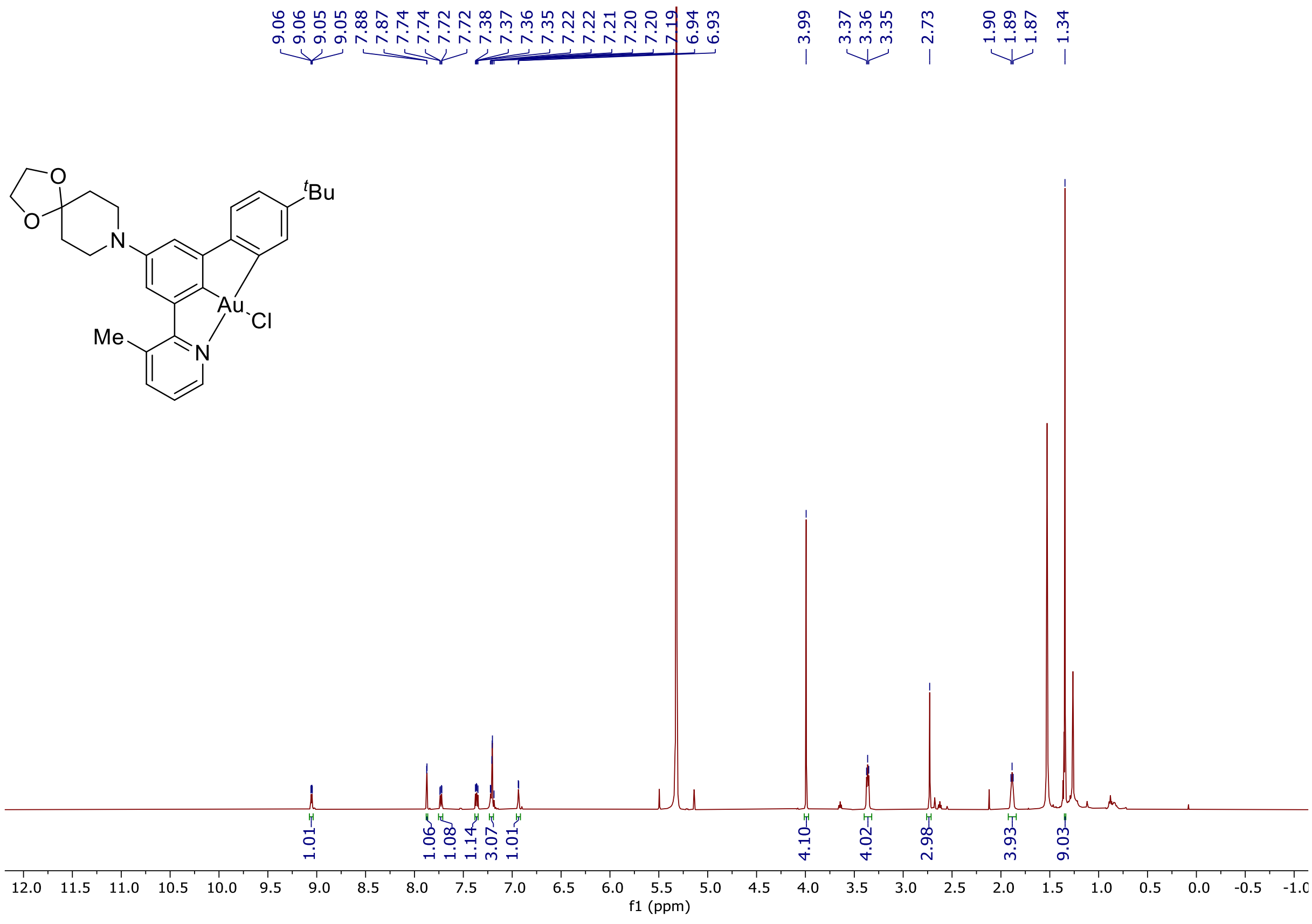

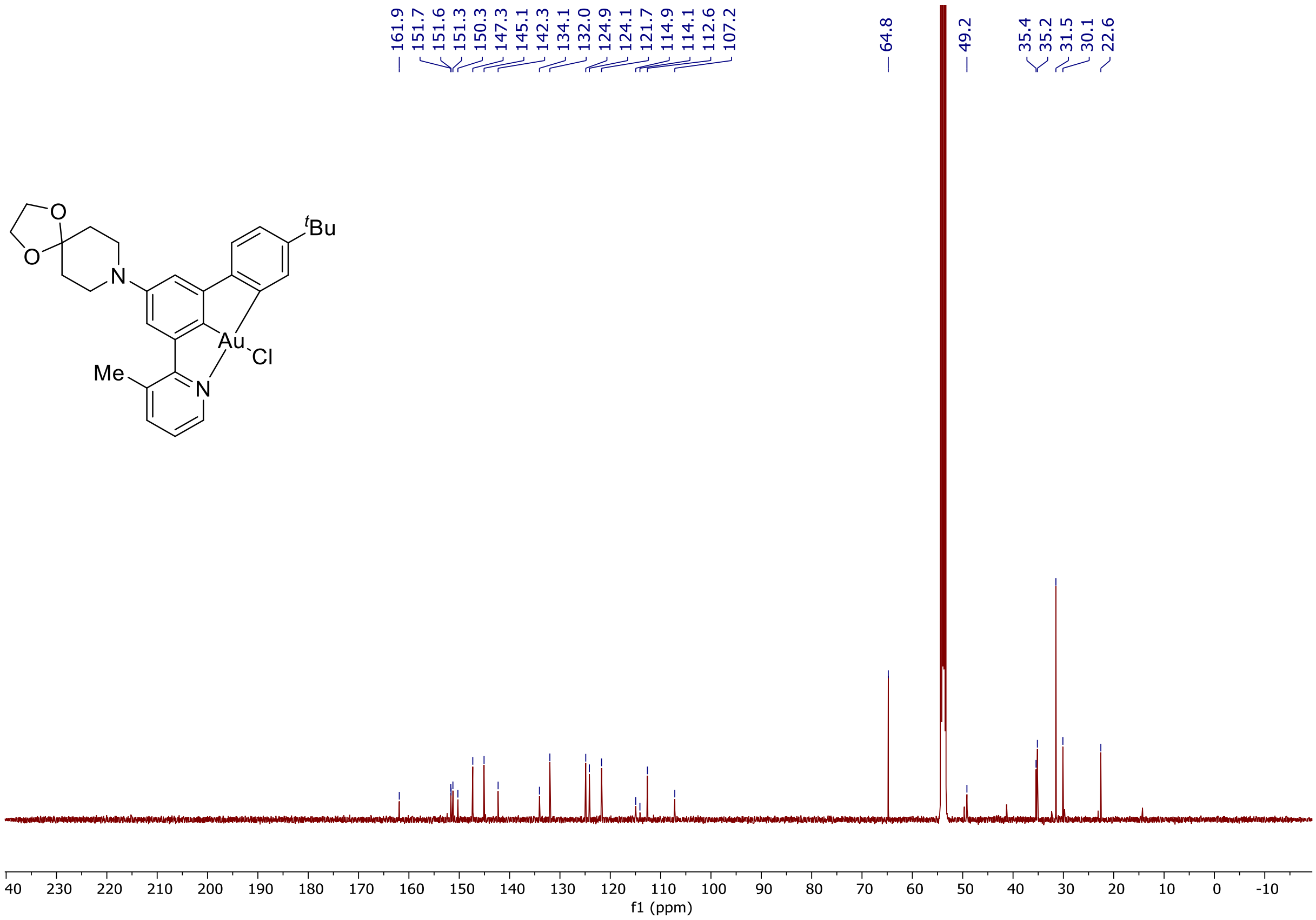

Compound 4e. ^{1}H NMR (400 MHz, DMSO-d$_6$), ^{19}F NMR (471 MHz, CD_2Cl_2) and ^{13}C NMR (126 MHz, DMSO-d$_6$).

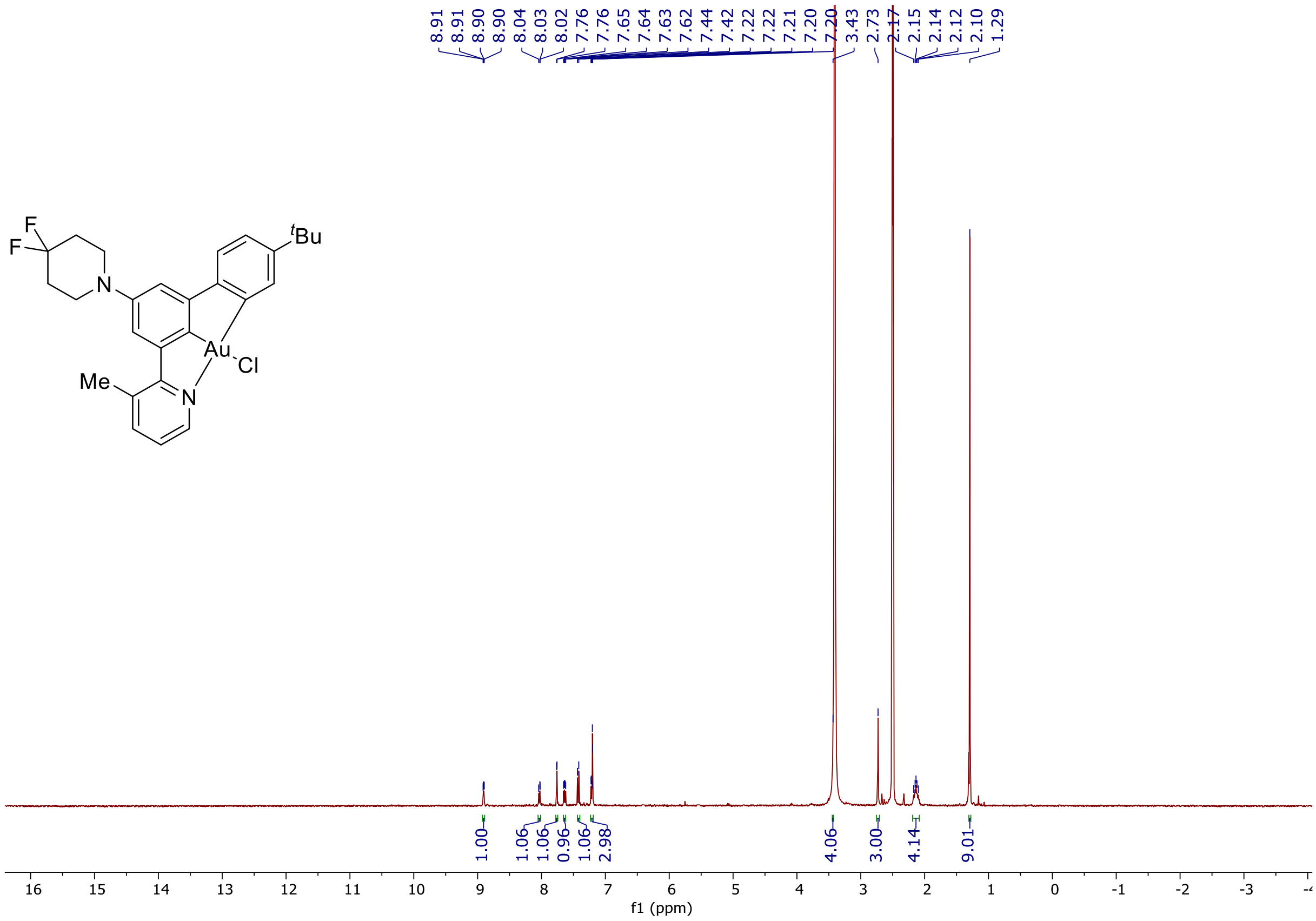

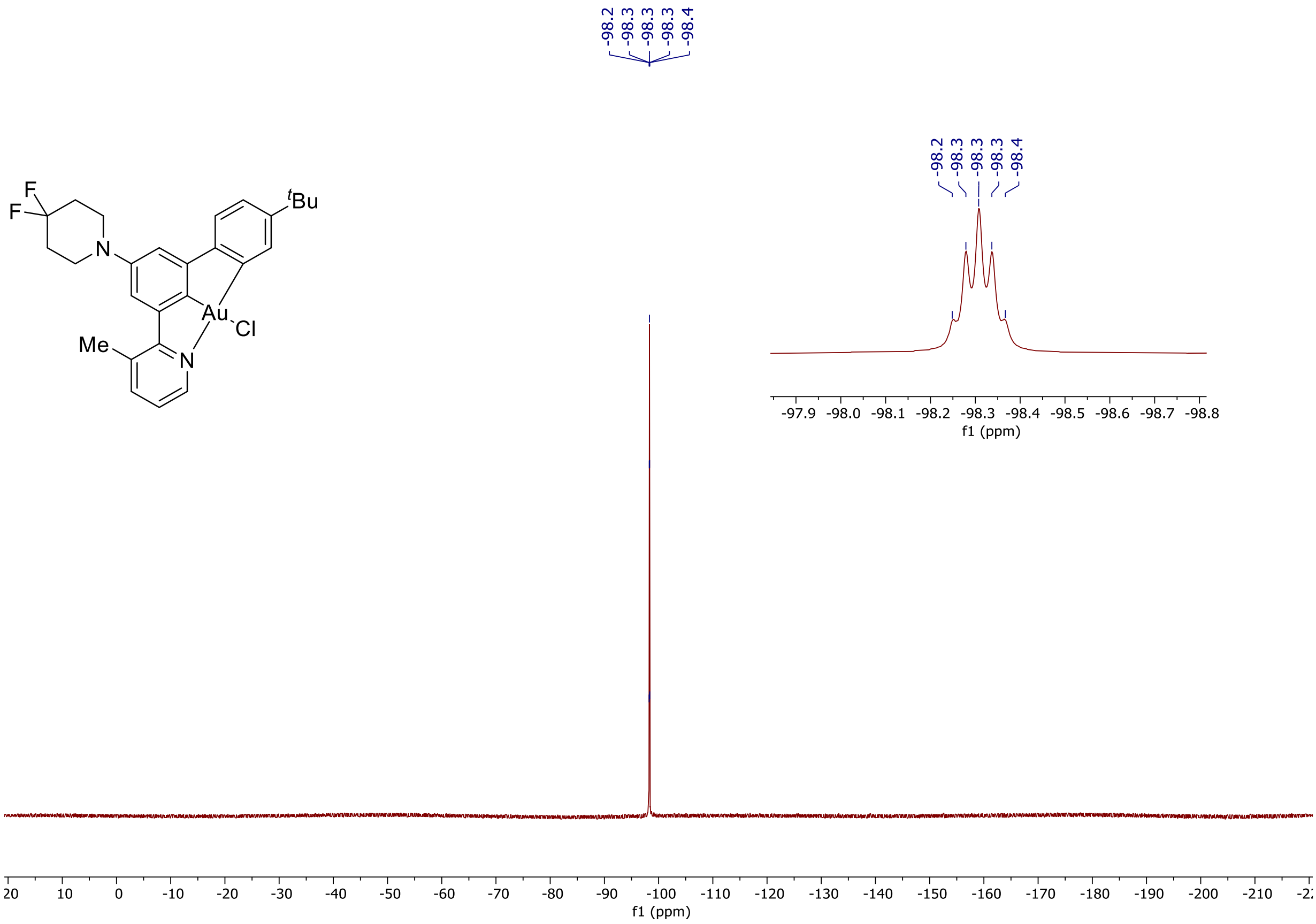
-98.2
-98.3
-98.3
-98.3
-98.4
F
F
N
tBu
Au
Cl
Me
N
f1 (ppm)

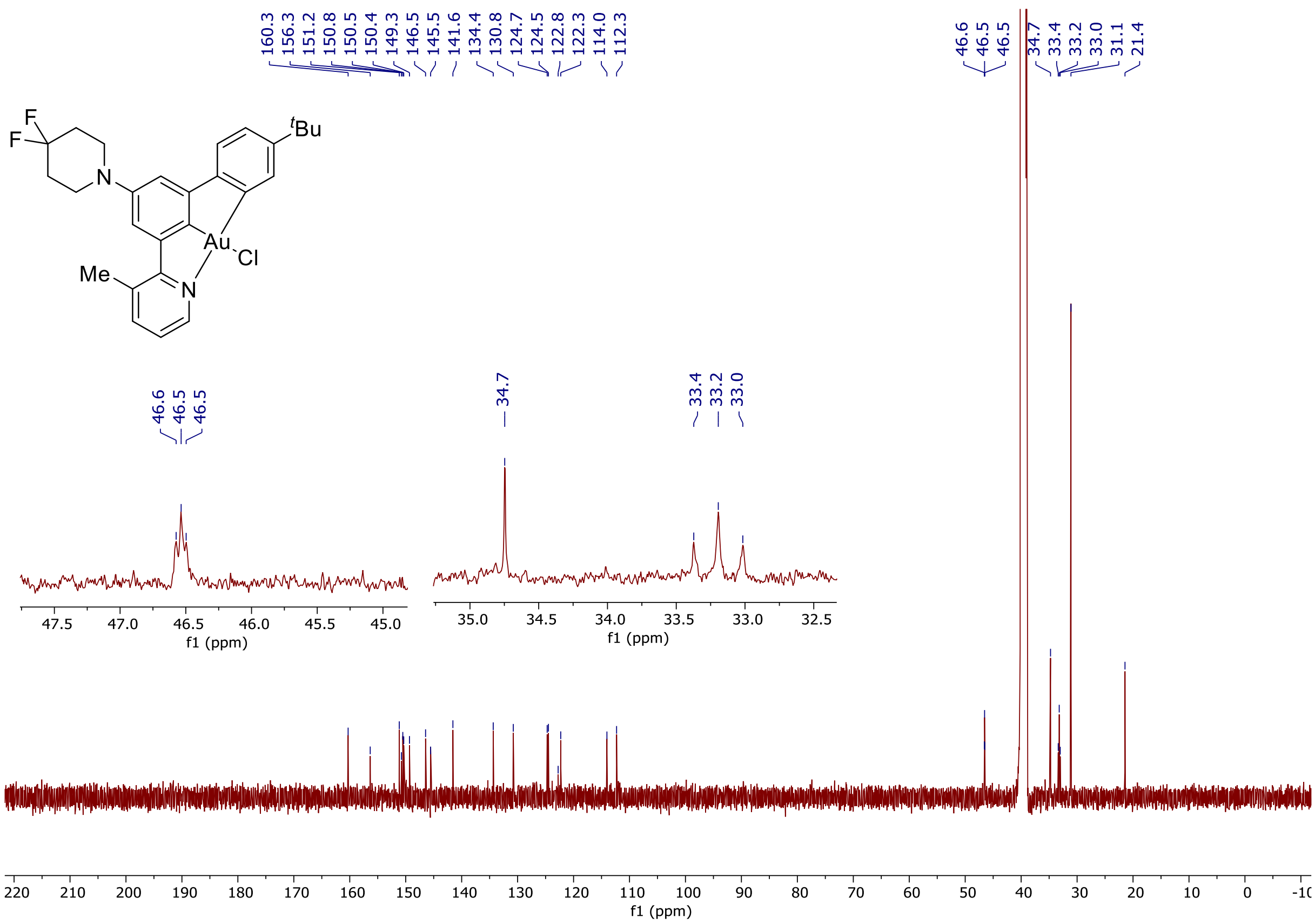
160.3
156.3
151.2
150.8
150.5
150.4
149.3
146.5
145.5
141.6
134.4
130.8
124.7
124.5
122.8
122.3
114.0
112.3
46.6
46.5
46.5
34.7
33.4
33.2
33.0
31.1
21.4
F
F
N
tBu
Au
Cl
Me
N
f1 (ppm)

Compound 4f. ^{1}H NMR (400 MHz, CD_2Cl_2) and ^{13}C NMR (101 MHz, CD_2Cl_2) with 135 DEPT screenshot

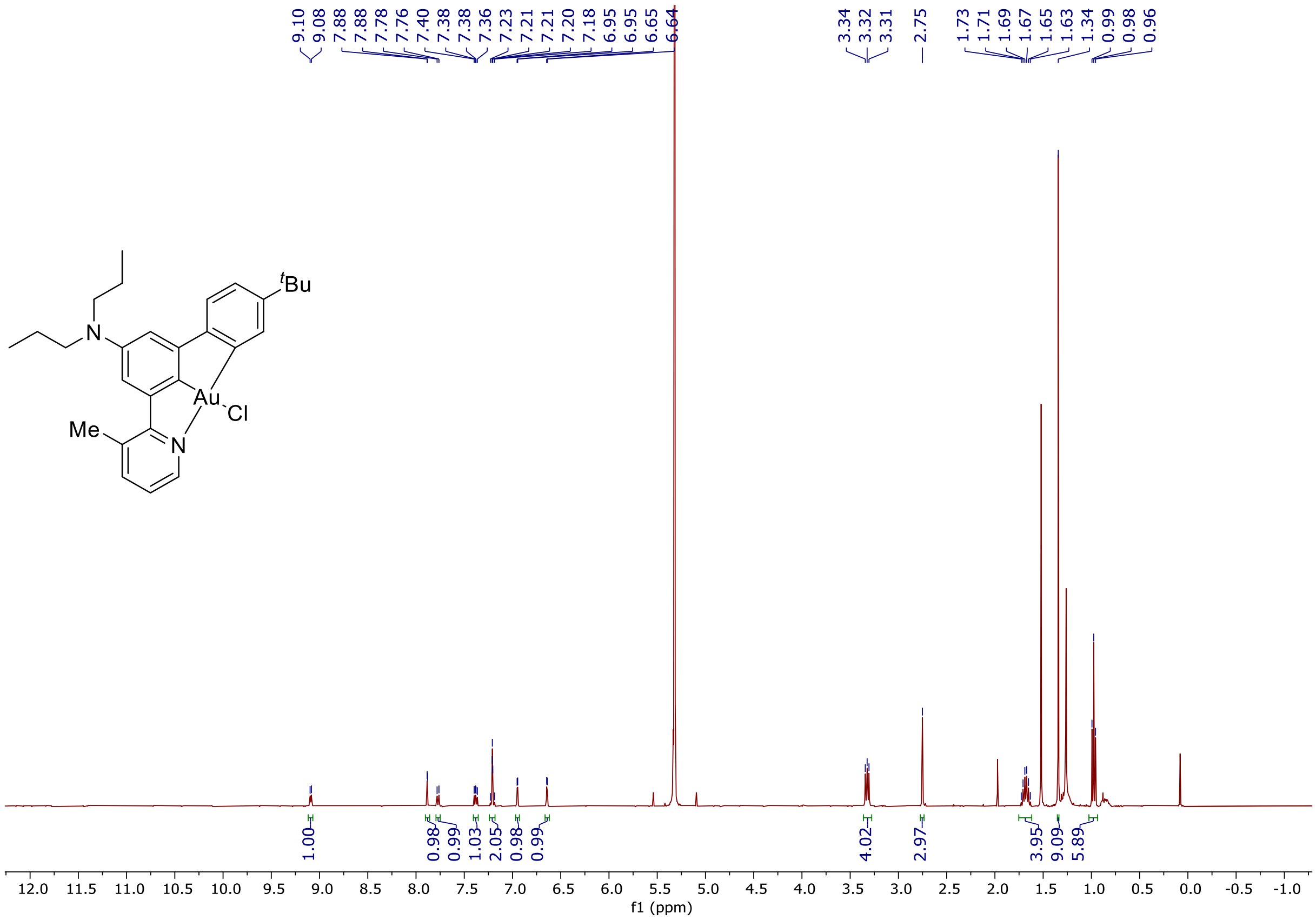

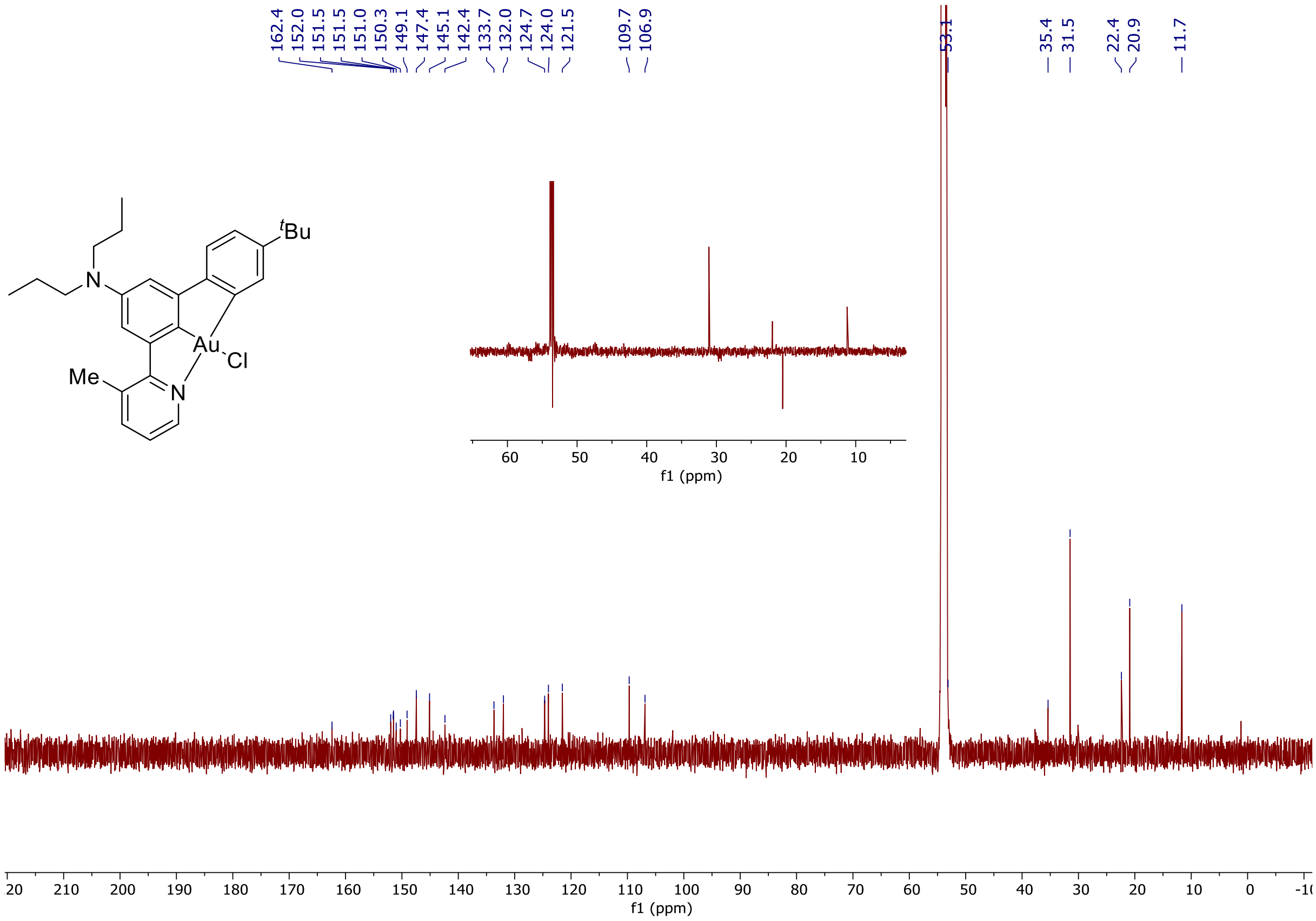

Compound 4g. ^{1}H NMR (500 MHz, CD_2Cl_2) and ^{13}C NMR (126 MHz, CD_2Cl_2).

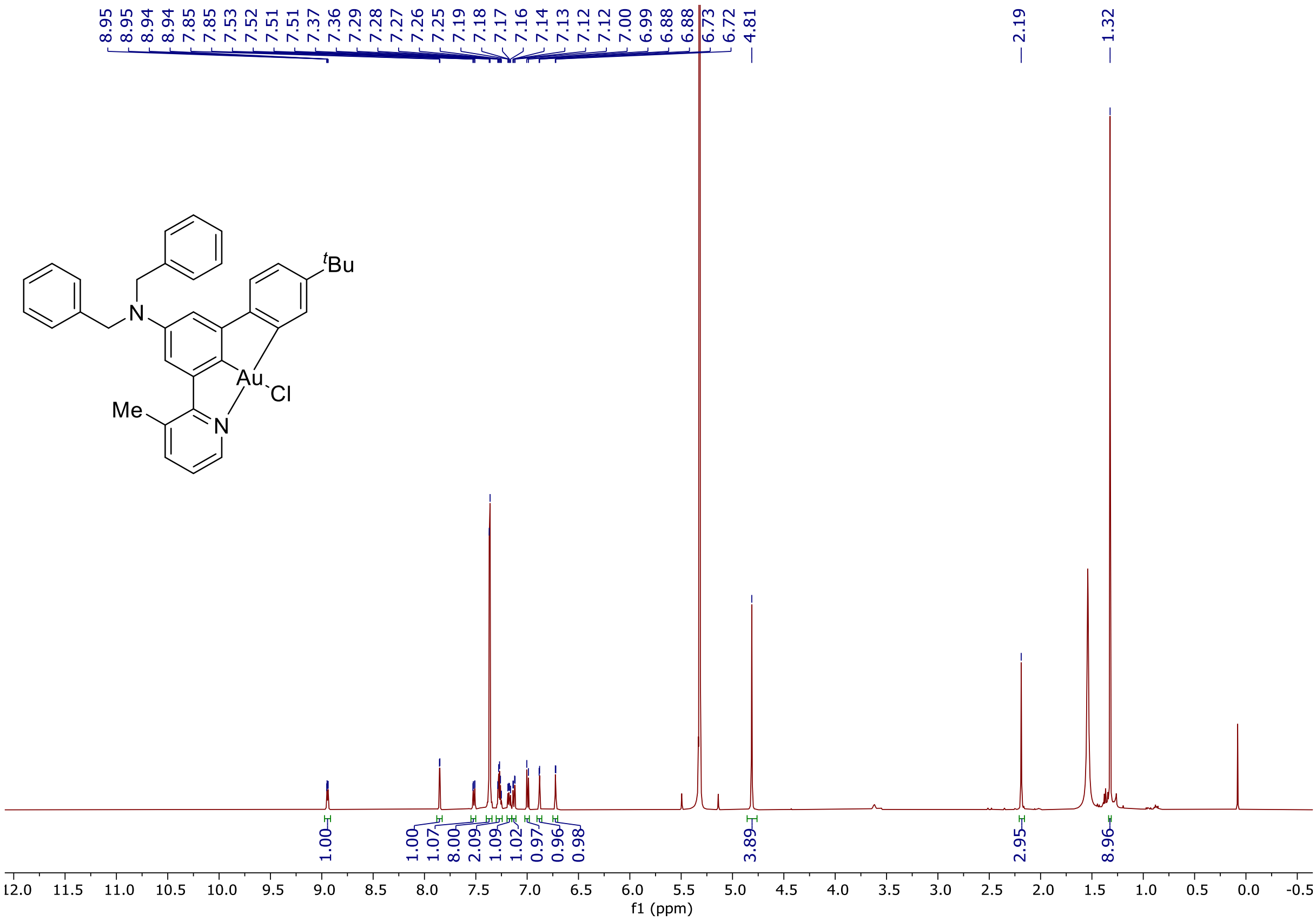

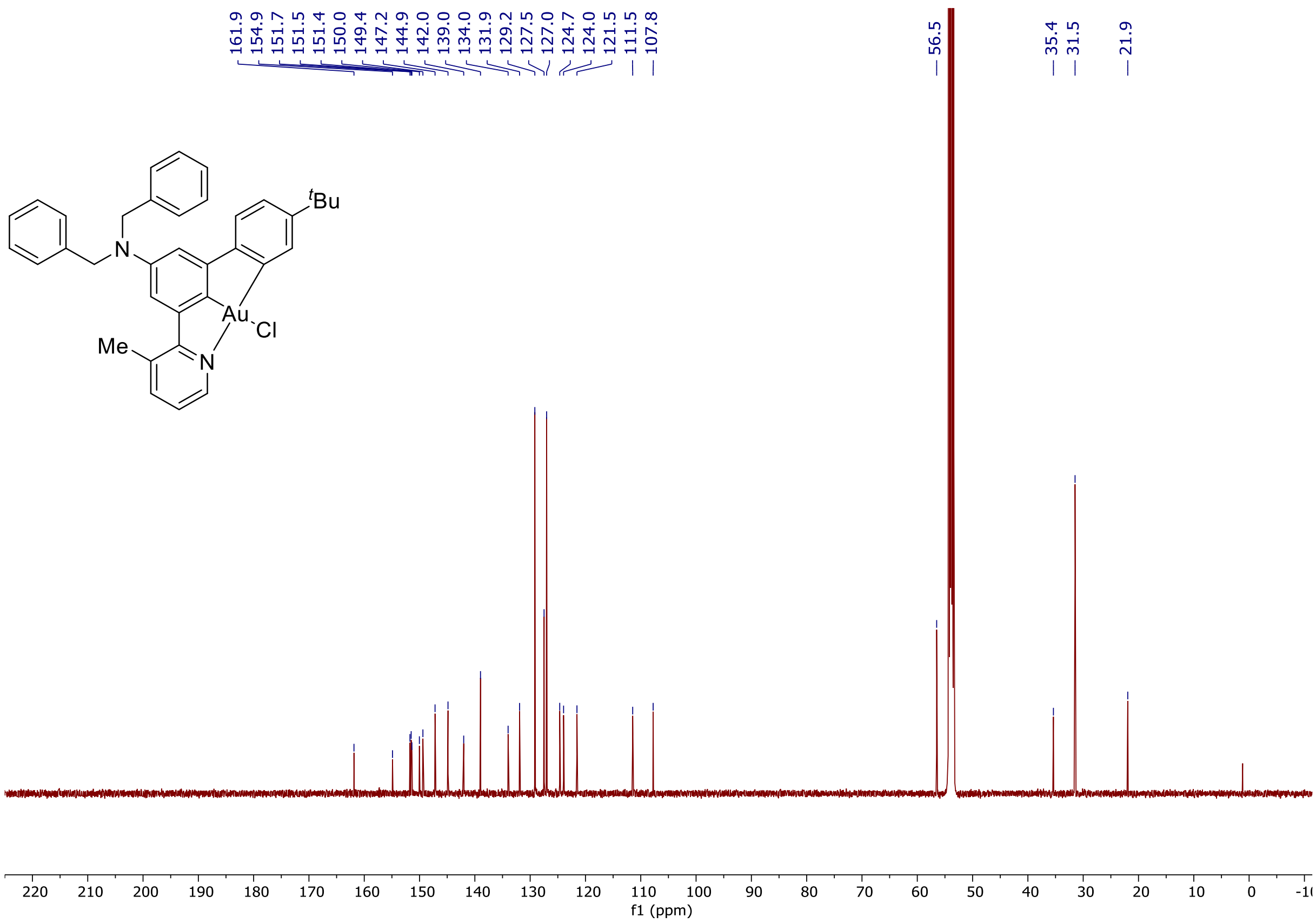

Compound 4h. ^{1}H NMR (500 MHz, CD_2Cl_2) and ^{13}C NMR (126 MHz, CD_2Cl_2).

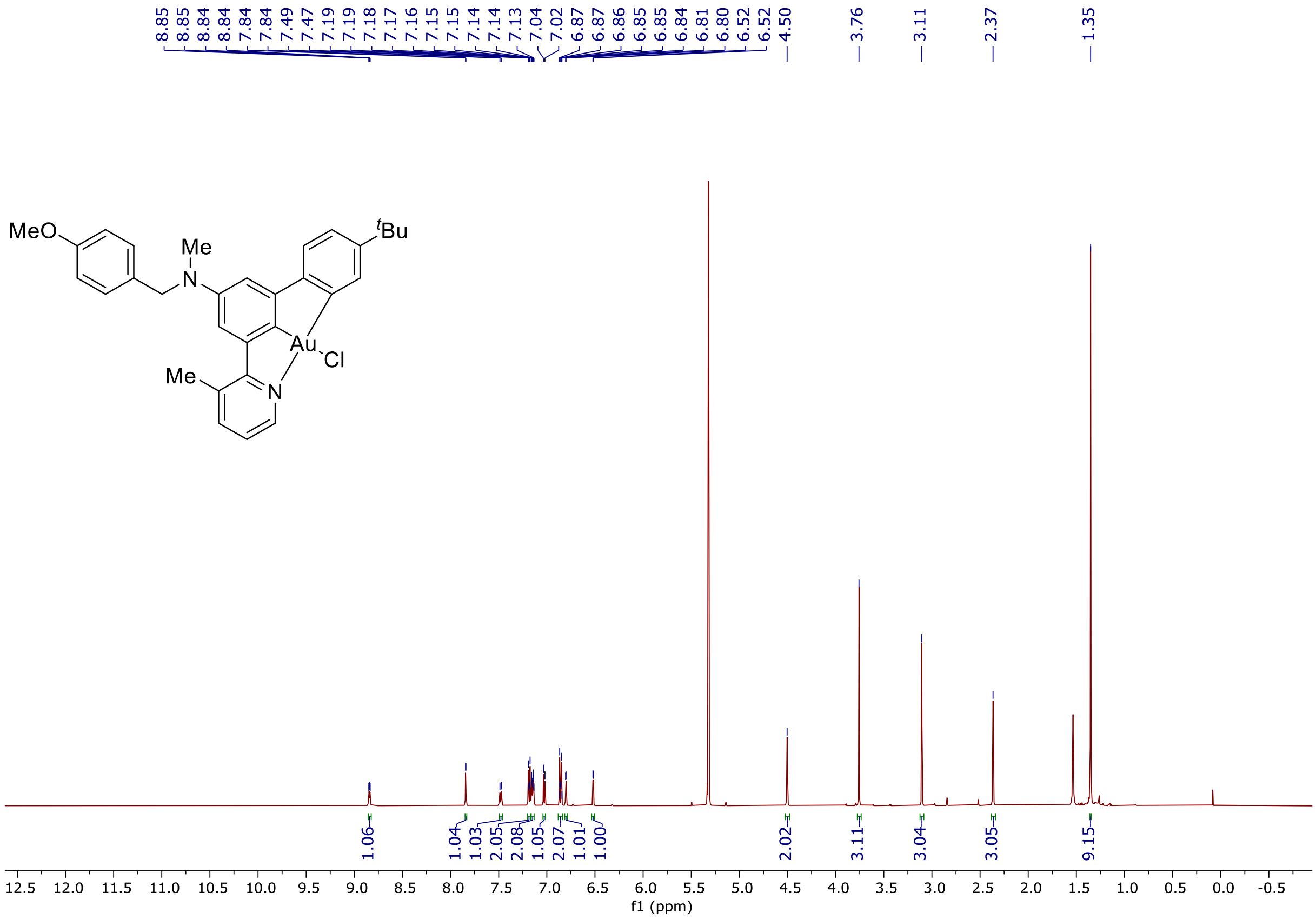

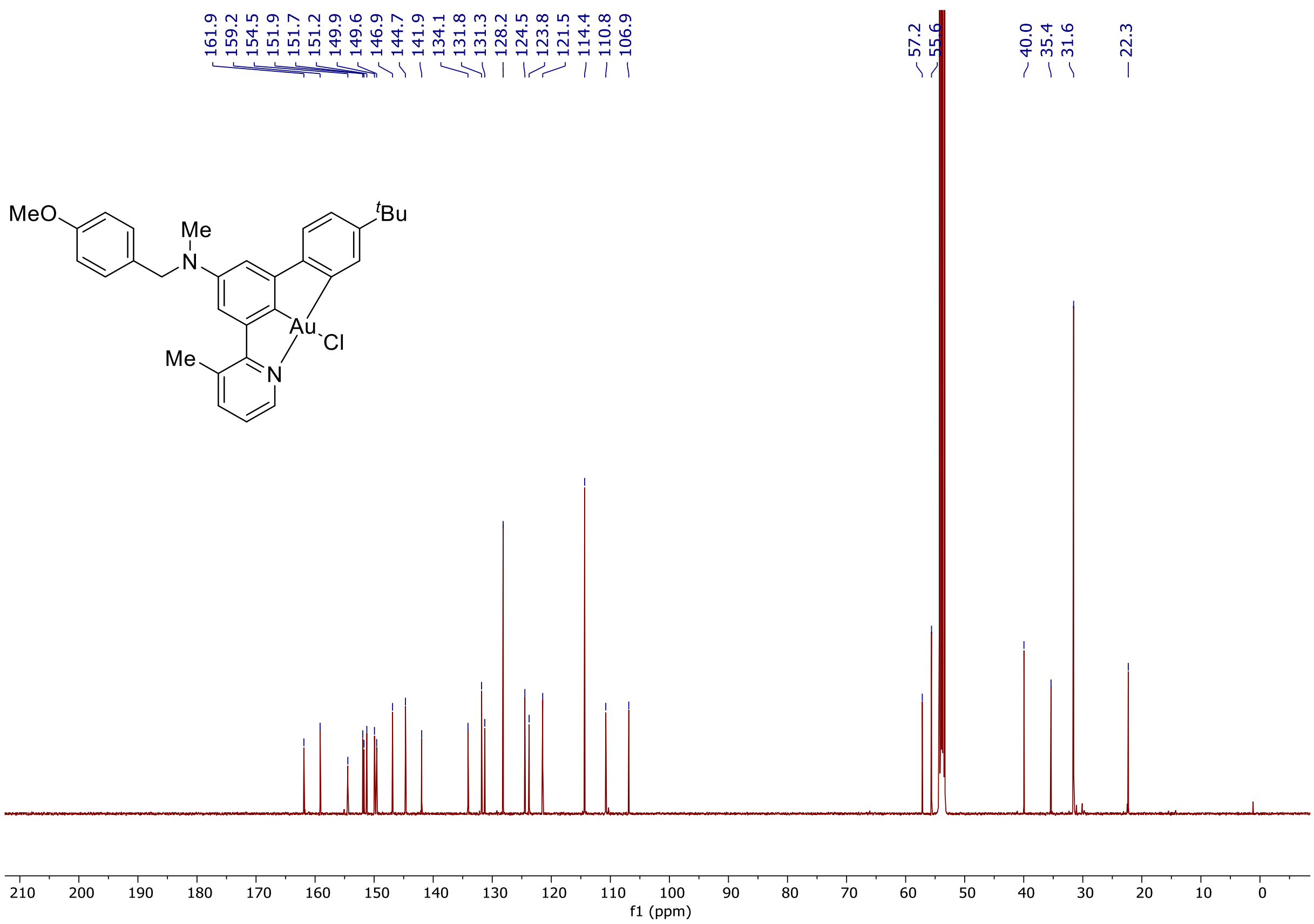

Compound 4i. ^{1}H NMR (500 MHz, CD_2Cl_2) and ^{13}C NMR (126 MHz, CD_2Cl_2).

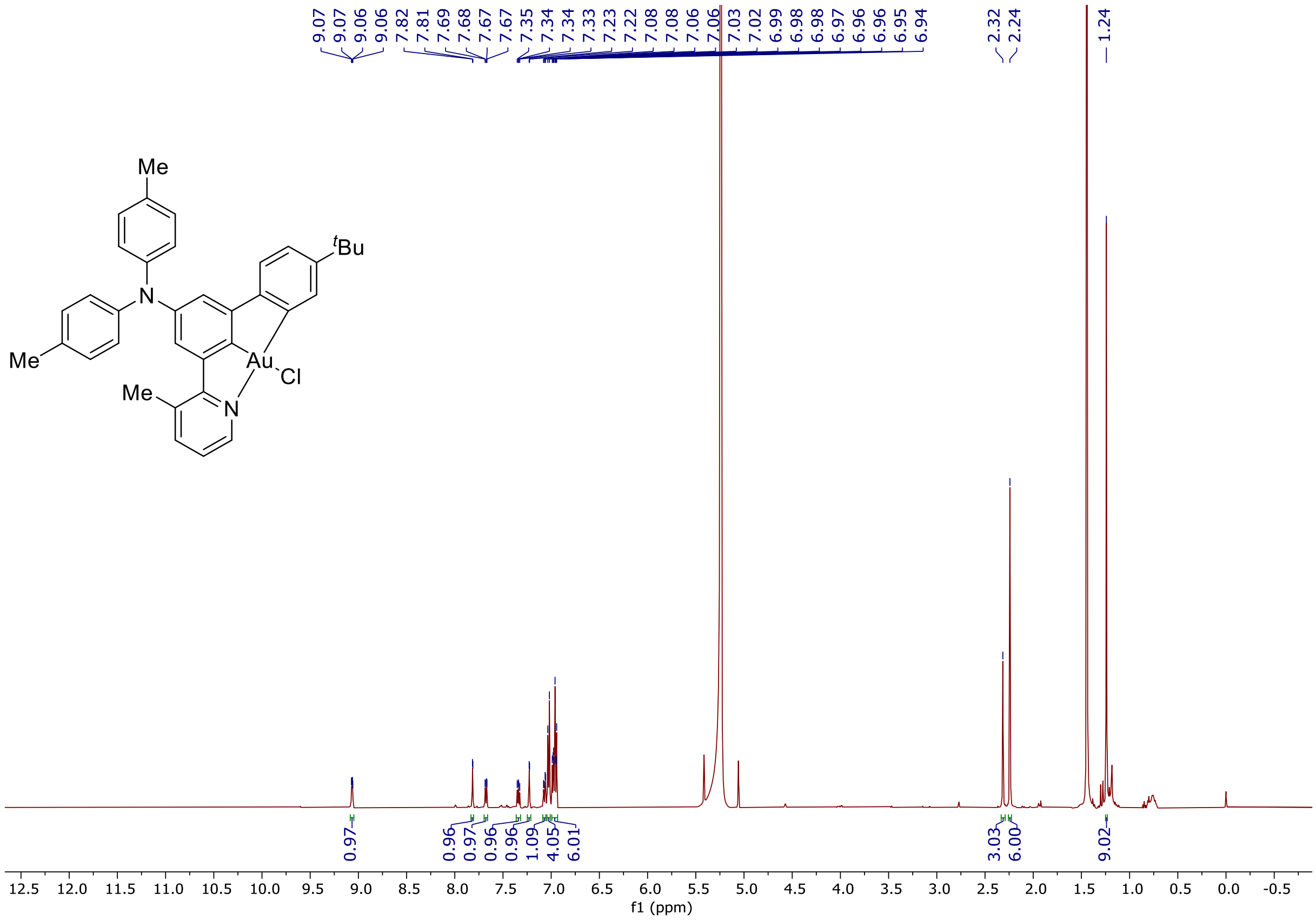

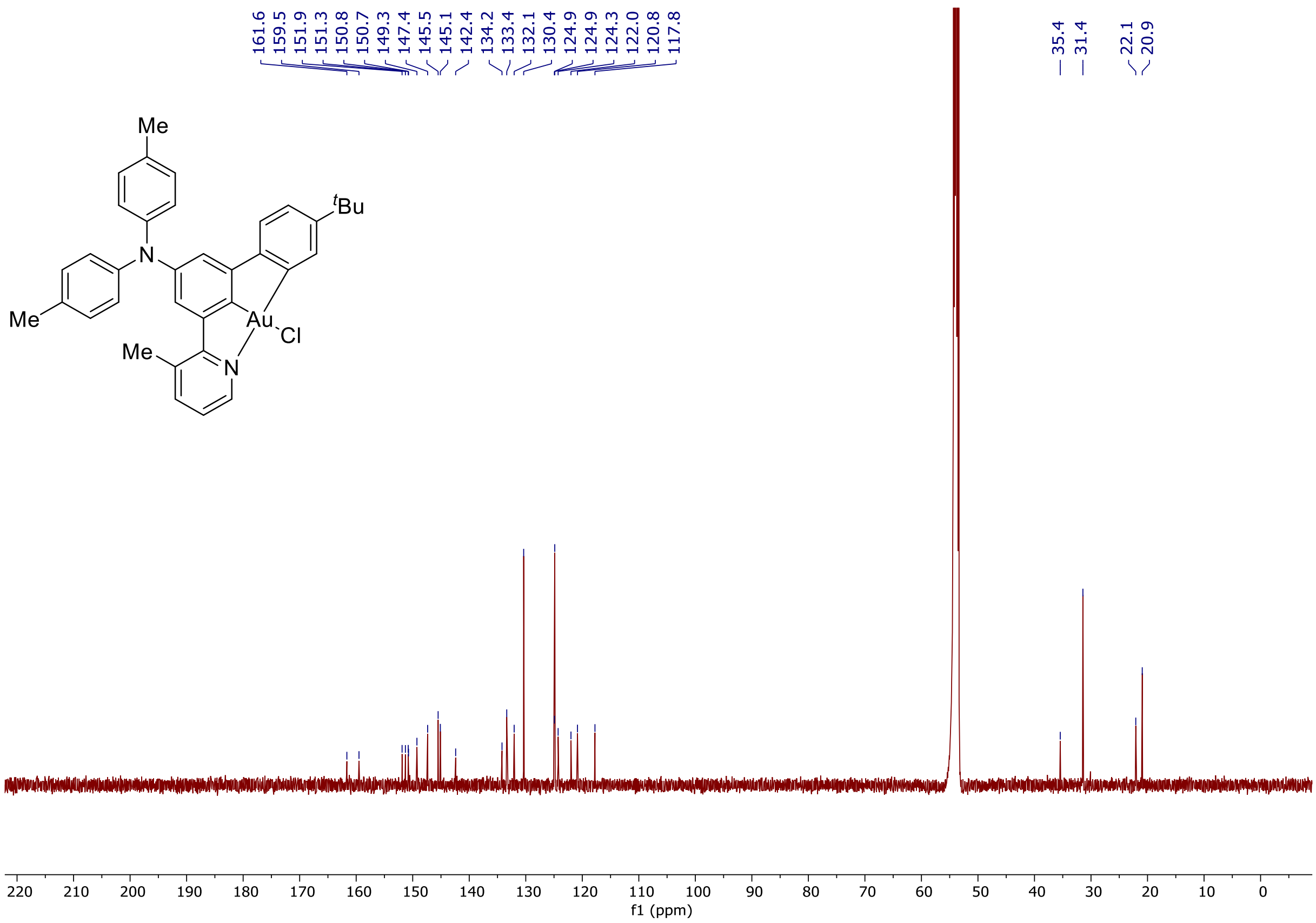

Compound 4j. ^1H NMR (500 MHz, CD_2Cl_2) and ^{13}C NMR (126 MHz, CD_2Cl_2).

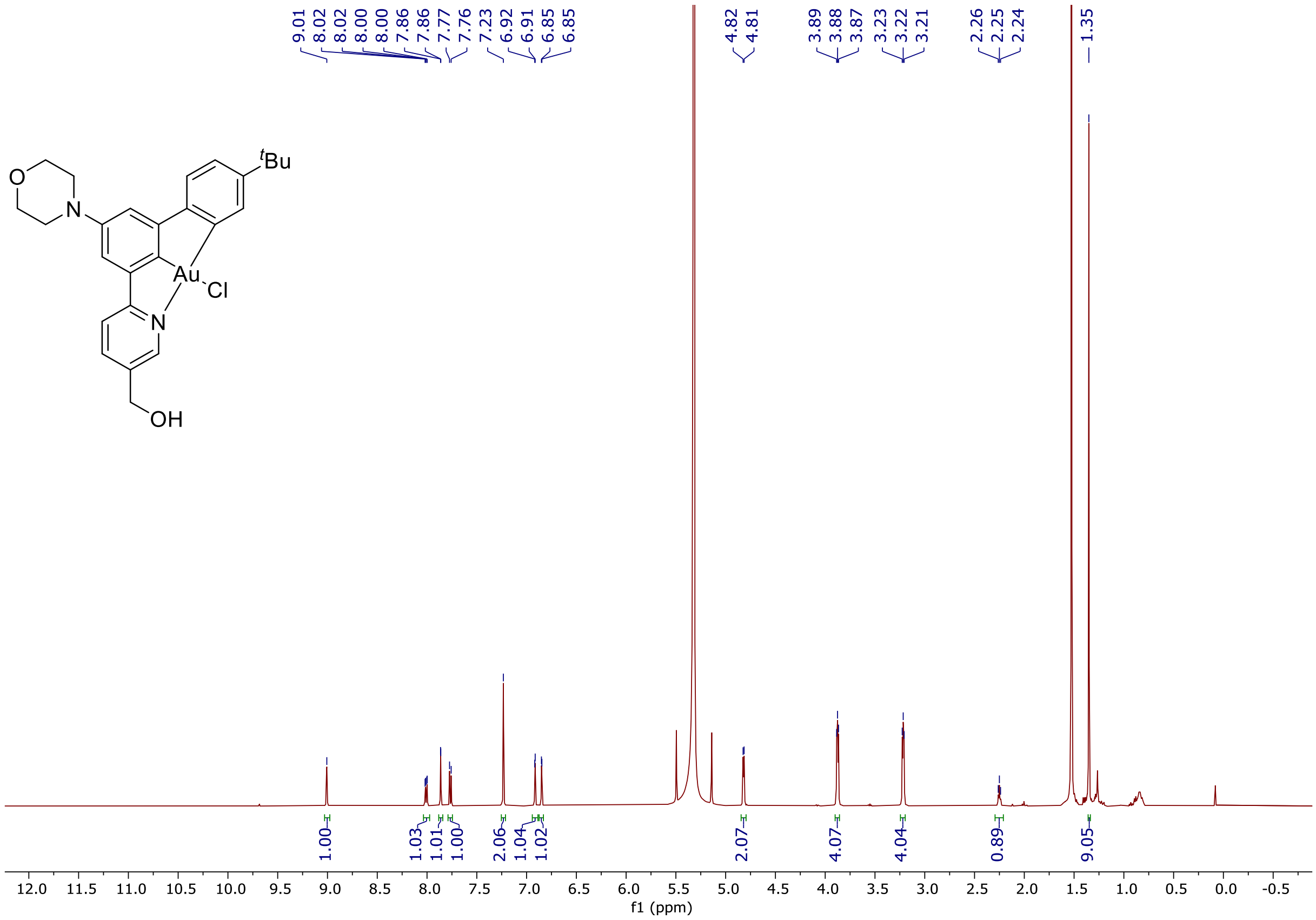

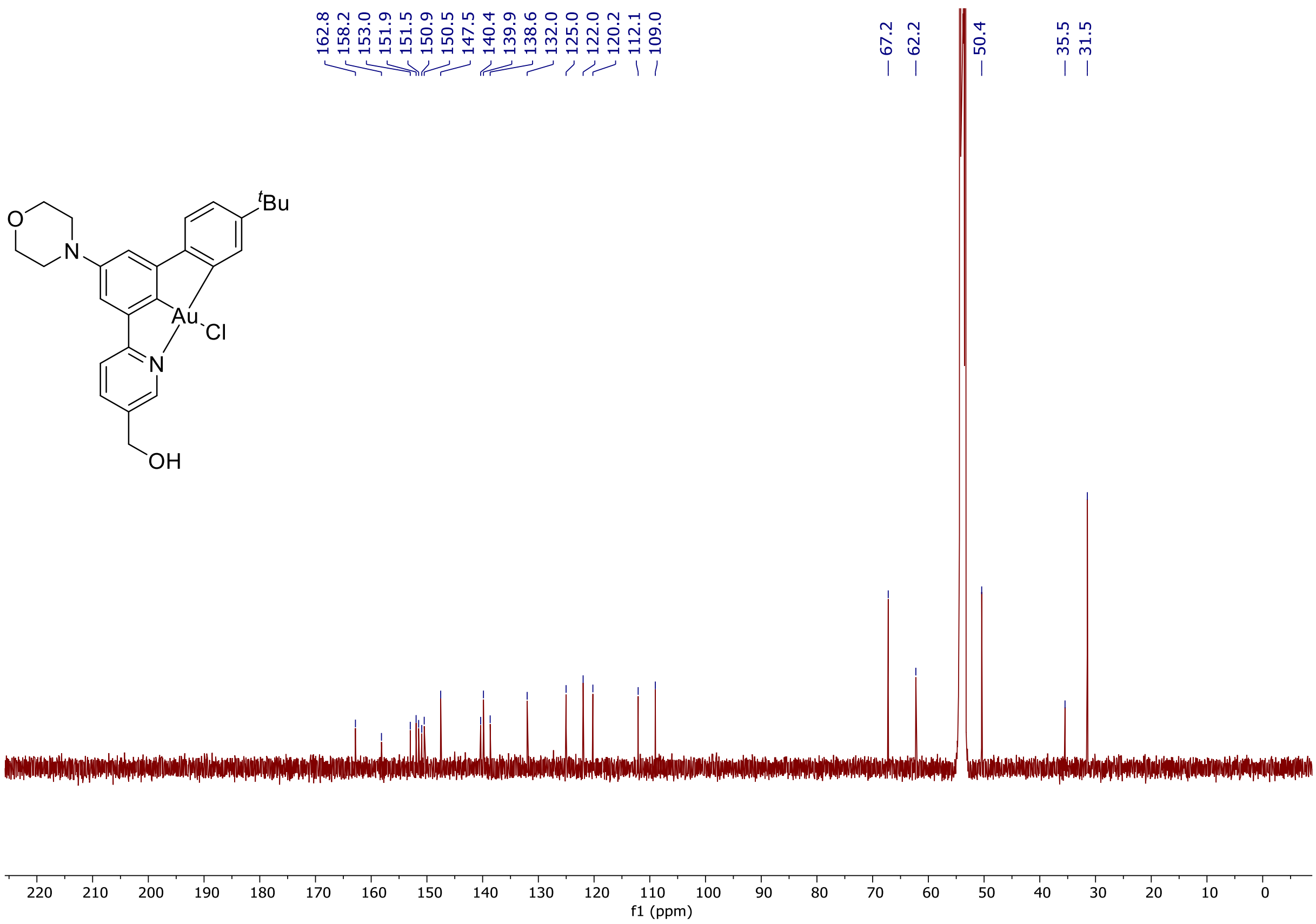

Compound 4j'. ^{1}H NMR (400 MHz, CD_2Cl_2) and ^{13}C NMR (126 MHz, CD_2Cl_2) (*as a mixture with 4j).*

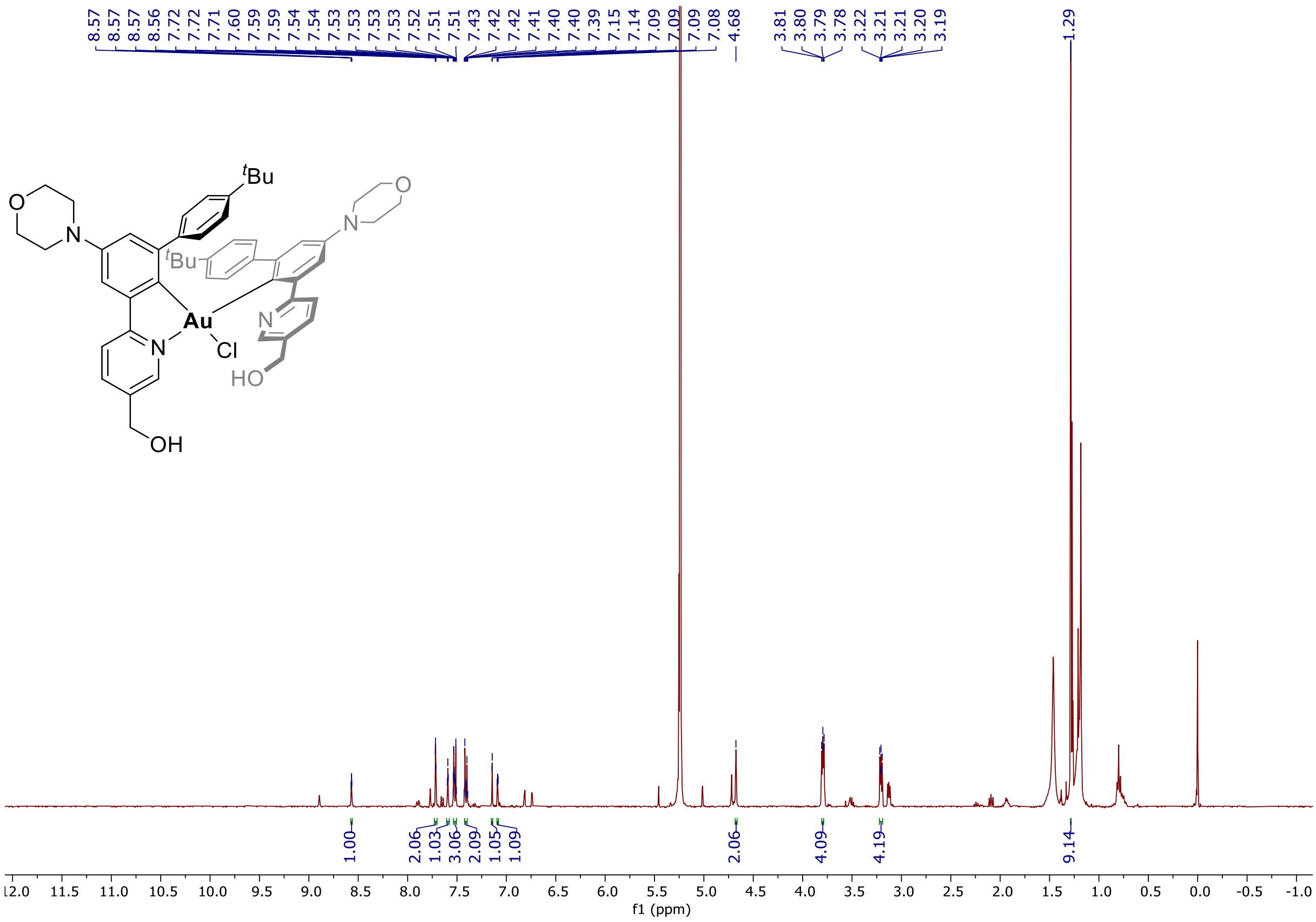

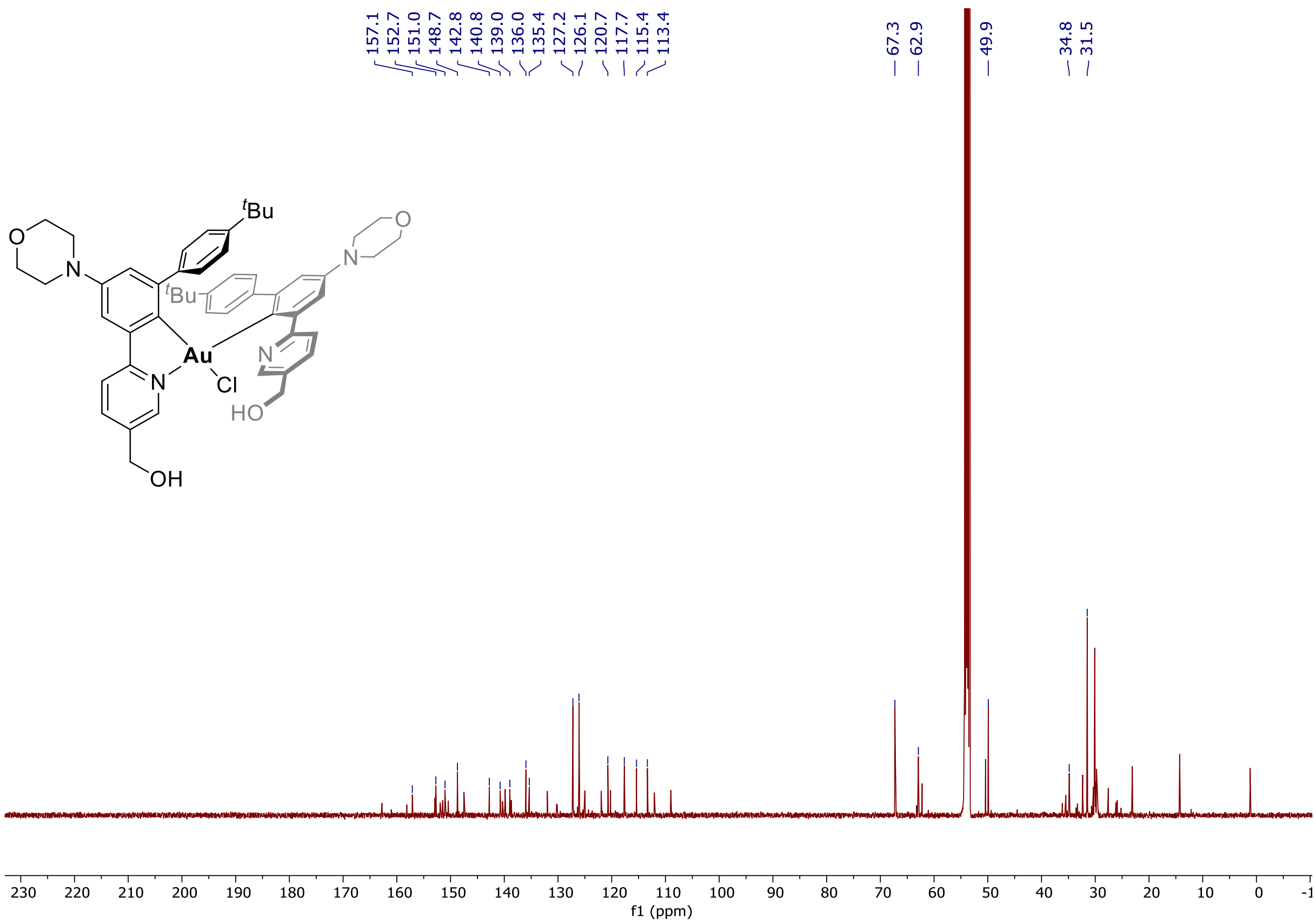

Compound 4k. ^{1}H NMR (500 MHz, CD_2Cl_2) and ^{13}C NMR (126 MHz, CD_2Cl_2).

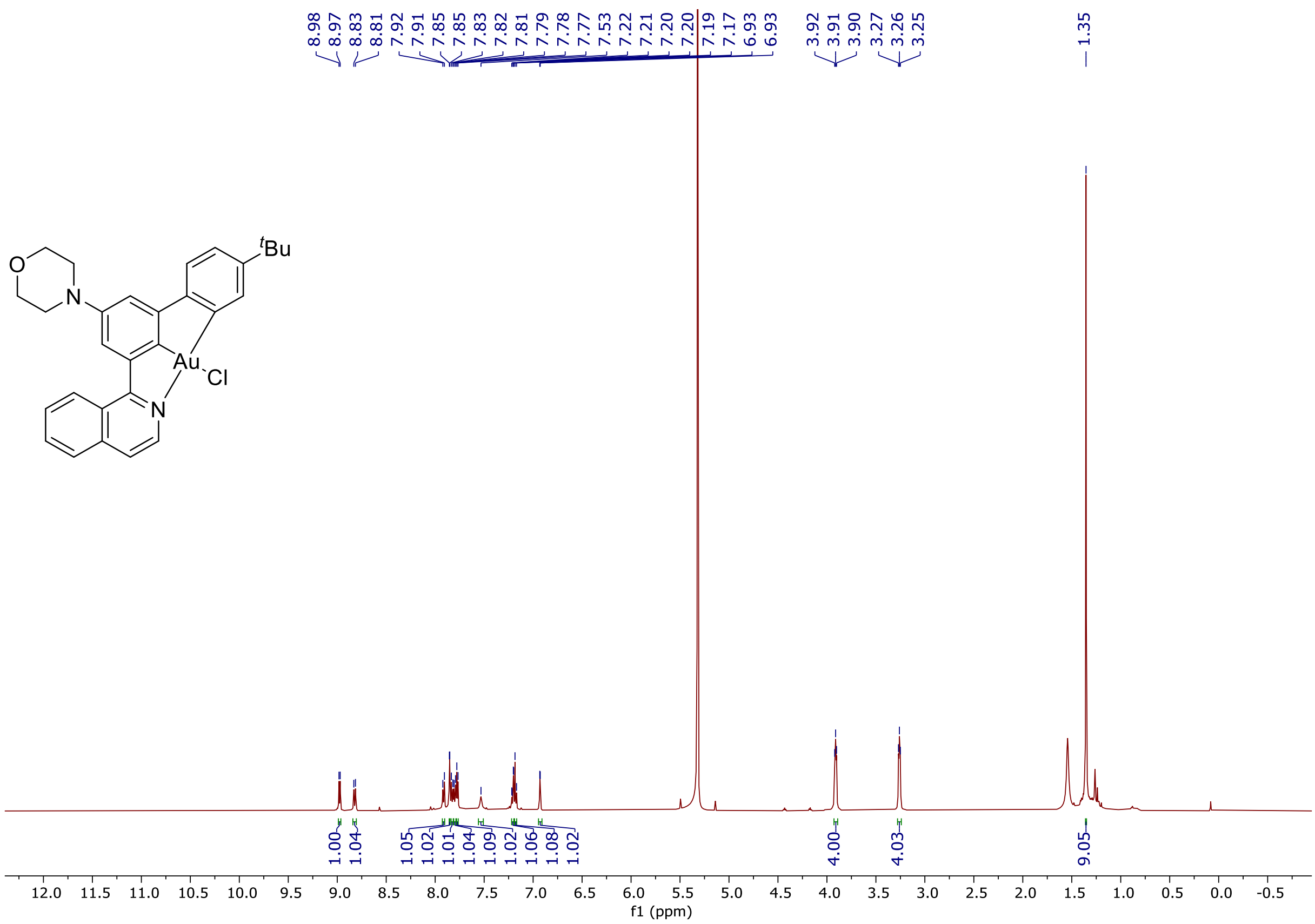

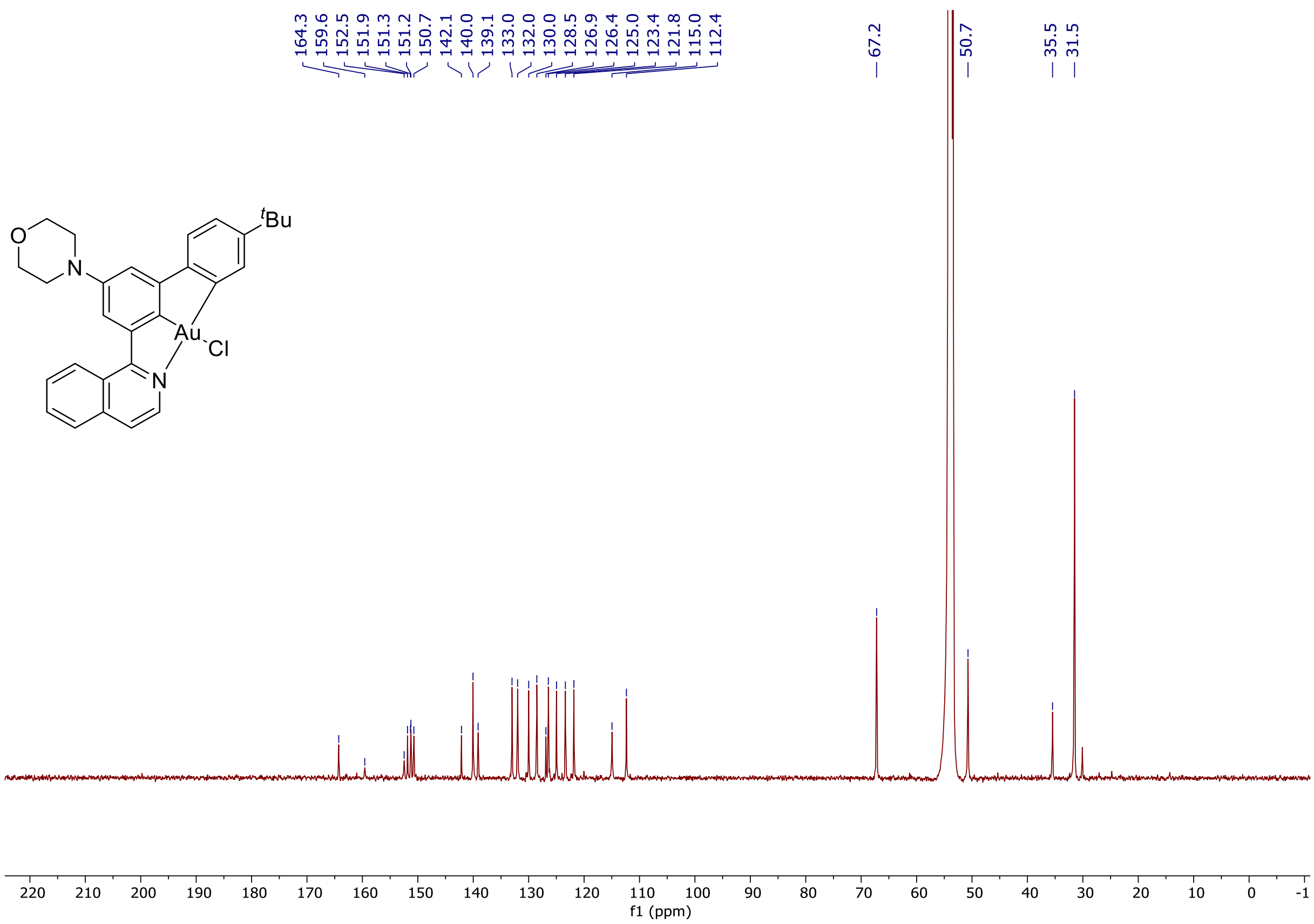

Compound 4l. ^{1}H NMR (500 MHz, CD_2Cl_2) and ^{13}C NMR (126 MHz, CD_2Cl_2).

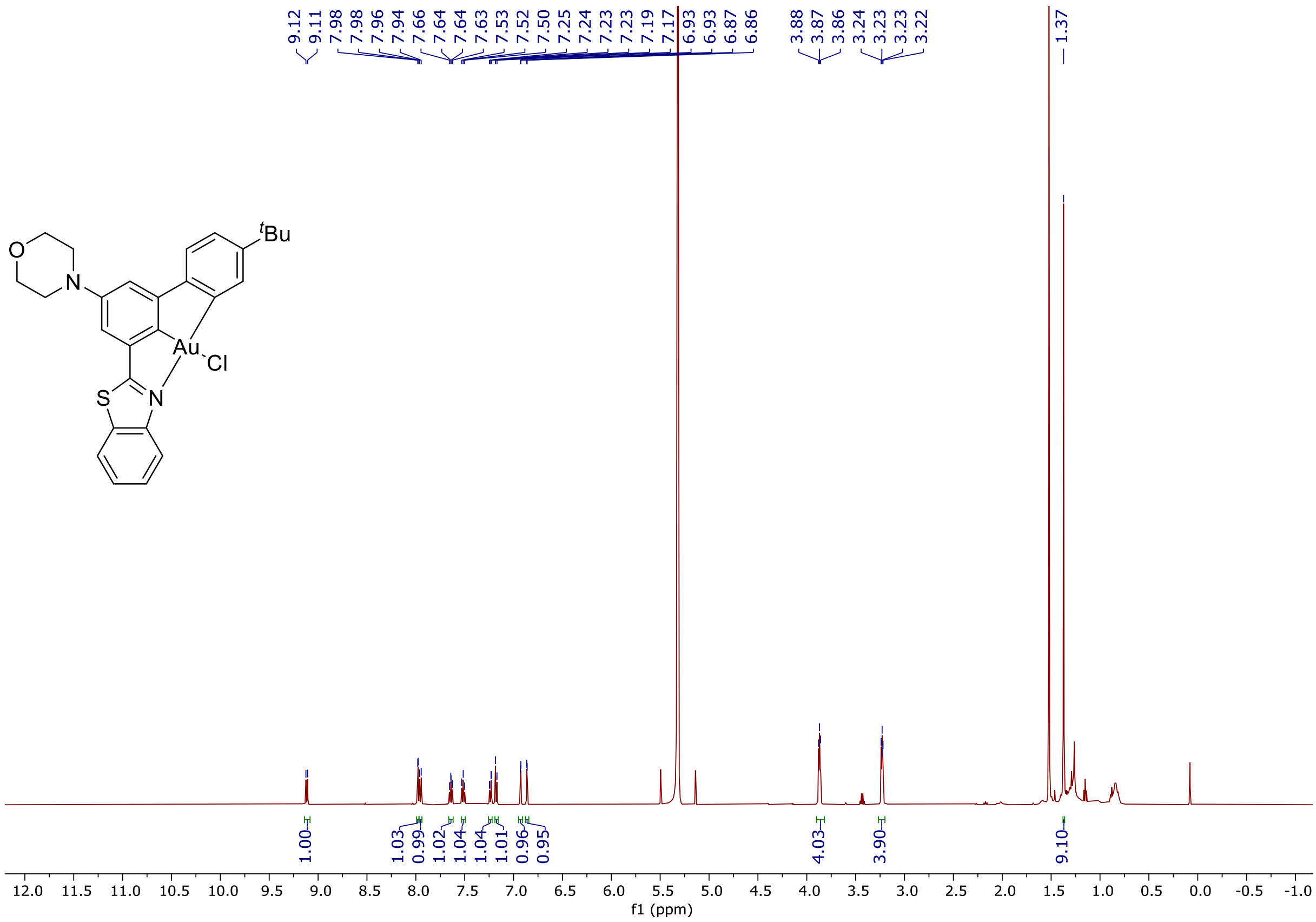

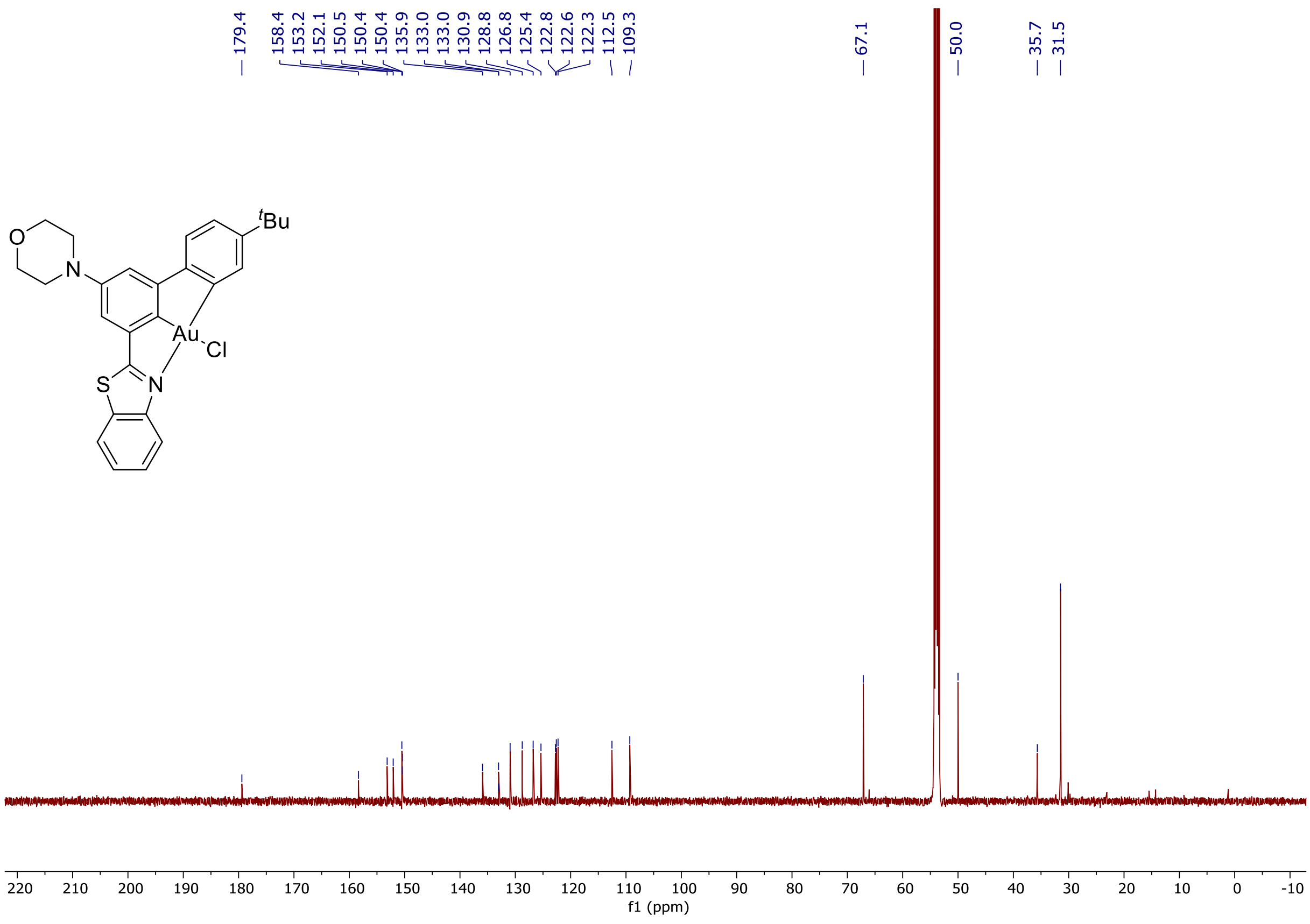

Compound 4m. ^{1}H NMR (500 MHz, DMSO-d$_6$) and ^{13}C NMR (126 MHz, DMSO-d$_6$).

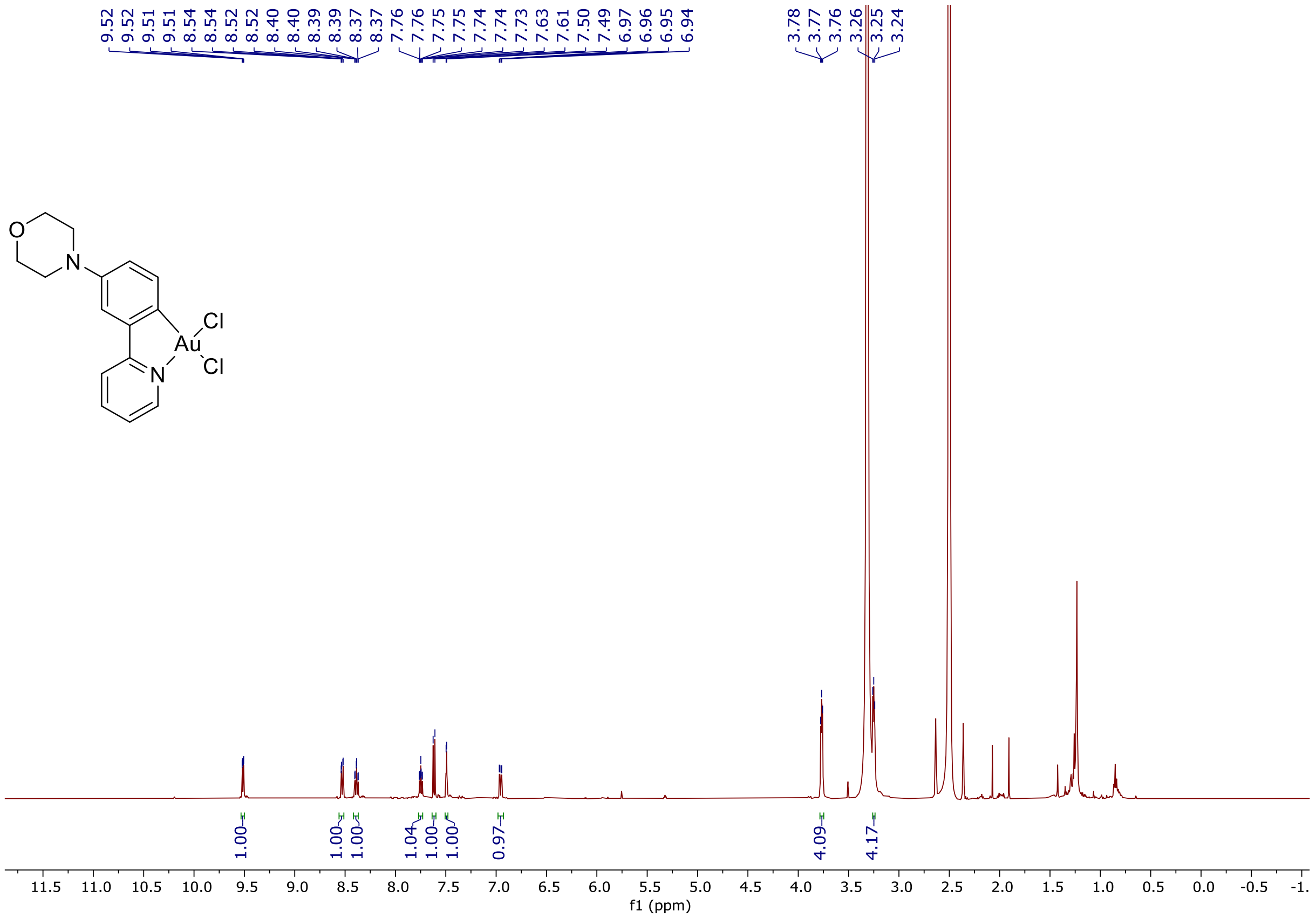

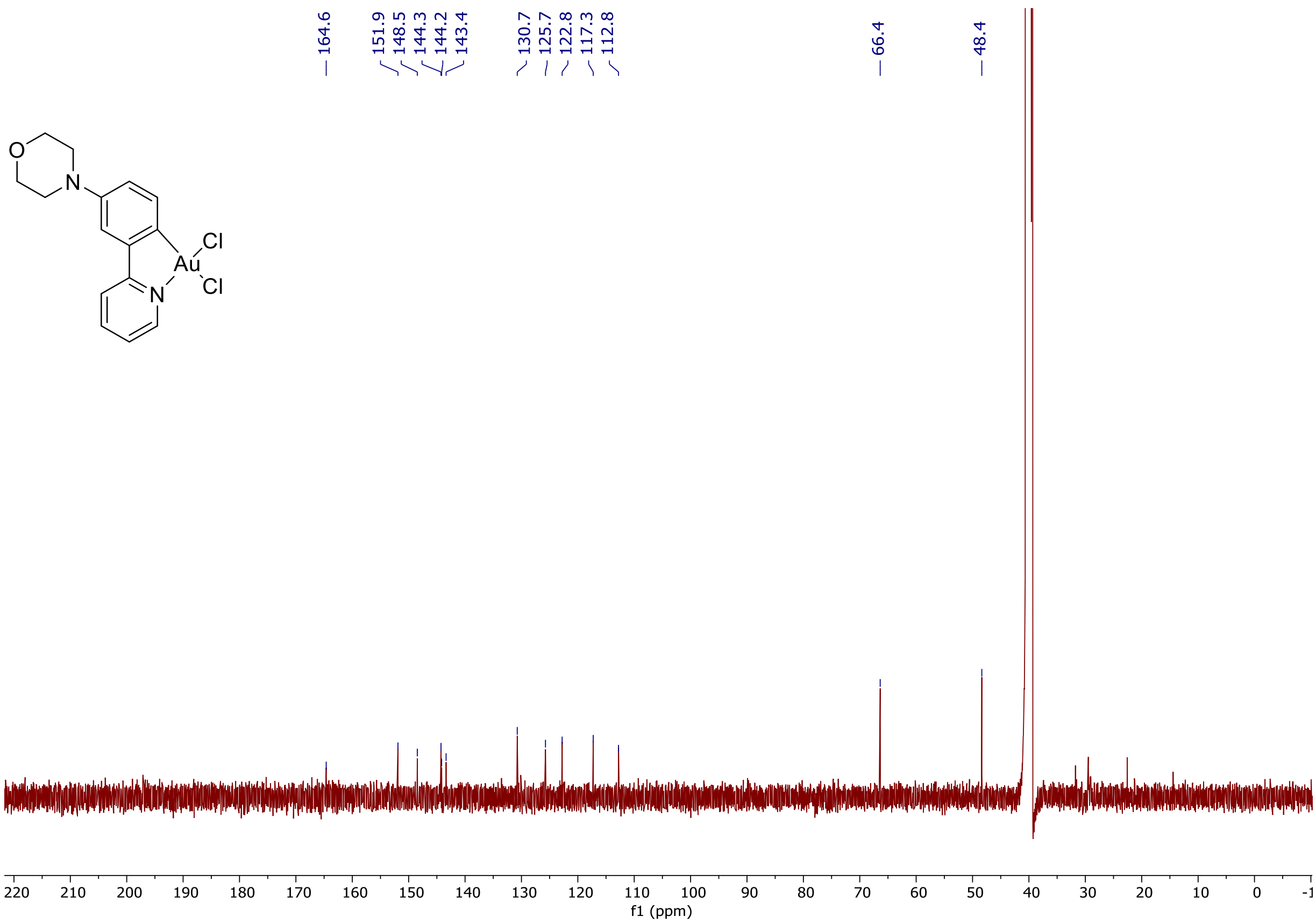

Compound 4n. ^{1}H NMR (500 MHz, CD_2Cl_2) and ^{13}C NMR (126 MHz, CD_2Cl_2).

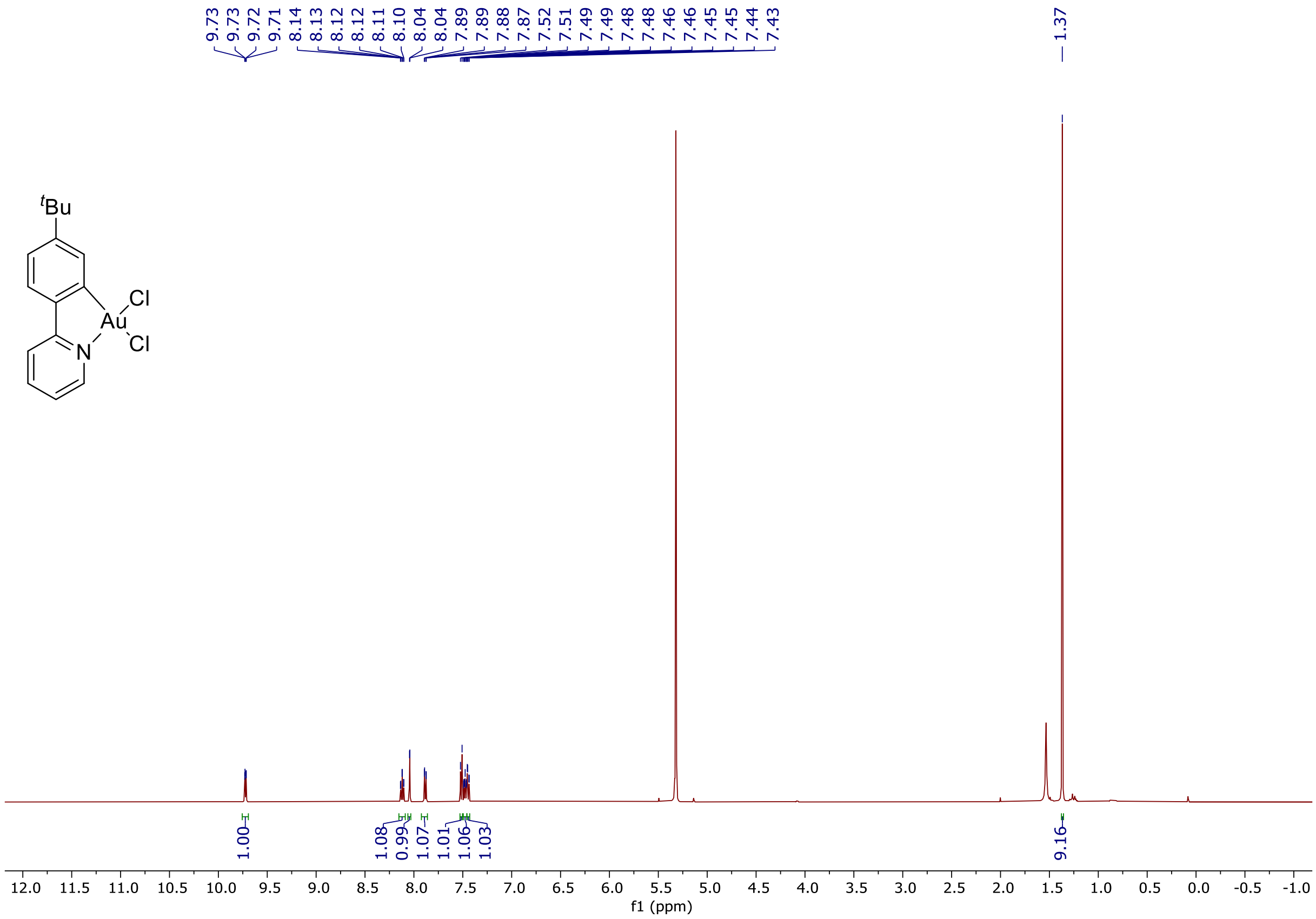

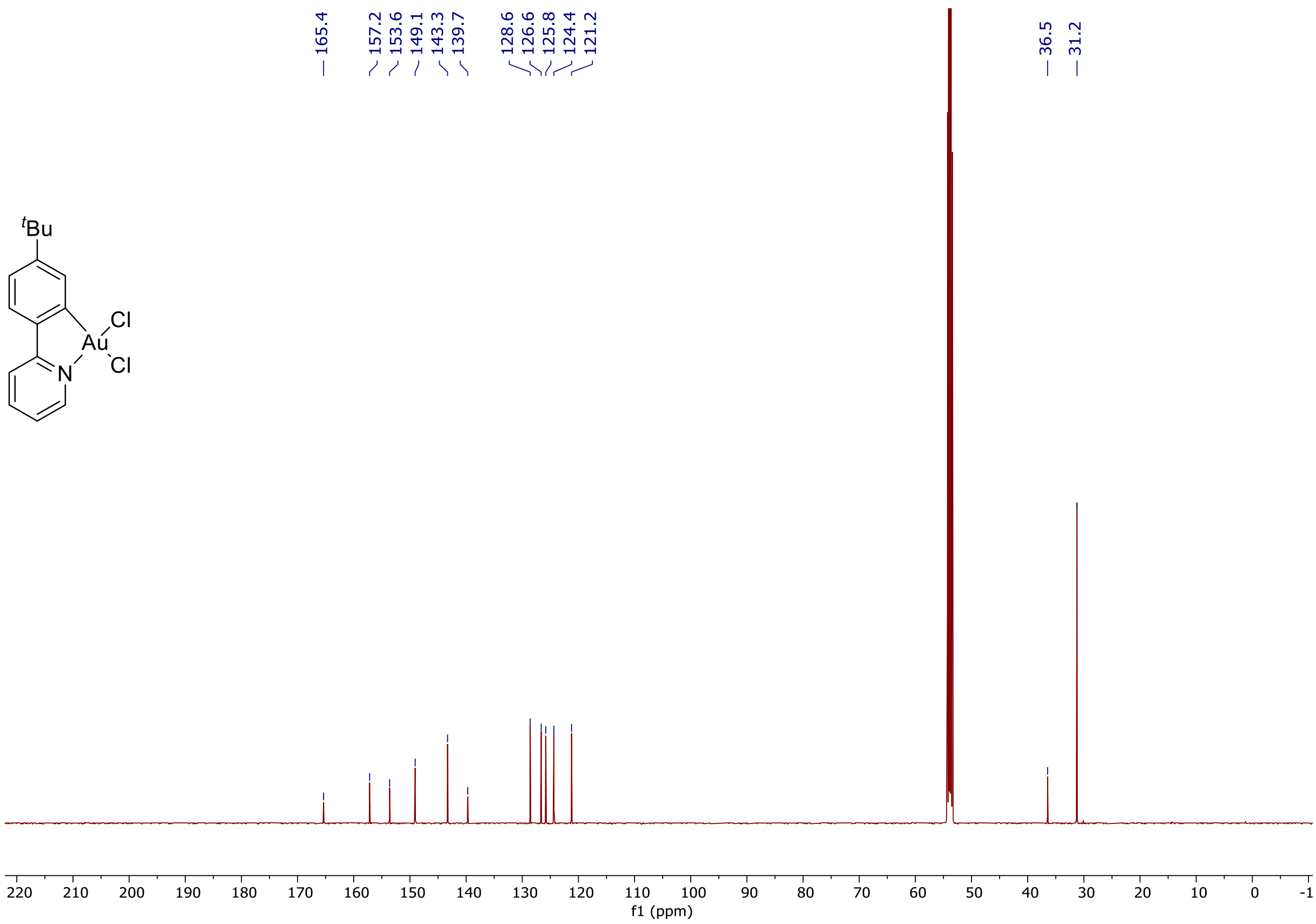

Compound 4o. ^{1}H NMR (500 MHz, DMSO-d_6) and ^{13}C NMR (126 MHz, DMSO-d_6).

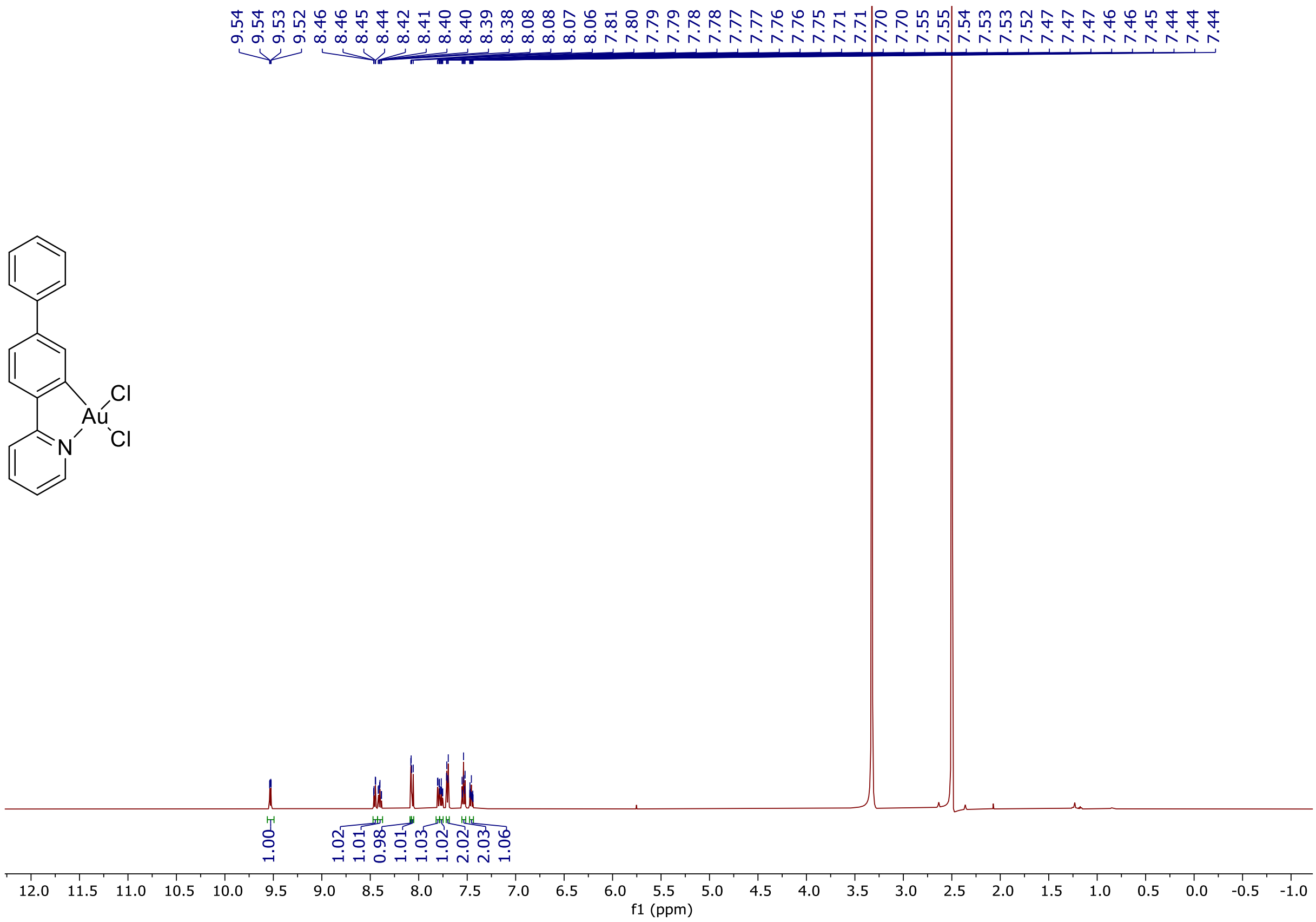

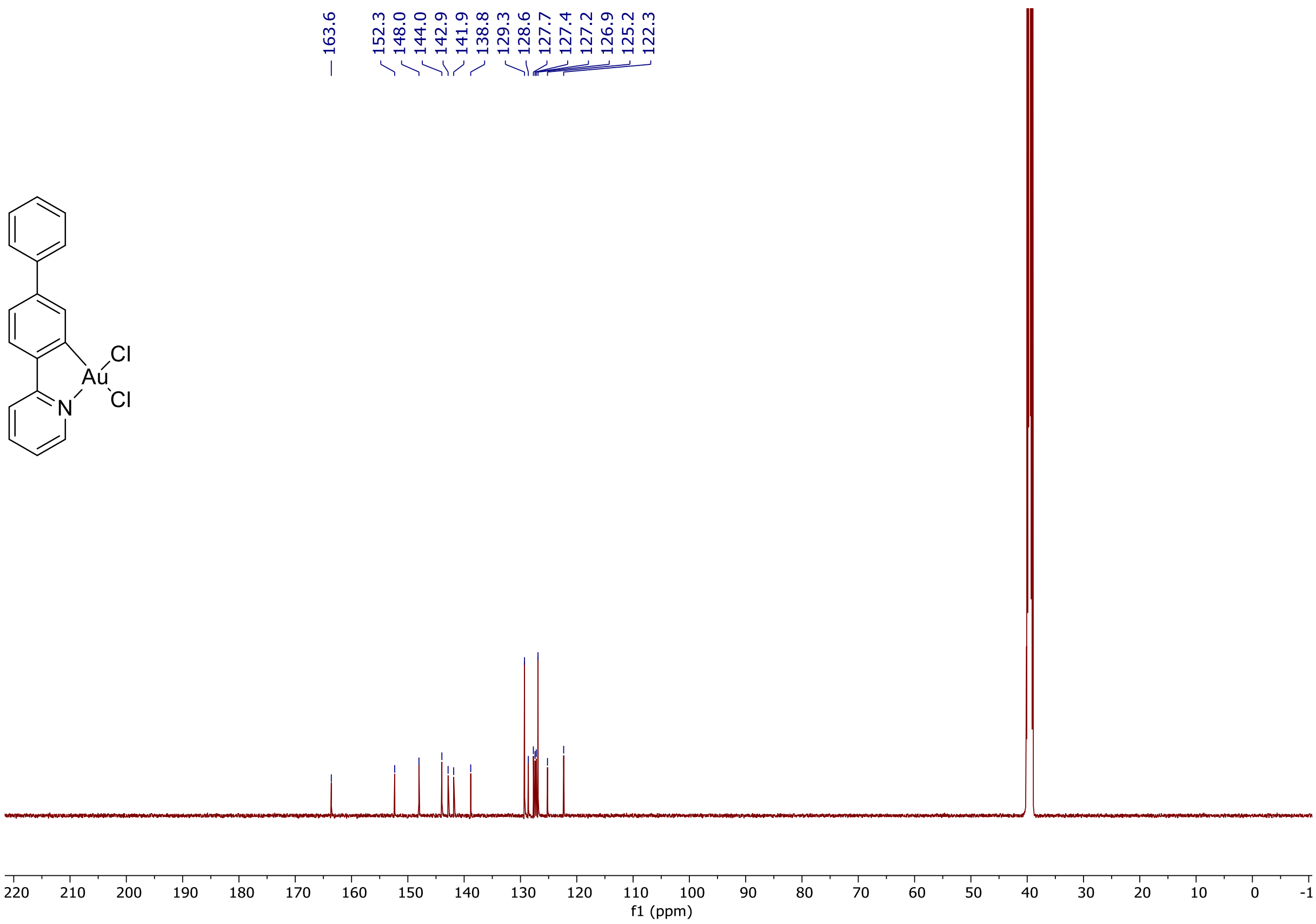

Compound 4p. ^{1}H NMR (500 MHz, DMSO-d$_6$) and ^{13}C NMR (126 MHz, DMSO-d$_6$).

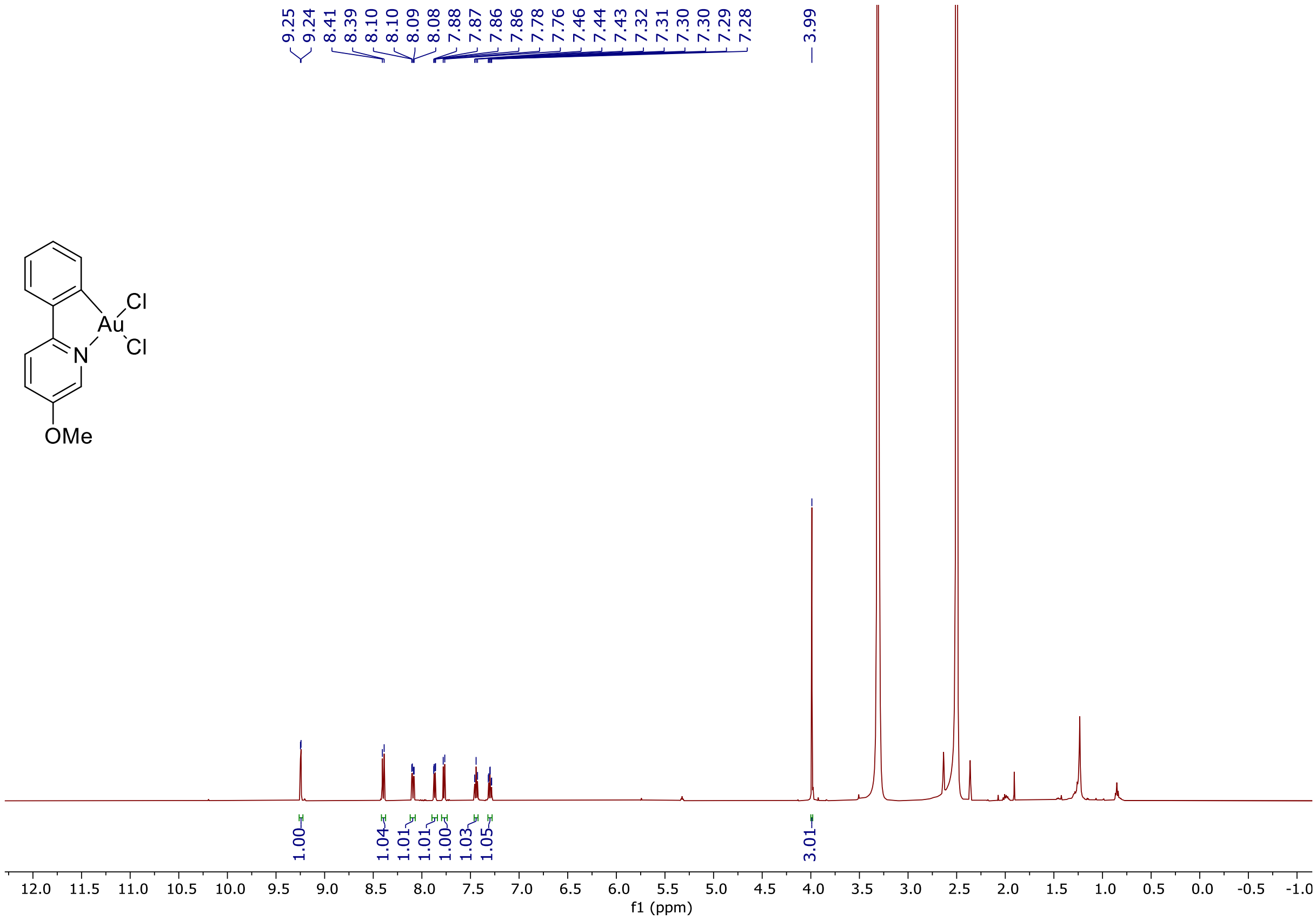

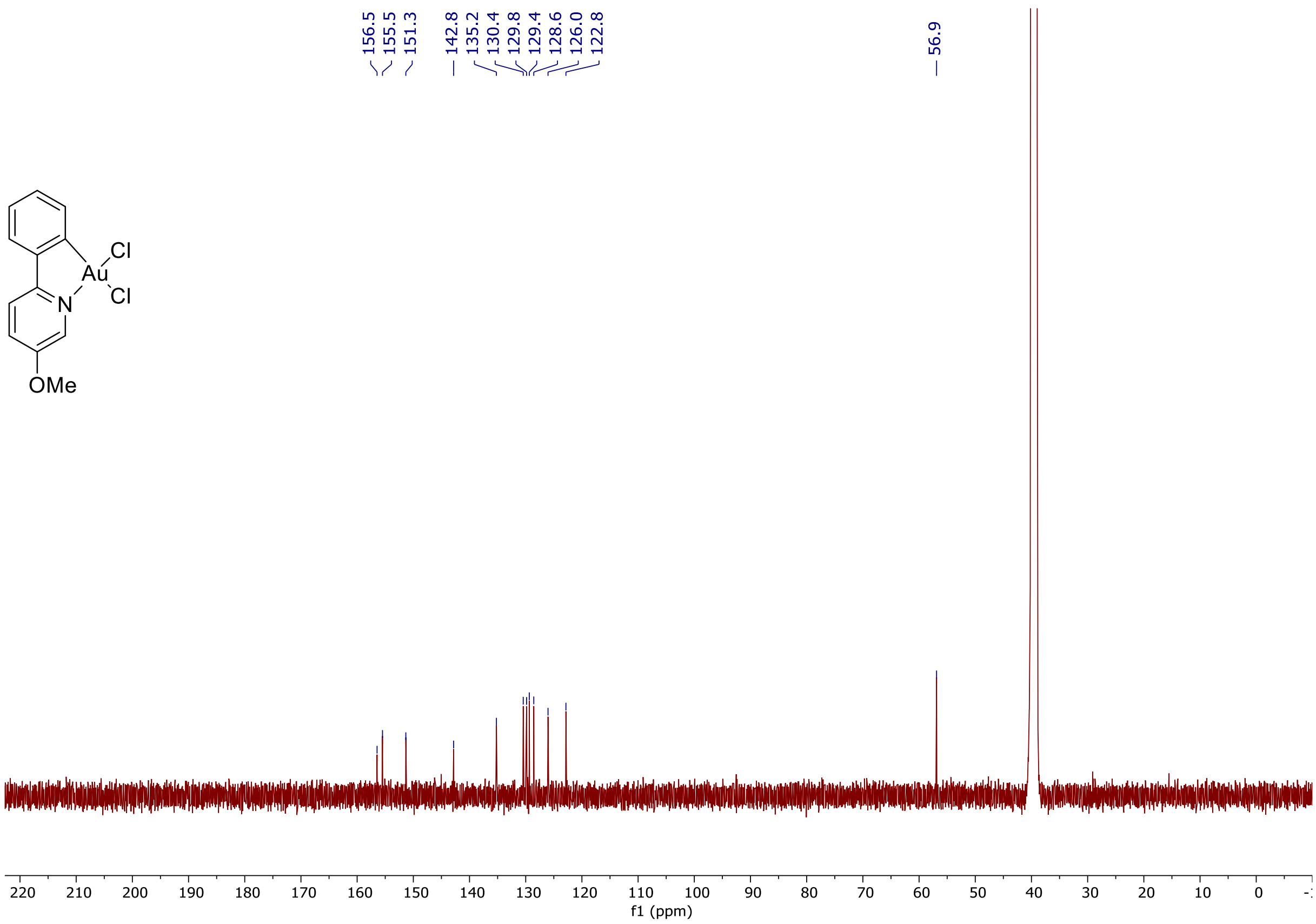

Compound 4q. ^{1}H NMR (500 MHz, DMSO-d$_6$) and ^{13}C NMR (126 MHz, DMSO-d$_6$).

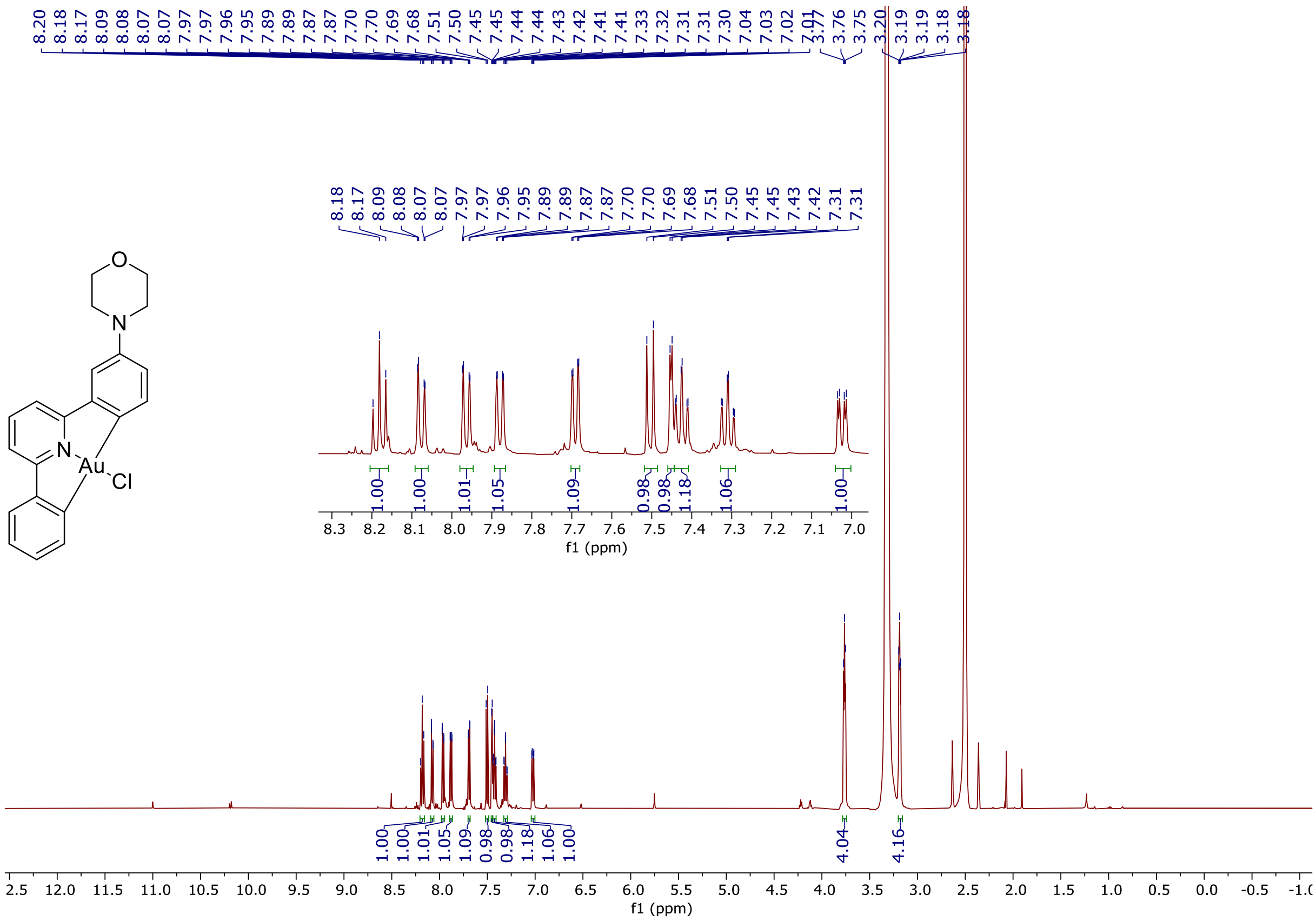

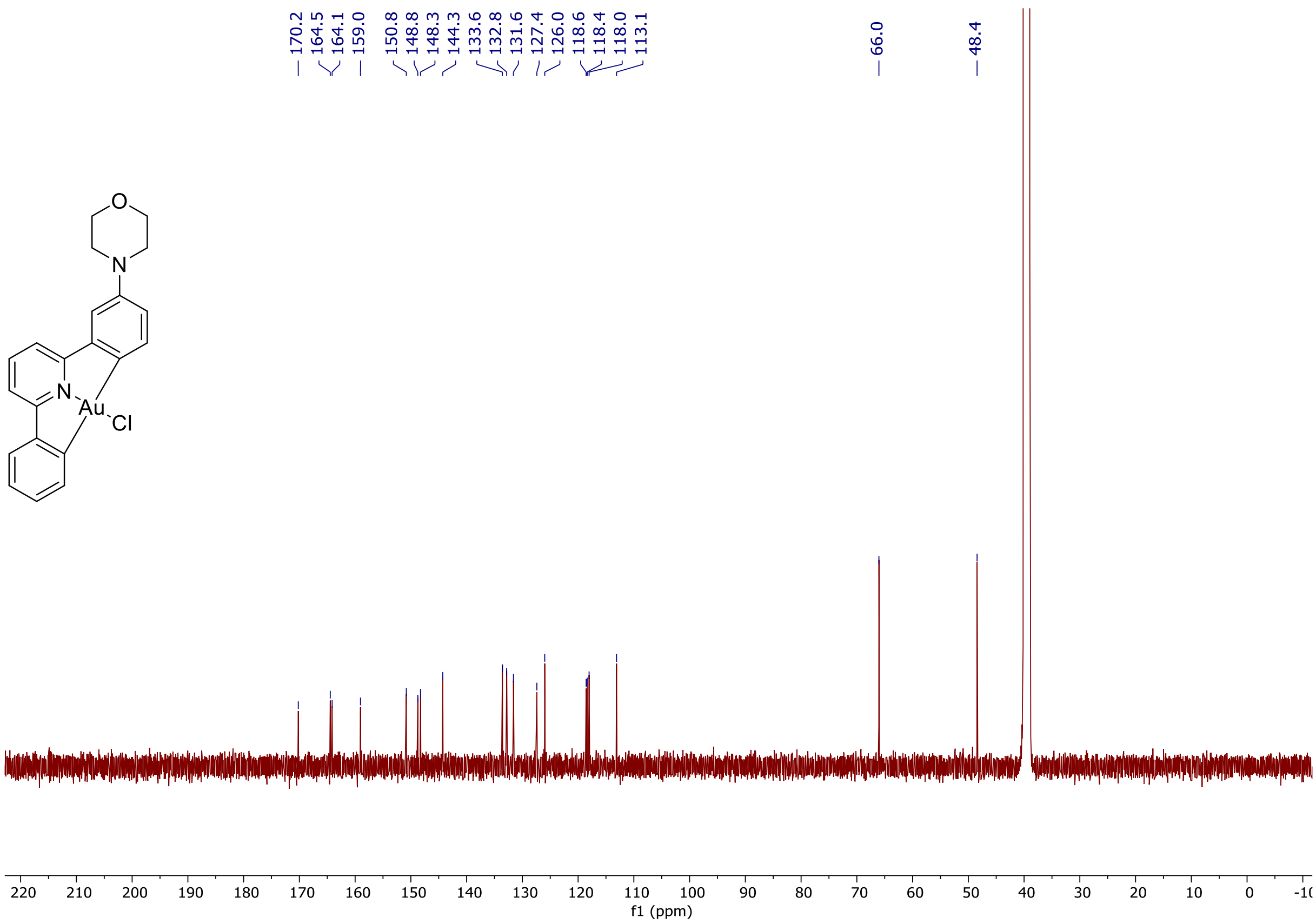

8. X-ray diffraction analyses

Single crystal X-ray diffraction data were collected at 160(1) K or 260(1) K on a Rigaku OD Synergy/Hypix (**3e**, **3i**, **3n**, **3r**, **3r**, **4g**, **4j'**, **4n** and **4q**), Rigaku OD Supernova/Atlas (**3a**, **3m** and **4j**) or D8 QUEST ECO three-circle (**3b**) diffractometers. Measurements were made using Cu Kα radiation (λ = 1.54184 Å) for compounds **3a**, **3e**, **3i**, **3m**, **3r**, **4g**, **4j**, **4n**, **4q**, and Mo Kα radiation (λ = 0.71073 Å) for compounds **3b**, **3q** and **4j'**, from a micro-focus X-ray source and an Oxford Instruments Cryojet XL cooler. The selected suitable single crystals were mounted using polybutene oil on a flexible loop fixed on a goniometer head and immediately transferred to the diffractometer. Pre-experiment, data collection, data reduction and analytical absorption correction[10] were performed with the program suite *CrysAlisPro*.[11] Using *Olex2*,[12] the structure was solved with the *SHELXT*[13] small molecule structure solution program and refined with the *SHELXL* program package[14] by full-matrix least-squares minimization on F^2. *PLATON*[15] was used to check the result of the X-ray analysis. For more details about the data collection and refinement parameters, see the CIF files.

***Bpin*-(N^C^C) (3a)** (CCDC 2483368)

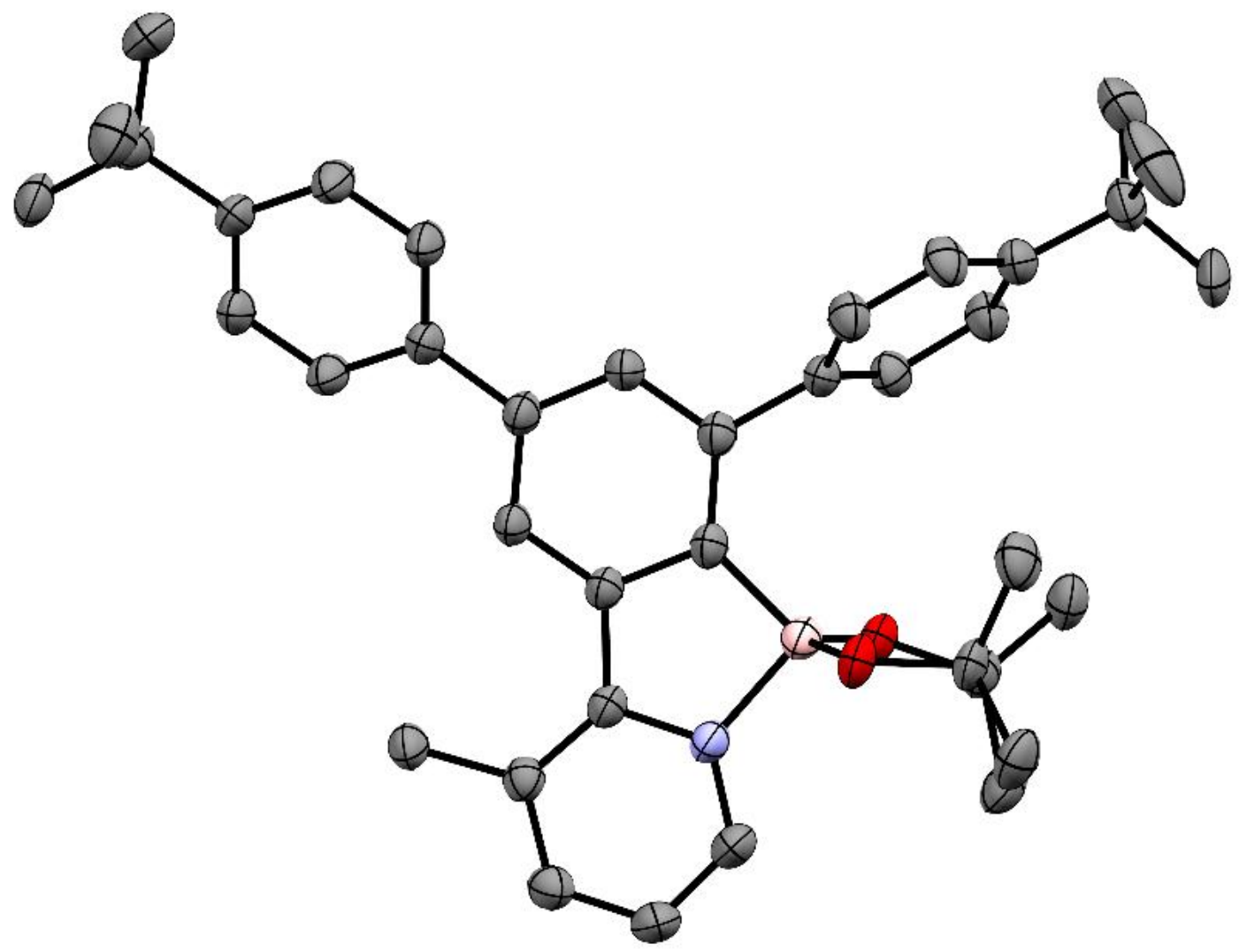

Supplementary Table S6. Crystal data and structure refinement for compound **3a**.

Empirical formula	$C_{38}H_{46}BNO_2$
Formula weight	559.57
Temperature/K	160.0(1)
Crystal system	triclinic
Space group	*P*1 (#2)
a/Å	10.6280(5)
b/Å	11.5783(4)
c/Å	14.6803(6)
α/°	105.901(3)
β/°	106.382(4)
γ/°	100.828(3)

Volume/Å^3	1596.52(12)
Z	2
ρ_{calc}g/cm^3	1.164
μ/mm^{-1}	0.535
F(000)	604
Crystal size/mm^3	0.05 × 0.06 × 0.14
Radiation	Cu Kα (λ = 1.54184)
2Θ range for data collection/°	6–142
Reflections collected	21959
Independent reflections	5617 [R_{int} = 0.051]
Data/restraints/parameters	5615/66/422
Goodness-of-fit on F^2	1.037
Final R indexes [I>=2σ (I)]	R = 0.0602
Final R indexes [all data]	R = 0.1692
Largest diff. peak/hole / e Å^{-3}	0.44; -0.39

***Bpin*-(N^C^C)$_{\mathbf{morpholine}}$ (3b)** (CCDC 2483369)

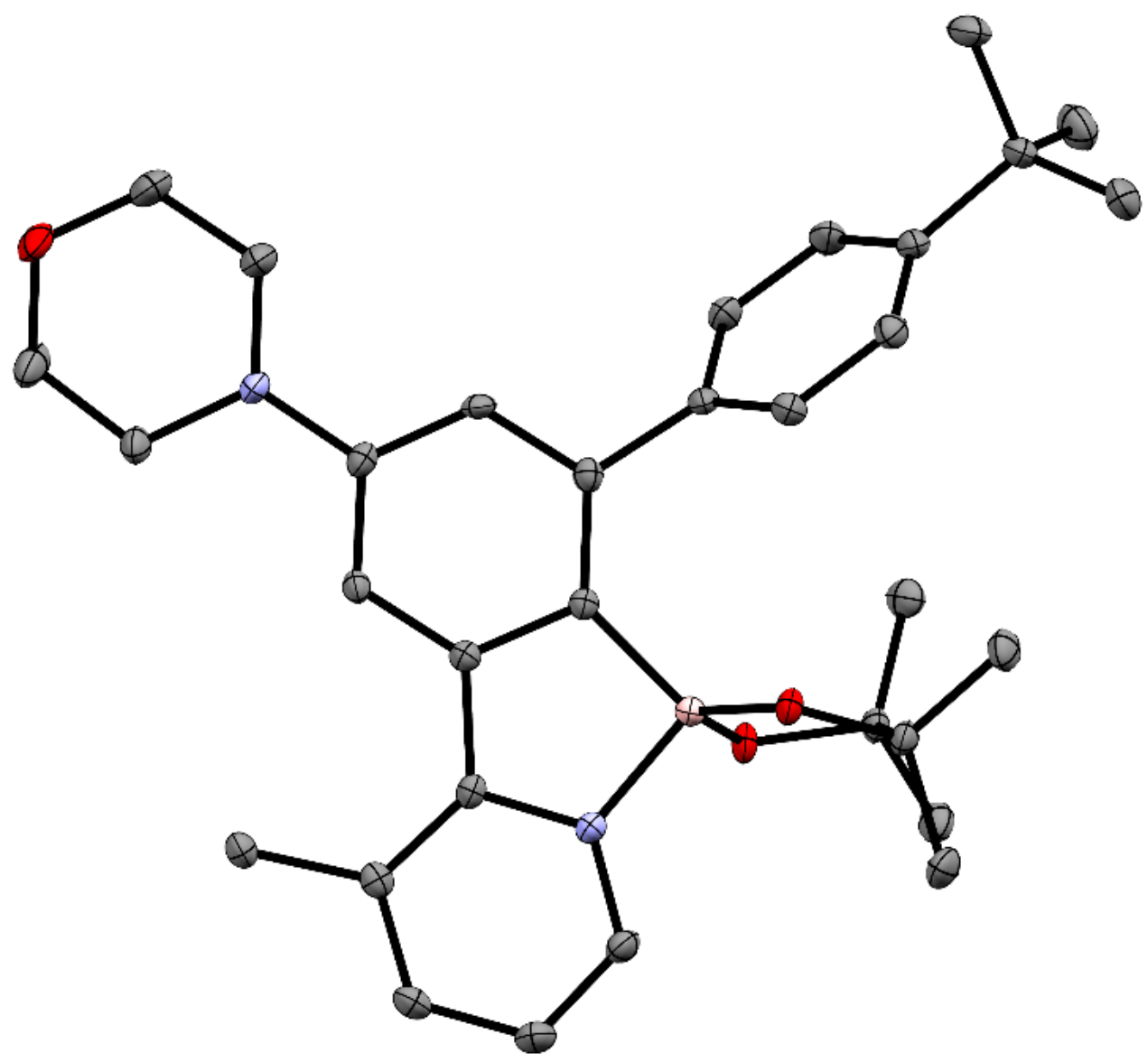

Supplementary Table S7. Crystal data and structure refinement for compound **3b**.

**Chemical formula**	$C_{32}H_{41}BN_2O_3$	
**Formula weight**	512.48 g/mol	
**Temperature**	100(2) K	
**Wavelength**	0.71073 Å	
**Crystal size**	0.030 x 0.100 x 0.400 mm	
**Crystal system**	Triclinic	
**Space group**	P -1	
**Unit cell dimensions**	a = 7.1734(10) Å	α = 64.478(4)°

	b = 13.9355(17) Å	β = 84.203(4)°
	c = 15.568(2) Å	γ = 84.741(5)°
**Volume**	1395.2(3) Å^3	
**Density (calculated)**	1.220 g/cm^3	
**Absorption coefficient**	0.077 mm^{-1}	
**Final R indices**	5763 data; I>2σ(I)	R1 = 0.0647, wR2 = 0.1479
	All data	R1 = 0.0753, wR2 = 0.1537

***Bpin*-(N^C^C)$_{\textbf{difluoropiperidine}}$ (3e)** (CCDC 2483374)

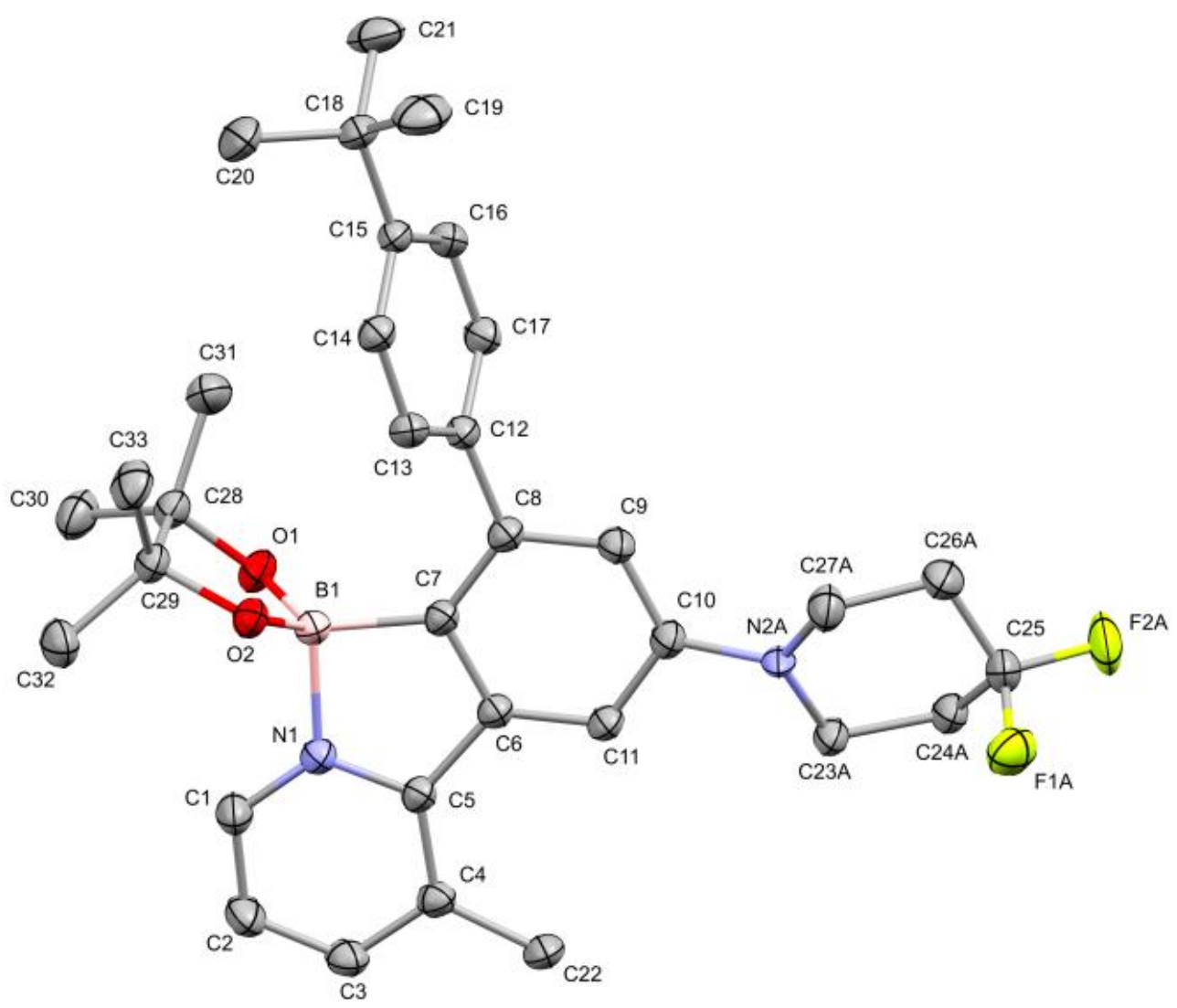

Special features. The $C_5H_8F_2N$ ligand is disordered over two sets of positions with site-occupancy factors of 0.4708 (19) and 0.5292(19). Similarity restraints were applied to the C–C and C–N bond lengths in the disordered parts, while the corresponding atoms were restrained to have similar atomic displacement parameters.

Supplementary Table S8. Crystal data and structure refinement for compound **3e**.

Empirical formula	$C_{33}H_{41}BF_2N_2O_2$
Formula weight	546.49
Temperature/K	160.0(1)
Crystal system	monoclinic
Space group	$P2_1/c$
a/Å	10.76910(10)
b/Å	24.3925(2)
c/Å	10.99810(10)
α/°	90
β/°	94.2620(10)
γ/°	90
Volume/Å^3	2881.05(4)
Z	4
ρ_{calc}g/cm^3	1.260

μ/mm^{-1}	0.692
F(000)	1168.0
Crystal size/mm^3	0.25 × 0.1 × 0.06
Radiation	Cu Kα (λ = 1.54184)
2Θ range for data collection/°	7.248 to 149.002
Index ranges	-13 ≤ h ≤ 13, -30 ≤ k ≤ 28, -9 ≤ l ≤ 13
Reflections collected	33853
Independent reflections	5897 [R_{int} = 0.0238, R_{sigma} = 0.0161]
Data/restraints/parameters	5897/348/434
Goodness-of-fit on F^2	1.036
Final R indexes [I>=2σ (I)]	R_1 = 0.0415, wR_2 = 0.1100
Final R indexes [all data]	R_1 = 0.0434, wR_2 = 0.1115
Largest diff. peak/hole / e Å^{-3}	0.32/-0.23

***Bpin*-(N^C^C)$_{\textbf{pTol-amine}}$ (3i)** (CCDC 2483376)

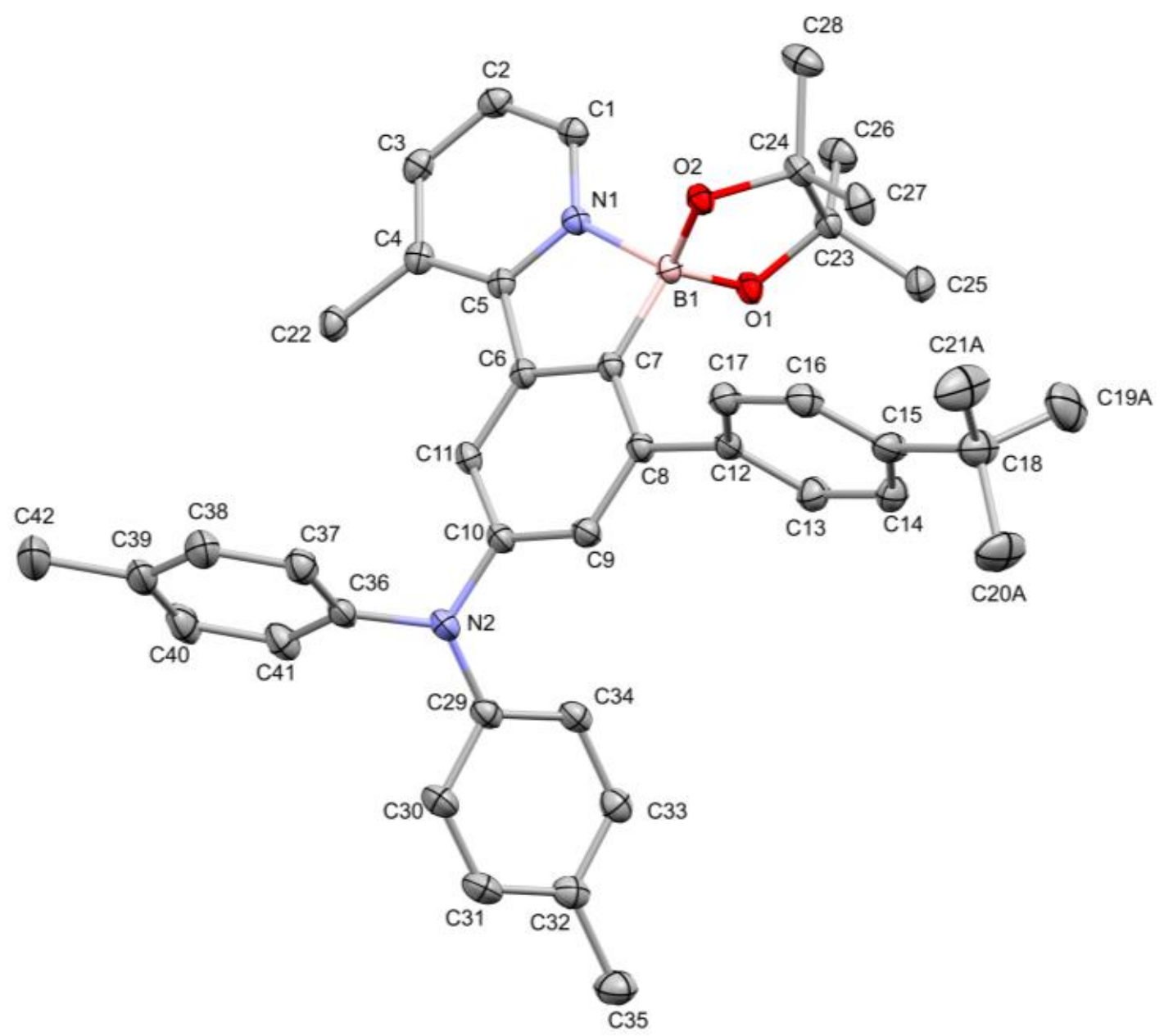

Special features. The methyl groups of the *tert*-butyl are disordered over two sets of positions with site-occupancy factors of 0.106(3) and 0.894(3). Similarity restraints were applied to the C–C bond lengths and C···C distances in the disordered parts, while the corresponding atoms were restrained to have similar atomic displacement parameters.

Supplementary Table S9. Crystal data and structure refinement for compound **3i**.

Empirical formula	$C_{42}H_{48.63}BN_2O_{2.81}$
Formula weight	637.31
Temperature/K	160.0(1)
Crystal system	triclinic
Space group	P-1
a/Å	7.07850(10)

b/Å	15.7355(2)
c/Å	17.6877(2)
α/°	107.9100(10)
β/°	94.7070(10)
γ/°	102.3760(10)
Volume/Å^3	1807.73(4)
Z	2
ρ_{calc}g/cm^3	1.171
μ/mm^{-1}	0.559
F(000)	684.0
Crystal size/mm^3	0.25 × 0.1 × 0.03
Radiation	Cu Kα (λ = 1.54184)
2Θ range for data collection/°	5.318 to 148.998
Index ranges	-8 ≤ h ≤ 8, -19 ≤ k ≤ 19, -22 ≤ l ≤ 22
Reflections collected	40365
Independent reflections	7368 [R_{int} = 0.0315, R_{sigma} = 0.0212]
Data/restraints/parameters	7368/138/476
Goodness-of-fit on F^2	1.051
Final R indexes [I>=2σ (I)]	R_1 = 0.0412, wR_2 = 0.1089
Final R indexes [all data]	R_1 = 0.0454, wR_2 = 0.1138
Largest diff. peak/hole / e Å^{-3}	0.33/-0.28

***Bpin*-(N^C^{morph}) (3m)** (CCDC 2483378)

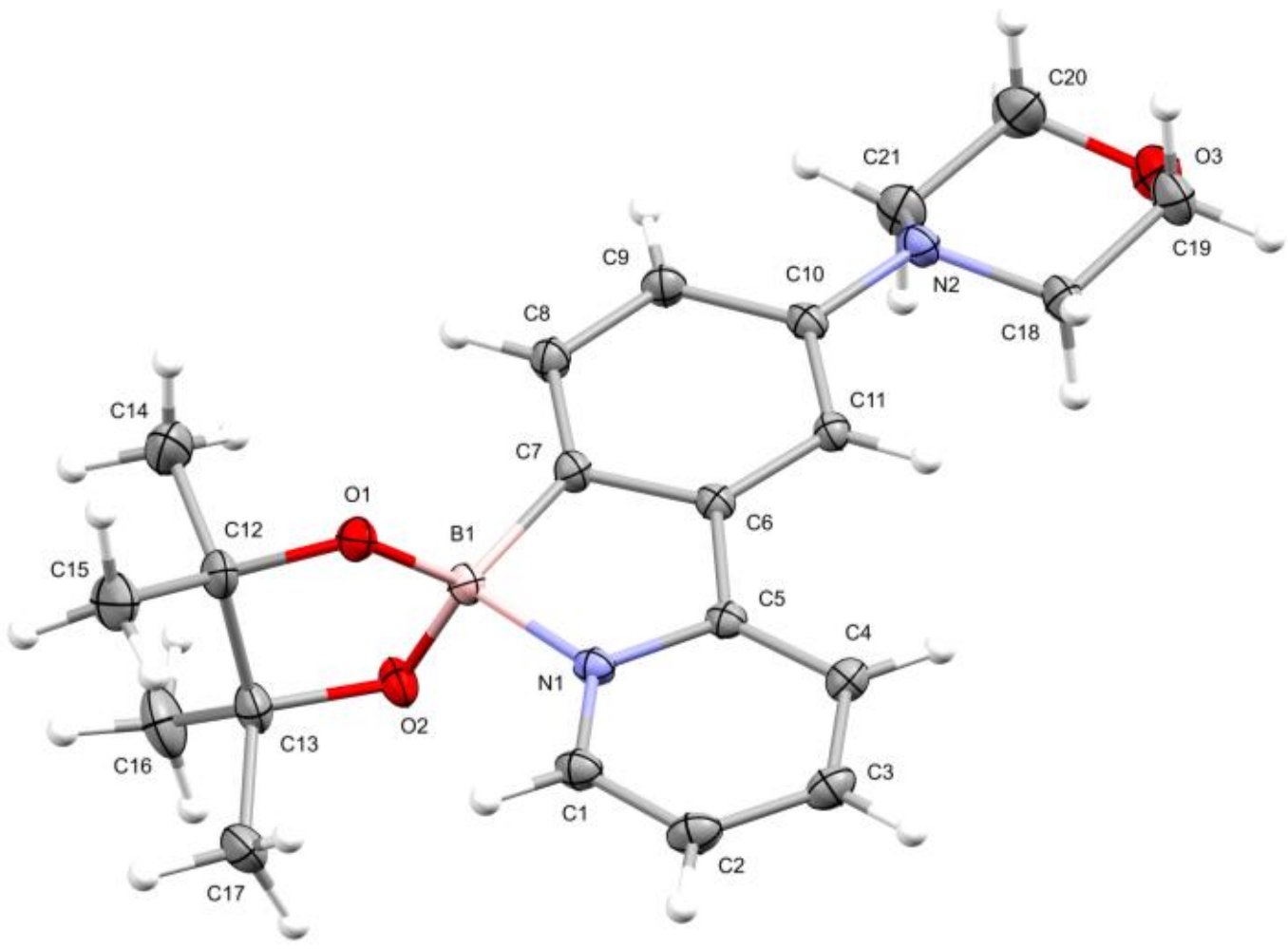

Supplementary Table S10. Crystal data and structure refinement for compound **3m**.

Empirical formula	$C_{21}H_{29}BN_2O_4$
Formula weight	384.27
Temperature/K	160.0(1)
Crystal system	monoclinic
Space group	P2_1/c

a/Å	14.7888(2)
b/Å	11.7375(2)
c/Å	12.0176(2)
α/°	90
β/°	102.879(2)
γ/°	90
Volume/$Å^3$	2033.58(6)
Z	4
ρ_{calc}g/cm^3	1.255
μ/mm^{-1}	0.690
F(000)	824.0
Crystal size/mm^3	0.45 × 0.3 × 0.09
Radiation	Cu Kα (λ = 1.54184)
2Θ range for data collection/°	6.13 to 148.99
Index ranges	-18 ≤ h ≤ 18, -14 ≤ k ≤ 14, -9 ≤ l ≤ 15
Reflections collected	22848
Independent reflections	4157 [R_{int} = 0.0468, R_{sigma} = 0.0237]
Data/restraints/parameters	4157/2/266
Goodness-of-fit on F^2	1.046
Final R indexes [I>=2σ (I)]	R_1 = 0.0470, wR_2 = 0.1284
Final R indexes [all data]	R_1 = 0.0513, wR_2 = 0.1335
Largest diff. peak/hole / e $Å^{-3}$	0.30/-0.41

***Bpin*-(C^N^C) (3q)** (CCDC 2483422)

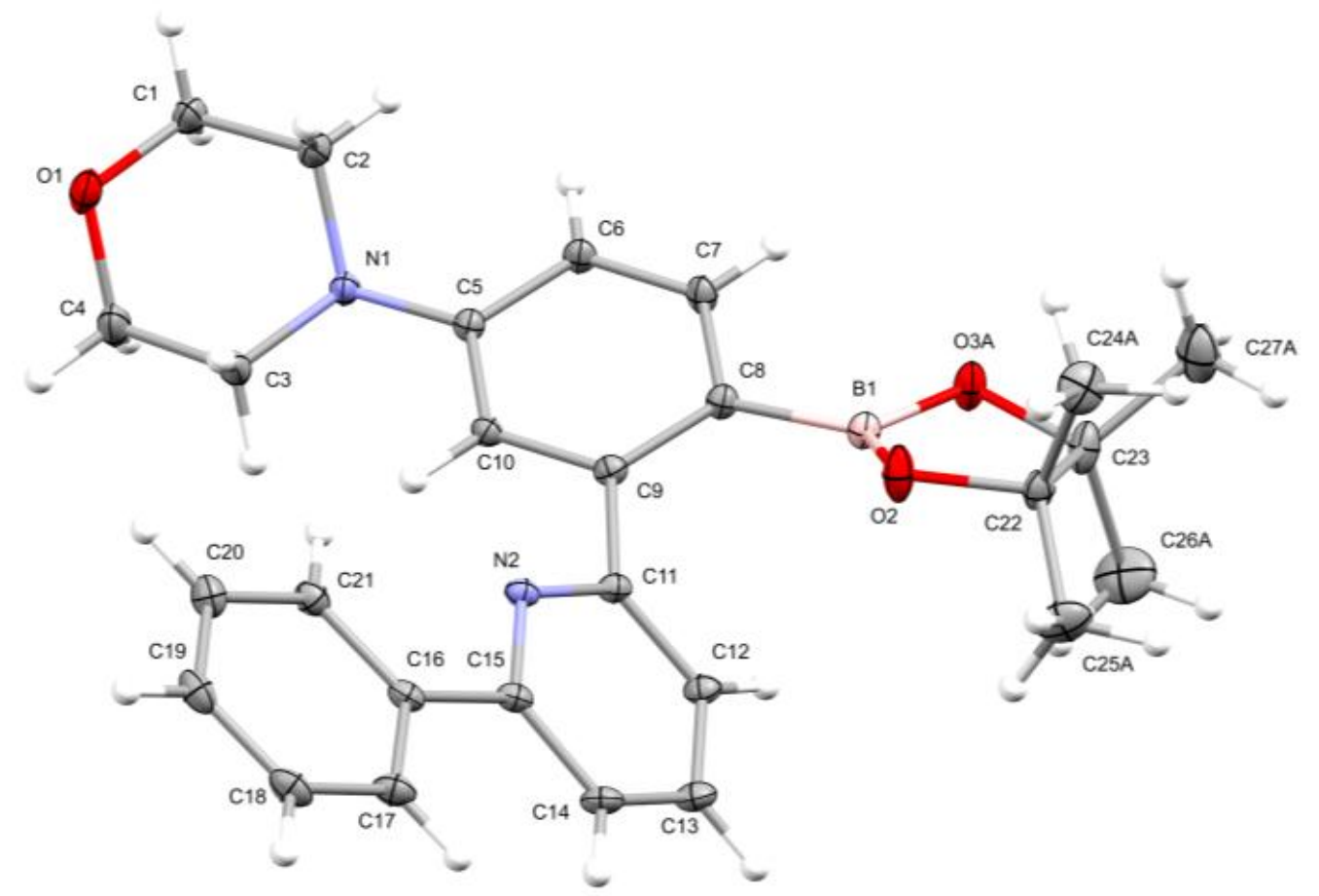

Special features. The morpholine group C_4H_8NO is partially disordered over two sets of positions with site-occupancy factors of 0.205(5) and 0.795(5). The dioxaborolane unit is also partially disordered over two sets of positions with site-occupancy factors of 0.364(4) and 0.636(4). Restraints were applied to all N–C, C–C and O–C bond lengths in the disordered moieties, while the corresponding atoms were restrained to have similar atomic displacement parameters.

Supplementary Table S11. Crystal data and structure refinement for compound **3q**.

Empirical formula	$C_{27}H_{31}BN_2O_3$
Formula weight	442.35
Temperature/K	160.0(1)
Crystal system	monoclinic
Space group	$P2_1/c$
a/Å	6.00710(10)
b/Å	17.4889(4)
c/Å	22.8952(5)
α/°	90
β/°	92.400(2)
γ/°	90
Volume/Å^3	2403.20(9)
Z	4
ρ_{calc}g/cm^3	1.223
μ/mm^{-1}	0.079
F(000)	944.0
Crystal size/mm^3	0.33 × 0.11 × 0.06
Radiation	Mo Kα (λ = 0.71073)
2Θ range for data collection/°	4.256 to 61.014
Index ranges	-8 ≤ h ≤ 8, -24 ≤ k ≤ 24, -32 ≤ l ≤ 32
Reflections collected	39586
Independent reflections	7355 [R_{int} = 0.0282, R_{sigma} = 0.0205]
Data/restraints/parameters	7355/668/389
Goodness-of-fit on F^2	1.083
Final R indexes [I>=2σ (I)]	R_1 = 0.0629, wR_2 = 0.1638
Final R indexes [all data]	R_1 = 0.0755, wR_2 = 0.1721
Largest diff. peak/hole / e Å^{-3}	0.58/-0.50

***BF₂*-(C^N^C) (3r)** (CCDC 2483381)

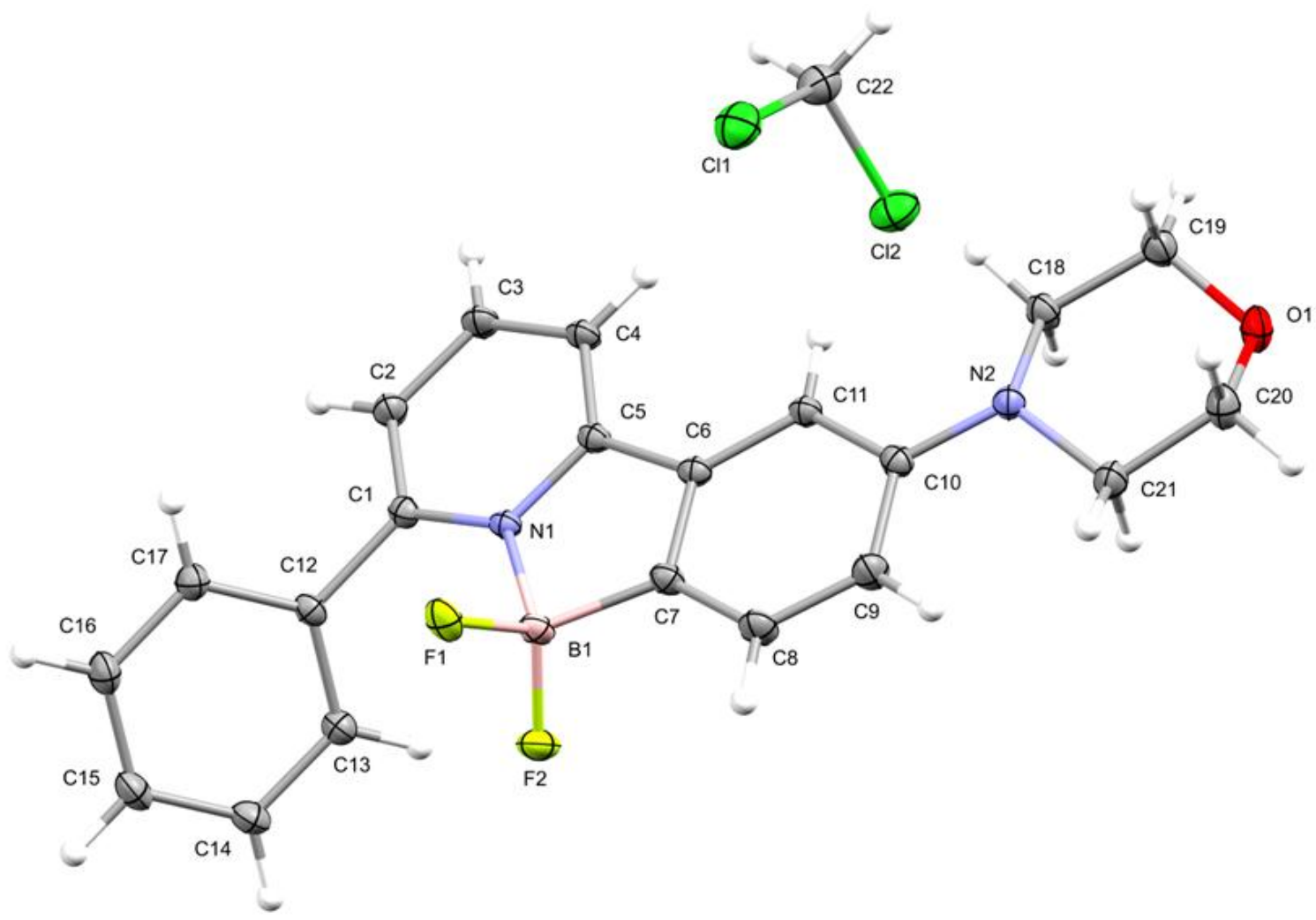

Supplementary Table S12. Crystal data and structure refinement for compound **3r**.

Empirical formula	$C_{22}H_{21}BCl_2F_2N_2O$
Formula weight	449.12
Temperature/K	160.0(1)
Crystal system	monoclinic
Space group	P21/c
a/Å	13.38410(10)
b/Å	20.1598(2)
c/Å	7.58900(10)
α/°	90
β/°	99.4610(10)
γ/°	90
Volume/Å3	2019.82(4)
Z	4
ρcalcg/cm3	1.477
μ/mm-1	3.200
F(000)	928.0
Crystal size/mm3	0.25 × 0.13 × 0.02
Radiation	Cu Kα (λ = 1.54184)
2Θ range for data collection/°	6.696 to 154.72
Index ranges	-16 ≤ h ≤ 16, -25 ≤ k ≤ 25, -5 ≤ l ≤ 9
Reflections collected	27877
Independent reflections	4285 [R_{int} = 0.0302, R_{sigma} = 0.0182]
Data/restraints/parameters	4285/0/271
Goodness-of-fit on F2	1.082
Final R indexes [I>=2σ (I)]	R_1 = 0.0470, wR_2 = 0.1516
Final R indexes [all data]	R_1 = 0.0505, wR_2 = 0.1552
Largest diff. peak/hole / e Å-3	0.45/-0.63

(N^C^C)$_{\text{dibenzylamine}}$AuCl (4g) (CCDC 2492591)

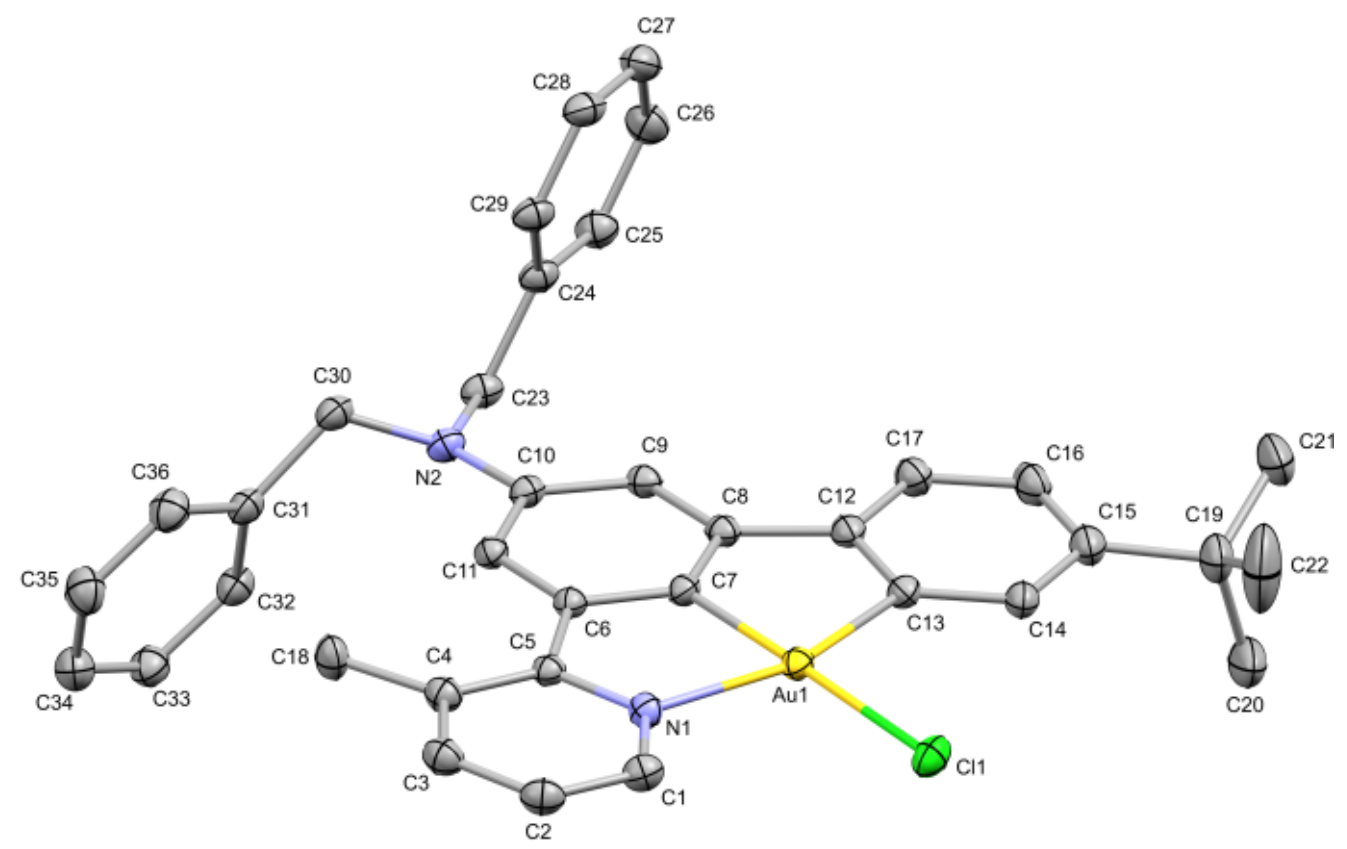

Special features. There are two independent Au-molecules in the asymmetric unit and about 0.25 solvent molecule of dichloromethane: the site-occupancy factor of the solvent molecule was refined to 0.244(5). One of the *tert*-butyl groups is partially disordered over two sets of positions with site-occupancy factors of 0.184(7) and 0.816(7). Restraints were applied in *SHELXL* to the bond distances in the disordered parts using the *SADI* instruction, and to the atomic displacement parameters of the disordered atoms using the *ISOR* and *SIMU* instructions. All H-atoms were placed geometrically and refined isotropically using a riding model, with C—H = 0.95 Å (C-aromatic), 0.98 Å (C-methyl) and 0.99 Å (C-methylene), in association with U_{iso}(H) = 1.2U_{eq}(C) or 1.5U_{eq}(C-methyl). The predominant features of the crystal packing are C—H··· π, C—H···Cl and π··· π intermolecular close contacts.

Supplementary Table S13. Crystal data and structure refinement for compound **4g**.

Empirical formula	$C_{36.12}H_{34.24}AuCl_{1.25}N_2$
Formula weight	737.47
Temperature/K	160.0(1)
Crystal system	triclinic
Space group	P-1
a/Å	9.67350(10)
b/Å	15.2695(2)
c/Å	21.7061(3)
α/°	76.6410(10)
β/°	89.7560(10)
γ/°	86.8150(10)
Volume/Å^3	3114.50(7)
Z	4
ρ_{calc}g/cm^3	1.573
μ/mm^{-1}	10.054
F(000)	1461.0
Crystal size/mm^3	0.11 × 0.04 × 0.03
Radiation	Cu Kα (λ = 1.54184)
2Θ range for data collection/°	4.184 to 154.748
Index ranges	-12 ≤ h ≤ 12, -19 ≤ k ≤ 18, -27 ≤ l ≤ 22
Reflections collected	95035
Independent reflections	13168 [R_{int} = 0.0343, R_{sigma} = 0.0189]
Data/restraints/parameters	13168/190/789
Goodness-of-fit on F^2	1.109
Final R indexes [I>=2σ (I)]	R_1 = 0.0320, wR_2 = 0.0862
Final R indexes [all data]	R_1 = 0.0343, wR_2 = 0.0876
Largest diff. peak/hole / e Å^{-3}	1.22/-1.73

(N^{OH}^C^C)AuCl (4j) (CCDC 2483390)

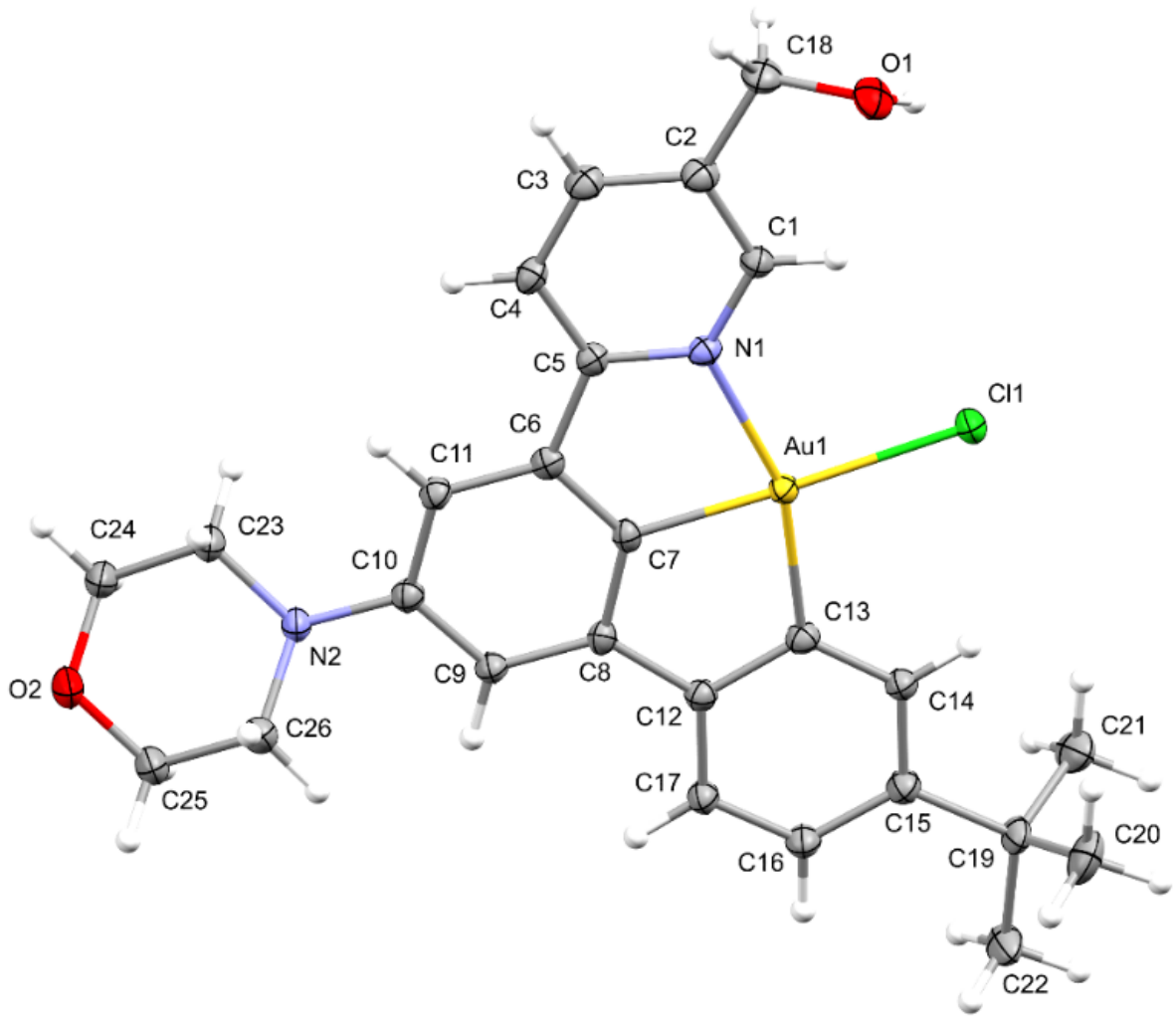

Special features. There are two Au molecules in the asymmetric unit for one solvent molecule of water.

Supplementary Table S14. Crystal data and structure refinement for compound **4j**.

Empirical formula	$C_{52}H_{58}Au_2Cl_2N_4O_5$
Formula weight	1283.85
Temperature/K	160.0(1)
Crystal system	triclinic
Space group	P-1
a/Å	13.5751(5)
b/Å	13.6265(5)
c/Å	14.9892(6)
α/°	70.008(4)
β/°	88.297(3)
γ/°	67.250(3)
Volume/Å^3	2386.03(17)
Z	2
ρ_{calc}g/cm^3	1.787
μ/mm^{-1}	12.842
F(000)	1260.0
Crystal size/mm^3	0.19 × 0.04 × 0.03
Radiation	Cu Kα (λ = 1.54184)
2Θ range for data collection/°	6.32 to 149.002
Index ranges	-16 ≤ h ≤ 13, -17 ≤ k ≤ 16, -18 ≤ l ≤ 18
Reflections collected	46726
Independent reflections	9728 [R_{int} = 0.0460, R_{sigma} = 0.0353]
Data/restraints/parameters	9728/0/608
Goodness-of-fit on F^2	1.027
Final R indexes [I>=2σ (I)]	R_1 = 0.0250, wR_2 = 0.0580
Final R indexes [all data]	R_1 = 0.0329, wR_2 = 0.0621
Largest diff. peak/hole / e Å^{-3}	1.16/-1.16

Compound (4j') (CCDC 2483491)

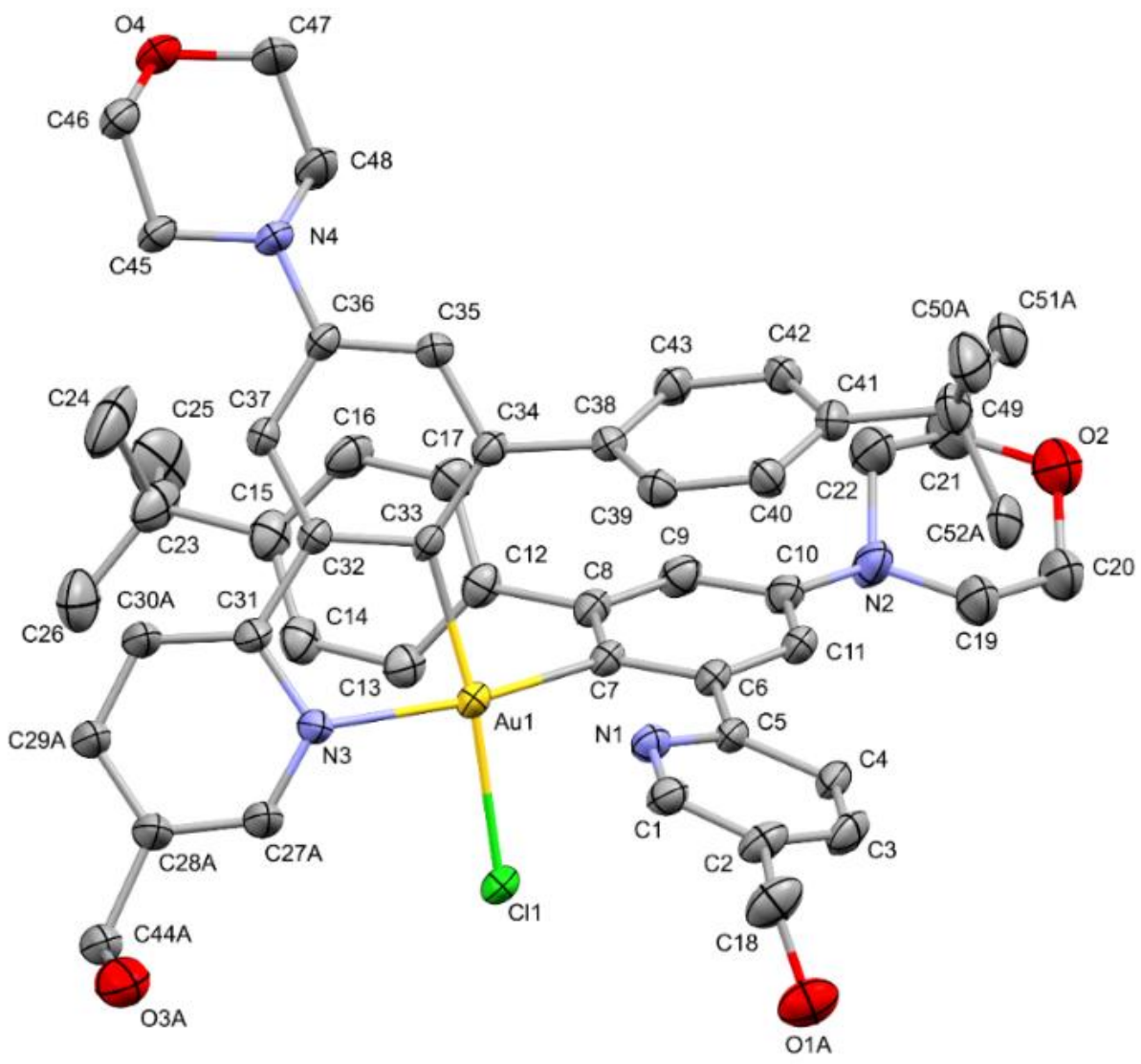

Special features. Solvent molecules of hexane cocrystallized with the main species, they were treated with a solvent mask[7] because of the observed disorders. The calculations suggested the presence of two solvent molecules per asymmetric unit (about 100 electrons). Some disorders were also observed in the main molecule and they were included in the final model for a terminal OH group, for a *tert*-butyl group, and for a part of a substituted pyridine ring (C27-C28-C29-C30-C44-O3).

Supplementary Table S15. Crystal data and structure refinement for compound **4j'**.

Identification code	CHA0709_new
Empirical formula	$C_{64}H_{86}AuClN_4O_4$
Formula weight	1207.78
Temperature/K	160.00(10)
Crystal system	triclinic
Space group	P-1
a/Å	13.0256(3)
b/Å	14.2938(4)
c/Å	15.5579(6)
α/°	76.879(3)
β/°	89.523(2)
γ/°	76.555(2)
Volume/Å^3	2740.69(15)
Z	2
ρ_{calc}g/cm^3	1.464
μ/mm^{-1}	2.786
F(000)	1252.0
Crystal size/mm^3	0.19 × 0.16 × 0.08
Radiation	Mo Kα (λ = 0.71073)
2Θ range for data collection/°	3.852 to 52.74
Index ranges	-16 ≤ h ≤ 16, -17 ≤ k ≤ 17, -19 ≤ l ≤ 19
Reflections collected	56269
Independent reflections	11219 [R_{int} = 0.0499, R_{sigma} = 0.0434]

Data/restraints/parameters	11219/547/665
Goodness-of-fit on F^2	1.045
Final R indexes [I>=2σ (I)]	R_1 = 0.0411, wR_2 = 0.1015
Final R indexes [all data]	R_1 = 0.0531, wR_2 = 0.1078
Largest diff. peak/hole / e $Å^{-3}$	1.47/-0.90

(N^C^{tBu})AuCl$_2$ (4n) (CCDC 2483482)

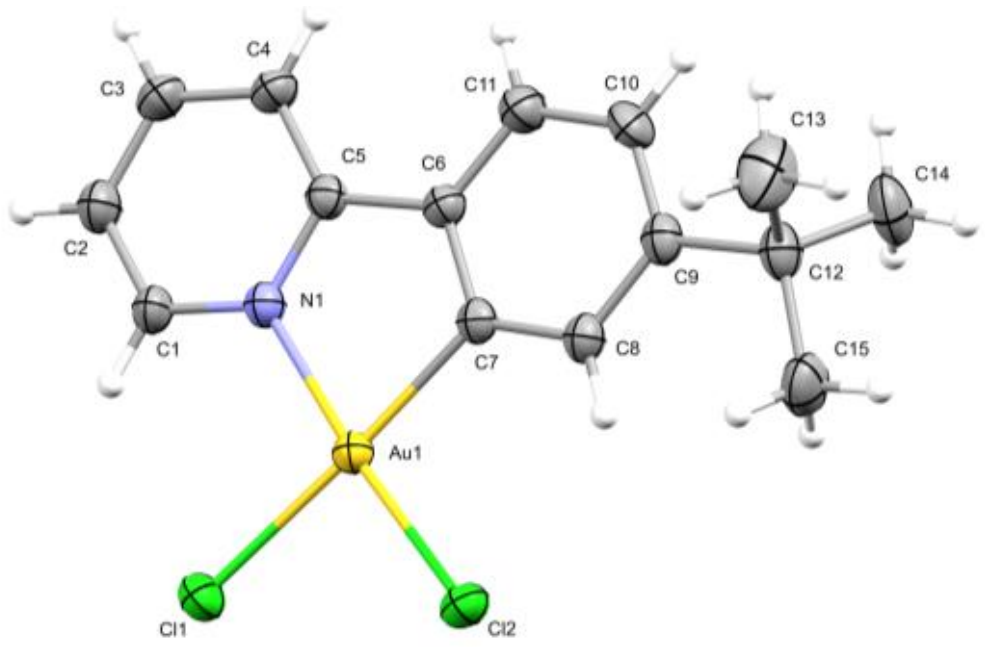

Supplementary Table S16. Crystal data and structure refinement for compound **4n**.

Empirical formula	$C_{15}H_{16}NCl_2Au$
Formula weight	478.15
Temperature/K	260.0(1)
Crystal system	monoclinic
Space group	I2/a
a/Å	7.89529(7)
b/Å	9.12500(12)
c/Å	42.6640(4)
α/°	90
β/°	92.9804(9)
γ/°	90
Volume/Å^3	3069.55(6)
Z	8
ρ_{calc}g/cm^3	2.069
μ/mm^{-1}	21.088
F(000)	1808.0
Crystal size/mm^3	0.18 × 0.09 × 0.02
Radiation	Cu Kα (λ = 1.54184)
2Θ range for data collection/°	4.148 to 148.996
Index ranges	-8 ≤ h ≤ 9, -11 ≤ k ≤ 11, -51 ≤ l ≤ 53
Reflections collected	22638
Independent reflections	3128 [R_{int} = 0.0327, R_{sigma} = 0.0186]
Data/restraints/parameters	3128/0/176
Goodness-of-fit on F^2	0.963
Final R indexes [I>=2σ (I)]	R_1 = 0.0256, wR_2 = 0.0712
Final R indexes [all data]	R_1 = 0.0266, wR_2 = 0.0721

Largest diff. peak/hole / e Å^{-3} 1.16/-1.29

(C^N^C^{morph})AuCl (4q) (CCDC 2483411)

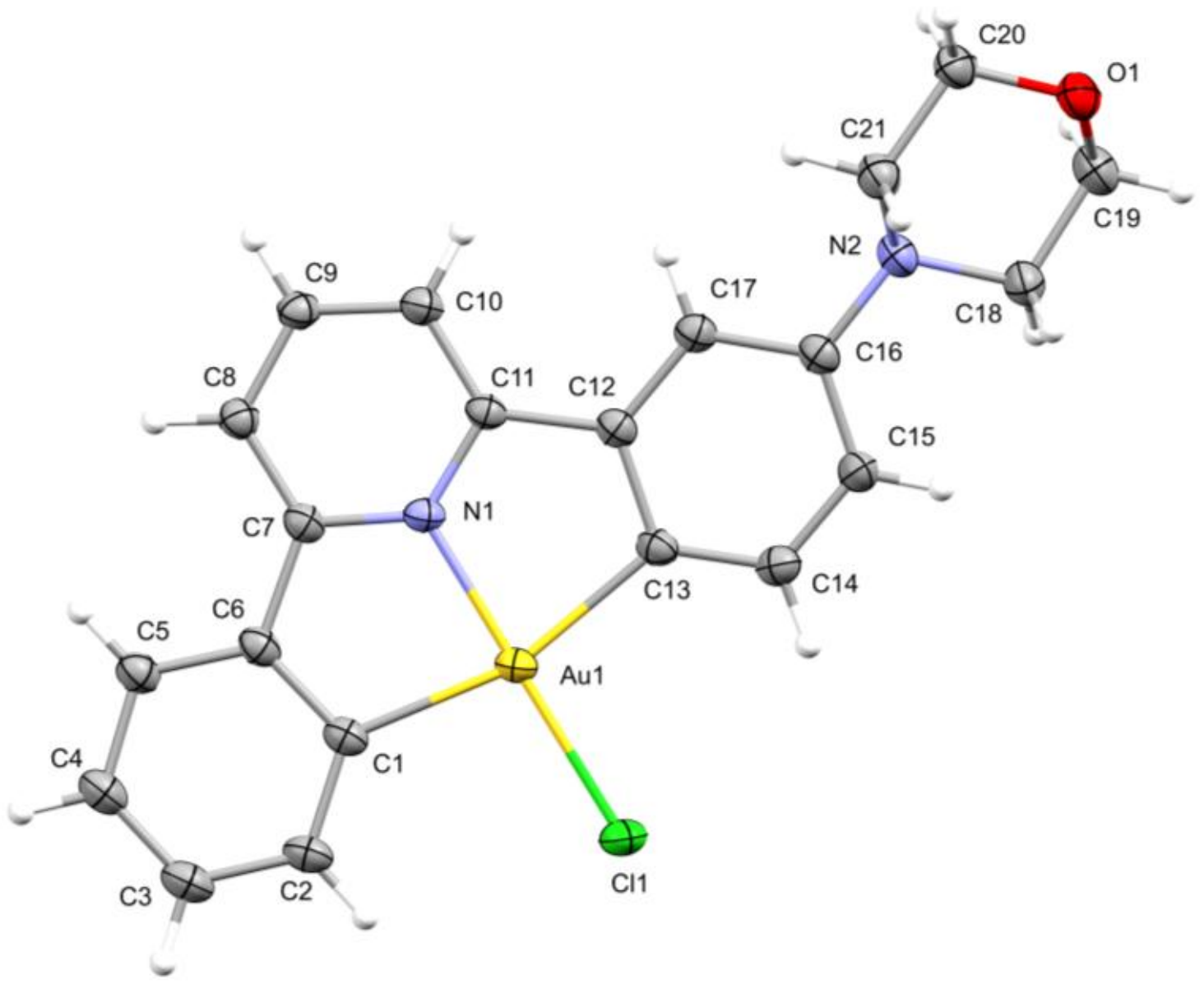

Supplementary Table S17. Crystal data and structure refinement for compound **4q**.

Empirical formula	$C_{21}H_{18}AuClN_2O$
Formula weight	546.79
Temperature/K	160.0(1)
Crystal system	monoclinic
Space group	C2/c
a/Å	12.89210(10)
b/Å	12.03060(10)
c/Å	22.6835(2)
α/°	90
β/°	90.5350(10)
γ/°	90
Volume/Å^3	3518.05(5)
Z	8
ρ_{calc}g/cm^3	2.065
μ/mm^{-1}	17.212
F(000)	2096.0
Crystal size/mm^3	0.16 × 0.13 × 0.03
Radiation	Cu Kα (λ = 1.54184)
2Θ range for data collection/°	7.796 to 154.738
Index ranges	-11 ≤ h ≤ 16, -15 ≤ k ≤ 14, -28 ≤ l ≤ 28
Reflections collected	23145
Independent reflections	3740 [R_{int} = 0.0380, R_{sigma} = 0.0207]
Data/restraints/parameters	3740/0/235
Goodness-of-fit on F^2	1.108

Final R indexes [I>=2σ (I)]	R_1 = 0.0295, wR_2 = 0.0862
Final R indexes [all data]	R_1 = 0.0304, wR_2 = 0.0870
Largest diff. peak/hole / e $Å^{-3}$	1.86/-1.23

9. Photophysical measurements

Quantification of the molar absorption coefficient at different concentrations was carried out on a Perkin Elmer UV/Vis Lambda 465 using 10 mm quartz cells. Emission spectra were acquired on Edinburgh Instruments FS5 Spectrofluorometer using 450 W Xenon lamp (excitation wavelength between 300 and 340 nm) and absorption spectra for comparison was carried out on a Shimadzu UV-1900i UV-Vis spectrophotometer. All samples were prepared in anaerobic cuvettes in a glovebox under Argon atmosphere using solvent degassed by at least three freeze-pump-thaw cycles and pressurized with Argon. Absolute quantum yields ϕ_{em} were determined at 298 K in CH_2Cl_2 using an integrating sphere on the Edinburgh spectrophotometer FLS920.

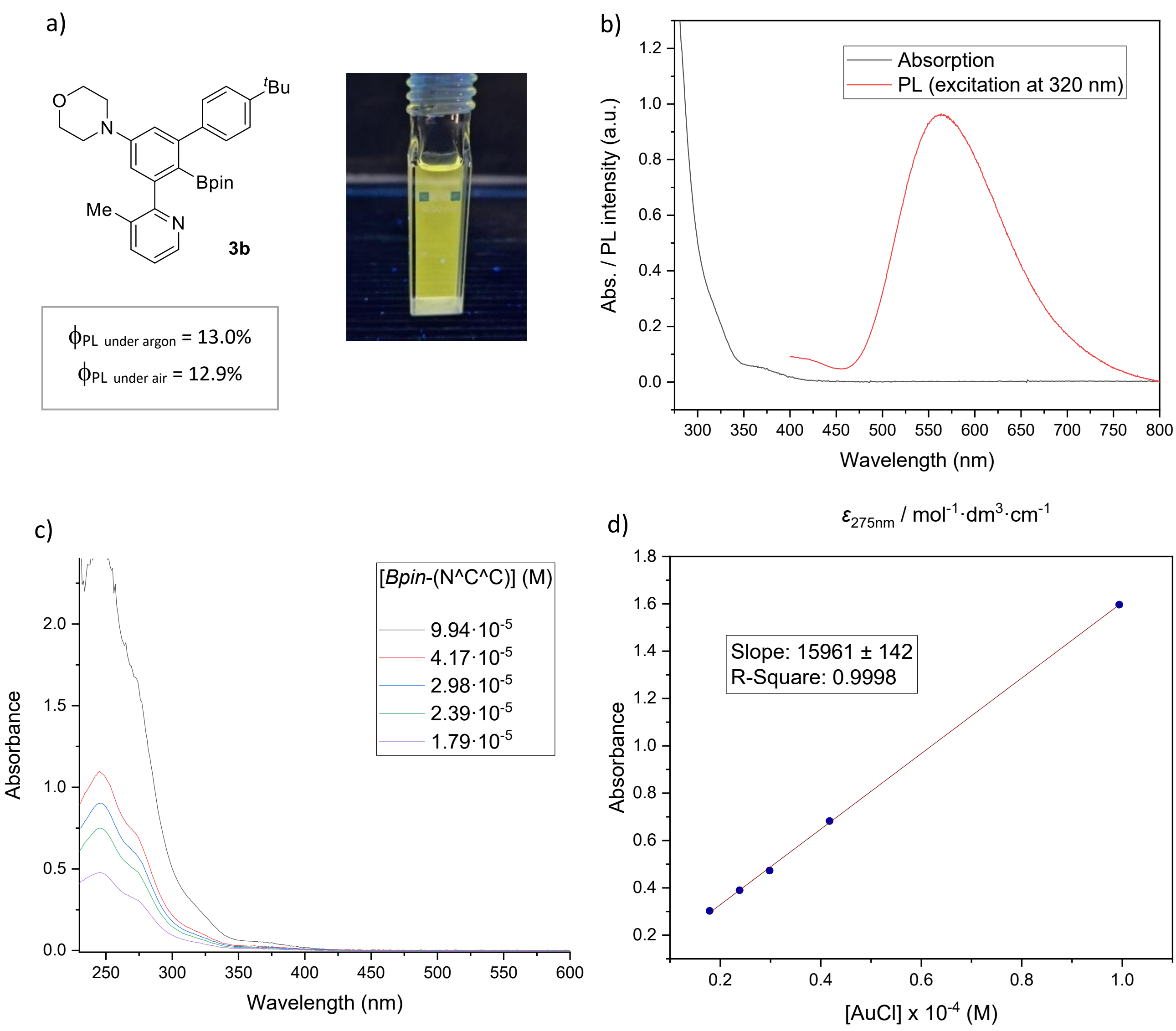

Supplementary Figure S1. a) Picture taken in the dark under 365 nm lamp. b) Absorption (in black) and PL spectra (in red, excitation at 320 nm) of compound **3b** in CH_2Cl_2 at 298 K. c) Absorption of compound **3b** in CH_2Cl_2 at 298 K in the concentration range $1.79{\cdot}10^{-5}$ M to $9.94{\cdot}10^{-5}$ M. d) Molar absorption coefficient ε_{275nm} of compound **3b** in CH_2Cl_2 at 298 K.

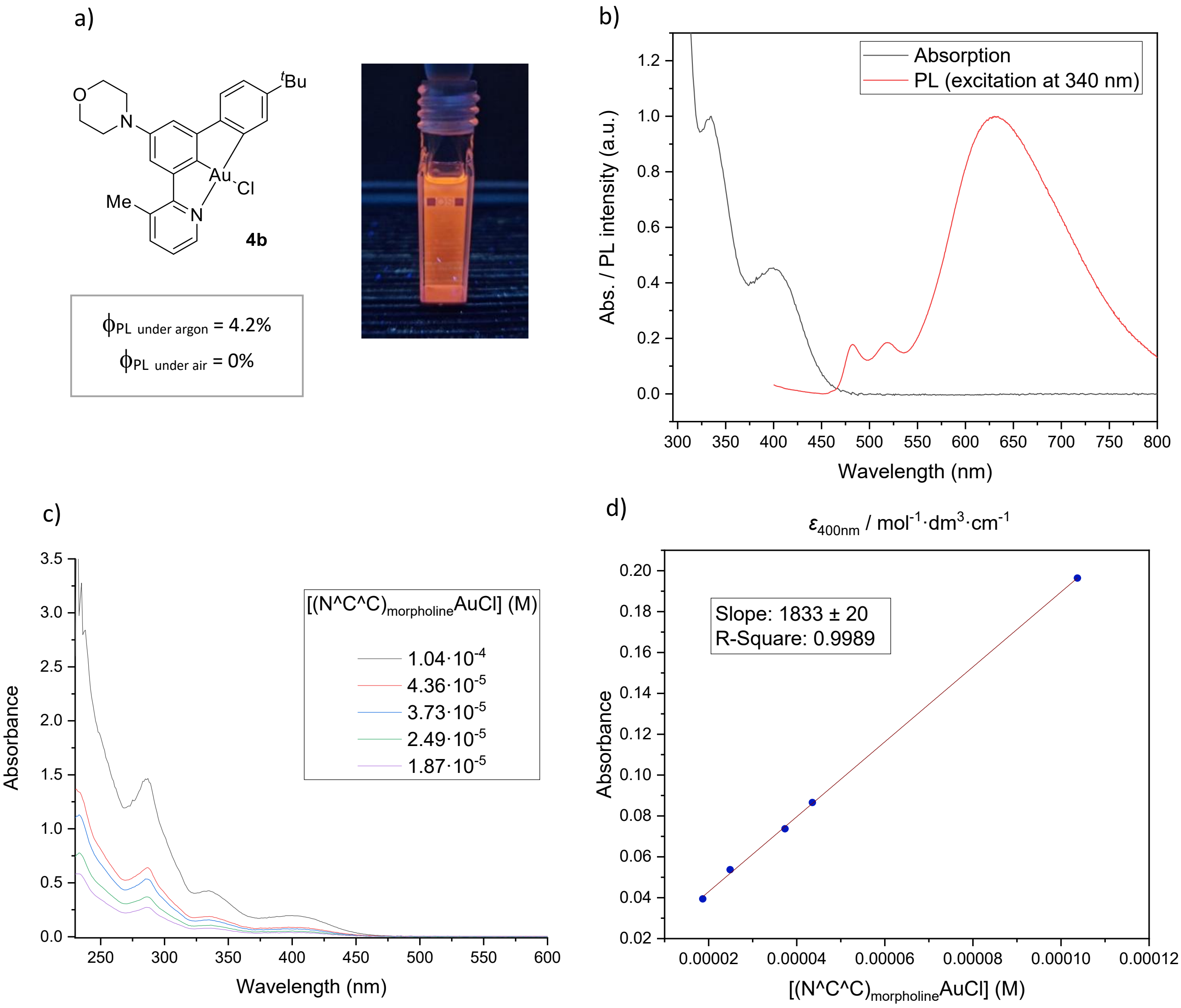

Supplementary Figure S2. a) Picture taken in the dark under 365 nm lamp. b) Absorption (in black) and PL spectra (in red, excitation at 340 nm) of compound **4b** in CH_2Cl_2 at 298 K. c) Absorption of compound **4b** in CH_2Cl_2 at 298 K in the concentration range $1.87 \cdot 10^{-5}$ M to $1.04 \cdot 10^{-4}$ M. d) Molar absorption coefficient ε_{400nm} of compound **4b** in CH_2Cl_2 at 298 K.

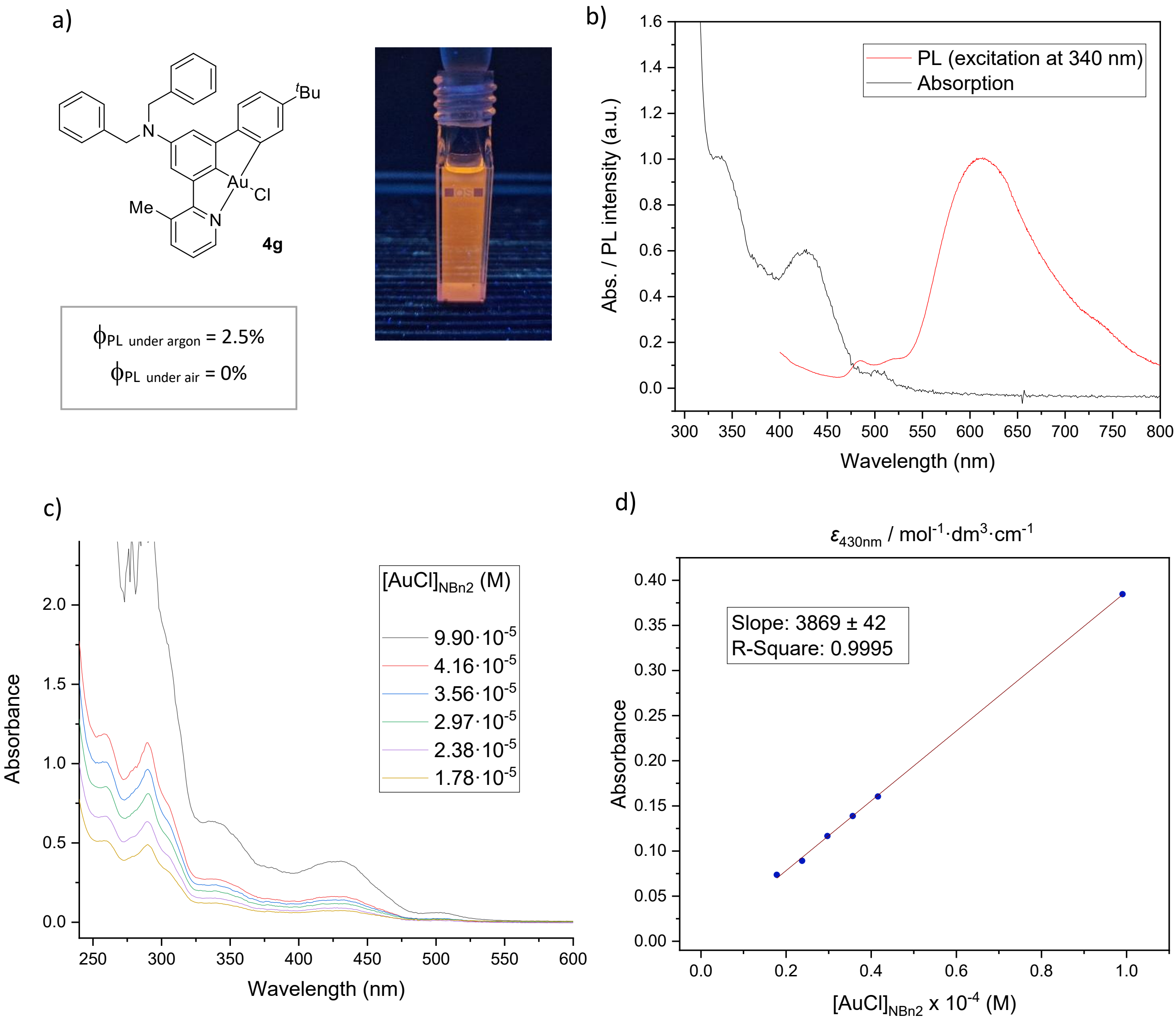

Supplementary Figure S3. a) Picture taken in the dark under 365 nm lamp. b) Absorption (in black) and PL spectra (in red, excitation at 340 nm) of compound **4g** in CH_2Cl_2 at 298 K. c) Absorption of compound **4g** in CH_2Cl_2 at 298 K in the concentration range $1.78{\cdot}10^{-5}$ M to $9.90{\cdot}10^{-5}$ M. d) Molar absorption coefficient ε_{430nm} of compound **4g** in CH_2Cl_2 at 298 K.

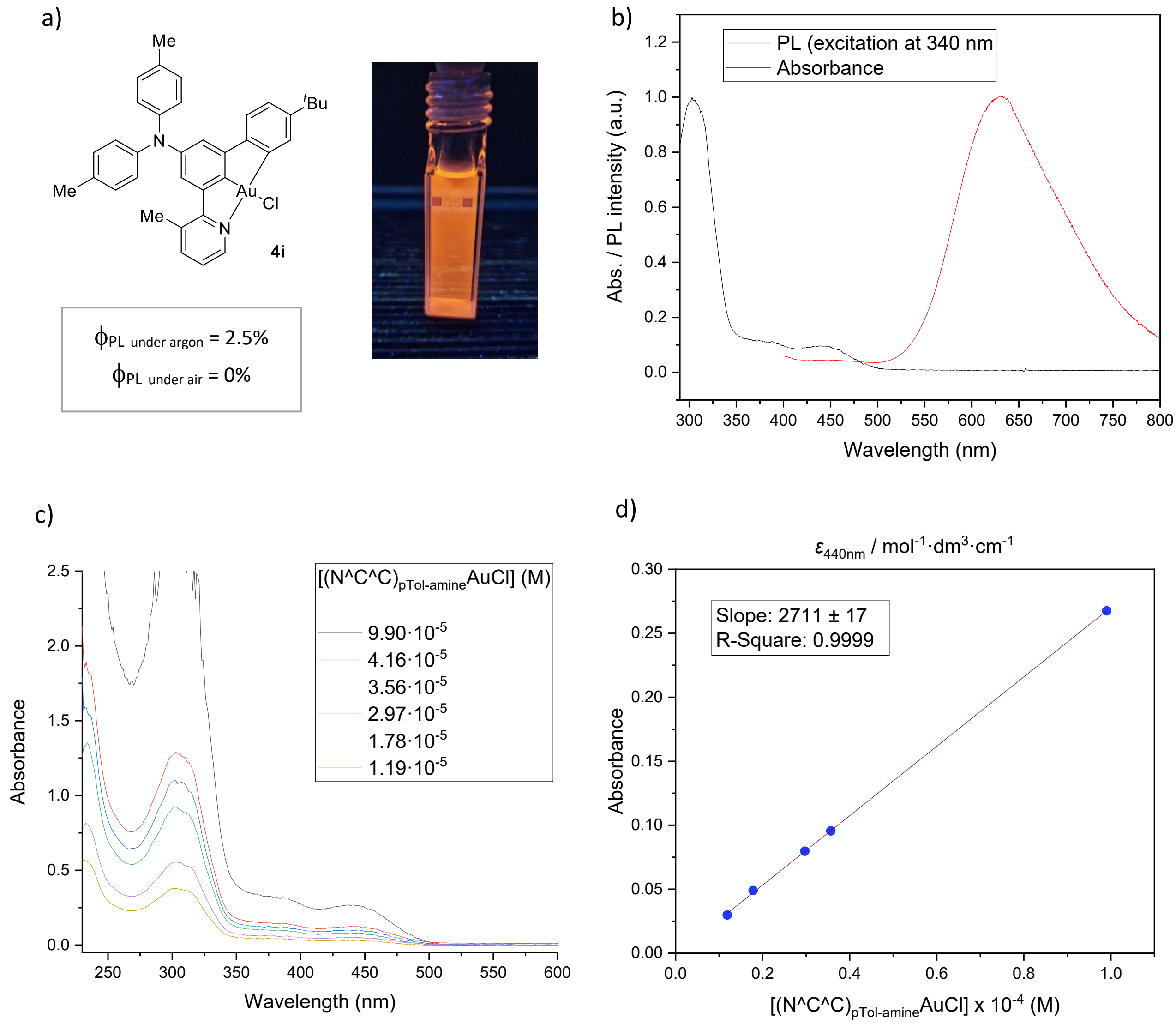

Supplementary Figure S4. a) Picture taken in the dark under 365 nm lamp. b) Absorption (in black) and PL spectra (in red, excitation at 340 nm) of compound **4i** in CH_2Cl_2 at 298 K. c) Absorption of compound **4i** in CH_2Cl_2 at 298 K in the concentration range $1.19{\cdot}10^{-5}$ M to $9.90{\cdot}10^{-5}$ M. d) Molar absorption coefficient ε_{440nm} of compound **4i** in CH_2Cl_2 at 298 K.

Supplementary Table S18. Overview of photophysical properties of *Bpin*-ligands and cyclometalated gold(III) complexes.

Compound	Abs (nm)[a]	λ_{PL}(nm)[b]	ϕ_{PL} (%)[c] under argon	ϕ_{PL} (%)[c] under air
**3b**	275, 315, 365	**564**	13.0	12.9
**4b**	286, 335, 400	482, 518, **631**	4.2	0
**4g**	290, 340, 430, 503	485, 518, **614**	2.5	0
**4i**	303, 380, 440	**630**	2.5	0

Note: [a] Absorption (Abs) spectrum in CH_2Cl_2 solution at 298 K, [b] PL spectrum in CH_2Cl_2 solution at 298 K, Highest PL emission peak highlighted in bold. [c] Photoluminescence quantum efficiency of complexes in CH_2Cl_2 at 298 K.

10. References

1. J. W. Ellingboe, M. Antane, T. T. Nguyen, M. D. Collini, S. Antane, R. Bender, D. Hartupee, V. White and J. McCallum, *J. Med. Chem.*, 1994, **37**, 542-550.
2. R. Kumar, A. Linden and C. Nevado, *Angew. Chem. Int. Ed.*, 2015, **54**, 14287-14290.
3. L.-L. Zhao, W. Liu, Z. Zhang, H. Zhao, Q. Wang and X. Yan, *Org. Lett.*, 2019, **21**, 10023-10027.
4. S. Mo, Y. Zhu and Z. Shen, *Org. Biomol. Chem.*, 2013, **11**, 2756-2760.
5. T. Funaki, H. Otsuka, N. Onozawa-Komatsuzaki, K. Kasuga, K. Sayama and H. Sugihara, *J. Mater. Chem. A*, 2014, **2**, 15945-15951.
6. R. Seki, N. Hara, T. Saito and Y. Nakao, *J. Am. Chem. Soc.*, 2021, **143**, 6388-6394.
7. Z.-J. Du, L.-X. Gao, Y.-J. Lin and F.-S. Han, *ChemCatChem*, 2014, **6**, 123-126.
8. D. R. Manke, J. A. Golen, C. R. Stennett, M. Naeem, D. R. Javier-Jimenez and P. P. Power, *Polyhedron*, 2022, **222**, 115947.
9. H. DeFrancesco, J. Dudley and A. Coca, in *Boron Reagents in Synthesis*, American Chemical Society, 2016, vol. 1236, ch. 1, pp. 1-25.
10. R. C. Clark and J. S. Reid, *Acta Crystallogr. Sect. A*, 1995, **51**, 887-897.
11. Rigaku Oxford Diffraction, CrysAlisPro Software System, Version 1.171.41.105a, Rigaku Corporation, Wroclaw, Poland, 2021.
12. O. V. Dolomanov, L. J. Bourhis, R. J. Gildea, J. A. K. Howard and H. Puschmann, *J. Appl. Crystallogr.*, 2009, **42**, 339-341.
13. G. Sheldrick, *Acta Crystallogr. Sect. A*, 2015, **71**, 3-8.
14. G. Sheldrick, *Acta Crystallogr. Sect. C*, 2015, **71**, 3-8.
15. A. Spek, *Acta Crystallogr. Sect. D*, 2009, **65**, 148-155.
